# Supplementary material for: Enantioselective copper-catalyzed azidation/click cascade reaction for access to chiral 1,2,3-triazoles
Source: Nat Commun. 2024 Jun 10;15:4919. doi: 10.1038/s41467-024-49313-x (PMC11164697; doi:10.1038/s41467-024-49313-x)
Supplement: Supplementary file 1 — Supplementary Information [file 41467_2024_49313_MOESM1_ESM.pdf]

Supplementary Information

Enantioselective copper-catalyzed azidation/click cascade reaction for access to chiral 1,2,3-triazoles

Ling-Feng Jiang[1], Shao-Hua Wu[1], Yu-Xuan Jiang[1], Hong-Xiang Ma[1], Jia-Jun He[1], Yang-Bo Bi[1], De-Yi Kong[1], Yi-Fei Cheng[1], Xuan Cheng[1] and Qing-Hai Deng[1]*

E-mail: qinghaideng@shnu.edu.cn

[1] The Education Ministry Key Laboratory of Resource Chemistry, Joint International Research Laboratory of Resource Chemistry of Ministry of Education, Shanghai Key Laboratory of Rare Earth Functional Materials, and Shanghai Frontiers Science Center of Biomimetic Catalysis, Shanghai Normal University, Shanghai 200234, China.

Table of Contents

Supplementary Information 1

1.Supplementary Methods 3

1.1 General Information.. 3
1.2. Optimization of Reaction Conditions 3
1.3. General Procedure for the Synthesis of Substrates **2a-2x**, **4a-4z**............ 6
1.4. General Procedure for the Synthesis of **1a-1g** 40
1.5. General Procedure for the Synthesis of Racemic Products **3** and **5**....... 44
1.6. General Procedure for the Synthesis of Chiral Products **3** and **5**........... 45
1.7. Procedure for synthesis of **3a** in gram-scale 77

2. Supplementary Discussion.. 79

2.1. Radical inhibition experiments 79
2.2. Nonlinear effect study 79
2.3. Investigation of intermediate azide.. 80
2.4. Control experiments with other substrates.. 82
2.5. DFT calculations 86

3. Supplementary Notes.. 88

3.1 Determination of the Absolute Configuration of **5b** by X-ray Analysis 88
3.2 Determination of the Absolute Configuration of **5d** by X-ray Analysis . 90
3.3 Determination of the Absolute Configuration of **5x** by X-ray Analysis . 92

4. Supplementary Figures 94

4.1 NMR Spectra of New Compounds (^{1}H NMR, ^{13}C NMR and ^{19}F NMR) 94
4.2 Chromatographic Data for Chiral Products 318

5. Supplementary References.. 373

CAUTION: It might be potentially explosive about azidating reagents, intermediates, or products. Although we did not encounter any problems under the conditions and scale described here, appropriate precautions should be taken when handling these compounds. A blast shield was necessary while the azidation reactions and subsequent workups were performed.

1.Supplementary Methods

1.1 General Information

All manipulations were maintained under an atmosphere of nitrogen unless otherwise stated. Commercially available reagents were used without further purification. Solvents were pre-dried over activated 4 Å molecular sieves and were refluxed over sodium-benzophenone (toluene, tetrahydrofuran), phosphorus pentoxide (chloroform) or calcium hydride (dichloromethane, dichloroethane, acetonitrile). Column chromatography was performed on silica gel (200-300 mesh). ^{1}H NMR spectra were recorded on a 400 or 600 MHz NMR spectrometer and ^{13}C NMR spectra were recorded on a 101 MHz NMR spectrometer. Infrared spectra were prepared as KBr pellets and were recorded on a Varian Excalibur 3100 series FT-IR spectrometer. Mass spectra were recorded by the mass spectrometry service of Shanghai Institute of Organic Chemistry. HPLC analyses on a Waters 1596 or Shimadzu SPD-15C. Optical rotations were measured with Rudolph Research Analytical in a 1 dm cuvette.I.

1.2. Optimization of Reaction Conditions

Supplementary Table 1. Optimization of the Reaction Conditions (Ligand) for the Synthesis of Product **3a**[a]

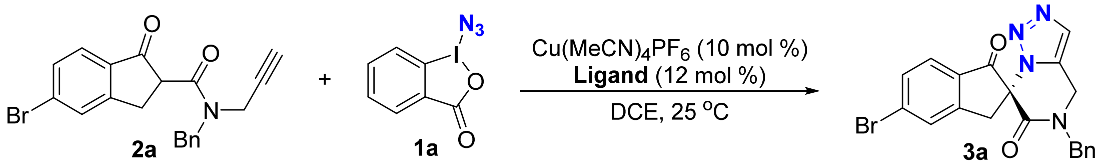

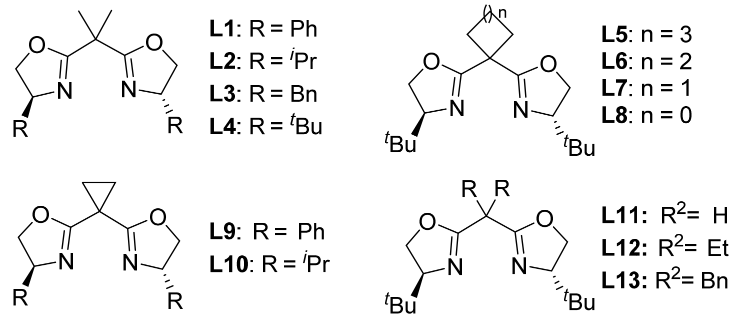

Entry	L	Time (h)	Yield%[b]	Ee%[c]

1	**L1**	24	35	14
2	**L2**	24	28	12
3	**L3**	24	50	23
4	**L4**	24	37	75
5	**L5**	24	44	44
6	**L6**	24	40	82
7	**L7**	24	45	80
**8**	**L8**	**24**	**62**	**92**
9	**L9**	24	47	33
10	**L10**	24	52	31
11	**L11**	24	47	14
12	**L12**	24	68	81
13	**L13**	24	42	32

[a]Reaction conditions: **2a** (0.10 mmol), **1a** (1.5 equiv), $Cu(MeCN)_4PF_6$ (10 mol %), **Ligand** (12 mol %), DCE (2.0 mL), 25 °C, nitrogen. [b]The yields of isolated products. [c]Determined by HPLC analysis.

Supplementary Table 2. Optimization of the Reaction Conditions (Temperature) for the Synthesis of Product **3a**[a]

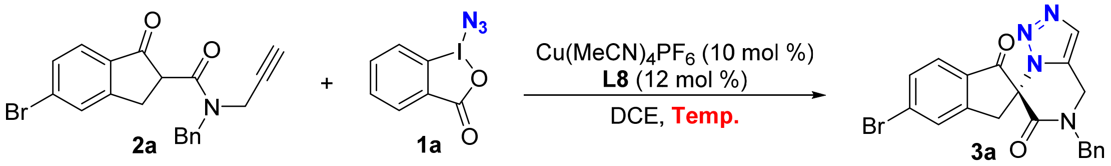

**Entry**	**Time (h)**	**Temp. (°C)**	**Yield%**[b]	**Ee%**[c]
1	24	40 °C	57	89
2	24	30 °C	62	91
**3**	**24**	**25 °C**	**62**	**92**

[a]Reaction conditions: **2a** (0.10 mmol), **1a** (1.5 equiv), $Cu(MeCN)_4PF_6$ (10 mol %), **L8** (12 mol %), DCE (2.0 mL), Temperature, nitrogen. [b]The yields of isolated products. [c]Determined by HPLC analysis.

Supplementary Table 3. Optimization of the Reaction Conditions (Solvent) for the Synthesis of Product **3a**[a]

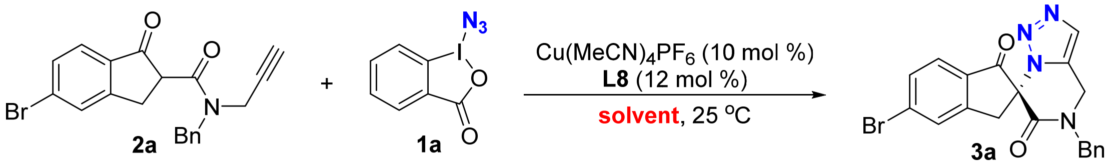

Entry	Solvent	Time (h)	Yield%[b]	Ee%[c]
1	DCE	24	62	92
**2**	**DCM**	**24**	**70**	**94**
3	MeCN	24	43	88
4	THF	24	trace	/

[a]Reaction conditions: **2a** (0.10 mmol), **1a** (1.5 equiv), $Cu(MeCN)_4PF_6$ (10 mol %), **L8** (12 mol %), Solvent (2.0 mL), 25 °C, nitrogen. [b]The yields of isolated products. [c]Determined by HPLC analysis.

Supplementary Table 4. Optimization of the "N_3" at the standard Conditions for the Synthesis of Product **3a**[a]

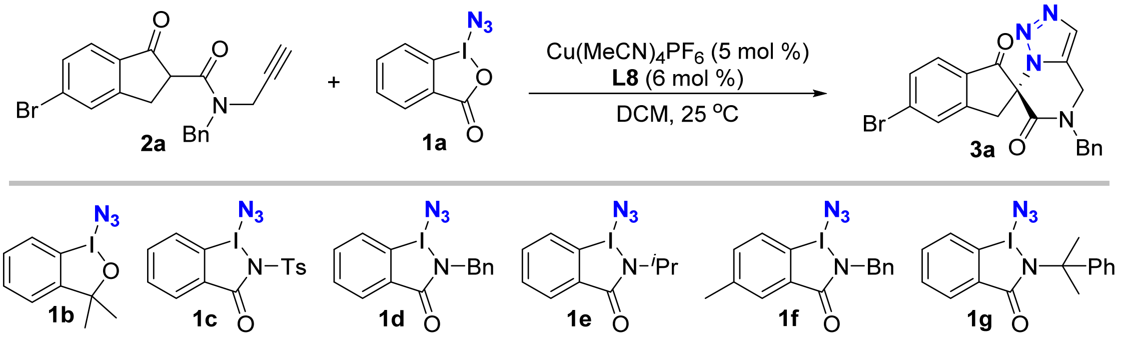

Entry	N_3	Oxidant	Time (h)	Yield%[b]	Ee%[c]
1[d]	**1a**	/	24	70	94
2	**1a**	/	24	60	94
3[d]	**1b**	/	24	30	27
4	**1c**	/	24	80	90
**5**	**1d**	/	24	**99**	**94**

6	**1e**	/	24	78	94
7	**1f**	/	24	95	93
8	**1g**	/	24	93	93
9	**$TMSN_3$**	O_2	24	trace	/
10	**$TMSN_3$**	TPHP	24	20	10
11	**$TMSN_3$**	TBPB	24	30	18
12	**$TMSN_3$**	$PhI(OAc)_2$	24	50	23

[a]Reaction conditions: **2a** (0.10 mmol), "N_3" (1.5 equiv), $Cu(MeCN)_4PF_6$ (5 mol %), **L8** (6 mol %), DCM (2.0 mL), 30 °C, nitrogen. [b]The yields of isolated products. [c]Determined by HPLC analysis. [d]10 mol % of $Cu(MeCN)_4PF_6$ and 12 mol % of **L8** were used instead.

1.3. General Procedure for the Synthesis of Substrates 2a-2x, 4a-4z

1.3.1 General Procedure for the Synthesis of Substrates **2a-2x**, **4w-4z**[1-2]

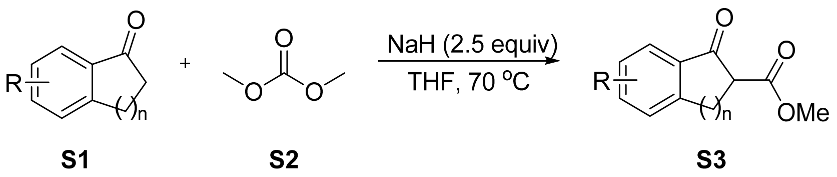

NaH (12.5 mmol, 2.5 equiv, 60% dispersion in mineral oil) was dispersed in dry tetrahydrofuran solution. Dimethyl carbonate (**S2**) (25 mmol, 5 equiv) was added at 0 °C, then **S1** (5 mmol, 1.0 equiv) was droped slowly. The mixture was stirred at 70 °C overnight. After cooling to room temperature, the mixture was quenched with HCl (aq.) and was then extracted with EtOAc (three times). The combined organic layer was washed with brine, dried over $MgSO_4$, and concentrated in vacuo. The residue was purified by column chromatography on silica gel (ethyl acetate/ petroleum ether = 1 : 8) to give the product **S3** as yellow solid.

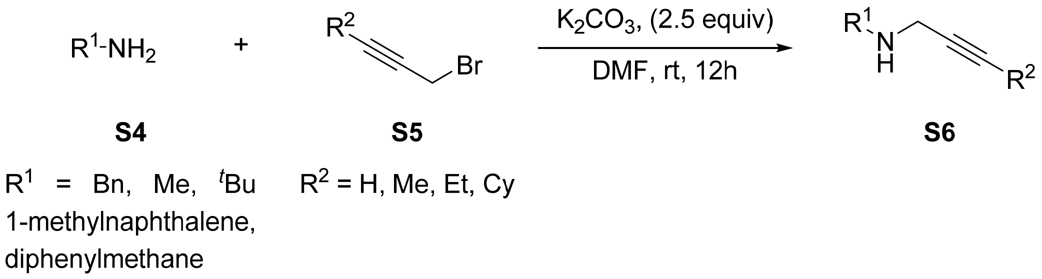

The mixture of **S4** (15 mmol, 3.0 equiv), K_2CO_3 (17.5 mmol, 2.5 equiv) and DMF (5 mL) was stirred at the room temperature for 15 min, and then **S5** (5 mmol, 1.0 equiv) in DMF (5 mL) was slowly added dropwise. The mixture was stirred at room temperature for 12 h. Once the reaction was completed, water (20 mL) was added and the mixture was extracted three times with ethyl acetate (20 mL). The combined organic layer was washed with water (20 mL) and saturated brine (20 mL), dried over anhydrous Na_2SO_4, and concentrated in vacuo. The crude mixture was purified via column chromatography with hexane/ethyl acetate (5:1) to afford **S6** as yellow oil.

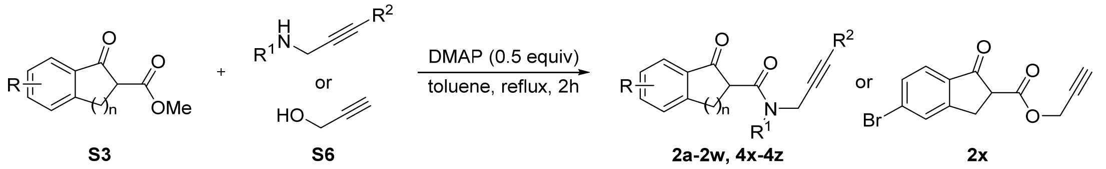

DMAP (0.5 mmol, 0.5 equiv) and **S3** (1 mmol, 1.0 equiv) were dissolved in dry toluene (3 mL), then **S6** (1.5 mmol) was added. The mixture was stirring at 110 °C for 2 h. After disappearance of **S3** (monitored by TLC), the mixture was cooled to room temperature and was quenched with HCl (aq.). The residue was extracted three times with ethyl acetate, and was then washed with $NaHCO_3$ (aq.). Finally, the crude product was purified by silica gel flash chromatography to get the desired products **2a-2x** and **4x-4z**.

1.3.2 General Procedure for the Synthesis of Substrates **4a-4v**[3-4]

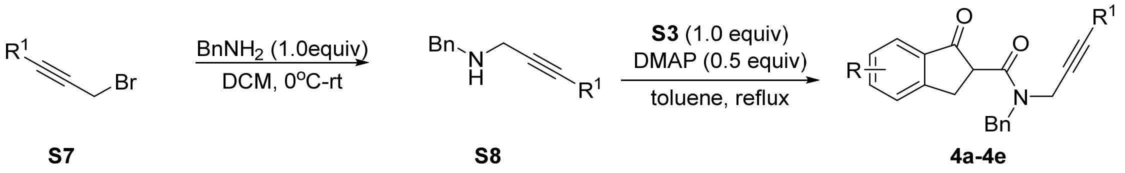

1) $BnNH_2$ (5 mmol, 1.0 equiv) was added dropwise to **S7** (30 mmol, 6 equiv) in DCM at 0 °C. Upon complete addition, the reaction was allowed to warm to room temperature and stirred over 17 h. Then, aqueous 1 M NaOH (22.5 mL) and Et_2O (22.5 mL) were added and the layers were separated. After extraction of the aqueous layer with Et_2O (2 x 25 mL), the combined organic layers were washed with brine (25 mL), dried over $MgSO_4$ and the solvent was removed under vacuo. The crude was purified by flash column chromatography to afford **S8** as a yellow oil.

2) DMAP (0.5 mmol, 0.5 equiv) and **S3** (1 mmol, 1.0 equiv) were dissolved in dry toluene (3 mL), then **S8** (1.5 mmol) was added, and the mixture was stirring at 110 °C for 2 h. After the disappearance of substrate **S8** (monitored by TLC), the mixture was cooled to room temperature and then quenched with HCl (aq.). The residue was extracted three times with ethyl acetate, and was then washed with $NaHCO_3$ (aq.). Finally, the crude product was purified by silica gel flash chromatography to afford the desired products **4a-4e**.

1.3.3 General Procedure for the Synthesis of Substrates **4f-4w**[5]

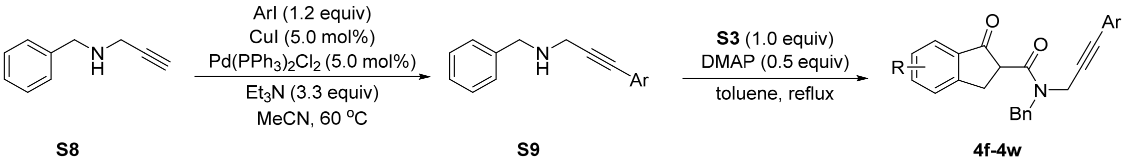

1) To a flame-dried 100 mL round bottom flask equipped with a Teflon-coated magnetic stirring bar, $Pd(PPh_3)_2Cl_2$ (5 mol %), CuI (5mol %), Et_3N (3.3 equiv) and degassed (by bubbling dry N_2 for 10 minutes) MeCN (30 mL) were added. Then, the iodoarene (1.1 equiv) was added and the mixture was heated to 60 °C and stirred for 5 minutes. Benzyl propargyl amine **S8** (1.0 equiv) was added and the reaction mixture

was stirred for 7 hours at 60 °C. Then, the reaction mixture was cooled down to ambient temperature and concentrated in vacuo. The resulting crude mixture was dissolved in EtOAc (20 mL), then washed with water (20 mL) and brine (20 mL). The organic layer was dried over Na_2SO_4, filtered, and concentrated in vacuo. The crude product **S9** was purified by silica gel flash chromatography.

2) DMAP (0.5 mmol, 0.5 equiv) and **S3** (1 mmol, 1.0 equiv) were dissolved in dry toluene (3 mL), then **S9** (1.5 mmol) was added, and the mixture was stirring at 110 °C for 2 h. After the disappearance of substrate **S9** (monitored by TLC), the mixture was cooled to room temperature and then quenched with HCl (aq.). The residue was extracted three times with ethyl acetate, and was then washed with $NaHCO_3$ (aq.). Finally, the crude product was purified by silica gel flash chromatography to afford the desired products **4f-4w**.

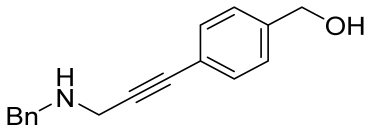

S9i

(4-(3-(benzylamino)prop-1-yn-1-yl)phenyl)methanol (S9i); **TLC**: R_f = 0.20 (petroleum ether /ethyl acetate = 2/1, v/v, UV); brown oil; yield: 27%; **^{1}H NMR (400 MHz, $CDCl_3$)**: δ 7.72-7.05 (m, 9H), 4.64 (s, 2H), 3.93 (brs, 2H), 3.62 (brs, 2H), 2.36 (s, 2H); **^{13}C{^{1}H}NMR (101 MHz, $CDCl_3$)**: δ 141.3, 139.3, 131.9, 128.9, 128.6, 127.4, 126.8, 122.2, 87.3, 83.9, 64.7, 52.5, 38.1; **HRMS (ESI, m/z)**: calcd. For $C_{17}H_{18}NO^+$ $[M+H]^+$: 252.1383, found: 252.1385.

1.3.4 Failed substrate

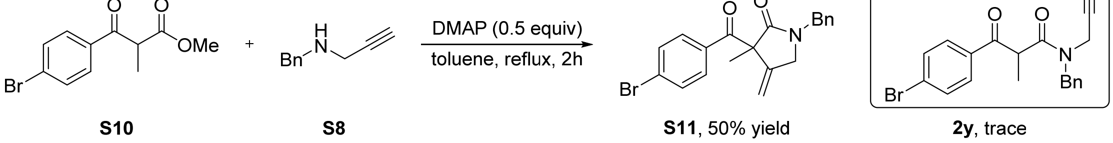

S10 **S8** **S11**, 50% yield **2y**, trace

DMAP (0.5 mmol, 0.5 equiv) and **S10** (1 mmol, 1.0 equiv) were dissolved in dry

toluene (3 mL), then **S8** (1.5 mmol) was added. The mixture was stirring at 110 °C for 12 h. After disappearance of **S10** (monitored by TLC), the mixture was cooled to room temperature and was quenched with HCl (aq.). The residue was extracted three times with ethyl acetate, and was then washed with $NaHCO_3$ (aq.). Finally, the crude product was purified by silica gel flash chromatography to get the product **S11**.

1-benzyl-3-(4-bromobenzoyl)-3-methyl-4-methylenepyrrolidin-2-one (S11); **TLC**: R_f = 0.40 (petroleum ether /ethyl acetate = 5/1, v/v, UV); yellow oil; yield: 50%; **^{1}H NMR (400 MHz, $CDCl_3$)**: δ 7.42 – 7.31 (m, 7H), 7.26-7.24 (m, 2H), 5.07 (s, 1H), 4.96 (s, 1H), 4.82 (d, *J* = 14.4 Hz, 1H), 4.25 (d, *J* = 14.4 Hz, 1H), 4.13 – 4.05 (m, 2H), 1.61 (s, 3H); **^{13}C{^{1}H}NMR (101 MHz, $CDCl_3$)**: δ 195.2, 173.6, 143.1, 135.3, 134.8, 131.7, 130.0, 129.0, 128.9, 128.3, 127.4, 111.2, 60.9, 50.0, 46.7, 23.1; **HRMS (ESI, m/z)**: calcd. For $C_{20}H_{19}BrNO_2^+$ $[M+H]^+$: 384.0594, found: 384.0601.

Compound 2a (Fig. 2)

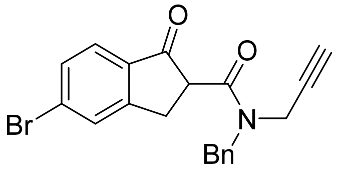

2a

***N*-benzyl-5-bromo-1-oxo-*N*-(prop-2-yn-1-yl)-2,3-dihydro-1*H*-indene-2-carboxamide (2a)**; **TLC**: R_f = 0.40 (petroleum ether /ethyl acetate = 5/1, v/v, UV); white solid (mp: 92-93°C); yield: 42%; Enol isomerization were observed by NMR; **^{1}H NMR (400 MHz, $CDCl_3$)**: δ 7.69 (d, *J* = 20.6 Hz, 1H), 7.58 (dd, *J* = 8.0, 6.0 Hz, 1H), 7.50 (td, *J* = 8.4, 1.6 Hz, 1H), 7.39 (dd, *J* = 8.0, 6.8 Hz, 1H), 7.36-7.30 (m, 2H), 7.29-7.22 (m, 2H), 5.25 (dd, *J* = 16.1, 12.2 Hz, 1H), 4.90-4.83 (m, 1H), 4.35 (dd, *J* = 17.5, 2.4 Hz, 0.4H), 4.31-4.24 (m, 1.2H), 4.17 (dd, *J* = 17.4, 2.5 Hz, 0.4H), 4.04 (dd, *J* = 7.9, 3.6 Hz, 0.4H), 3.97-3.83 (m, 1.2H), 3.76 (dd, *J* = 17.3, 3.7 Hz, 0.4H), 3.34-2.12 (m, 1H), 2.33-2.22 (m, 1H); **^{13}C{^{1}H}NMR (101 MHz, $CDCl_3$)**: δ 200.5 (200.1), 167.7 (168.3), 156.4 (156.2), 136.3 (136.2), 134.1 (134.2), 131.41 (131.40), 131.1 (131.0), 129.9 (130.0), 128.8 (129.1), 128.0, 126.9 (127.7), 125.73 (125.72), 78.7 (78.5), 73.0 (72.5), 51.1 (49.3), 51.0 (50.8), 37.0 (35.5), 30.5 (30.7); **HRMS (ESI, m/z)**: calcd. For

$C_{20}H_{17}BrNO_2^+$ $[M+H]^+$: 382.0437, found: 382.0438.

Compound 2b (Fig. 2)

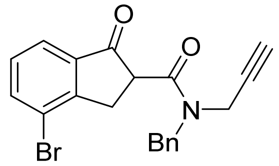

2b

***N*-benzyl-4-bromo-1-oxo-*N*-(prop-2-yn-1-yl)-2,3-dihydro-1*H*-indene-2-carboxamide (2b)**; **TLC**: R_f = 0.60 (petroleum ether /ethyl acetate = 5/1, v/v, UV); yellow oil; yield: 69%; Enol isomerization were observed by NMR; **^{1}H NMR (400 MHz, $CDCl_3$)**: δ 7.83-7.74 (m, 1H), 7.74-7.66 (m, 1H), 7.46-7.39 (m, 1H), 7.35 (d, *J* = 8.2 Hz, 2H), 7.32-7.24 (m, 3H), 5.25 (dd, *J* = 16.2, 8.4 Hz, 1H), 4.92-4.82 (m, 1H), 4.41-4.29 (m, 1.6H), 4.22 (dd, *J* = 17.3, 2.5 Hz, 0.4H), 4.08 (dd, *J* = 8.0, 3.7 Hz, 0.4H), 3.95 (dd, *J* = 18.9, 2.5 Hz, 0.6H), 3.84 (dd, *J* = 17.7, 3.6 Hz, 0.6H), 3.71 (dd, *J* = 17.6, 3.6 Hz, 0.4H), 3.35-3.12 (m, 1H), 2.35-2.25 (m, 1H); **^{13}C{^{1}H}NMR (101 MHz, $CDCl_3$)**: δ 201.1 (200.6), 167.7 (168.3), 154.5 (154.2), 138.3 (138.2), 137.3 (137.2), 136.4 (136.2), 129.5 (129.1), 128.8, 128.0, 126.9 (127.6), 123.4, 122.1 (122.0), 78.7 (78.5), 73.1 (72.5), 51.1 (49.3), 50.90 (50.87), 37.1 (35.5), 31.2 (32.2); **HRMS (ESI, m/z)**: calcd. For $C_{20}H_{17}BrNO_2^+$ $[M+H]^+$: 382.0437, found: 382.0439.

Compound 2c (Fig. 2)

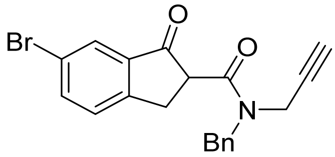

2c

***N*-benzyl-6-bromo-1-oxo-*N*-(prop-2-yn-1-yl)-2,3-dihydro-1*H*-indene-2-carboxamide (2c)**; **TLC**: R_f = 0.50 (petroleum ether /ethyl acetate = 5/1, v/v, UV); yellow oil; yield: 25%; Enol isomerization were observed by NMR; **^{1}H NMR (400 MHz, $CDCl_3$)**: δ 7.84 (dd, *J* = 7.0, 1.9 Hz, 1H), 7.69 (ddd, *J* = 11.8, 8.1, 1.9 Hz, 1H), 7.44-7.30 (m, 4H), 7.29-7.22 (m, 2H), 5.24 (dd, *J* = 16.1, 7.3 Hz, 1H), 4.91-4.79 (m, 1H), 4.39-4.24 (m, 1.6H), 4.17 (dd, *J* = 17.4, 2.5 Hz, 0.4H), 4.07 (dd, *J* = 7.9, 3.6 Hz, 0.4H), 3.91 (dd, *J* = 19.0, 2.5 Hz, 0.6H), 3.82 (dd, *J* = 17.4, 3.5 Hz, 0.6H), 3.70 (dd, *J*

= 17.2, 3.6 Hz, 0.4H), 3.31-3.09 (m, 1H), 2.33-2.23 (m, 1H); **^{13}C{^{1}H}NMR (101 MHz, CDCl$_3$)**: δ 200.4 (199.9), 167.7 (168.3), 153.5 (153.2), 138.3 (138.2), 137.1 (137.0), 136.3 (136.2), 128.8 (129.1), 128.2 (128.1), 127.97 (127.98), 127.7 (127.4), 126.9 (127.4), 121.8, 78.7 (78.5), 73.0 (72.5), 51.4 (49.3), 51.3 (50.8), 37.0 (35.5), 30.5 (30.7); **HRMS (ESI, m/z)**: calcd. For $C_{20}H_{17}BrNO_2^+$ [M+H]$^+$: 382.0437, found: 382.0438.

Compound 2d (Fig. 2)

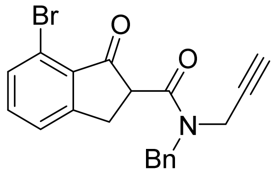

2d

***N*-benzyl-7-bromo-1-oxo-*N*-(prop-2-yn-1-yl)-2,3-dihydro-1*H*-indene-2-carboxamide (2d)**; **TLC**: R*f* = 0.50 (petroleum ether /ethyl acetate = 5/1, v/v, UV); white solid (mp: 83-84°C.); yield: 83%; **^{1}H NMR (400 MHz, Chloroform-*d*)**: δ 7.56-7.35 (m, 4H), 7.36-7.22 (m, 4H), 5.29 (dd, *J* = 30.9, 16.2 Hz, 1H), 4.94-4.81 (m, 1H), 4.39 (dd, *J* = 17.4, 2.5 Hz, 0.5H), 4.33 (dd, *J* = 8.0, 3.9 Hz, 0.5H), 4.28 (d, *J* = 15.2 Hz, 0.5H), 4.14 (dd, *J* = 17.3, 2.5 Hz, 0.5H), 4.07 (dd, *J* = 8.0, 3.8 Hz, 0.5H), 3.89 (td, *J* = 19.1, 3.2 Hz, 1H), 3.75 (dd, *J* = 17.2, 3.9 Hz, 0.5H), 3.31-3.08 (m, 1H), 2.31-2.22 (m, 1H); **^{13}C{^{1}H}NMR (101 MHz, CDCl$_3$)**: δ 199.1 (198.6), 167.8 (168.3), 157.6 (157.5), 136.25 (136.29), 135.9 (135.8), 132.8 (132.7), 132.48 (132.53), 128.8 (129.1), 127.93 (127.90), 126.8 (127.6), 125.7 (125.6), 120.32 (120.28), 78.7 (78.5), 73.0 (72.5), 51.8 (51.6), 49.3 (50.8), 37.0 (35.6), 29.8 (30.0); **HRMS (ESI, m/z)**: calcd. For $C_{20}H_{17}BrNO_2^+$ [M+H]$^+$: 382.0437, found: 382.0440.

Compound 2e (Fig. 2)

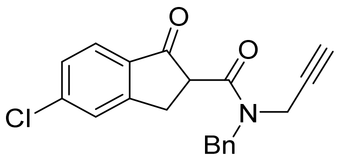

2e

***N*-benzyl-5-chloro-1-oxo-*N*-(prop-2-yn-1-yl)-2,3-dihydro-1*H*-indene-2-carboxamid (2e)**; **TLC**: R*f* = 0.30 (petroleum ether /ethyl acetate = 5/1, v/v, UV); white

solid (mp: 774-75°C.); yield: 44%; Enol isomerization were observed by NMR; **^{1}H NMR (400 MHz, $CDCl_3$)** δ 7.65 (dd, *J* = 8., 5.6 Hz, 1H), 7.50 (dd, *J* = 20.4, 1.6 Hz, 1H), 7.43-7.36 (m, 1H), 7.36-7.31 (m, 3H), 7.30-7.24 (m, 2H), 5.25 (dd, *J* = 14.8, 12.0 Hz, 1H), 4.92-4.81 (m, 1H), 4.40-4.23 (m, 1.5H), 4.17 (dd, *J* = 17.2, 2.4 Hz, 0.5H), 4.06 (dd, *J* = 8.0, 3.6 Hz, 0.5H), 3.90 (td, *J* = 18.8, 2.8 Hz, 1H), 3.75 (dd, *J* = 17.2, 3.6 Hz, 0.5H), 3.34-3.12 (m, 1H), 2.33-2.23 (m, 1H); **$^{13}C\{^1H\}$NMR (101 MHz, $CDCl_3$)**: δ 200.3 (199.8), 167.8 (168.3), 156.3 (156.1), 142.2 (142.1), 136.4 (136.3), 133.7 (133.8), 129.1 (128.6), 128.0 (128.8), 127.7, 126.9, 126.9 (126.8), 125.7 (125.6), 78.7 (78.5), 73.0 (72.5), 51.2 (49.3), 51.0 (50.8), 37.0 (35.5), 30.5 (30.7); **HRMS (ESI, m/z)**: calcd. For $C_{20}H_{17}ClNO_2^+$ $[M+H]^+$: 338.0942, found: 338.0940.

Compound 2f (Fig. 2)

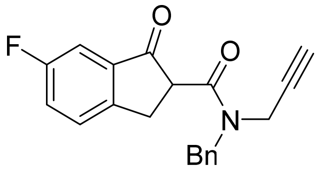

2f

***N*-benzyl-6-fluoro-1-oxo-*N*-(prop-2-yn-1-yl)-2,3-dihydro-1*H*-indene-2-carboxamide (2f)**; **TLC**: R_f = 0.30 (petroleum ether /ethyl acetate = 5/1, v/v, UV); yellow oil; yield: 76%; Enol isomerization were observed by NMR; **^{1}H NMR (400 MHz, $CDCl_3$)**: δ 7.53-7.41 (m, 1H), 7.41-7.30 (m, 5H), 7.30-7.23 (m, 2H), 5.25 (dd, *J* = 16.1, 7.5 Hz, 1H), 4.92-4.80 (m, 1H), 4.39-4.24 (m, 1.6H), 4.19 (dd, *J* = 17.4, 2.5 Hz, 0.4H), 4.09 (dd, *J* = 7.8, 3.6 Hz, 0.4H), 3.92 (dd, *J* = 18.9, 2.5 Hz, 0.6H), 3.85 (dd, *J* = 17.5, 3.5 Hz, 0.6H), 3.73 (dd, *J* = 17.0, 4.1 Hz, 0.4H), 3.34-3.12 (m, 1H), 2.33-2.23 (m, 1H); **$^{13}C\{^1H\}$NMR (101 MHz, $CDCl_3$)**: δ 200.9 (d, *J* = 3.2 Hz) [200.4 (d, *J* = 3.1 Hz)], 167.8 (168.4), 162.5 (d, *J* = 248.3 Hz), 150.4 (d, *J* = 2.2 Hz) [150.1 (d, *J* = 2.2 Hz)], 136.9 (d, *J* = 7.5 Hz) [137.1 (d, *J* = 7.4 Hz)], 136.4 (136.3), 128.9 (129.1), 128.1 (127.9), 128.02 (128.00), 126.9 (127.7), 123.4 (d, *J* = 23.9 Hz) [123.2 (d, *J* = 23.7 Hz)], 110.3 (d, *J* = 22.1 Hz), 78.8 (78.6), 73.0 (72.5), 52.0 (49.3), 51.8 (50.9), 37.1 (35.5), 30.3 (30.5); **^{19}FNMR** (376 MHz, Chloroform-*d*): δ -114.2; **HRMS (ESI, m/z)**: calcd. For $C_{20}H_{17}FNO_2^+$ $[M+H]^+$: 322.1238, found: 322.1240.

Compound 2g (Fig. 2)

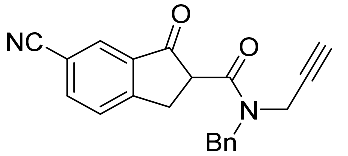

2g

***N*-benzyl-6-cyano-1-oxo-*N*-(prop-2-yn-1-yl)-2,3-dihydro-1*H*-indene-2-carboxamide (2g)**; **TLC**: R_f = 0.30 (petroleum ether /ethyl acetate = 5/1, v/v, UV); yellow oi; yield: 65%; Enol isomerization were observed by NMR; **^{1}H NMR (400 MHz, $CDCl_3$)**: δ 8.01 (d, *J* = 7.5 Hz, 1H), 7.85 (ddd, *J* = 11.6, 8.0, 1.6 Hz, 1H), 7.65 (dd, *J* = 20.4, 8.0 Hz, 1H), 7.45-7.19 (m, 5H), 5.24 (t, *J* = 15.6 Hz, 1H), 4.85 (dd, *J* = 21.9, 17.6 Hz, 1H), 4.38-4.25 (m, 1.6H), 4.17 (dd, *J* = 17.3, 2.5 Hz, 0.5H), 4.11 (dd, *J* = 8.0, 3.6 Hz, 0.4H), 3.95 (td, *J* = 18.6, 3.0 Hz, 1H), 3.86 (dd, *J* = 17.9, 3.6 Hz, 0.5H), 3.45-3.22 (m, 1H), 2.34-2.24 (m, 1H); **^{13}C{^{1}H}NMR (101 MHz, $CDCl_3$)**: δ 199.9 (199.4), 167.2 (167.8), 158.9 (158.7), 138.0 (137.9), 136.2 (136.0), 136.0 (135.9), 128.9 (129.1), 128.8 (128.8), 128.0 (126.9), 127.9 (128.1), 127.77 (127.83), 117.9, 112.13 (112.11), 78.5 (78.3), 73.2 (72.6), 51.2 (49.4), 51.0 (50.9), 37.1 (35.6), 31.3 (31.5); **HRMS (ESI, m/z)**: calcd. For $C_{21}H_{17}N_2O_2^+$ [M+H]$^+$: 329.1285, found: 329.1286.

Compound 2h (Fig. 2)

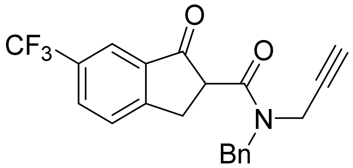

2h

***N*-benzyl-1-oxo-*N*-(prop-2-yn-1-yl)-6-(trifluoromethyl)-2,3-dihydro-1*H*-indene-2-carboxamide (2h)**; **TLC**: R_f = 0.30 (petroleum ether /ethyl acetate = 5/1, v/v, UV); yellow oil; yield: 89%; Enol isomerization were observed by NMR; **^{1}H NMR (400 MHz, $CDCl_3$)**: δ 7.99 (dd, *J* = 7.4, 1.7 Hz, 1H), 7.85 (ddd, *J* = 11.8, 8.1, 1.8 Hz, 1H), 7.65 (dd, *J* = 20.6, 8.0 Hz, 1H), 7.44-7.23 (m, 5H), 5.35-5.16 (m, 1H), 4.94-4.81 (m, 1H), 4.44-4.23 (m, 1.6H), 4.23-4.06 (m, 1H), 3.95 (ddd, *J* = 18.9, 9.7, 3.0 Hz, 1H), 3.84 (dd, *J* = 17.6, 3.6 Hz, 0.4H), 3.44-3.21 (m, 1H), 2.34-2.23 (m, 1H); **^{13}C{^{1}H}NMR (101 MHz, $CDCl_3$)**: δ 200.6 (200.1), 167.5 (168.1), 158.1 (157.8), 136.3 (136.2), 135.6

(135.7), 131.9 (q, J = 3.5 Hz) [131.8 (q, J = 3.4 Hz)], 130.6 (q, J = 33.1 Hz), 128.8 (129.1), 128.0 (126.9), 127.7 (128.0), 127.4 (127.3), 123.8 (q, J = 272.3 Hz), 121.8 (q, J = 3.6 Hz) 78.6 (78.4), 73.1 (72.5), 51.4 (49.3), 51.2 (50.9), 37.1 (35.5), 30.9 (31.1); **^{19}FNMR (376 MHz, Chloroform-*d*)**: δ -62.5 (d, J = 3.8 Hz); **HRMS (ESI, m/z)**: calcd. For $C_{21}H_{17}F_3NO_2^+$ $[M+H]^+$: 372.1206, found: 372.1208.

Compound 2i (Fig. 2)

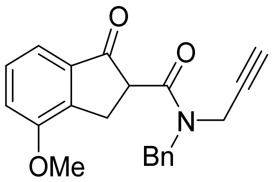

2i

***N*-benzyl-4-methoxy-1-oxo-*N*-(prop-2-yn-1-yl)-2,3-dihydro-1*H*-indene-2-carboxamide (2i)**; **TLC**: R_f = 0.50 (petroleum ether /ethyl acetate = 5/1, v/v, UV); white solid (mp: 92-93°C.); yield: 82%; Enol isomerization were observed by NMR; **^{1}H NMR (400 MHz, $CDCl_3$)**: δ 7.43-7.21 (m, 7H), 7.03 (ddd, J = 11.6, 6.1, 2.7 Hz, 1H), 5.23 (d, J = 16.0 Hz, 1H), 4.94-4.79 (m, 1H), 4.38-4.26 (m, 1H), 4.28-4.13 (m, 1H), 4.00 (dd, J = 7.8, 3.5 Hz, 0.4H), 3.97-3.84 (m, 3.6H), 3.74 (dd, J = 17.6, 3.5 Hz, 0.6H), 3.62 (dd, J = 17.5, 3.5 Hz, 0.4H), 3.28-3.08 (m, 1H), 2.34-2.22 (m, 1H); **^{13}C{^{1}H}NMR (101 MHz, $CDCl_3$)**: δ 201.9 (201.5), 168.2 (168.8), 157.0 (156.9), 143.8 (143.5), 136.8 (136.6), 136.5 (136.3), 129.2 (129.0), 128.7, 127.9 (126.9), 127.5 (127.8), 115.7, 115.9 (115.5), 78.8 (78.6), 72.9 (72.3), 55.6 (55.5), 50.9 (49.0), 50.7 (50.7), 37.0 (35.2), 27.6 (27.9); **HRMS (ESI, m/z)**: calcd. For $C_{21}H_{20}NO_3^+$ $[M+H]^+$: 334.1438, found: 334.1439.

Compound 2j (Fig. 2)

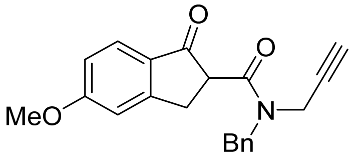

2j

***N*-benzyl-5-methoxy-1-oxo-*N*-(prop-2-yn-1-yl)-2,3-dihydro-1*H*-indene-2-carboxamide (2j)**; **TLC**: R_f = 0.20 (petroleum ether /ethyl acetate = 5/1, v/v, UV);

white solid (mp: 108-109°C.); yield: 83%; Enol isomerization were observed by NMR; **^{1}H NMR (400 MHz, $CDCl_3$)**: δ 7.65 (dd, *J* = 8.4, 5.6 Hz, 1H), 7.43-7.22 (m, 5H), 6.97-6.84 (m, 2H), 5.29 (dd, *J* = 24.0, 17.2 Hz, 1H), 4.91 (dd, *J* = 28.8, 18.0 Hz, 1H), 4.35 (dd, *J* = 17.6, 2.0 Hz, 0.4H), 4.32-4.23 (m, 1H), 4.18 (dd, *J* = 17.2, 2.4 Hz, 0.4H), 4.04 (dd, *J* = 8.0, 3.6 Hz, 0.4H), 3.93 (d, *J* = 2.4 Hz, 0.3H), 3.88 (d, *J* = 8.0 Hz, 3.5H), 3.82 (d, *J* = 3.6 Hz, 0.3H), 3.73 (dd, *J* = 17.2, 3.6 Hz, 0.4H), 3.30-3.08 (m, 1H), 2.31-2.21 (m, 1H); **$^{13}C\{^1H\}$NMR (101 MHz, $CDCl_3$)**: δ 199.7 (199.3), 168.5 (169.0), 166.1 (166.0), 158.1 (157.8), 136.54 (136.51), 128.8 (129.0), 128.6 (128.5), 128.0 (127.0), 127.6 (127.9), 126.30 (126.29), 116.1 (116.0), 109.6 (109.5), 79.0 (78.7), 72.8 (72.4), 55.9 (55.8), 51.3 (49.2), 51.1 (50.9), 37.0 (35.4), 30.8 (31.0); **HRMS (ESI, m/z)**: calcd. For $C_{21}H_{20}NO_3^+$ $[M+H]^+$: 334.1438, found: 334.1440.

Compound 2k (Fig. 2)

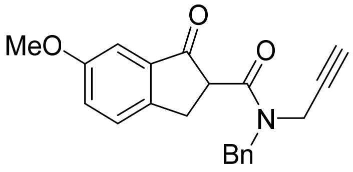

2k

***N*-benzyl-6-methoxy-1-oxo-*N*-(prop-2-yn-1-yl)-2,3-dihydro-1*H*-indene-2-carboxamide (2k)**; **TLC**: R_f = 0.40 (petroleum ether /ethyl acetate = 5/1, v/v, UV); white solid (mp: 115-116°C.); yield: 55%; Enol isomerization were observed by NMR; **^{1}H NMR (400 MHz, $CDCl_3$)**: δ 7.43-7.38 (m, 1H), 7.38-7.30 (m, 3H), 7.30-7.24 (m, 2H), 7.24-7.13 (m, 2H), 5.24 (d, *J* = 14.8 Hz, 1H), 4.93-4.82 (m, 1H), 4.38-4.26 (m, 1.6H), 4.20 (dd, *J* = 17.3, 2.5 Hz, 0.4H), 4.06 (dd, *J* = 7.7, 3.5 Hz, 0.4H), 3.92 (dd, *J* = 18.9, 2.5 Hz, 0.6H), 3.84-3.73 (m, 3.6H), 3.65 (dd, *J* = 16.6, 3.6 Hz, 0.4H), 3.29-3.08 (m, 1H), 2.32-2.22 (m, 1H); **$^{13}C\{^1H\}$NMR (101 MHz, $CDCl_3$)**: δ 201.7 (201.3), 168.2 (168.9), 159.7, 147.9 (147.6), 136.5 (136.4), 128.8 (129.0), 128.0, 127.6 (127.9), 127.3 (127.2), 127.0, 125.0 (124.9), 105.6, 78.9 (78.7), 72.9 (72.4), 55.7, 51.8 (51.7), 49.1 (50.8), 37.0 (35.3), 30.1 (30.3); **HRMS (ESI, m/z)**: calcd. For $C_{21}H_{20}NO_3^+$ $[M+H]^+$: 334.1438, found: 334.1439.

Compound 2l (Fig. 2)

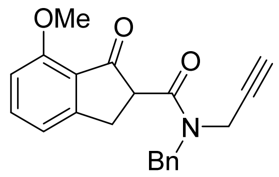

2l

***N*-benzyl-7-methoxy-1-oxo-*N*-(prop-2-yn-1-yl)-2,3-dihydro-1*H*-indene-2-carboxamide (2l)**; **TLC**: R$_f$ = 0.30 (petroleum ether /ethyl acetate = 5/1, v/v, UV); white solid (mp: 139-140°C.); yield: 93%; Enol isomerization were observed by NMR; **^{1}H NMR (400 MHz, CDCl$_3$)**: δ 7.53 (dt, *J* = 12.1, 7.9 Hz, 1H), 7.41-7.21 (m, 5H), 7.03 (dd, *J* = 19.0, 7.6 Hz, 1H), 6.77 (t, *J* = 8.9 Hz, 1H), 5.33 (dd, *J* = 16.2, 8.9 Hz, 1H), 5.01-4.78 (m, 1H), 4.35 (dd, *J* = 17.3, 2.5 Hz, 0.4H), 4.27 (dd, *J* = 7.9, 4.0 Hz, 0.6H), 4.22 (d, *J* = 15.2 Hz, 0.6H), 4.14 (dd, *J* = 17.3, 2.5 Hz, 0.4H), 4.03 (dd, *J* = 7.9, 3.8 Hz, 0.4H), 3.93 (d, *J* = 7.5 Hz, 3H), 3.86 (ddd, *J* = 17.2, 9.0, 3.2 Hz, 1.2H), 3.74 (dd, *J* = 17.1, 3.8 Hz, 0.4H), 3.29-3.08 (m, 1H), 2.29-2.19 (m, 1H); **^{13}C{^{1}H}NMR (101 MHz, CDCl$_3$)**: δ 199.2 (198.8), 168.3 (168.7), 158.64 (158.67), 157.2 (157.1), 137.3 (137.2), 136.4 (136.5), 128.7 (128.9), 127.8 (127.0), 127.4 (127.8), 123.3 (123.4), 118.3 (118.2), 109.1, 78.9 (78.7), 72.7 (72.3), 55.81 (55.78), 51.4 (49.1), 51.2 (50.7), 36.8 (35.3), 30.2 (30.5); **HRMS (ESI, m/z)**: calcd. For $C_{21}H_{20}NO_3^+$ $[M+H]^+$: 334.1438, found: 334.1437.

Compound 2m (Fig. 2)

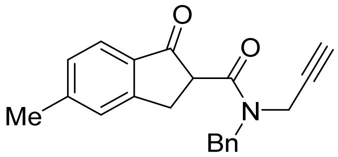

2m

***N*-benzyl-5-methyl-1-oxo-*N*-(prop-2-yn-1-yl)-2,3-dihydro-1*H*-indene-2-carboxamide (2m)**; **TLC**: R$_f$ = 0.30 (petroleum ether /ethyl acetate = 5/1, v/v, UV); white solid (mp: 88-89°C.); yield: 89%; Enol isomerization were observed by NMR; **^{1}H NMR (400 MHz, CDCl$_3$)**: δ 7.62 (dd, *J* = 7.9, 5.3 Hz, 1H), 7.43-7.29 (m, 4H), 7.27 (d, *J* = 10.7 Hz, 2H), 7.17 (t, *J* = 8.1 Hz, 1H), 5.28 (t, *J* = 17.0 Hz, 1H), 4.96-4.84 (m, 1H), 4.40-4.24 (m, 1.6H), 4.19 (dd, *J* = 17.3, 2.5 Hz, 0.4H), 4.04 (dd, *J* = 7.8, 3.7 Hz, 0.4H), 3.92 (dd, *J* = 19.0, 2.5 Hz, 0.6H), 3.84 (dd, *J* = 17.1, 3.6 Hz, 0.6H), 3.72 (dd, *J*

= 17.0, 3.7 Hz, 0.4H), 3.31-3.10 (m, 1H), 2.31-2.21 (d, *J* = 8.5 Hz, 3H), 2.26 (m, 1H); **^{13}C{^{1}H}NMR (101 MHz, CDCl$_3$)**: δ 201.1 (200.6), 168.3 (168.8), 155.3 (155.1), 146.8 (146.7), 136.5 (136.4), 133.0 (132.9), 128.93 (128.91), 128.7, 127.9 (127.8), 127.5, 126.9 (126.8), 124.3, 78.9 (78.6), 72.7 (72.2), 51.2 (49.1), 51.0 (50.7), 36.9 (35.3), 30.5 (30.7), 22.2 (22.1); **HRMS (ESI, m/z)**: calcd. For $C_{21}H_{20}NO_2^+$ [M+H]$^+$: 318.1489, found: 318.1491.

Compound 2n (Fig. 2)

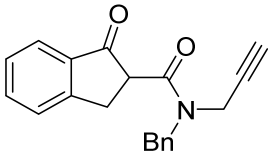

2n

***N*-benzyl-1-oxo-*N*-(prop-2-yn-1-yl)-2,3-dihydro-1*H*-indene-2-carboxamide (2n)**; **TLC**: R$_f$ = 0.50 (petroleum ether /ethyl acetate = 5/1, v/v, UV); white solid (mp: 65-66°C.); yield: 79%; Enol isomerization were observed by NMR; **^{1}H NMR (400 MHz, CDCl$_3$)**: δ 7.78-7.70 (m, 1H), 7.66-7.57 (m, 1H), 7.51 (dd, *J* = 20.2, 7.7 Hz, 1H), 7.44-7.31 (m, 4H), 7.31-7.23 (m, 2H), 5.27 (dd, *J* = 16.2, 11.3 Hz, 1H), 4.94-4.85 (m, 1H), 4.41-4.24 (m, 1.6H), 4.19 (dd, *J* = 17.3, 2.5 Hz, 0.4H), 4.05 (dd, *J* = 7.9, 3.7 Hz, 0.4H), 3.91 (ddd, *J* = 17.1, 11.6, 3.1 Hz, 1.2H), 3.78 (dd, *J* = 17.1, 3.7 Hz, 0.4H), 3.38-3.16 (m, 1H), 2.32-2.22 (m, 1H); **^{13}C{^{1}H}NMR (101 MHz, CDCl$_3$)**: δ 201.8 (201.4), 168.2 (168.8), 154.9 (154.6), 136.5 (136.4), 135.6 (135.5), 135.2 (135.3), 128.8 (129.1), 128.0 (127.7), 127.6 (127.9), 127.0, 126.7 (126.6), 124.6, 78.9 (78.7), 72.9 (72.4), 51.1 (51.0), 49.2 (50.9), 37.0 (35.4), 30.8 (31.0); **HRMS (ESI, m/z)**: calcd. For $C_{20}H_{18}NO_2^+$ [M+H]$^+$: 304.1332, found: 304.1329.

Compound 2o (Fig. 2)

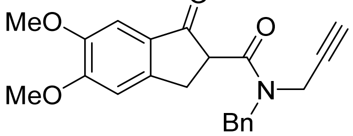

2o

***N*-benzyl-5,6-dimethoxy-1-oxo-*N*-(prop-2-yn-1-yl)-2,3-dihydro-1*H*-indene-2-carboxamide (2o)**; **TLC**: R$_f$ = 0.50 (petroleum ether /ethyl acetate = 5/1, v/v, UV);

white solid (mp: 159-160°C.); yield: 55%; Enol isomerization were observed by NMR; **^{1}H NMR (400 MHz, $CDCl_3$)**: δ 7.42-7.29 (m, 3H), 7.29-7.21 (m, 2H), 7.12 (d, J = 5.7 Hz, 1H), 6.91 (d, J = 20.1 Hz, 1H), 5.27 (dd, J = 18.6, 15.4 Hz, 1H), 5.02-4.81 (m, 1H), 4.36-4.23 (m, 1.6H), 4.19 (dd, J = 17.4, 2.5 Hz, 0.4H), 4.04 (dd, J = 7.5, 3.2 Hz, 0.4H), 3.96 (d, J = 8.6 Hz, 3.6H), 3.88 (d, J = 5.7 Hz, 3H), 3.76 (dd, J = 17.1, 3.1 Hz, 0.6H), 3.64 (dd, J = 16.9, 3.2 Hz, 0.4H), 3.26-3.04 (m, 1H), 2.31-2.21 (m, 1H),; **^{13}C{^{1}H}NMR (101 MHz, $CDCl_3$)**: δ 200.1 (199.7), 168.5 (169.0), 156.2 (156.1), 150.6 (150.3), 149.73 (149.70), 136.52 (136.45), 128.8 (129.0), 127.9 (127.0), 127.9 (128.0), 127.5 (127.8), 107.4 (107.4), 104.8, 79.0 (78.7), 72.8 (72.3), 56.42 (56.39), 56.2, 51.3 (49.1), 51.1 (50.8), 37.0 (35.3), 30.5 (30.7); **HRMS (ESI, m/z)**: calcd. For $C_{22}H_{22}NO_4^+$ $[M+H]^+$: 364.1543, found: 364.1540.

Compound 2p (Fig. 2)

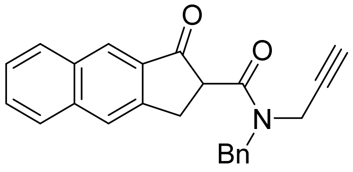

2p

***N*-benzyl-1-oxo-*N*-(prop-2-yn-1-yl)-2,3-dihydro-1*H*-cyclopenta[*b*]naphthalene-2-carboxamide (2p)**; **TLC**: R_f = 0.60 (petroleum ether /ethyl acetate = 5/1, v/v, UV); white solid (mp: 151-152°C.); yield: 28%; Enol isomerization were observed by NMR; **^{1}H NMR (400 MHz, $CDCl_3$)**: δ 8.31 (d, J = 6.4 Hz, 1H), δ 8.02-7.81 (m, 3H), 7.63-7.56 (m, 1H), 7.54-7.44 (m, 1H), 7.45-7.26 (m, 5H), 5.30 (dd, J = 16.4, 13.6 Hz, 1H), 4.92 (d, J = 16.8 Hz, 1H), 4.44-4.34 (m, 1H), 4.32 (d, J = 15.2 Hz, 0.5H), 4.21 (dd, J = 17.6, 2.4 Hz, 0.5H), 4.15 (dd, J = 8.5, 4.5 Hz, 0.5H), 4.07 (ddd, J = 16.8, 4.4, 1.2 Hz, 0.5H), 3.96 (dd, J = 19.2, 2.4 Hz, 1H), 3.56-3.34 (m, 1H), 2.34-2.23 (m, 1H); **^{13}C{^{1}H}NMR (101 MHz, $CDCl_3$)**: δ 202.1 (201.7), 168.3 (168.9), 146.9 (146.7), 137.7 (137.6), 136.5 (136.4), 133.1 (133.2), 132.5, 130.5 (129.2), 129.1, 128.8, 128.0, 127.98 (127.96), 127.0 (127.6), 126.4 (126.3), 125.70 (125.66), 124.82 (124.75), 78.9 (78.7), 72.9 (72.4), 51.9 (51.8), 49.2 (50.9), 37.1 (35.4), 30.3 (30.6); **HRMS (ESI, m/z)**: calcd. For $C_{24}H_{20}NO_2^+$ $[M+H]^+$: 354.1489, found: 354.1492.

Compound 2q (Fig. 2)

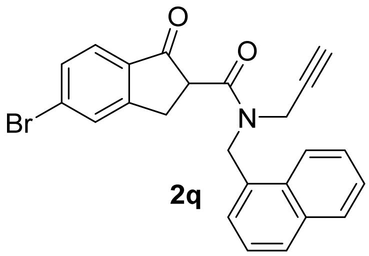

5-bromo-*N*-(naphthalen-1-ylmethyl)-1-oxo-*N*-(prop-2-yn-1-yl)-2,3-dihydro-1*H*-indene-2-carboxamide (2q); **TLC**: R_f = 0.40 (petroleum ether /ethyl acetate = 5/1, v/v, UV); white solid (mp: 105-106°C.); yield: 86%; Enol isomerization were observed by NMR; **^{1}H NMR (400 MHz, $CDCl_3$)**: δ 8.01 (dd, *J* = 27.4, 9.2 Hz, 1H), 7.93-7.73 (m, 22H), 7.65-7.32 (m, 7H), 5.90 (d, *J* = 18.3 Hz, 0.4H), 5.66 (d, *J* = 15.3 Hz, 0.6H), 5.28 (d, *J* = 18.3 Hz, 0.4H), 4.86-4.77 (m, 1.2H), 4.62 (dd, *J* = 17.4, 2.6 Hz, 0.4H), 4.31 (dd, *J* = 7.9, 3.6 Hz, 0.6H), 4.19 (dd, *J* = 17.4, 2.5 Hz, 0.4H), 3.95-3.87 (m, 1.6H), 3.78 (dd, *J* = 17.4, 3.7 Hz, 0.4H), 3.36-3.05 (m, 1H), 2.31-2.23 (m, 1H); **$^{13}C\{^1H\}$NMR (101 MHz, $CDCl_3$)**: δ 200.3 (200.1), 167.7 (169.0), 156.4 (156.2), 134.2 (134.1), 134.0 (133.9) 131.6 (131.8), 131.4 (131.5), 131.4 (131.1), 131. (130.7), 130.0 (129.9), 128.8 (129.1), 128.7 (128.4), 126.6 (126.8), 126.44 (126.36), 126.1 (123.5), 125.8 (125.7), 125.5 (125.6), 122.9 (122.5), 78.7 (78.6), 73.1 (72.7), 51.3 (51.1), 47.1 (48.6), 36.7 (36.4), 30.5 (30.7); **HRMS (ESI, m/z)**: calcd. For $C_{24}H_{19}BrNO_2^+$ $[M+H]^+$: 432.0594, found: 432.0595.

Compound 2r (Fig. 2)

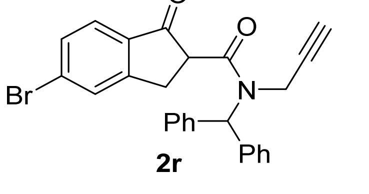

***N*-benzhydryl-5-bromo-1-oxo-*N*-(prop-2-yn-1-yl)-2,3-dihydro-1*H*-indene-2-carboxamide (2r)**; **TLC**: R_f = 0.50 (petroleum ether /ethyl acetate = 5/1, v/v, UV); white solid (mp: 133-134°C.); yield: 27%; Enol isomerization were observed by NMR; **^{1}H NMR (400 MHz, Chloroform-*d*)**: δ 7.67 (d, *J* = 29.3 Hz, 1H), 7.61 (d, *J* = 8.2 Hz, 1H), 7.55-7.33 (m, 9H), 7.27 (t, *J* = 8.4 Hz, 2H), 7.08 (d, *J* = 4.3 Hz, 1H), 5.02 (dd, *J* =

19.1, 2.5 Hz, 0.7H), 4.52 (dd, J = 7.8, 3.7 Hz, 0.7H), 4.21 (dd, J = 17.2, 2.5 Hz, 0.3H), 4.05-3.71 (m, 2.3H), 3.36-2.93 (m, 1H), 2.05-1.99 (m, 1H); **$^{13}C\{^1H\}$NMR (101 MHz, $CDCl_3$)**: δ 201.1 (200.5), 168.5 (168.7), 156.6 (156.5), 138.8 (139.3), 138.6 (138.7), 134.2, 131.4 (131.1), 130.3 (130.8), 130.0 (129.9), 128.8(128.9), 128.6 (128.8), 128.5, 128.1 (127.9), 128.0, 127.5 (127.7), 125.74 (125.71), 79.8, 72.0 (71.0), 62.2 (65.0), 52.1 (51.8), 35.1 (34.5), 30.5 (30.8); **HRMS (ESI, m/z)**: calcd. For $C_{26}H_{21}BrNO_2^+$ $[M+H]^+$: 458.0750, found: 458.0748.

Compound 2s (Fig. 2)

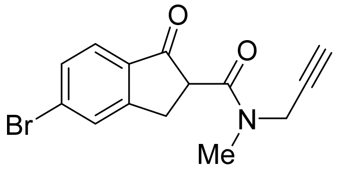

2s

5-bromo-*N*-methyl-1-oxo-*N*-(prop-2-yn-1-yl)-2,3-dihydro-1*H*-indene-2-carboxamide (2s); **TLC**: R_f = 0.30 (petroleum ether /ethyl acetate = 5/1, v/v, UV); white solid (mp: 109-110°C.); yield: 82%; Enol isomerization were observed by NMR; **^1H NMR (400 MHz, $CDCl_3$)**: δ 7.69 (d, J = 4.6 Hz, 1H), 7.58-7.46 (m, 2H), 4.96 (dd, J = 18.8, 2.5 Hz, 0.4H), 4.50 (dd, J = 17.3, 2.5 Hz, 0.6H), 4.14 (ddd, J = 24.9, 7.9, 3.6 Hz, 1H), 4.08-3.96 (m, 1H), 3.86-3.71 (m, 1H), 3.38 (s, 1.8H), 3.24 (dt, J = 17.4, 8.9 Hz, 1H), 3.07 (s, 1.2H), 2.35-2.24 (m, 1H); **$^{13}C\{^1H\}$NMR (101 MHz, $CDCl_3$)**: δ 200.2 (200.5), 167.6 (167.2), 156.2 (156.4), 134.2 (134.1), 131.38 (131.39), 131.0 (131.1), 129.95 (129.96), 125.65 (125.68), 78.5 (78.4), 72.3 (73.2), 50.8 (51.0), 37.3 (40.1), 35.5 (34.5), 30.3 (30.40; **HRMS (ESI, m/z)**: calcd. For $C_{14}H_{13}BrNO_2^+$ $[M+H]^+$: 306.0124, found: 306.0120.

Compound 2t (Fig. 2)

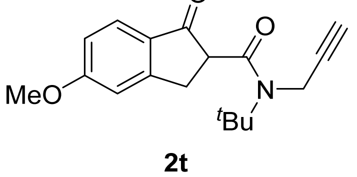

2t

***N*-(*tert*-butyl)-5-methoxy-1-oxo-*N*-(prop-2-yn-1-yl)-2,3-dihydro-1*H*-indene-2-**

carboxamide (2t); **TLC**: R$_f$ = 0.30 (petroleum ether /ethyl acetate = 5/1, v/v, UV); white solid (mp: 123-124°C.); yield: 22%; **^{1}H NMR (400 MHz, CDCl$_3$)**: δ 7.63 (d, *J* = 8.4 Hz, 1H), 6.94-6.84 (m, 2H), 5.01 (dd, *J* = 20.0, 2.4 Hz, 1H), 4.25 (dd, *J* = 7.6, 3.2 Hz, 1H), 4.13 (dd, *J* = 20.0, 2.4 Hz, 1H), 3.88 (s, 3H), 3.72 (dd, *J* = 16.8, 3.2 Hz, 1H), 3.20 (dd, *J* = 17.2, 7.6 Hz, 1H), 2.30 (t, *J* = 2.4 Hz, 1H), 1.50 (s, 9H); **^{13}C{^{1}H}NMR (101 MHz, CDCl$_3$)**: δ 200.6, 168.9, 165.9, 158.3, 128.7, 126.2, 115.9, 109.4, 81.5, 72.2, 58.7, 55.8, 53.3, 34.9, 31.5, 28.8; **HRMS (ESI, m/z)**: calcd. For $C_{18}H_{22}NO_3^+$ [M+H]$^+$: 300.1594, found: 300.1594.

Compound 2u (Fig. 2)

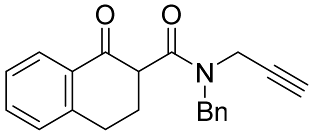

2u

***N*-benzyl-1-oxo-*N*-(prop-2-yn-1-yl)-1,2,3,4-tetrahydronaphthalene-2-carboxamide (2u)**; **TLC**: R$_f$ = 0.30 (petroleum ether /ethyl acetate = 5/1, v/v, UV); white solid (mp: 75-76°C.); yield: 40%; **^{1}H NMR (400 MHz, CDCl$_3$)**: δ 8.04 (t, *J* = 6.7 Hz, 1H), 7.53 – 7.22 (m, 8H), 5.32 (d, *J* = 15.1 Hz, 0.6H), 4.79 (q, *J* = 20.6 Hz 0.8H), 4.37 – 4.21 (m, 2H), 4.00 (dd, *J* = 12.2, 4.6 Hz, 0.6H), 3.85 – 3.78 (m, 1H), 3.14 – 3.06 (m, 1.6H), 2.91 (ddd, *J* = 16.5, 11.7, 4.4 Hz, 0.4H), 2.63 (dqd, *J* = 23.7, 12.1, 5.0 Hz, 1H), 2.38 – 2.24 (m, 2H); **^{13}C{^{1}H}NMR (101 MHz, CDCl$_3$)**: δ 194.6 (194.3), 170.2 (170.6), 144.3 (144.1), 136.5 (136.3), 134.1 (134.0), 132.0, 129.1 (128.9), 128.8 (129.0), 128.1 (127.8), 127.5 (127.9), 126.94 (126.92), 126.8, 78.9 (78.8), 72.9 (72.2), 52.2 (52.0), 48.8 (50.7), 36.7 (34.8), 28.5 (28.3), 26.6(26.7); **HRMS (ESI, m/z)**: calcd. For $C_{21}H_{20}NO_2^+$ [M+H]$^+$: 318.1489, found: 318.1498.

Compound 2v (Fig. 2)

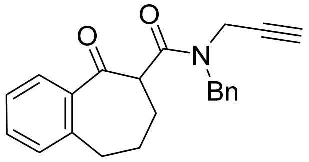

2v

***N*-benzyl-5-oxo-*N*-(prop-2-yn-1-yl)-6,7,8,9-tetrahydro-5*H*-benzo[7]annulene-6-carboxamide (2v)**; **TLC**: R$_f$ = 0.30 (petroleum ether /ethyl acetate = 5/1, v/v, UV); white solid (mp: 96-97°C.); yield: 30%; **^{1}H NMR (400 MHz, CDCl$_3$)**: δ 7.72 (dd, *J* = 7.8, 1.5 Hz, 0.5H), 7.62 (dd, *J* = 7.7, 1.5 Hz, 0.5H), 7.50 – 7.14 (m, 8H), 5.15 (d, *J* = 15.1 Hz, 0.5H), 4.62 (s, 1H), 4.40 (d, *J* = 15.1 Hz, 0.5H), 4.34 (dd, *J* = 17.3, 2.5 Hz, 0.5H), 4.21 (dd, *J* = 17.4, 2.5 Hz, 0.5H), 4.13 – 4.09 (m, 1H), 3.92 (dd, *J* = 11.3, 3.9 Hz, 0.5H), 3.75 (dd, *J* = 18.7, 2.5 Hz,0.5H), 3.00 (dd, *J* = 8.1, 4.6 Hz, 1H), 2.93 – 2.89 (m, 1H), 2.30 – 1.72 (m, 5H).; **^{13}C{^{1}H}NMR (101 MHz, CDCl$_3$)**: δ 202.4 (202.2), 170.7 (170.5), 141.0 (141.0), 138.3 (138.2), 136.6 (136.2), 132.7 (132.5), 129.9 (129.8), 129.1(128.9), 129.0 (128.8), 128.2 (127.0), 128.0 (127.6), 127.02 (126.98), 78.7 (78.5), 73.0 (72.3), 54.1 (54.0), 50.7 (48.7), 36.7 (34.9), 32.8 (32.7), 26.0 (25.8), 24.6 (24.5); **HRMS (ESI, m/z)**: calcd. For $C_{18}H_{22}NO_3^+$ [M+H]$^+$: 332.1645, found: 332.1655.

Compound 2w (Fig. 2)

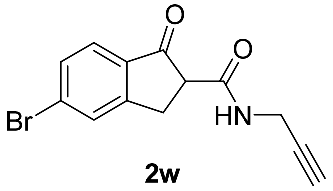

2w

5-bromo-1-oxo-*N*-(prop-2-yn-1-yl)-2,3-dihydro-1*H*-indene-2-carboxamide (2w); **TLC**: R$_f$ = 0.30 (petroleum ether /ethyl acetate = 3/1, v/v, UV); white solid (mp: 180-181°C.); yield: 50%; **^{1}H NMR (400 MHz, CDCl$_3$)**: δ 7.70 (s, 1H), 7.62 – 7.52 (m, 2H), 7.33 (s, 1H), 4.19 – 4.01(m, 2H), 3.78 (dd, *J* = 17.9, 4.2 Hz, 1H), 3.57 (dd, *J* = 8.5, 4.1 Hz, 1H), 3.34 (dd, *J* = 17.9, 8.3 Hz, 1H), 2.25 (s, 1H); **^{13}C{^{1}H}NMR (101 MHz, CDCl$_3$)**: δ 201.8, 165.9, 155.8, 134.2, 131.6, 131.6, 130.2, 125.7, 79.3, 71.8, 52.8, 29.6, 28.4; **HRMS (ESI, m/z)**: calcd. For $C_{13}H_{11}BrNO_2^+$ [M+H]$^+$: 291.9968, found: 291.9976.

Compound 2x (Fig. 2)

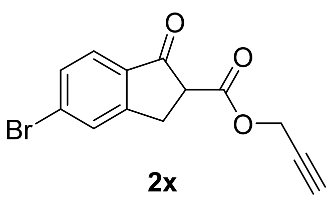

2x

prop-2-yn-1-yl 5-bromo-1-oxo-2,3-dihydro-1*H*-indene-2-carboxylate (2x); **TLC**: R*f* = 0.30 (petroleum ether /ethyl acetate = 5/1, v/v, UV); white solid (mp: 119-120°C.); yield: 45%; **^{1}H NMR (400 MHz, CDCl$_3$)**: δ 7.69 (d, *J* = 1.5 Hz, 1H), 7.63-7.61 (m, 1H), 7.55 – 7.51 (m, 1H), 4.86 – 4.73 (m, 2H), 3.77 (dd, *J* = 8.3, 4.2 Hz, 1H), 3.59 – 3.54 (m, 1H), 3.38 (dd, *J* = 17.5, 8.3 Hz, 1H), 2.54-2.50 (m, 1H); **^{13}C{^{1}H}NMR (101 MHz, CDCl$_3$)**: δ 197.6, 168.1, 155.0 (145.2), 134.1 (135.7), 131.8 (131.2), 130.0 (130.4), 126.0 (128.3), 122.2 (124.4), 77.8, 75.6 (75.3), 53.3, 53.0 (51.8), 30.0 (32.5); **HRMS (ESI, m/z)**: calcd. For $C_{13}H_{10}BrO_3^+$ [M+H]$^+$: 292.9808, found: 292.9810.

Compound 4a (Fig. 3)

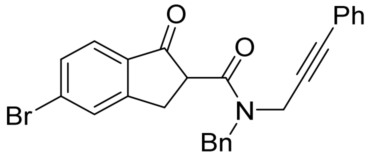

4a

***N*-benzyl-5-bromo-1-oxo-*N*-(3-phenylprop-2-yn-1-yl)-2,3-dihydro-1*H*-indene-2-carboxamide (4a)**; **TLC**: R*f* = 0.60 (petroleum ether /ethyl acetate = 5/1, v/v, UV); white solid (mp: 79-80°C.); yield: 78%; Enol isomerization were observed by NMR; **^{1}H NMR (400 MHz, CDCl$_3$)**: δ 7.70 (dd, *J* = 19.5, 1.5 Hz, 1H), 7.62-7.49 (m, 2H), 7.42-7.26 (m, 10H), 5.35-5.22 (m, 1H), 5.11-4.93 (m, 1H), δ 4.54-4.34=8 (m, 2H), 4.17-4.04 (m, 1H), 3.93-3.74 (m, 1H), 3.37-3.13 (m, 1H); **^{13}C{^{1}H}NMR (101 MHz, CDCl$_3$)**: δ 200.6 (200.2), 167.9 (168.4), 156.4 (156.2), 136.61 (136.58), 134.2 (134.3), 131.8 (131.4), 131.1 (131.0), 130.0 (129.9), 128.8 (129.1), 128.5 (128.8), 128.3 (128.5), 128.1, 127.6 (127.9), 126.9, 125.7, 122.3 (122.8), 84.8 (84.5), 83.9 (84.0), 51.3 (49.6), 51.1 (50.9), 38.1 (36.4), 30.6 (30.8); **HRMS (ESI, m/z)**: calcd. For $C_{26}H_{21}BrNO_2^+$ [M+H]$^+$: 458.0751, found: 458.0750.

Compound 4b (Fig. 3)

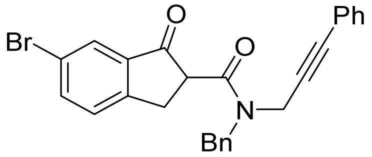

4b

***N*-benzyl-6-bromo-1-oxo-*N*-(3-phenylprop-2-yn-1-yl)-2,3-dihydro-1*H*-indene-2-**

carboxamide (4b); **TLC**: R*f* = 0.50 (petroleum ether /ethyl acetate = 5/1, v/v, UV); yellow oil; yield: 31%; Enol isomerization were observed by NMR; **^{1}H NMR (400 MHz, $CDCl_3$)**: δ 7.86 (dd, *J* = 9.6, 1.9 Hz, 1H), 7.73-7.67 (m, 1H), 7.42-7.26 (m, 11H), 5.34-5.22 (m, 1H), 5.01-4.93 (m, 1H), 4.54-4.40 (m, 2H), 4.18-4.09 (m, 1H), 3.87-3.69 (m, 1H), 3.33-3.09 (m, 1H); **^{13}C{^{1}H}NMR (101 MHz, $CDCl_3$)**: δ 200.4 (200.0), 167.8 (168.4), 153.5 (153.2), 138.2 (138.1), 137.1 (137.2), 136.6, 131.8, 128.8 (129.1), 128.8 (128.4), 128.5 (128.3), 128.2 (128.1), 128.0 (127.4), 127.6 (127.9), 126.9 (127.4), 122.3 (122.7), 121.8, 84.8 (84.5), 83.9 (83.9), 51.5 (49.6), 51.4 (50.9), 38.1 (36.4), 30.5 (30.8); **HRMS (ESI, m/z)**: calcd. For $C_{26}H_{21}BrNO_2^+$ $[M+H]^+$: 458.0751, found: 458.0753.

Compound 4c (Fig. 3)

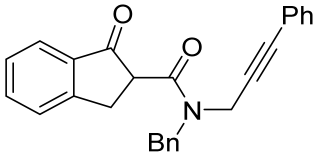

4c

***N*-benzyl-1-oxo-*N*-(3-phenylprop-2-yn-1-yl)-2,3-dihydro-1*H*-indene-2-carboxamide (4c)**; **TLC**: R*f* = 0.50 (petroleum ether /ethyl acetate = 5/1, v/v, UV); white solid (mp: 76-77°C.); yield: 39%; Enol isomerization were observed by NMR; **^{1}H NMR (400 MHz, $CDCl_3$)**: δ 7.76 (t, *J* = 8.3 Hz, 1H), 7.61 (dt, *J* = 11.8, 7.4 Hz, 1H), 7.51 (dd, *J* = 19.5, 7.7 Hz, 1H), 7.43-7.26 (m, 11H), 5.37-5.25 (m, 1H), 5.15-4.95 (m, 1H), 4.56-4.38-4.34 (m, 2H), 4.20-4.05 (m, 1H), 3.95-3.76 (m, 1H), 3.40-3.16 (m, 1H); **^{13}C{^{1}H}NMR (101 MHz, $CDCl_3$)**: δ 201.9 (201.5), 168.4 (168.9), 154.9 (154.6), 136.68 (136.71), 135.5, 135.4 (135.3), 131.83 (131.82), 128.8 (129.0), 128.7 (128.4), 128.5 (128.3), 128.0 (127.7), 127.6 (127.8), 126.9, 126.7 (126.6), 124.6, 122.4 (122.8), 84.6 (84.4), 84.0 (84.1), 51.2 (49.5), 51.1 (50.9), 38.1 (36.3), 30.8 (31.1); **HRMS (ESI, m/z)**: calcd. For $C_{26}H_{22}NO_2^+$ $[M+H]^+$: 380.1646, found: 380.1648.

Compound 4d (Fig. 3)

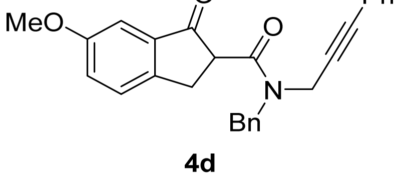

4d

***N*-benzyl-6-methoxy-1-oxo-*N*-(3-phenylprop-2-yn-1-yl)-2,3-dihydro-1*H*-indene-2-carboxamide (4d)**; **TLC**: R_f = 0.40 (petroleum ether /ethyl acetate = 5/1, v/v, UV); yellow oi; yield: 81%; Enol isomerization were observed by NMR; **^{1}H NMR (400 MHz, $CDCl_3$)**: δ 7.42-7.22 (m, 11H), 7.18 (td, *J* = 9.4, 2.9 Hz, 2H), 5.34-5.23 (m, 1H), 5.12-4.94 (m, 1H), 4.56-4.39-4.37 (m, 2H), 4.16 (d, *J* = 19.0 Hz, 0.6H), 4.08 (dd, *J* = 7.7, 3.5 Hz, 0.4H), 3.82 (d, *J* = 7.4 Hz, 3.4H), 3.72-3.59 (m, 0.6H), 3.32-3.08 (m, 1H); **$^{13}C\{^1H\}$NMR (101 MHz, $CDCl_3$)**: δ 201.8 (201.4), 168.4 (169.0), 159.66 (159.65), 147.9 (147.6), 136.72 (136.70), 136.5 (136.6), 131.83 (131.82), 128.8 (129.0), 128.7 (128.4), 128.5 (128.3), 128.0 (127.0), 127.5 (127.8), 127.3 (127.2), 125.0 (124.9), 122.4 (122.8), 105.6, 84.6 (84.4), 84.0 (84.1), 55.7, 51.9 (49.5), 51.8 (50.91, 38.1 (36.3), 30.2 (30.5); **HRMS (ESI, m/z)**: calcd. For $C_{27}H_{24}NO_3^+$ $[M+H]^+$: 410.1751, found: 410.1752.

Compound 4e (Fig. 3)

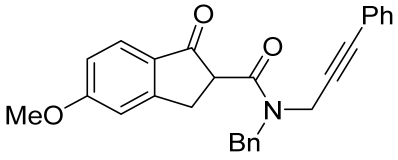

4e

***N*-benzyl-5-methoxy-1-oxo-*N*-(3-phenylprop-2-yn-1-yl)-2,3-dihydro-1*H*-indene-2-carboxamide (4e)**; **TLC**: R_f = 0.30 (petroleum ether /ethyl acetate = 5/1, v/v, UV); yellow oil; yield: 95%; Enol isomerization were observed by NMR; **^{1}H NMR (400 MHz, $CDCl_3$)**: δ 7.67 (t, *J* = 8.8 Hz, 1H), 7.42-7.39 (m, 3H), 7.36-7.31 (m, 5H), 7.30-7.26 (m, 2H), 6.95-6.88 (m, 2H), 5.41-5.25 (m, 1H), 5.21-4.94 (m, 1H), 4.55-4.37 (m, 2H), 4.17-4.04 (m, 1H), 3.87 (d, *J* = 11.6 Hz, 3.6H), 3.74 (dd, *J* = 16.8, 3.6 Hz, 0.4H), 3.32-3.09 (m, 1H); **$^{13}C\{^1H\}$NMR (101 MHz, $CDCl_3$)**: δ 199.8 (199.4), 168.6 (169.1), 166.0 (165.9), 158.0 (157.8), 136.8 (136.7), 131.8, 128.7 (128.9), 128.7 (128.6), 128.5 (128.3), 128.4 (128.3), 128.0 (126.9), 127.5 (127.7), 126.20 (126.18), 122.4 (122.8), 116.0 (115.9), 109.52 (109.49), 84.5 (84.3), 84.2, 55.78 (55.76), 51.3 (49.4), 51.2 (50.9), 38.0 (36.3), 30.8 (31.1); **HRMS (ESI, m/z)**: calcd. For $C_{27}H_{24}NO_3^+$ $[M+H]^+$: 410.1751, found: 410.1755.

Compound 4f (Fig. 3)

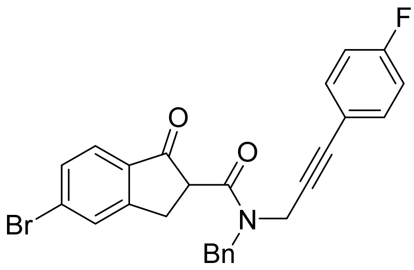

4f

***N*-benzyl-5-bromo-*N*-(3-(4-fluorophenyl)prop-2-yn-1-yl)-1-oxo-2,3-dihydro-1*H*-indene-2-carboxamide (4f)**; **TLC**: R_f = 0.50 (petroleum ether /ethyl acetate = 5/1, v/v, UV); white solid (mp: 94-95°C.); yield: 53%; Enol isomerization were observed by NMR; **^{1}H NMR (400 MHz, $CDCl_3$)**: δ 7.69 (d, *J* = 19.3 Hz, 1H), 7.59 (t, *J* = 8.8 Hz, 1H), 7.51 (t, *J* = 9.0 Hz, 1H), 7.43 – 7.24 (m, 7H), 7.03 – 6.95 (m, 2H), 5.36-5.18 (m, 1H), 5.09-4.90 (m, 1H), 4.51 (d, *J* = 5.4 Hz, 1H), 4.43 (d, *J* = 15.1 Hz, 0.4H), 4.36 (dd, *J* = 7.9, 3.6 Hz, 0.6H), 4.15 (d, *J* = 19.0 Hz, 0.6H), 4.05 (dd, *J* = 7.9, 3.6 Hz, 0.4H), 3.92-3.74 (m, 1H), 3.36-3.12 (m, 1H); **^{13}C{^{1}H}NMR (101 MHz, $CDCl_3$)**: δ 200.6 (200.2), 167.9 (168.4), 163.9 (d, *J* = 18.2 Hz) [161.5 (d, *J* = 17.5 Hz)], 156.4 (156.2), 136.6, 134.15 (134.23), 133.8 (d, *J* = 2.5 Hz) [133.7 (d, *J* = 2.4 Hz)], 131.43 (131.41), 131.1 (131.0), 130.0 (129.9), 128.8 (129.1), 128.1 (126.8), 127.7 (127.9), 125.7 (125.7), 118.4 (d, *J* = 3.7 Hz) [118.8 (d, *J* = 3.6 Hz)], 115.7 (t, *J* = 21.9 Hz), 83.7 (83.6), 83.6 (83.3), 51.2 (49.6), 51.1 (51.0), 38.0 (36.4), 30.6 (30.8); **^{19}FNMR (376 MHz, Chloroform-*d*)**: δ -110.1 (-110.8); **HRMS (ESI, m/z)**: calcd. For $C_{26}H_{20}BrFNO_2^+$ $[M+H]^+$: 476.0656, found: 476.0658.

Compound 4g (Fig. 3)

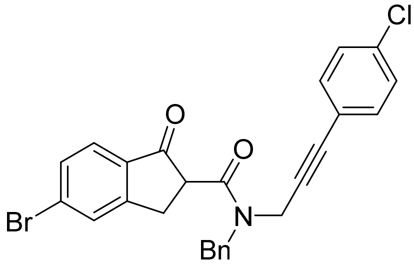

4g

***N*-benzyl-5-bromo-*N*-(3-(4-chlorophenyl)prop-2-yn-1-yl)-1-oxo-2,3-dihydro-1*H*-indene-2-carboxamide (4g)**; **TLC**: R_f = 0.50 (petroleum ether /ethyl acetate = 5/1, v/v,

UV); white solid (mp: 111-112°C.); yield: 46%; Enol isomerization were observed by NMR; **^{1}H NMR (400 MHz, CDCl$_3$)**: δ 7.71 (d, *J* = 19.1 Hz, 1H), 7.65-7.49 (m, 2H), 7.43-7.23 (m, 9H), 5.37-5.19 (m, 1H), 5.11-4.90 (m, 1H), 4.53 (d, *J* = 3.7 Hz, 1H), 4.43 (d, *J* = 15.1 Hz, 0.5H), 4.37 (dd, *J* = 7.9, 3.6 Hz, 0.5H), 4.17 (d, *J* = 19.1 Hz, 0.5H), 4.07 (dd, *J* = 7.9, 3.7 Hz, 0.5H), 3.94-3.76 (m, 1H), 3.37-3.14 (m, 1H); **^{13}C{^{1}H}NMR (101 MHz, CDCl$_3$)**: δ 200.6 (200.2), 167.9 (168.4), 156.4 (156.2), 136.5, 134.9 (134.5), 134.1 (134.2), 133.1, 131.47 (131.45), 131.2 (131.0), 130.0 (129.9), 128.9 (129.1), 128.9 (128.7), 128.1 (126.8), 127.7 (128.0), 125.77 (125.75), 120.8 (121.2), 84.9 (85.1), 83.7, (83.3), 51.2 (49.7), 51.1 (51.1), 38.1 (36.5), 30.6 (30.8); **HRMS (ESI, m/z)**: calcd. For $C_{26}H_{20}BrClNO_2^+$ [M+H]$^+$: 492.0361, found: 492.0363.

Compound 4h (Fig. 3)

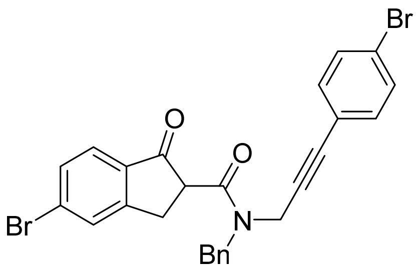

4h

***N*-benzyl-5-bromo-*N*-(3-(4-bromophenyl)prop-2-yn-1-yl)-1-oxo-2,3-dihydro-1*H*-indene-2-carboxamide (4h)**; **TLC**: R*f* = 0.50 (petroleum ether /ethyl acetate = 5/1, v/v, UV); white solid (mp: 113-114°C.); yield: 29%; Enol isomerization were observed by NMR; **^{1}H NMR (400 MHz, CDCl$_3$)**: δ 7.69 (d, *J* = 19.1 Hz, 1H), 7.59 (t, *J* = 8.5 Hz, 1H), 7.51 (t, *J* = 8.8 Hz, 1H), 7.48-7.21 (m, 8H), 7.18 (d, *J* = 8.5 Hz, 1H), 5.36-5.18 (m, 1H), 5.09-4.89 (dd, *J* = 63.8, 18.2 Hz, 1H), 4.51 (d, *J* = 2.7 Hz, 1H), 4.42 (d, *J* = 15.2 Hz, 0.5H), 4.35 (dd, *J* = 7.8, 3.7 Hz, 0.5H), 4.15 (d, *J* = 19.1 Hz, 0.5H), 4.05 (dd, *J* = 7.8, 3.6 Hz, 0.5H), 3.92-3.74 (m , 1H), 3.36-3.13 (m, 1H); **^{13}C{^{1}H}NMR (101 MHz, CDCl$_3$)**: δ 200.6 (200.2), 167.8 (168.4), 156.4 (156.2), 136.5, 134.2 (134.1), 133.30 (133.27), 131.8 (131.6), 131.5 (131.4), 131.2 (131.0), 130.0 (129.9), 128.9 (129.1), 128.1 (126.8), 127.7 (128.0), 125.8 (125.7), 123.1 (122.7), 121.2 (121.7), 85.1 (85.3), 83.7 (83.4), 51.2 (49.7), 51.1 (51.1), 38.1 (36.5), 30.6 (30.8); **HRMS (ESI, m/z)**: calcd. For $C_{26}H_{20}Br_2NO_2^+$ [M+H]$^+$: 537.9835, found: 537.9838.

Compound 4i (Fig. 3)

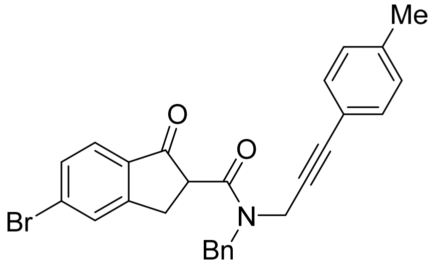

4i

***N*-benzyl-5-bromo-1-oxo-*N*-(3-(p-tolyl)prop-2-yn-1-yl)-2,3-dihydro-1*H*-indene-2-carboxamide (4i)**; **TLC**: R*f* = 0.60 (petroleum ether /ethyl acetate = 5/1, v/v, UV); white solid (mp: 120-121°C.); yield: 71%; Enol isomerization were observed by NMR; **^{1}H NMR (400 MHz, CDCl$_3$)**: δ 7.69 (d, *J* = 19.5 Hz, 1H), 7.59 (t, *J* = 8.6 Hz, 1H), 7.51 (t, *J* = 9.0 Hz, 1H), 7.45-7.21 (m, 7H), 7.11 (dd, *J* = 17.1, 7.9 Hz, 2H), 534-5.22 (m, 1H), 5.10-5.94 (m, 1H), 4.53 (d, *J* = 2.3 Hz, 1H), 4.45-4.36 (m, 1H), 4.22-4.00 (m, 1H), 3.92-3.73 (m, 1H), 3.36-3.11 (m, 1H), 2.35 (d, *J* = 8.6 Hz, 3H); **^{13}C{^{1}H}NMR (101 MHz, CDCl$_3$)**: δ 200.6 (200.2), 167.9 (168.4), 156.4 (156.2), 139.0 (138.5), 136.6 (136.6), 134.2 (134.3), 131.70 (131.71), 131.4, 131.0 (130.9), 130.0 (129.9), 129.2 (129.1), 128.8 (129.0), 128.0 (126.9), 127.6 (127.8), 125.71 (125.69), 119.2 (119.6), 84.9 (84.6), 83.15 (83.17), 51.2 (49.5), 51.1 (50.8), 38.1 (36.4), 30.6 (30.8), 21.59 (21.56); **HRMS (ESI, m/z)**: calcd. For $C_{27}H_{23}BrNO_2^+$ $[M+H]^+$: 472.0907, found: 472.0908.

Compound 4j (Fig. 3)

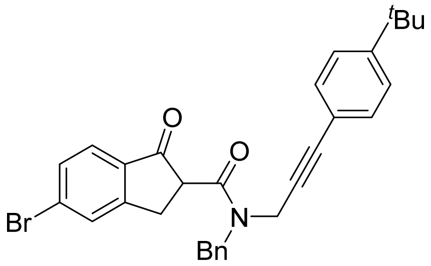

4j

***N*-benzyl-5-bromo-*N*-(3-(4-(tert-butyl)phenyl)prop-2-yn-1-yl)-1-oxo-2,3-dihydro-1*H*-indene-2-carboxamide (4j)**; **TLC**: R*f* = 0.50 (petroleum ether /ethyl acetate = 5/1, v/v, UV); white solid (mp: 91-92°C.); yield: 40%; Enol isomerization were observed

by NMR; **^{1}H NMR (400 MHz, $CDCl_3$)**: δ 7.73-7.65 (m, 1H), 7.60 (t, *J* = 8.5 Hz, 2H), 7.56-7.48 (m, 1H), 7.45-7.25 (m, 9H), 5.34-5.22 (m, 1H), 5.10-4.94 (m, 1H), 4.54 (d, *J* = 3.4 Hz, 1H), 4.46-4.36 (m, 1H), 4.21-4.00 (m, 1H), 3.92-3.74 (m, 1H), 3.36-3.12 (m, 1H), 1.31 (d, *J* = 5.1 Hz, 9H); **$^{13}C\{^1H\}$NMR (101 MHz, $CDCl_3$)**: δ 200.7 (200.2), 168.0 (168.4), 156.5 (156.2), 152.2 (151.7), 136.6 (136.7), 134.2 (134.3), 131.6, 131.4, 131.1 (130.9), 130.0 (129.9), 128.8 (129.1), 128.1 (126.9), 127.6 (127.9), 125.8, 125.5 (125.3), 119.3 (119.7), 84.9 (84.7), 83.2, 51.3 (49.6), 51.2 (50.8), 38.2 (36.5), 34.93 (34.87), 31.3, 30.6 (30.8); **HRMS (ESI, m/z)**: calcd. For $C_{30}H_{29}BrNO_2^+$ $[M+H]^+$: 514.1377, found: 514.1379.

Compound 4k (Fig. 3)

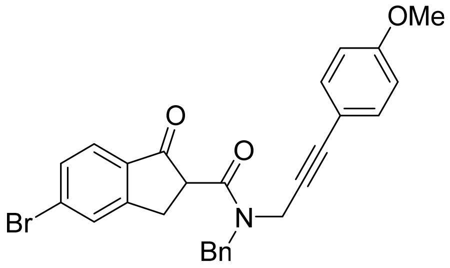

4k

***N*-benzyl-5-bromo-*N*-(3-(4-methoxyphenyl)prop-2-yn-1-yl)-1-oxo-2,3-dihydro-1*H*-indene-2-carboxamide (4k)**; **TLC**: R_f = 0.50 (petroleum ether /ethyl acetate = 5/1, v/v, UV); white solid (mp: 86-87°C.); yield: 33%; Enol isomerization were observed by NMR; **^{1}H NMR (400 MHz, $CDCl_3$)**: δ 7.70 (d, *J* = 19.8 Hz, 1H), 7.66-7.58 (m, 1H), 7.53 (t, *J* = 8.8 Hz, 1H), 7.46-7.26 (m, 7H), 6.85 (dd, *J* = 15.8, 8.8 Hz, 2H), 5.36-5.23 (m, 1H), 5.10-4.95 (m, 1H), 4.54 (s, 1H), 4.48-4.37 (m, 1H), 4.21-4.04 (m, 1H), 3.99-3.67 (m, 4H), 3.38-3.13 (m 1H); **$^{13}C\{^1H\}$NMR (101 MHz, $CDCl_3$)**: δ 200.6 (200.2), 168.0 (168.4), 160.0 (159.7), 156.4 (156.2), 136.65 (136.68), 134.2 (134.3), 133.3, 131.4, 131.0 (130.9), 130.0 (129.9), 128.8 (129.0), 128.0 (126.9), 127.6 (127.8), 125.72 (125.70), 114.3 (114.8), 114.1 (113.9), 84.7 (84.4), 82.5, 55.41 (55.37), 51.2 (49.6), 51.1 (50.9), 38.2 (36.5), 30.6 (30.8); **HRMS (ESI, m/z)**: calcd. For $C_{27}H_{23}BrNO_3^+$ $[M+H]^+$: 488.0856, found: 488.0858.

Compound 4l (Fig. 3)

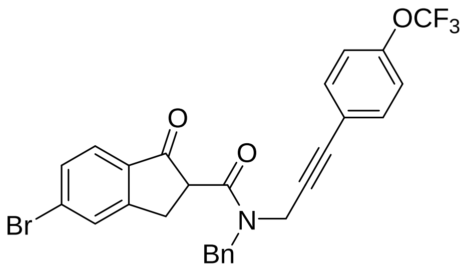

4l

***N*-benzyl-5-bromo-1-oxo-*N*-(3-(4-(trifluoromethoxy)phenyl)prop-2-yn-1-yl)-2,3-dihydro-1*H*-indene-2-carboxamide (4l)**; **TLC**: R$_f$ = 0.50 (petroleum ether /ethyl acetate = 5/1, v/v, UV); white solid (mp: 96-97°C.); yield: 32%; Enol isomerization were observed by NMR; **^{1}H NMR (400 MHz, CDCl$_3$)**: δ 7.69 (d, *J* = 19.0 Hz, 1H), 7.64-7.48 (m, 2H), 7.45-7.24 (m, 7H), 7.15 (dd, *J* = 18.2, 8.2 Hz, 2H), 5.37-5.17 (m, 1H), 5.13-4.87 (m, 1H), 4.52 (d, *J* = 6.5 Hz, 1H), 4.44 (d, *J* = 15.1 Hz, 0.5H), 4.36 (dd, *J* = 7.9, 3.6 Hz, 0.5H), 4.17 (d, *J* = 19.1 Hz, 0.5H), 4.06 (dd, *J* = 7.9, 3.7 Hz, 0.5H), 3.93-3.75 (m, 1H), 3.36-3.13 (m, 1H); **^{13}C{^{1}H}NMR (101 MHz, CDCl$_3$)**: δ 200.6 (200.2), 167.8 (168.4), 156.4 (156.2), 149.3 (149.1), 136.5, 134.1 (134.2), 133.42 (133.39), 131.48 (131.46), 131.2 (131.1), 130.0 (129.9), 128.9 (129.1), 128.1 (126.8), 127.7 (128.0), 125.78 (125.75), 121.1 (121.5), 121.0 (120.9), 120.5 (q, *J* = 258.9 Hz), 84.9 (85.0), 83.4 (83.0), 51.2 (49.7), 51.12 (51.08), 38.0 (36.5), 30.6 (30.8); **^{19}FNMR (376 MHz, Chloroform-*d*)**: δ -57.8; **HRMS (ESI, m/z)**: calcd. For $C_{27}H_{20}BrF_3NO_3^+$ [M+H]$^+$: 542.0574, found: 5542.0576.

Compound 4m (Fig. 3)

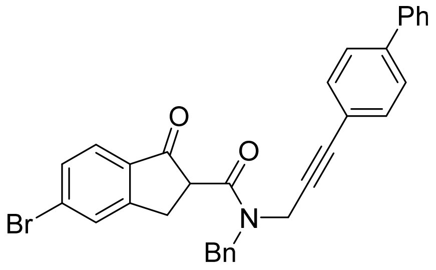

4m

***N*-(3-([1,1'-biphenyl]-4-yl)prop-2-yn-1-yl)-*N*-benzyl-5-bromo-1-oxo-2,3-dihydro-1*H*-indene-2-carboxamide (4m)**; **TLC**: R$_f$ = 0.30 (petroleum ether /ethyl acetate = 5/1, v/v, UV); white solid (mp: 148-149°C.); yield: 63%; Enol isomerization were observed

by NMR; **^{1}H NMR (400 MHz, CDCl$_3$)**: δ 7.70 (d, *J* = 20.4 Hz, 1H), 7.65-7.50 (m, 6H), 7.50-7.26 (m, 10H), 5.37-5.24 (m, 1H), 5.14-4.96 (m, 1H), 4.57 (s, 1H), 4.50-4.37 (m, 1H), 4.24-4.03 (m, 1H), 3.94-3.76 (m, 1H), 3.38-3.13 (m, 1H); **^{13}C{^{1}H}NMR (101 MHz, CDCl$_3$)**: δ 200.6 (200.2), 167.9 (168.4), 156.4 (156.2), 141.5 (141.1), 140.2 (140.4), 136.57 (136.59), 134.16 (134.24), 132.3, 131.39 (131.38), 131.1 (130.9), 130.0 (129.9), 129.0 (129.1), 128.8 (128.9), 128.0 (127.9), 127.6 (127.7), 127.14 (127.09), 127.1, 127.0 (126.9), 125.7 (125.7), 121.1 (121.6), 84.6 (84.5), 84.3, 51.2 (49.6), 51.1 (50.9), 38.1 (36.5), 30.6 (30.8); **HRMS (ESI, m/z)**: calcd. For $C_{32}H_{25}BrNO_2^+$ [M+H]$^+$: 534.1064, found: 534.1065.

Compound 4n (Fig. 3)

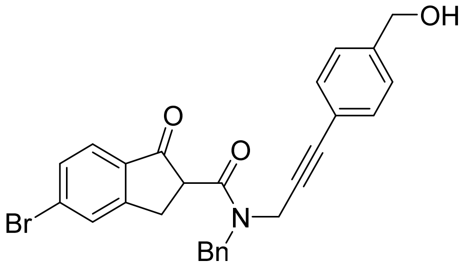

4n

***N*-benzyl-5-bromo-*N*-(3-(4-(hydroxymethyl)phenyl)prop-2-yn-1-yl)-1-oxo-2,3-dihydro-1*H*-indene-2-carboxamide (4n)**; **TLC**: R*f* = 0.30 (petroleum ether /ethyl acetate = 2/1, v/v, UV); white solid (mp: 125-126°C.); yield: 35%; Enol isomerization were observed by NMR; **^{1}H NMR (400 MHz, CDCl$_3$)**: δ 7.68 (d, *J* = 18.7 Hz, 1H), 7.59 (t, *J* = 9.1 Hz, 1H), 7.51 (t, *J* = 9.2 Hz, 1H), 7.43-7.22 (m, 9H), 5.34-5.20 (m, 1H), 5.09-4.92 (m, 1H), 4.75-4.49 (m, 3H), 4.44-4.34 (m, 1H), 4.15 (d, *J* = 19.0 Hz, 0.5H), 4.05 (dd, *J* = 7.9, 3.7 Hz, 0.5H), 3.90-3.72 (m, 1H), 3.36-3.11 (m, 1H), 2.06 (s, 1H); **^{13}C{^{1}H}NMR (101 MHz, CDCl$_3$)**: δ 200.7 (200.3), 168.0 (168.5), 156.4 (156.2), 141.8 (141.4), 136.51 (136.49), 134.2 (134.1), 131.96 (131.95), 131.42 (131.41), 131.1 (131.0), 130.0 (129.9), 128.8 (129.1), 128.0, 127.6 (127.9), 126.9 (126.7), 125.73 (125.71), 121.3 (121.8), 84.6 (84.3), 83.8 (83.9), 64.8 (64.9), 51.2 (51.1), 49. (51.0), 38.1 (36.5), 30.5 (30.8); **HRMS (ESI, m/z)**: calcd. For $C_{27}H_{23}BrNO_3^+$ [M+H]$^+$: 488.0856, found: 488.0859.

Compound 4o (Fig. 3)

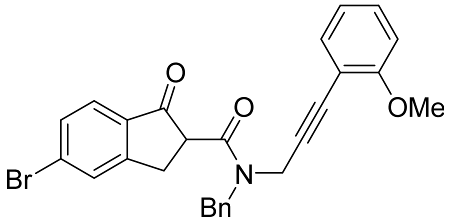

4o

***N*-benzyl-5-bromo-*N*-(3-(2-methoxyphenyl)prop-2-yn-1-yl)-1-oxo-2,3-dihydro-1*H*-indene-2-carboxamide (4o)**; **TLC**: R*f* = 0.50 (petroleum ether /ethyl acetate = 5/1, v/v, UV); white solid (mp: 46-47°C.); yield: 89%; Enol isomerization were observed by NMR; **^{1}H NMR (400 MHz, CDCl$_3$)**: δ 7.68 (d, *J* = 20.9 Hz, 1H), 7.63-7.54 (m, 1H), 7.55-7.45 (m, 1H), 7.45-7.20 (m, 7H), 6.95-6.82 (m, 2H), 5.33-5.26 (m, 1H), 5.13-5.01 (m, 1H), 4.59 (d, *J* = 5.8 Hz, 1H), 4.50-4.37 (m, 1H), 4.23-4.02 (m, 1H), 3.95-3.69 (m, 4H), 3.36-3.10 (m, 1H); **^{13}C{^{1}H}NMR (101 MHz, CDCl$_3$)**: δ 200.7 (200.2), 168.1 (168.4), 160.3, 156.4 (156.2), 136.6 (136.7), 134.2 (134.3), 133.7 (133.8), 131.4, 131.0 (130.9), 130.2, 129.9 (129.9), 128.8 (129.0), 128.0 (127.1), 127.5 (127.8), 125.7 (125.7), 120.6 (120.4), 111.5 (111.9), 110.72 (110.67), 87.92 (87.88), 81.3 (81.2), 55.80 (55.78), 51.3 (49.5), 51.1 (50.6), 38.4 (36.5), 30.6 (30.8); **HRMS (ESI, m/z)**: calcd. For $C_{27}H_{23}BrNO_3^+$ $[M+H]^+$: 488.0856, found: 488.0853.

Compound 4p (Fig. 3)

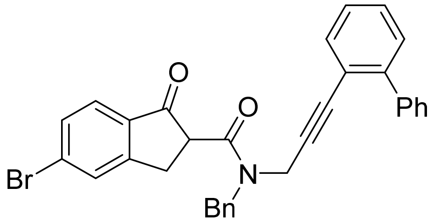

4p

***N*-(3-([1,1'-biphenyl]-2-yl)prop-2-yn-1-yl)-*N*-benzyl-5-bromo-1-oxo-2,3-dihydro-1*H*-indene-2-carboxamide (4p)**; **TLC**: R*f* = 0.60 (petroleum ether /ethyl acetate = 5/1, v/v, UV); white solid (mp: 42-43°C.); yield: 60%; Enol isomerization were observed by NMR; **^{1}H NMR (400 MHz, CDCl$_3$)**: δ 7.68 (dd, *J* = 5.0, 1.5 Hz, 1H), 7.61-7.46 (m, 5H), 7.46-7.16 (m, 11H), 5.14 (d, *J* = 15.2 Hz, 0.7H), 5.05 (d, *J* = 17.1 Hz, 0.3H), 4.97 (dd, *J* = 19.1, 1.2 Hz, 0.7H), 4.69 (d, *J* = 17.1 Hz, 0.3H), 4.46 (d, *J* = 17.5 Hz, 0.3H),

4.33 (d, J = 17.5 Hz, 0.3H), 4.16 (d, J = 15.2 Hz, 0.7H), 4.09-3.93 (m, 1.7H), 3.76-3.69 (m, 1H), 3.17-2.93 (m, 1H); **^{13}C{^{1}H}NMR (101 MHz, CDCl$_3$)**: δ 200.7 (200.1), 167.8 (168.3), 156.5 (156.2), 144.5 (144.2), 140.6(140.7), 136.6 (136.5), 134.2 (134.3), 133.1 (133.4), 131.3 (131.4), 131.0 (130.9), 129.93 (129.91), 129.7 (129.6), 129.3 (129.4), 129.1 (129.0), 128.7 (128.8), 128.1 (128.2), 128.0 (127.8), 127.7 (127.), 127.5 (127.3), 127.1 (126.9), 125.68 (125.72), 120.7 (121.1), 86.83 (86.76), 84.4 (84.1), 51.2 (51.0), 49.5 (50.5), 38.0 (36.1), 30.4 (30.8); **HRMS (ESI, m/z)**: calcd. For $C_{32}H_{25}BrNO_2^+$ $[M+H]^+$: 534.1064, found: 534.1065.

Compound 4q (Fig. 3)

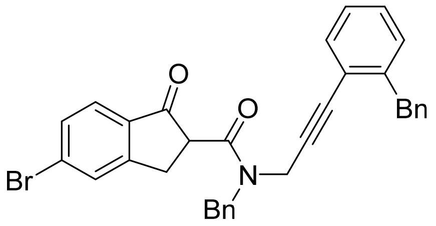

4q

***N*-benzyl-*N*-(3-(2-benzylphenyl)prop-2-yn-1-yl)-5-bromo-1-oxo-2,3-dihydro-1*H*-indene-2-carboxamide (4q)**; **TLC**: R$_f$ = 0.50 (petroleum ether /ethyl acetate = 10/1, v/v, UV); white solid (mp: 79-80°C.); yield: 54%; Enol isomerization were observed by NMR; **^{1}H NMR (400 MHz, CDCl$_3$)**: δ 7.67 (d, J = 6.7 Hz, 1H), 7.63-7.47 (m, 2H), 7.44-7.08 (m, 14H), 5.20-5.05 (m, 1.5H), 4.85 (d, J = 17.2 Hz, 0.5H), 4.53 (q, J = 17.4 Hz, 1H), 4.30 (d, J = 15.1 Hz, 0.6H), 4.21 (dd, J = 7.8, 3.6 Hz, 0.6H), 4.17-4.07 (m, 2.4H), 4.03 (dd, J = 7.9, 3.6 Hz, 0.4H), 3.83-3.70 (m, 1H), 3.13 (dt, J = 17.1, 8.2 Hz, 1H); **^{13}C{^{1}H}NMR (101 MHz, CDCl$_3$)**: δ 168.4 (167.8), 156.2 (156.5), 143.2 (143.0), 140.4 (140.6), 136.6 (136.5), 134.2 (134.3), 132.7 (132.6), 131.4, 131.1 (130.9), 129.9 (130.0), 129.5, 129.1, 128.8, 128.7, 128.54 (128.50), 128.0, 127.9 (127.6), 126.9, 126.5 (126.2), 126.2 (126.1), 125.73 (125.72), 122.6 (122.2), 87.9, 83.6 (83.3), 51.2 (51.1), 49.6 (50.8), 40.4 (40.2), 38.1 (36.4), 30.5 (30.8); **HRMS (ESI, m/z)**: calcd. For $C_{33}H_{27}BrNO_2^+$ $[M+H]^+$: 548.1220, found: 548.1222.

Compound 4r (Fig. 3)

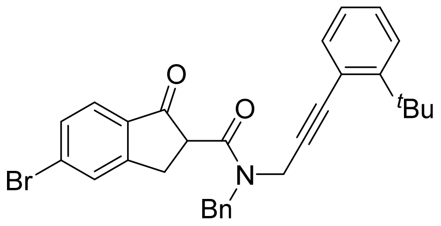

4r

***N*-benzyl-5-bromo-*N*-(3-(2-(*tert*-butyl)phenyl)prop-2-yn-1-yl)-1-oxo-2,3-dihydro-1*H*-indene-2-carboxamide (4r)**; **TLC**: R_f = 0.50 (petroleum ether /ethyl acetate = 5/1, v/v, UV); yellow oil; yield: 30%; Enol isomerization were observed by NMR; **^{1}H NMR (400 MHz, Chloroform-*d*)** δ 7.72 – 7.68 (m, 1H), 7.63- 7.58(m, 1H), 7.56-7.49 (m, 1H), 7.43 – 7.22 (m, 8H), 7.17 – 7.09 (m, 1H), 5.35 (dd, *J* = 16.2, 4.1 Hz, 1H), 5.20 – 5.14 (m, 0.6H), 4.98 – 4.94 (m, 0.4H), 4.66 (d, *J* = 17.4 Hz, 0.4H), 4.51 (d, *J* = 20.0 Hz, 0.4H), 4.45 – 4.41 (m, 0.7H), 4.33 (d, *J* = 15.2 Hz, 0.6H), 4.18 (d, *J* = 19.1 Hz,0.6H), 4.07 (dd, *J* = 7.9, 3.6 Hz, 0.3H), 3.90 (dd, *J* = 17.3, 3.6 Hz, 0.6H), 3.78 (dd, *J* = 17.3, 3.6 Hz, 0.4H), 3.30 (dd, *J* = 17.3, 7.8 Hz, 0.6H), 3.17 (dd, *J* = 17.1, 7.8 Hz, 0.4H), 1.48 (d, *J* = 7.9 Hz, 9H); **^{13}C{^{1}H}NMR (101 MHz, $CDCl_3$)**: δ 200.6 (200.1), 167.9 (168.3), 156.5 (156.2), 151.66 (151.73), 136.6 (136.5), 135.55 (135.64), 134.1 (134.3), 131.5 (131.4), 131.2 (130.9), 130.0 (129.9), 129.1 (128.87), 128.89 (128.5), 128.0, 127.7 (127.9), 126.8, 125.76 (125.79), 125.6 (125.7), 120.5 (121.0), 89.5 (89.6), 86.2 (85.6), 51.2 (49.6), 51.1 (50.9), 38.3 (36.6), 35.9, 30.5 (30.8), 30.1; **HRMS (ESI, m/z)**: calcd. For $C_{30}H_{29}BrNO_2^+$ $[M+H]^+$: 514.1376, found: 514.1380.

Compound 4s (Fig. 3)

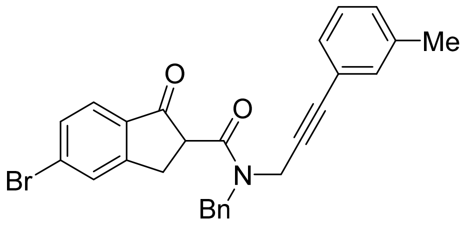

4s

***N*-benzyl-5-bromo-1-oxo-*N*-(3-(m-tolyl)prop-2-yn-1-yl)-2,3-dihydro-1*H*-indene-2-carboxamide (4s)**; **TLC**: R_f = 0.50 (petroleum ether /ethyl acetate = 5/1, v/v, UV); yellow oil; yield: 23%; Enol isomerization were observed by NMR; **^{1}H NMR (400**

MHz, Chloroform-*d*) δ 7.69 (d, *J* = 21.0 Hz, 1H), 7.65-7.47 (m, 2H), 7.47-7.26 (m, 5H), 7.28-7.08 (m, 4H), 5.35-5.22 (m, 1H), 5.14-4.90 (m, 1H), 4.65-4.27 (m, 2H), 4.14 (d, *J* = 19.0 Hz, 0.6H), 4.05 (dd, *J* = 7.9, 3.7 Hz, 0.4H), 3.93-3.74 (m, 1H), 3.37-3.12 (m, 1H), 2.32 (d, *J* = 12.0 Hz, 3H); **$^{13}C\{^1H\}$NMR (101 MHz, $CDCl_3$)**: δ 200.6 (200.2), 167.9 (168.4), 156.5 (156.2), 138.2 (138.0), 136.6 (136.7), 134.2 (134.3), 132.4 (132.5), 131.4, 131.1 (131.0), 130.0 (129.9), 129.7 (129.4), 128.9 (129.1), 128.8 (128.4), 128.1 (128.2), 127.9 (127.6), 126.9, 125.76 (125.75), 122.6 (122.1), 85.0 (84.7), 83.5 (83.6), 51.3 (51.2), 50.9 (49.6), 38.1 (36.5), 30.6 (30.8), 21.33 (21.30); **HRMS (ESI, m/z)**: calcd. For $C_{27}H_{23}BrNO_2^+$ $[M+H]^+$: 472.0907, found: 472.0909.

Compound 4t (Fig. 3)

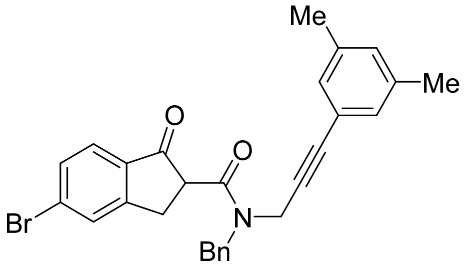

4t

***N*-benzyl-5-bromo-*N*-(3-(3,5-dimethylphenyl)prop-2-yn-1-yl)-1-oxo-2,3-dihydro-1*H*-indene-2-carboxamide (4t)**; **TLC**: R_f = 0.50 (petroleum ether /ethyl acetate = 10/1, v/v, UV); white solid (mp: 101-102°C.); yield: 48%; Enol isomerization were observed by NMR; **^1H NMR (400 MHz, $CDCl_3$)**: δ 7.69 (d, *J* = 19.6 Hz, 1H), 7.60 (t, *J* = 8.2 Hz, 1H), 7.55-7.47 (m, 1H), 7.45-7.24 (m, 5H), 7.13-6.89 (m, 3H), 5.34-5.23 (m, 1H), 5.09-4.93 (m, 1H), 4.60-4.29 (m, 2H), 4.21-4.00 (m, 1H), 3.95-3.63 (m, 1H), 3.36-3.12 (m, 1H), 2.28 (d, *J* = 12.0 Hz, 6H); **$^{13}C\{^1H\}$NMR (101 MHz, $CDCl_3$)**: δ 200.6 (200.2), 167.9 (168.4), 156.5 (156.2), 138.1 (137.9), 136.6 (136.7), 134.2 (134.3), 131.4, 131.1 (130.9), 130.7 (130.3), 130.0 (129.9), 129.5 (128.8), 128.0 (129.1), 127.8 (127.6), 126.9, 125.73 (125.71), 121.9 (122.3), 85.1 (84.9), 83.2 (83.1), 51.2 (51.1), 49.5 (50.8), 38.1 (36.4), 30.6 (30.8), 21.20 (21.17); **HRMS (ESI, m/z)**: calcd. For $C_{28}H_{25}BrNO_2^+$ $[M+H]^+$: 486.1064, found: 486.1066.

Compound 4u (Fig. 3)

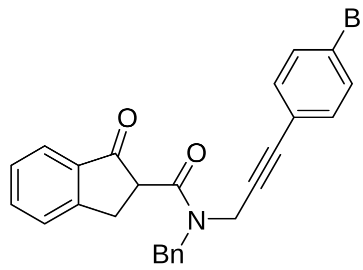

4u

***N*-benzyl-*N*-(3-(4-bromophenyl)prop-2-yn-1-yl)-1-oxo-2,3-dihydro-1*H*-indene-2-carboxamide (4u)**; **TLC**: R$_f$ = 0.50 (petroleum ether /ethyl acetate = 5/1, v/v, UV); white solid (mp: 102-103°C.); yield: 58%; Enol isomerization were observed by NMR; **^{1}H NMR (400 MHz, CDCl$_3$)**: δ 7.75 (t, *J* = 8.3 Hz, 1H), 7.65-7.58 (m, 1H), 7.55-7.12 (m, 11H), 5.36 (d, *J* = 17.3 Hz, 0.5H), 5.22 (d, *J* = 15.2 Hz, 0.5H), 5.11 (dd, *J* = 19.0, 1.3 Hz, 0.5H), 4.92 (d, *J* = 17.2 Hz, 0.5H), 4.52 (s, 1H), 4.43 (d, *J* = 15.2 Hz, 0.5H), 4.36 (dd, *J* = 7.8, 3.7 Hz, 0.5H), 4.16 (d, *J* = 19.0 Hz, 0.5H), 4.05 (d, *J* = 3.8 Hz, 0.5H), 3.95-3.77 (m, 1H), 3.39-3.17 (m, 1H); **^{13}C{^{1}H}NMR (101 MHz, CDCl$_3$)**: δ 201.8 (201.5), 168.3 (168.9), 154.9 (154.7), 136.6 (136.7), 135.6 (135.5), 135.3 (135.4), 133.28 (133.31), 131.8 (131.6), 128.8 (129.1), 128.1 (127.8), 127.7 (127.9), 126.9 (127.6), 126.7 (126.6), 124.63 (124.62), 123.1 (122.7), 121.8, (121.3), 85.3 (85.4), 83.6 (83.3), 51.2 (51.1), 49.6 (51.1), 38.1 (36.4), 30.8 (31.1); **HRMS (ESI, m/z)**: calcd. For $C_{26}H_{21}BrNO_2^+$ $[M+H]^+$: 458.0751, found: 458.0752.

Compound 4v (Fig. 3)

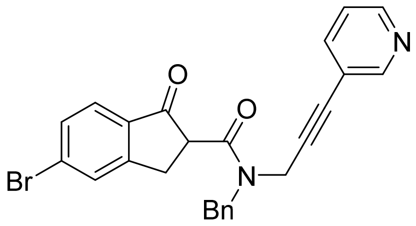

4v

***N*-benzyl-5-bromo-1-oxo-*N*-(3-(pyridin-3-yl)prop-2-yn-1-yl)-2,3-dihydro-1*H*-indene-2-carboxamide (4v)**; **TLC**: R$_f$ = 0.30 (petroleum ether /ethyl acetate = 2/1, v/v, UV); yellow solid (mp: 98-99°C.); yield: 25%; Enol isomerization were observed by NMR; **^{1}H NMR (400 MHz, CDCl$_3$)**: δ 8.55 (t, *J* = 21.0 Hz, 2H), 7.75-7.46 (m, 4H), 7.45-7.17 (m, 6H), 5.35 (d, *J* = 17.3 Hz, 0.5H), 5.24-5.06 (m, 1H), 4.90 (d, *J* = 17.3 Hz,

0.5H), 4.53 (s, 1H), 4.46 (d, J = 15.1 Hz, 0.5H), 4.35 (dd, J = 7.8, 3.6 Hz, 0.5H), 4.19 (d, J = 19.0 Hz, 0.5H), 4.07 (dd, J = 7.9, 3.6 Hz, 0.5H), 3.93-3.75 (m, 1H), 3.37-3.14 (m, 1H); **$^{13}C\{^1H\}$NMR (101 MHz, $CDCl_3$)**: δ 200.5 (200.1), 167.8 (168.4), 156.4 (156.2), 152.44 (152.41), 149.1 (148.7), 138.88 (138.85), 136.44 (136.37), 134.2 (134.1), 131.48 (131.45), 131.2 (131.1), 130.0 (129.9), 128.9 (129.1), 128.1, 128.0 (127.7), 125.8 (125.7), 126.8, 123.2 (123.1), 87.7 (87.5), 81.5 (81.0), 51.2, 51.1 (49.7), 38.0 (36.5), 30.7 (30.5); **HRMS (ESI, m/z)**: calcd. For $C_{25}H_{20}BrN_2O_2^+$ $[M+H]^+$: 459.0703, found: 459.0701.

Compound 4w (Fig. 3)

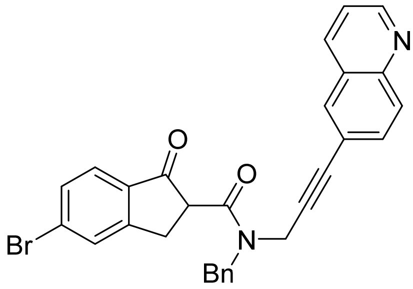

4w

***N*-benzyl-5-bromo-1-oxo-*N*-(3-(quinolin-6-yl)prop-2-yn-1-yl)-2,3-dihydro-1*H*-indene-2-carboxamide (4w)**; **TLC**: R_f = 0.30 (petroleum ether /ethyl acetate = 2/1, v/v, UV); white solid (mp: 116-117°C.); yield: 23%; Enol isomerization were observed by NMR; **^1H NMR (400 MHz, $CDCl_3$)**: δ 8.90 (ddd, J = 10.6, 4.4, 1.7 Hz, 1H), 8.15-7.96 (m, 2H), 7.84 (d, J = 29.4 Hz, 1H), 7.75-7.47 (m, 4H), 7.45-7.23 (m, 6H), 5.38 (d, J = 17.3 Hz, 0.5H), 5.19 (dd, J = 36.9, 17.1 Hz, 1H), 4.96 (d, J = 17.3 Hz, 0.5H), 4.58 (d, J = 3.5 Hz, 1H), 4.47 (d, J = 15.1 Hz, 0.5H), 4.40 (dd, J = 7.8, 3.6 Hz, 0.5H), 4.22 (d, J = 19.1 Hz, 0.5H), 4.07 (dd, J = 7.9, 3.6 Hz, 0.5H), 3.95-3.76 (m, 1H), 3.38-3.14 (m, 1H); **$^{13}C\{^1H\}$NMR (101 MHz, $CDCl_3$)**: δ 200.6 (200.2), 167.8 (168.4), 156.4 (156.2), 151.3 (151.1), 147.9 (147.7), 136.6, 135.9, 134.2 (134.1), 132.3 (132.1), 131.6 (131.5), 131.5 (131.4), 131.2 (131.0), 130.0 (129.9), 129.8 (129.6), 128.9 (129.1), 128.1, 128.0 (127.7), 126.9, 125.8 (125.7), 122.0 (121.8), 120.6 (121.0), 85.4 (85.2), 84.4 (84.0), 51.3 (51.1), 51.1 (49.7), 38.1 (36.5), 30.6 (30.8).; **HRMS (ESI, m/z)**: calcd. For $C_{29}H_{22}BrN_2O_2^+$ $[M+H]^+$: 509.0860, found: 509.0862.

Compound 4x (Fig. 3)

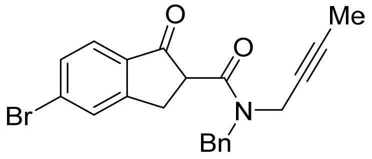

4x

***N*-benzyl-5-bromo-*N*-(but-2-yn-1-yl)-1-oxo-2,3-dihydro-1*H*-indene-2-carboxamide (4x)**; **TLC**: R_f = 0.50 (petroleum ether /ethyl acetate = 5/1, v/v, UV); yellow oil; yield: 91%; Enol isomerization were observed by NMR; **^{1}H NMR (400 MHz, $CDCl_3$)**: δ 7.68 (d, *J* = 21.9 Hz, 1H), 7.57 (t, *J* = 7.2 Hz, 1H), 7.50 (t, *J* = 9.3 Hz, 1H), 7.42-7.19 (m, 5H), 5.23 (dd, *J* = 16.2, 13.0 Hz, 1H), 4.95-4.65 (m, 1H),4.39-4.22 (m, 1.5H), 4.13 (dd, *J* = 17.1, 2.5 Hz, 0.5H), 4.01 (dd, *J* = 7.9, 3.6 Hz, 0.5H), 3.86 (dd, *J* = 16.4, 2.7 Hz, 1H), 3.74 (dd, *J* = 17.3, 3.6 Hz, 0.5H), 3.34-3.09 (m, 1H), 1.80 (dt, *J* = 17.8, 2.4 Hz, 3H); **^{13}C{^{1}H}NMR (101 MHz, $CDCl_3$)**: δ 200.7 (200.3), 167.8 (168.2), 156.4 (156.3), 136.71 (136.68), 134.2 (134.3), 131.4, 131.0 (130.9), 129.94 (129.87), 128.7 (129.0), 127.9 (127.5), 126.8 (127.8), 125.7, 80.8 (80.3), 73.9 (73.7), 51.14 (51.06), 49.2 (50.6), 37.5 (36.0), 30.6 (30.8), 3.70 (3.67); **HRMS (ESI, m/z)**: calcd. For $C_{21}H_{19}BrNO_2^+$ $[M+H]^+$: 396.0594, found: 396.0596.

Compound 4y (Fig. 3)

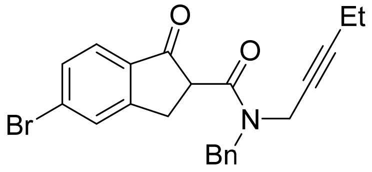

4y

***N*-benzyl-5-bromo-1-oxo-*N*-(pent-2-yn-1-yl)-2,3-dihydro-1*H*-indene-2-carboxamide (4y)**; **TLC**: R_f = 0.30 (petroleum ether /ethyl acetate = 5/1, v/v, UV); white solid (mp: 75-76°C.); yield: 50%; Enol isomerization were observed by NMR; **^{1}H NMR (400 MHz, Chloroform-*d*)** δ 7.68 (d, *J* = 23.3 Hz, 1H), 7.58 (t, *J* = 7.7 Hz, 1H), 7.50 (t, *J* = 8.4 Hz, 1H), 7.42-7.21 (m, 5H), 5.22 (dd, *J* = 28.2, 16.2 Hz, 1H), 4.87 (d, *J* = 17.3 Hz, 0.4H), 4.78 (dq, *J* = 18.7, 2.0 Hz, 0.6H), 4.36-4.17 (m, 2H), 4.00 (dd, *J* = 7.9, 3.6 Hz, 0.5H), 3.93-3.80 (m, 1H), 3.73 (dd, *J* = 17.3, 3.6 Hz, 0.5H), 3.34-3.08 (m, 1H), 2.27-2.09 (m, 2H), 1.10 (dt, *J* = 19.9, 7.5 Hz, 3H); **^{13}C{^{1}H}NMR (101 MHz,**

CDCl$_3$): δ 200.6 (200.3), 167.9 (168.3), 156.4 (156.3), 136.76 (136.84), 134.28 (134.34), 131.37 (131.35), 131.0 (130.9), 130.0 (129.9), 128.7 (129.0), 128.0 (126.8), 127.5 (127.5), 125.71 (125.69), 86.7 (86.4), 74.1 (73.8), 51.2 (49.3), 51.2 (50.6), 37.7 (36.1), 30.6 (30.8), 13.9 (13.8), 12.47 (12.51); **HRMS (ESI, m/z)**: calcd. For $C_{22}H_{21}BrNO_2^+$ $[M+H]^+$: 410.0751, found: 410.0753.

Compound 4z (Fig. 3)

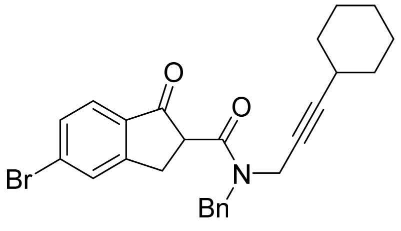

4z

***N*-benzyl-5-bromo-*N*-(3-cyclohexylprop-2-yn-1-yl)-1-oxo-2,3-dihydro-1*H*-indene-2-carboxamide (4z)**; **TLC**: R_f = 0.60 (petroleum ether /ethyl acetate = 5/1, v/v, UV); white solid (mp: 78-79°C.); yield: 65%; Enol isomerization were observed by NMR; **^{1}H NMR (400 MHz, CDCl$_3$)**: δ 7.70 (dd, *J* = 25.0, 1.5 Hz, 1H), 7.60 (t, *J* = 8.4 Hz, 1H), 7.56-7.49 (m, 1H), 7.44-7.18 (m, 5H), 5.31-5.16 (m, 1H), 4.92-4.79 (m, 1H), 4.41-4.26 (m, 2H), 4.01 (dd, *J* = 7.9, 3.7 Hz, 0.5H), 3.94-3.85 (ddd, *J* = 16.5, 14.2, 2.9 Hz, 1H), 3.78-3.69 (m, 0.5H), 3.35-3.10 (m, 1H), 2.48-2.28 (m, 1H), 1.87-1.62 (m, 4H), 1.59-1.22 (m,6H); **^{13}C{^{1}H}NMR (101 MHz, CDCl$_3$)**: δ 200.7 (200.4), 168.0 (168.3), 156.4 (156.3), 136.8 (137.0), 134.30 (134.34), 131.38 (131.36), 131.0 (130.9), 130.0 (129.9), 128.8 (129.0), 128.0 (127.5), 126.8 (127.7), 125.73 (125.70), 89.6 (89.4), 74.7 (74.3), 51.22 (51.24), 49.4 (50.5), 37.8 (36.2), 32.7 (32.6), 30.6 (30.8), 29.1 (29.1), 25.9 (26.0), 24.9; **HRMS (ESI, m/z)**: calcd. For $C_{26}H_{27}BrNO_2^+$ $[M+H]^+$: 464.1220, found: 464.1223.

1.4. General Procedure for the Synthesis of 1a-1g

1.4.1 General Procedure for the Synthesis of **1a**[6]

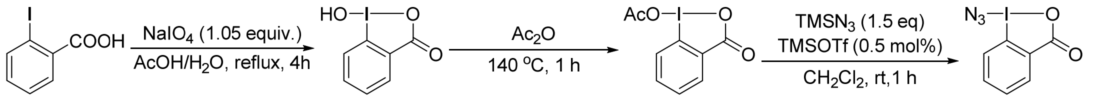

S12 S13 S14 1a

1) ***Caution: reaction carried out behind a safety shield!*** $NaIO_4$ (7.24 g, 33.8 mmol, 1.00 equiv) and 2-iodo benzoic acid (**S12**) (8.00 g, 32.2 mmol, 1.00 equiv) were suspended in 30% (v:v) aq AcOH (48 mL) under air. The mixture was vigorously stirred and refluxed for 4 h. the reaction mixture was then diluted with cold water (180 mL) and allowed to cool to room temperature, protecting it from light. The mixture is then filtered and further washed with ice water and cold acetone, air dried in the dark overnight to give the pure compound **S13** (8.14 g, 30.4 mmol, 94%) as a colorless solid.

2) Compound **S13** (3.00 g, 11.3 mmol, 1.00 equiv) was heated in Ac_2O (10 mL) to reflux until the solution turned clear (without suspension). The mixture was then left to cool down and white crystals started to form. The crystallization was continued at -18 °C. The crystal were then collected and dried overnight under high vacuum to give compound **S14** (3.06 g, 10.0 mmol, 86%).

3) ***Caution: reaction carried out behind a safety shield!*** **S14** (1.00 g, 3.28 mmol, 1.00 equiv) was stirred in dry DCM (3 mL) then $TMSN_3$ (0.66 mL, 4.9 mmol, 1.5 equiv) was cautiously added. A catalytic amount of TMSOTf (3 μL, 0.02 mmol, 0.005 equiv) was added last to the mixture which was then stirred for 30 minutes. The reaction mixture was then died in vacuo to give a yellow precipitate, which was washed a few times with hexanes to give compound **1a** (0.70 g, 2.4 mmol, 74%) as a pure pale yellow crystal.

1.4.2 General Procedure for the Synthesis of **1b**[6]

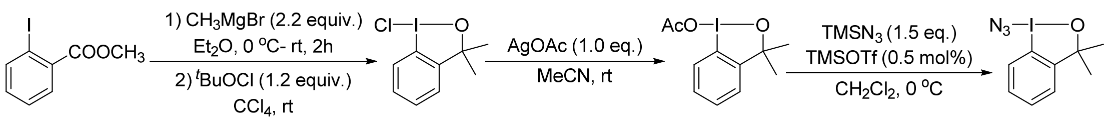

S15 S16 S17 1b

1) Methyl 2-iodobenzoate (**S15**) (12 mL, 76 mmol) was dissolved under N_2

atmosphere in dry diethyl ether (400 mL) and then the solution was cooled down at 0 °C with an ice bath. Methylmagnesium bromide (56.0 mL, 168 mmol, 2.20 equiv) was added dropwise and the reaction was stirred for 30 min at 0 °C. The reaction mixture was then allowed to warm to room temperature and it was further stirred for 2 h. The reaction was quenched with NH_4Cl in an iced bath. The organic layer was separated and extracted with Et_2O (3 x 100 mL), water (2 x 200 mL), brine (1 x 100 mL) and the combined organic layers were dried over $MgSO_4$. The solvent was removed in vacuum.

2) With no further purification the crude mixture was dissolved in CCl_4 (7 mL) and tert-butyl hypochlorite (10 mL, 92 mmol, 1.2 equiv) and the reaction mixture was stirred at room temperature. After one hour a yellow precipitate was collected by filtration and washed with hexane (60 mL) to afford compound **S16** (7.7 g, 26 mmol, 34% yield) as a yellow solid.

3) 1-Chloro-1,3-dihydro-3,3-dimethyl-1,2-benziodoxole (**S16**) (2.60 g, 8.77 mmol) was dissolved in dry acetonitrile (25 mL) under N_2 atmosphere. The reaction flask was covered with aluminum foils and protected from light. Silver acetate (1.46 g, 8.77 mmol, 1.00 equiv) was then added in one portion. The reaction mixture was stirred in the dark at room temperature for 16 h. Filtration over a Celite plug and evaporation of the solvent yielded compound **S17** (2.6 g, 8.8 mmol, 93%) as a light brownish solid.

4) ***Caution: This reaction should be carried out behind a safety shield!*** 1-Acetoxy-1,3-dihydro-3,3-dimethyl-1,2-benziodoxole (**S17**) (2.30 g, 7.18 mmol) was dissolved in dry CH_2Cl_2 (36 mL) under N_2 atmosphere. The reaction was placed in an iced bath and trimethylsilylazide (0.954 mL, 7.18 mmol, 1.00 equiv) was added via syringe, followed by TMSOTf (0.0065 mL, 0.036 mmol, 0.0050 equiv). The reaction was stirred for 15 min then the ice bath was removed and the stirring was continued for 1 h. The solvent was evaporated and the solid obtained was washed with n-hexane (2 x 30 mL) to afford **1b** as a yellow crystalline solid (2.10 g, 7.18 mmol, 96%).

1.4.3 General Procedure for the Synthesis of **1c-1g**[7]

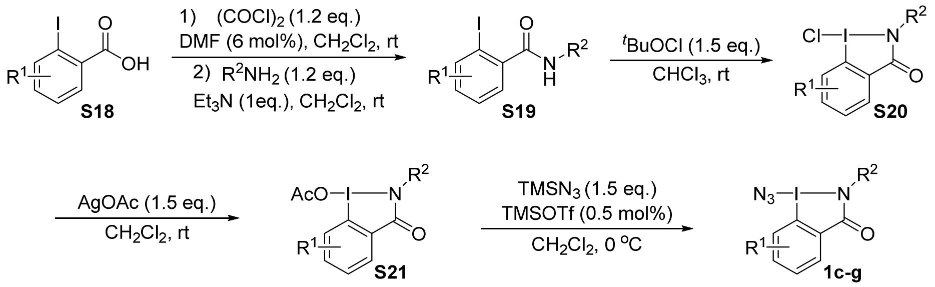

1) Under N_2, the *o*-iodobenzoic acid **S18** (20 mmol, 1.0 equiv.) and DMF (6 mol %) were dissolved in anhydrous CH_2Cl_2 (100 mL, 0.2 M), and $(COCl)_2$ (3.05g, 24 mmol, 1.2 equiv.) was dropwise added at 0 °C. The reaction mixture was then stirred at room temperature for 3 h. Upon completion, the solvent was removed under reduced pressure to afford *o*-iodobenzoyl chloride. To a 200 mL Shrek bottle equipped with a magnetic stir bar were sequentially added *o*-iodobenzoyl chloride, primary amine (24 mmol, 1.2 equiv.) and anhydrous CH_2Cl_2 (50 mL, 0.4 M). Thereafter, the mixture was cooled to 0 °C followed by addition of triethylamine (2.02 g, 20 mmol, 1.0 equiv.). The mixture was then heated with stirring at room temperature for 4 h. And then the mixture was washed with brine twice. The organic phase was dried over $MgSO_4$, filtered and the solvent was removed under reduced pressure. The crude mixture was purified by column chromatography (eluent: petroleum ether/EtOAc = 4/1) to give *o*-iodobenzamide **S19**.

2) To a solution of *o*-iodobenzamide **S19** (15 mmol, 1.0 equiv.) in $CHCl_3$ (40 mL, 0.375 M) was added ^{t}BuOCl (2.44 g, 22.5 mmol, 1.5 equiv.), the reaction mixture was stirred at room temperature for 30 min. Thereafter, the precipitate was filtered and crystallized from petroleum ether to give compound **S20**.

3) To a solution of **S20** (10 mmol, 1.0 equiv.) in DCM (80 mL, 0.125 M) was added AgOAc (2.0 g, 12 mmol, 1.2 equiv.) in dark, the reaction mixture was stirred at room temperature in dark overninght. Thereafter, the precipitate was filtered and crystallized from petroleum ether to give compound **S21**.

4) ***Caution: This reaction should be carried out behind a safety shield!*** Compound **S21** (4-9 mmol, 1.0 equiv.) was placed into an oven-dried Schlenk flask

equipped with a stirring bar under an N_2 atmosphere, and then freshly distilled DCM (0.2 M), $TMSN_3$ (1.5 equiv.), and TMSOTf (0.5 mol%) were added in that order. The reaction was stirred at 0 °C for 20 min. The precipitate was filtered off, washed with pentane and dried under vacuum to afford the corresponding product **1c-1g**

1.5. General Procedure for the Synthesis of Racemic Products 3 and 5

1.5.1 General Procedure for the synthesis of racemic **3a-3v** and **3w'-3x'**

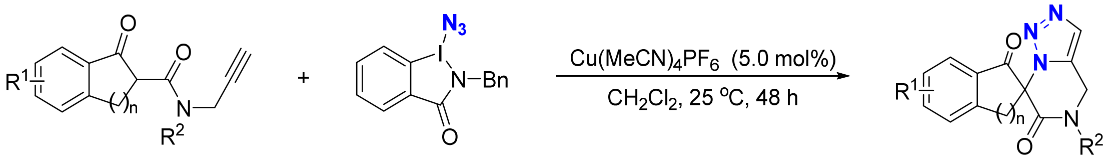

To a mixture of $Cu(MeCN)_4PF_6$ (0.005 mmol, 5 mol %), substrates **2a-2v** (0.10 mmol), **1d** (0.15 mmol) and dry dichloromethane (2 mL) under nitrogen atmosphere. The reaction system was stirred at 25 °C for 48 h, the substrates **2a-2v** was disappeared (monitored by TLC) completely. Finally, the crude product was purified by silica gel flash chromatography to afford the desired product *rac*-**3a** - *rac*-**3v**.

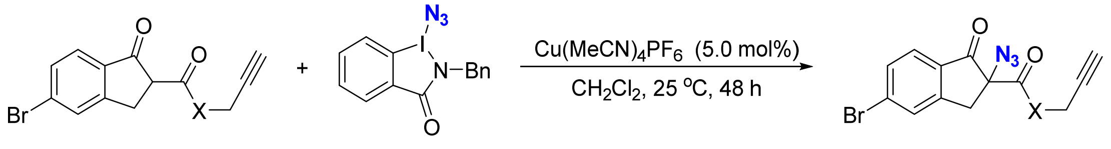

To a mixture of $Cu(MeCN)_4PF_6$ (0.005 mmol, 5 mol %), substrates **2w-2x** (0.10 mmol), **1d** (0.15 mmol) and dry dichloromethane (2 mL) under nitrogen atmosphere. The reaction system was stirred at 25 °C for 48 h, the substrates **2w-2x** was disappeared (monitored by TLC) completely. Finally, the crude product was purified by silica gel flash chromatography to afford the azidation product *rac*-**3w'** - *rac*-**3x'**.

1.5.2 General Procedure for the synthesis of racemic **5a-5z**

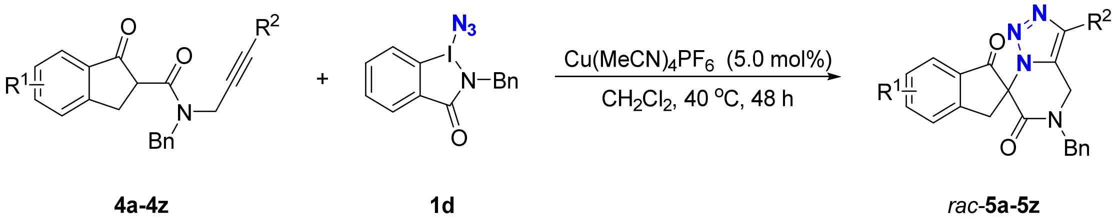

To a mixture of $Cu(MeCN)_4PF_6$ (0.005 mmol, 5 mol %), substrates **4a-4z** (0.10 mmol), **1d** (0.15 mmol) and dry dichloromethane (2 mL) under nitrogen atmosphere. The reaction system was stirred at 40 °C for 48 h, the substrates **4a-4z** was disappeared (monitored by TLC) completely. Finally, the crude product was purified by silica gel flash chromatography to afford the desired products *rac*-**5a** - *rac*-**5z**.

1.6. General Procedure for the Synthesis of Chiral Products 3 and 5

1.6.1 General Procedure for the synthesis of chiral **3a-3v** and **3w'-3x'**

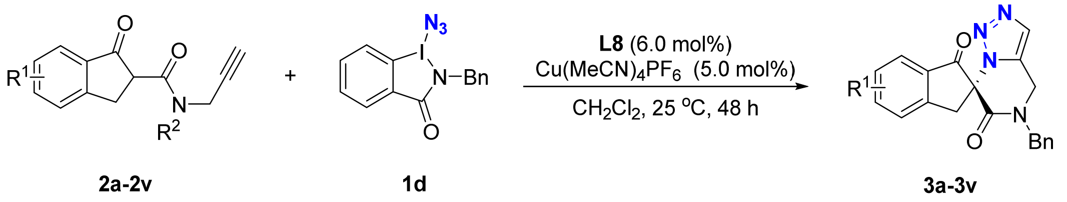

After stirring a mixture of $Cu(MeCN)_4PF_6$ (0.005 mmol, 5 mol %) and **L8** (0.006 mmol, 6 mol %) in dry dichloromethane (1 mL) at 25 °C under nitrogen atmosphere for 1.5 h, substrates **2a-2v** (0.10 mmol) and **1d** (0.15 mmol) in dry dichloromethane (1 mL) was added and the reaction mixture was stirred at 25 °C under nitrogen atmosphere. After 48 h, the substrates **2a-2v** was disappeared (monitored by TLC) completely. Finally, the crude product was purified by silica gel flash chromatography to afford the desired products **3a-3v**.

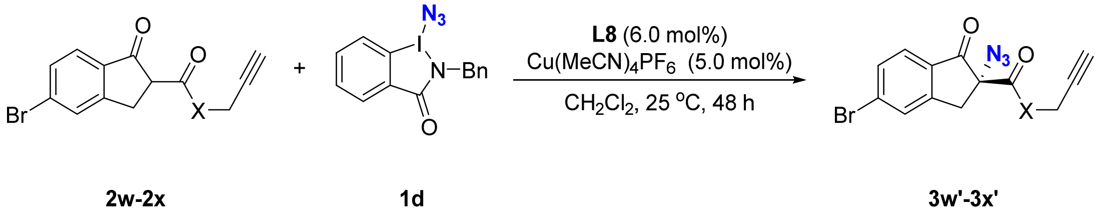

After stirring a mixture of $Cu(MeCN)_4PF_6$ (0.005 mmol, 5 mol %) and **L8** (0.006

mmol, 6 mol %) in dry dichloromethane (1 mL) at 25 °C under nitrogen atmosphere for 1.5 h, substrates **2w-2x** (0.10 mmol) and **1d** (0.15 mmol) in dry dichloromethane (1 mL) was added and the reaction mixture was stirred at 25 °C under nitrogen atmosphere. After 48 h, the substrates **2w-2x** was disappeared (monitored by TLC) completely. Finally, the crude product was purified by silica gel flash chromatography to afford the azidation products **3w**'-**3x**'.

1.6.2 General Procedure for the synthesis of chiral **5a-5z**

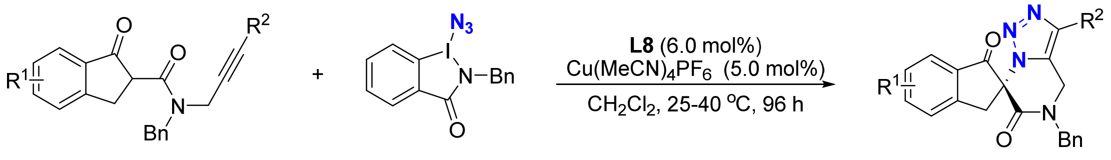

4a-4z **1d** **5a-5z**

After stirring a mixture of Cu(MeCN)4PF6 (0.005 mmol, 5 mol %) and **L8** (0.006 mmol, 6 mol %) in dry dichloromethane (1 mL) at 25 °C under nitrogen atmosphere for 1.5 h, substrates **4a-4z** (0.10 mmol) and **1d** (0.15 mmol) in dry dichloromethane (1 mL) was added and the reaction mixture was stirred at 25 °C under nitrogen atmosphere. After 48 h, when the substrates **4a-4z** was disappeared (monitored by TLC), the reaction mixture was stirred at 40 °C for more 48 h. Finally, the crude product was purified by silica gel flash chromatography to afford the desired product **5a-5z**.

Compound 3a (Fig. 2)

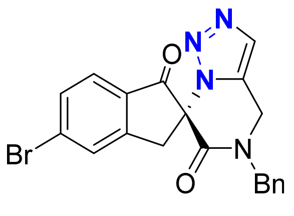

3a

(*R*)-5'-benzyl-5-bromo-4',5'-dihydro-6'*H*-spiro[indene-2,7'-[1,2,3]triazolo[1,5-*a*]pyrazine]-1,6'(3*H*)-dione (3a); **TLC**: R_f = 0.20 (petroleum ether /ethyl acetate = 3/1, v/v, UV); white solid (mp: 70-71 °C); yield: 99% (41.9 mg, 0.099 mmol); **$[\alpha]_D^{25}$** = 103

(c = 0.1, $CHCl_3$); **^{1}H NMR (400 MHz, $CDCl_3$)**: δ 7.84 (s, 1H), 7.68-7.51 (m, 3H), 7.43-7.29 (m, 3H), 7.26 (d, *J* = 8.0 Hz, 1H), 4.89 (dd, *J* = 16.2, 1.0 Hz, 1H), 4.82 (d, *J* = 14.8 Hz, 1H), 4.72 (d, *J* = 14.8 Hz, 1H), 4.58 (d, *J* = 16.2 Hz, 1H), 4.35 (d, *J* = 17.2 Hz, 1H), 4.27 (d, *J* = 17.2 Hz, 1H); **^{13}C{^{1}H}NMR (101 MHz, $CDCl_3$)**: δ 195.4, 162.8, 155.4, 134.7, 132.9, 132.4, 130.6, 130.0, 129.2, 128.6, 128.5, 128.2, 127.1, 72.0, 51.3, 41.8, 36.5; **HRMS (ESI, m/z)**: calcd. For $C_{20}H_{16}BrN_4O_2^+$ $[M+H]^+$: 423.0451, found: 423.0452; **HPLC**: Chiralpak AD-H Column, hexane/^{i}PrOH = 70/30, 1.0 mL/min, 254 nm, 30 ℃; RT = 14.7 min (major), 20.8 min (minor), ee: 94%.

Compound 3b (Fig. 2)

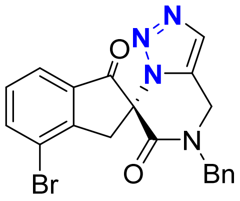

3b

(*R*)-5'-benzyl-4-bromo-4',5'-dihydro-6'*H*-spiro[indene-2,7'-[1,2,3]triazolo[1,5-*a*]pyrazine]-1,6'(3*H*)-dione (3b); **TLC**: R_f = 0.20 (petroleum ether /ethyl acetate = 3/1, v/v, UV); white solid (mp: 82-83 °C); yield: 90% (38.1 mg, 0.090 mmol); **$[\alpha]_D^{25}$** = 67 (c = 0.1, $CHCl_3$); **^{1}H NMR (400 MHz, $CDCl_3$)**: δ 7.92 (dd, *J* = 7.8, 1.0 Hz, 1H), 7.75 (dd, *J* = 7.6, 1.0 Hz, 1H), 7.62 (s, 1H), 7.42-7.31 (m, 4H), 7.27 (dd, *J* = 7.9, 1.7 Hz, 2H), 4.90 (dd, *J* = 16.2, 1.0 Hz, 1H), 4.84 (d, *J* = 14.8 Hz, 1H), 4.75 (d, *J* = 14.7 Hz, 1H), 4.60 (d, *J* = 16.6 Hz, 1H), 4.26 (s, 2H).; **^{13}C{^{1}H}NMR (101 MHz, $CDCl_3$)**: δ 196.0, 162.8, 153.8, 139.7, 134.7, 133.8, 130.3, 130.0, 129.3, 128.6, 128.5, 128.3, 124.9, 121.9, 71.7, 51.3, 41.9, 38.4; **HRMS (ESI, m/z)**: calcd. For $C_{20}H_{16}BrN_4O_2^+$ $[M+H]^+$: 423.0451, found: 423.0450; **HPLC**: Chiralpak OD-H Column, hexane/^{i}PrOH = 70/30, 1.0 mL/min, 254 nm, 30 ℃; RT = 58.2 min (major), 55.0 min (minor), ee: 90%.

Compound 3c (Fig. 2)

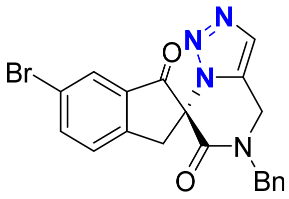

3c

(*R*)-5'-benzyl-6-bromo-4',5'-dihydro-6'*H*-spiro[indene-2,7'-[1,2,3]triazolo[1,5-*a*]pyrazine]-1,6'(3*H*)-dione (3c); **TLC**: R_f = 0.30 (petroleum ether /ethyl acetate = 2/1, v/v, UV); white solid (mp: 83-84 °C); yield: 95% (40.2 mg, 0.095 mmol); **[α]$_D^{25}$** = 70 (c = 0.1, $CHCl_3$); **^{1}H NMR (400 MHz, $CDCl_3$)**: δ 7.91 (d, *J* = 1.9 Hz, 1H), 7.85 (dd, *J* = 8.2, 1.9 Hz, 1H), 7.62 (s, 1H), 7.55 (d, *J* = 8.2 Hz, 1H), 7.41-7.32 (m, 3H), 7.29-7.20 (m, 2H), 4.90 (dd, *J* = 16.2, 1.0 Hz, 1H), 4.83 (d, *J* = 14.8 Hz, 1H), 4.73 (d, *J* = 14.8 Hz, 1H), 4.58 (d, *J* = 16.2 Hz, 1H), 4.33 (dd, *J* = 17.1, 1.0 Hz, 1H), 4.24 (d, *J* = 17.1 Hz, 1H).; **^{13}C{^{1}H}NMR (101 MHz, $CDCl_3$)**: δ 195.3, 162.7, 152.7, 139.8, 134.7, 133.6, 130.0, 129.3, 128.8, 128.6, 128.5, 128.2, 128.1, 122.7, 72.3, 51.3, 41.9, 36.6; **HRMS (ESI, m/z)**: calcd. For $C_{20}H_{16}BrN_4O_2^+$ $[M+H]^+$: 423.0451, found: 423.0449; **HPLC**: Chiralpak AD-H Column, hexane/^{i}PrOH = 70/30, 1.0 mL/min, 254 nm, 30 ℃; RT = 12.7 min (major), 21.7 min (minor), ee: 95%.

Compound 3d (Fig. 2)

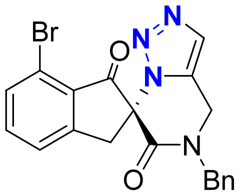

3d

(*R*)-5'-benzyl-7-bromo-4',5'-dihydro-6'*H*-spiro[indene-2,7'-[1,2,3]triazolo[1,5-*a*]pyrazine]-1,6'(3*H*)-dione (3d); **TLC**: R_f = 0.30 (petroleum ether /ethyl acetate = 2/1, v/v, UV); white solid (mp: 78-79 °C); yield: 90% (38.1 mg, 0.090 mmol); **[α]$_D^{25}$** = 103 (c = 0.1, $CHCl_3$); **^{1}H NMR (400 MHz, $CDCl_3$)**: δ 7.67-7.52 (m, 4H), 7.41-7.30 (m, 3H), 7.26 (dd, *J* = 5.5, 2.7 Hz, 2H), 4.95 (dd, *J* = 16.1, 1.1 Hz, 1H), 4.85 (d, *J* = 14.7 Hz, 1H), 4.70 (d, *J* = 14.8 Hz, 1H), 4.58 (d, *J* = 16.2 Hz, 1H), 4.40 (d, *J* = 17.1 Hz, 1H),

4.28 (d, J = 17.1 Hz, 1H); **$^{13}C\{^1H\}$NMR (101 MHz, $CDCl_3$)**: δ 194.2, 162.7, 156.9, 137.3, 134.7, 133.6, 130.1, 129.6, 129.2, 128.9, 128.5, 128.2, 125.5, 122.0, 72.7, 51.4, 41.8, 35.5; **HRMS (ESI, m/z)**: calcd. For $C_{20}H_{16}BrN_4O_2^+$ $[M+H]^+$: 423.0451, found: 423.0453; **HPLC**: Chiralpak AD-H Column, hexane/^{i}PrOH = 70/30, 1.0 mL/min, 254 nm, 30 ℃; RT = 14.3 min (major), 21.1 min (minor), ee: 91%.

Compound 3e (Fig. 2)

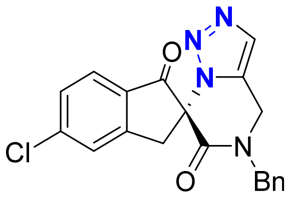

3e

(*R*)-5'-benzyl-5-chloro-4',5'-dihydro-6'*H*-spiro[indene-2,7'-[1,2,3]triazolo[1,5-*a*]pyrazine]-1,6'(3*H*)-dione (3e); **TLC**: R_f = 0.30 (petroleum ether /ethyl acetate = 2/1, v/v, UV); white solid (mp: 71-72 ºC); yield: 98% (37.1 mg, 0.098 mmol); **$[\alpha]_D^{25}$** = 75 (c = 0.1, $CHCl_3$); **^{1}H NMR (400 MHz, $CDCl_3$)**: δ 7.72 (d, J = 8.2 Hz, 1H), 7.64 (d, J = 18.6 Hz, 2H), 7.45 (dd, J = 8.3, 1.8 Hz, 1H), 7.40-7.30 (m, 3H), 7.26 (dd, J = 5.8, 2.2 Hz, 2H), 4.91 (dd, J = 16.2, 1.0 Hz, 1H), 4.83 (d, J = 14.8 Hz, 1H), 4.74 (d, J = 14.8 Hz, 1H), 4.58 (d, J = 16.2 Hz, 1H), 4.37 (d, J = 17.2 Hz, 1H), 4.28 (d, J = 17.2 Hz, 1H); **$^{13}C\{^1H\}$NMR (101 MHz, $CDCl_3$)**: δ 195.1, 162.8, 155.4, 143.9, 134.7, 130.2, 130.0, 129.6, 129.2, 128.6, 128.5, 128.2, 127.1, 126.9, 72.1, 51.3, 41.9, 36.6; **HRMS (ESI, m/z)**: calcd. For $C_{20}H_{16}ClN_4O_2^+$ $[M+H]^+$: 379.0956, found: 379.0957; **HPLC**: Chiralpak AD-H Column, hexane/^{i}PrOH = 70/30, 1.0 mL/min, 254 nm, 30 ℃; RT = 14.1 min (major), 21.2 min (minor), ee: 92%.

Compound 3f (Fig. 2)

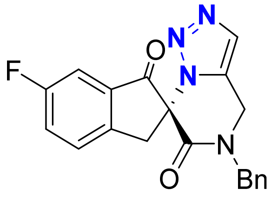

3f

(*R*)-5'-benzyl-6-fluoro-4',5'-dihydro-6'*H*-spiro[indene-2,7'-[1,2,3]triazolo[1,5-*a*]pyrazine]-1,6'(3*H*)-dione (3f); **TLC**: R_f = 0.30 (petroleum ether /ethyl acetate = 1/1, v/v, UV); white solid (mp: 68-69 °C); yield: 91% (33.0 mg, 0.091 mmol); **$[\alpha]_D^{25}$** = 88 (c = 0.1, $CHCl_3$); **^{1}H NMR (400 MHz, $CDCl_3$)**: δ 7.69-7.60 (m, 2H), 7.48 (td, *J* = 8.5, 2.6 Hz, 1H), 7.42 (dd, *J* = 7.2, 2.5 Hz, 1H), 7.40-7.32 (m, 3H), 7.26 (dd, *J* = 5.9, 2.2 Hz, 2H), 4.91 (dd, *J* = 16.2, 1.0 Hz, 1H), 4.83 (d, *J* = 14.8 Hz, 1H), 4.74 (d, *J* = 14.8 Hz, 1H), 4.59 (d, *J* = 16.1 Hz, 1H), 4.35 (d, *J* = 16.7 Hz, 1H), 4.26 (d, *J* = 16.8 Hz, 1H); **^{13}C{^{1}H}NMR (101 MHz, $CDCl_3$)**: δ 195.8 (d, *J* = 3.2 Hz), 162.84 (d, *J* = 250.0 Hz), 162.80, 149.8 (d, *J* = 2.2 Hz), 134.7, 133.5 (d, *J* = 8.0 Hz), 130.0, 129.3, 128.6, 128.5, 128.2, 128.1 (d, *J* = 8.0 Hz), 124.9 (d, *J* = 23.8 Hz), 111.7 (d, *J* = 22.5 Hz), 72.7, 51.3, 41.9, 36.5; **^{19}F NMR (376 MHz, Chloroform-*d*)**: δ -112.5; **HRMS (ESI, m/z)**: calcd. For $C_{20}H_{16}FN_4O_2^+$ $[M+H]^+$: 363.1252, found: 363.1250; **HPLC**: Chiralpak AD-H Column, hexane/^{i}PrOH = 70/30, 1.0 mL/min, 254 nm, 30 ℃; RT = 12.0 min (major), 18.8 min (minor), ee: 90%.

Compound 3g (Fig. 2)

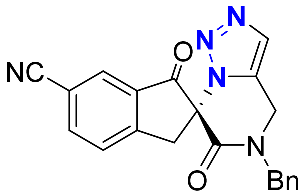

3g

(*R*)-5'-benzyl-1,6'-dioxo-1,3,5',6'-tetrahydro-4'*H*-spiro[indene-2,7'-[1,2,3]triazolo[1,5-*a*]pyrazine]-6-carbonitrile (3g); **TLC**: R_f = 0.20 (petroleum ether /ethyl acetate = 2/1, v/v, UV); white solid (mp: 92-93 °C); yield: 81% (30.0 mg, 0.081 mmol); **$[\alpha]_D^{25}$** = 127 (c = 0.1, $CHCl_3$); **^{1}H NMR (400 MHz, $CDCl_3$)**: δ 8.08 (d, *J* = 1.6 Hz, 1H), 7.99 (dd, *J* = 8.0, 1.6 Hz, 1H), 7.81 (dd, *J* = 8.1, 1.0 Hz, 1H), 7.63 (s, 1H), 7.46-7.32 (m, 3H), 7.32-7.22 (m, 2H), 4.91 (dd, *J* = 16.3, 0.9 Hz, 1H), 4.84 (d, *J* = 14.8 Hz, 1H), 4.73 (d, *J* = 14.8 Hz, 1H), 4.61 (d, *J* = 16.3 Hz, 1H), 4.45 (d, *J* = 17.7 Hz, 1H), 4.36 (d, *J* = 17.7 Hz, 1H); **^{13}C{^{1}H}NMR (101 MHz, $CDCl_3$)**: δ 194.9, 162.3, 157.8, 139.2, 134.5, 132.7, 130.1, 129.3, 128.6, 128.4, 128.2, 127.8, 126.5, 117.4, 113.1, 71.8,

51.4, 41.9, 37.3; **HRMS (ESI, m/z)**: calcd. For $C_{21}H_{16}BrN_5O_2^+$ [M+H]$^+$: 370.1299, found: 370.1297; **HPLC**: Chiralpak AD-H Column, hexane/^{i}PrOH = 60/40, 1.0 mL/min, 254 nm, 30 ℃; RT = 13.8 min (major), 27.2 min (minor), ee: 95%.

Compound 3h (Fig. 2)

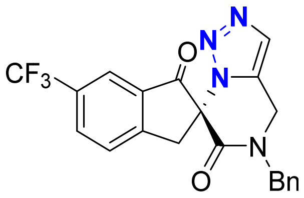

3h

(*R*)-5'-benzyl-6-(trifluoromethyl)-4',5'-dihydro-6'*H*-spiro[indene-2,7'-[1,2,3]triazolo[1,5-*a*]pyrazine]-1,6'(3*H*)-dione (3h); **TLC**: R_f = 0.30 (petroleum ether /ethyl acetate = 2/1, v/v, UV); white solid (mp: 60-61 °C); yield: 90% (37.2 mg, 0.090 mmol); **[α]$_D$^{25}** = 80 (c = 0.1, $CHCl_3$); **^{1}H NMR (400 MHz, $CDCl_3$)**: δ 8.06 (s, 1H), 8.00 (dd, *J* = 8.1, 1.8 Hz, 1H), 7.81 (d, *J* = 8.1 Hz, 1H), 7.63 (s, 1H), 7.47-7.32 (m, 3H), 7.32-7.23 (m, 2H), 4.92 (dd, *J* = 16.3, 1.0 Hz, 1H), 4.85 (d, *J* = 14.8 Hz, 1H), 4.73 (d, *J* = 14.8 Hz, 1H), 4.61 (d, *J* = 16.3 Hz, 1H), 4.45 (d, *J* = 17.4 Hz, 1H), 4.37 (d, *J* = 17.4 Hz, 1H); **^{13}C{^{1}H}NMR (101 MHz, $CDCl_3$)**: δ 195.6, 162.6, 157.2, 134.7, 133.4 (q, *J* = 3.4 Hz), 132.3, 131.6 (d, *J* = 33.5 Hz), 130.1, 129.3, 128.7, 128.6, 128.3, 127.4, 123.5 (q, *J* = 272.9 Hz), 123.3 (q, *J* = 4.0 Hz), 72.1, 51.4, 41.9, 37.1; **^{19}FNMR (376 MHz, Chloroform-*d*)**: δ -62.7; **HRMS (ESI, m/z)**: calcd. For $C_{21}H_{16}F_3N_4O_2^+$ [M+H]$^+$: 413.1220, found: 413.1218; **HPLC**: Chiralpak AD-H Column, hexane/^{i}PrOH = 70/30, 1.0 mL/min, 254 nm, 30 ℃; RT = 8.6 min (major), 12.9 min (minor), ee: 93%.

Compound 3i (Fig. 2)

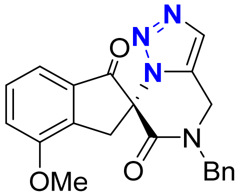

3i

(*R*)-5'-benzyl-4-methoxy-4',5'-dihydro-6'*H*-spiro[indene-2,7'-[1,2,3]triazolo[1,5-

***a*]pyrazine]-1,6'(3*H*)-dione (3i)**; **TLC**: R_f = 0.20 (petroleum ether /ethyl acetate = 2/1, v/v, UV); white solid (mp: 74-75 ºC); yield: 95% (35.6 mg, 0.095 mmol); **$[\alpha]_D^{25}$** = 95 (c = 0.1, $CHCl_3$); **^{1}H NMR (400 MHz, $CDCl_3$)**: δ 7.68 (t, *J* = 7.9 Hz, 1H), 7.59 (s, 1H), 7.41-7.29 (m, 3H), 7.31-7.23 (m, 2H), 7.22-7.15 (m, 1H), 6.85 (d, *J* = 8.3 Hz, 1H), 4.95 (dd, *J* = 15.9, 1.0 Hz, 1H), 4.81 (d, *J* = 14.9 Hz, 1H), 4.74 (d, *J* = 14.9 Hz, 1H), 4.54 (d, *J* = 15.9 Hz, 1H), 4.38 (d, *J* = 17.2 Hz, 1H), 4.27 (d, *J* = 17.1 Hz, 1H), 3.93 (s, 3H); **^{13}C{^{1}H}NMR (101 MHz, $CDCl_3$)**: δ 196.8, 163.2, 156.8, 143.3, 134.9, 133.1, 130.2, 129.9, 129.2, 128.6, 128.4, 128.3, 117.3, 117.1, 72.0, 55.8, 51.2, 41.9, 34.2; **HRMS (ESI, m/z)**: calcd. For $C_{21}H_{19}N_4O_3^+$ $[M+H]^+$: 375.1452, found: 375.1450; **HPLC**: Chiralpak OD-H Column, hexane/^{i}PrOH = 50/50, 1.0 mL/min, 254 nm, 30 ℃; RT = 27.7 min (major), 25.7 min (minor), ee: 95%.

Compound 3j (Fig. 2)

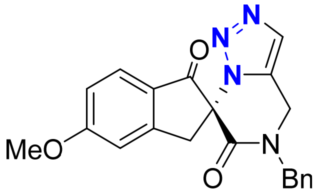

3j

(*R*)-5'-benzyl-5-methoxy-4',5'-dihydro-6'*H*-spiro[indene-2,7'-[1,2,3]triazolo[1,5-*a*]pyrazine]-1,6'(3*H*)-dione (3j); **TLC**: R_f = 0.20 (petroleum ether /ethyl acetate = 2/1, v/v, UV); white solid (mp: 81-82 ºC); yield: 91% (34.1 mg, 0.091 mmol); **$[\alpha]_D^{25}$** = 85 (c = 0.1, $CHCl_3$); **^{1}H NMR (400 MHz, $CDCl_3$)**: δ 7.70 (d, *J* = 8.6 Hz, 1H), 7.60 (s, 1H), 7.42-7.21 (m, 5H), 7.07 (d, *J* = 2.2 Hz, 1H), 6.98 (dd, *J* = 8.6, 2.2 Hz, 1H), 4.93 (d, *J* = 15.9 Hz, 1H), 4.83 (d, *J* = 14.8 Hz, 1H), 4.75 (d, *J* = 14.8 Hz, 1H), 4.56 (d, *J* = 16.0 Hz, 1H), 4.36 (d, *J* = 17.0 Hz, 1H), 4.26 (d, *J* = 17.1 Hz, 1H), 3.95 (s, 3H); **^{13}C{^{1}H}NMR (101 MHz, $CDCl_3$)**: δ 194.3, 167.2, 163.5, 157.4, 134.9, 130.0, 129.2, 128.8, 128.4, 128.2, 127.9, 124.6, 117.3, 109.5, 72.6, 56.1, 51.3, 41.9, 36.5; **HRMS (ESI, m/z)**: calcd. For $C_{21}H_{19}N_4O_3^+$ $[M+H]^+$: 375.1452, found: 375.1452; **HPLC**: Chiralpak AD-H Column, hexane/^{i}PrOH = 70/30, 1.0 mL/min, 254 nm, 30 ℃; RT = 14.7 min (major), 21.0 min (minor), ee: 85%.

Compound 3k (Fig. 2)

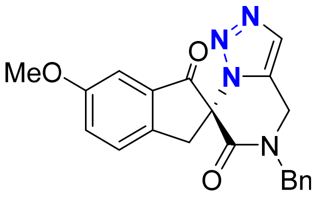

3k

(*R*)-5'-benzyl-6-methoxy-4',5'-dihydro-6'*H*-spiro[indene-2,7'-[1,2,3]triazolo[1,5-*a*]pyrazine]-1,6'(3*H*)-dione (3k); **TLC**: R_f = 0.20 (petroleum ether /ethyl acetate = 2/1, v/v, UV); white solid (mp: 73-74 °C); yield: 95% (35.6 mg, 0.095 mmol); **$[\alpha]_D^{25}$** = 69 (c = 0.1, $CHCl_3$); **^{1}H NMR (400 MHz, $CDCl_3$)**: δ 7.61 (s,1H), 7.54 (d, *J* = 8.5 Hz, 1H), 7.42-7.24 (m, 6H), 7.18 (d, *J* = 2.5 Hz, 1H), 4.91 (dd, *J* = 16.1, 1.0 Hz, 1H), 4.85 (d, *J* = 14.8 Hz, 1H), 4.73 (d, *J* = 14.8 Hz, 1H), 4.58 (d, *J* = 16.2 Hz, 1H), 4.30 (d, *J* = 16.7 Hz, 1H), 4.21 (d, *J* = 16.7 Hz, 1H), 3.84 (s, 3H); **^{13}C{^{1}H}NMR (101 MHz, $CDCl_3$)**: δ 196.5, 163.2, 160.3, 147.4, 134.9, 132.9, 129.9, 129.2, 128.6, 128.5, 128.3, 127.3, 126.7, 106.8, 72.8, 55.9, 51.3, 41.9, 36.4; **HRMS (ESI, m/z)**: calcd. For $C_{21}H_{19}N_4O_3^+$ [M+H]$^+$: 375.1452, found: 375.1450; **HPLC**: Chiralpak AD-H Column, hexane/^{i}PrOH = 70/30, 1.0 mL/min, 254 nm, 30 ℃; RT = 15.5 min (major), 20.8 min (minor), ee: 89%.

Compound 3l (Fig. 2)

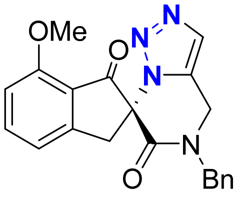

3l

(*R*)-5'-benzyl-7-methoxy-4',5'-dihydro-6'*H*-spiro[indene-2,7'-[1,2,3]triazolo[1,5-*a*]pyrazine]-1,6'(3*H*)-dione (3l); **TLC**: R_f = 0.20 (petroleum ether /ethyl acetate = 2/1, v/v, UV); white solid (mp: 85-86 °C); yield: 75% (28.1mg, 0.075 mmol); **$[\alpha]_D^{25}$** = 75 (c = 0.1, $CHCl_3$); **^{1}H NMR (400 MHz, $CDCl_3$)**: δ 7.60 (s, 1H), 7.43 (t, *J* = 7.8 Hz, 1H), 7.40-7.32 (m, 4H), 7.30-7.24 (m, 2H), 7.17 (dd, *J* = 7.9, 1.0 Hz, 1H), 4.91 (dd, *J* = 16.1, 1.0 Hz, 1H), 4.83 (d, *J* = 14.8 Hz, 1H), 4.75 (d, *J* = 14.8 Hz, 1H), 4.58 (dd, *J* = 16.1, 0.7 Hz, 1H), 4.23 (s, 2H), 3.96 (s, 3H); **^{13}C{^{1}H}NMR (101 MHz, $CDCl_3$)**: δ 193.8,

163.4, 159.8, 156.1, 139.1, 134.9, 130.0, 129.2, 129.0, 128.3, 128.2, 120.0, 118.3, 110.0, 72.7, 56.0, 51.3, 41.9, 35.7; **HRMS (ESI, m/z)**: calcd. For $C_{21}H_{19}N_4O_3^+$ $[M+H]^+$: 375.1452, found: 375.1451; **HPLC**: Chiralpak OD-H Column, hexane/^{i}PrOH = 50/50, 1.0 mL/min, 254 nm, 30 ℃; RT = 44.4 min (major), 26.6 min (minor), ee: 93%.

Compound 3m (Fig. 2)

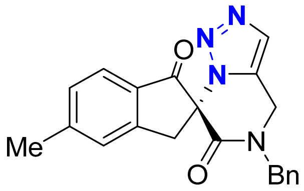

3m

(*R*)-5'-benzyl-5-methyl-4',5'-dihydro-6'*H*-spiro[indene-2,7'-[1,2,3]triazolo[1,5-*a*]pyrazine]-1,6'(3*H*)-dione (3m); **TLC**: R_f = 0.30 (petroleum ether /ethyl acetate = 2/1, v/v, UV); white solid (mp: 66-67 ºC); yield: 84% (30.2 mg, 0.084 mmol); $[\alpha]_D^{25}$ = 70 (c = 0.1, $CHCl_3$); **^{1}H NMR (400 MHz, $CDCl_3$)**: δ 7.67 (d, J = 7.9 Hz, 1H), 7.59 (s, 1H), 7.45 (s, 1H), 7.40-7.31 (m, 3H), 7.27 (dd, J = 6.5, 1.9 Hz, 3H), 4.92 (dd, J = 16.1, 1.0 Hz, 1H), 4.82 (d, J = 14.8 Hz, 1H), 4.74 (d, J = 14.8 Hz, 1H), 4.56 (d, J = 16.1 Hz, 1H), 4.35 (d, J = 17.0 Hz, 1H), 4.25 (d, J = 17.0 Hz, 1H), 2.50 (s, 3H); **^{13}C{^{1}H}NMR (101 MHz, $CDCl_3$)**: δ 195.8, 163.3, 154.6, 148.9, 134.9, 130.0, 129.9, 129.4, 129.2, 128.7, 128.4, 128.2, 127.0, 126.0, 72.5, 51.2, 41.9, 36.6, 22.5; **HRMS (ESI, m/z)**: calcd. For $C_{21}H_{19}N_4O_2^+$ $[M+H]^+$: 359.1503, found: 359.1503; **HPLC**: Chiralpak AD-H Column, hexane/^{i}PrOH = 70/30, 1.0 mL/min, 254 nm, 30 ℃; RT = 12.3 min (major), 16.7 min (minor), ee: 90%.

Compound 3n (Fig. 2)

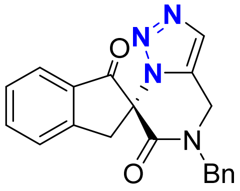

3n

(*R*)-5'-benzyl-4',5'-dihydro-6'*H*-spiro[indene-2,7'-[1,2,3]triazolo[1,5-*a*]pyrazine]-

1,6'(3*H*)-dione (3n); **TLC**: R_f = 0.20 (petroleum ether /ethyl acetate = 2/1, v/v, UV); white solid (mp: 67-68 °C); yield: 93% (32.1 mg, 0.093 mmol); **$[\alpha]_D^{25}$** = 74 (c = 0.1, $CHCl_3$); **^{1}H NMR (400 MHz, $CDCl_3$)**: δ 7.80-7.75 (m, 2H), 7.66 (dt, *J* = 7.8, 1.0 Hz, 1H), 7.61 (s, 1H), 7.48 (t, *J* = 8.0, 1H), 7.41-7.32 (m, 3H), 7.31-7.24 (m, 2H), 4.93 (dd, *J* = 16.2, 1.0 Hz, 1H), 4.83 (d, *J* = 14.8 Hz, 1H), 4.75 (d, *J* = 14.8 Hz, 1H), 4.58 (d, *J* = 16.1 Hz, 1H), 4.41 (d, *J* = 17.0 Hz, 1H), 4.32 (d, *J* = 17.0 Hz, 1H); **^{13}C{^{1}H}NMR (101 MHz, $CDCl_3$)**: δ 196.6, 163.2, 154.2, 137.1, 134.9, 131.8, 130.0, 129.2, 128.7, 128.7, 128.5, 128.2, 126.6, 126.2, 72.2, 51.3, 41.9, 36.8; **HRMS (ESI, m/z)**: calcd. For $C_{20}H_{17}N_4O_2^+$ $[M+H]^+$: 345.1346, found: 345.1346; **HPLC**: Chiralpak AD-H Column, hexane/^{i}PrOH = 70/30, 1.0 mL/min, 254 nm, 30 ℃; RT = 13.0 min (major), 15.1 min (minor), ee: 93%.

Compound 3o (Fig. 2)

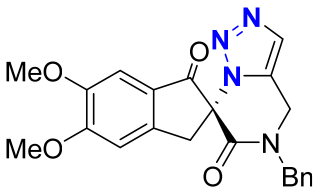

3o

(*R*)-5'-benzyl-5,6-dimethoxy-4',5'-dihydro-6'*H*-spiro[indene-2,7'-[1,2,3]triazolo[1,5-*a*]pyrazine]-1,6'(3*H*)-dione (3o); **TLC**: R_f = 0.20 (petroleum ether /ethyl acetate = 2/1, v/v, UV); white solid (mp: 170-171 °C); yield: 80% (32.4 mg, 0.080 mmol); **$[\alpha]_D^{25}$** = 46 (c = 0.1, $CHCl_3$); **^{1}H NMR (400 MHz, $CDCl_3$)**: δ 7.60 (s, 1H), 7.41-7.30 (m, 3H), 7.31-7.24 (m, 2H), 7.14 (s, 1H), 7.06 (s, 1H), 4.93 (d, *J* = 16.0 Hz, 1H), 4.86 (d, *J* = 14.8 Hz, 1H), 4.72 (d, *J* = 14.8 Hz, 1H), 4.56 (d, *J* = 16.1 Hz, 1H), 4.30 (d, *J* = 16.7 Hz, 1H), 4.21 (d, *J* = 16.8 Hz, 1H), 4.03 (s, 3H), 3.90 (s, 3H); **^{13}C{^{1}H}NMR (101 MHz, $CDCl_3$)**: δ 194.7, 163.5, 157.6, 150.5, 150.4, 135.0, 129.9, 129.2, 128.7, 128.4, 128.2, 124.3, 107.5, 105.8, 72.7, 56.7, 56.3, 51.3, 41.9, 36.5; **HRMS (ESI, m/z)**: calcd. For $C_{22}H_{21}N_4O_2^+$ $[M+H]^+$: 405.1557, found: 405.1555; **HPLC**: Chiralpak AD-H Column, hexane/^{i}PrOH = 60/40, 1.0 mL/min, 254 nm, 30 ℃; RT = 14.1 min (major), 24.1 min (minor), ee: 81%.

Compound 3p (Fig. 2)

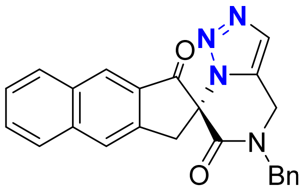

3p

(*R*)-5'-benzyl-4',5'-dihydro-6'*H*-spiro[cyclopenta[*b*]naphthalene-2,7'-[1,2,3]triazolo[1,5-*a*]pyrazine]-1,6'(3*H*)-dione (3p); **TLC**: $R_f = 0.20$ (petroleum ether /ethyl acetate = 2/1, v/v, UV); white solid (mp: 85-86 °C); yield: 93% (36.7mg, 0.093 mmol); **$[\alpha]_D^{25}$** = 66 (c = 0.1, $CHCl_3$); **^{1}H NMR (400 MHz, $CDCl_3$)**: δ 8.41 (s, 1H), 8.06 (s, 1H), 7.99 (d, *J* = 8.3 Hz, 1H), 7.93 (d, *J* = 8.8 Hz, 1H), 7.70-7.61 (m, 2H), 7.54 (td, *J* = 7.5, 6.8, 1.3 Hz, 1H), 7.42-7.31 (m, 3H), 7.31-7.27 (m, 2H), 4.95 (dd, *J* = 16.1, 1.0 Hz, 1H), 4.84 (d, *J* = 14.8 Hz, 1H), 4.76 (d, *J* = 14.8 Hz, 1H), 4.61-4.58 (m, 2H), 4.49 (dd, *J* = 16.9, 1.3 Hz, 1H).; **^{13}C{^{1}H}NMR (101 MHz, $CDCl_3$)**: δ 196.8, 163.4, 145.4, 138.4, 134.9, 132.9, 130.7, 130.1, 130.0, 129.5, 129.3, 128.8, 128.5, 128.3, 128.2, 128.1, 126.8, 124.9, 72.9, 51.3, 41.9, 36.5; **HRMS (ESI, m/z)**: calcd. For $C_{24}H_{19}N_4O_2^+$ $[M+H]^+$: 395.1503, found: 395.1501; **HPLC**: Chiralpak AD-H Column, hexane/^{i}PrOH = 70/30, 1.0 mL/min, 254 nm, 30 ℃; RT = 20.6 min (major), 29.2 min (minor), ee: 91%.

Compound 3q (Fig. 2)

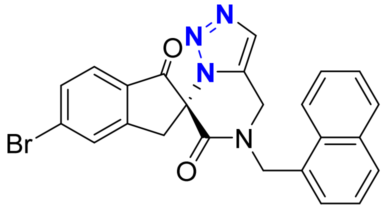

3q

(*R*)-5-bromo-5'-(naphthalen-1-ylmethyl)-4',5'-dihydro-6'*H*-spiro[indene-2,7'-[1,2,3]triazolo[1,5-*a*]pyrazine]-1,6'(3*H*)-dione (3q); **TLC**: $R_f = 0.20$ (petroleum ether /ethyl acetate = 3/1, v/v, UV); white solid (mp: 93-94 °C); yield: 89% (42.1 mg, 0.089 mmol); **$[\alpha]_D^{25}$** = 101 (c = 0.1, $CHCl_3$); **^{1}H NMR (400 MHz, $CDCl_3$)**: δ 8.01-7.93 (m, 1H), 7.94-7.85 (m, 3H), 7.70-7.60 (m, 2H), 7.58-7.40 (m, 5H), 5.34 (d, *J* = 14.9 Hz,

1H), 5.20 (d, J = 14.9 Hz, 1H), 4.77 (d, J = 16.3 Hz, 1H), 4.52 (d, J = 16.3 Hz, 1H), 4.37 (d, J = 17.1 Hz, 1H), 4.31 (d, J = 17.1 Hz, 1H); **^{13}C{^{1}H}NMR (101 MHz, CDCl$_3$)**: δ 195.2, 162.6, 155.4, 134.1, 133.0, 132.5, 131.6, 130.8, 130.1, 130.0, 130.0, 129.7, 129.1, 128.6, 127.8, 127.2, 127.1, 126.6, 125.4, 123.3, 72.1, 49.3, 41.5, 36.7; **HRMS (ESI, m/z)**: calcd. For $C_{24}H_{18}BrN_4O_2^+$ [M+H]$^+$: 473.0608, found: 473.0606; **HPLC**: Chiralpak AS-H Column, hexane/^{i}PrOH = 60/40, 1.0 mL/min, 254 nm, 30 ℃; RT = 23.7 min (major), 19.7 min (minor), ee: 94%.

Compound 3r (Fig. 2)

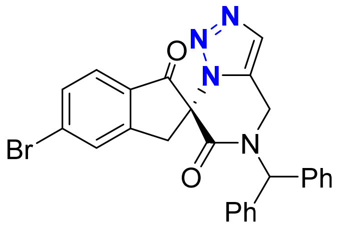

3r

(*R*)-5'-benzhydryl-5-bromo-4',5'-dihydro-6'*H*-spiro[indene-2,7'-[1,2,3]triazolo[1,5-*a*]pyrazine]-1,6'(3*H*)-dione (3r); **TLC**: R_f = 0.20 (petroleum ether /ethyl acetate = 3/1, v/v, UV); white solid (mp: 156-157 °C); yield: 98% (48.9mg, 0.098 mmol); **[α]$_D$^{25}** = 33 (c = 0.1, CHCl$_3$); **^{1}H NMR (400 MHz, CDCl$_3$)**: δ 7.85 (s, 1H), 7.64-7.59 (m, 3H), 7.45-7.32 (m, 6H), 7.26 (d, J = 7.0 Hz, 2H), 7.20-7.11 (m, 3H), 4.70 (dd, J = 16.3, 1.0 Hz, 1H), 4.55-4.39 (m, 2H), 4.31 (d, J = 17.2 Hz, 1H); **^{13}C{^{1}H}NMR (101 MHz, CDCl$_3$)**: δ 195.5, 163.1, 155.5, 136.9, 133.0, 132.4, 130.6, 130.4, 130.0, 129.1, 128.9, 128.6, 128.4, 127.2, 72.5, 61.9, 39.4, 36.2; **HRMS (ESI, m/z)**: calcd. For $C_{26}H_{20}BrN_4O_2^+$ [M+H]$^+$: 499.0764, found: 499.0763; **HPLC**: Chiralpak AD-H Column, hexane/^{i}PrOH = 70/30, 1.0 mL/min, 254 nm, 30 ℃; RT = 14.0 min (major), 26.7 min (minor), ee: 88%.

Compound 3s (Fig. 2)

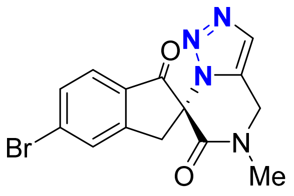

3s

(*R*)-5-bromo-5'-methyl-4',5'-dihydro-6'*H*-spiro[indene-2,7'-[1,2,3]triazolo[1,5-*a*]pyrazine]-1,6'(3*H*)-dione (3s); **TLC**: R_f = 0.20 (petroleum ether /ethyl acetate = 3/1, v/v, UV); white solid (mp: 229-230 °C); yield: 80% (27.8 mg, 0.080 mmol); $[\alpha]_D^{25}$ = 110 (c = 0.1, $CHCl_3$); **^{1}H NMR (400 MHz, $CDCl_3$)**: δ 7.83 (t, *J* = 1.1 Hz, 1H), 7.69 (s, 1H), 7.60 (d, *J* = 0.8 Hz, 1H), 5.06 (dd, *J* = 16.1, 1.0 Hz, 1H), 4.69 (dd, *J* = 16.1, 0.7 Hz, 1H), 4.32 (dd, *J* = 17.1, 1.1 Hz, 1H), 4.20 (dd, *J* = 17.0, 1.0 Hz, 1H), 3.18 (s, 3H); **$^{13}C\{^1H\}$NMR (101 MHz, $CDCl_3$)**: δ 195.4, 162.6, 155.5, 132.9, 132.4, 130.7, 130.0, 129.9, 128.5, 127.1, 72.0, 44.4, 36.4, 35.7; **HRMS (ESI, m/z)**: calcd. For $C_{14}H_{12}BrN_4O_2^+$ $[M+H]^+$: 347.0138, found: 347.0140; **HPLC**: Chiralpak AD-H Column, hexane/^{i}PrOH = 90/10, 1.0 mL/min, 254 nm, 30 ℃; RT = 42.7 min (major), 46.9 min (minor), ee: 95%.

Compound 3t (Fig. 2)

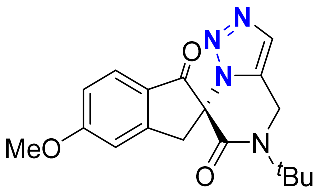

3t

(*R*)-5'-(*tert*-butyl)-5-methoxy-4',5'-dihydro-6'*H*-spiro[indene-2,7'-[1,2,3]triazolo[1,5-*a*]pyrazine]-1,6'(3*H*)-dione (3t); **TLC**: R_f = 0.20 (petroleum ether /ethyl acetate = 3/1, v/v, UV); white solid (mp: 70-71 °C); yield: 80% (27.3 mg, 0.080 mmol); $[\alpha]_D^{25}$ = 90 (c = 0.1, $CHCl_3$); **^{1}H NMR (400 MHz, $CDCl_3$)**: δ 7.67 (s, 1H), 7.66 (d, *J* = 8.6 Hz, 1H), 7.04 (d, *J* = 2.2 Hz, 1H), 6.95 (dd, *J* = 8.6, 2.2 Hz, 1H), 4.97 (dd, *J* = 15.7, 1.1 Hz, 1H), 4.84 (d, *J* = 15.7 Hz, 1H), 4.30 (d, *J* = 17.0 Hz, 1H), 4.16 (d, *J* = 17.0 Hz, 1H), 3.92 (s, 3H); **$^{13}C\{^1H\}$NMR (101 MHz, $CDCl_3$)**: δ 194.9, 167.1, 163.8, 157.6, 130.1, 129.7, 127.7, 124.8, 117.1, 109.4, 73.3, 59.9, 56.0, 39.1, 36.3, 27.9; **HRMS (ESI, m/z)**: calcd. For $C_{18}H_{21}N_4O_3^+$ $[M+H]^+$: 341.1608, found: 341.1606; **HPLC**: Chiralpak AD-H Column, hexane/^{i}PrOH = 70/30, 1.0 mL/min, 254 nm, 30 ℃; RT = 7.5 min (major), 9.9 min (minor), ee: 78%.

Compound 3u (Fig. 2)

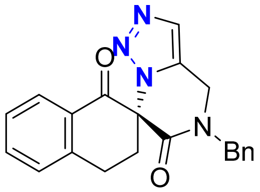

3u

(*R*)-5'-benzyl-3,4,4',5'-tetrahydro-1*H*,6'*H*-spiro[naphthalene-2,7'-[1,2,3]triazolo[1,5-*a*]pyrazine]-1,6'-dione (3u); **TLC**: R*f* = 0.40 (petroleum ether /ethyl acetate = 3/1, v/v, UV); colorless oil; yield: 39% (14.0 mg, 0.039 mmol); **[α]$_D^{25}$** = 25 (c = 0.1, $CHCl_3$); **^{1}H NMR (400 MHz, $CDCl_3$)**: δ 8.00 (dd, *J* = 7.8, 1.4 Hz, 1H), 7.61 – 7.57 (m, 2H), 7.38-7.30 (m, 5H), 7.27 – 7.24 (m, 2H), 4.80-4.76 (m, 3H), 4.54 (d, *J* = 16.2 Hz, 1H), 3.79 (ddd, *J* = 16.9, 8.9, 5.5 Hz, 1H), 3.53 (dt, *J* = 16.9, 5.8 Hz, 1H), 3.34 (ddd, *J* = 14.3, 8.9, 5.5 Hz, 1H), 3.15 (dt, *J* = 14.2, 5.9 Hz, 1H); **^{13}C{^{1}H}NMR (101 MHz, $CDCl_3$)**: δ 189.7, 164.6, 145.0, 135.2, 135.0, 130.0, 129.2, 129.2, 129.1, 129.0, 128.4, 128.2, 128.1, 127.2, 69.3, 51.0, 41.8, 31.6, 26.3; **HRMS (ESI, m/z)**: calcd. For $C_{21}H_{19}N_4O_2^+$ $[M+H]^+$: 359.1053, found: 359.1045; **HPLC**: Chiralpak AD-H Column, hexane/^{i}PrOH = 70/30, 1.0 mL/min, 254 nm, 30 ℃; RT = 10.6 min (major), 16.1 min (minor), ee: 44%.

Compound 3v (Fig. 2)

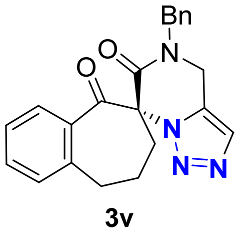

3v

(*R*)-5'-benzyl-4',5',8,9-tetrahydro-6'*H*-spiro[benzo[7]annulene-6,7'-[1,2,3]triazolo[1,5-*a*]pyrazine]-5,6'(7*H*)-dione (3v); **TLC**: R*f* = 0.40 (petroleum ether /ethyl acetate = 3/1, v/v, UV); colorless oil; yield: 43% (16.0 mg, 0.043 mmol); **[α]$_D^{25}$** = 2 (c = 0.1, $CHCl_3$); **^{1}H NMR (400 MHz, $CDCl_3$)**: δ 7.58 (s, 1H), 7.48 (td, *J* = 7.3, 2.0 Hz, 1H), 7.35-7.29 (m, 5H), 7.28 – 7.20 (m, 3H), 4.77 (d, *J* = 14.7 Hz, 1H), 4.65 (d, *J* = 15.2 Hz, 2H), 4.50 (d, *J* = 16.1 Hz, 1H), 3.51 (ddd, *J* = 15.6, 9.6, 6.5 Hz, 1H), 3.14 – 2.97 (m, 2H), 2.57 (dt, *J* = 15.2, 4.8 Hz, 1H), 2.37 – 2.12 (m, 2H); **^{13}C{^{1}H}NMR (101 MHz, $CDCl_3$)**: δ 201.2, 164.5, 139.0, 137.8, 135.0, 132.7, 129.2, 129.2, 128.9, 128.8,

128.7, 128.4, 128.2, 127.0, 74.0, 51.1, 41.6, 32.2, 31.9, 22.3; **HRMS (ESI, m/z)**: calcd. For $C_{22}H_{21}N_4O_2^+$ $[M+H]^+$: 373.1659, found: 373.1665; **HPLC**: Chiralpak AD-H Column, hexane/^{i}PrOH = 70/30, 1.0 mL/min, 254 nm, 30 ℃; RT = 12.9 min (major), 9.4 min (minor), ee: 10%.

Compound 3w' (Fig. 2)

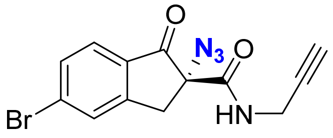

3w'

(*R*)-2-azido-5-bromo-1-oxo-*N*-(prop-2-yn-1-yl)-2,3-dihydro-1*H*-indene-2-carboxamide (3w'); **TLC**: R_f = 0.70 (petroleum ether /ethyl acetate = 3/1, v/v, UV); white solid (mp: 80-81°C); yield: 55% (18.3 mg, 0.055 mmol); $[\alpha]_D^{25}$ = 10 (c = 0.1, $CHCl_3$); **^{1}H NMR (400 MHz, $CDCl_3$)**: δ 7.70 (d, J = 1.6 Hz, 1H), 7.66 (d, J = 8.2 Hz, 1H), 7.59 (dd, J = 8.3, 1.6 Hz, 1H), 6.95 (s, 1H), 4.14 (ddd, J = 17.6, 5.8, 2.6 Hz, 1H), 4.05 – 3.93 (m, 2H), 3.22 (d, J = 17.6 Hz, 1H), 2.28 (t, J = 2.6 Hz, 1H); **^{13}C{^{1}H}NMR (101 MHz, $CDCl_3$)**: δ 196.7, 165.8, 153.8, 132.7, 132.4, 132.2, 130.0, 126.7, 78.5, 72.7, 72.55, 37.4, 30.0; **HRMS (ESI, m/z)**: calcd. For $C_{13}H_{10}BrN_4O_2^+$ $[M+H]^+$: 332.9982, found: 332.9990; **HPLC**: Chiralpak AD-H Column, hexane/^{i}PrOH = 90/10, 1.0 mL/min, 254 nm, 30 ℃; RT = 15.7 min (major), 18.2 min (minor), ee: 74%.

Compound 3x' (Fig. 2)

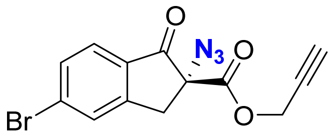

3x'

prop-2-yn-1-yl (*R*)-2-azido-5-bromo-1-oxo-2,3-dihydro-1*H*-indene-2-carboxylate (3x'); **TLC**: R_f = 0.70 (petroleum ether /ethyl acetate = 3/1, v/v, UV); white solid (mp: 99-100°C); yield: 76% (25.4 mg, 0.076 mmol); $[\alpha]_D^{25}$ = 96 (c = 0.1, $CHCl_3$); **^{1}H NMR (400 MHz, $CDCl_3$)**: δ 7.73 – 7.66 (m, 2H), 7.61 (dd, J = 8.2, 1.6 Hz, 1H), 4.86 (dd, J = 15.5, 2.5 Hz, 1H), 4.71 (dd, J = 15.5, 2.5 Hz, 1H), 3.68 (d, J = 17.6 Hz, 1H), 3.03 (d,

J = 17.6 Hz, 1H), 2.51 (t, J = 2.5 Hz, 1H); **$^{13}C\{^1H\}$NMR (101 MHz, $CDCl_3$)**: δ 195.8, 167.5, 153.5, 132.4, 132.4, 131.8, 130.0, 126.9, 76.4, 76.3, 70.1, 54.2, 38.1; **HRMS (ESI, m/z)**: calcd. For $C_{13}H_9BrN_3O_3^+$ $[M+H]^+$: 333.9822, found: 333.9812; **HPLC**: Chiralpak AD-H Column, hexane/^{i}PrOH = 95/5, 1.0 mL/min, 254 nm, 30 ℃; RT = 17.0 min (major), 15.7 min (minor), ee: 52%.

Compound 5a (Fig. 3)

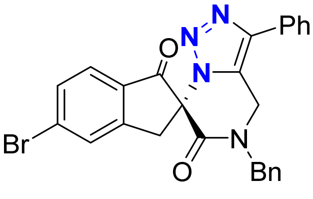

5a

(*R*)-5'-benzyl-5-bromo-3'-phenyl-4',5'-dihydro-6'*H*-spiro[indene-2,7'-[1,2,3]triazolo[1,5-*a*]pyrazine]-1,6'(3*H*)-dione (5a); **TLC**: R_f = 0.50 (petroleum ether /ethyl acetate = 4/1, v/v, UV); white solid (mp: 86-87 ºC); yield: 75% (37.4 mg, 0.075 mmol); **$[\alpha]_D^{25}$** = 165 (c = 0.1, $CHCl_3$); **^1H NMR (400 MHz, $CDCl_3$)**: δ 7.86 (s, 1H), 7.69-7.59 (m, 4H), 7.44 (t, J = 7.5 Hz, 2H), 7.36 (tt, J = 7.0, 5.9 Hz, 4H), 7.32-7.25 (m, 2H), 5.12 (d, J = 16.2 Hz, 1H), 4.96 (d, J = 14.9 Hz, 1H), 4.69 (t, J = 15.9 Hz, 2H), 4.43 (d, J = 17.2 Hz, 1H), 4.30 (d, J = 17.2 Hz, 1H); **$^{13}C\{^1H\}$NMR (101 MHz, $CDCl_3$)**: δ 195.4, 162.8, 155.5, 142.9, 134.8, 133.0, 132.4, 130.7, 130.1, 130.0, 129.3, 129.2, 128.5, 128.5, 128.0, 127.2, 126.5, 124.8, 72.2, 51.5, 43.2, 36.4; **HRMS (ESI, m/z)**: calcd. For $C_{26}H_{20}BrN_4O_2^+$ $[M+H]^+$: 499.0764, found: 499.0763; **HPLC**: Chiralpak AD-H Column, hexane/^{i}PrOH = 70/30, 1.0 mL/min, 254 nm, 30 ℃; RT = 25.8 min (major), 18.3 min (minor), ee: 93%.

Compound 5b (Fig. 3)

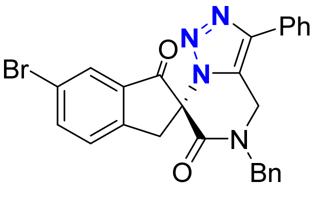

5b

(*R*)-5'-benzyl-6-bromo-3'-phenyl-4',5'-dihydro-6'*H*-spiro[indene-2,7'-[1,2,3]triazolo[1,5-*a*]pyrazine]-1,6'(3*H*)-dione (5b); **TLC**: R_f = 0.50 (petroleum ether

/ethyl acetate = 4/1, v/v, UV); white solid (mp: 190-191 °C); yield: 64% (31.9 mg, 0.064 mmol); **[α]D[25]** = 78 (c = 0.1, $CHCl_3$); **^{1}H NMR (400 MHz, $CDCl_3$)**: δ 7.93 (d, *J* = 1.9 Hz, 1H), 7.86 (dd, *J* = 8.2, 2.0 Hz, 1H), 7.64-7.62 (m, 2H), 7.56 (d, *J* = 8.2 Hz, 1H), 7.47-7.44 (m, 2H), 7.41-7.32 (m, 4H), 7.29-7.27 (m, 2H), 5.12 (d, *J* = 16.2 Hz, 1H), 4.97 (d, *J* = 14.9 Hz, 1H), 4.71 (d, *J* = 16.2 Hz, 1H), 4.67 (d, *J* = 14.9 Hz, 1H), 4.39 (d, *J* = 17.3 Hz, 1H), 4.27 (d, *J* = 17.2 Hz, 1H); **^{13}C{^{1}H}NMR (101 MHz, $CDCl_3$)**: δ 195.3, 162.8, 152.8, 142.9, 139.8, 134.7, 133.6, 130.1, 129.3, 129.2, 128.8, 128.5, 128.5, 128.1, 128.0, 126.5, 124.8, 122.7, 72.4, 51.5, 43.2, 36.5; **HRMS (ESI, m/z)**: calcd. For $C_{26}H_{20}BrN_4O_2^+$ $[M+H]^+$: 499.0764, found: 499.0762; **HPLC**: Chiralpak AD-H Column, hexane/^{i}PrOH = 70/30, 1.0 mL/min, 254 nm, 30 ℃; RT = 28.1 min (major), 19.2 min (minor), ee: 93%.

Compound 5c (Fig. 3)

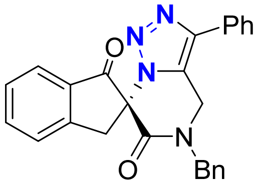

5c

(*R*)-5'-benzyl-3'-phenyl-4',5'-dihydro-6'*H*-spiro[indene-2,7'-[1,2,3]triazolo[1,5-*a*]pyrazine]-1,6'(3*H*)-dione (5c); **TLC**: R_f = 0.50 (petroleum ether /ethyl acetate = 4/1, v/v, UV); white solid (mp: 89-90 °C); yield: 70% (29.5 mg, 0.070 mmol); **[α]D[25]** = 190 (c = 0.1, $CHCl_3$); **^{1}H NMR (400 MHz, $CDCl_3$)**: δ 7.82 (d, *J* = 7.7 Hz, 1H), 7.77 (td, *J* = 7.5, 1.2 Hz, 1H), 7.68 (d, *J* = 7.8 Hz, 1H), 7.65-7.63 (m, 2H), 7.53-7.42 (m, 3H), 7.39-7.25 (m, 4H), 7.30 (d, *J* = 6.5 Hz, 2H), 5.15 (d, *J* = 16.1 Hz, 1H), 4.97 (d, *J* = 14.9 Hz, 1H), 4.70 (dd, *J* = 15.5, 7.4 Hz, 2H), 4.48 (d, *J* = 17.0 Hz, 1H), 4.35 (d, *J* = 17.0 Hz, 1H); **^{13}C{^{1}H}NMR (101 MHz, $CDCl_3$)**: δ 196.5, 163.2, 154.3, 142.8, 137.1, 134.9, 131.8, 130.3, 129.3, 129.2, 128.7, 128.4, 128.4, 128.0, 126.7, 126.5, 126.2, 124.9, 72.4, 51.5, 43.3, 36.7; **HRMS (ESI, m/z)**: calcd. For $C_{26}H_{21}N_4O_2^+$ $[M+H]^+$: 421.1659, found: 421.1660; **HPLC**: Chiralpak AD-H Column, hexane/^{i}PrOH = 70/30, 1.0 mL/min, 254 nm, 30 ℃; RT = 18.6 min (major), 13.3 min (minor), ee: 94%.

Compound 5d (Fig. 3)

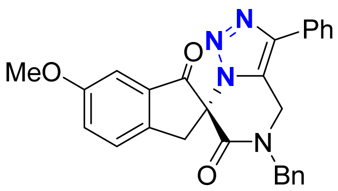

5d

(*R*)-5'-benzyl-6-methoxy-3'-phenyl-4',5'-dihydro-6'*H*-spiro[indene-2,7'-[1,2,3]triazolo[1,5-*a*]pyrazine]-1,6'(3*H*)-dione (5d); **TLC**: R_f = 0.40 (petroleum ether /ethyl acetate = 4/1, v/v, UV); white solid (mp: 219-220 °C); yield: 67% (30.2 mg, 0.067 mmol); **$[\alpha]_D^{25}$** = 120 (c = 0.1, $CHCl_3$); **^{1}H NMR (400 MHz, $CDCl_3$)**: δ 7.65-7.63 (m, 2H), 7.55 (d, *J* = 8.4 Hz, 1H), 7.46-7.43 (m, 2H), 7.41-7.32 (m, 5H), 7.30-7.29 (m, 2H), 7.20 (d, *J* = 2.6 Hz, 1H), 5.13 (d, *J* = 16.1 Hz, 1H), 4.98 (d, *J* = 14.9 Hz, 1H), 4.71 (d, *J* = 16.1 Hz, 1H), 4.67 (d, *J* = 14.9 Hz, 1H), 4.37 (d, *J* = 16.6 Hz, 1H), 4.24 (d, *J* = 16.7 Hz, 1H), 3.85 (s, 3H); **^{13}C{^{1}H}NMR (101 MHz, $CDCl_3$)**: δ 196.5, 163.2, 160.3, 147.5, 142.7, 134.9, 133.0, 130.3, 129.3, 129.1, 128.40, 128.38, 128.0, 127.3, 126.7, 126.5, 124.8, 106.8, 73.0, 55.9, 51.4, 43.3, 36.3; **HRMS (ESI, m/z)**: calcd. For $C_{27}H_{23}N_4O_3^+$ $[M+H]^+$: 451.1765, found: 451.1763; **HPLC**: Chiralpak AD-H Column, hexane/^{i}PrOH = 70/30, 1.0 mL/min, 254 nm, 30 ℃; RT = 27.7 min (major), 18.6 min (minor), ee: 88%.

Compound 5e (Fig. 3)

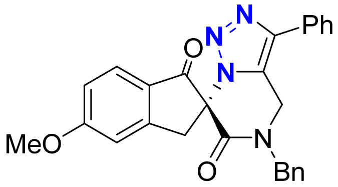

5e

(*R*)-5'-benzyl-5-methoxy-3'-phenyl-4',5'-dihydro-6'*H*-spiro[indene-2,7'-[1,2,3]triazolo[1,5-*a*]pyrazine]-1,6'(3*H*)-dione (5e); **TLC**: R_f = 0.20 (petroleum ether /ethyl acetate = 3/1, v/v, UV); white solid (mp: 120-121 °C); yield: 62% (28.0 mg, 0.062 mmol); **$[\alpha]_D^{25}$** = 105 (c = 0.1, $CHCl_3$); **^{1}H NMR (400 MHz, $CDCl_3$)**: δ 7.72 (d, *J* = 8.6 Hz, 1H), 7.64-7.62 (m, 2H), 7.44-7.42 (m, 2H), 7.39-7.27 (m, 6H), 7.09 (d, *J* = 2.2 Hz, 1H), 6.99 (dd, *J* = 8.6, 2.2 Hz, 1H), 5.16 (d, *J* = 16.0 Hz, 1H), 4.97 (d, *J* = 15.0 Hz,10H), 4.70 (d, *J* = 5.1 Hz, 1H), 4.66 (d, *J* = 4.0 Hz, 1H), 4.43 (d, *J* = 17.0 Hz, 1H), 4.29 (d, *J*

= 17.1 Hz, 1H), 3.95 (s, 3H); **$^{13}C\{^1H\}$NMR (101 MHz, $CDCl_3$)**: δ 194.3, 167.3, 163.3, 157.5, 142.8, 135.0, 130.3, 129.2, 129.1, 128.4, 128.3, 128.0, 127.9, 126.5, 125.0, 124.7, 117.3, 109.6, 72.8, 56.1, 51.5, 43.3, 36.4; **HRMS (ESI, m/z)**: calcd. For $C_{27}H_{23}N_4O_3^+$ $[M+H]^+$: 451.1765, found: 451.1768; **HPLC**: Chiralpak AD-H Column, hexane/^{i}PrOH = 70/30, 1.0 mL/min, 254 nm, 30 ℃; RT = 20.1 min (major), 18.8 min (minor), ee: 83%.

Compound 5f (Fig. 3)

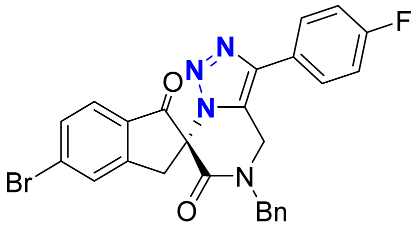

5f

(*R*)-5'-benzyl-5-bromo-3'-(4-fluorophenyl)-4',5'-dihydro-6'*H*-spiro[indene-2,7'-[1,2,3]triazolo[1,5-*a*]pyrazine]-1,6'(3*H*)-dione (5f); **TLC**: R_f = 0.50 (petroleum ether /ethyl acetate = 4/1, v/v, UV); white solid (mp: 198-199 °C); yield: 63% (32.6 mg, 0.063 mmol); **$[\alpha]_D^{25}$** = 75 (c = 0.1, $CHCl_3$); **^{1}H NMR (400 MHz, $CDCl_3$)**: δ 7.86 (d, *J* = 1.5 Hz, 1H), 7.70-7.52 (m, 4H), 7.44-7.31 (m, 3H), 7.29-7.27 (m, 2H), 7.16-7.12 (m, 2H), 5.10 (d, *J* = 16.1 Hz, 1H), 4.96 (d, *J* = 14.9 Hz, 1H), 4.67 (dd, *J* = 15.5, 3.6 Hz, 2H), 4.43 (d, *J* = 17.2 Hz, 1H), 4.31 (d, *J* = 17.2 Hz, 1H); **$^{13}C\{^1H\}$NMR (101 MHz, $CDCl_3$)**: δ 195.4, 162.81, 162.80 (d, *J* = 249.7 Hz), 155.5, 142.1, 134.7, 133.1, 132.5, 130.6, 130.1, 129.3, 128.5, 128.3 (d, *J* = 8.3 Hz), 128.0, 127.2, 126.4 (d, *J* = 3.3 Hz), 124.6, 116.3 (d, *J* = 21.8 Hz), 72.2, 51.5, 43.1, 36.3; **^{19}FNMR (376 MHz, Chloroform-*d*)**: δ -112.6; **HRMS (ESI, m/z)**: calcd. For $C_{26}H_{19}BrFN_4O_2^+$ $[M+H]^+$: 517.0670, found: 517.0671; **HPLC**: Chiralpak AD-H Column, hexane/^{i}PrOH = 70/30, 1.0 mL/min, 254 nm, 30 ℃; RT = 19.7 min (major), 17.3 min (minor), ee: 94%.

Compound 5g (Fig. 3)

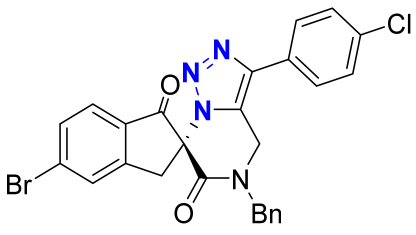

5g

(*R*)-5'-benzyl-5-bromo-3'-(4-chlorophenyl)-4',5'-dihydro-6'*H*-spiro[indene-2,7'-[1,2,3]triazolo[1,5-*a*]pyrazine]-1,6'(3*H*)-dione (5g); **TLC**: R_f = 0.50 (petroleum ether /ethyl acetate = 4/1, v/v, UV); white solid (mp: 178-179 °C); yield: 74% (39.4 mg, 0.074 mmol); **$[\alpha]_D^{25}$** = 65 (c = 0.1, $CHCl_3$); **^{1}H NMR (400 MHz, $CDCl_3$)**: δ 7.86 (d, *J* = 1.5 Hz, 1H), 7.69-7.59 (m, 2H), 7.57-7.54 (m, 2H), 7.44-7.31 (m, 5H), 7.29-7.27 (m, 2H), 5.10 (d, *J* = 16.2 Hz, 1H), 4.96 (d, *J* = 14.9 Hz, 1H), 4.67 (d, *J* = 16.2 Hz, 2H), 4.41 (d, *J* = 17.2 Hz, 1H), 4.30 (d, *J* = 17.2 Hz, 1H); **^{13}C{^{1}H}NMR (101 MHz, $CDCl_3$)**: δ 195.3, 162.7, 155.5, 141.9, 134.7, 134.4, 133.1, 132.5, 130.6, 130.0, 129.4, 129.3, 128.6, 128.5, 128.0, 127.7, 127.2, 124.9, 72.2, 51.5, 43.1, 36.3; **HRMS (ESI, m/z)**: calcd. For $C_{26}H_{19}BrClN_4O_2^+$ $[M+H]^+$: 533.0374, found: 533.0377; **HPLC**: Chiralpak AD-H Column, hexane/^{i}PrOH = 70/30, 1.0 mL/min, 254 nm, 30 ℃; RT = 23.8 min (major), 19.0 min (minor), ee: 94%.

Compound 5h (Fig. 3)

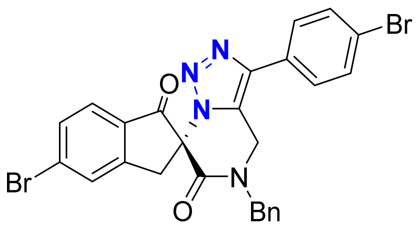

5h

(*R*)-5'-benzyl-5-bromo-3'-(4-bromophenyl)-4',5'-dihydro-6'*H*-spiro[indene-2,7'-[1,2,3]triazolo[1,5-*a*]pyrazine]-1,6'(3*H*)-dione (5h); **TLC**: R_f = 0.50 (petroleum ether /ethyl acetate = 4/1, v/v, UV); white solid (mp: 70-71 °C); yield: 71% (41.0mg, 0.071 mmol); **$[\alpha]_D^{25}$** = 105 (c = 0.1, $CHCl_3$); **^{1}H NMR (400 MHz, $CDCl_3$)**: δ 7.86 (d, *J* = 1.5 Hz, 1H), 7.69-7.59 (m, 2H), 7.57-7.55 (m, 2H), 7.50-7.47 (m, 2H), 7.43-7.31 (m, 4H), 7.29-7.27 (m, 2H), 5.09 (d, *J* = 16.2 Hz, 1H), 4.95 (d, *J* = 14.9 Hz, 1H), 4.67 (dd, *J* =

15.5, 4.0 Hz, 2H), 4.41 (d, J = 17.2 Hz, 1H), 4.30 (d, J = 17.2 Hz, 1H); **$^{13}C\{^1H\}$NMR (101 MHz, $CDCl_3$)**: δ 195.3, 162.7, 155.5, 141.9, 134.7, 133.1, 132.5, 132.3, 130.6, 130.1, 129.3, 128.5, 128.3, 128.0, 127.9, 127.2, 125.0, 122., 72.2, 51.5, 43.1, 36.3; **HRMS (ESI, m/z)**: calcd. For $C_{26}H_{19}Br_2N_4O_2^+$ $[M+H]^+$: 576.9869, found: 576.9870; **HPLC**: Chiralpak AD-H Column, hexane/^{i}PrOH = 70/30, 1.0 mL/min, 254 nm, 30 ℃; RT = 26.0 min (major), 20.2 min (minor), ee: 94%.

Compound 5i (Fig. 3)

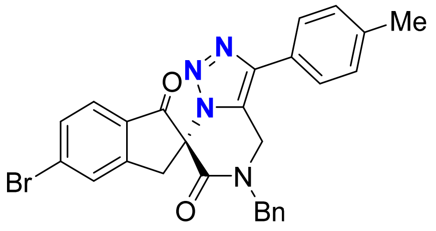

5i

(*R*)-5'-benzyl-5-bromo-3'-(p-tolyl)-4',5'-dihydro-6'*H*-spiro[indene-2,7'-[1,2,3]triazolo[1,5-*a*]pyrazine]-1,6'(3*H*)-dione (5i); **TLC**: R_f = 0.50 (petroleum ether /ethyl acetate = 4/1, v/v, UV); white solid (mp: 86-87 ºC); yield: 54% (26.2 mg, 0.054 mmol); **$[\alpha]_D^{25}$** = 150 (c = 0.1, $CHCl_3$); **^1H NMR (400 MHz, $CDCl_3$)**: δ 7.86 (d, J = 1.4 Hz, 1H), 7.72-7.59 (m, 2H), 7.52-7.50 (m, 2H), 7.40-7.36 (m, 3H), 7.32-7.22 (m, 4H), 5.11 (d, J = 16.2 Hz, 1H), 4.94 (d, J = 14.9 Hz, 1H), 4.69 (d, J = 16.3 Hz, 2H), 4.44 (d, J = 17.1 Hz, 1H), 4.30 (d, J = 17.2 Hz, 1H), 2.38 (s, 3H); **$^{13}C\{^1H\}$NMR (101 MHz, $CDCl_3$)**: δ 195.5, 162.9, 155.6, 143.0, 138.5, 134.8, 133.0, 132.4, 130.7, 130.1, 129.9, 129.3, 128.5, 128.0, 127.3, 127.2, 126.4, 124.4, 72.2, 51.5, 43.2, 36.4, 21.4; **HRMS (ESI, m/z)**: calcd. For $C_{27}H_{22}BrN_4O_2^+$ $[M+H]^+$: 513.0921, found: 513.0920; **HPLC**: Chiralpak AD-H Column, hexane/^{i}PrOH = 70/30, 1.0 mL/min, 254 nm, 30 ℃; RT = 26.3 min (major), 19.6 min (minor), ee: 94%.

Compound 5j (Fig. 3)

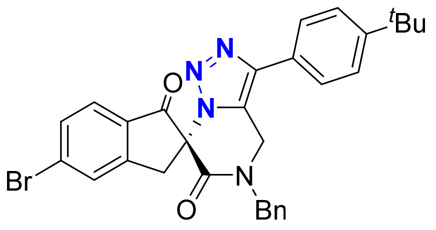

5j

(*R*)-5'-benzyl-5-bromo-3'-(4-(tert-butyl)phenyl)-4',5'-dihydro-6'*H*-spiro[indene-2,7'-[1,2,3]triazolo[1,5-*a*]pyrazine]-1,6'(3*H*)-dione (5j); **TLC**: R_f = 0.50 (petroleum ether /ethyl acetate = 4/1, v/v, UV); white solid (mp: 199-200 ºC); yield: 60% (33.3 mg, 0.060 mmol); **[α]$_D$^{25}** = 124 (c = 0.1, $CHCl_3$); **^{1}H NMR (400 MHz, $CDCl_3$)**: δ 7.86 (s, 1H), 7.71-7.59 (m, 2H), 7.58-7.56 (m, 2H), 7.48-7.46 (m, 2H), 7.41-7.26 (m, 5H), 5.12 (d, *J* = 16.1 Hz, 1H), 4.95 (d, *J* = 15.0 Hz, 1H), 4.69 (dd, *J* = 17.0, 15.5 Hz, 2H), 4.44 (d, *J* = 17.2 Hz, 1H), 4.31 (d, *J* = 17.2 Hz, 1H), 1.34 (s, 9H); **^{13}C{^{1}H}NMR (101 MHz, $CDCl_3$)**: δ 195.4, 162.9, 155.6, 151.6, 142.9, 134.8, 132.9, 132.4, 130.7, 130.0, 129.3, 128.4, 128.0, 127.3, 127.1, 126.2, 126.1, 124.5, 72.2, 51.5, 43.3, 36.4, 34.8, 31.4; **HRMS (ESI, m/z)**: calcd. For $C_{30}H_{28}BrN_4O_2^+$ $[M+H]^+$: 555.1390, found: 555.1394; **HPLC**: Chiralpak AD-H Column, hexane/^{i}PrOH = 70/30, 1.0 mL/min, 254 nm, 30 ℃; RT = 16.5 min (major), 15.5 min (minor), ee: 93%.

Compound 5k (Fig. 3)

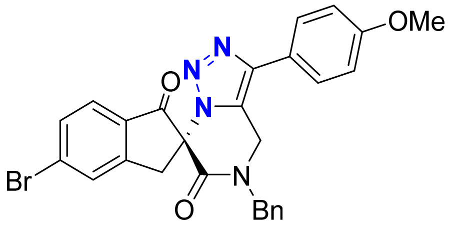

5k

(*R*)-5'-benzyl-5-bromo-3'-(4-methoxyphenyl)-4',5'-dihydro-6'*H*-spiro[indene-2,7'-[1,2,3]triazolo[1,5-*a*]pyrazine]-1,6'(3*H*)-dione; **TLC**: R_f = 0.50 (petroleum ether /ethyl acetate = 4/1, v/v, UV); white solid (mp: 70-71 ºC); yield: 50% (26.5 mg, 0.050 mmol); **[α]$_D$^{25}** = 99 (c = 0.1, $CHCl_3$); **^{1}H NMR (400 MHz, $CDCl_3$)**: δ 7.86 (s, 1H), 7.68-7.60 (m, 2H), 7.55 (d, *J* = 8.7 Hz, 2H), 7.44-7.28 (m, 5H), 6.97 (d, *J* = 8.8 Hz, 2H), 5.09 (d, *J* = 16.1 Hz, 1H), 4.95 (d, *J* = 14.9 Hz, 1H), 4.67 (dd, *J* = 15.5, 4.0 Hz, 2H), 4.43 (d,

J = 17.2 Hz, H), 4.30 (d, J = 17.2 Hz, 1H), 3.84 (s, 3H); **$^{13}C\{^1H\}$NMR (101 MHz, $CDCl_3$)**: δ 195.5, 162.9, 159.8, 155.6, 142.8, 134.8, 133.0, 132.4, 130.7, 130.1, 129.3, 128.5, 128.0, 127.9, 127.2, 123.9, 122.7, 114.6, 72.2, 55.5, 51.5, 43.2, 36.4; **HRMS (ESI, m/z)**: calcd. For $C_{27}H_{22}BrN_4O_2^+$ $[M+H]^+$: 529.0870, found: 529.0868; **HPLC**: Chiralpak AS-H Column, hexane/^{i}PrOH = 60/40, 1.0 mL/min, 254 nm, 30 ℃; RT = 25.4 min (major), 20.1 min (minor), ee: 94%.

Compound 5l (Fig. 3)

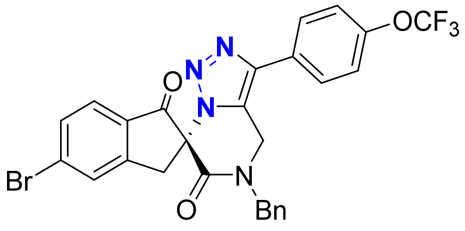

5l

(*R*)-5'-benzyl-5-bromo-3'-(4-(trifluoromethoxy)phenyl)-4',5'-dihydro-6'*H*-spiro[indene-2,7'-[1,2,3]triazolo[1,5-*a*]pyrazine]-1,6'(3*H*)-dione; **TLC**: R_f = 0.40 (petroleum ether /ethyl acetate = 4/1, v/v, UV); white solid (mp: 190-191 ºC); yield: 75% (43.7 mg, 0.075 mmol); **$[\alpha]_D^{25}$** = 136 (c = 0.1, $CHCl_3$); **^{1}H NMR (400 MHz, $CDCl_3$)**: δ 7.87 (s, 1H), 7.68-7.62 (m, 4H), 7.43-7.27 (m, 7H), 5.11 (d, J = 16.2 Hz, 1H), 4.98 (d, J = 14.9 Hz, 1H), 4.68 (dd, J = 15.5, 4.2 Hz, 2H), 4.44 (d, J = 17.2 Hz, 1H), 4.32 (d, J = 17.2 Hz, 1H); **$^{13}C\{^1H\}$NMR (101 MHz, $CDCl_3$)**: δ 195.3, 162., 155.5, 149.2, 141.7, 134.7, 133.1, 132.5, 130.6, 130.1, 129.4, 128.9, 128.6, 128.0, 127.9, 127.2, 125.1, 121.7, 120.6 (q, J = 257.9 Hz), 72.3, 51.5, 43.1, 36.3; **^{19}FNMR (376 MHz, Chloroform-*d*)**: δ -57.8; **HRMS (ESI, m/z)**: calcd. For $C_{27}H_{19}BrF_3N_4O_3^+$ $[M+H]^+$: 583.0587, found: 583.0588; **HPLC**: Chiralpak AD-H Column, hexane/^{i}PrOH = 70/30, 1.0 mL/min, 254 nm, 30 ℃; RT = 16.4 min (major), 13.2 min (minor), ee: 94%.

Compound 5m (Fig. 3)

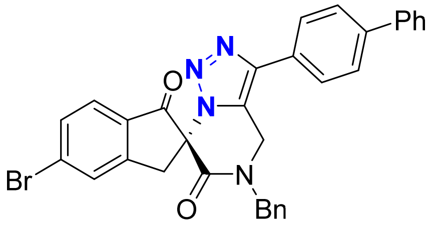

5m

(*R*)-3'-([1,1'-biphenyl]-4-yl)-5'-benzyl-5-bromo-4',5'-dihydro-6'*H*-spiro[indene-2,7'-[1,2,3]triazolo[1,5-*a*]pyrazine]-1,6'(3*H*)-dione (5m); **TLC**: R_f = 0.50 (petroleum ether /ethyl acetate = 4/1, v/v, UV); white solid (mp: 247-248 °C); yield: 50% (28.8 mg, 0.050 mmol); **$[\alpha]_D^{25}$** = 58 (c = 0.1, $CHCl_3$); **^{1}H NMR (400 MHz, $CDCl_3$)**: δ 7.87 (d, *J* = 1.5 Hz, 1H), 7.74-7.59 (m, 8H), 7.48-7.44 (m, 2H), 7.42-7.28 (m, 6H), 5.16 (d, *J* = 16.2 Hz, 1H), 4.98 (d, *J* = 15.0 Hz, 1H), 4.72 (dd, *J* = 20.7, 15.5 Hz, 2H), 4.45 (d, *J* = 17.2 Hz, 1H), 4.32 (d, *J* = 17.2 Hz, 1H); **^{13}C{^{1}H}NMR (101 MHz, $CDCl_3$)**: δ 195.4, 162.9, 155.6, 142.6, 141.3, 140.4, 134.8, 133.0, 132.5, 130.7, 130.1, 129.3, 129.1, 129.0, 128.5, 128.0, 127.8, 127.8, 127.2, 127.2, 126.9, 124.8, 72.2, 51.5, 43.3, 36.4; **HRMS (ESI, m/z)**: calcd. For $C_{32}H_{24}BrN_4O_2^+$ $[M+H]^+$: 575.1077, found: 575.1078; **HPLC**: Chiralpak OD-H Column, hexane/^{i}PrOH = 50/50, 1.0 mL/min, 254 nm, 30 ℃; RT = 25.8 min (major), 42.7 min (minor), ee: 94%.

Compound 5n (Fig. 3)

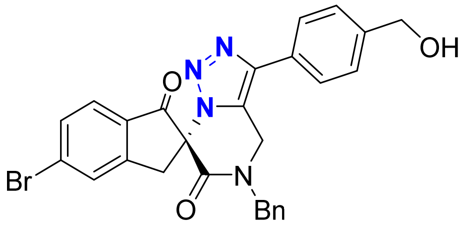

5n

(*R*)-5'-benzyl-5-bromo-3'-(4-(hydroxymethyl)phenyl)-4',5'-dihydro-6'*H*-spiro[indene-2,7'-[1,2,3]triazolo[1,5-*a*]pyrazine]-1,6'(3*H*)-dione (5n); **TLC**: R_f = 0.20 (petroleum ether /ethyl acetate = 1/1, v/v, UV); white solid (mp: 87-88 °C); yield: 51% (27.0 mg, 0.051 mmol); **$[\alpha]_D^{25}$** = 93 (c = 0.1, $CHCl_3$); **^{1}H NMR (400 MHz, $CDCl_3$)**: δ 7.86 (d, *J* = 1.4 Hz, 1H), 7.71-7.54 (m, 4H), 7.45-7.38 (m, 5H), 7.32-7.22 (m, 2H), 5.95 (brs, 1H), 5.11 (d, *J* = 16.3 Hz, 1H), 4.94 (d, *J* = 14.9 Hz, 1H), 4.71 (s, 2H), 4.69

(d, J = 16.4 Hz, 2H), 4.42 (d, J = 17.2 Hz, 1H), 4.30 (d, J = 17.2 Hz, 1H); **^{13}C{^{1}H}NMR (101 MHz, $CDCl_3$)**: δ 195.4, 162.9, 155.6, 142.7, 141.3, 134.8, 133.0, 132.5, 130.7, 130.1, 129.5, 129.3, 128.5, 128.1, 127.7, 127.2, 126.7, 124.8, 72.2, 65.0, 51.5, 43.2, 36.4; **HRMS (ESI, m/z)**: calcd. For $C_{27}H_{22}BrN_4O_3^+$ [M+H]$^+$: 529.0870, found: 529.0873; **HPLC**: Chiralpak AD-H Column, hexane/^{i}PrOH = 60/40, 1.0 mL/min, 254 nm, 30 ℃; RT = 12.2 min (major), 23.9 min (minor), ee: 92%.

Compound 5o (Fig. 3)

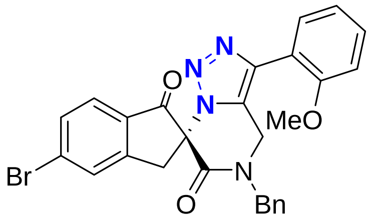

5o

(*R*)-5'-benzyl-5-bromo-3'-(2-methoxyphenyl)-4',5'-dihydro-6'*H*-spiro[indene-2,7'-[1,2,3]triazolo[1,5-*a*]pyrazine]-1,6'(3*H*)-dione (5o); **TLC**: R_f = 0.50 (petroleum ether /ethyl acetate = 5/1, v/v, UV); white solid (mp: 117-118 ºC); yield: 40% (21.2mg, 0.040 mmol); **$[\alpha]_D^{25}$** = 78 (c = 0.1, $CHCl_3$); **^{1}H NMR (400 MHz, $CDCl_3$)**: δ 7.86 (d, J = 1.5 Hz, 1H), 7.80 (dd, J = 7.6, 1.8 Hz, 1H), 7.69-7.57 (m, 2H), 7.43-7.27 (m, 6H), 7.09-7.05 (m, 1H), 6.92 (dd, J = 8.4, 1.0 Hz, 1H), 4.88 (d, J = 16.7 Hz, 1H), 4.78 (s, 2H), 4.62 (d, J = 16.7 Hz, 1H), 4.44 (d, J = 17.2 Hz, 1H), 4.31 (d, J = 17.2 Hz, 1H), 3.62 (s, 3H); **^{13}C{^{1}H}NMR (101 MHz, $CDCl_3$)**: δ 195.7, 162.9, 155.8, 155.6, 140.2, 135.1, 132.8, 132.3, 130.9, 130.8, 130.3, 130.0, 129.2, 128.4, 128.3, 127.1, 126.7, 121.4, 119.2, 111.1, 72.2, 55.2, 51.3, 43.6, 36.6; **HRMS (ESI, m/z)**: calcd. For $C_{27}H_{22}BrN_4O_3^+$ [M+H]$^+$: 529.0870, found: 529.0869; **HPLC**: Chiralpak AD-H Column, hexane/^{i}PrOH = 60/40, 1.0 mL/min, 254 nm, 30 ℃; RT = 30.3 min (major), 25.7 min (minor), ee: 92%.

Compound 5p (Fig. 3)

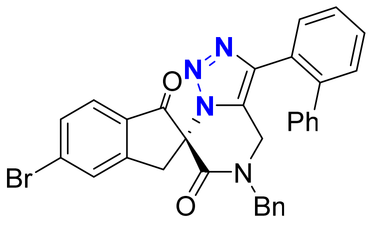

5p

(*R*)-3'-([1,1'-biphenyl]-2-yl)-5'-benzyl-5-bromo-4',5'-dihydro-6'*H*-spiro[indene-2,7'-[1,2,3]triazolo[1,5-*a*]pyrazine]-1,6'(3*H*)-dione (5p); **TLC**: R_f = 0.50 (petroleum ether /ethyl acetate = 5/1, v/v, UV); white solid (mp: 78-79 °C); yield: 60% (34.5 mg, 0.060 mmol); **$[\alpha]_D^{25}$** = 51 (c = 0.1, $CHCl_3$); **^{1}H NMR (400 MHz, $CDCl_3$)**: δ 7.84 (d, *J* = 1.5 Hz, 1H), 7.76 (dd, *J* = 6.9, 2.1 Hz, 1H), 7.67-7.57 (m, 2H), 7.52-7.38 (m, 3H), 7.35-7.31 (m, 3H), 7.25-7.12 (m, 5H), 7.02-7.00 (m, 2H), 4.85 (d, *J* = 14.7 Hz, 1H), 4.42 (d, *J* = 17.1 Hz, 1H), 4.23 (d, *J* = 17.2 Hz, 1H), 3.98 (d, *J* = 14.7 Hz, 1H), 3.46 (d, *J* = 16.6 Hz, 1H), 3.12 (d, *J* = 16.5 Hz, 1H); **^{13}C{^{1}H}NMR (101 MHz, $CDCl_3$)**: δ 195.2, 162.6, 155.5, 143.2, 140.6, 140.3, 134.6, 132.8, 132.4, 131.4, 130.7, 130.3, 130.0, 129.4, 129.2, 129.1, 129.0, 128.5, 128.2, 128.1, 128.0, 127.4, 127.1, 126.2, 72.2, 51.1, 42.0, 36.0; **HRMS (ESI, m/z)**: calcd. For $C_{32}H_{24}BrN_4O_2^+$ $[M+H]^+$: 575.1077, found: 575.1080; **HPLC**: Chiralpak AD-H Column, hexane/^{i}PrOH = 80/20, 1.0 mL/min, 254 nm, 30 ℃; RT = 19.8 min (major), 18.1 min (minor), ee: 93%.

Compound 5q (Fig. 3)

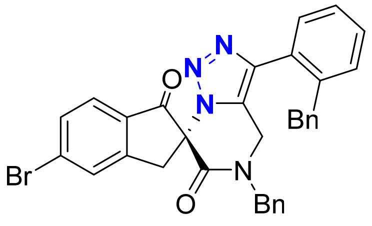

5q

(*R*)-5'-benzyl-3'-(2-benzylphenyl)-5-bromo-4',5'-dihydro-6'*H*-spiro[indene-2,7'-[1,2,3]triazolo[1,5-*a*]pyrazine]-1,6'(3*H*)-dione (5q); **TLC**: R_f = 0.50 (petroleum ether /ethyl acetate = 4/1, v/v, UV); white solid (mp: 88-89 °C); yield: 60% (35.3 mg, 0.060 mmol); **$[\alpha]_D^{25}$** = 63 (c = 0.1, $CHCl_3$); **^{1}H NMR (400 MHz, $CDCl_3$)**: δ 7.87 (d, *J* = 1.4 Hz, 1H), 7.74-7.58 (m, 2H), 7.40-7.24 (m, 6H), 7.17-7.14 (m, 3H), 7.12-7.08 (m, 2H),

7.06-7.01 (m, 1H), 6.89-6.84 (m, 2H), 4.82 (d, *J* = 14.8 Hz, 1H), 4.39 (d, *J* = 16.5 Hz, 2H), 4.34 (d, *J* = 14.8 Hz, 1H), 4.28 (d, *J* = 17.2 Hz, 1H), 4.07 (d, *J* = 15.2 Hz, 1H), 3.98 (d, *J* = 15.2 Hz, 1H), 3.83 (d, *J* = 16.3 Hz, 1H); **$^{13}C\{^1H\}$NMR (101 MHz, $CDCl_3$)**: δ 195.4, 162.8, 155.4, 142.7, 141.6, 141.0, 134.8, 132.9, 132.4, 131.0, 130.9, 130.5, 130.0, 129.5, 129.2, 129.1, 128.8, 128.3, 128.2, 128.1, 127.1, 126.7, 126.4, 126.0, 71.9, 51.3, 42.0, 39.7, 36.8; **HRMS (ESI, m/z)**: calcd. For $C_{33}H_{26}BrN_4O_2^+$ $[M+H]^+$: 589.1234, found: 589.1235; **HPLC**: Chiralpak AD-H Column, hexane/^{i}PrOH = 50/50, 1.0 mL/min, 254 nm, 30 ℃; RT = 51.8 min (major), 13.3 min (minor), ee: 94%.

Compound 5r (Fig. 3)

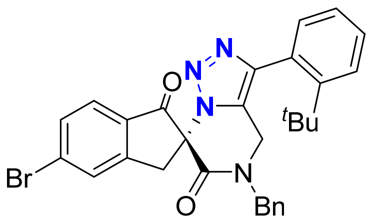

5r

(*R*)-5'-benzyl-5-bromo-3'-(2-(*tert*-butyl)phenyl)-4',5'-dihydro-6'*H*-spiro[indene-2,7'-[1,2,3]triazolo[1,5-*a*]pyrazine]-1,6'(3*H*)-dione (5r); **TLC**: R_f = 0.50 (petroleum ether /ethyl acetate = 4/1, v/v, UV); yellow oil; yield: 36% (20.0 mg, 0.036 mmol); **$[\alpha]_D^{25}$** = 102 (c = 0.1, $CHCl_3$); **^{1}H NMR (400 MHz, $CDCl_3$)**: δ 7.88 (s, 1H), 7.68 – 7.62 (m, 2H), 7.56 (dd, *J* = 8.1, 1.2 Hz, 1H), 7.40 – 7.29 (m, 4H), 7.21-7.17 (m, 3H), 6.98 (dd, *J* = 7.5, 1.6 Hz, 1H), 4.78 – 4.73 (m, 3H), 4.52 (d, *J* = 17.1 Hz, 1H), 4.34 (dd, *J* = 16.8, 14.3 Hz, 2H), 1.19 (s, 9H); **$^{13}C\{^1H\}$NMR (101 MHz, $CDCl_3$)**: δ 195.7, 163.0, 155.6, 150.9, 145.9, 134.7 133.01, 132.96, 132.4, 130.6, 130.1, 129.5, 129.2, 128.4, 128.2, 127.9, 127.3, 127.2, 126.5, 125.9, 72.5, 51.3, 42.2, 36.5, 35.9, 31.8; **HRMS (ESI, m/z)**: calcd. For $C_{30}H_{28}BrN_4O_2^+$ $[M+H]^+$: 555.1390, found: 555.1401; **HPLC**: Chiralpak AD-H Column, hexane/^{i}PrOH = 50/50, 1.0 mL/min, 254 nm, 30 ℃; RT = 59.8 min (major), 8.5 min (minor), ee: 93%.

Compound 5s (Fig. 3)

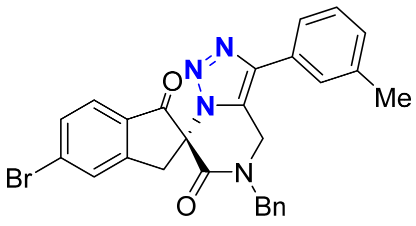

5s

(*R*)-5'-benzyl-5-bromo-3'-(m-tolyl)-4',5'-dihydro-6'*H*-spiro[indene-2,7'-[1,2,3]triazolo[1,5-*a*]pyrazine]-1,6'(3*H*)-dione (5s); **TLC**: R_f = 0.50 (petroleum ether /ethyl acetate = 4/1, v/v, UV); white solid (mp: 85-86 °C); yield: 65% (33.3 mg, 0.065 mmol); **[α]$_D^{25}$** = 92 (c = 0.1, $CHCl_3$); **^{1}H NMR (400 MHz, $CDCl_3$)**: δ 7.86 (s, 1H), 7.67-7.61 (m, 2H), 7.53 (s, 1H), 7.40-7.26 (m, 7H), 7.18 (t, *J* = 4.6 Hz, 1H), 5.12 (d, *J* = 16.1 Hz, 1H), 4.94 (d, *J* = 14.9 Hz, 1H), 4.70 (dd, *J* = 15.6, 3.5 Hz, 2H), 4.44 (d, *J* = 17.2 Hz, 1H), 4.30 (d, *J* = 17.2 Hz, 1H), 2.40 (s, 3H); **^{13}C{^{1}H}NMR (101 MHz, $CDCl_3$)**: δ 195.4, 162.9, 155.5, 143.0, 139.0, 134.8, 133.0, 132.4, 130.7, 130.0, 129.31, 129.27, 129.0, 128.4, 128.0, 127.3, 127.2, 125.9, 124.8, 123.5, 72.2, 51.5, 43.2, 36.3, 21.6; **HRMS (ESI, m/z)**: calcd. For $C_{27}H_{22}BrN_4O_2^+$ $[M+H]^+$: 513.0921, found: 513.0920; **HPLC**: Chiralpak AD-H Column, hexane/^{i}PrOH = 70/30, 1.0 mL/min, 254 nm, 30 ℃; RT = 22.4 min (major), 17.1 min (minor), ee: 90%.

Compound 5t (Fig. 3)

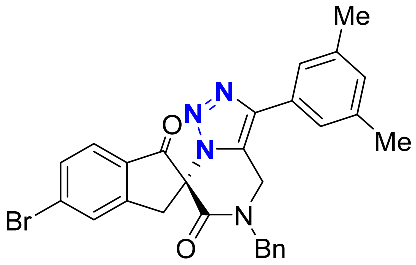

5t

(*R*)-5'-benzyl-5-bromo-3'-(3,5-dimethylphenyl)-4',5'-dihydro-6'*H*-spiro[indene-2,7'-[1,2,3]triazolo[1,5-*a*]pyrazine]-1,6'(3*H*)-dione (5t); **TLC**: R_f = 0.30 (petroleum ether /ethyl acetate = 4/1, v/v, UV); white solid (mp: 86-87 °C); yield: 50% (26.4 mg, 0.050 mmol); **[α]$_D^{25}$** = 121 (c = 0.1, $CHCl_3$); **^{1}H NMR (400 MHz, $CDCl_3$)**: δ 7.86 (s, 1H), 7.73-7.53 (m, 2H), 7.43-7.24 (m, 5H), 7.23 (s, 2H), 7.01 (s, 1H), 5.13 (d, *J* = 16.1 Hz, 1H), 4.92 (d, *J* = 14.9 Hz, 1H), 4.71 (dd, *J* = 15.5, 6.8 Hz, 2H), 4.45 (d, *J* = 17.2

Hz, 1H), 4.30 (d, J = 17.2 Hz, 1H), 2.35 (s, 6H); **^{13}C{^{1}H}NMR (101 MHz, CDCl$_3$)**: δ 195.4, 162.9, 155.6, 143.1, 138.8, 134.82, 132.9, 132.4, 130.7, 130.2, 130.0, 130.0, 129.3, 128.4, 128.0, 127.1, 124.7, 124.4, 72.2, 51.5, 43.2, 36.3, 21.5; **HRMS (ESI, m/z)**: calcd. For $C_{28}H_{24}BrN_4O_2^+$ [M+H]$^+$: 527.1077, found: 527.1080; **HPLC**: Chiralpak AD-H Column, hexane/^{i}PrOH = 50/50, 1.0 mL/min, 254 nm, 30 ℃; RT = 14.5 min (major), 12.8 min (minor), ee: 94%.

Compound 5u (Fig. 3)

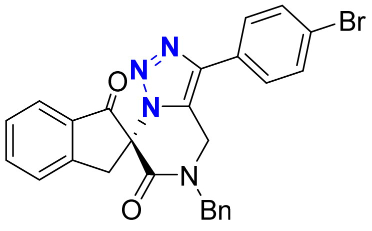

5u

(*R*)-5'-benzyl-3'-(4-bromophenyl)-4',5'-dihydro-6'*H*-spiro[indene-2,7'-[1,2,3]triazolo[1,5-*a*]pyrazine]-1,6'(3*H*)-dione (5u); **TLC**: R_f = 0.40 (petroleum ether /ethyl acetate = 4/1, v/v, UV); white solid (mp: 94-95 ºC); yield: 70% (34.9 mg, 0.070 mmol); **[α]$_D$^{25}** = 151 (c = 0.1, CHCl$_3$); **^{1}H NMR (400 MHz, CDCl$_3$)**: δ 7.83-7.75 (m, 2H), 7.68 (d, J = 7.8 Hz, 1H), 7.57 (d, J = 8.5 Hz, 2H), 7.53-7.46 (m, 3H), 7.41-7.29 (m, 5H), 5.12 (d, J = 16.1 Hz, 1H), 4.96 (d, J = 14.9 Hz, 1H), 4.68 (dd, J = 15.5, 9.3 Hz, 2H), 4.46 (d, J = 17.1 Hz, 1H), 4.34 (d, J = 17.1 Hz, 1H); **^{13}C{^{1}H}NMR (101 MHz, CDCl$_3$)**: δ 196.4, 163.1, 154.2, 141.9, 137.2, 134.8, 132.3, 131.7, 129.3, 129.2, 128.7, 128.5, 128.0, 128.0, 126.7, 126.2, 125.1, 122.5, 72.4, 51.5, 43.1, 36.6; **HRMS (ESI, m/z)**: calcd. For $C_{26}H_{20}BrN_4O_2^+$ [M+H]$^+$: 499.0764, found: 499.0760; **HPLC**: Chiralpak AD-H Column, hexane/^{i}PrOH = 70/30, 1.0 mL/min, 254 nm, 30 ℃; RT = 31.9 min (major), 18.7 min (minor), ee: 92%.

Compound 5v (Fig. 3)

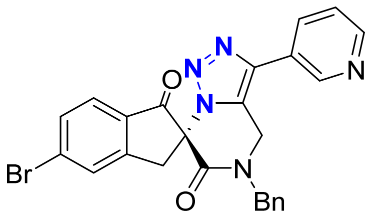

5v

(*R*)-5'-benzyl-5-bromo-3'-(pyridin-3-yl)-4',5'-dihydro-6'*H*-spiro[indene-2,7'-[1,2,3]triazolo[1,5-*a*]pyrazine]-1,6'(3*H*)-dione (5v); **TLC**: R_f = 0.20 (petroleum ether /ethyl acetate = 1/1, v/v, UV); white solid (mp: 109-110 ºC); yield: 60% (30.0 mg, 0.060 mmol); **[α]$_D^{25}$** = 102 (c = 0.1, $CHCl_3$); **^{1}H NMR (400 MHz, $CDCl_3$)**: δ 8.75 (s, 1H), 8.60 (s, 1H), 8.14 (d, *J* = 7.9 Hz, 1H), 7.87 (s, 1H), 7.72-7.61 (m, 2H), 7.46-7.27 (m, 6H), 5.13 (d, *J* = 16.3 Hz, 1H), 4.96 (d, *J* = 14.8 Hz, 1H), 4.72 (dd, *J* = 22.9, 15.5 Hz, 2H), 4.41 (d, *J* = 17.2 Hz, 1H), 4.31 (d, *J* = 17.2 Hz, 1H); **^{13}C{^{1}H}NMR (101 MHz, $CDCl_3$)**: δ 195.2, 162.6, 155.4, 149.4, 146.9, 139.8, 134.5, 134.1, 133.1, 132.5, 130.6, 130.0, 129.3, 128.6, 128.2, 127.2, 126.6, 125.6, 124.2, 72.2, 51.5, 43.0, 36.4; **HRMS (ESI, m/z)**: calcd. For $C_{25}H_{19}BrN_5O_2^+$ $[M+H]^+$: 500.0717, found: 500.0720; **HPLC**: Chiralpak AD-H Column, hexane/^{i}PrOH = 60/40, 1.0 mL/min, 254 nm, 30 ℃; RT = 17.2min (major), 20.5 min (minor), ee: 90%.

Compound 5w (Fig. 3)

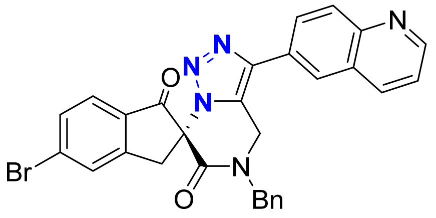

5w

(*R*)-5'-benzyl-5-bromo-3'-(quinolin-6-yl)-4',5'-dihydro-6'*H*-spiro[indene-2,7'-[1,2,3]triazolo[1,5-*a*]pyrazine]-1,6'(3*H*)-dione (5w); **TLC**: R_f = 0.20 (petroleum ether /ethyl acetate = 1/1, v/v, UV); white solid (mp: 119-120 ºC); yield: 60% (33.0 mg, 0.060 mmol); **[α]$_D^{25}$** = 106 (c = 0.1, $CHCl_3$); **^{1}H NMR (400 MHz, $CDCl_3$)**: δ 8.93 (d, *J* = 5.2 Hz, 1H), 8.20-8.16 (m, 2H), 8.09 (s, 1H), 7.96 (dd, *J* = 8.7, 2.0 Hz, 1H), 7.87 (s, 1H), 7.74-7.61 (m, 2H), 7.53-7.28 (m, 6H), 5.24 (d, *J* = 16.1 Hz, 1H), 4.99 (d, *J* = 15.0 Hz, 1H), 4.81 (d, *J* = 16.2 Hz, 1H), 4.71 (d, *J* = 15.0 Hz, 1H), 4.46 (d, *J* = 17.2 Hz, 1H), 4.33 (d, *J* = 17.2 Hz, 1H); **^{13}C{^{1}H}NMR (101 MHz, $CDCl_3$)**: δ 195.3, 162.7, 155.5, 151.1, 147.9, 142.2, 136.5, 134.7, 133.1, 132.5, 130.6, 130.5, 130.1, 129.3, 128.5, 128.5, 128.4, 128.0, 127.7, 127.2, 125.4, 125.4, 122.0, 72.3, 51.5, 43.3, 36.4; **HRMS (ESI, m/z)**: calcd. For $C_{29}H_{21}BrN_5O_2^+$ $[M+H]^+$: 550.0873, found: 550.0870; **HPLC**:

Chiralpak AD-H Column, hexane/^{i}PrOH = 60/40, 1.0 mL/min, 254 nm, 30 ℃; RT = 14.3 min (major), 25.0 min (minor), ee: 94%.

Compound 5x (Fig. 3)

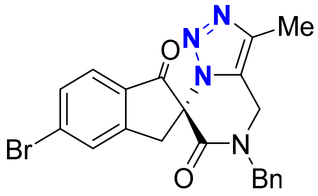

5x

(*R*)-5'-benzyl-5-bromo-3'-methyl-4',5'-dihydro-6'*H*-spiro[indene-2,7'-[1,2,3]triazolo[1,5-*a*]pyrazine]-1,6'(3*H*)-dione (5x); **TLC**: R_f = 0.30 (petroleum ether /ethyl acetate = 1/1, v/v, UV); white solid (mp: 206-207 °C); yield: 71% (31.0 mg, 0.071 mmol); **$[\alpha]_D^{25}$** = 105 (c = 0.1, $CHCl_3$); **^{1}H NMR (400 MHz, $CDCl_3$)**: δ 7.83 (s, 1H), 7.67-7.56 (m, 2H), 7.42-7.31 (m, 3H), 7.28-7.26 (m, 2H), 4.89 (d, *J* = 14.8 Hz, 1H), 4.80 (d, *J* = 16.0 Hz, 1H), 4.68 (d, *J* = 14.8 Hz, 1H), 4.44 (d, *J* = 16.0 Hz, 1H), 4.33 (d, *J* = 17.2 Hz, 1H), 4.24 (d, *J* = 17.2 Hz, 1H), 2.28 (s, 3H); **^{13}C{^{1}H}NMR (101 MHz, $CDCl_3$)**: δ 195.6, 163.0, 155.5, 138.9, 134.8, 132.9, 132.4, 130.7, 130.0, 129.2, 128.4, 128.1, 127.1, 125.1, 71.9, 51.4, 41.9, 36.5, 10.2; **HRMS (ESI, m/z)**: calcd. For $C_{21}H_{18}BrN_4O_2^+$ [M+H]$^+$: 437.0608, found: 437.0610; **HPLC**: Chiralpak AD-H Column, hexane/^{i}PrOH = 70/30, 1.0 mL/min, 254 nm, 30 ℃; RT = 10.7 min (major), 14.9 min (minor), ee: 95%.

Compound 5y (Fig. 3)

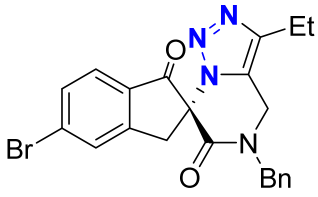

5y

(*R*)-5'-benzyl-5-bromo-3'-ethyl-4',5'-dihydro-6'*H*-spiro[indene-2,7'-[1,2,3]triazolo[1,5-*a*]pyrazine]-1,6'(3*H*)-dione (5y); **TLC**: R_f = 0.30 (petroleum ether /ethyl acetate = 1/1, v/v, UV); white solid (mp: 204-205 °C); yield: 60% (27.1 mg, 0.060

mmol); **[α]$_D^{25}$** = 133 (c = 0.1, $CHCl_3$); **^{1}H NMR (400 MHz, $CDCl_3$)**: δ 7.84 (d, *J* = 1.5 Hz, 1H), 7.68-7.56 (m, 2H), 7.42-7.31 (m, 3H), 7.29-7.24 (m, 2H), 4.85 (dd, *J* = 22.0, 15.4 Hz, 2H), 4.70 (d, *J* = 14.8 Hz, 1H), 4.46 (d, *J* = 15.9 Hz, 1H), 4.35 (d, *J* = 17.1 Hz, 1H), 4.25 (d, *J* = 17.2 Hz, 1H), 2.67 (q, *J* = 7.6 Hz, 2H), 1.25 (t, *J* = 7.6 Hz, 3H); **^{13}C{^{1}H}NMR (101 MHz, $CDCl_3$)**: δ 195.6, 163.1, 155.6, 144.4, 134.9, 132.8, 132.3, 130.8, 130.0, 129.2, 128.4, 128.1, 127.1, 124.6, 72.0, 51.4, 41.9, 36.5, 18.6, 13.3; **HRMS (ESI, m/z)**: calcd. For $C_{22}H_{20}BrN_4O_2^+$ $[M+H]^+$: 451.0764, found: 451.0760; **HPLC**: Chiralpak AD-H Column, hexane/^{i}PrOH = 70/30, 1.0 mL/min, 254 nm, 30 ℃; RT = 10.5 min (major), 14.2 min (minor), ee: 92%.

Compound 5z (Fig. 3)

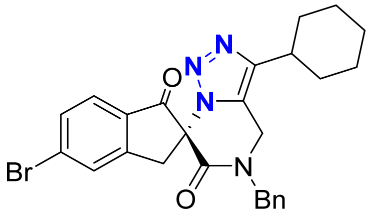

5z

(*R*)-5'-benzyl-5-bromo-3'-cyclohexyl-4',5'-dihydro-6'*H*-spiro[indene-2,7'-[1,2,3]triazolo[1,5-*a*]pyrazine]-1,6'(3*H*)-dione (5z); **TLC**: R_f = 0.50 (petroleum ether /ethyl acetate = 3/1, v/v, UV); white solid (mp: 70-71 ºC); yield: 50% (25.3 mg, 0.050 mmol); **[α]$_D^{25}$** = 39 (c = 0.1, $CHCl_3$); **^{1}H NMR (400 MHz, $CDCl_3$)**: δ 7.83 (s, 1H), 7.67-7.56 (m, 2H), 7.40-7.33 (m, 3H), 7.32-7.28 (m, 2H), 4.89 (dd, *J* = 18.8, 15.4 Hz, 2H), 4.67 (d, *J* = 14.9 Hz, 1H), 4.51 (d, *J* = 15.9 Hz, 1H), 4.36 (d, *J* = 17.2 Hz, 1H), 4.24 (d, *J* = 17.2 Hz, 1H), 2.67 (tt, *J* = 12.0, 3.6 Hz, 1H), 1.85 (dd, *J* = 29.0, 11.7 Hz, 4H), 1.71 (d, *J* = 11.5 Hz, 1H), 1.57-1.47 (m, 2H), 1.39-1.22 (m, 3H); **^{13}C{^{1}H}NMR (101 MHz, $CDCl_3$)**: δ 195.7, 163.1, 155.6, 147.8, 134.9, 132.8, 132.3, 130.8, 130.0, 129.2, 128.4, 128.0, 127.1, 123.9, 72.0, 51.4, 42.4, 36.5, 35.6, 32.3, 32.3, 26.4, 26.4, 25.9; **HRMS (ESI, m/z)**: calcd. For $C_{26}H_{26}BrN_4O_2^+$ $[M+H]^+$: 505.1234, found: 505.1230; **HPLC**: Chiralpak AD-H Column, hexane/^{i}PrOH = 70/30, 1.0 mL/min, 254 nm, 30 ℃; RT = 15.9 min (major), 9.9 min (minor), ee: 93%.

1.7. Procedure for synthesis of 3a in gram-scale

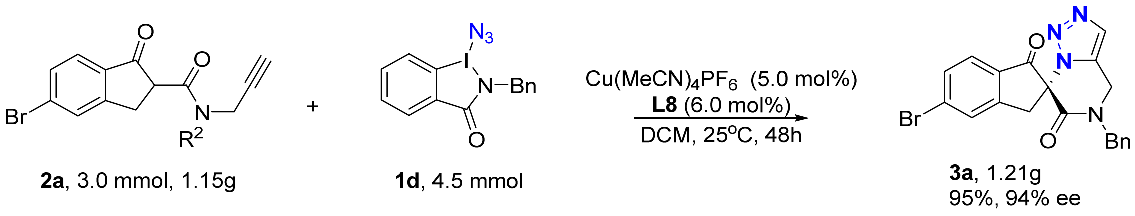

After stirring a mixture of $Cu(CH_3CN)_4PF_6$ (5 mol %) and **L8** (6 mol %) in dry dichloromethane (10 mL) at 25 ºC under nitrogen atmosphere at for 2 h, substrates **2a** (3.0 mmol) and **1d** (4.5 mmol) in dry dichloromethane (30 mL) was added and the reaction mixture was stirred at 25 ºC under nitrogen atmosphere. After 48 h, when the substrates **2a** was disappeared (monitored by TLC), the reaction mixture was stirred at 40 ºC for another 48 h. Finally, the crude product was purified by silica gel flash chromatography to afford the desired product **3a** (1.21g, 95% yield, 94% ee).

2. Supplementary Discussion

2.1. Radical inhibition experiments

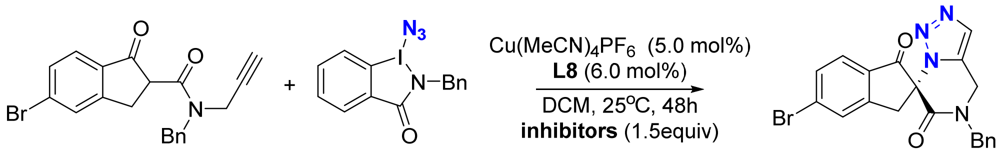

2a 1d Tempo, BHT, DBE **3a**, trace

The radical inhibition experiments were carried out according to the general procedure: After stirring a mixture of $Cu(CH_3CN)_4PF_6$ (5 mol %) and **L8** (6 mol %) in dry dichloromethane (1 mL) at 25 °C under nitrogen atmosphere for 1.5 h, substrates **2a** (0.1 mmol), **1d** (0.15 mmol) and inhibitors (1.5 equiv) in dry dichloromethane (1 mL) was added and the reaction mixture was stirred at 25 °C under nitrogen atmosphere. After 48 h, the reaction was monitored by TLC and ^{1}H NMR, all of these control reactions did not work.

2.2. Nonlinear effect study

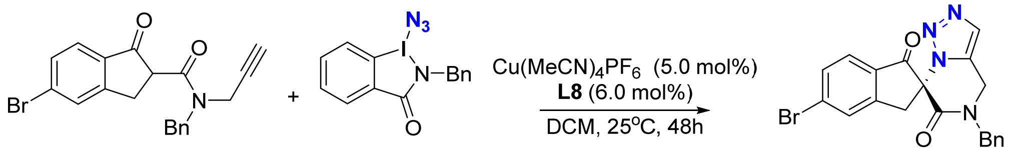

2a **1d** **3a**

The nonlinear effect study experiments were carried out according to the general procedure:After stirring a mixture of $Cu(CH_3CN)_4PF_6$ (0.005 mmol, 5 mol %) and **L8** (0.006 mmol, 6 mol %) in dry dichloromethane (1 mL) at 25 °C under nitrogen atmosphere for 1.5 h, substrates **2a** (0.10 mmol) and **1d** (0.15 mmol) in dry dichloromethane (1 mL) was added and the reaction mixture was stirred at 25 °C under nitrogen atmosphere. After 48 h, the substrates **2a** was disappeared (monitored by TLC) completely. The crude product was purified by silica gel flash chromatography to afford the desired product **3a**. And the ee values were determined by HPLC analysis.

Supplementary Table 5. Nonlinear effect study comparing the ee of **L8** with the ee of the product **3a**

entry	ee of L8 (%)	ee of 3a (%)
1	0	0
2	20	20
3	40	39
4	60	55
5	80	77
6	99	94

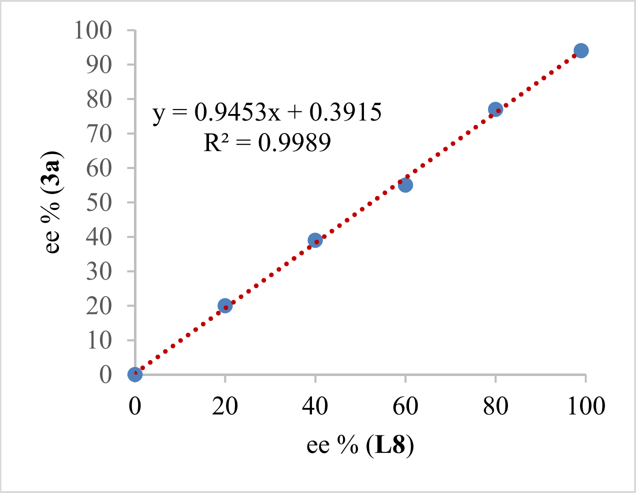

Supplementary Fig. 1. Nonlinear effect study comparing the ee of **L8** with the ee of the product **3a**

2.3. Investigation of intermediate azide

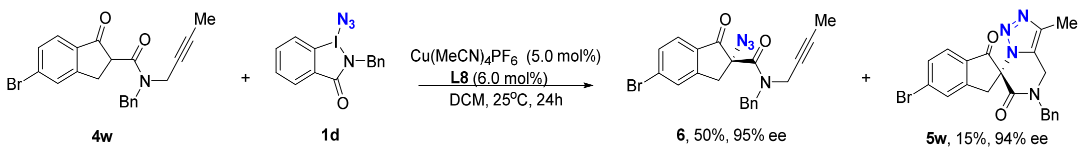

The investigation of intermediate azide experiment was carried out according to the general procedure: After stirring a mixture of $Cu(CH_3CN)_4PF_6$ (0.005 mmol, 5 mol %) and **L8** (0.006 mmol, 6 mol %) in dry dichloromethane (1 mL) at 25 °C under

nitrogen atmosphere for 1.5 h, substrates **4w** (0.10 mmol) and **1d** (0.15 mmol) in dry dichloromethane (1 mL) was added and the reaction mixture was stirred at room temperature under nitrogen atmosphere. The reaction was terminated after stirring for 24 h. The crude product was purified by silica gel flash chromatography to afford the desired product **6** with 50% yield and 95% ee.

Compound 6 (Fig. 4)

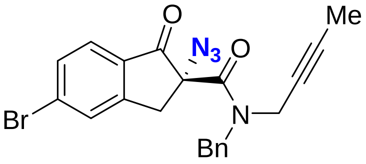

6

(*R*)-2-azido-*N*-benzyl-5-bromo-*N*-(but-2-yn-1-yl)-1-oxo-2,3-dihydro-1*H*-indene-2-carboxamide (6); **TLC**: R_f = 0.70 (petroleum ether /ethyl acetate = 3/1, v/v, UV); white solid; yield; $[\alpha]_D^{25}$ = 229 (c = 0.1, $CHCl_3$); **^{1}H NMR (400 MHz, $CDCl_3$)**: δ 7.71 (d, *J* = 8.1 Hz, 1H), 7.66-7.58 (m, 2H), 7.45-7.15 (m, 5H), 4.74 (s, 2H), 4.40-3.84 (m, 2H), 3.75 (d, *J* = 17.5 Hz, 1H), 3.14 (d, *J* = 17.4 Hz, 1H), 1.86 (s, 3H); **$^{13}C\{^1H\}$NMR (101 MHz, $CDCl_3$)**: δ 195.7, 167.4, 151.8, 135.4, 132.7, 132.4, 131.9, 129.9, 128.9, 128.5, 128.0, 126.8, 84.2, 73.0, 69.4, 48.8, 38.3, 37.0, 3.7; **HRMS (ESI, m/z)**: calcd. For $C_{21}H_{18}BrN_4O_2^+$ $[M+H]^+$: 437.0608, found:437.0600; **HPLC**: Chiralpak AD-H Column, hexane/^{i}PrOH = 90/10, 1.0 mL/min, 254 nm, 30 ℃; RT = 18.9 min (major), 20.4 min (minor), ee: 95%.

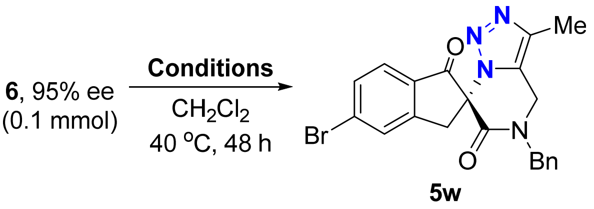

Conditions	5w
**A**: $Cu(MeCN)_4PF_6$ (5 mol %) **L8** (6 mol %)	75%, 94% ee
**B**: --	10%, 95% ee
**C**: $Cu(MeCN)_4PF_6$ (5 mol %)	31%, 94% ee

Condition A: After stirring a mixture of $Cu(CH_3CN)_4PF_6$ (0.005 mmol, 5 mol %) and **L8** (0.006 mmol, 6 mol %) in dry dichloromethane (1 mL) at 25 °C under nitrogen atmosphere for 1.5 h, intermediated **6** (0.1 mmol) was added and the reaction was stirred at 40 °C under nitrogen atmosphere for 48 h. when the intermediated **6** was disappeared

and the product **5w** was detected (monitored by TLC), the crude product was purified by silica gel flash chromatography to afford the desired product **5w** with 75% yield and 94% ee.

Condition B: The intermediated **6** (0.05 mmol) was dissolved in dry dichloromethane (1 mL) and stirred at 40 ^{o}C under nitrogen atmosphere for 48 h, the reaction was terminated and the crude product was purified by silica gel flash chromatography to afford the desired product **5w** with 10% yield and 95% ee.

Condition C: After stirring a mixture of $Cu(CH_3CN)_4PF_6$ (0.005 mmol, 5 mol %) and **6** (0.1 mmol) in dry dichloromethane (1 mL) at 40 ^{o}C under nitrogen atmosphere for 48 h, the reaction was terminated and the crude product was purified by silica gel flash chromatography to afford the desired product **5w** with 31% yield and 94% ee.

2.4. Control experiments with other substrates

2.4.1 General Procedure for the synthesis of racemic **7a-7d**

7a and **7b** were synthesized according to the literature procedures.[8-9] **7c** and **7d** were synthesized according to the general procedure.

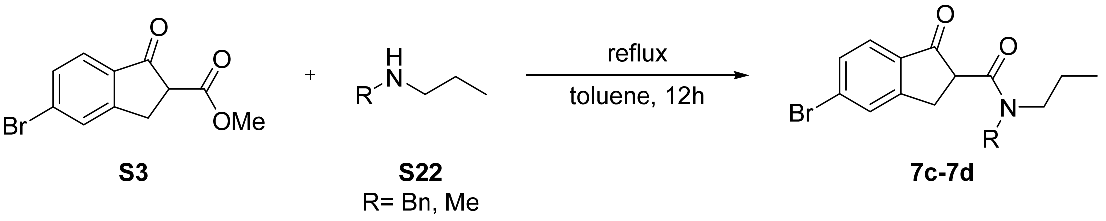

S3 (1 mmol, 1.0 equiv) were dissolved in dry toluene (20 mL), then propylamine **S22** (1.5 mmol) was added, and the mixture was stirring at 110 oC for 12 h. After the disappearance of substrate (monitored by TLC), the mixture was cooled to room temperature and was quenched with HCl (aq.). The residue was extracted three times with ethyl acetate, and was then washed with $NaHCO_3$ (aq.). Finally, the crude product was purified by silica gel flash chromatography to get the desired products **7c-7d**.

Compound 7c (Fig. 4)

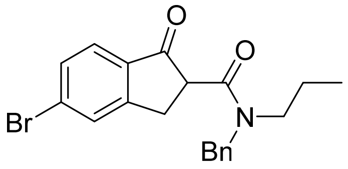

7c

***N*-benzyl-5-bromo-1-oxo-*N*-propyl-2,3-dihydro-1*H*-indene-2-carboxamide**; **TLC**: R*f* = 0.40 (petroleum ether /ethyl acetate = 10/1, v/v, UV); yellow oil; Enol isomerization were observed by NMR; **^{1}H NMR (400 MHz, CDCl$_3$)**: δ 7.67 (dd, J = 17.7, 1.5 Hz, 1H), 7.58 (dd, J = 15.8, 8.2 Hz, 1H), 7.50 (ddd, J = 9.9, 8.4, 1.5 Hz, 1H), 7.39-7.22 (m, 5H), 5.33 (d, J = 17.7 Hz, 0.5H), 5.01 (d, J = 15.2 Hz, 0.5H), 4.56 (d, J = 17.8 Hz, 0.5H), 4.34 (d, J = 15.2 Hz, 0.5H), 4.13 (dd, J = 7.9, 3.8 Hz, 0.5H), 3.96 (dd, J = 7.9, 3.6 Hz, 0.5H), 3.85-3.73 (m, 2H), 3.32-3.19 (m, 1H), 3.34-2.99 (m, 1H), 1.77-1.58 (m, 2H), 0.91 (dt, J = 10.3, 7.4 Hz, 3H); **^{13}C{^{1}H}NMR (101 MHz, CDCl$_3$)**: δ 200.9 (200.8), 168.41 (168.39), 156.44 (156.40), 137.3 (137.2), 134.4 (134.3), 131.33 (131.27), 130.84 (130.82), 129.91 (129.87), 129.1 (128.7), 127.7 (127.3) 127.6 (126.2), 125.7 (125.6), 51.4 (51.1), 50.5 (49.4), 49.1 (48.9), 30.9 (30.6), 22.2 (20.9), 11.42 (11.35); **HRMS (ESI, m/z)**: calcd. For $C_{20}H_{21}BrNO_2^+$ $[M+H]^+$: 386.0750, found: 386.0741.

Compound 7d (Fig. 4)

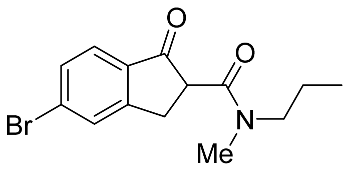

7d

5-bromo-*N*-methyl-1-oxo-*N*-propyl-2,3-dihydro-1*H*-indene-2-carboxamide; **TLC**: R*f* = 0.40 (petroleum ether /ethyl acetate = 10/1, v/v, UV); yellow oil; Enol isomerization were observed by NMR; **^{1}H NMR (400 MHz, CDCl$_3$)**: δ 7.66 (d, *J* = 1.6 Hz, 1H), 7.53 (dd, *J* = 8.2, 4.2 Hz, 1H), 7.47 (d, *J* = 8.1 Hz, 1H), δ 4.09 (dd, *J* = 7.9, 3.6 Hz, 0.6H), 4.04 (dd, *J* = 7.9, 3.6 Hz, 0.4H), 3.84 – 3.78 (m, 0.4H), 3.77 – 3.67 (m, 1H), 3.47 (dt, *J* = 13.1, 7.5 Hz, 0.6H), 3.34-3.16 (m, 4H), 2.97 (s, 1H), 1.78-1.54 (m, 2H), 0.91 (dt, *J* = 27.1, 7.5 Hz, 3H); **^{13}C{^{1}H}NMR (101 MHz, CDCl$_3$)**: δ 200.7 (200.5), 167.6 (167.4), 156.23 (156.21), 134.22 (134.18), 131.02 (130.97), 130.48 (130.46),

129.68 (129.67), 125.33 (125.29), 52.0 (50.8), 50.2 (50.0), 36.0 (34.1), 30.7 (30.2), 21.8 (20.3), 11.12 (11.08); **HRMS (ESI, m/z)**: calcd. For $C_{14}H_{17}BrNO_2^+$ [M+H]$^+$: 310.0473, found: 310.0473.

2.4.2 General Procedure for the synthesis of racemic **8a-8d**

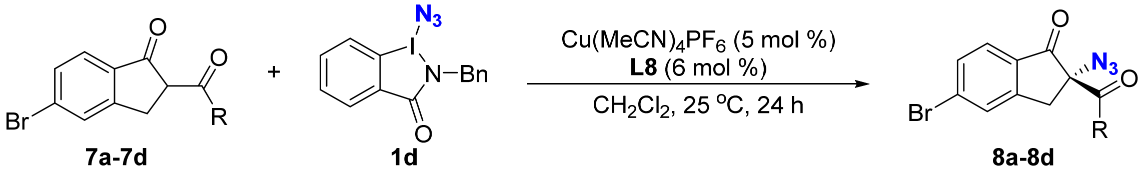

After stirring a mixture of $Cu(MeCN)_4PF_6$ (0.005 mmol, 5 mol %) and **L8** (0.006 mmol, 6 mol %) in dry dichloromethane (1 mL) at 25 °C under nitrogen atmosphere for 1.5 h, substrates **7a-7d** (0.10 mmol) and **1d** (0.15 mmol) in dry dichloromethane (1 mL) was added and the reaction mixture was stirred at 25 °C under nitrogen atmosphere. After 24 h, the substrates **7a-7d** was disappeared (monitored by TLC) completely. Finally, the crude product was purified by silica gel flash chromatography to afford the desired products **8a-8d**.

Compound 8a (Fig. 4)

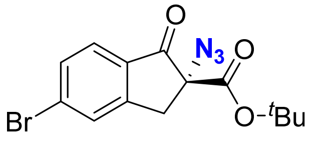

8a

The NMR of **8a** according to the literature[8].

tert-butyl (*R*)-2-azido-5-bromo-1-oxo-2,3-dihydro-1*H*-indene-2-carboxylate (8a); **TLC**: R_f = 0.80 (petroleum ether /ethyl acetate = 10/1, v/v, UV); colorless oil; yield: 91% (32.0 mg, 0.091 mmol); **$[\alpha]_D^{25}$** = 115 (c = 1.0, $CHCl_3$); {ref. 8: $[\alpha]_D^{25}$ +143.2 (*c* = 0.94, $CHCl_3$, 90% ee, *R* absolute configuration)}; **^{1}H NMR (400 MHz, $CDCl_3$)**: δ 7.67 (d, *J* = 8.2 Hz, 1H), 7.64 (d, *J* = 1.5 Hz, 1H), 7.58 (dd, *J* = 8.2, 1.6 Hz, 1H), 3.61 (d, *J* = 17.4 Hz, 1H), 2.96 (d, *J* = 17.4 Hz, 1H), 1.46 (s, 9H); **HPLC**: Chiralpak AD-H Column, hexane/^{i}PrOH = 99/1, 1.0 mL/min, 254 nm, 30 ℃; RT = 12.6 min (major), 10.6 min (minor), ee: 55%.

Compound 8b (Fig. 4)

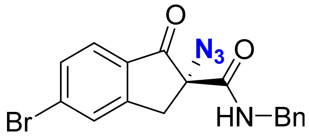

8b

(*R*)-2-azido-*N*-benzyl-5-bromo-1-oxo-2,3-dihydro-1*H*-indene-2-carboxamide (8b); **TLC**: R_f = 0.40 (petroleum ether /ethyl acetate = 5/1, v/v, UV); colorless oil; yield: 71% (27.3 mg, 0.071 mmol); **$[\alpha]_D^{25}$** = 29 (c = 0.5, $CHCl_3$); **^{1}H NMR (400 MHz, $CDCl_3$)**: δ 7.71 (s, 1H), 7.67 (d, *J* = 8.2 Hz, 1H), 7.59 (d, *J* = 8.3 Hz, 1H), 7.40 – 7.23 (m, 5H), 7.06 (s, 1H), 4.57 – 4.40 (m, 2H), 4.02 (d, *J* = 17.5 Hz, 1H), 3.23 (d, *J* = 17.5 Hz, 1H); **^{13}C{^{1}H}NMR (101 MHz, $CDCl_3$)**: δ 197.0, 166.0, 153.9, 137.3, 132.6, 132.4, 132.3, 130.0, 129.0, 127.9, 127.8, 126.7, 73.0, 44.2, 37.5; **HRMS (ESI, m/z)**: calcd. For $C_{17}H_{14}BrN_4O_2^+$ $[M+H]^+$: 385.0295, found: 385.0289; **HPLC**: Chiralpak AD-H Column, hexane/^{i}PrOH = 80/20, 1.0 mL/min, 254 nm, 30 ℃; RT = 10.0 min (major), 14.6 min (minor), ee: 46%.

Compound 8c (Fig. 4)

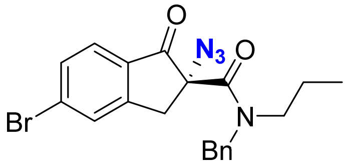

8c

(*R*)-2-azido-*N*-benzyl-5-bromo-1-oxo-*N*-propyl-2,3-dihydro-1*H*-indene-2-carboxamidee (8c); **TLC**: R_f = 0.60 (petroleum ether /ethyl acetate = 10/1, v/v, UV); colorless oil; yield: 90% (38.4 mg, 0.090 mmol); **$[\alpha]_D^{25}$** = 199 (c = 1.0, $CHCl_3$); two rotamers were observed by NMR;[10-14] **^{1}H NMR (400 MHz, $CDCl_3$)**: δ 7.73-7.68 (m, 1H), 7.63-7.57 (m, 2H), 7.37-7.17 (m, 5H), 4.80-4.59 (m, 2H), 3.57 (dd, *J* = 36.5, 17.4 Hz, 1H), 3.29-3.07 (m, 3H), 1.65-1.53 (m, 2H), 0.80 (dt, *J* = 27.2, 7.5 Hz, 3H); **^{13}C{^{1}H}NMR (101 MHz, $CDCl_3$)**: δ 196.6, 167.5, 151.8, 136.7 (136.0), 132.7 (132.4), 131.8, 129.9, 129.0 (128.9), 128.0, 127.7, 126.8, 126.7, 72.2 (71.8), 50.9 (48.6), 48.3, 38.6 (38.5), 21.2 (20.1), 11.3 (11.2); **HRMS (ESI, m/z)**: calcd. For $C_{20}H_{20}BrN_4O_2^+$ $[M+H]^+$: 427.0764, found: 427.0760; **HPLC**: Chiralpak AD-H Column, hexane/^{i}PrOH

= 90/10, 1.0 mL/min, 254 nm, 30 ℃; RT = 21.8 min (major), 17.5 min (minor), ee: 90%.

Compound 8d (Fig. 4)

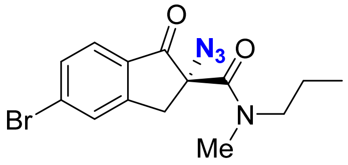

8d

(*R*)-2-azido-5-bromo-*N*-methyl-1-oxo-N-propyl-2,3-dihydro-1*H*-indene-2-carboxamide (8d); **TLC**: R_f = 0.60 (petroleum ether /ethyl acetate = 10/1, v/v, UV); colorless oil; yield: 85% (29.8 mg, 0.085 mmol); $[\alpha]_D^{25}$ = 232 (c = 1.0, $CHCl_3$); **^{1}H NMR (400 MHz, $CDCl_3$)**: δ 7.69 (d, *J* = 8.2 Hz, 1H), 7.62 (d, *J* = 1.6 Hz, 1H), 7.58 (dd, *J* = 8.1, 1.6 Hz, 1H), 3.55 (d, *J* = 17.4 Hz, 1H), 3.40-3.26 (m, 2H), 3.13 (d, *J* = 17.4 Hz, 1H), 2.97 (d, *J* = 17.0 Hz, 3H), 1.59 (q, *J* = 7.5 Hz, 2H), 0.90-0.88 (m, 3H).; **^{13}C{^{1}H}NMR (101 MHz, $CDCl_3$)**: δ 196.7, 166.7, 151.8, 132.7, 132.4, 131.7, 129.9, 126.6, 72.0, 51.2, 38.3, 35.4, 20.1, 11.2; **HRMS (ESI, m/z)**: calcd. For $C_{20}H_{20}BrN_4O_2^+$ $[M+H]^+$: 351.0451, found: 351.0442. **HPLC**: Chiralpak AD-H Column, hexane/^{i}PrOH = 80/20, 1.0 mL/min, 254 nm, 30 ℃; RT = 8.8 min (major), 7.8 min (minor), ee: 83%.

2.5. DFT calculations

For both conformations, the systems were set with a charge and spin multiplicity of 1 and 3, respectively. Optimizations were then performed at the M06-2X/def2-SVP level, incorporating Grimme's D3 empirical dispersion correction[15]. The Polarizable Continuum Model (PCM) was employed across all calculations, utilizing dichloromethane as the solvent to mimic the dielectric environment of the solution. Subsequently, frequency calculations were conducted to obtain the free energies. All computational analyses were executed using the Gaussian 16 software.

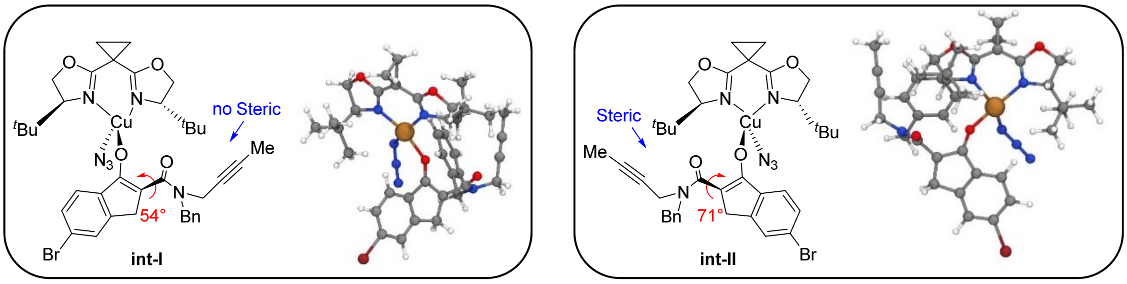

Supplementary Fig. 2. Chemical structure of **int-I** and **int-II**

Discussions: The favored conformation exhibits an energy that is 8.337718 kcal/mol lower than that of the other side. This discrepancy is primarily attributed to the steric hindrance caused by the carbonyl group and the tert-butyl group. Such hindrance leads to an increase in the angle between the exocyclic amide carbonyl and the five-membered ring from 54 degrees on the left to 71 degrees on the right. The enlargement of this angle results in a deterioration of conjugation, which not only elevates the energy level but also makes the enol double bond more reactive due to the loss of conjugation.

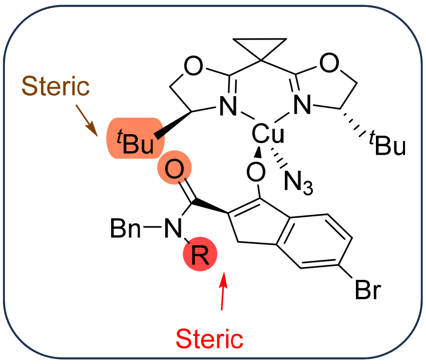

Supplementary Fig. 3. The influence of *N*-propargyl group

Discussions: Additionally, the enlargement of the angle causes the groups attached to the amide, such as the alkynyl group in this case, to rotate towards the backside of the α-carbon, thereby obstructing the approach of azides, as depicted in the figure below

3. Supplementary Notes

3.1 Determination of the Absolute Configuration of 5b by X-ray Analysis .

Compound 5b (Fig. 3)

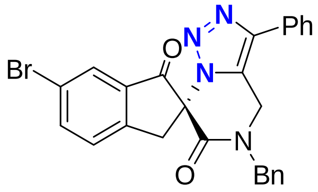

5b

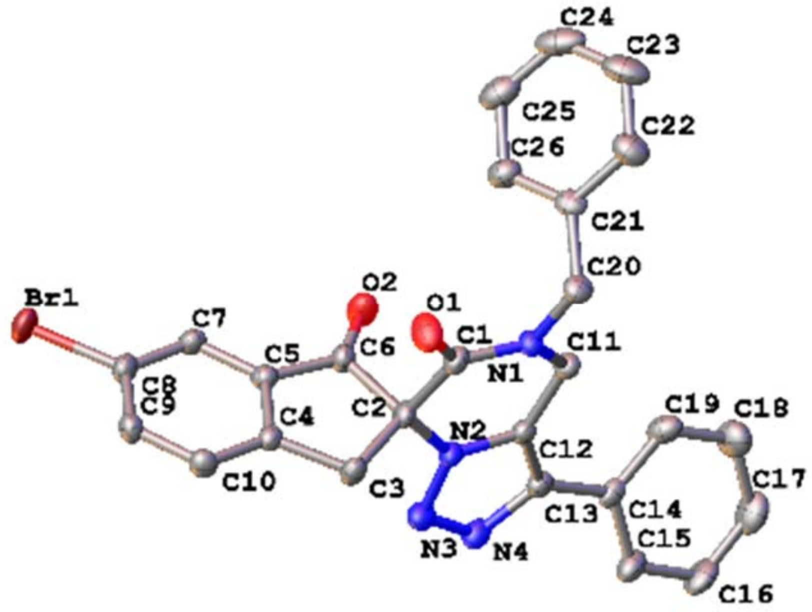

Supplementary Fig. 4. X-ray analysis to determine the absolutecon figuration of compound **5b** (CCDC2327853)

Supplementary Table 6. Crystal data and structure refinement for **5b** (CCDC2327853)

Identification code	231127cx_a	
Empirical formula	C_{26} H_{19} Br N_4 O_2	
Formula weight	499.36	
Temperature	173.00 K	
Wavelength	1.34139 Å	
Crystal system	Orthorhombic	
Space group	$P2_12_12_1$	
Unit cell dimensions	a = 10.7747(2) Å	a= 90°.

	b = 10.8254(2) Å	b= 90°.
	c = 18.5960(3) Å	g = 90°.
Volume	2169.05(7) $Å^3$	
Z	4	
Density (calculated)	1.529 Mg/m^3	
Absorption coefficient	1.874 mm^{-1}	
F(000)	1016	
Crystal size	0.17 x 0.17 x 0.05 mm^3	
Theta range for data collection	4.111 to 54.949°.	
Index ranges	-13<=h<=13, -13<=k<=13, -22<=l<=20	
Reflections collected	19722	
Independent reflections	4113 [R(int) = 0.0469]	
Completeness to theta = 53.594°	99.8 %	
Absorption correction	Semi-empirical from equivalents	
Max. and min. transmission	0.7508 and 0.5919	
Refinement method	Full-matrix least-squares on F^2	
Data / restraints / parameters	4113 / 0 / 298	
Goodness-of-fit on F^2	1.061	
Final R indices [I>2sigma(I)]	R1 = 0.0292, wR2 = 0.0624	
R indices (all data)	R1 = 0.0352, wR2 = 0.0654	
Absolute structure parameter	0.021(10)	
Extinction coefficient	n/a	
Largest diff. peak and hole	0.263 and -0.512 e.$Å^{-3}$	

3.2 Determination of the Absolute Configuration of 5d by X-ray Analysis

Compound 5d (Fig. 3)

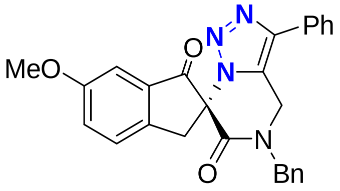

5d

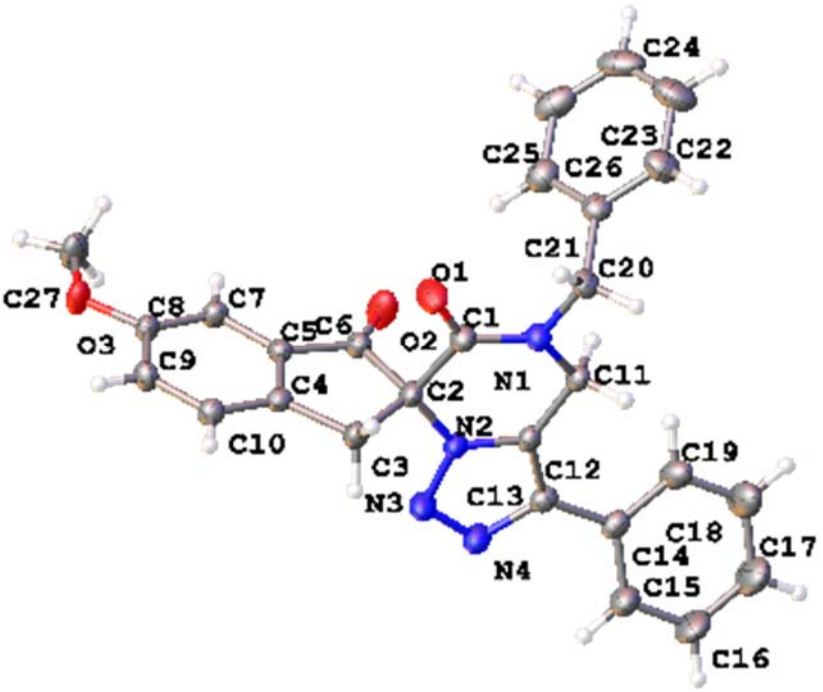

Supplementary Fig. 5. X-ray analysis to determine the absolutecon figuration of compound **5d** (CCDC2327852)

Supplementary Table 7. Crystal data and structure refinement for **5d** (CCDC2327852)

Identification code	231127cx_c	
Empirical formula	$C_{27} H_{21} N_4 O_3$	
Formula weight	449.48	
Temperature	173.00 K	
Wavelength	1.34139 Å	
Crystal system	Orthorhombic	
Space group	$P2_12_12_1$	
Unit cell dimensions	a = 10.8149(2) Å	a= 90°.
	b = 11.3892(2) Å	b= 90°.

	c = 18.1350(4) Å	g = 90°.
Volume	2233.74(8) $Å^3$	
Z	4	
Density (calculated)	1.337 Mg/m^3	
Absorption coefficient	0.461 mm^{-1}	
F(000)	940	
Crystal size	0.17 x 0.17 x 0.05 mm^3	
Theta range for data collection	4.141 to 54.907°.	
Index ranges	-13<=h<=12, -13<=k<=13, -21<=l<=22	
Reflections collected	28567	
Independent reflections	4236 [R(int) = 0.0553]	
Completeness to theta = 53.594°	99.8 %	
Absorption correction	Semi-empirical from equivalents	
Max. and min. transmission	0.7508 and 0.6201	
Refinement method	Full-matrix least-squares on F^2	
Data / restraints / parameters	4236 / 0 / 308	
Goodness-of-fit on F^2	1.055	
Final R indices [I>2sigma(I)]	R1 = 0.0354, wR2 = 0.0949	
R indices (all data)	R1 = 0.0396, wR2 = 0.0970	
Absolute structure parameter	0.10(9)	
Extinction coefficient	n/a	
Largest diff. peak and hole	0.439 and -0.351 e.$Å^{-3}$	

3.3 Determination of the Absolute Configuration of 5x by X-ray Analysis

Compound 5x (Fig. 4)

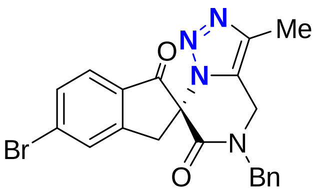

5x

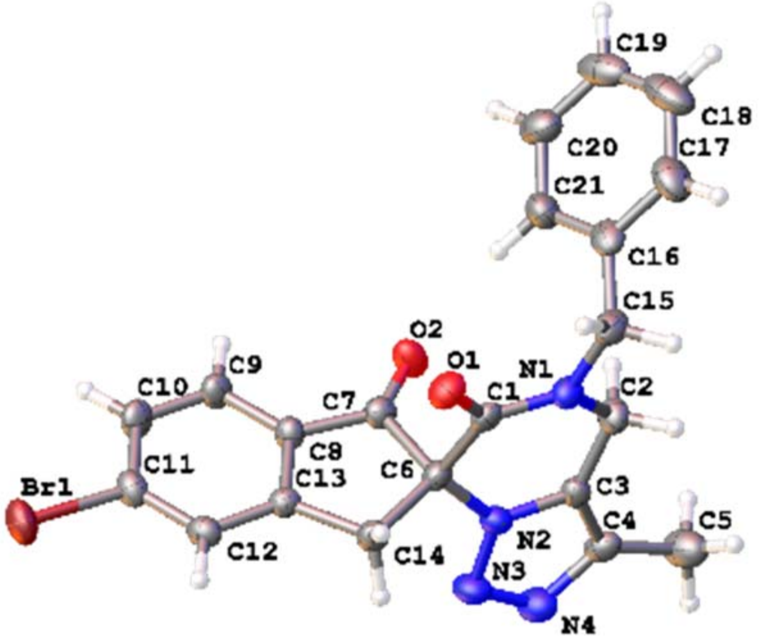

Supplementary Fig. 6. X-ray analysis to determine the absolutecon figuration of compound **5x** (CCDC2327851)

Supplementary Table 8. Crystal data and structure refinement for **5x** (CCDC2327851)

Identification code	231116cx_6_2a	
Empirical formula	C_{21} H_{16} Br N_4 O_2	
Formula weight	436.29	
Temperature	173.00 K	
Wavelength	1.34139 Å	
Crystal system	Orthorhombic	
Space group	$P2_12_12_1$	
Unit cell dimensions	a = 8.8875(2) Å	a= 90°.
	b = 10.0683(2) Å	b= 90°.
	c = 21.2805(4) Å	g = 90°.

Volume	1904.22(7) $Å^3$	
Z	4	
Density (calculated)	1.522 Mg/m^3	
Absorption coefficient	2.075 mm^{-1}	
F(000)	884	
Crystal size	0.17 x 0.17 x 0.05 mm^3	
Theta range for data collection	3.614 to 54.919°.	
Index ranges	-10<=h<=10, -10<=k<=12, -20<=l<=25	
Reflections collected	11847	
Independent reflections	3597 [R(int) = 0.0478]	
Completeness to theta = 53.594°	99.8 %	
Absorption correction	Semi-empirical from equivalents	
Max. and min. transmission	0.7508 and 0.4592	
Refinement method	Full-matrix least-squares on F^2	
Data / restraints / parameters	3597 / 0 / 254	
Goodness-of-fit on F^2	1.060	
Final R indices [I>2sigma(I)]	R1 = 0.0383, wR2 = 0.0850	
R indices (all data)	R1 = 0.0498, wR2 = 0.0907	
Absolute structure parameter	0.048(14)	
Extinction coefficient	n/a	
Largest diff. peak and hole	0.559 and -0.506 e.$Å^{-3}$	

4. Supplementary Figures

4.1 NMR Spectra of New Compounds (^{1}H NMR, ^{13}C NMR and ^{19}F NMR)

S9i, ^{1}H NMR (400 MHz, $CDCl_3$)

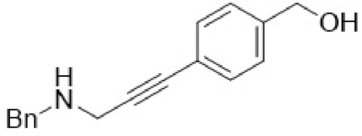

S9i

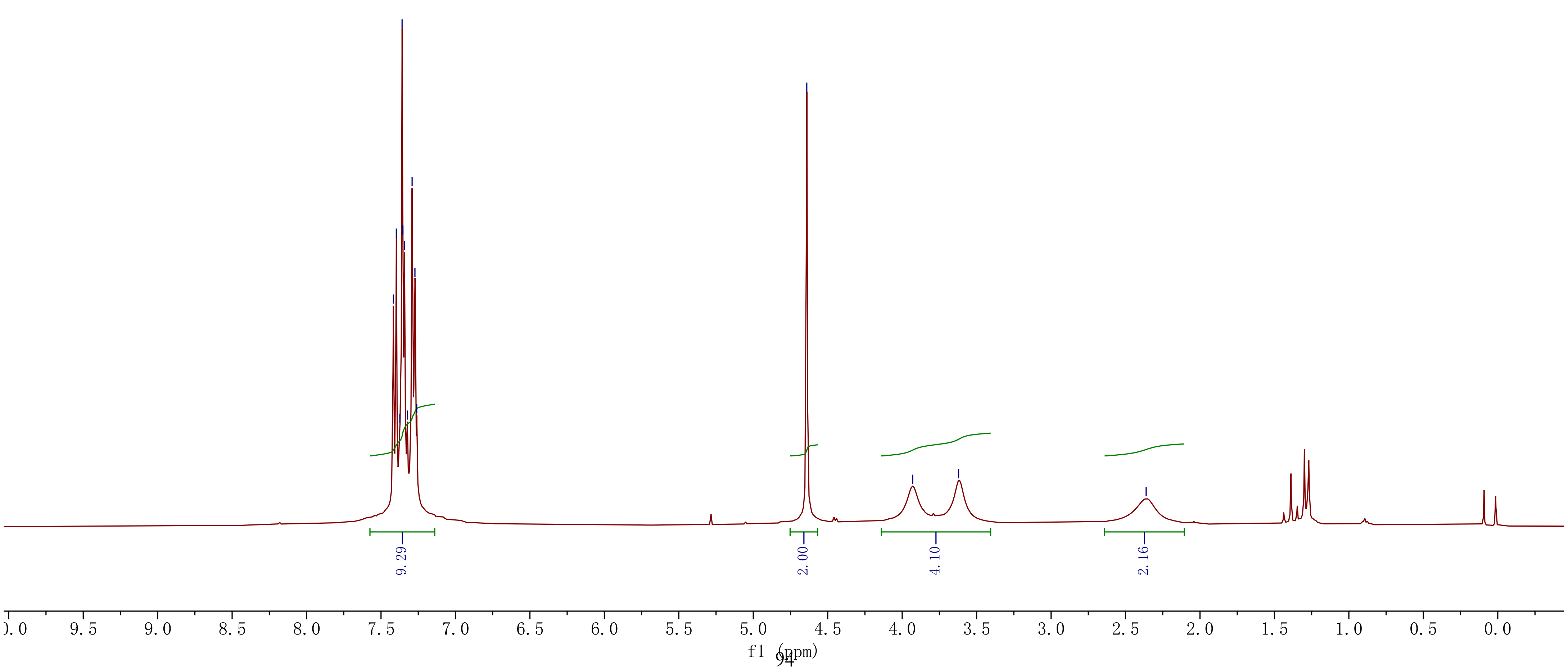

S9i, ^{13}C NMR (101 MHz, $CDCl_3$)

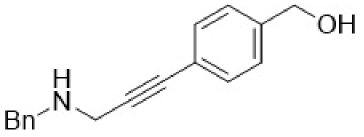

S9i

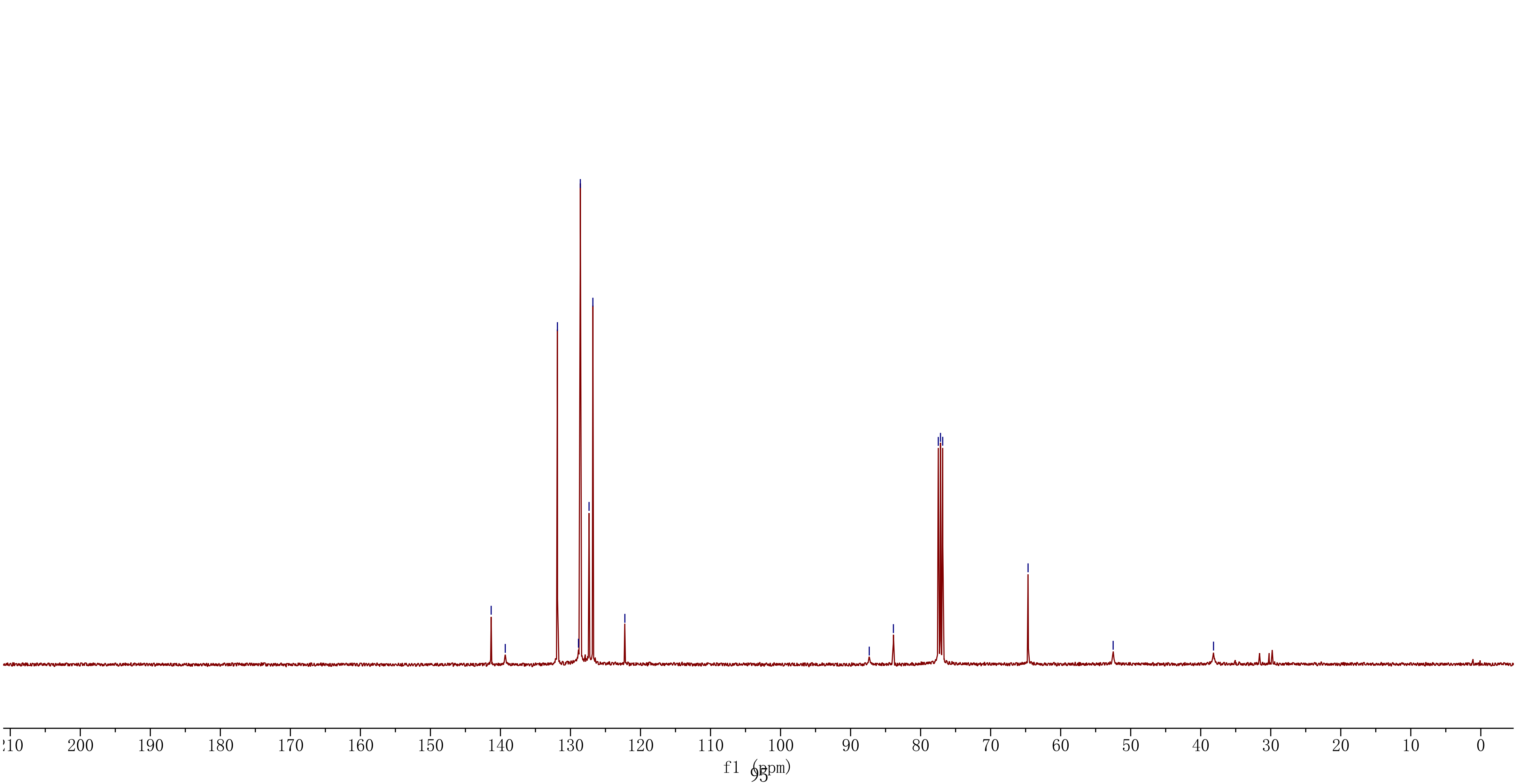

S11, ^{1}H NMR (400 MHz, $CDCl_3$)

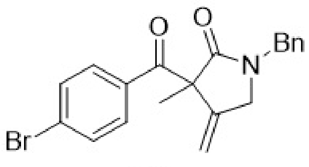

S11

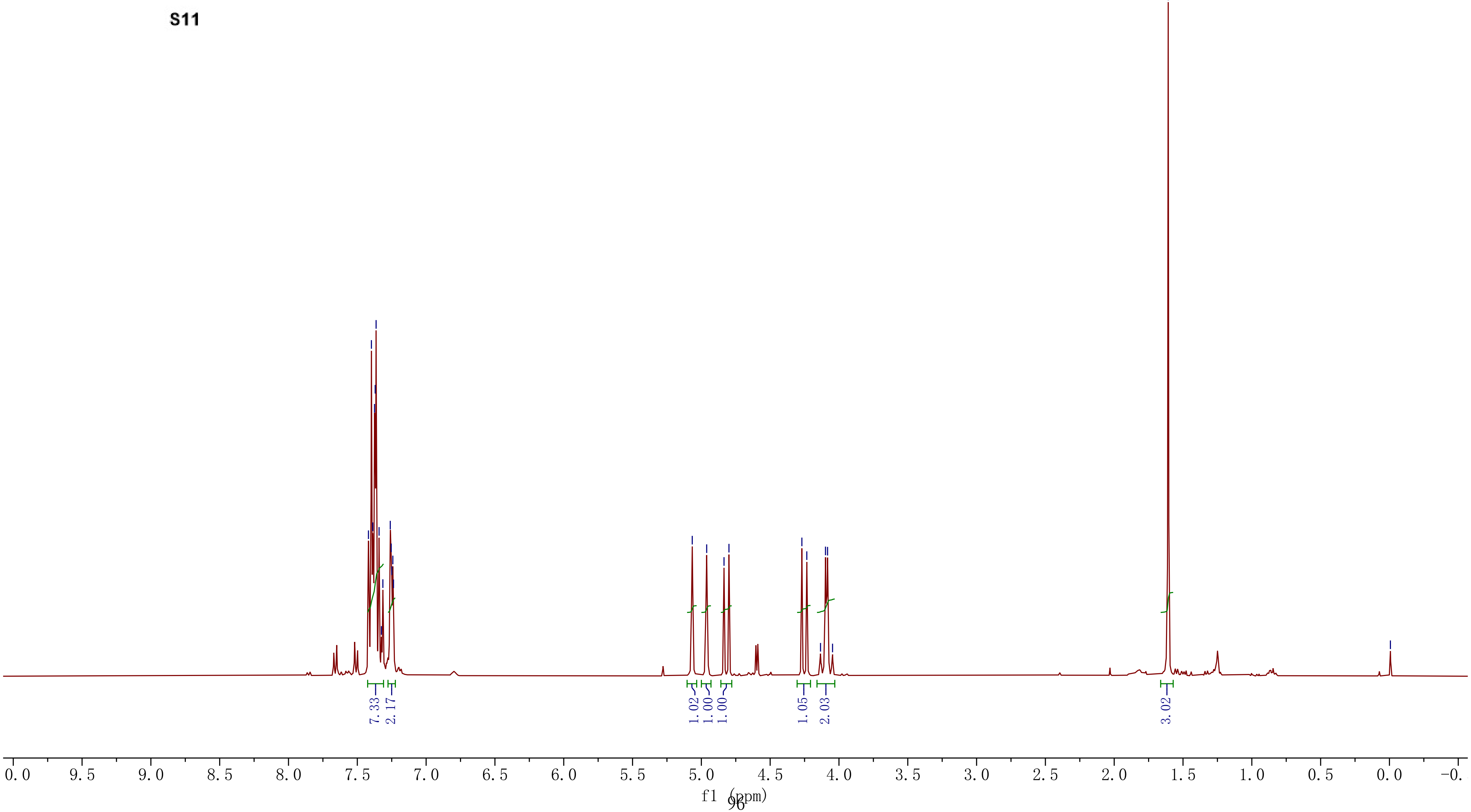

S11, ^{13}C NMR (101 MHz, $CDCl_3$)

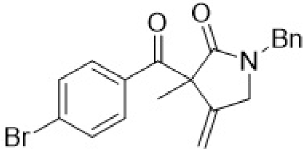

S11

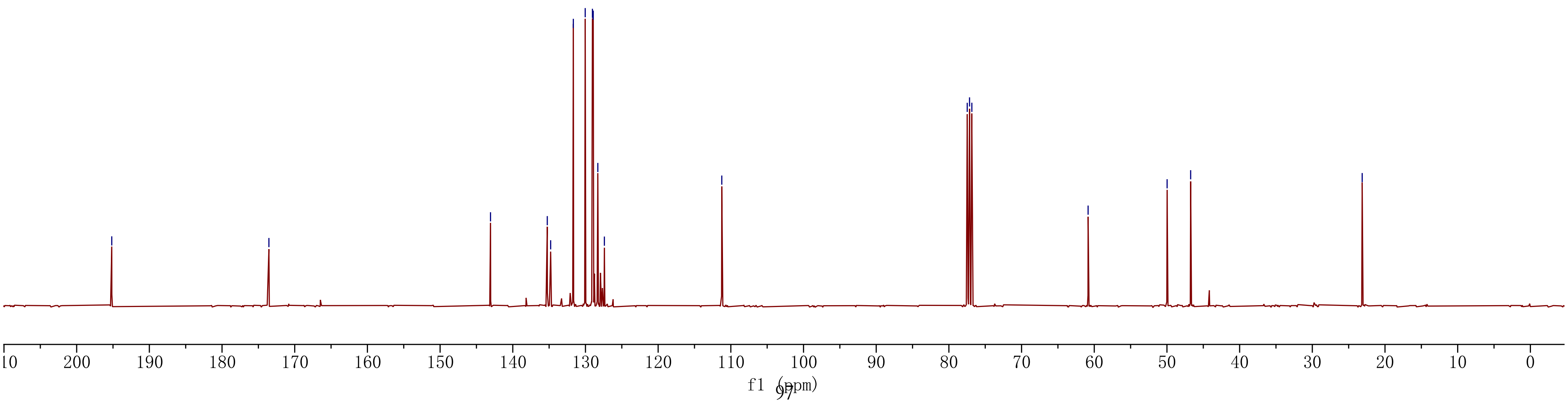

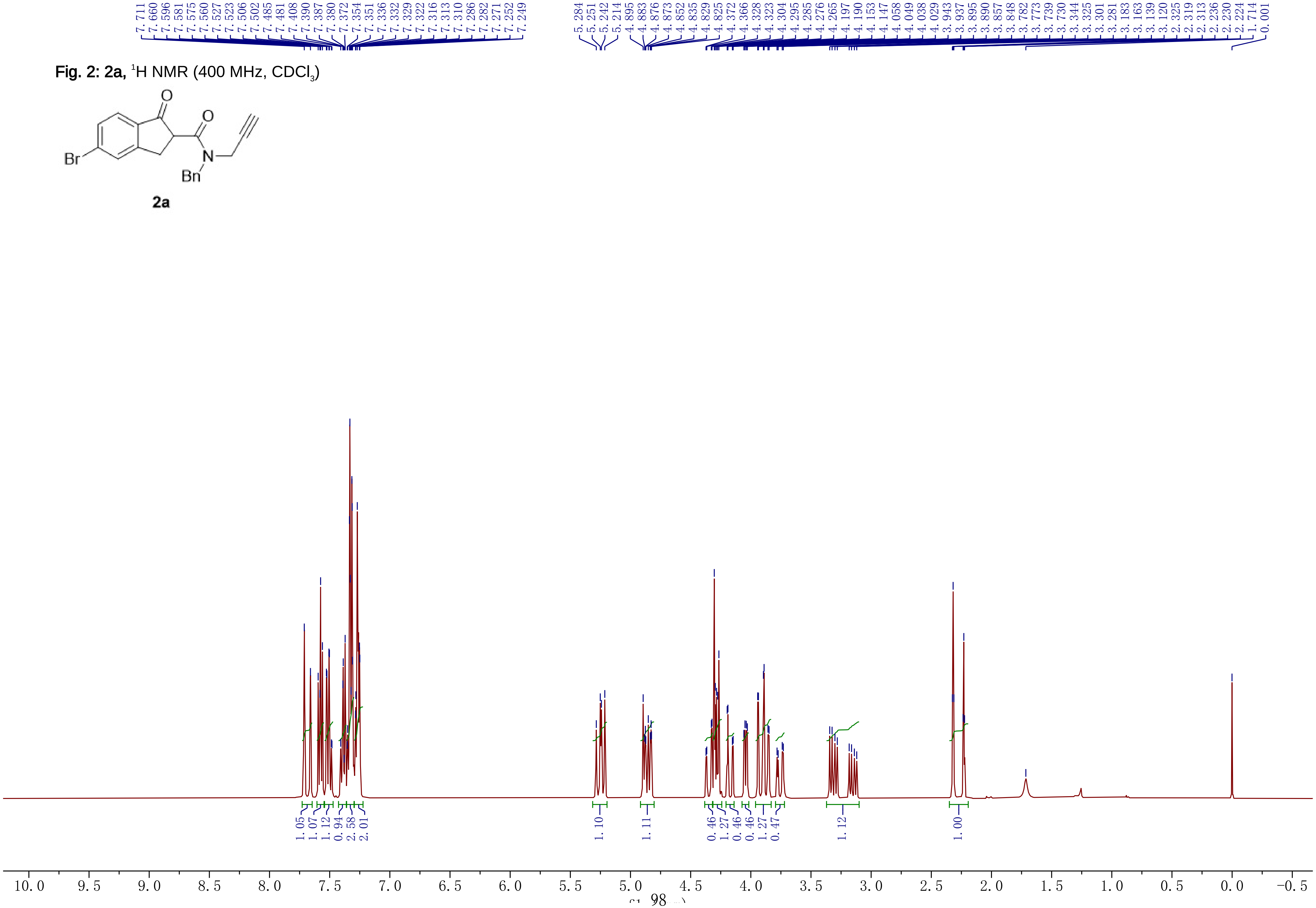

Fig. 2: 2a, ^{1}H NMR (400 MHz, $CDCl_3$)

Fig. 2: 2a, ^{13}C NMR (101 MHz, $CDCl_3$)

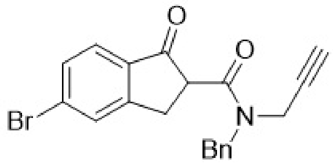

2a

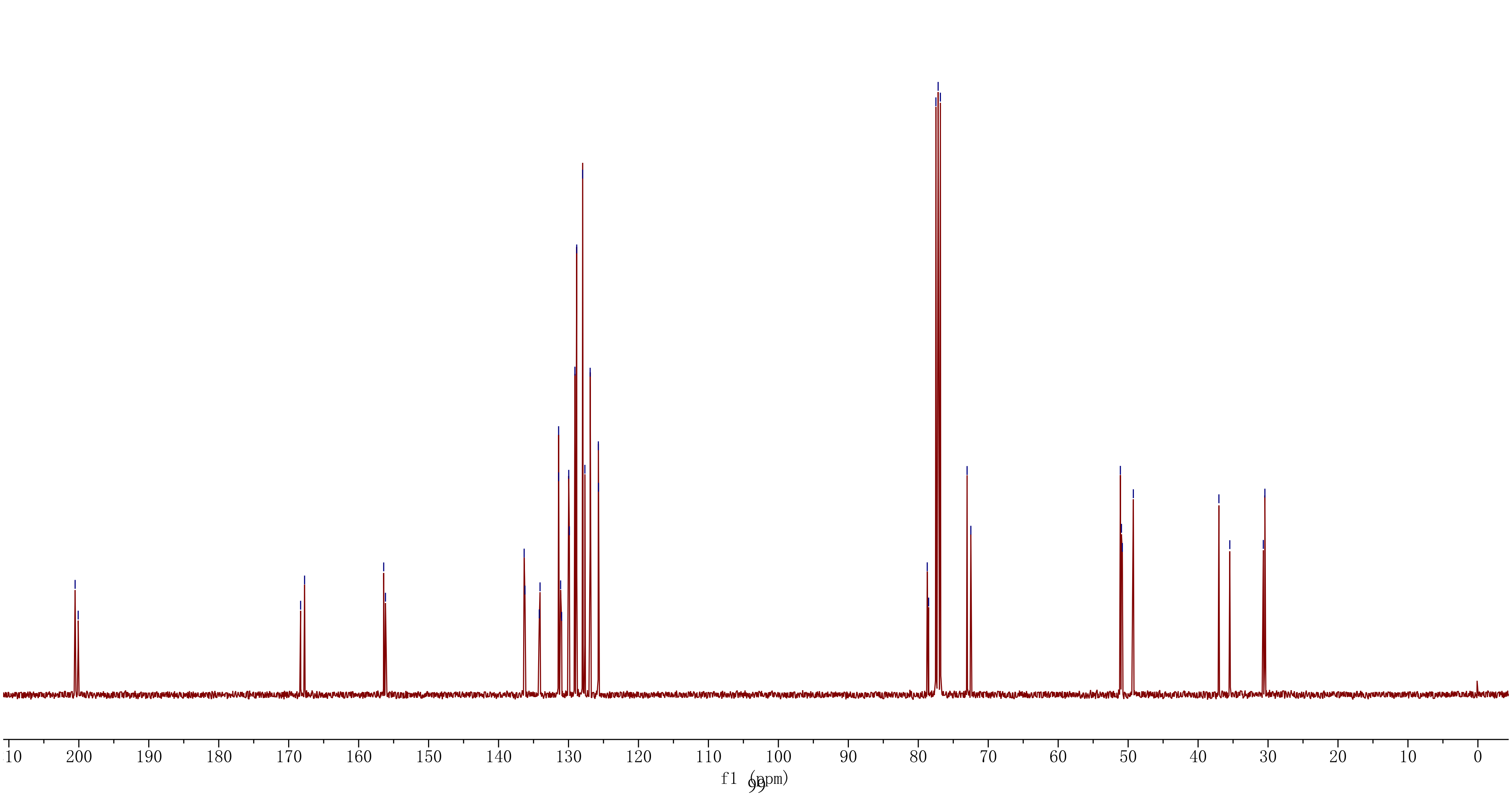

Fig. 2: 2b, ^{1}H NMR (400 MHz, $CDCl_3$)

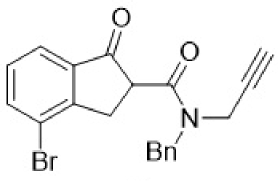

2b

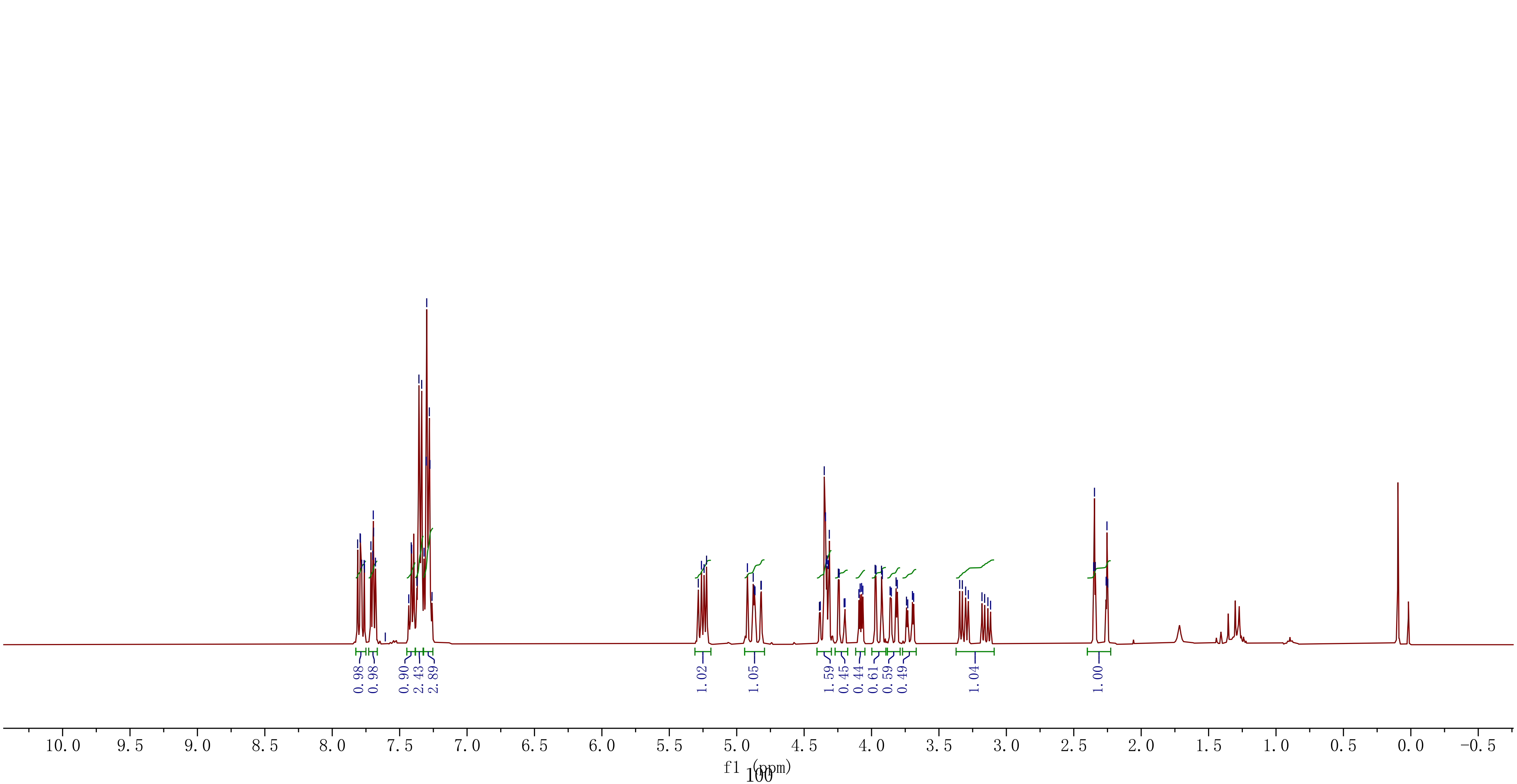

Fig. 2: 2b, ^{13}C NMR (101 MHz, $CDCl_3$)

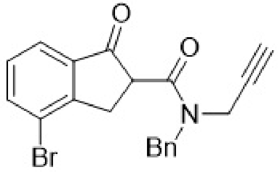

2b

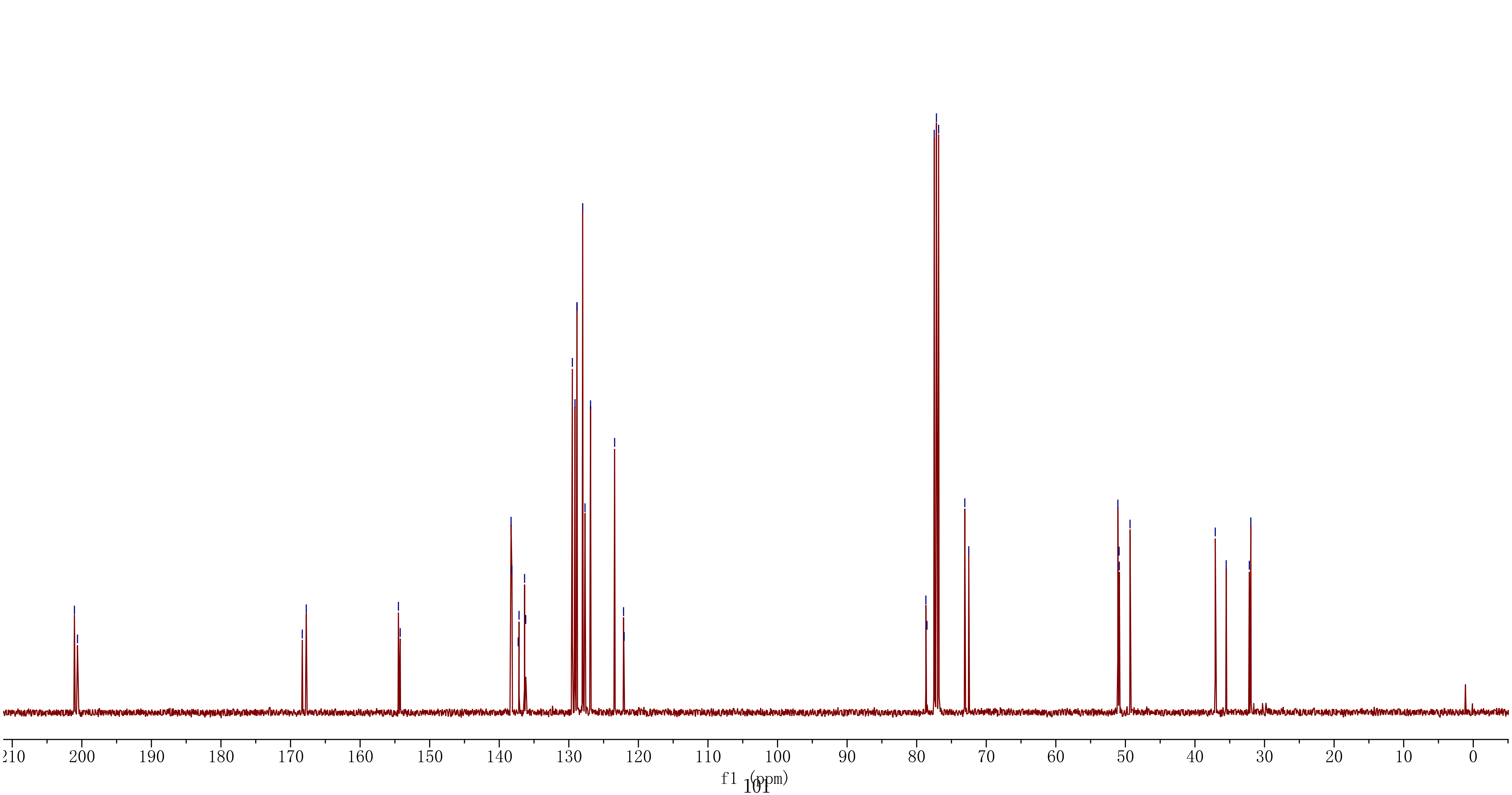

Fig. 2: 2c, ^1H NMR (400 MHz, $CDCl_3$)

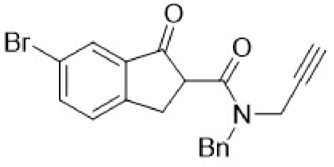

2c

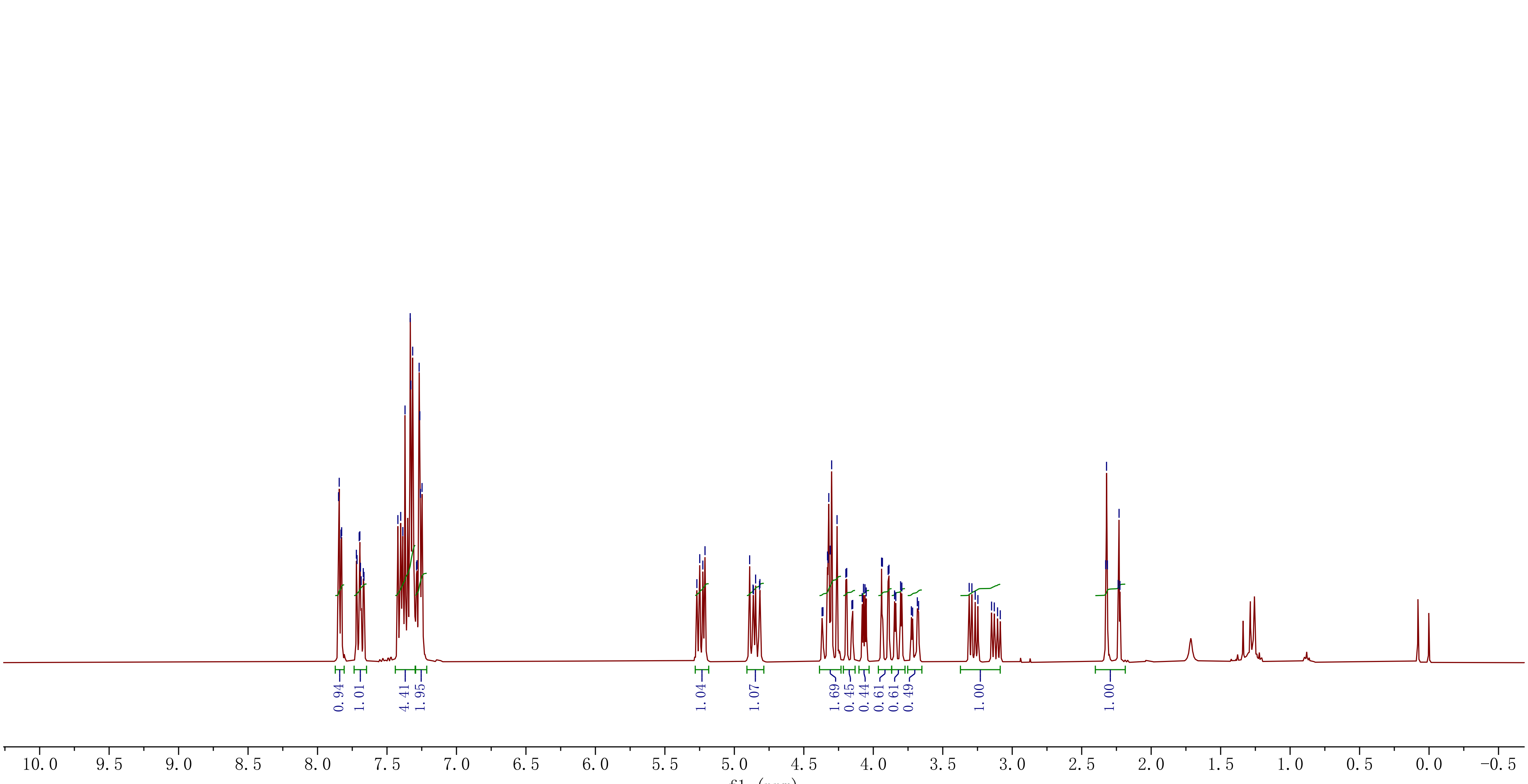

Fig. 2: 2c, ^{13}C NMR (101 MHz, $CDCl_3$)

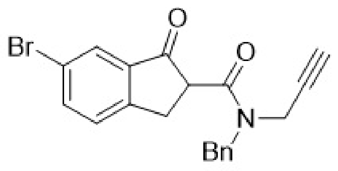

2c

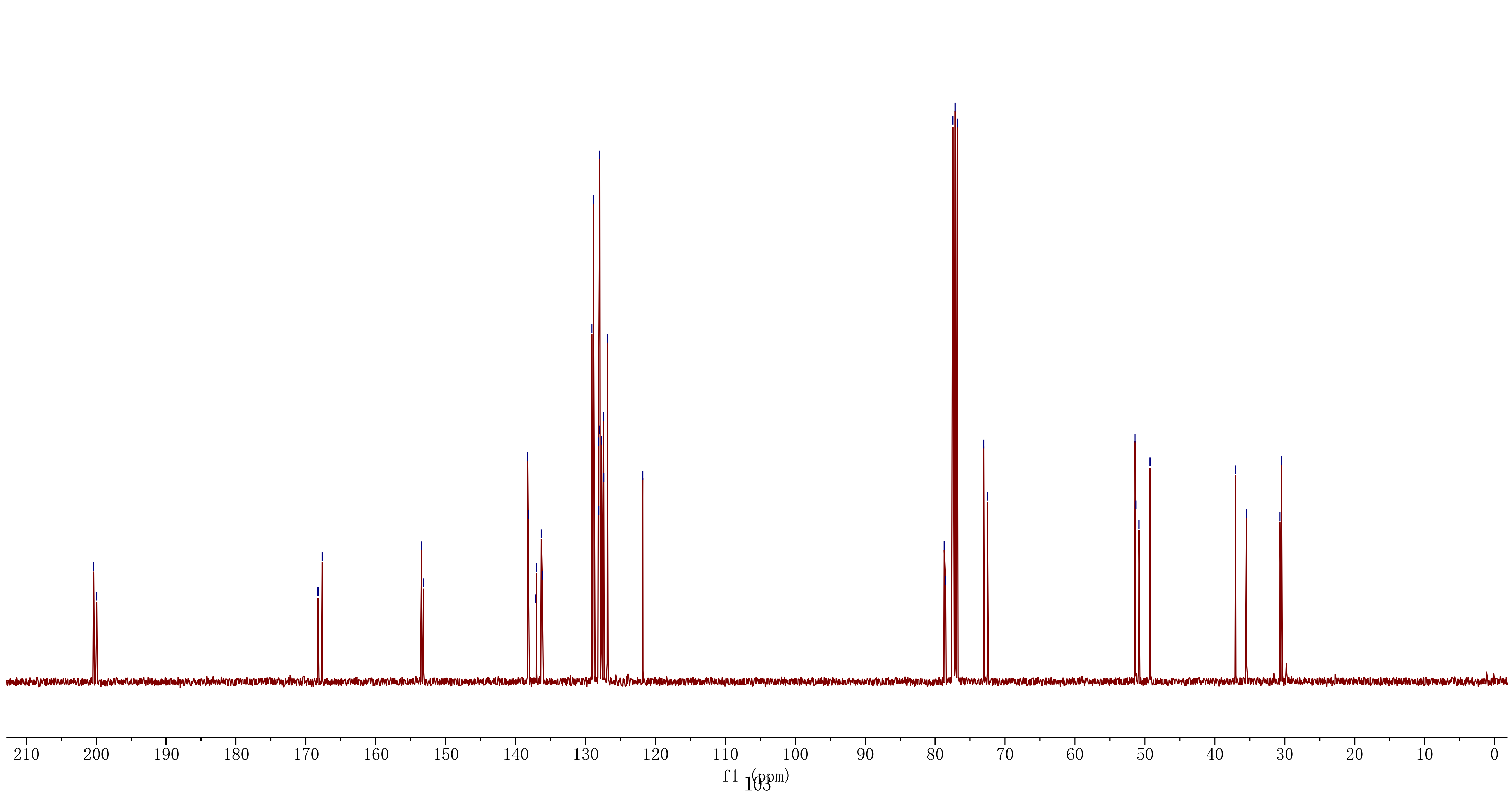

Fig. 2: 2d, ^{1}H NMR (400 MHz, $CDCl_3$)

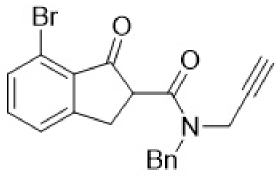

2d

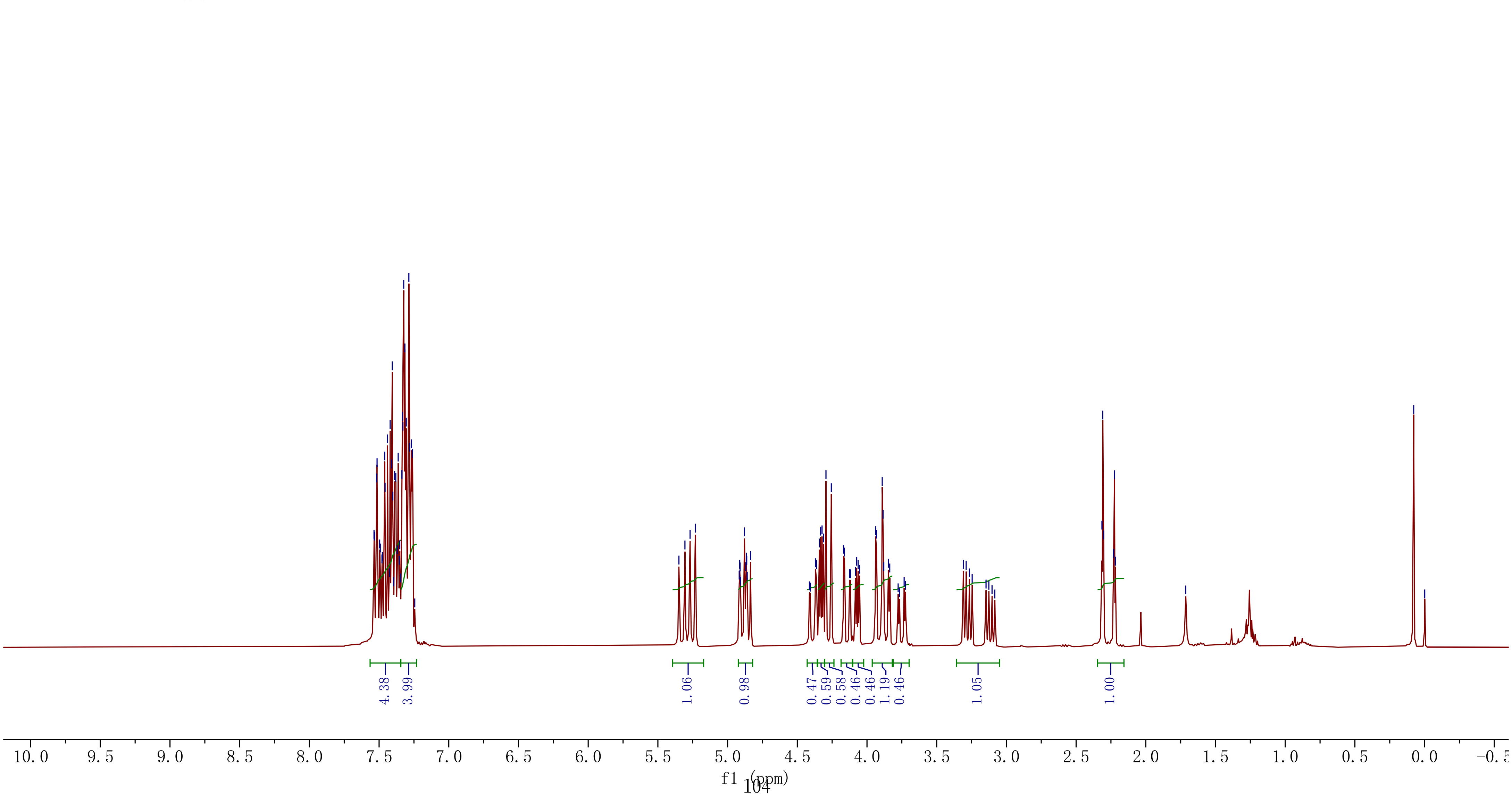

Fig. 2: 2d, ^{13}C NMR (101 MHz, $CDCl_3$)

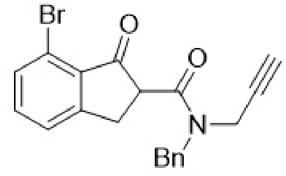

2d

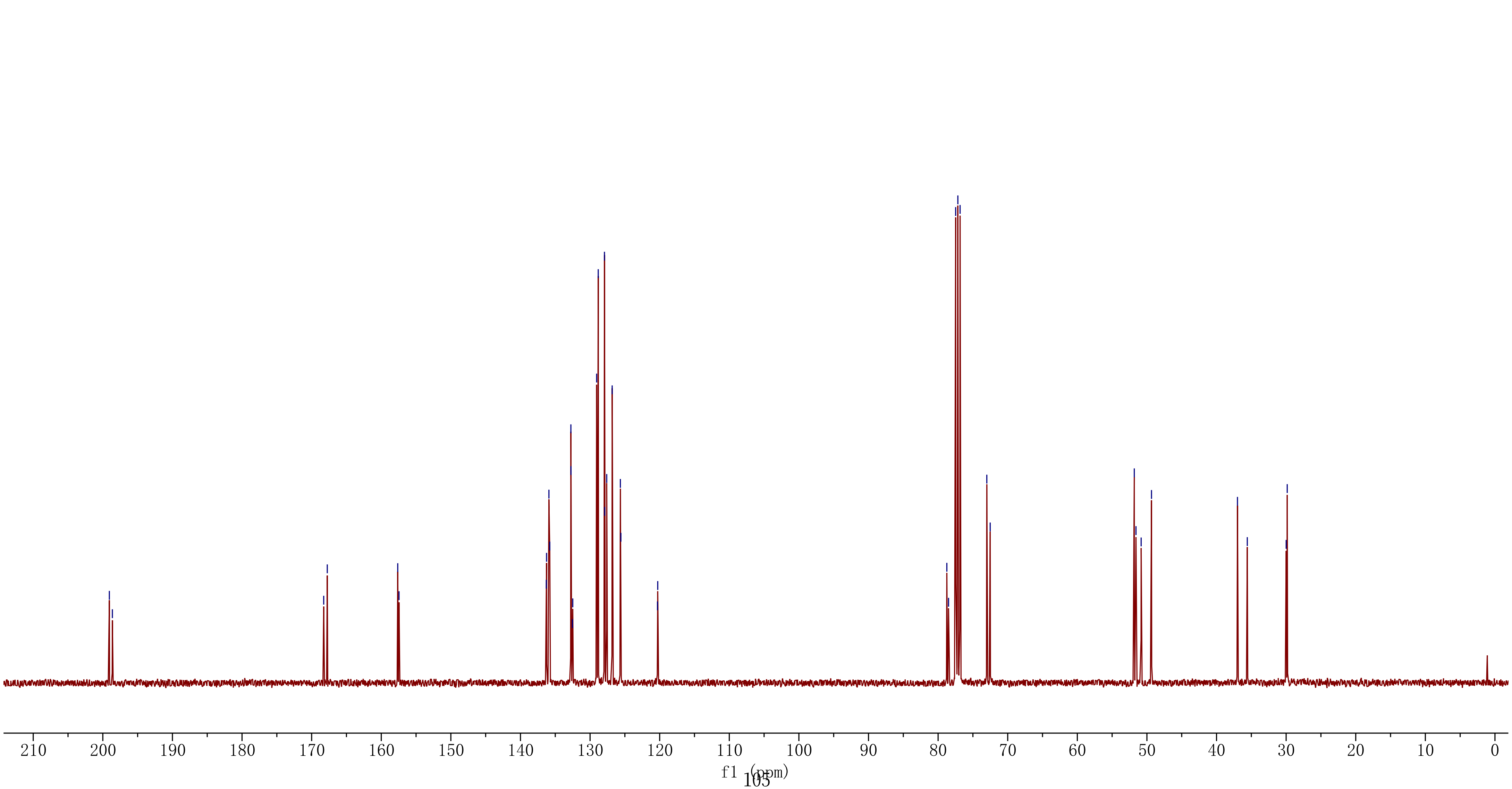

Fig. 2: 2e, ^{1}H NMR (400 MHz, $CDCl_3$)

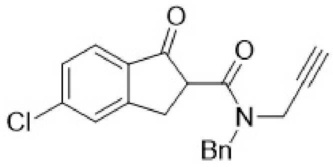

2e

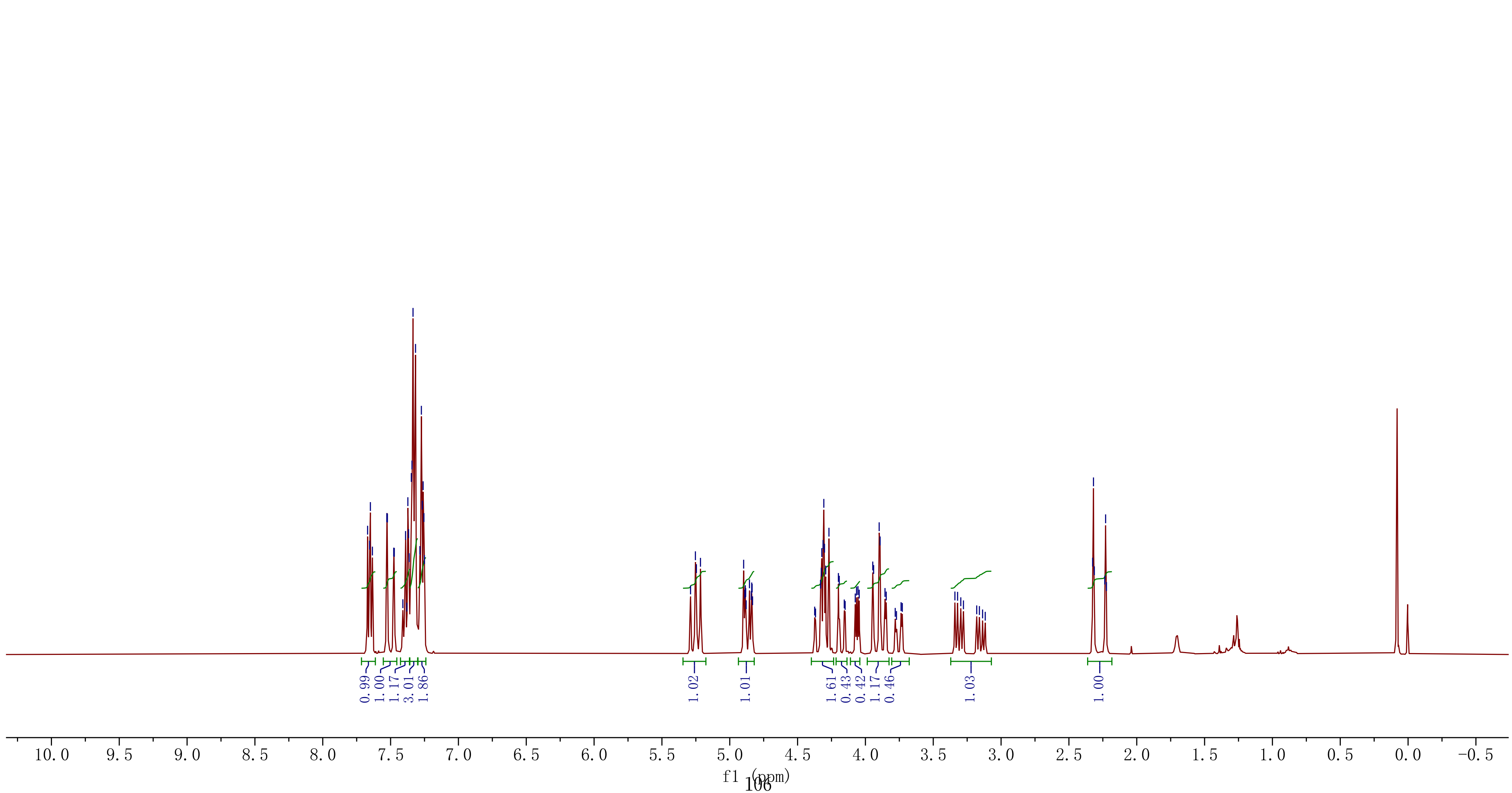

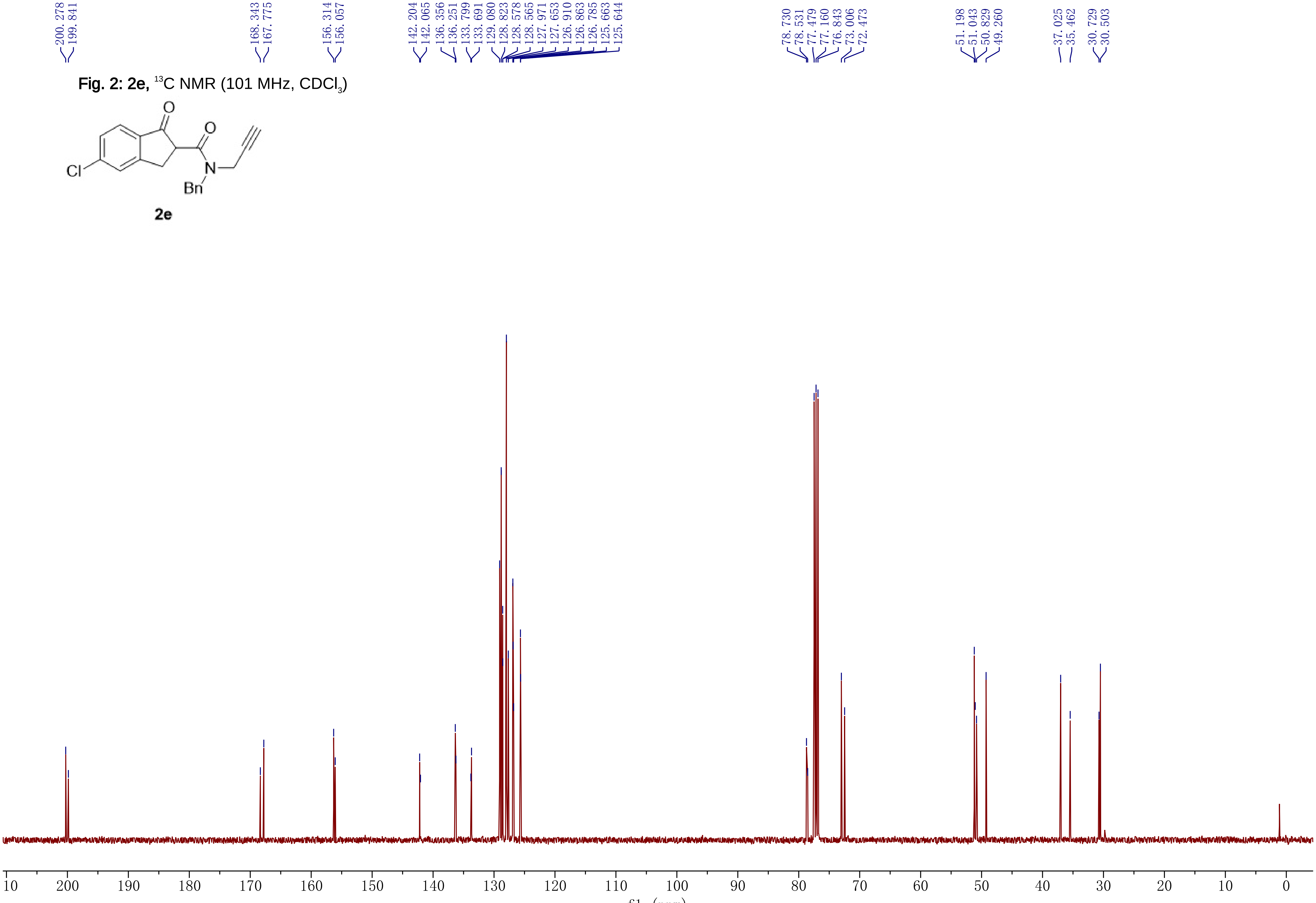

Fig. 2: 2e, ^{13}C NMR (101 MHz, $CDCl_3$)

2e

Fig. 2: 2f, ¹H NMR (400 MHz, $CDCl_3$)

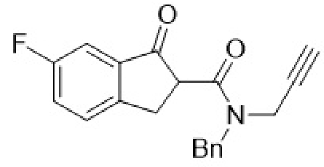

2f

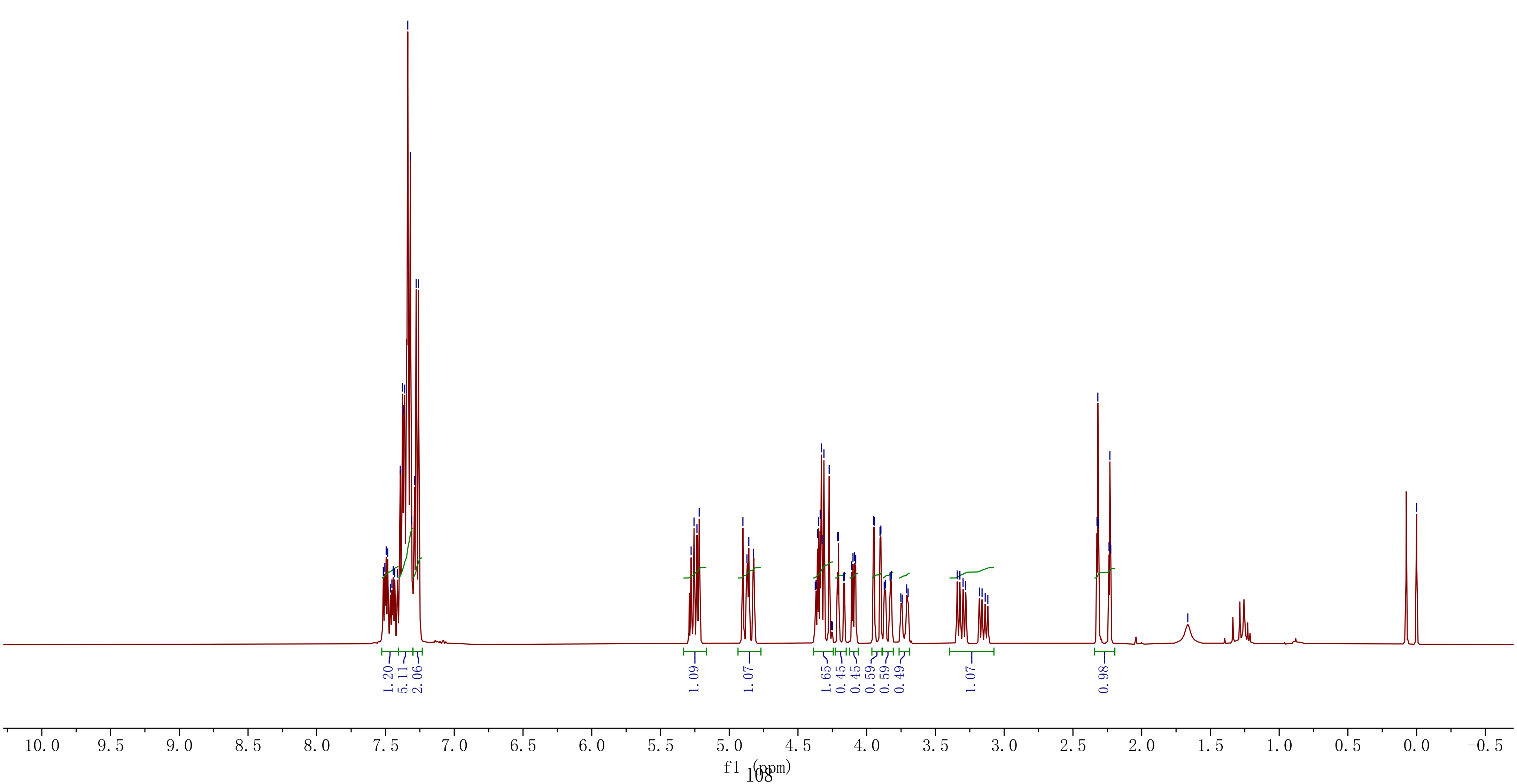

Fig. 2: 2f, ^{19}F NMR (376 MHz, $CDCl_3$)

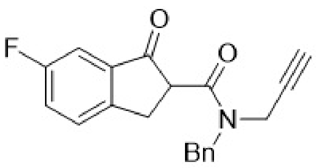

2f

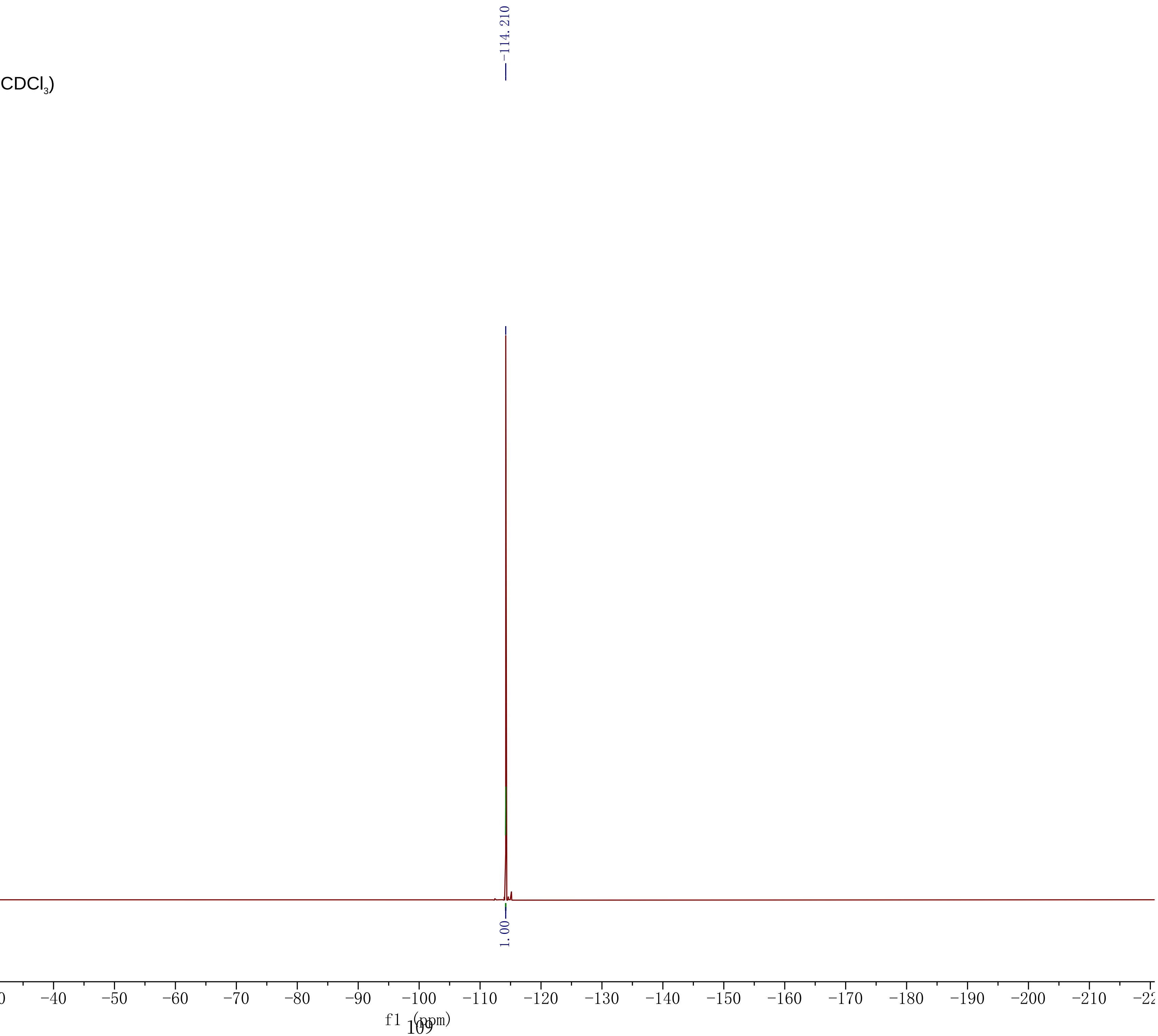

Fig. 2: 2f, ^{13}C NMR (101 MHz, $CDCl_3$)

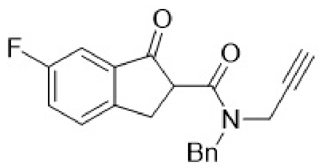

2f

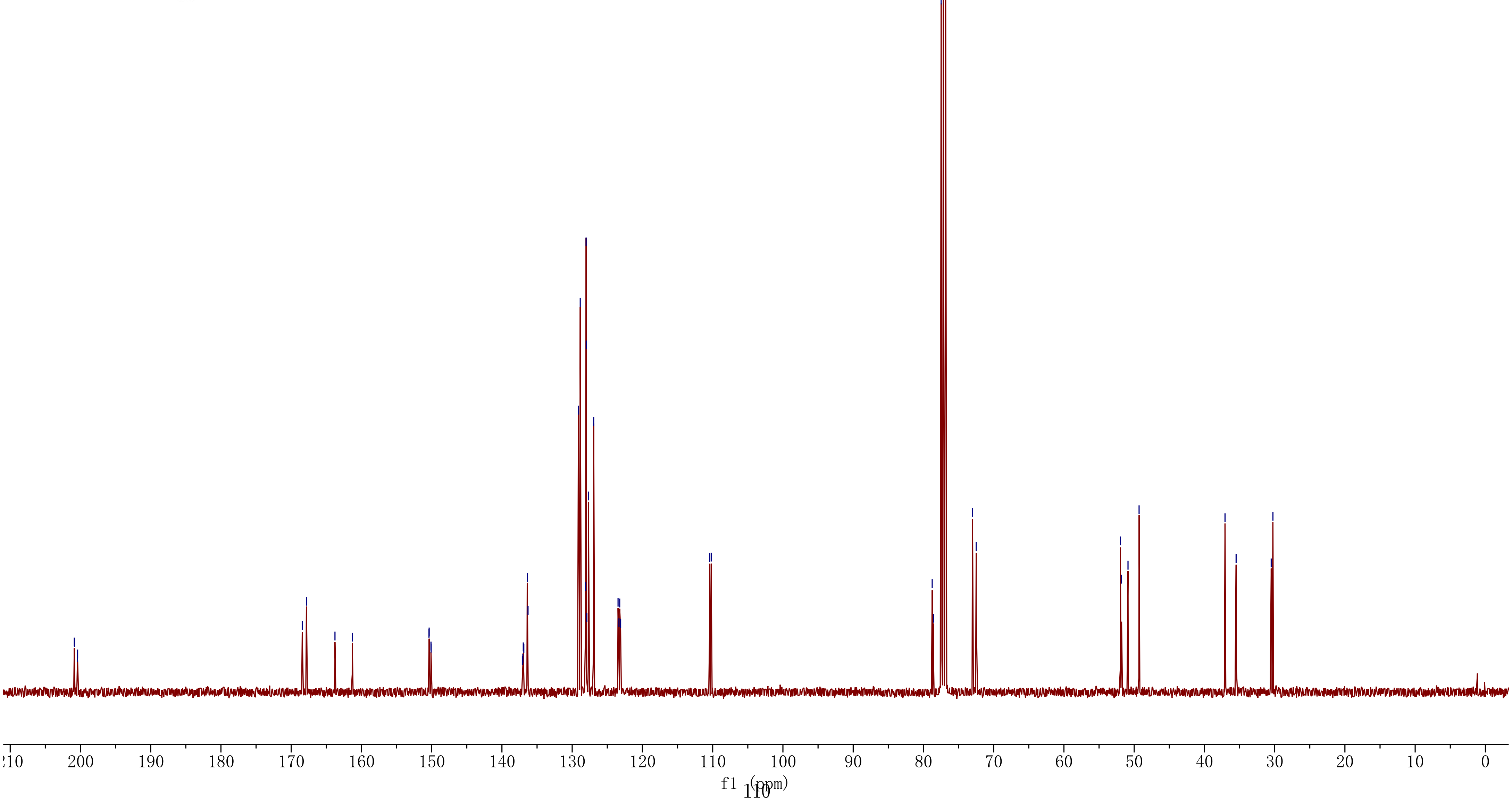

Fig. 2: 2g, ^{1}H NMR (400 MHz, $CDCl_3$)

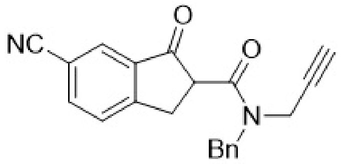

2g

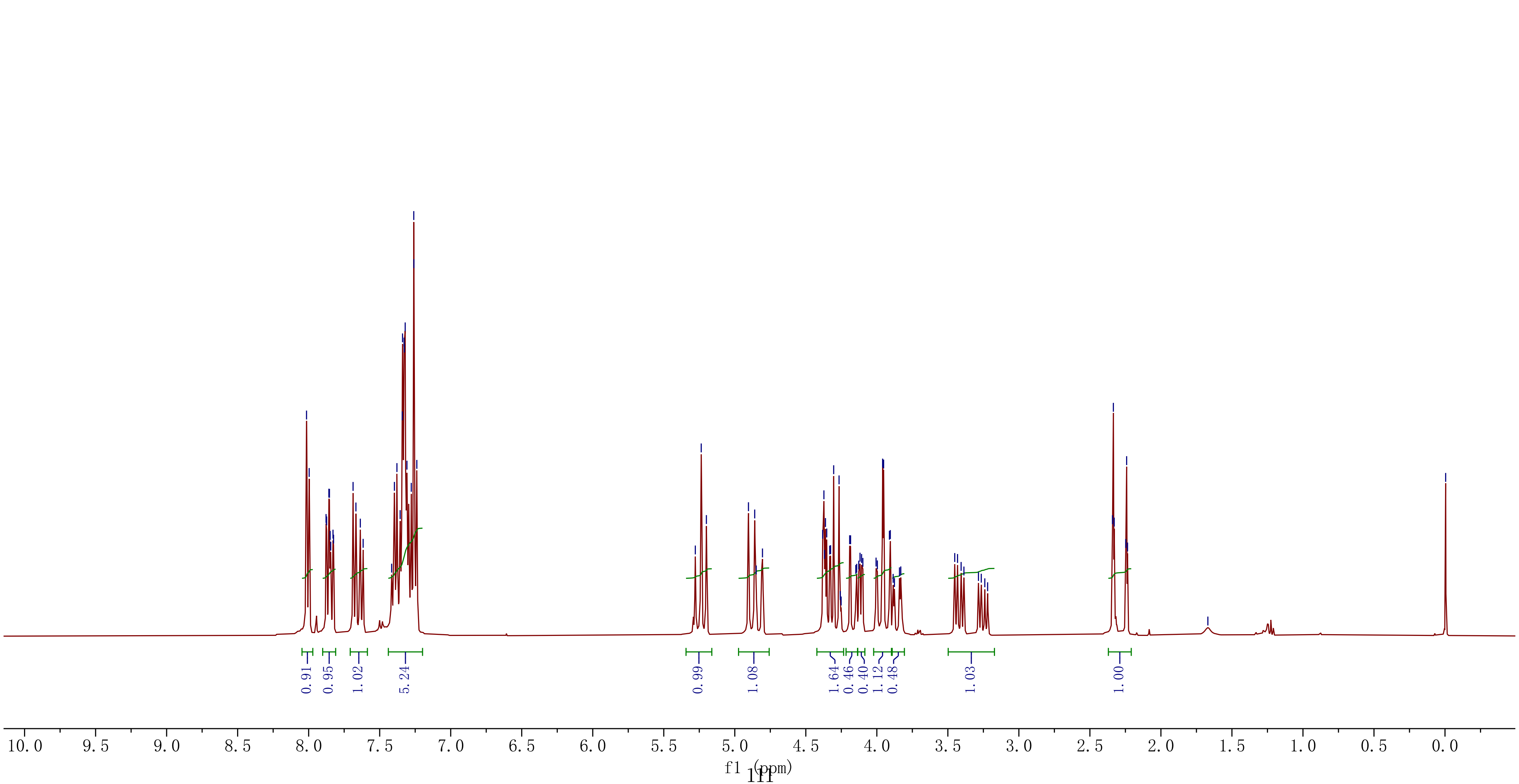

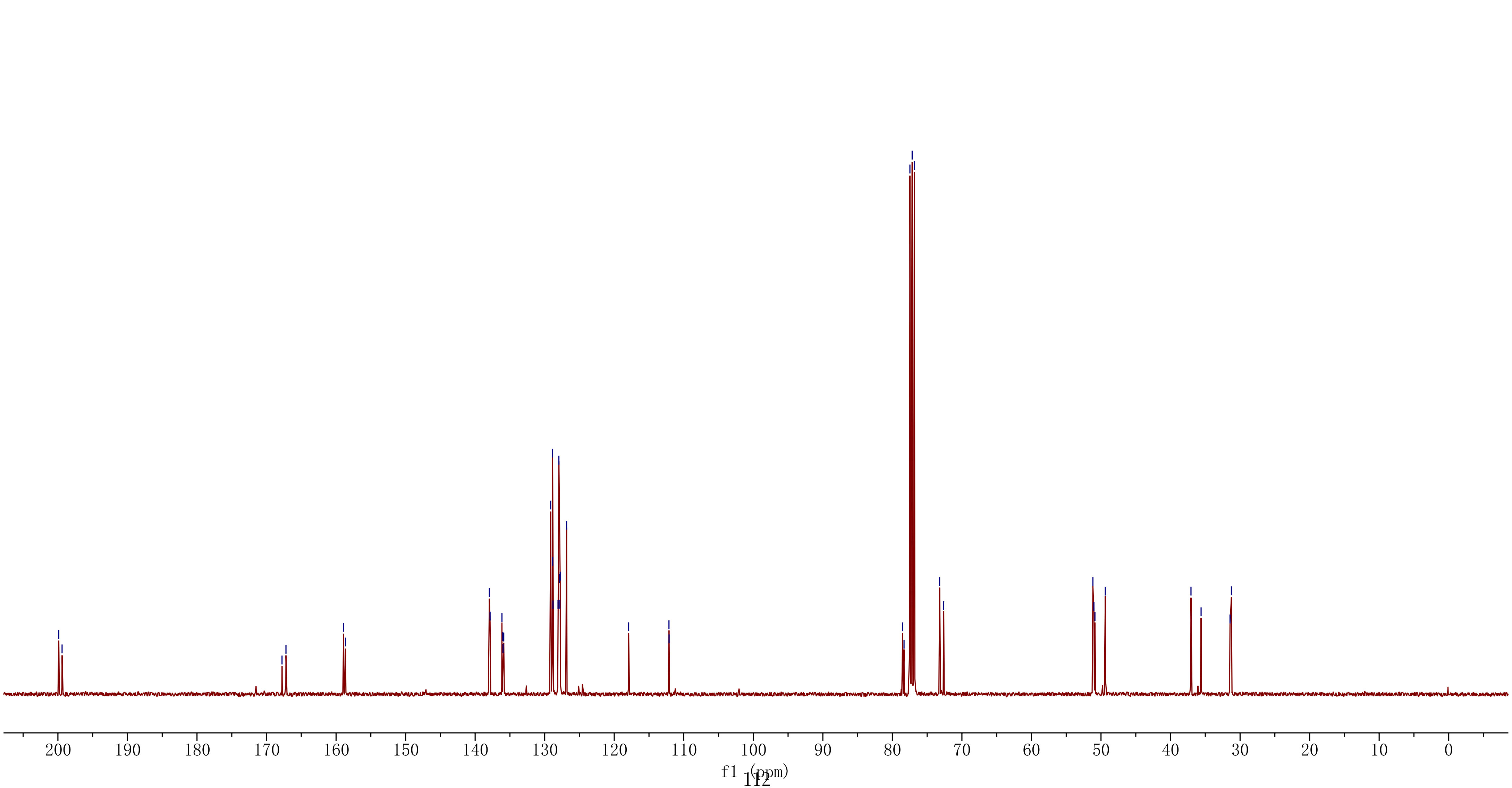

Fig. 2: 2g, ^{13}C NMR (101 MHz, $CDCl_3$)

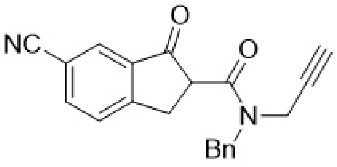

2g

Fig. 2: 2h, ^{1}H NMR (400 MHz, $CDCl_3$)

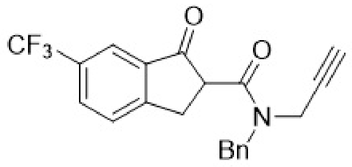

2h

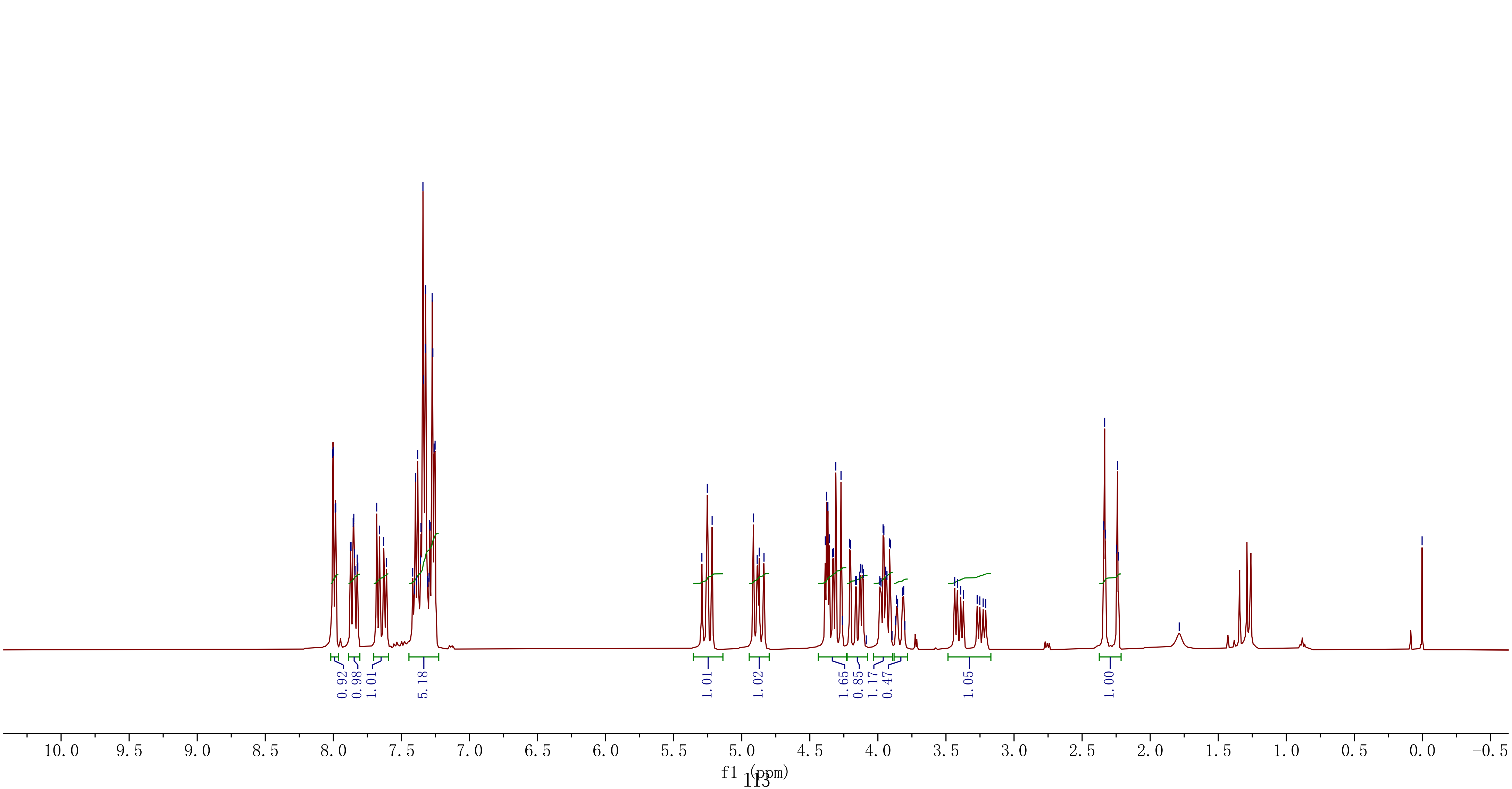

Fig. 2: 2h, ^{19}F NMR (376 MHz, $CDCl_3$)

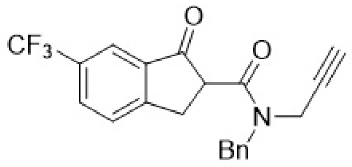

2h

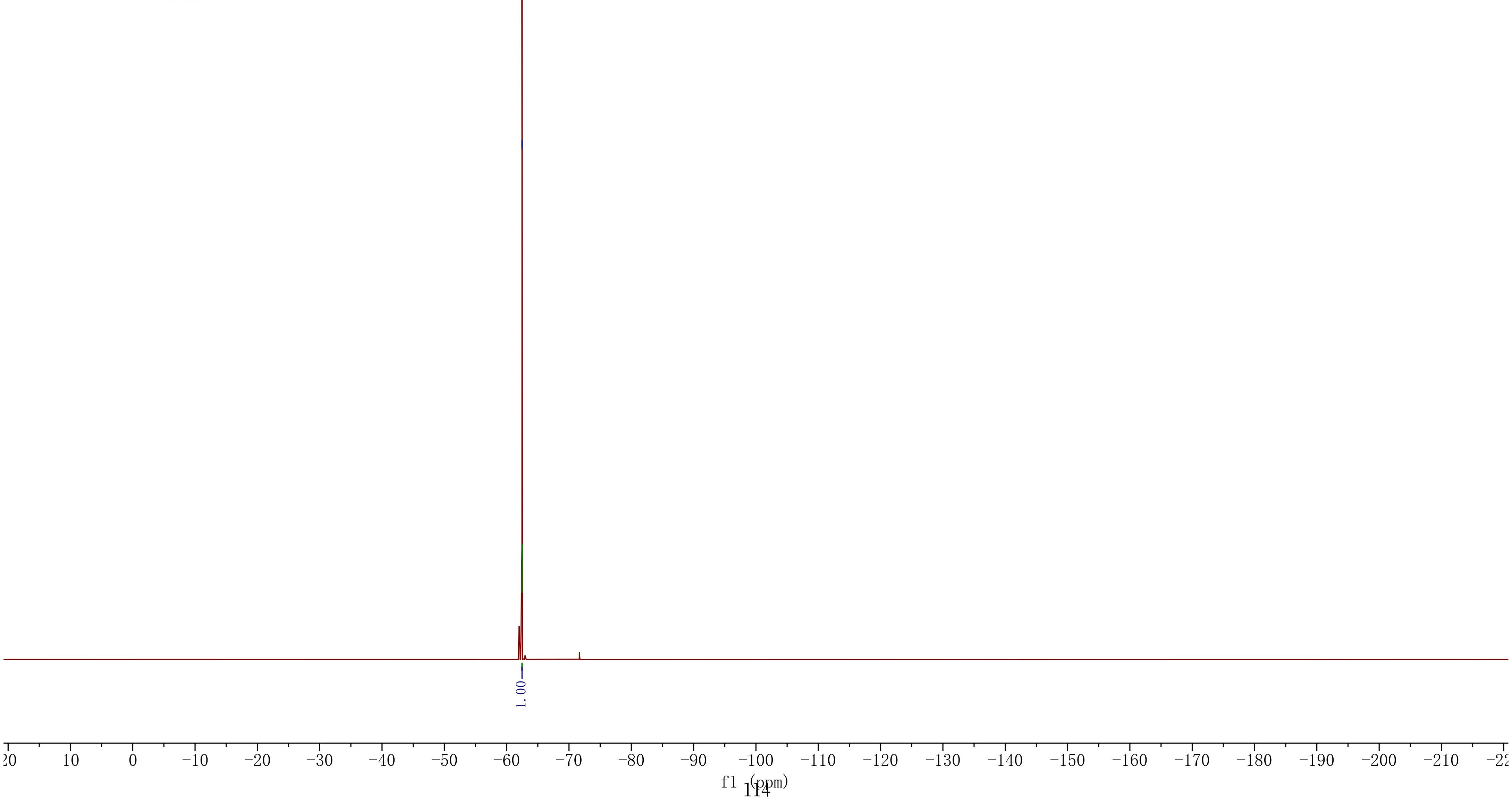

Fig. 2: 2h, ^{13}C NMR (101 MHz, $CDCl_3$)

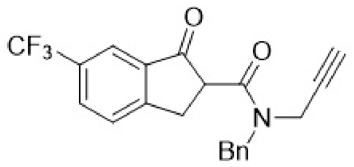

2h

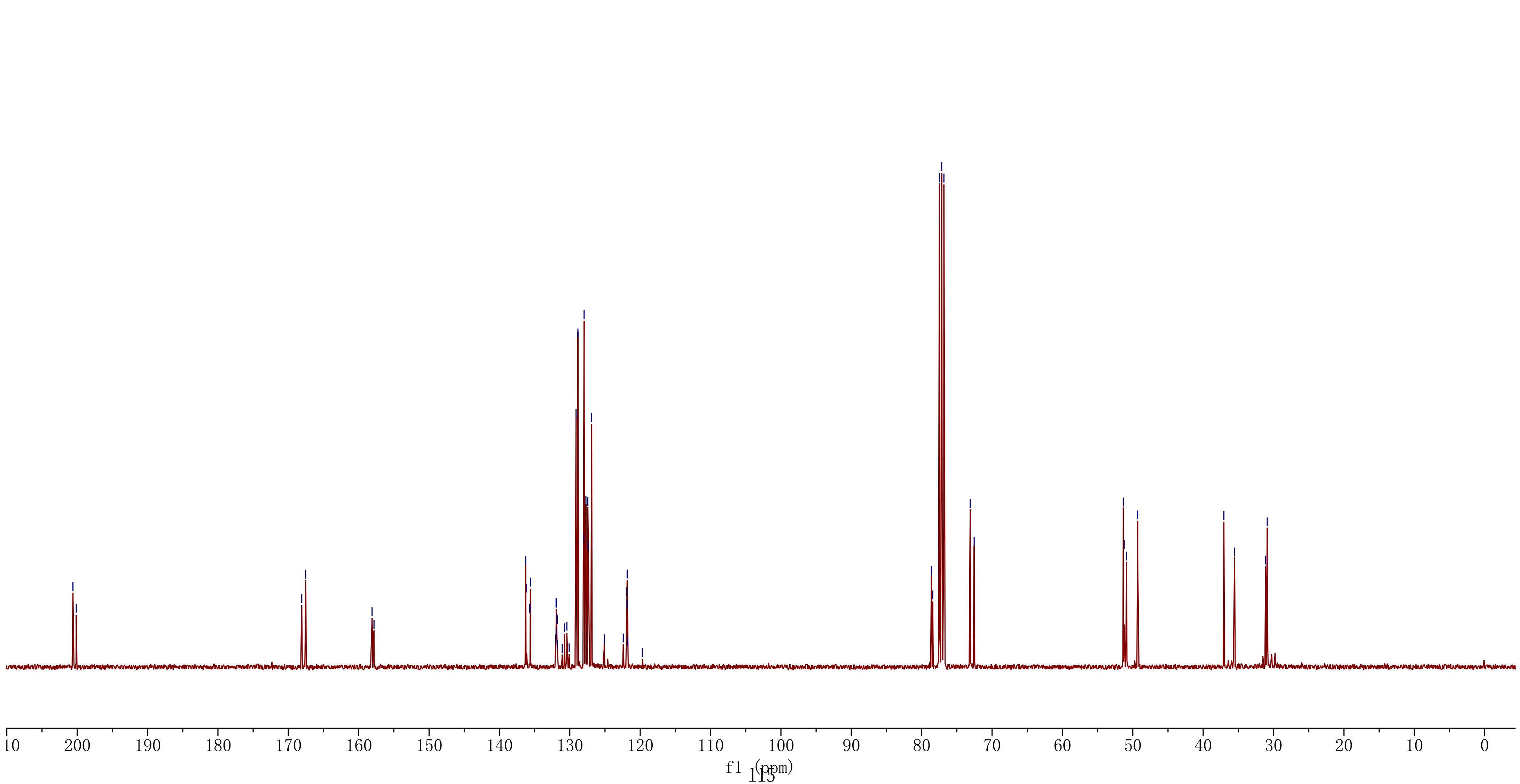

Fig. 2: 2i, ^{1}H NMR (400 MHz, $CDCl_3$)

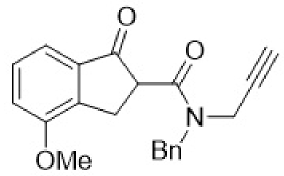

2i

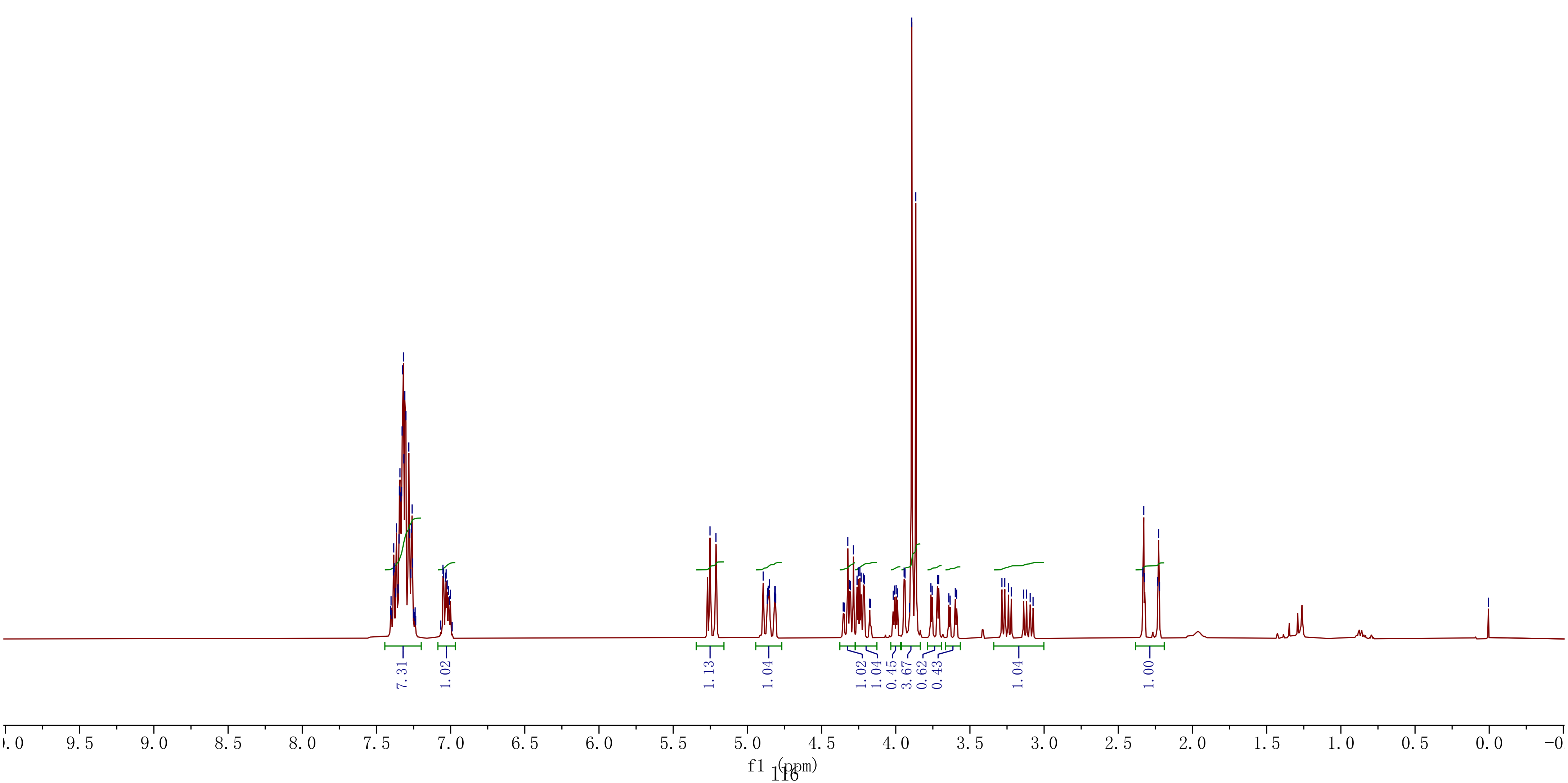

Fig. 2: 2i, ^{13}C NMR (101 MHz, $CDCl_3$)

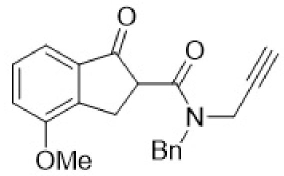

2i

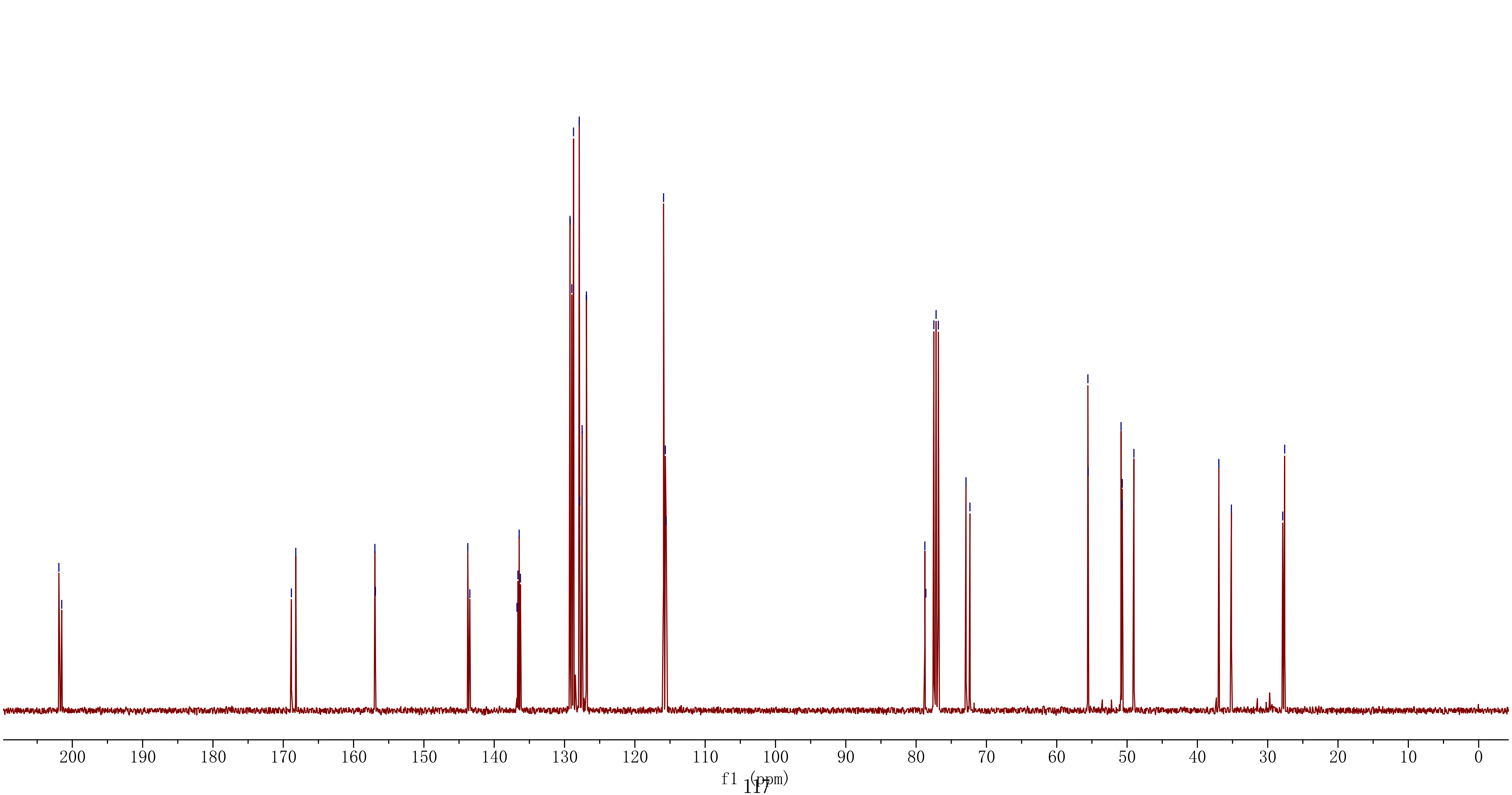

Fig. 2: 2j, ^{1}H NMR (400 MHz, $CDCl_3$)

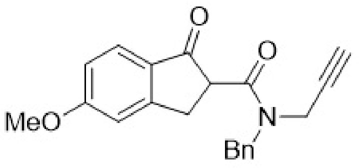

2j

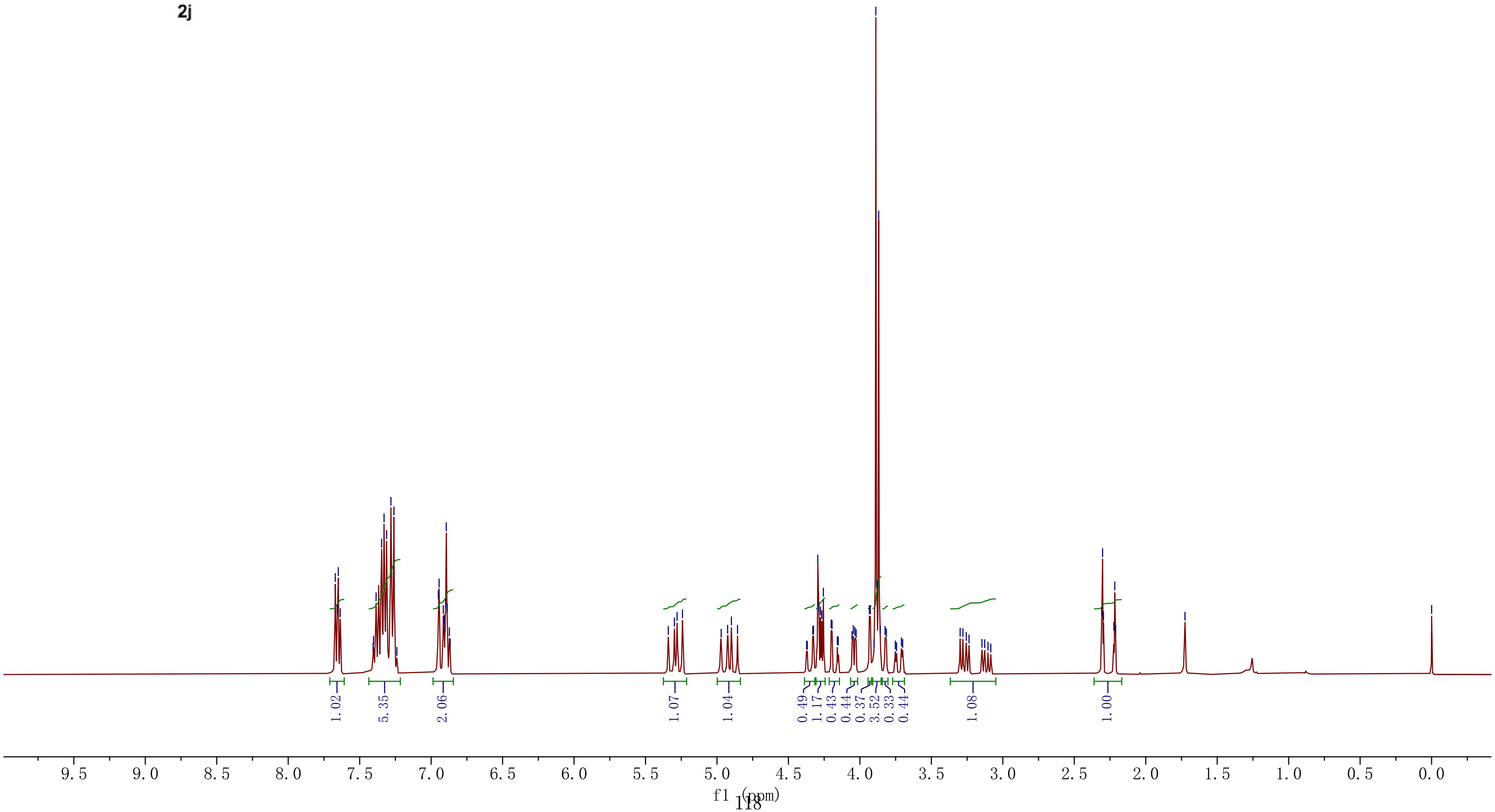

Fig. 2: 2j, ^{13}C NMR (101 MHz, $CDCl_3$)

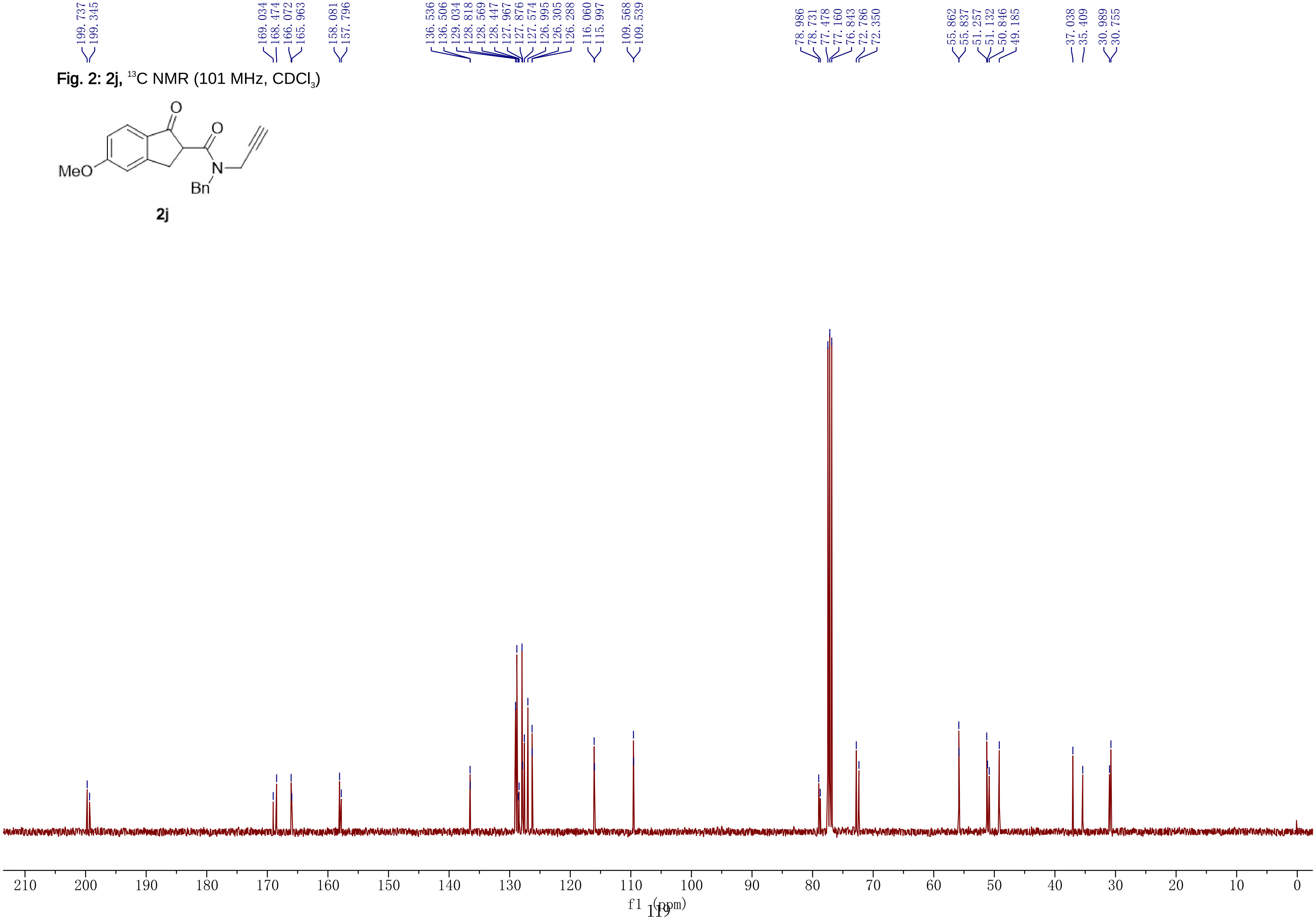

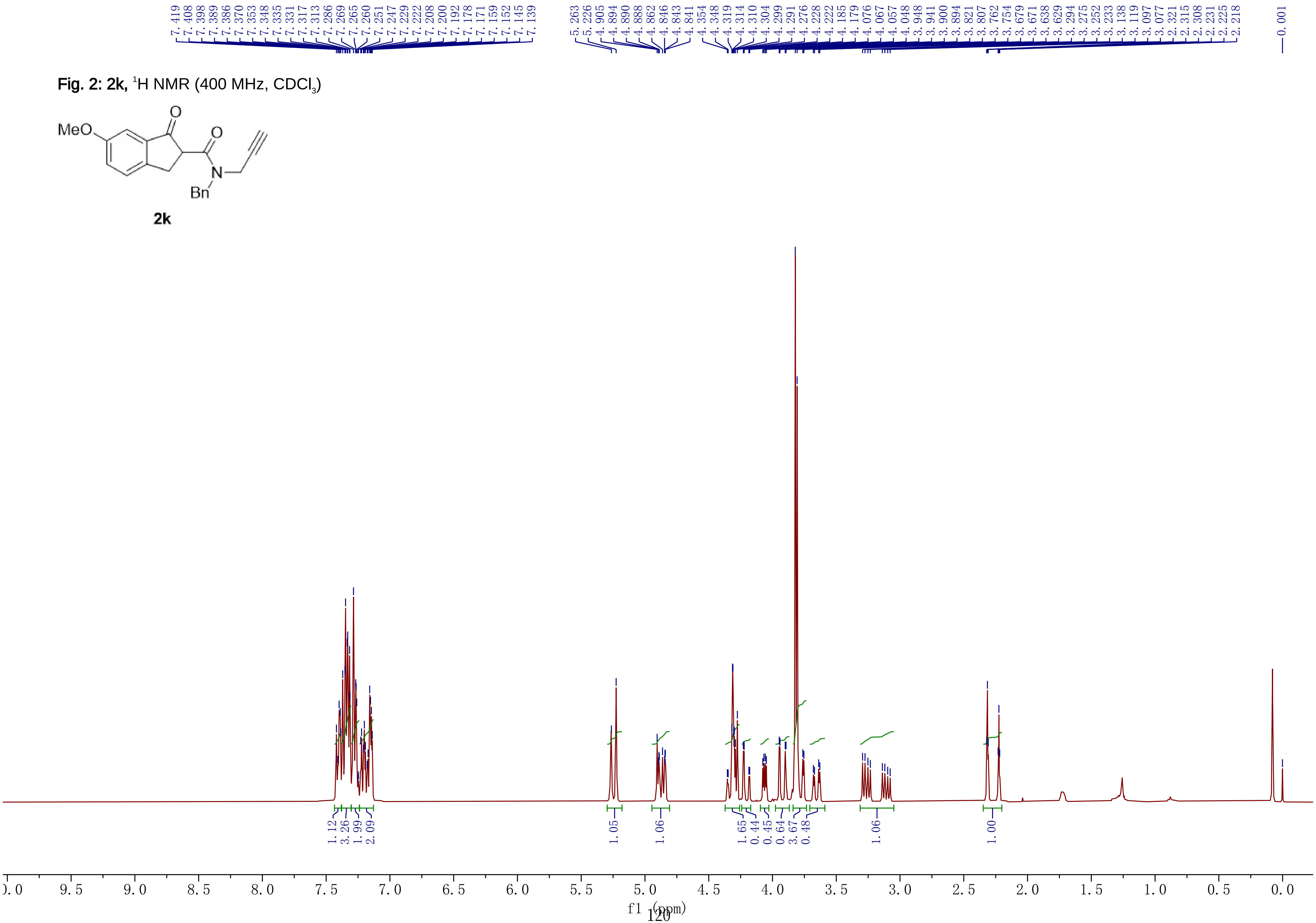

Fig. 2: 2k, ^{1}H NMR (400 MHz, $CDCl_3$)

Fig. 2: 2k, ^{13}C NMR (101 MHz, $CDCl_3$)

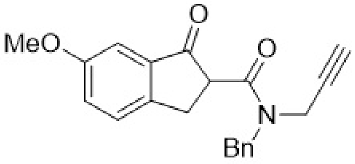

2k

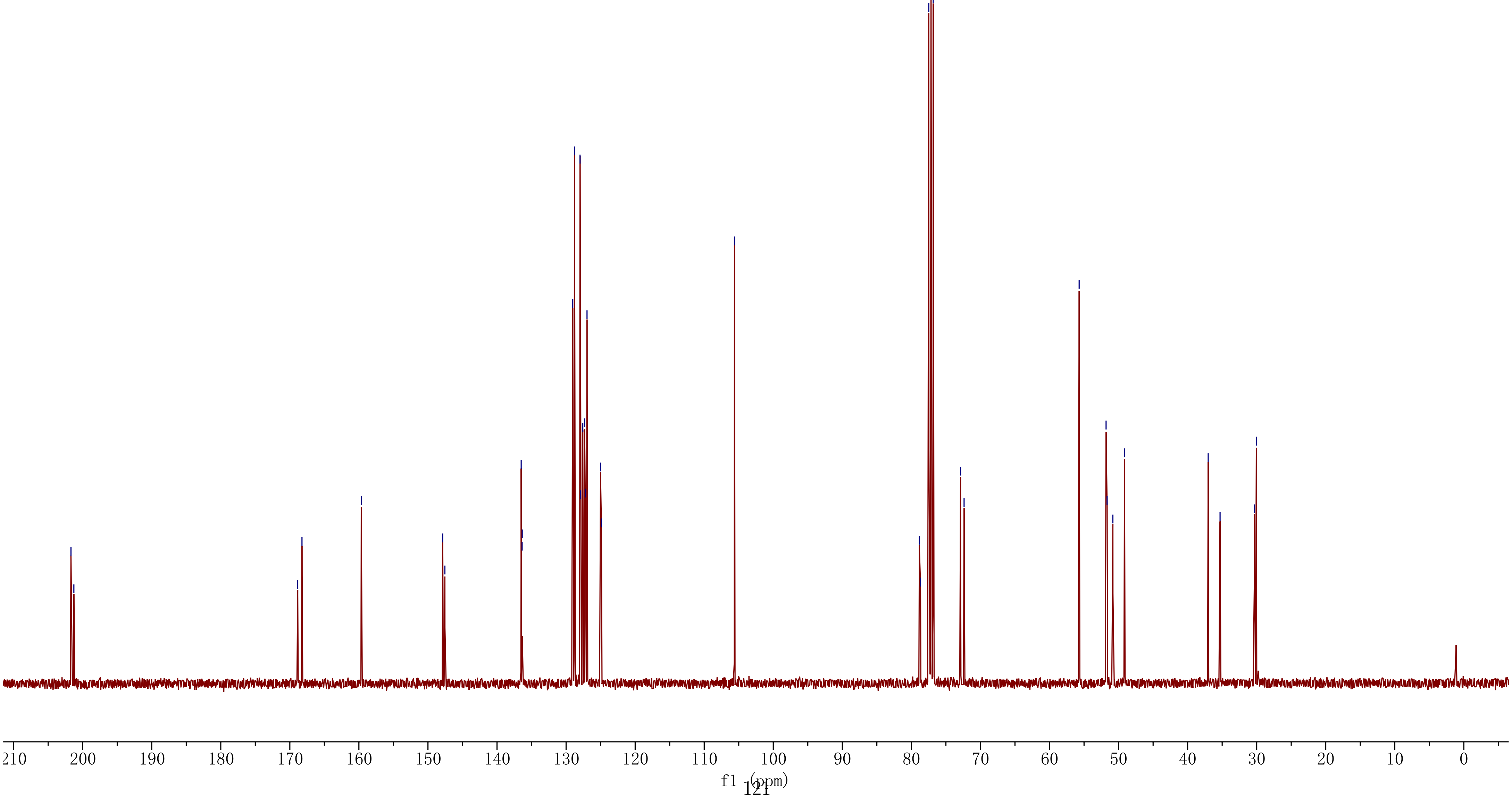

Fig. 2: 2l, ^{1}H NMR (400 MHz, $CDCl_3$)

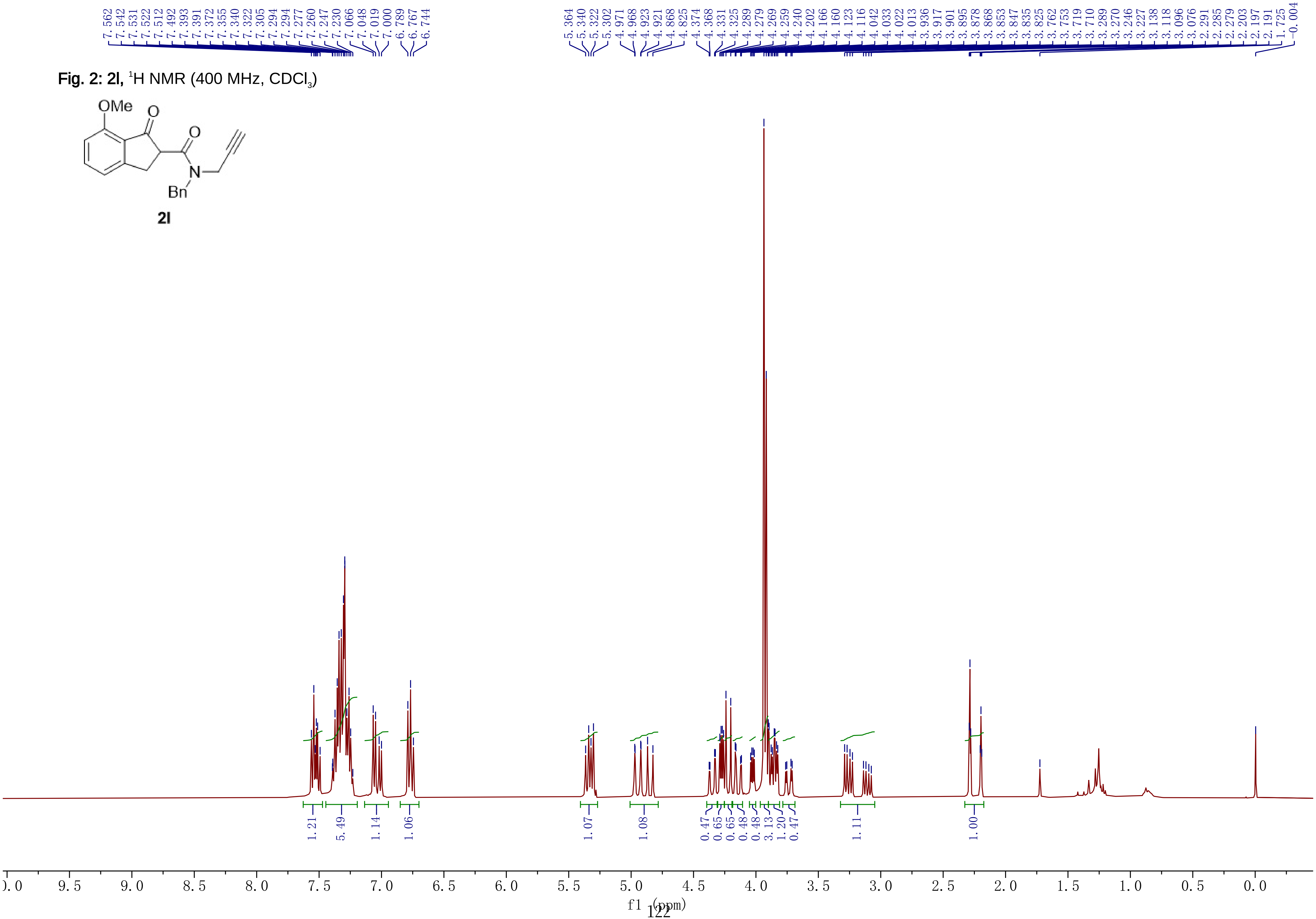

Fig. 2: 2l, ^{13}C NMR (101 MHz, $CDCl_3$)

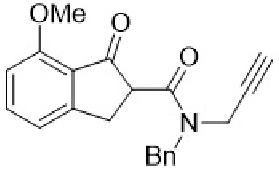

2l

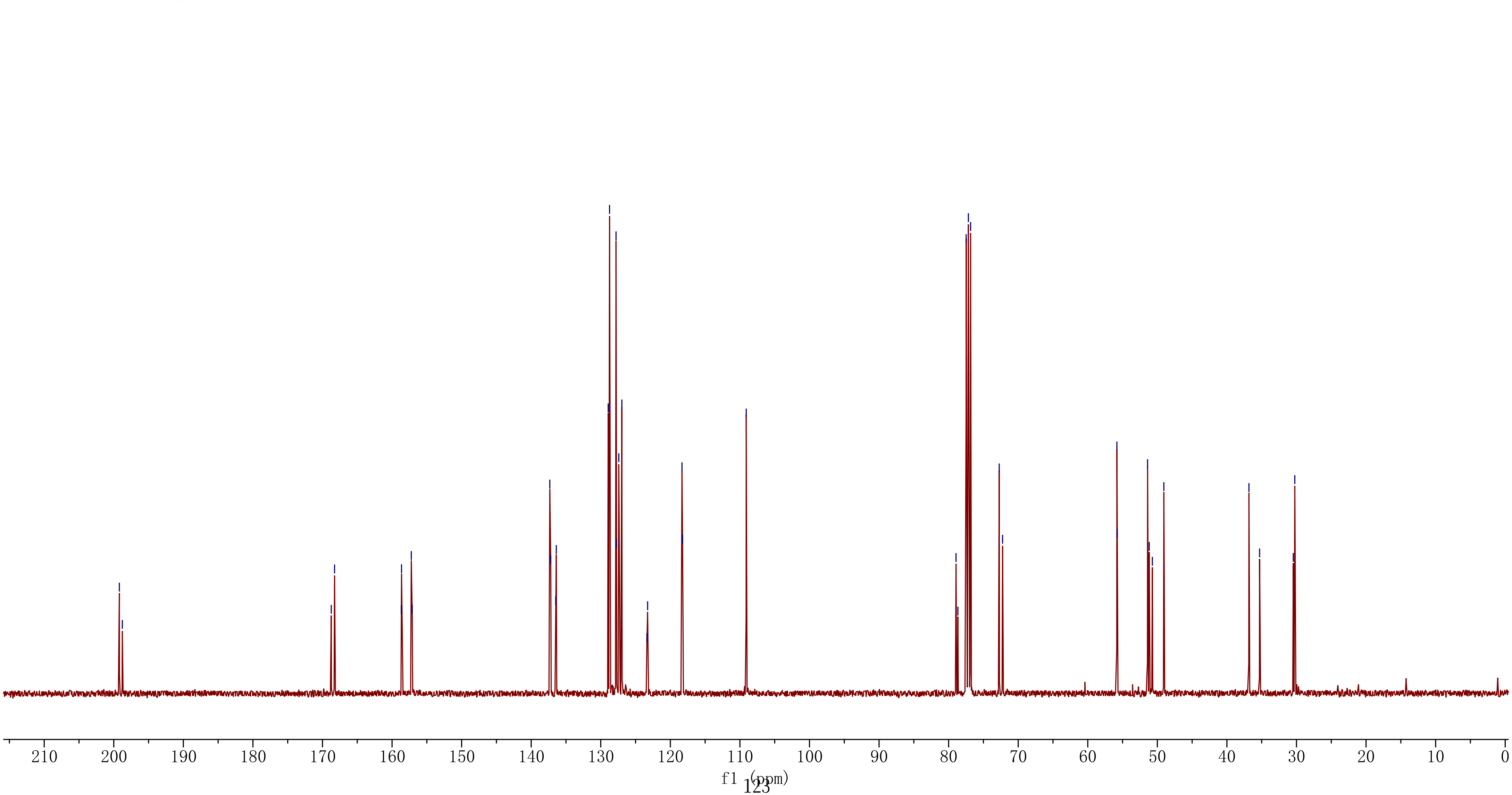

Fig. 2: 2m, ^{1}H NMR (400 MHz, $CDCl_3$)

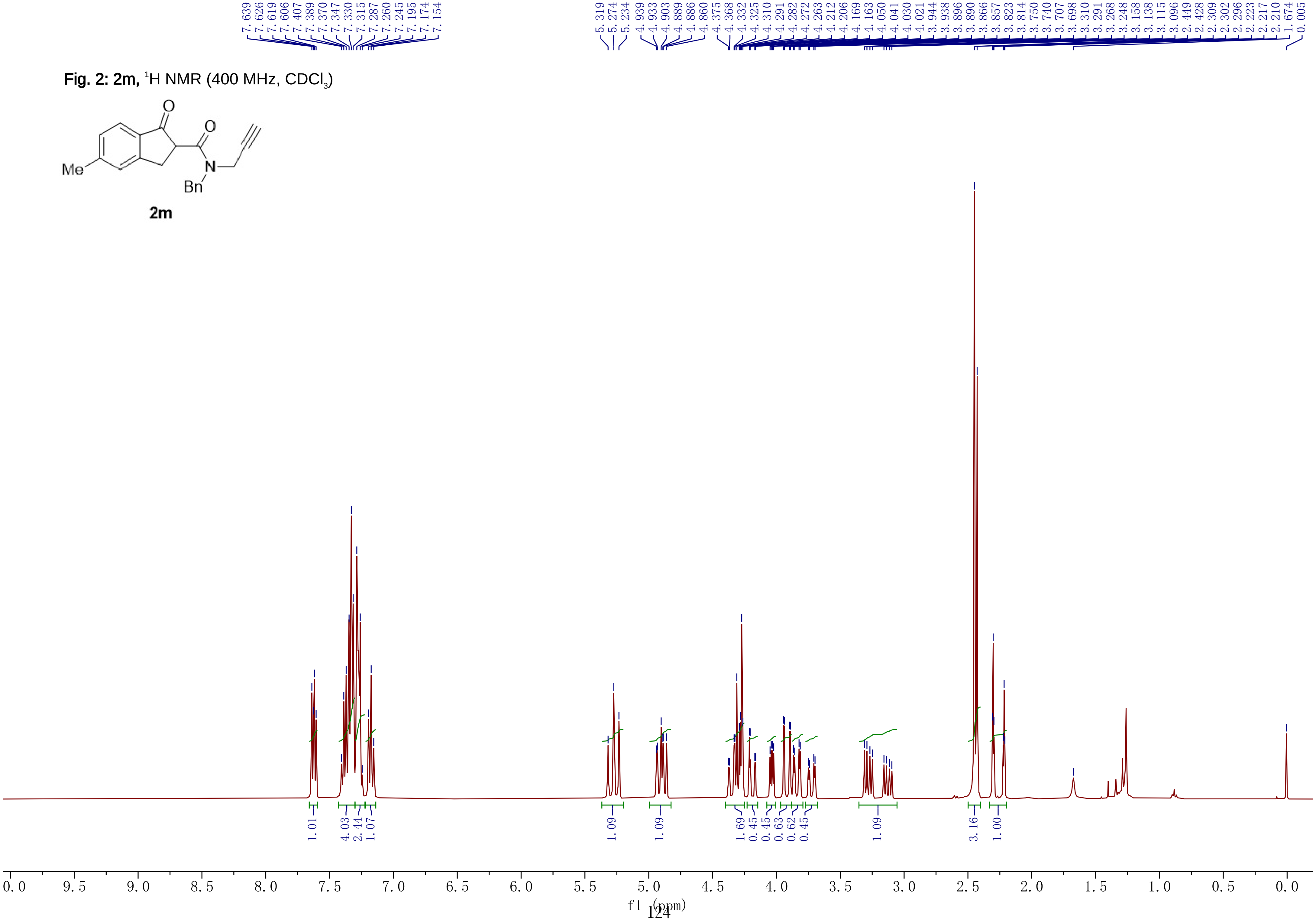

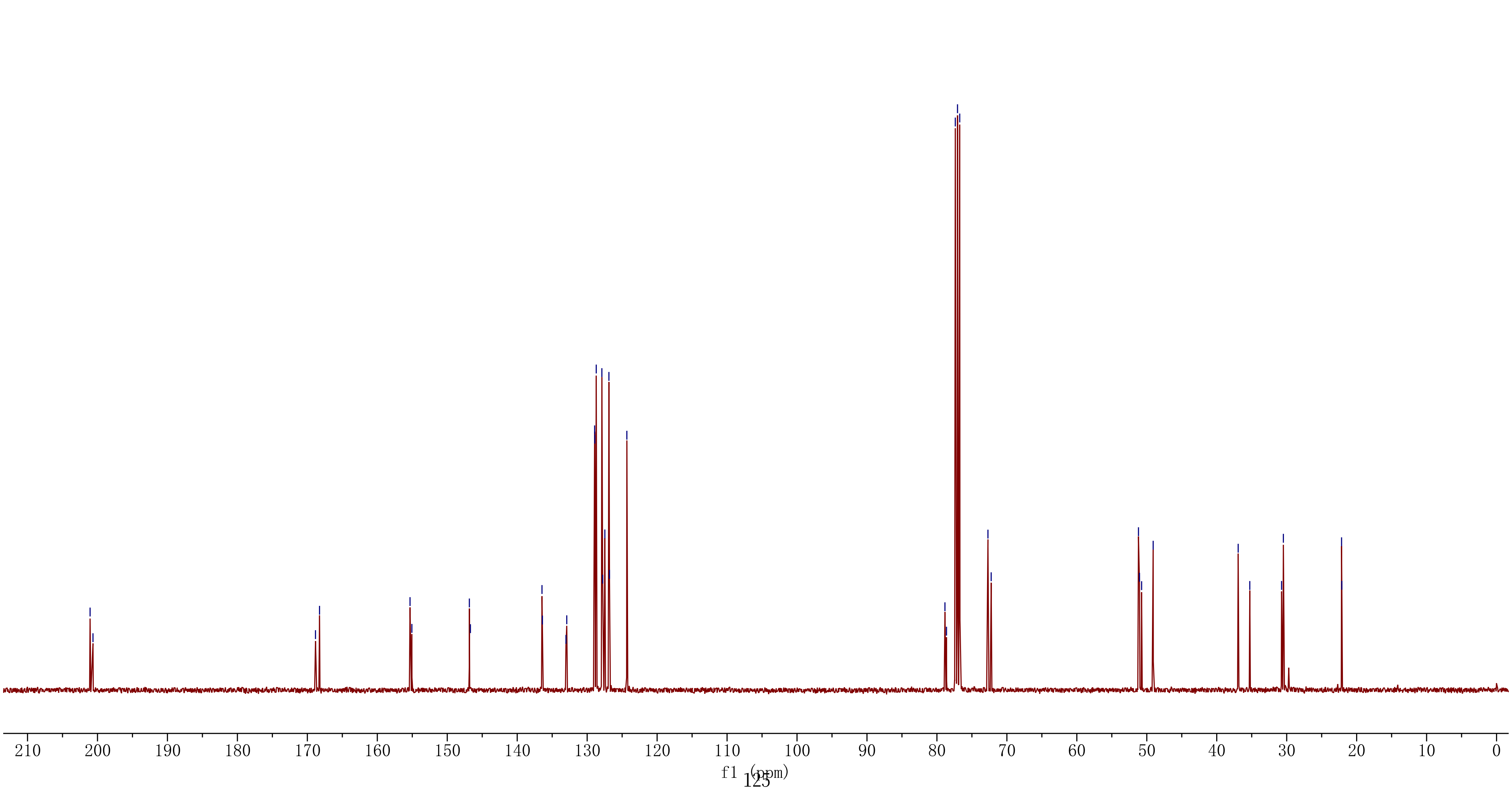

Fig. 2: 2m, ^{13}C NMR (101 MHz, $CDCl_3$)

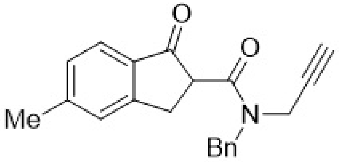

2m

Fig. 2: 2n, ^1H NMR (400 MHz, $CDCl_3$)

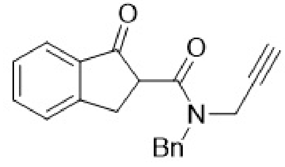

2n

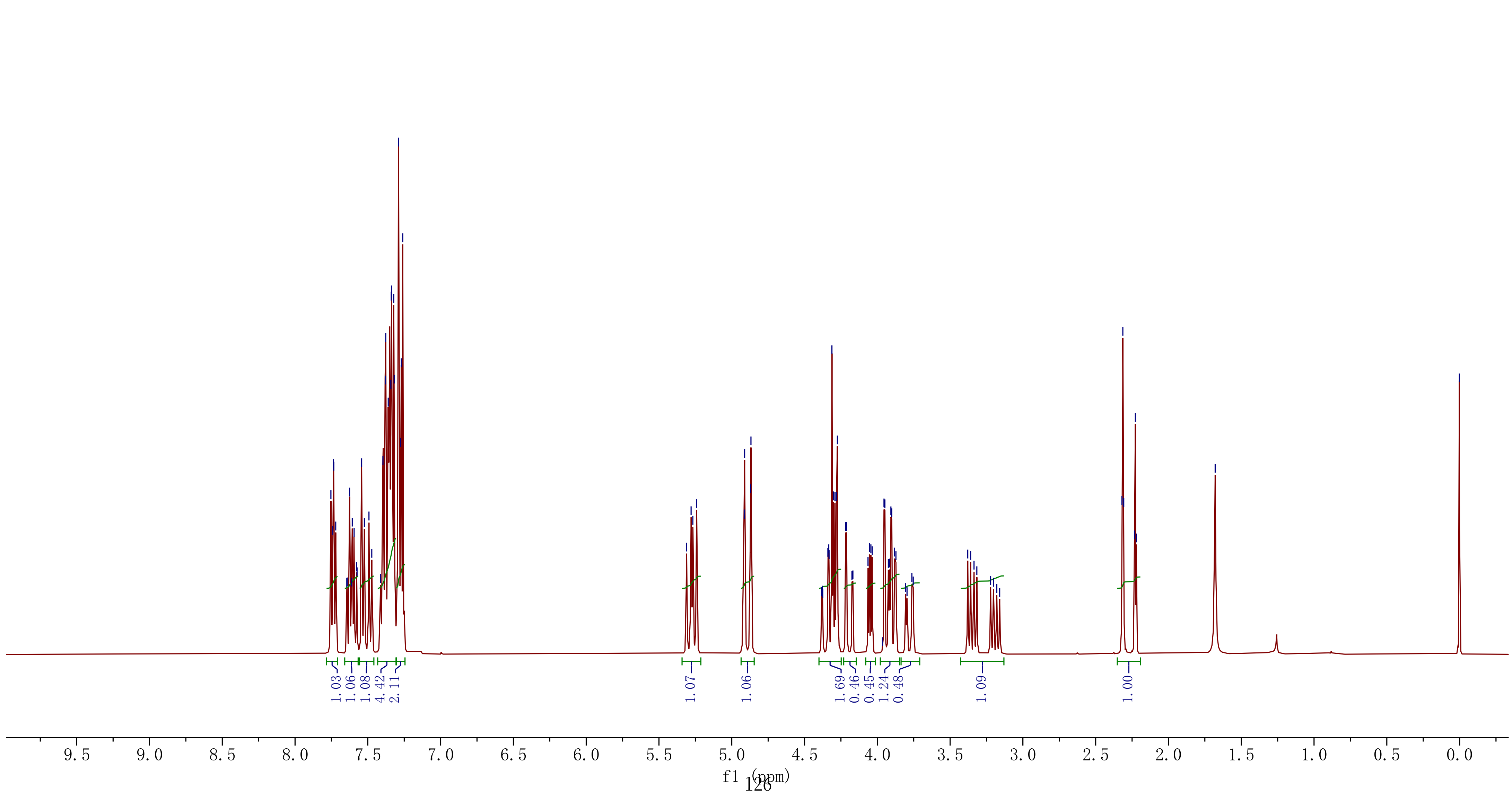

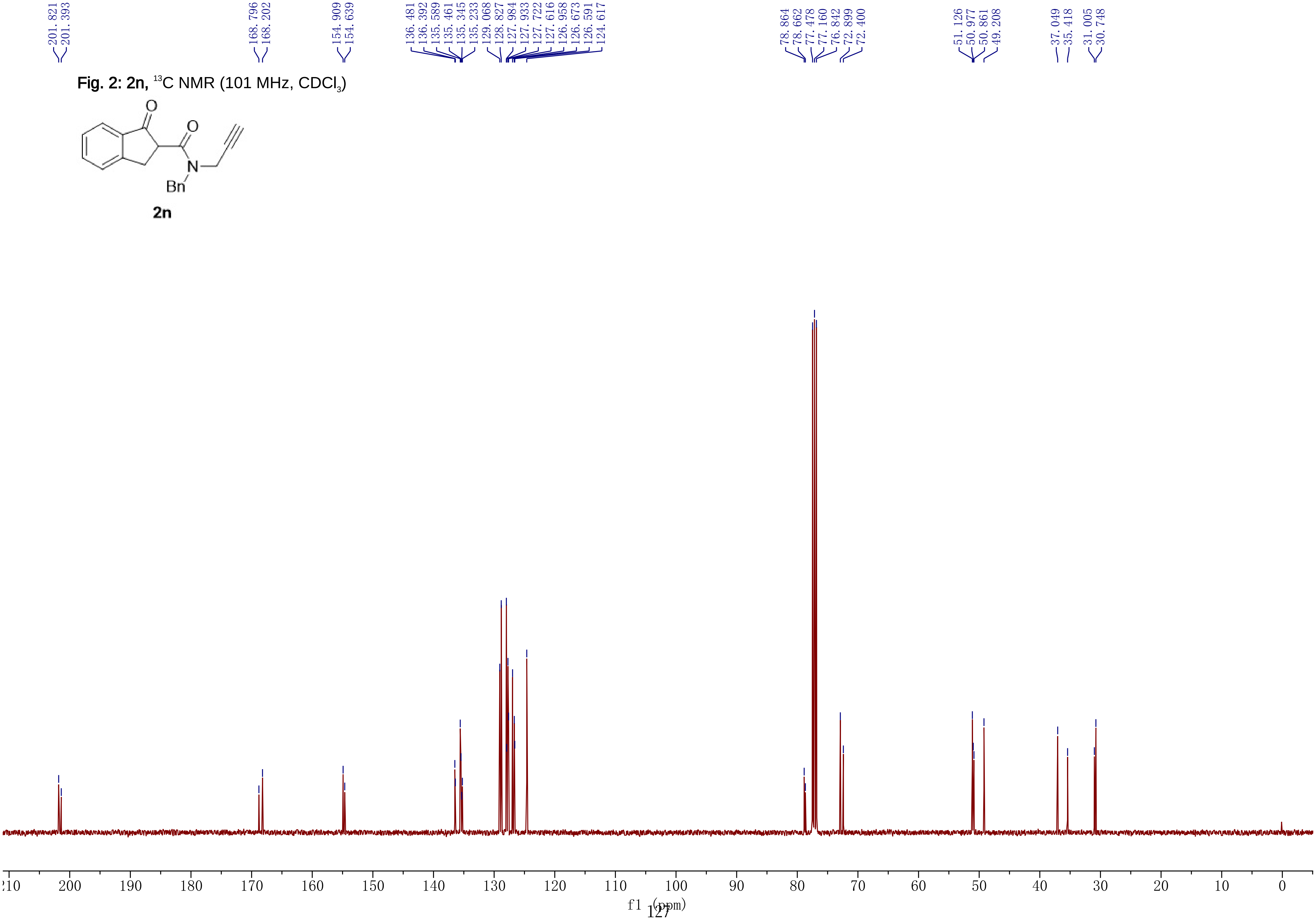

Fig. 2: 2n, ^{13}C NMR (101 MHz, $CDCl_3$)

Fig. 2: 2o, ^{1}H NMR (400 MHz, $CDCl_3$)

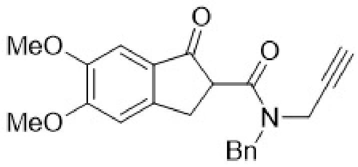

2o

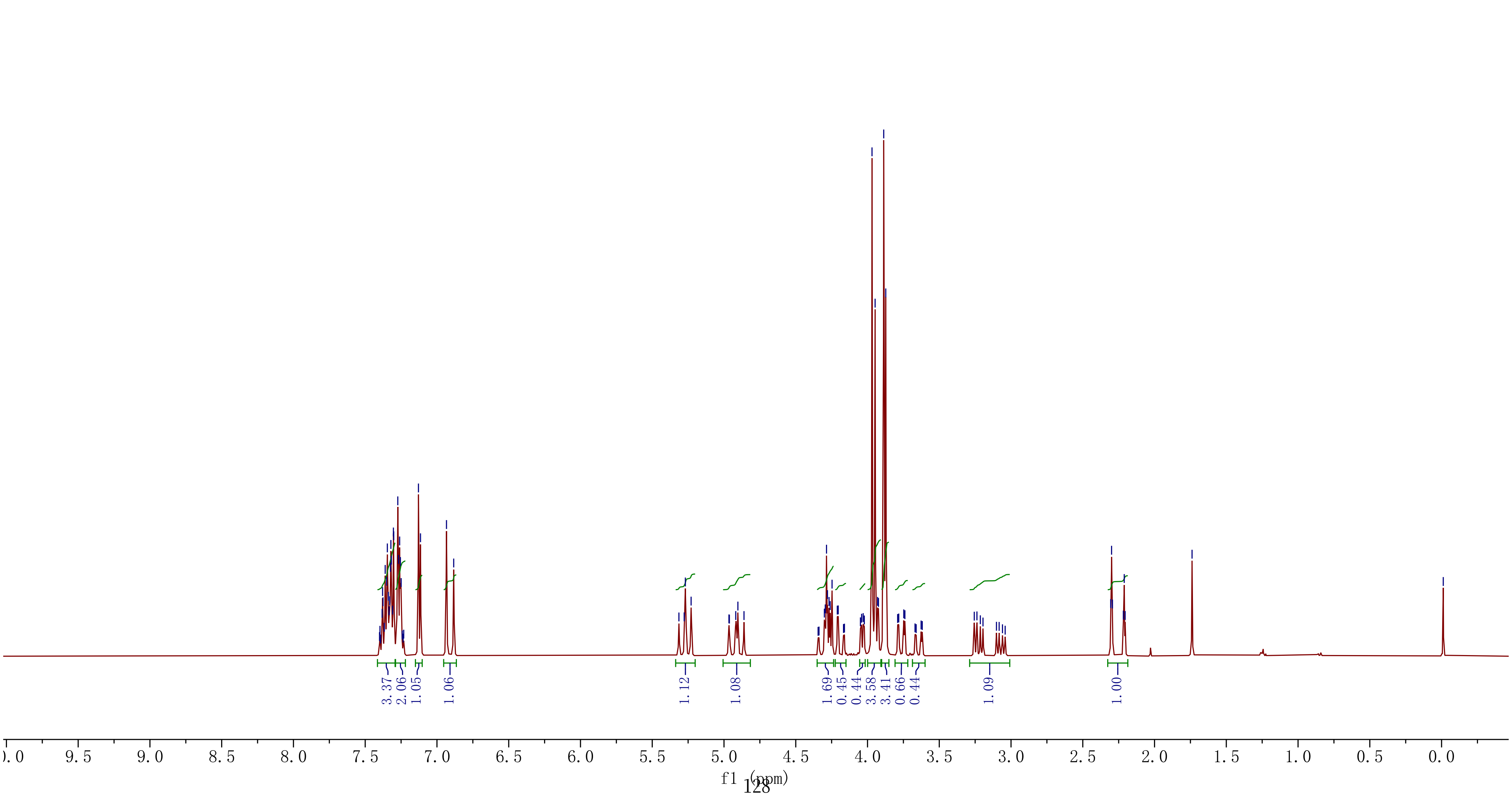

Fig. 2: 2o, ^{13}C NMR (101 MHz, CDCl$_3$)

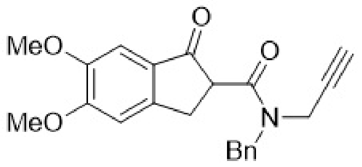

2o

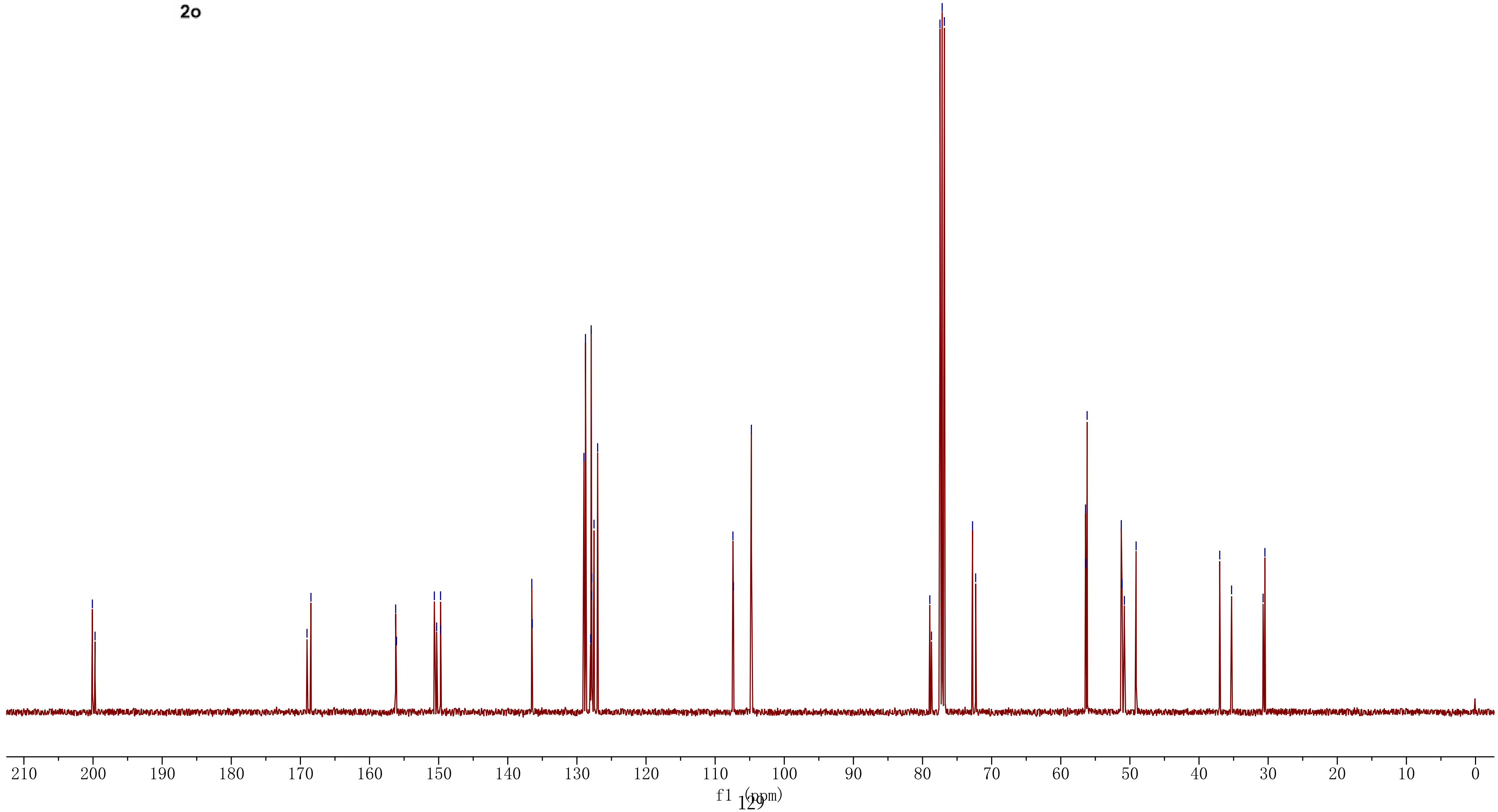

Fig. 2: 2p, ^{1}H NMR (400 MHz, $CDCl_3$)

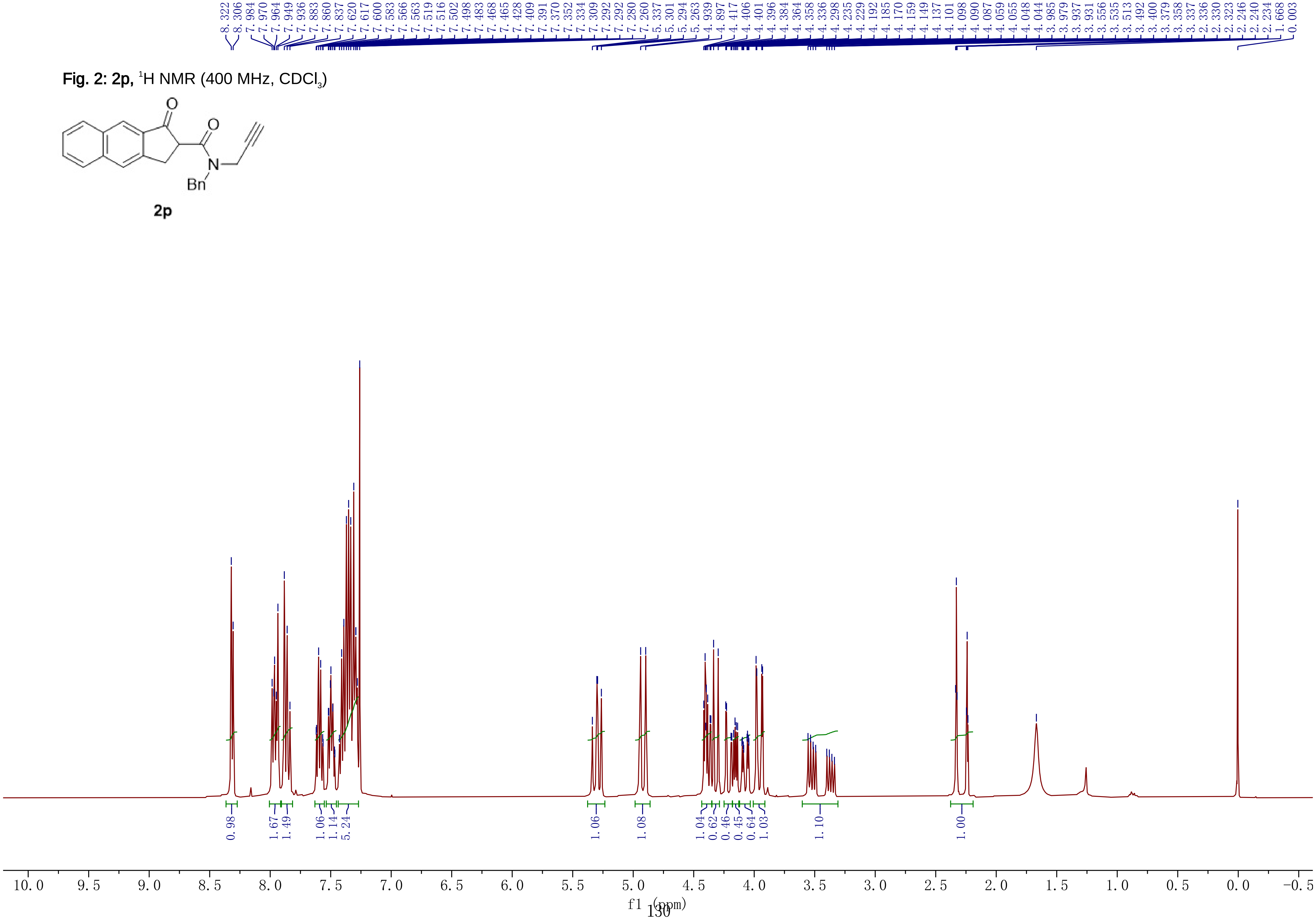

Fig. 2: 2p, ^{13}C NMR (101 MHz, $CDCl_3$)

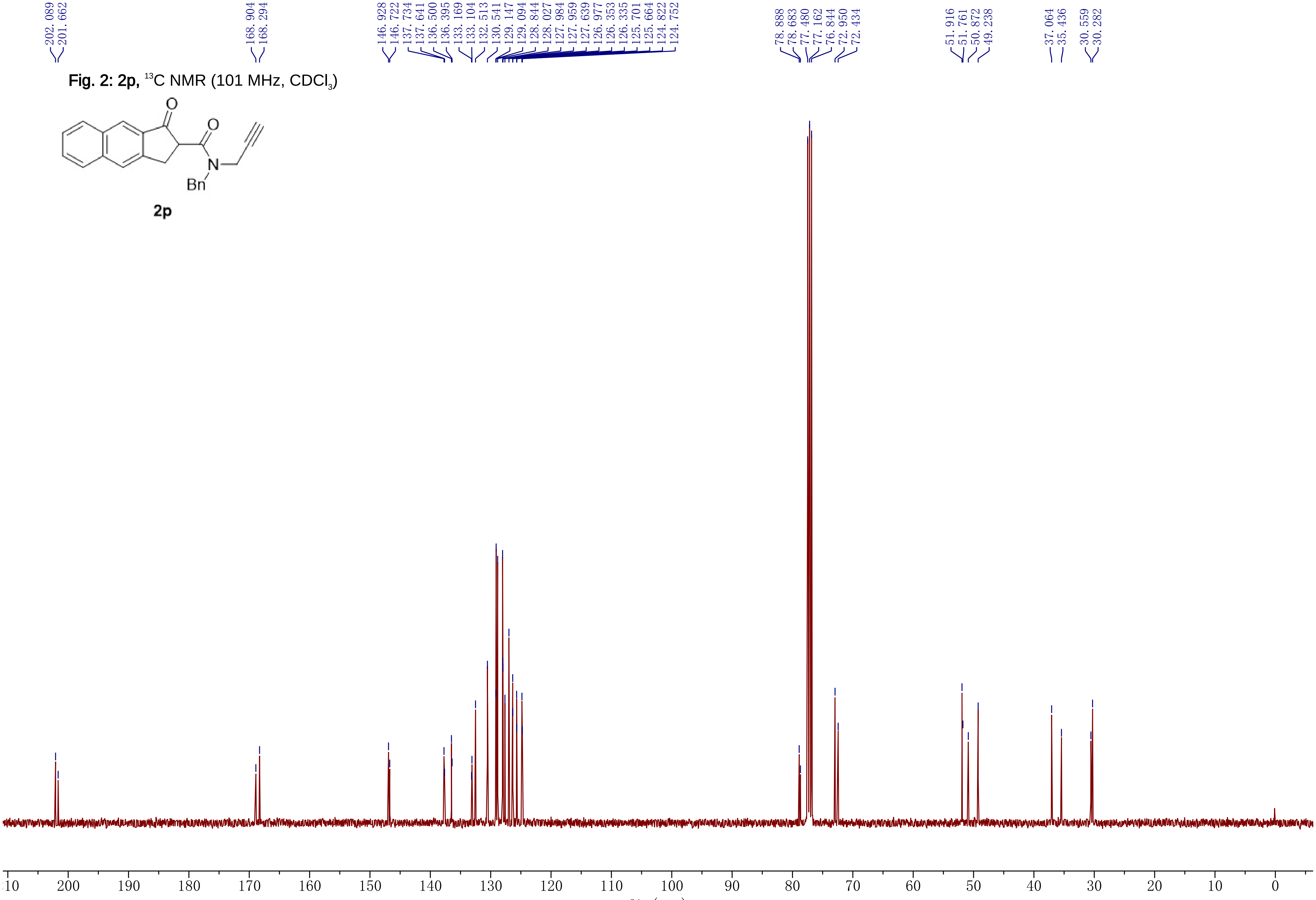

Fig. 2: 2q, ^{1}H NMR (400 MHz, $CDCl_3$)

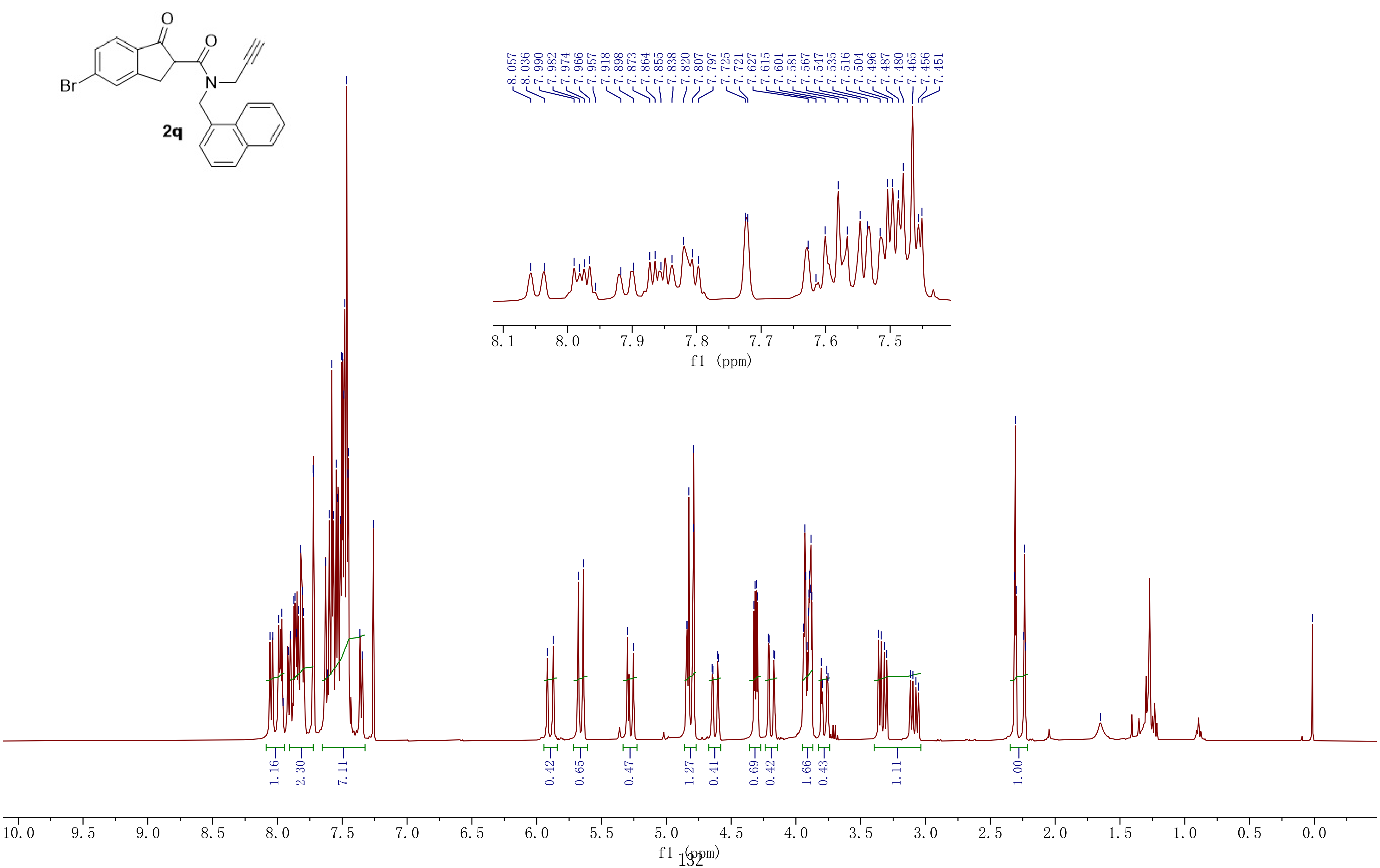

Fig. 2: 2q, ^{13}C NMR (101 MHz, $CDCl_3$)

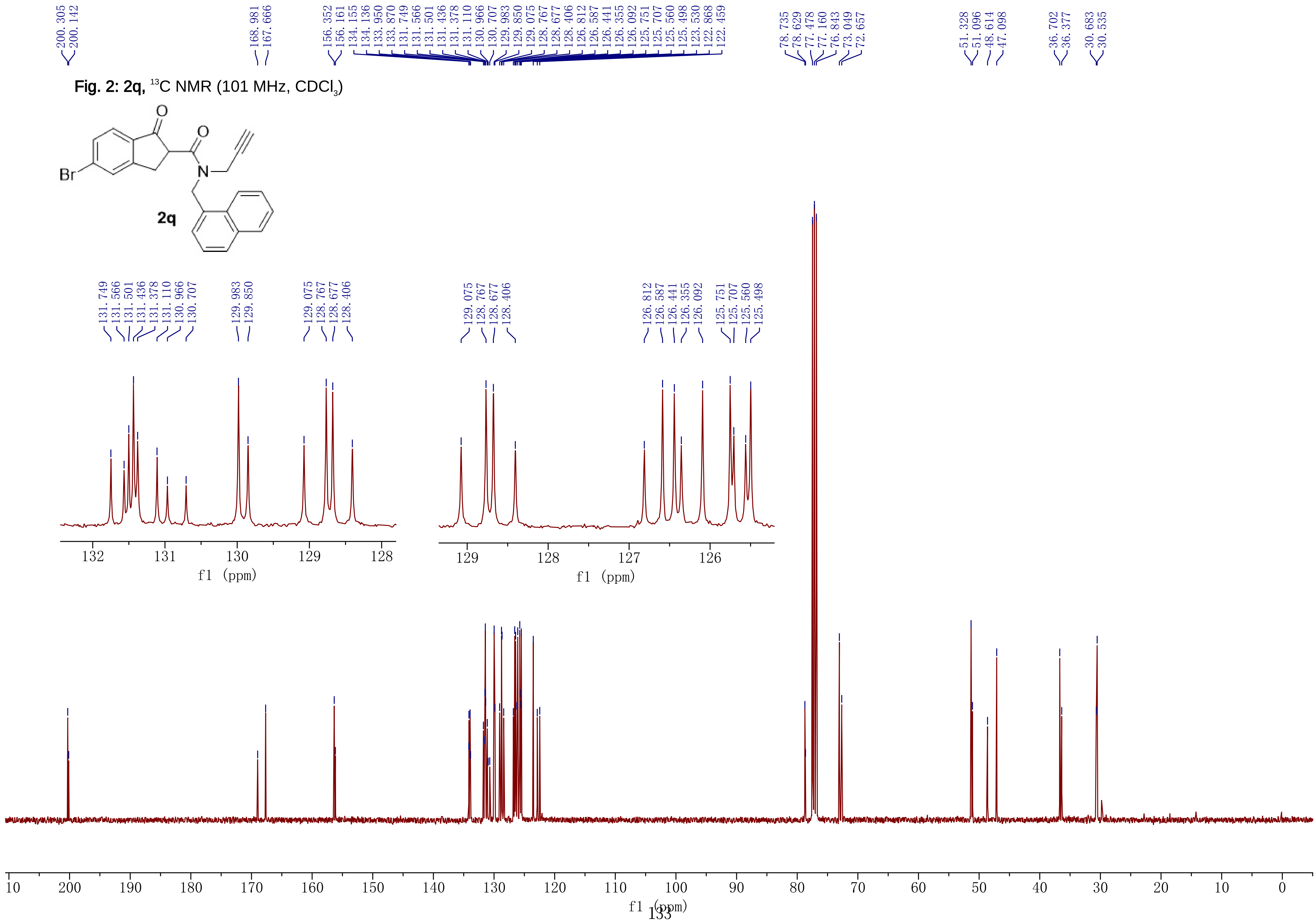

Fig. 2: 2r, ^{1}H NMR (400 MHz, $CDCl_3$)

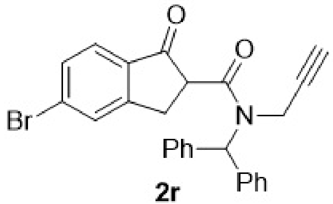

2r

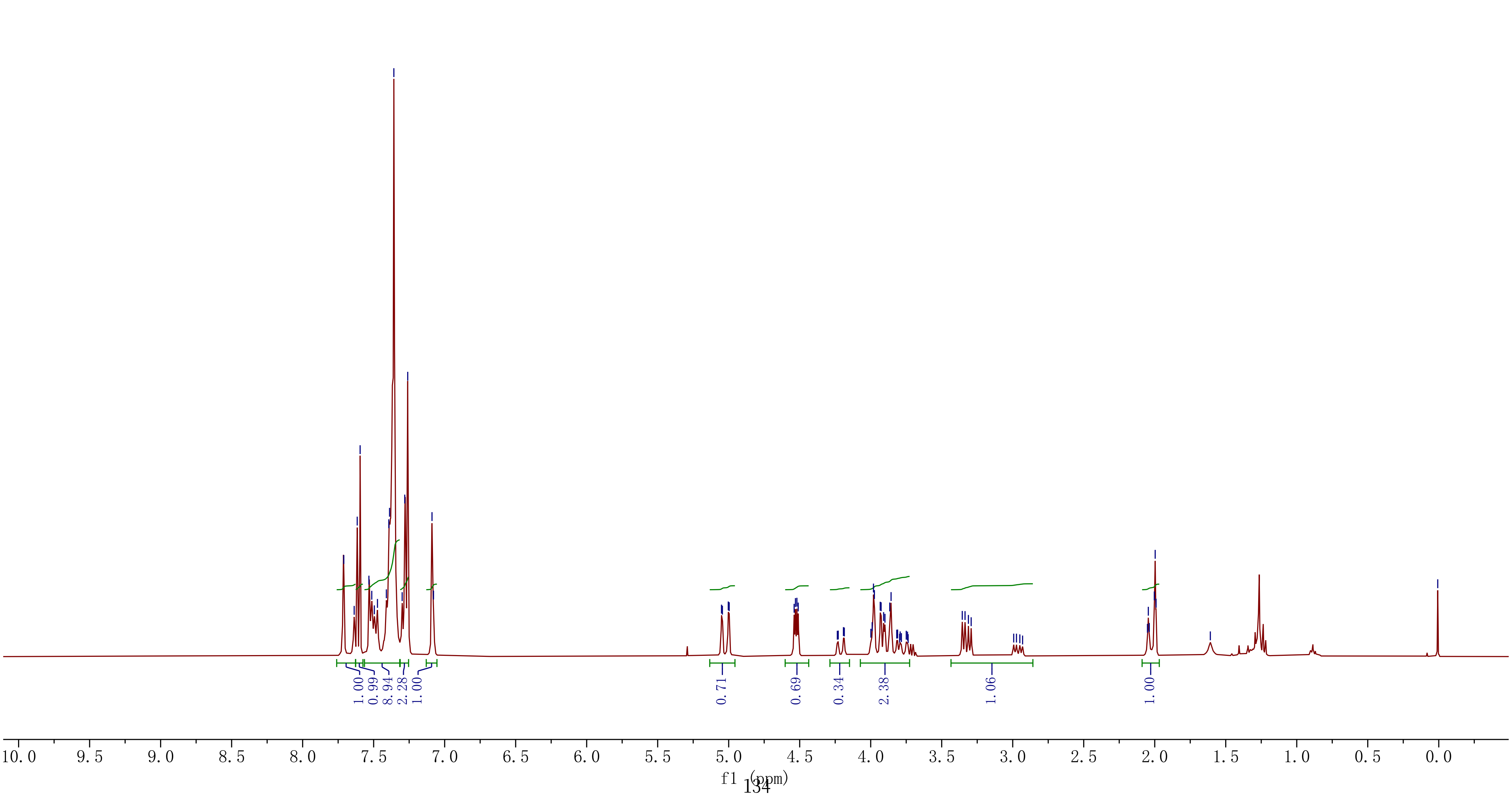

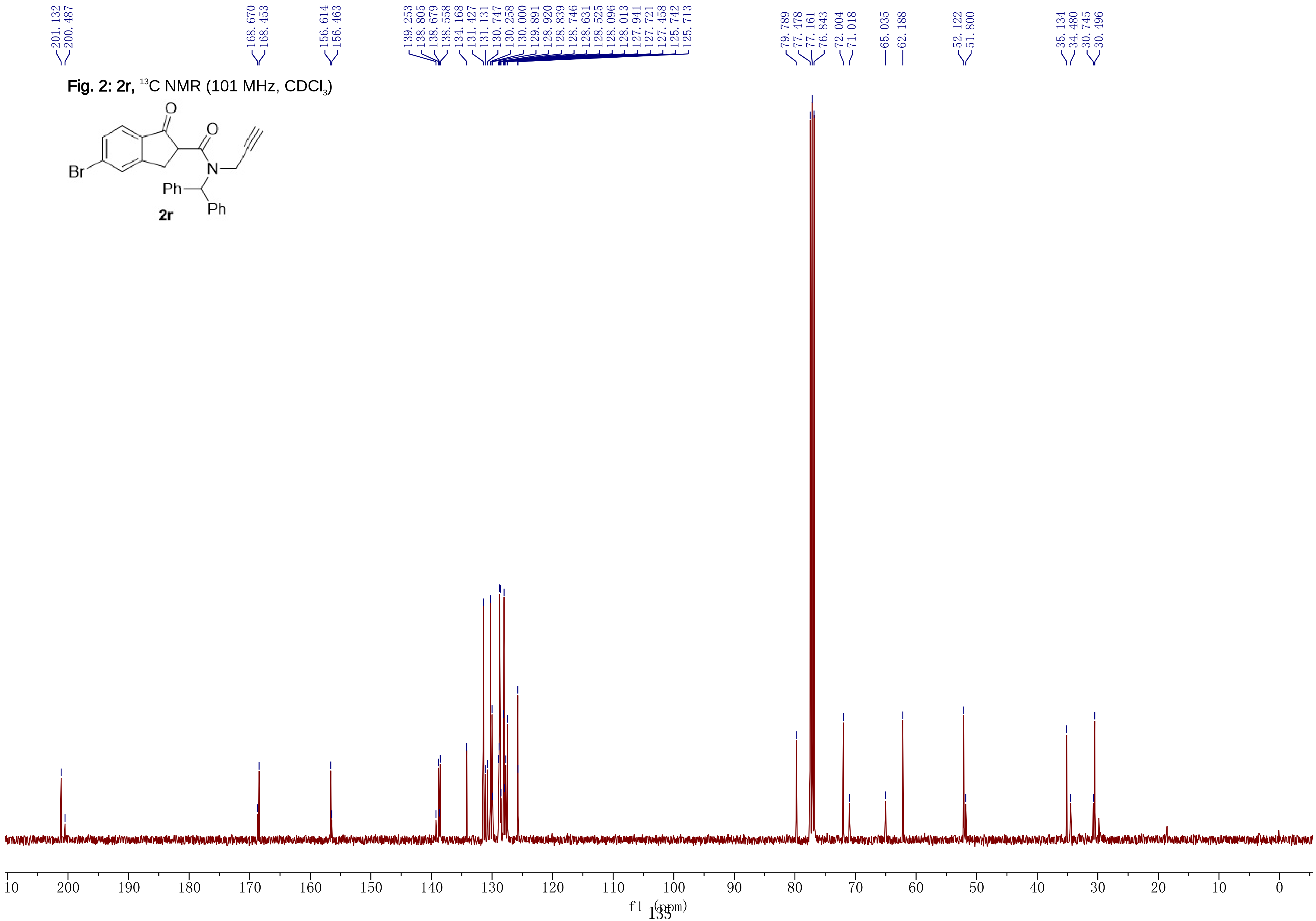

Fig. 2: 2r, ^{13}C NMR (101 MHz, $CDCl_3$)

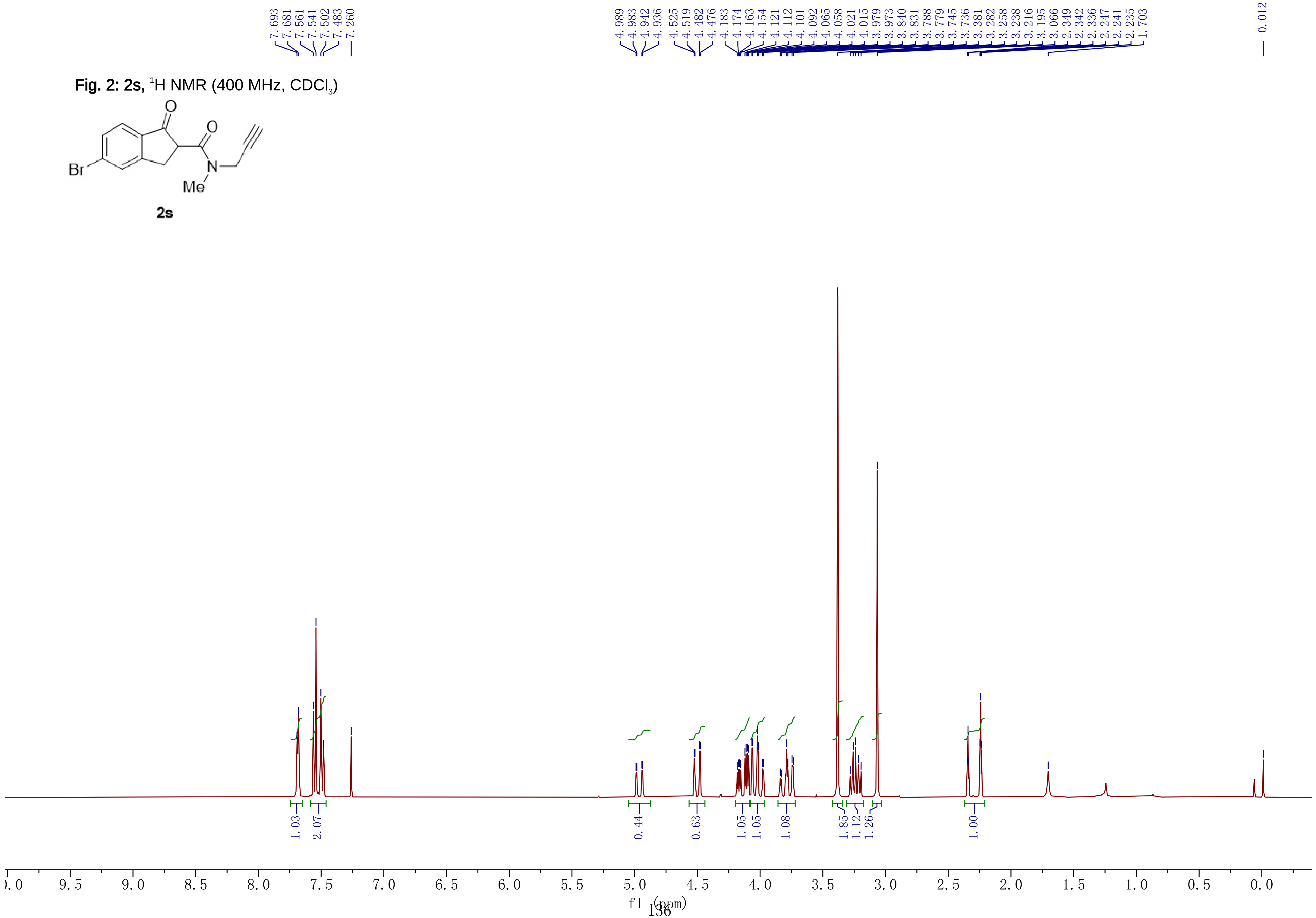

Fig. 2: 2s, ^{1}H NMR (400 MHz, $CDCl_3$)

Fig. 2: 2s, ^{13}C NMR (101 MHz, $CDCl_3$)

2s

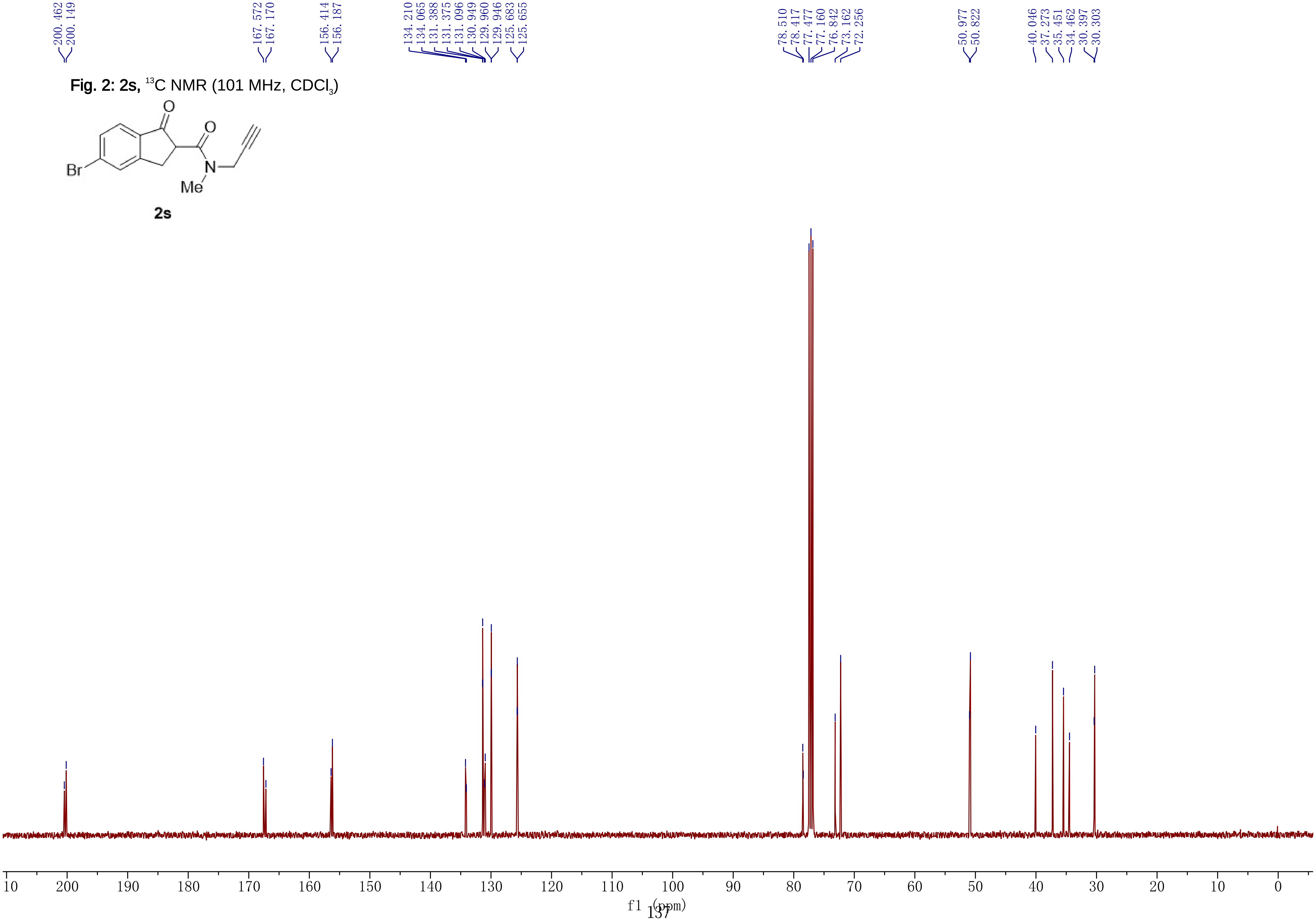

Fig. 2: 2t, ^{1}H NMR (400 MHz, $CDCl_3$)

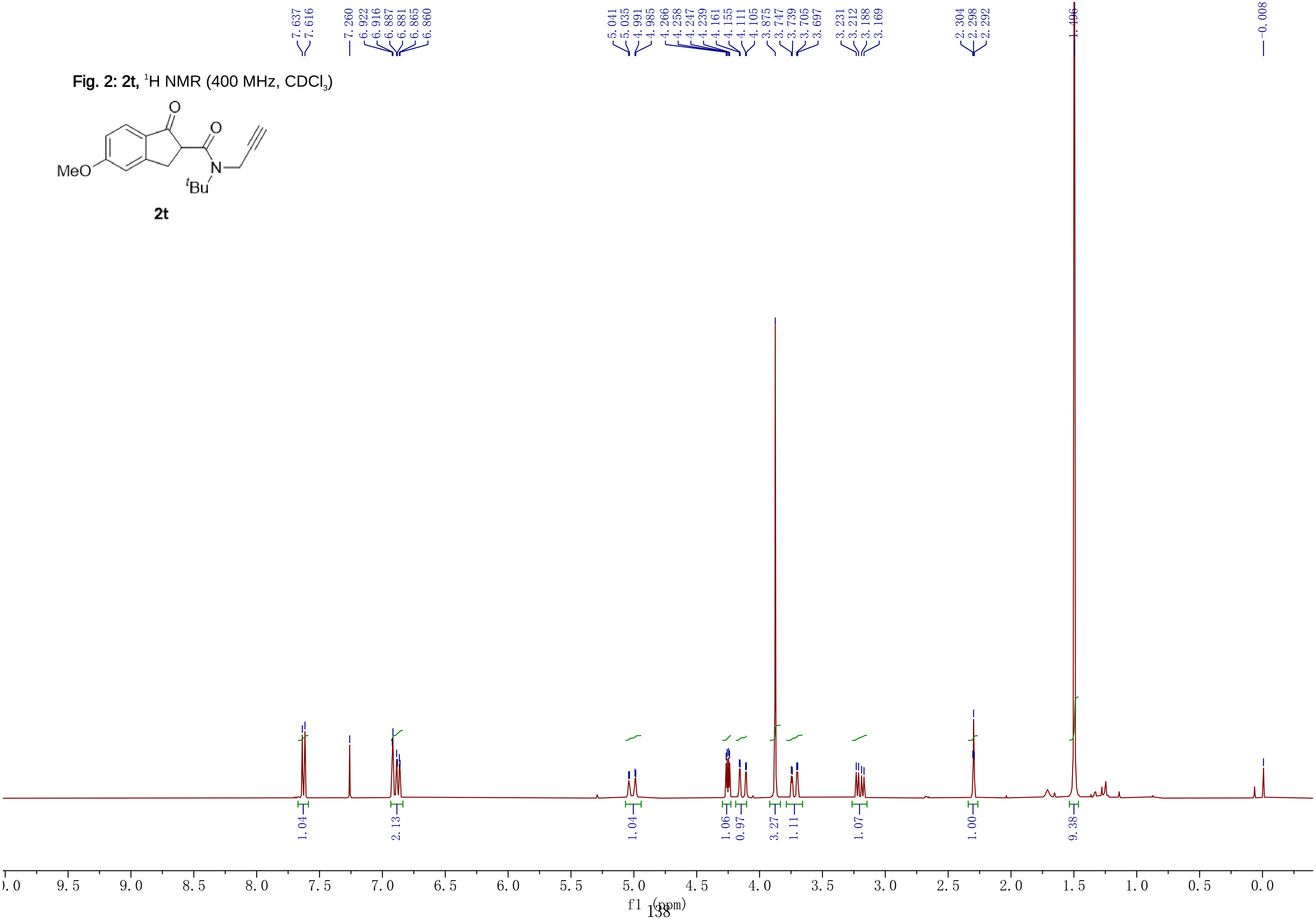

2t

Fig. 2: 2t, ^{13}C NMR (101 MHz, $CDCl_3$)

2t

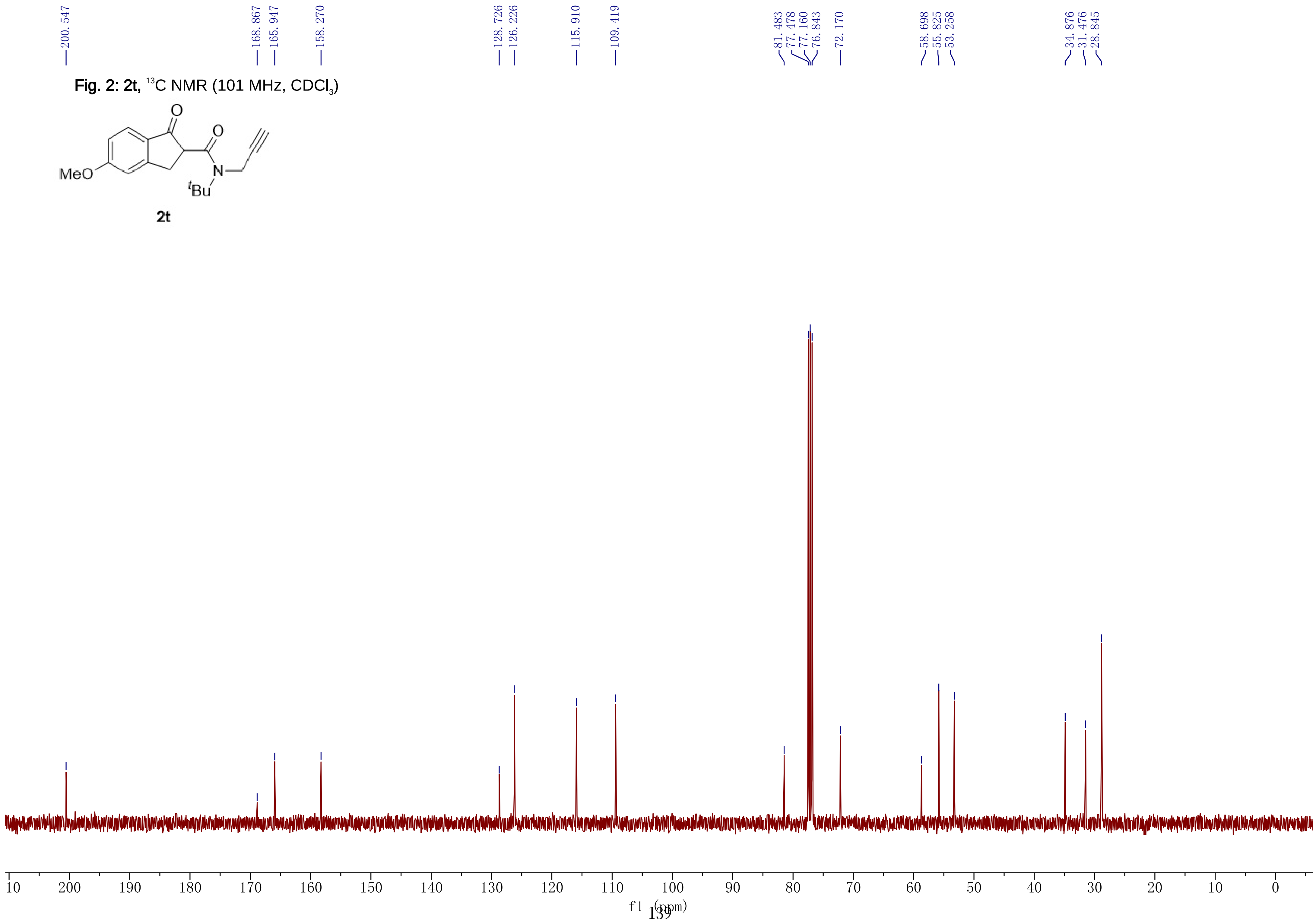

Fig. 2: 2u, ^{1}H NMR (400 MHz, $CDCl_3$)

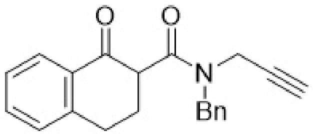

2u

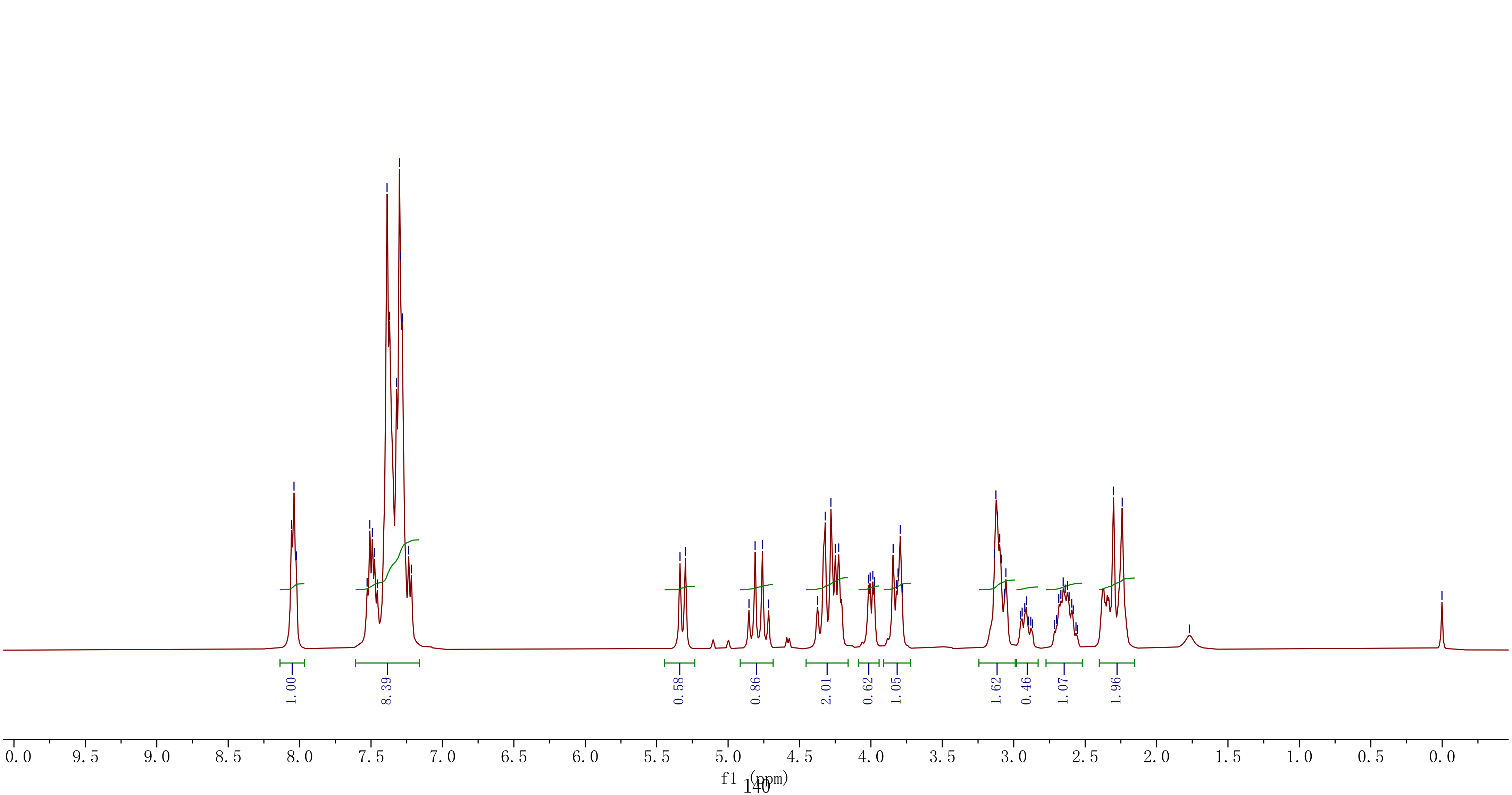

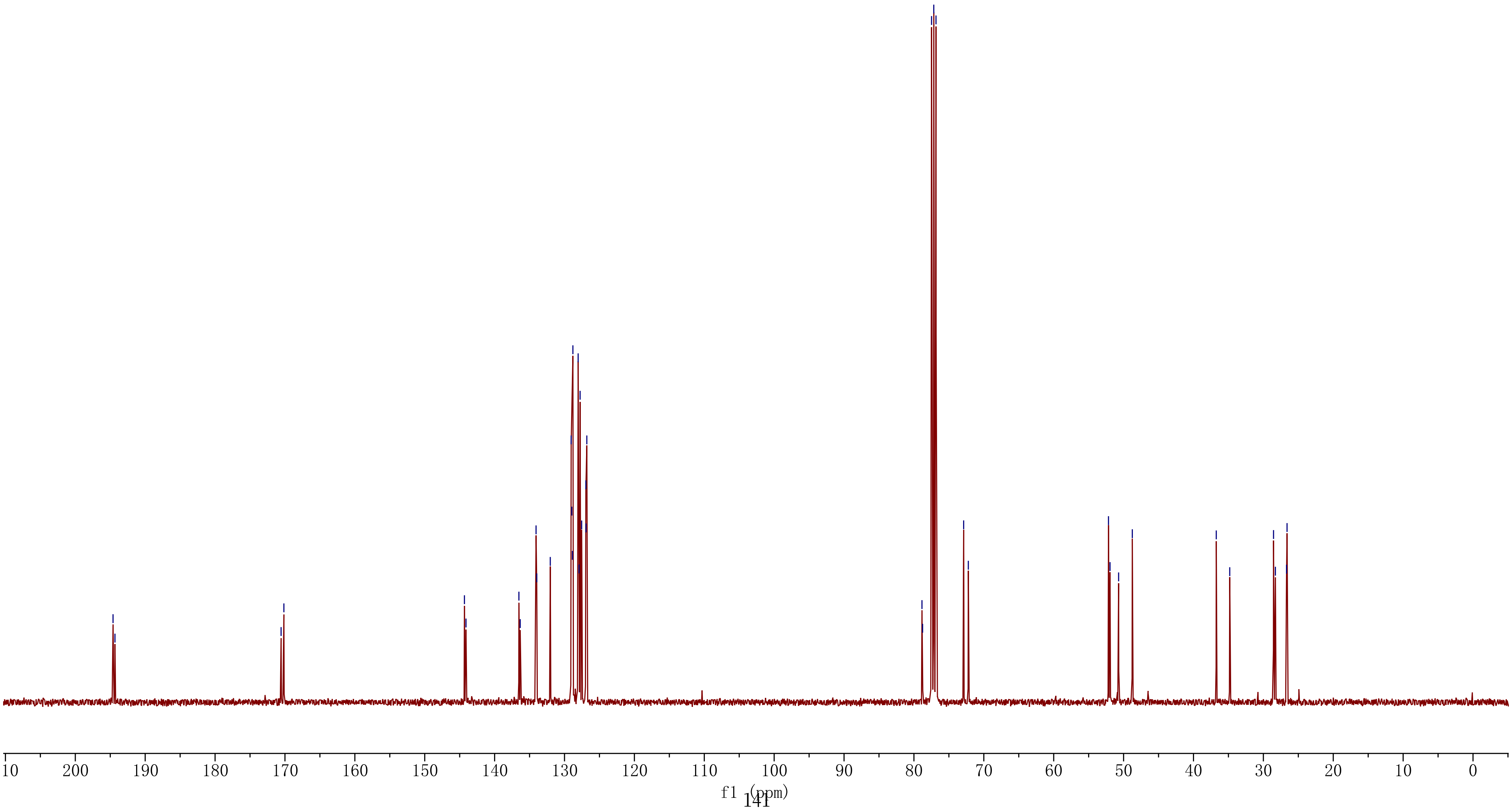

Fig. 2: 2u, ^{13}C NMR (101 MHz, $CDCl_3$)

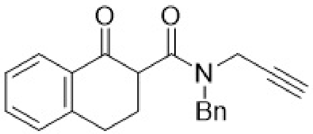

2u

Fig. 2: 2v, ^{1}H NMR (400 MHz, $CDCl_3$)

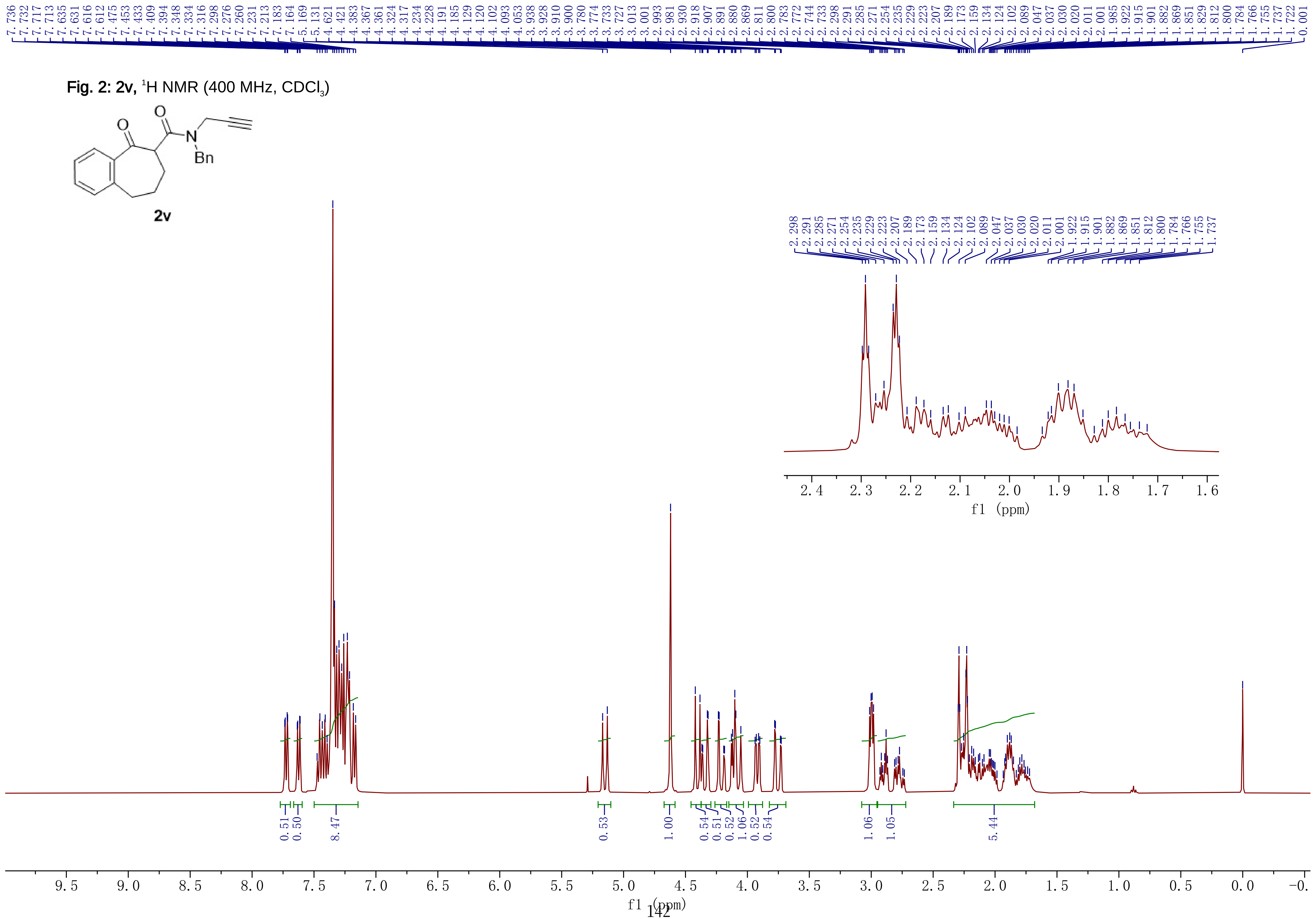

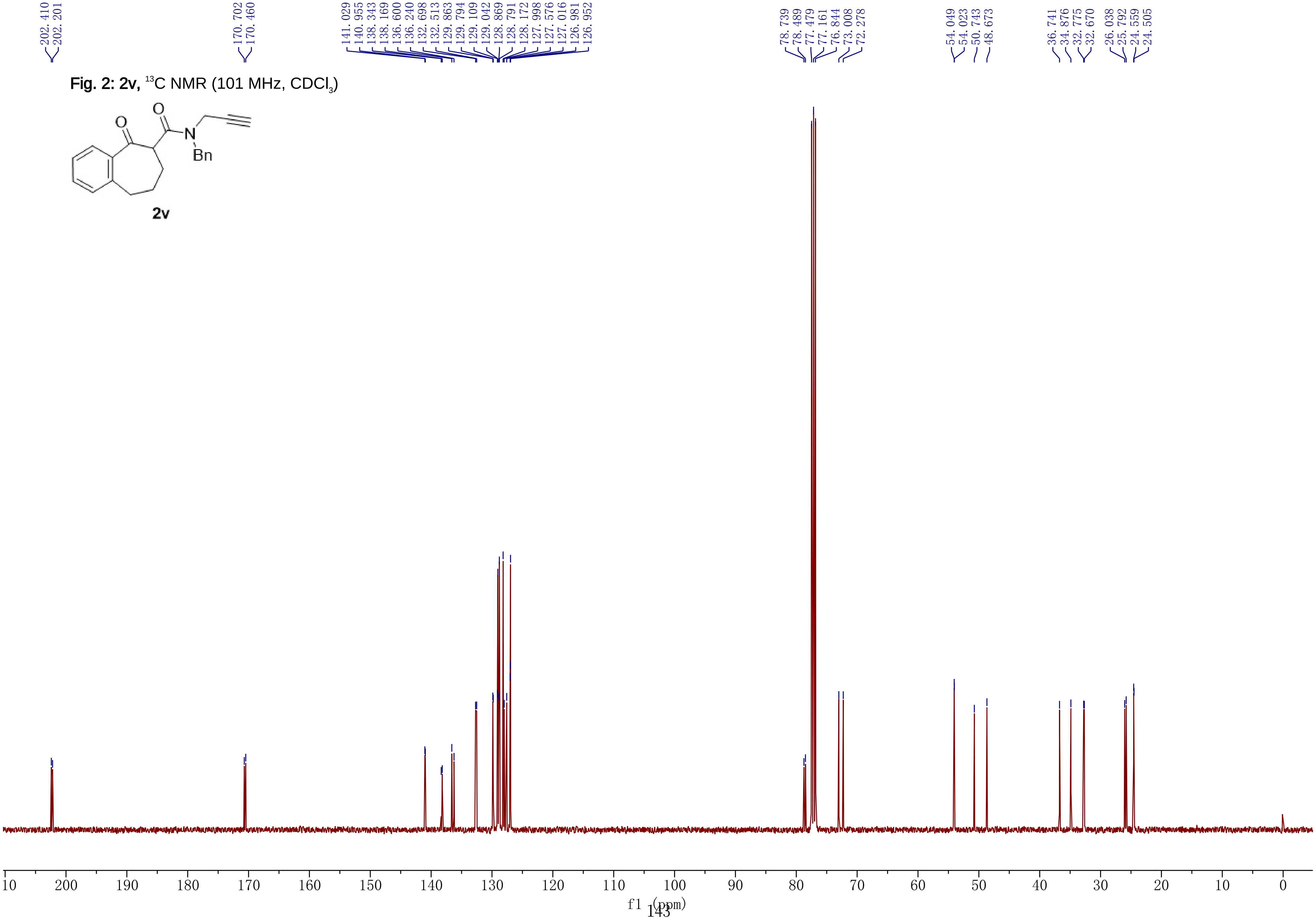

Fig. 2: 2v, ^{13}C NMR (101 MHz, $CDCl_3$)

Fig. 2: 2w, ^{1}H NMR (400 MHz, $CDCl_3$)

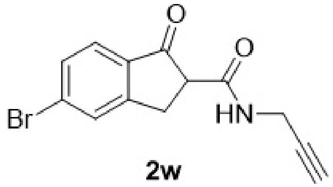

2w

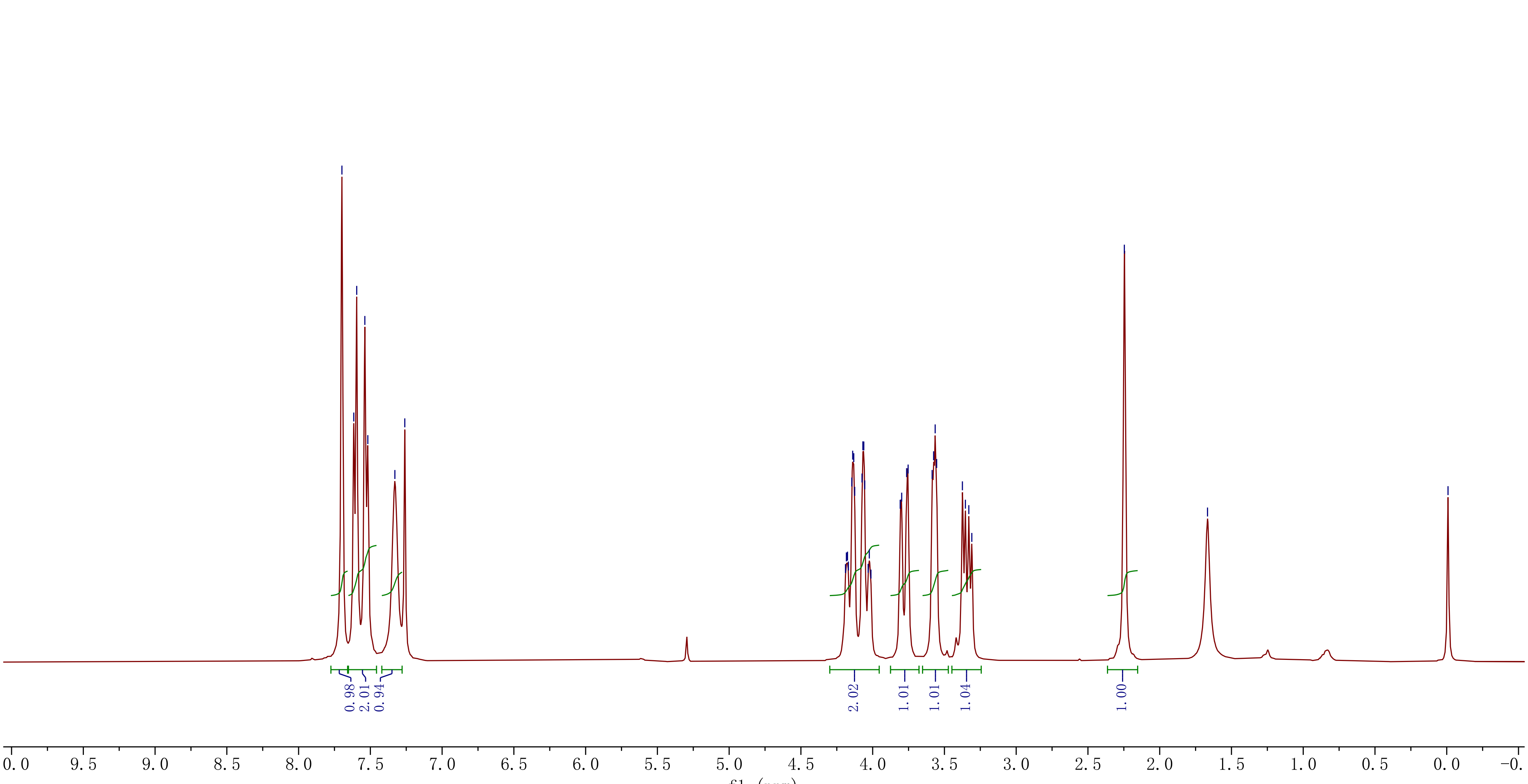

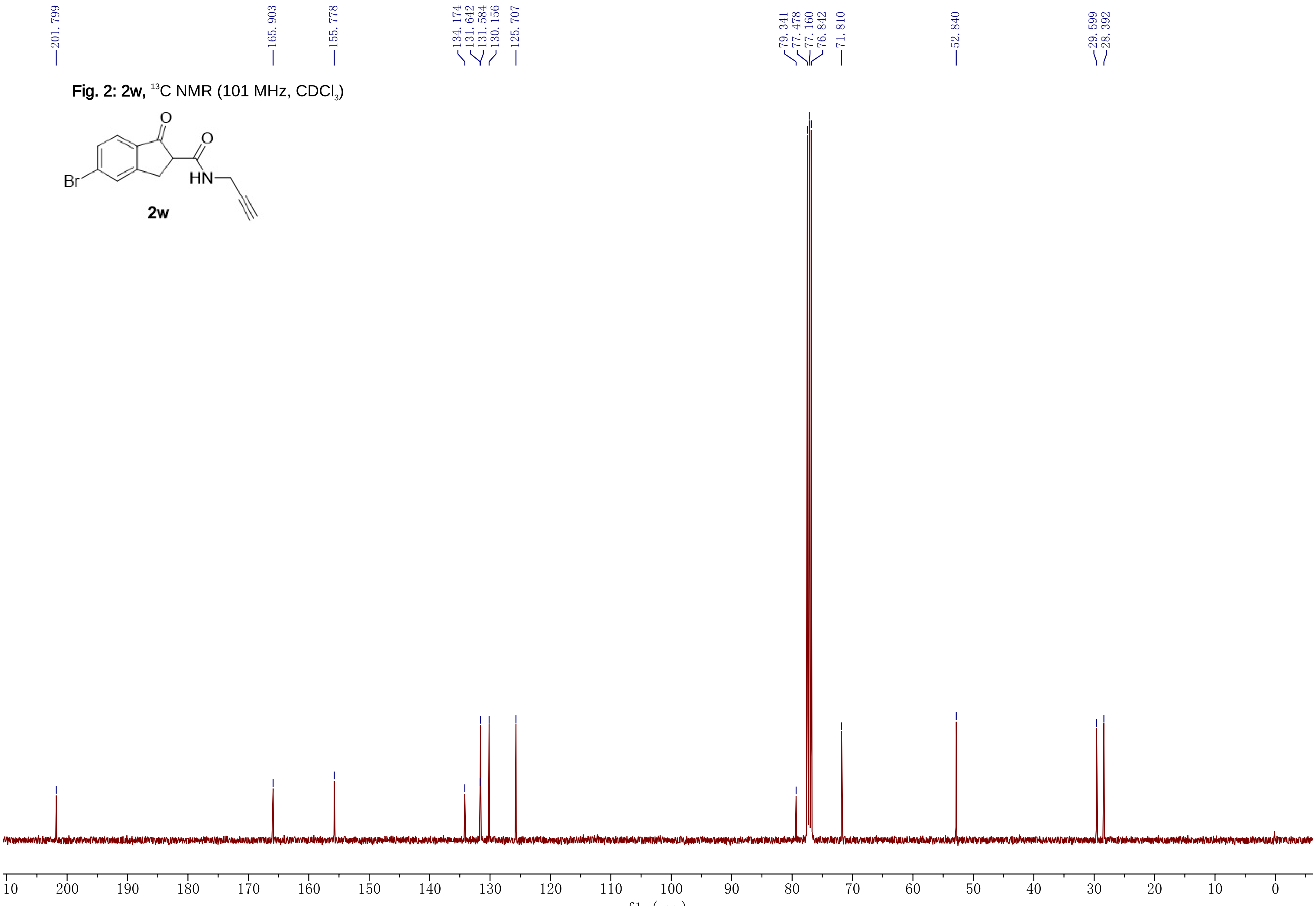

Fig. 2: 2w, ^{13}C NMR (101 MHz, $CDCl_3$)

Fig. 2: 2x, ^{1}H NMR (400 MHz, $CDCl_3$)

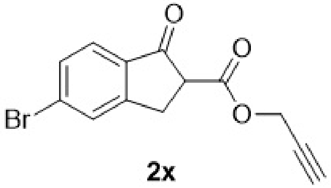

2x

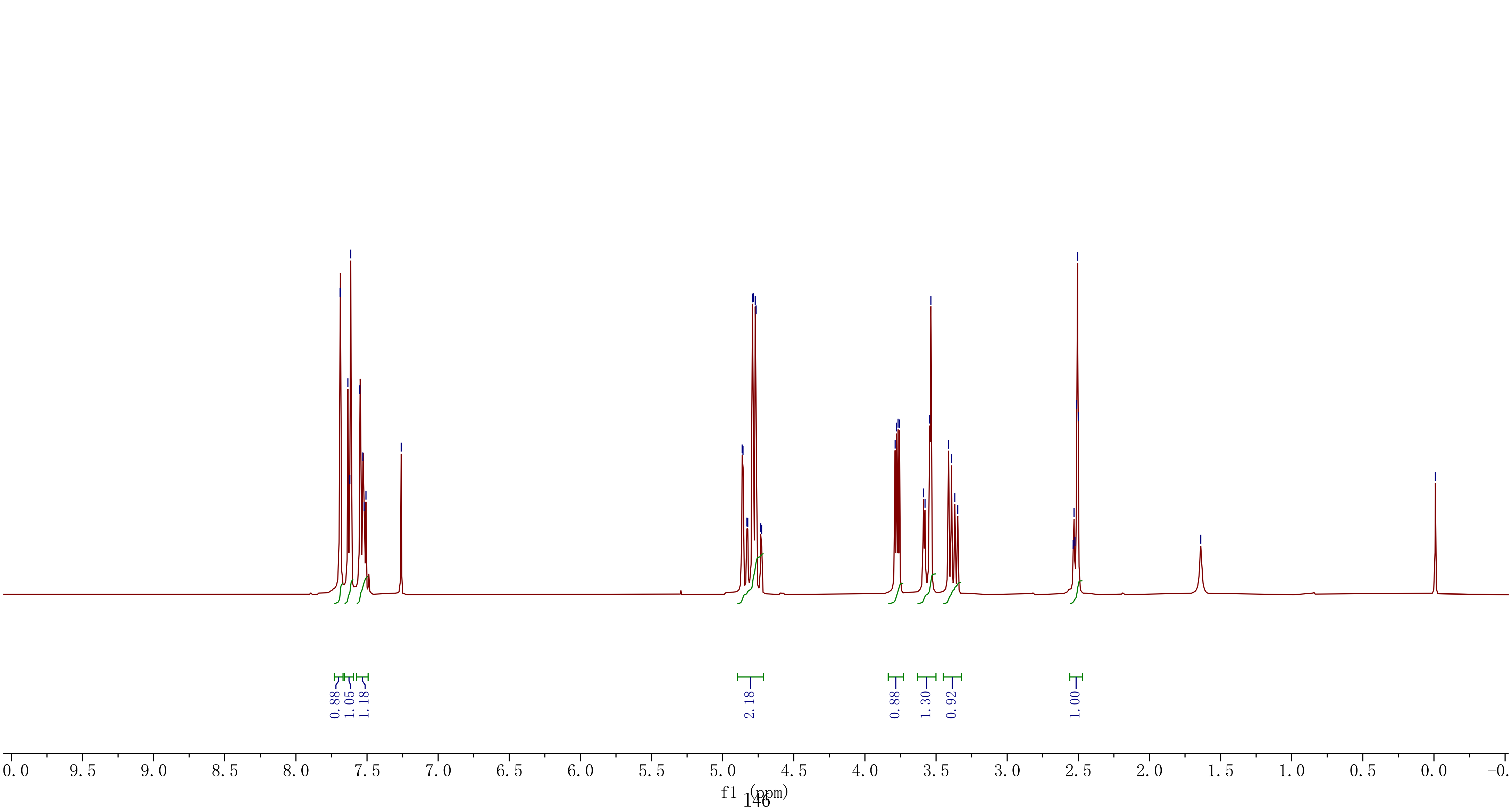

Fig. 2: 2x, ^{13}C NMR (101 MHz, $CDCl_3$)

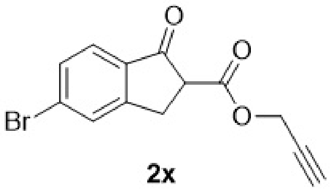

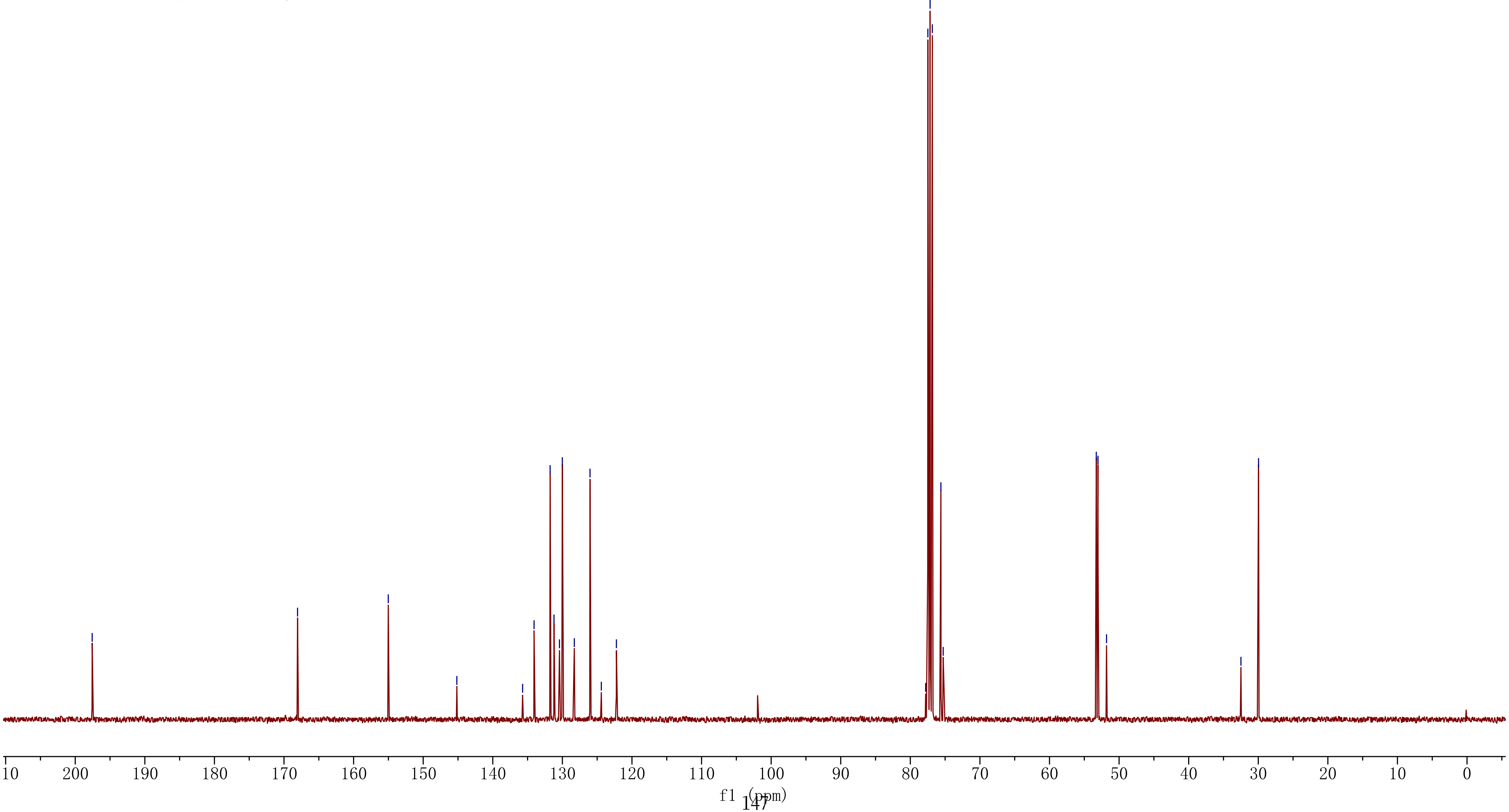

Fig. 3: 4a, ^{1}H NMR (400 MHz, $CDCl_3$)

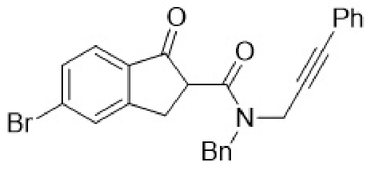

4a

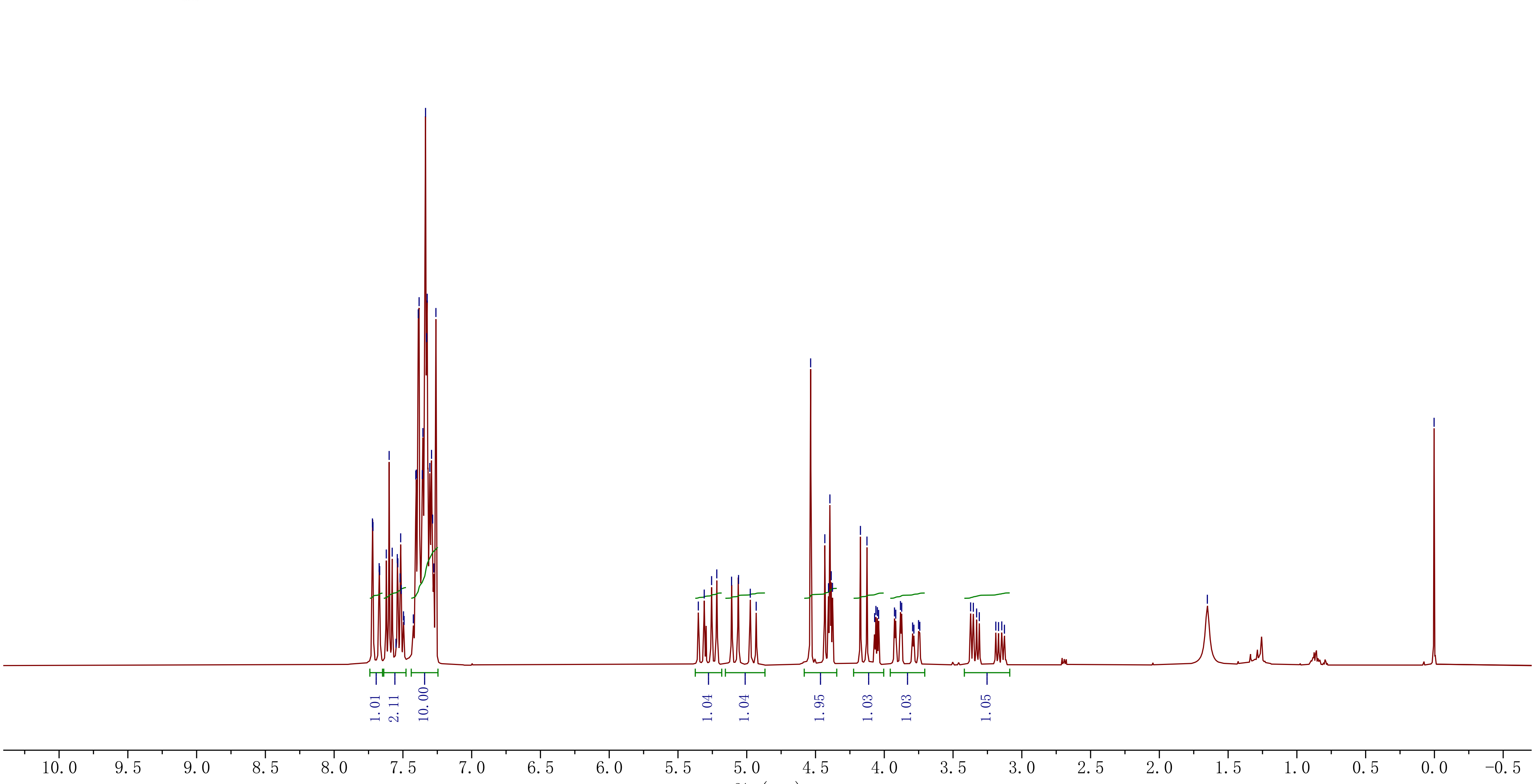

Fig. 3: 4a, ^{13}C NMR (101 MHz, $CDCl_3$)

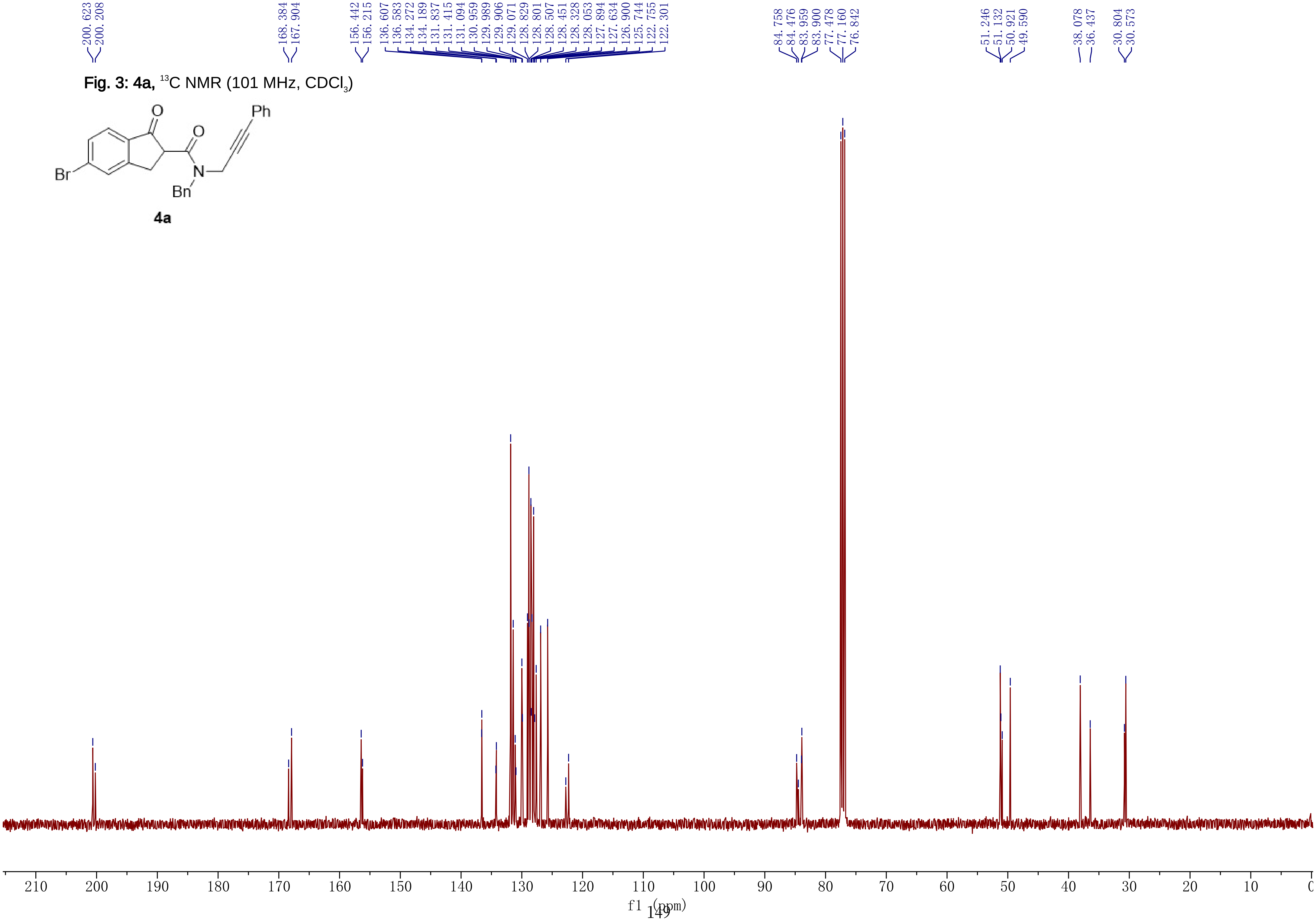

Fig. 3: 4b, ^{1}H NMR (400 MHz, $CDCl_3$)

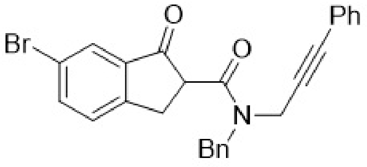

4b

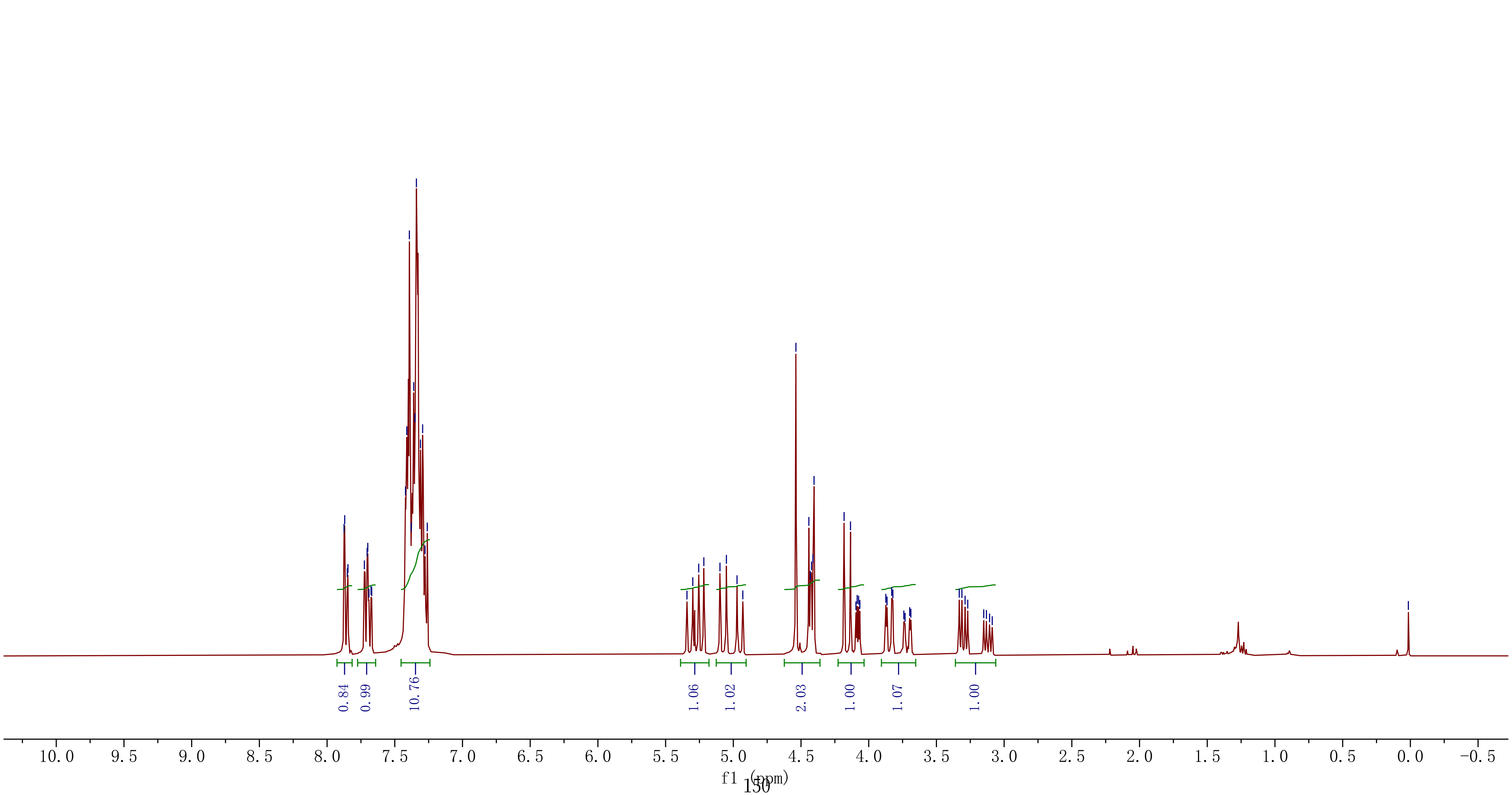

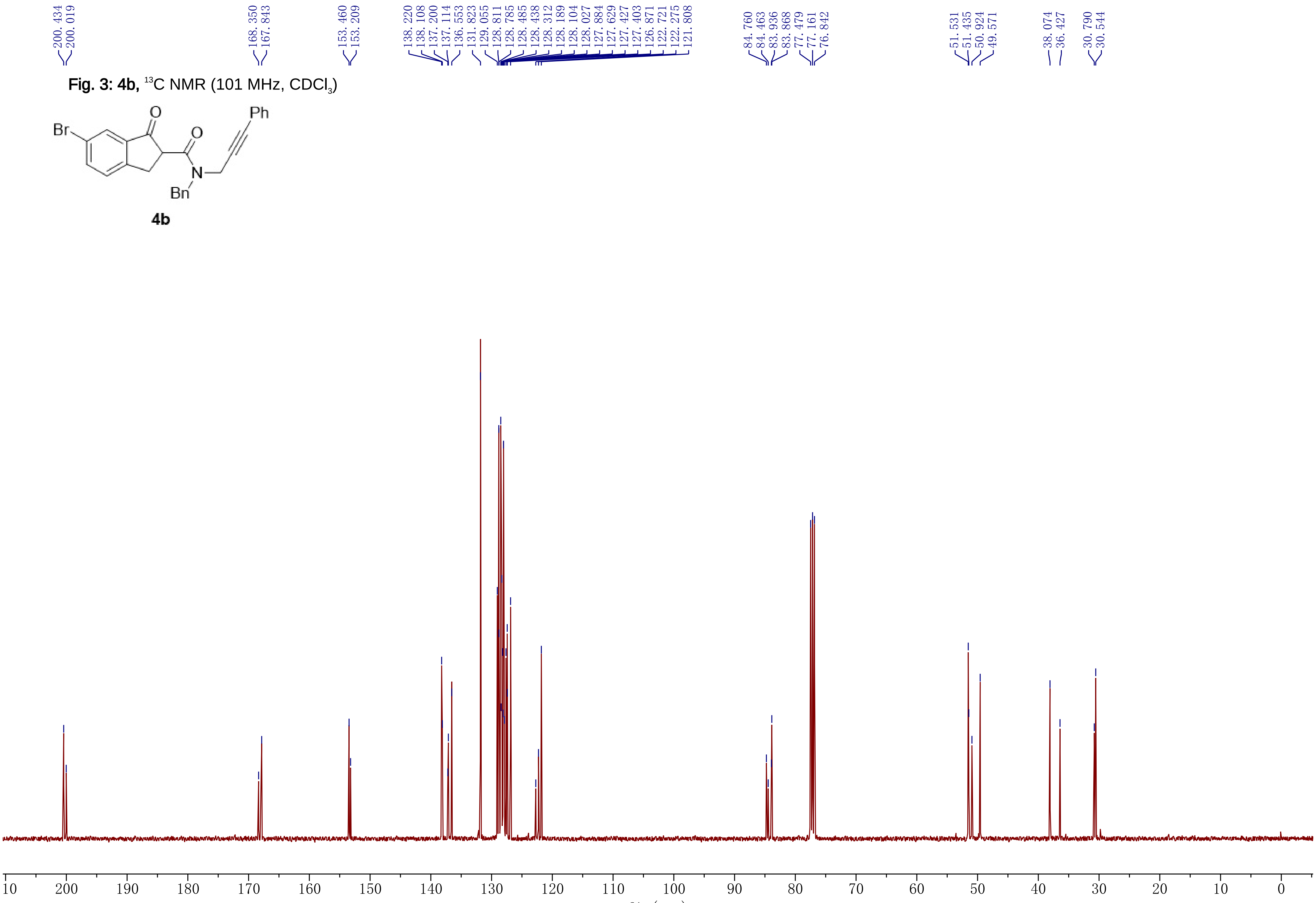

Fig. 3: 4b, ^{13}C NMR (101 MHz, $CDCl_3$)

Fig. 3: 4c, ^{1}H NMR (400 MHz, $CDCl_3$)

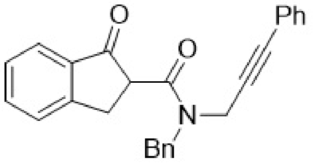

4c

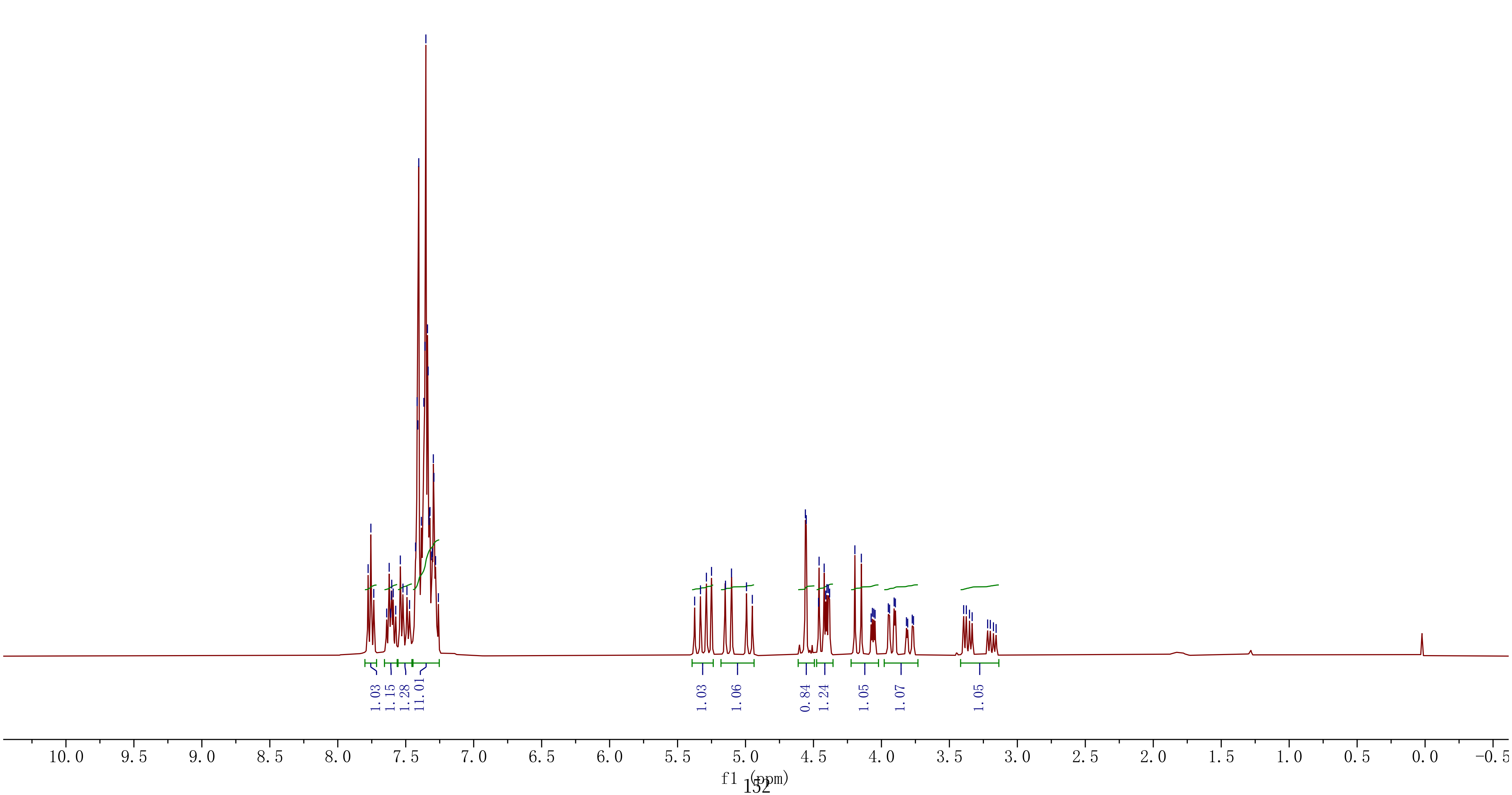

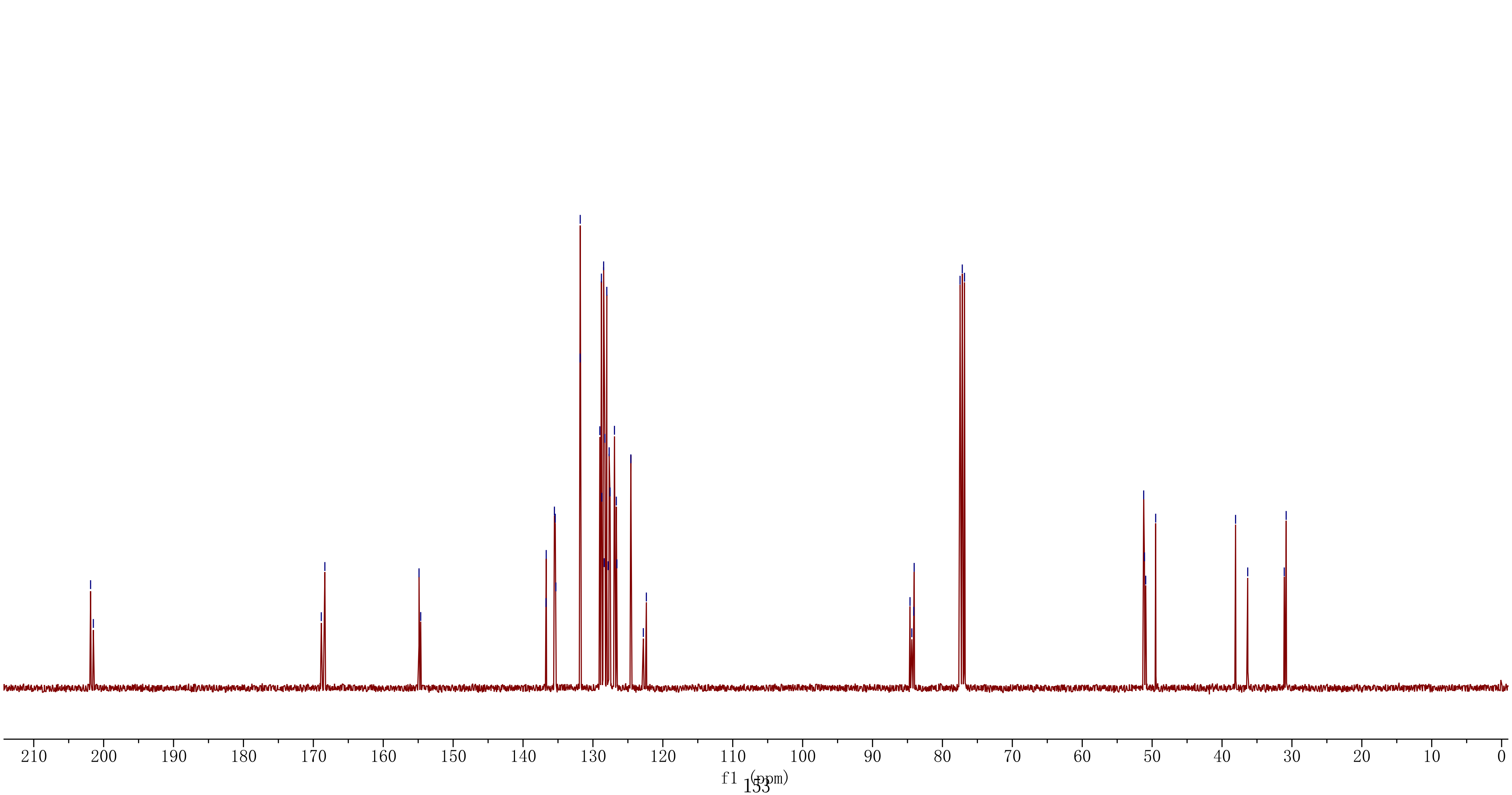

Fig. 3: 4c, ^{13}C NMR (101 MHz, $CDCl_3$)

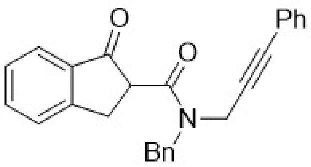

4c

Fig. 3: 4d, ^{1}H NMR (400 MHz, $CDCl_3$)

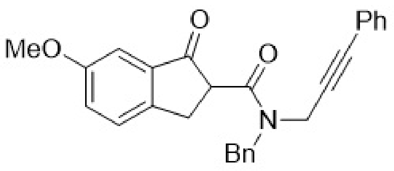

4d

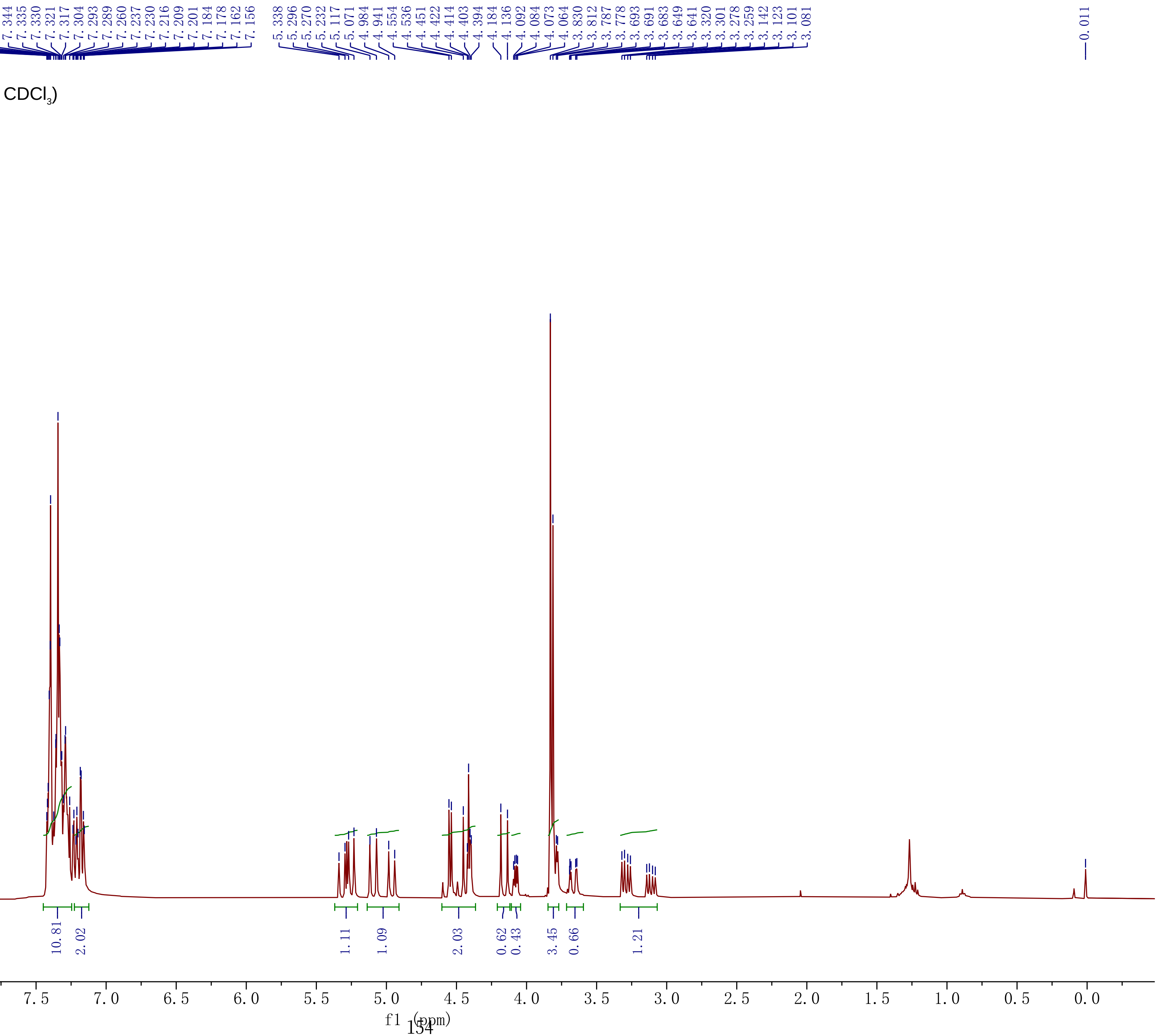

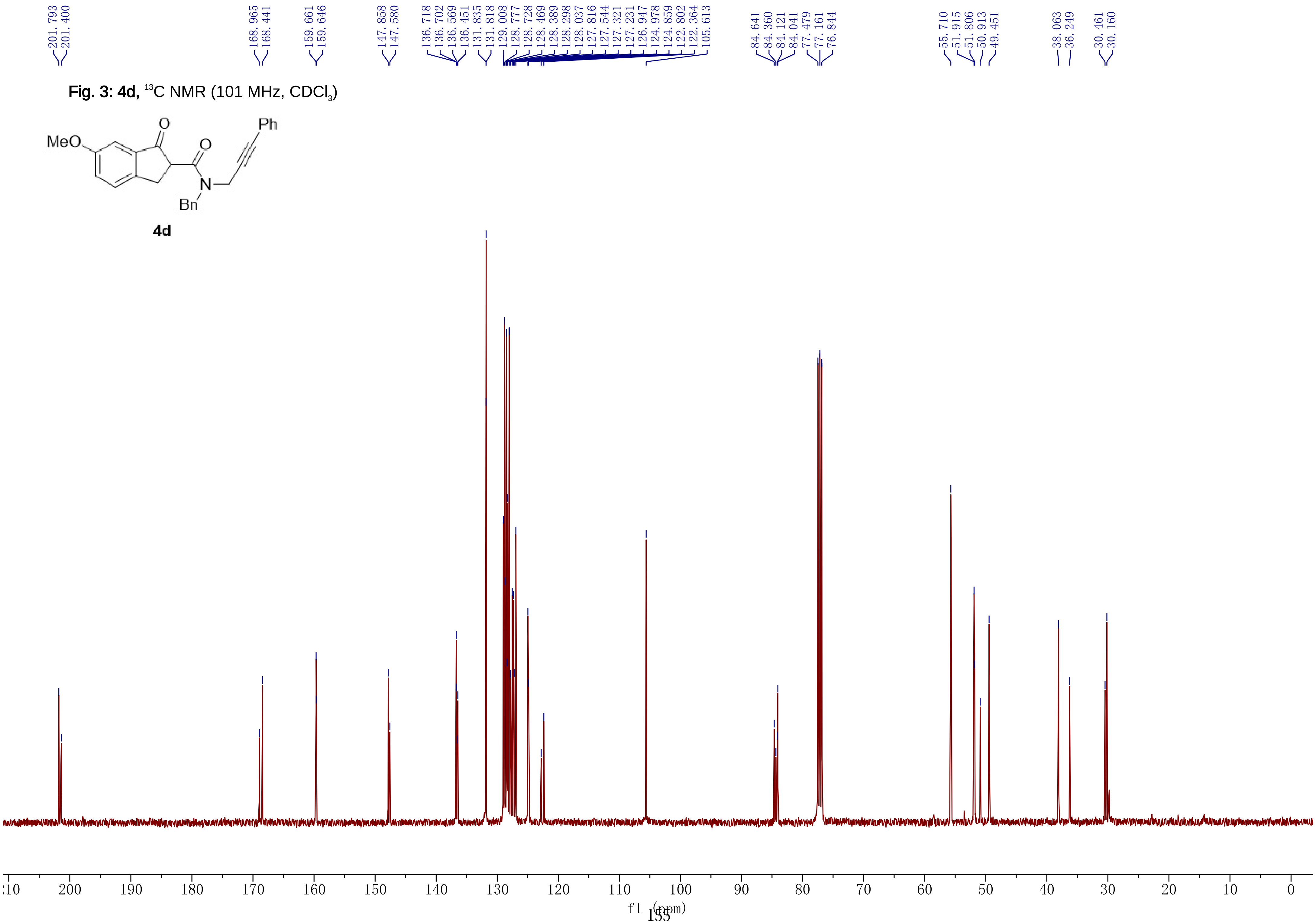

Fig. 3: 4d, ^{13}C NMR (101 MHz, $CDCl_3$)

Fig. 3: 4e, ^{1}H NMR (400 MHz, $CDCl_3$)

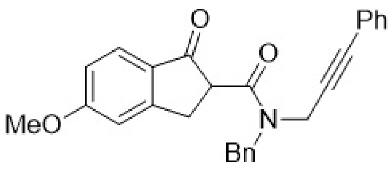

4e

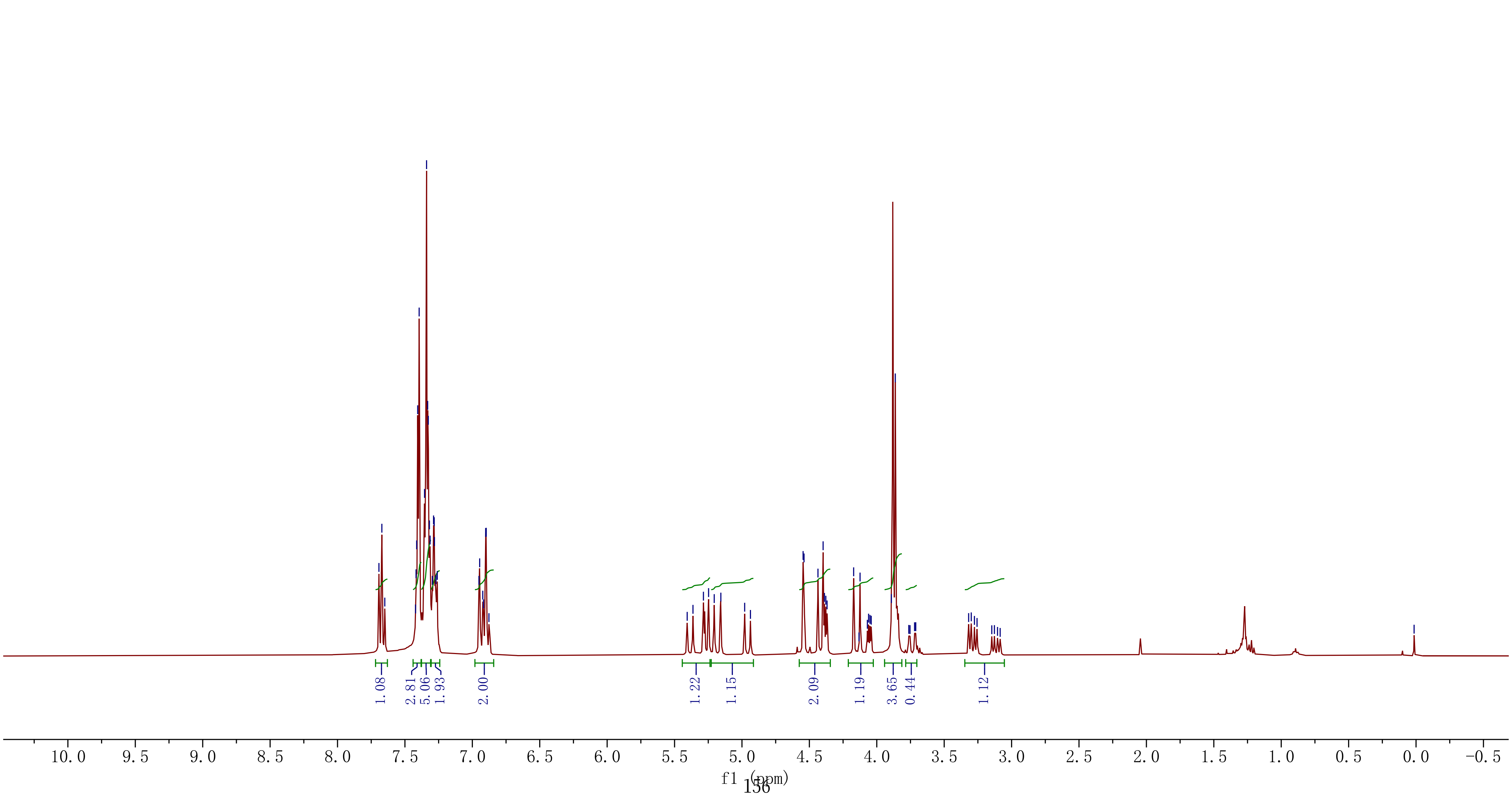

Fig. 3: 4e, ^{13}C NMR (101 MHz, $CDCl_3$)

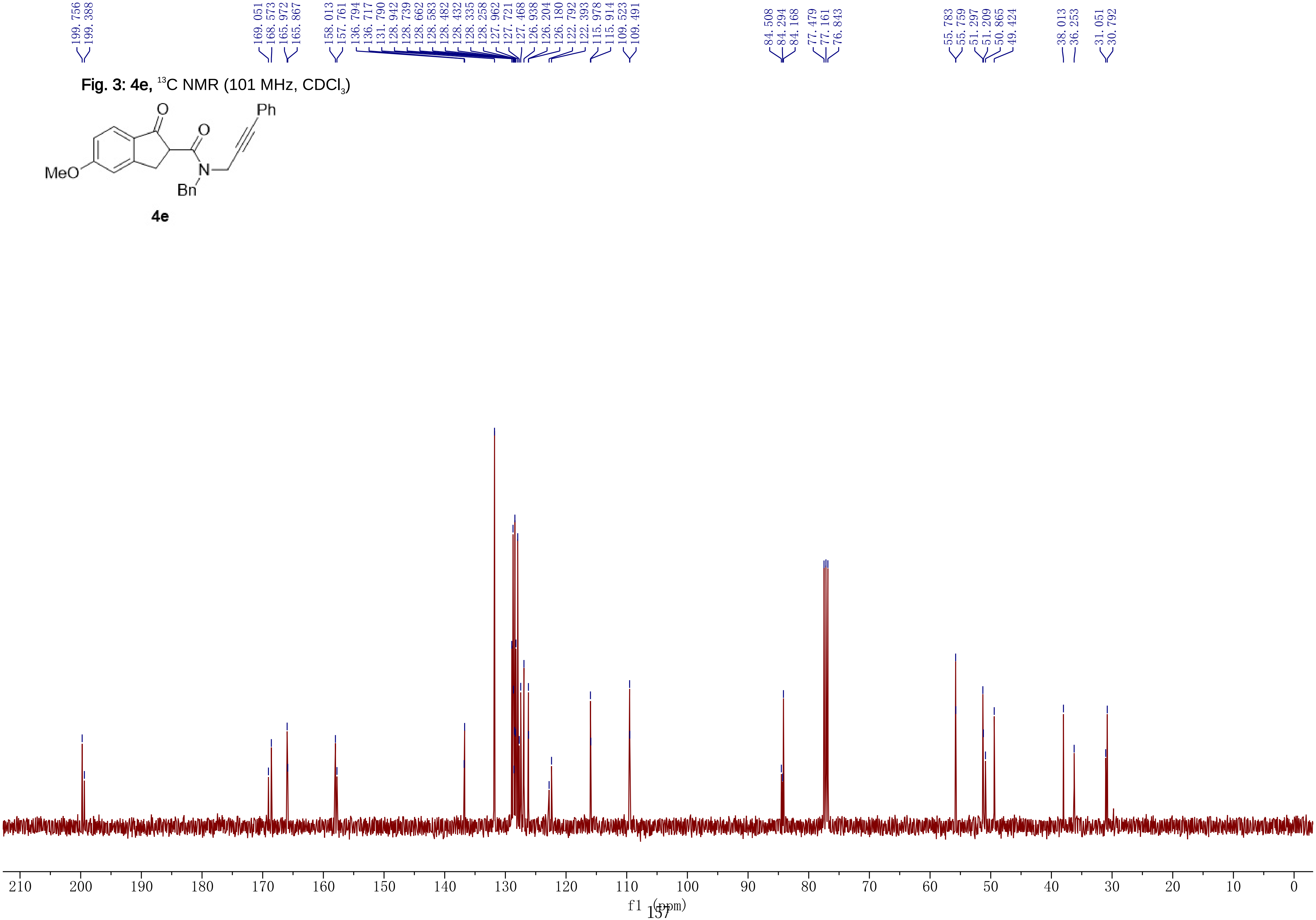

4e

Fig. 3: 4f, ^{1}H NMR (400 MHz, $CDCl_3$)

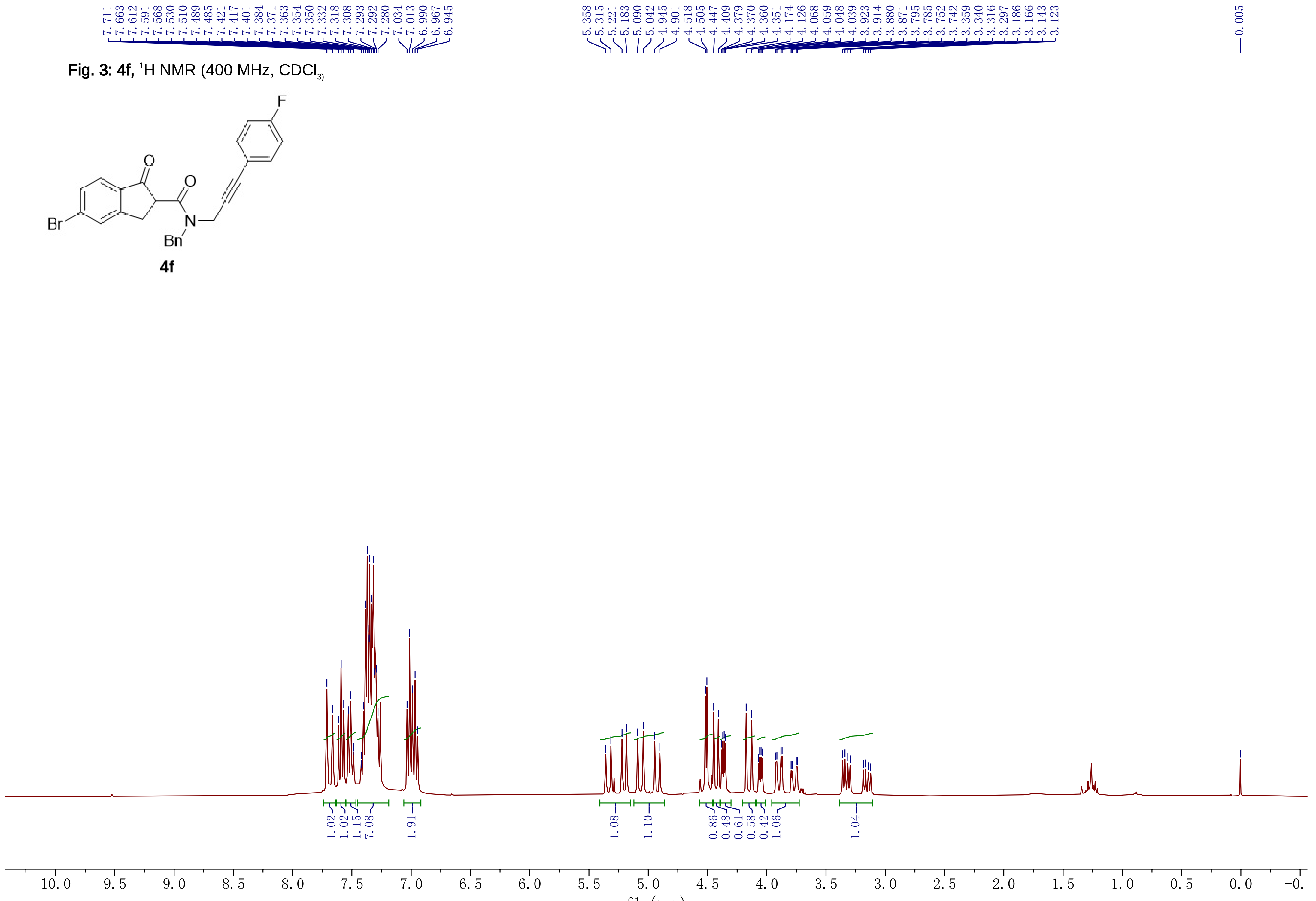

Fig. 3: 4f, ^{19}F NMR (376 MHz, $CDCl_3$)

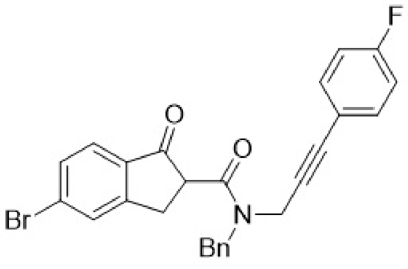

4f

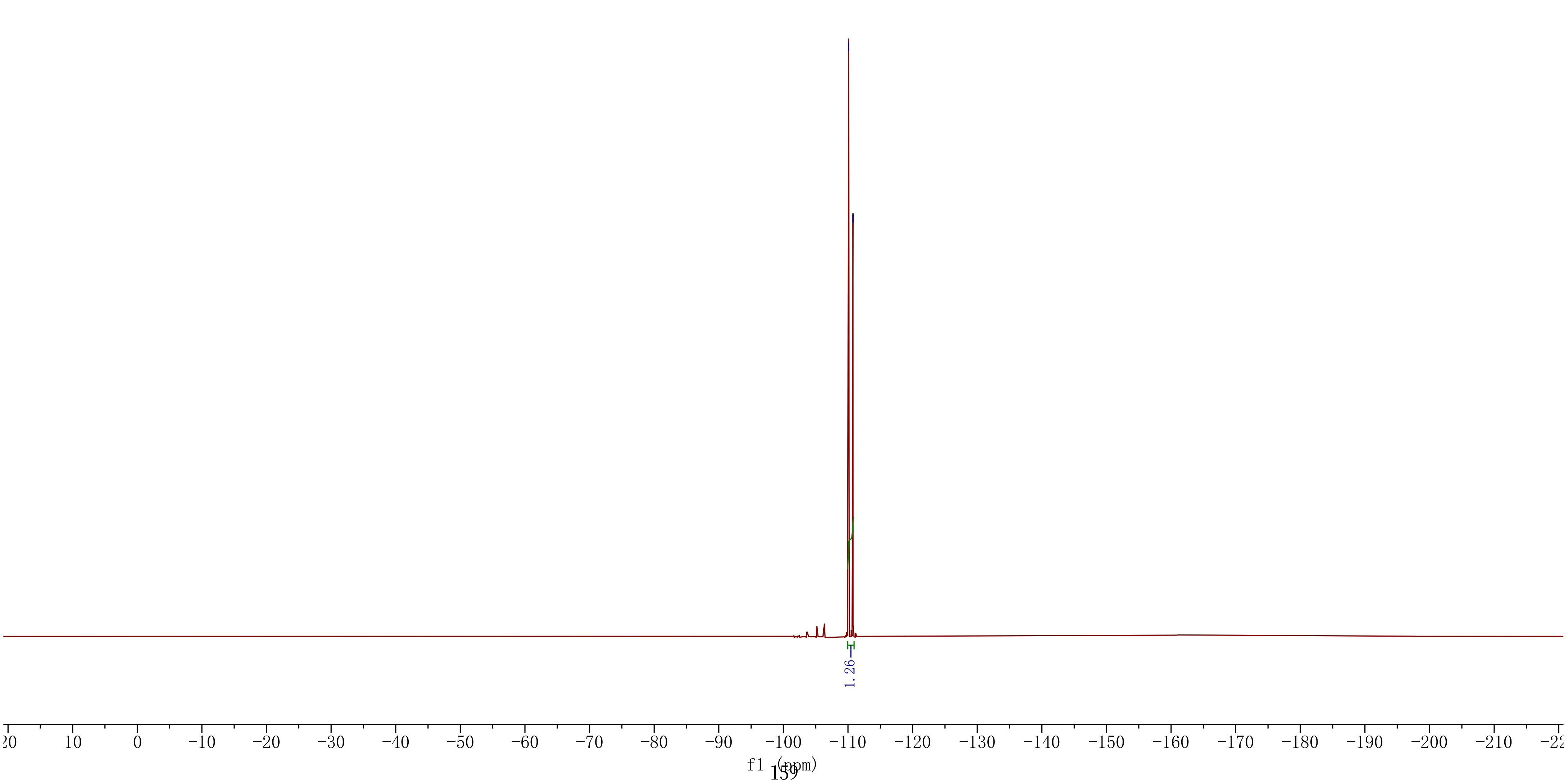

Fig. 3: **4f**, ^{13}C NMR (101 MHz, $CDCl_3$)

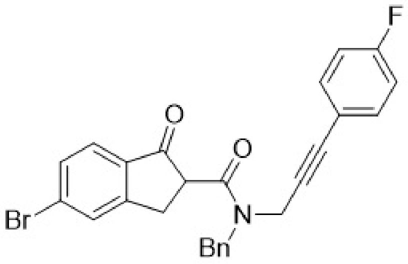

4f

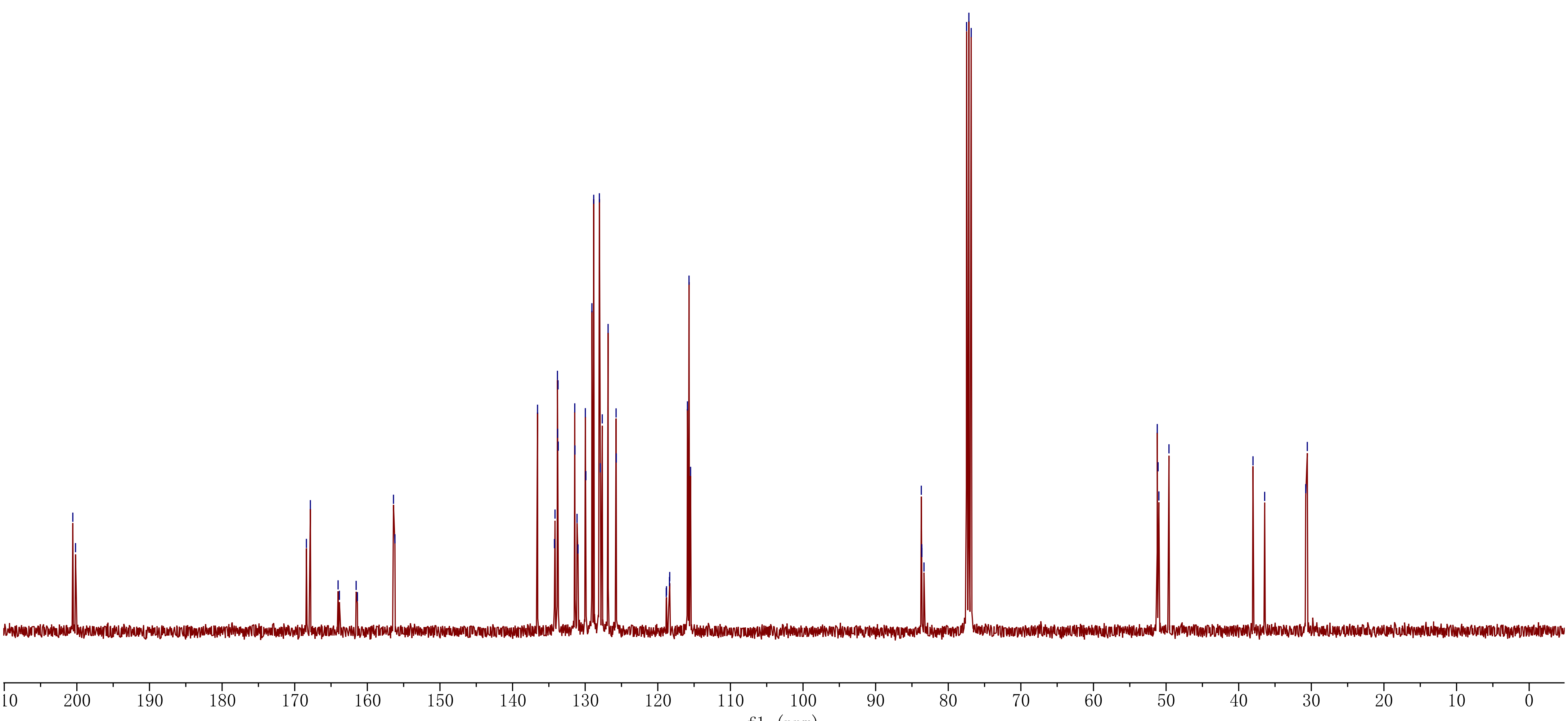

Fig. 3: 4g, ^1H NMR (400 MHz, $CDCl_3$)

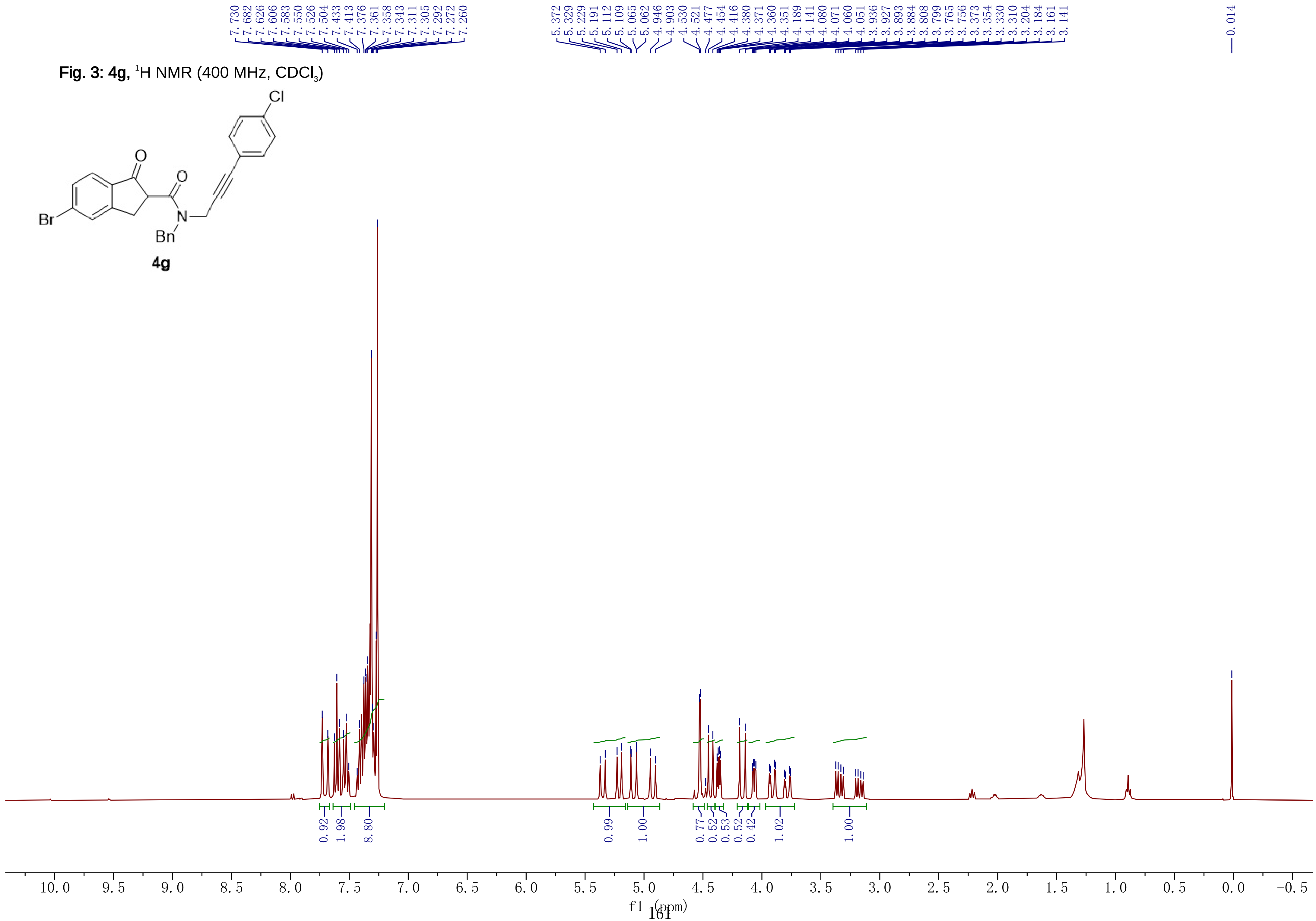

Fig. 3: 4g, ^{13}C NMR (101 MHz, $CDCl_3$)

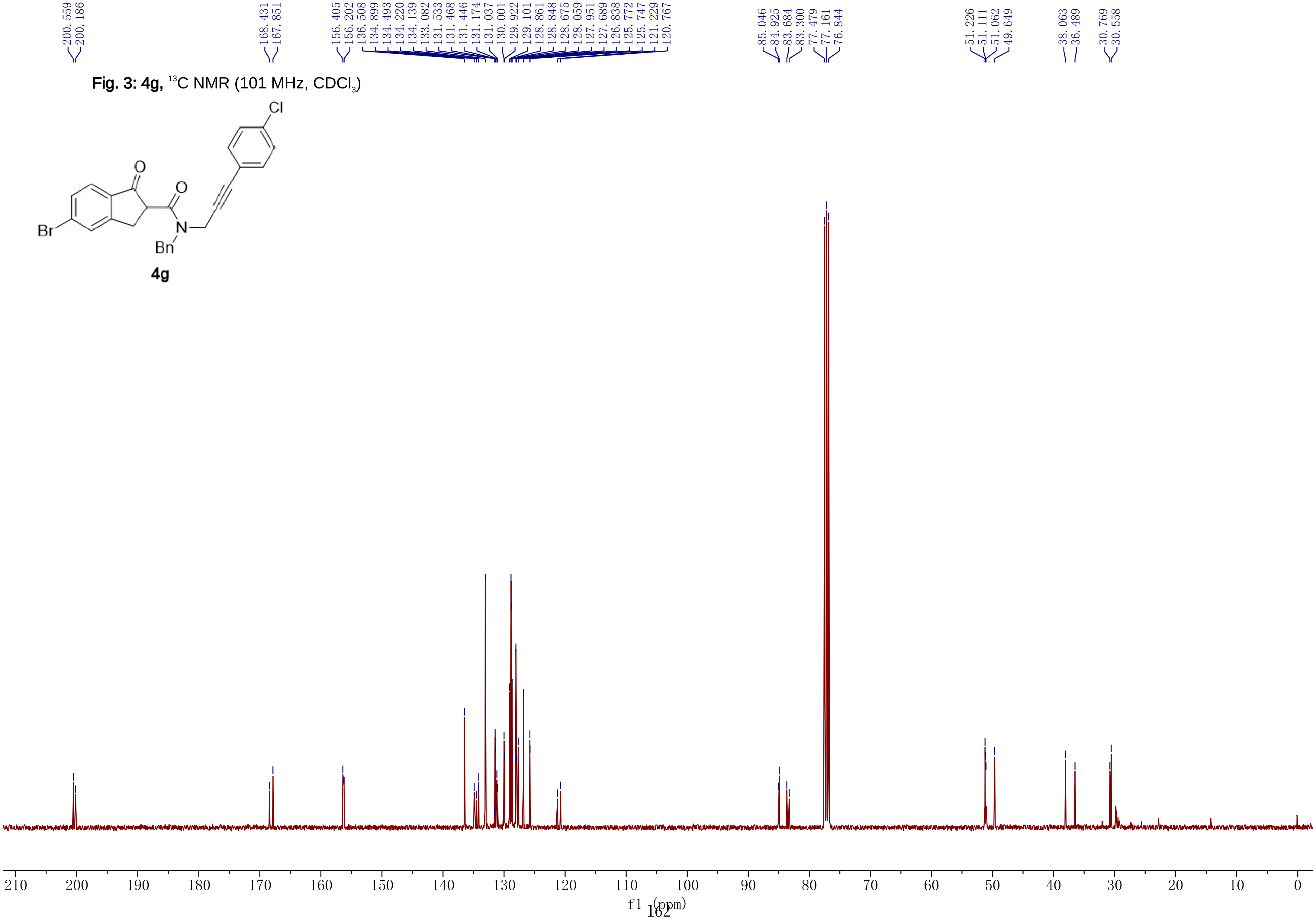

Fig. 3: 4h, ¹H NMR (400 MHz, $CDCl_3$)

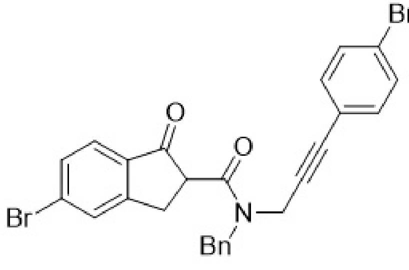

4h

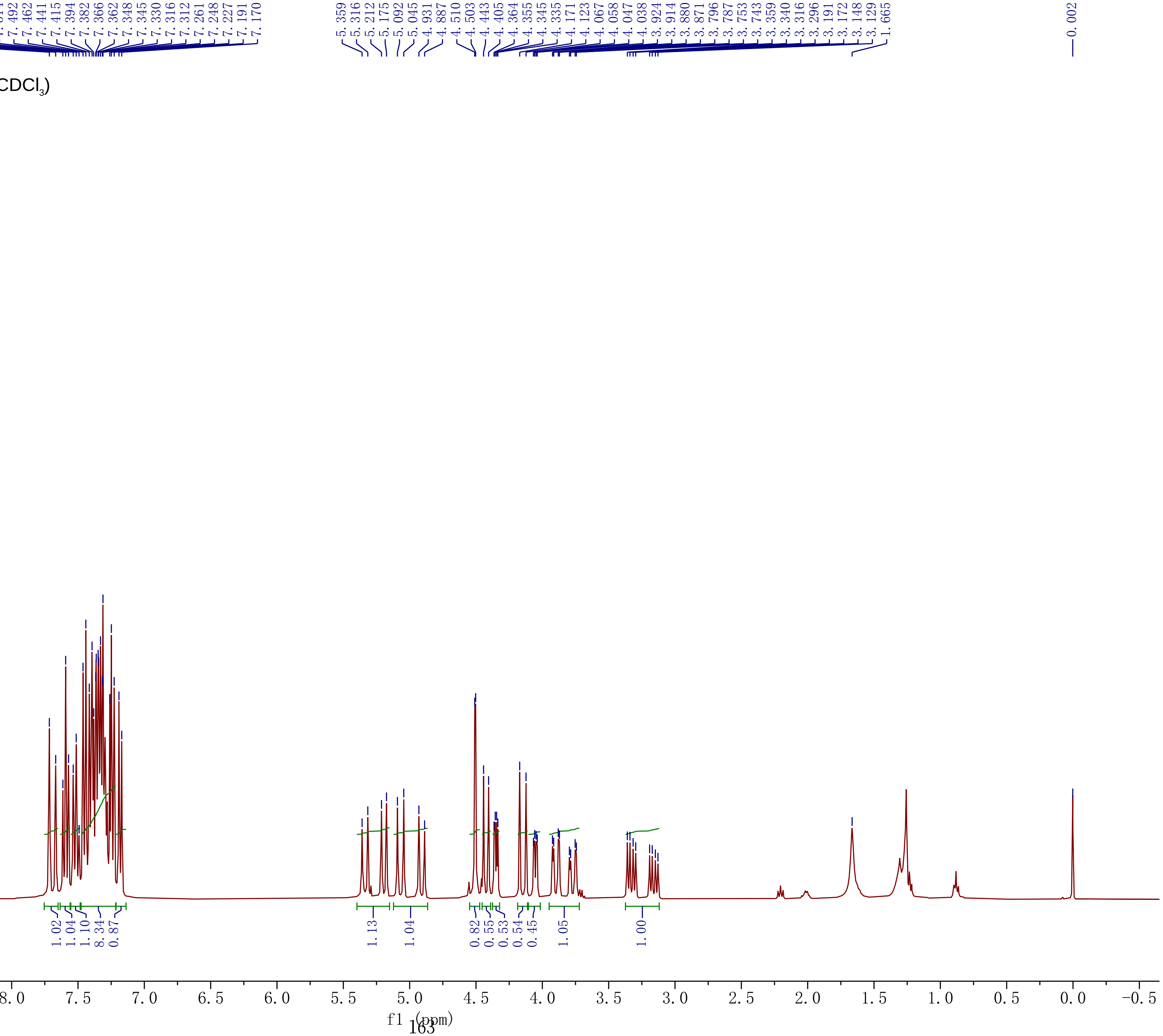

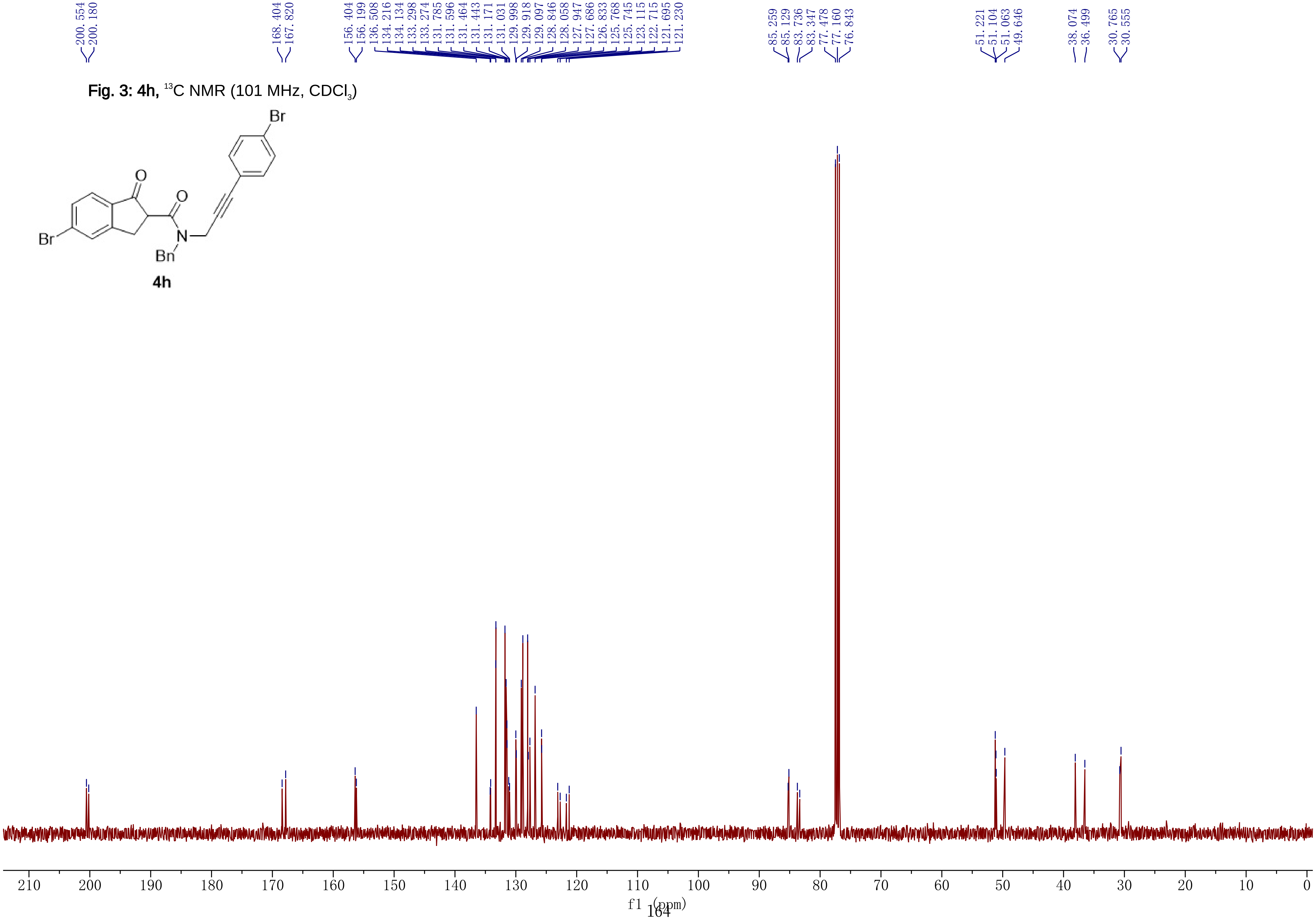

Fig. 3: 4h, ^{13}C NMR (101 MHz, $CDCl_3$)

4h

Fig. 3: **4i,** ^{1}H NMR (400 MHz, $CDCl_3$)

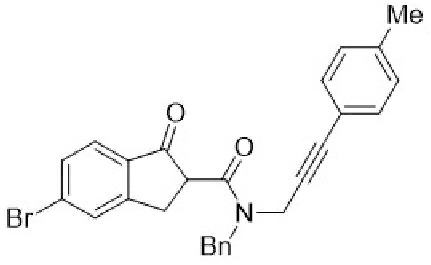

4i

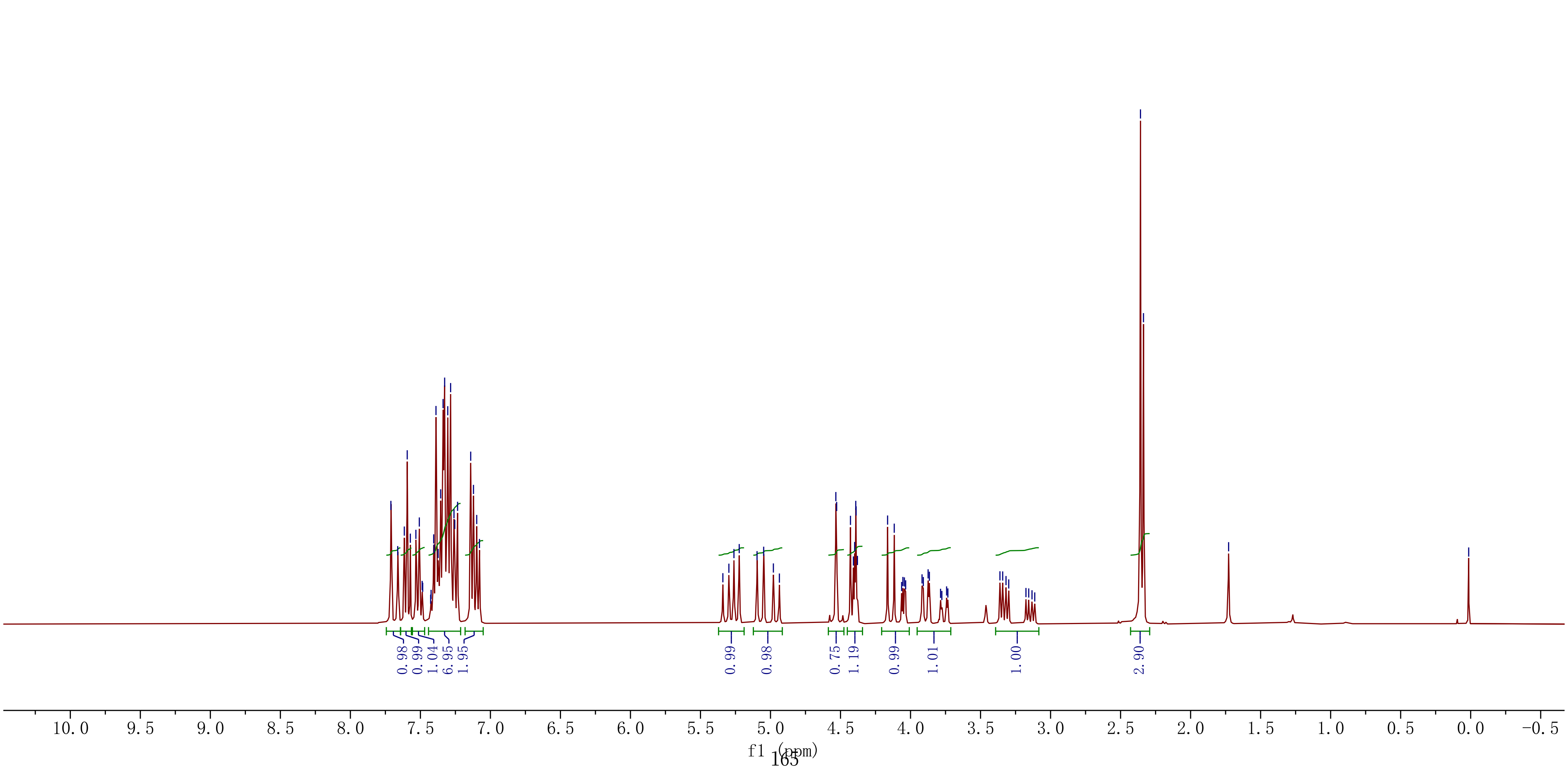

Fig. 3: 4i, ^{13}C NMR (101 MHz, $CDCl_3$)

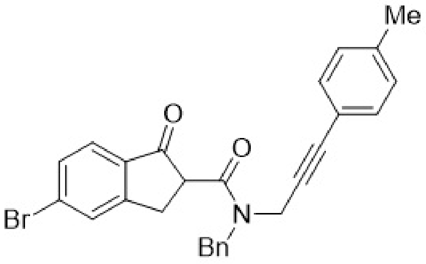

4i

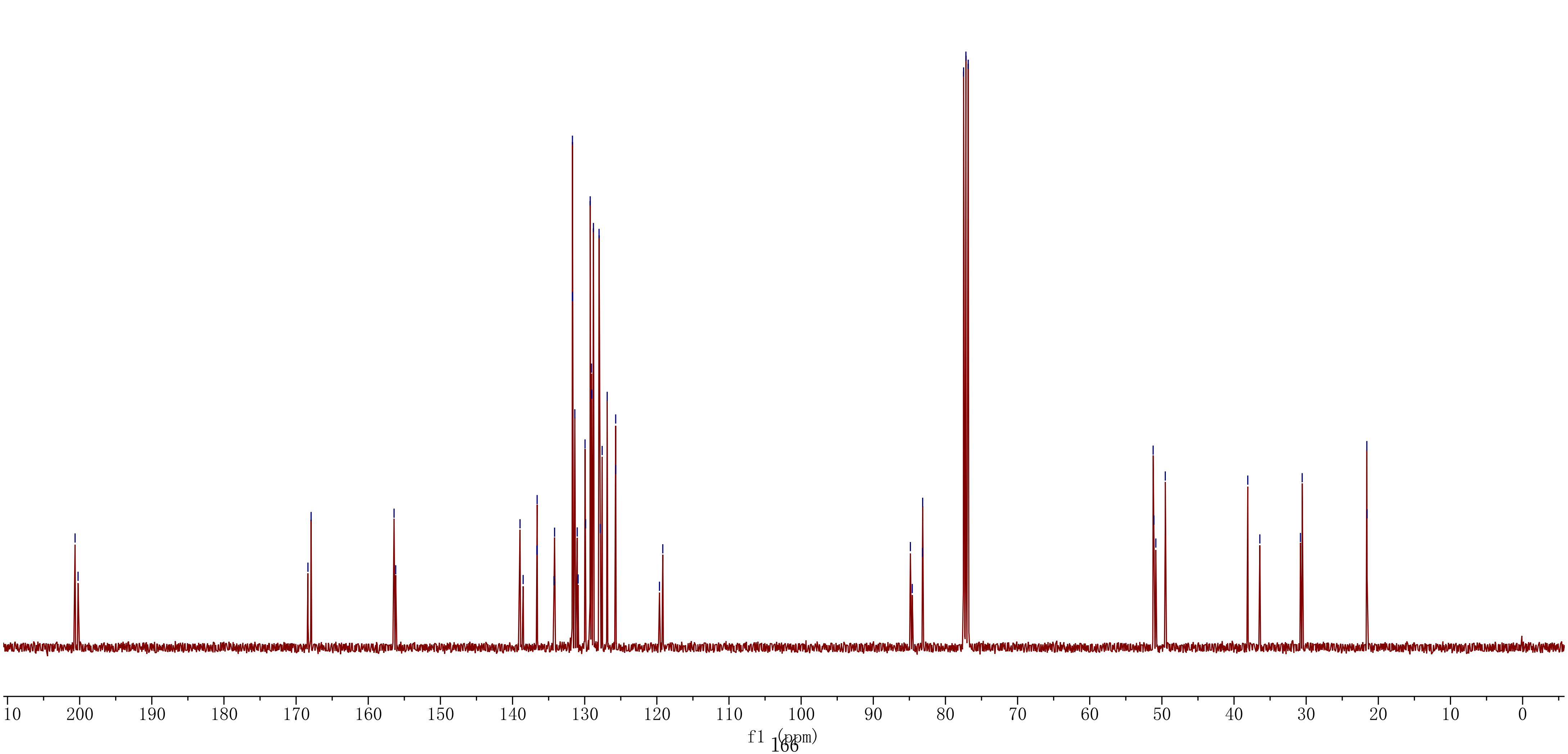

Fig. 3: 4j, ^{1}H NMR (400 MHz, $CDCl_3$)

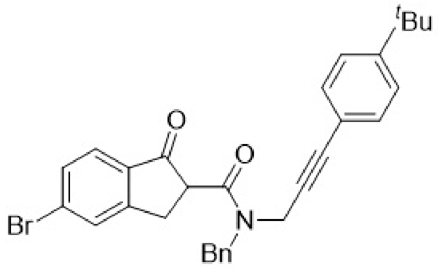

4j

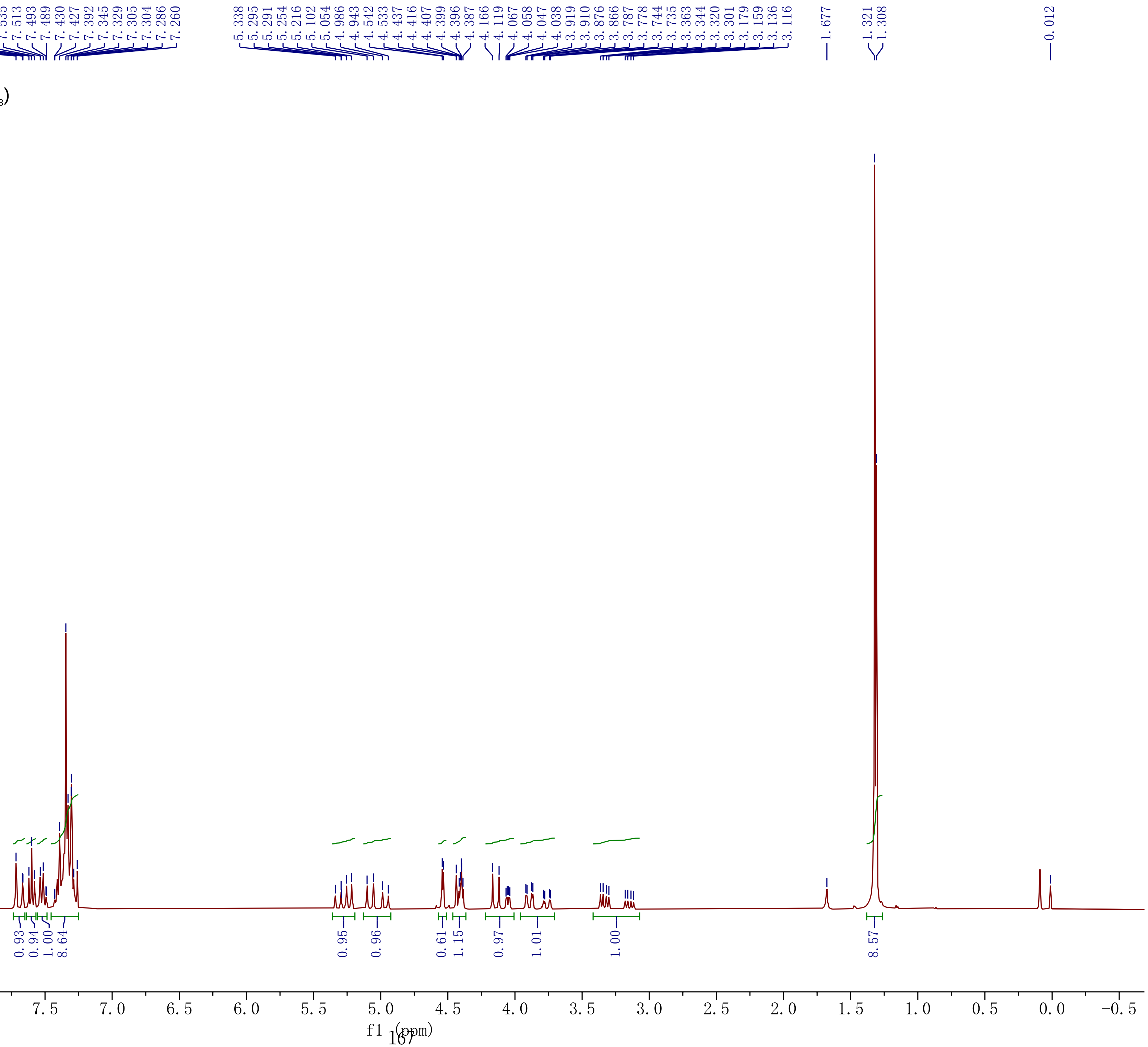

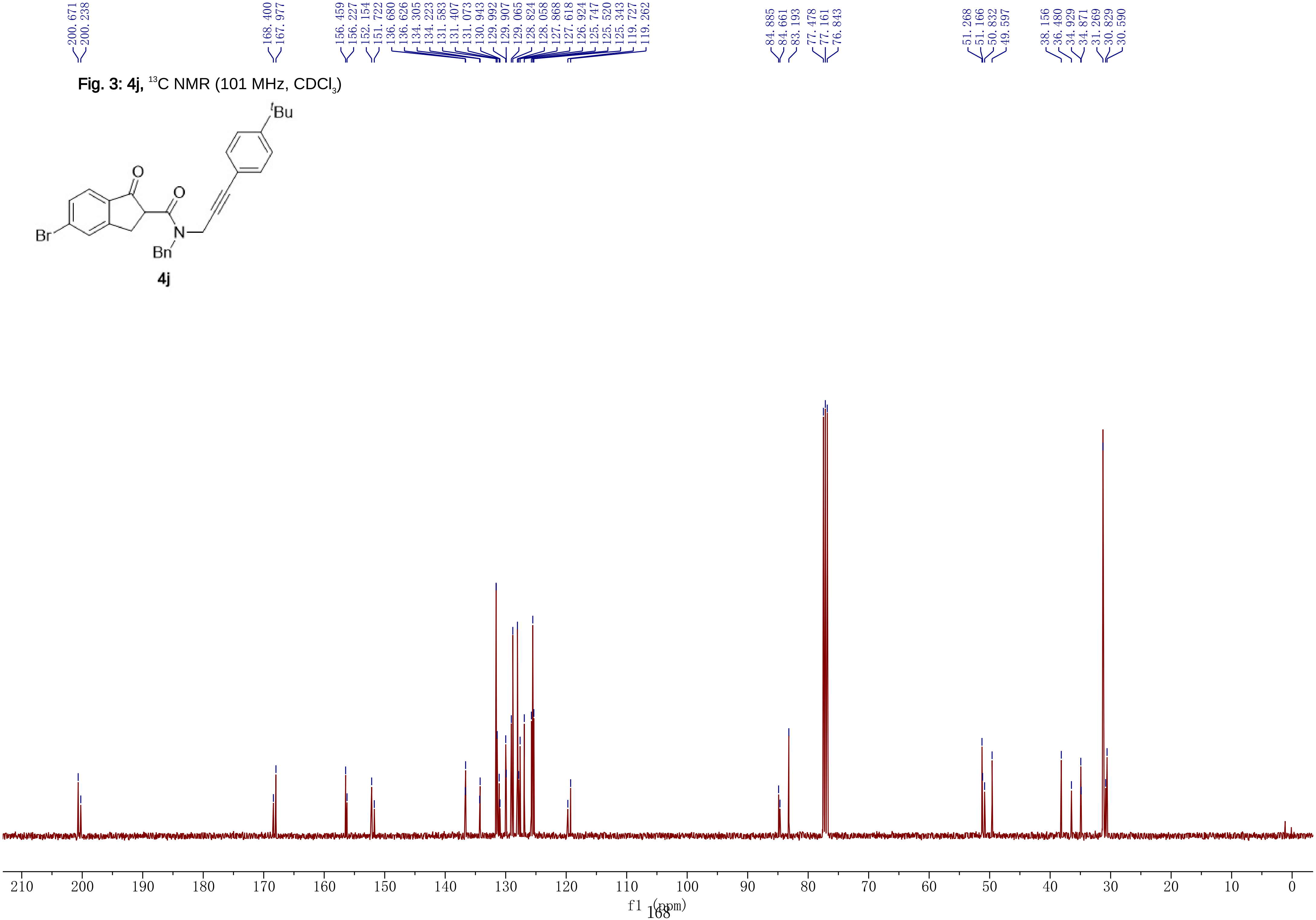

4j

Fig. 3: 4j, ^{13}C NMR (101 MHz, $CDCl_3$)

Fig. 3: 4k, ^{1}H NMR (400 MHz, $CDCl_3$)

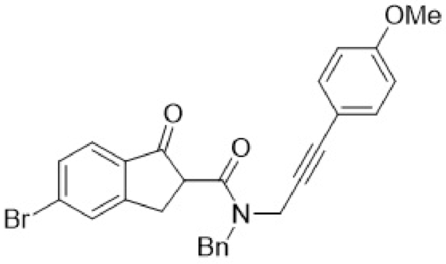

4k

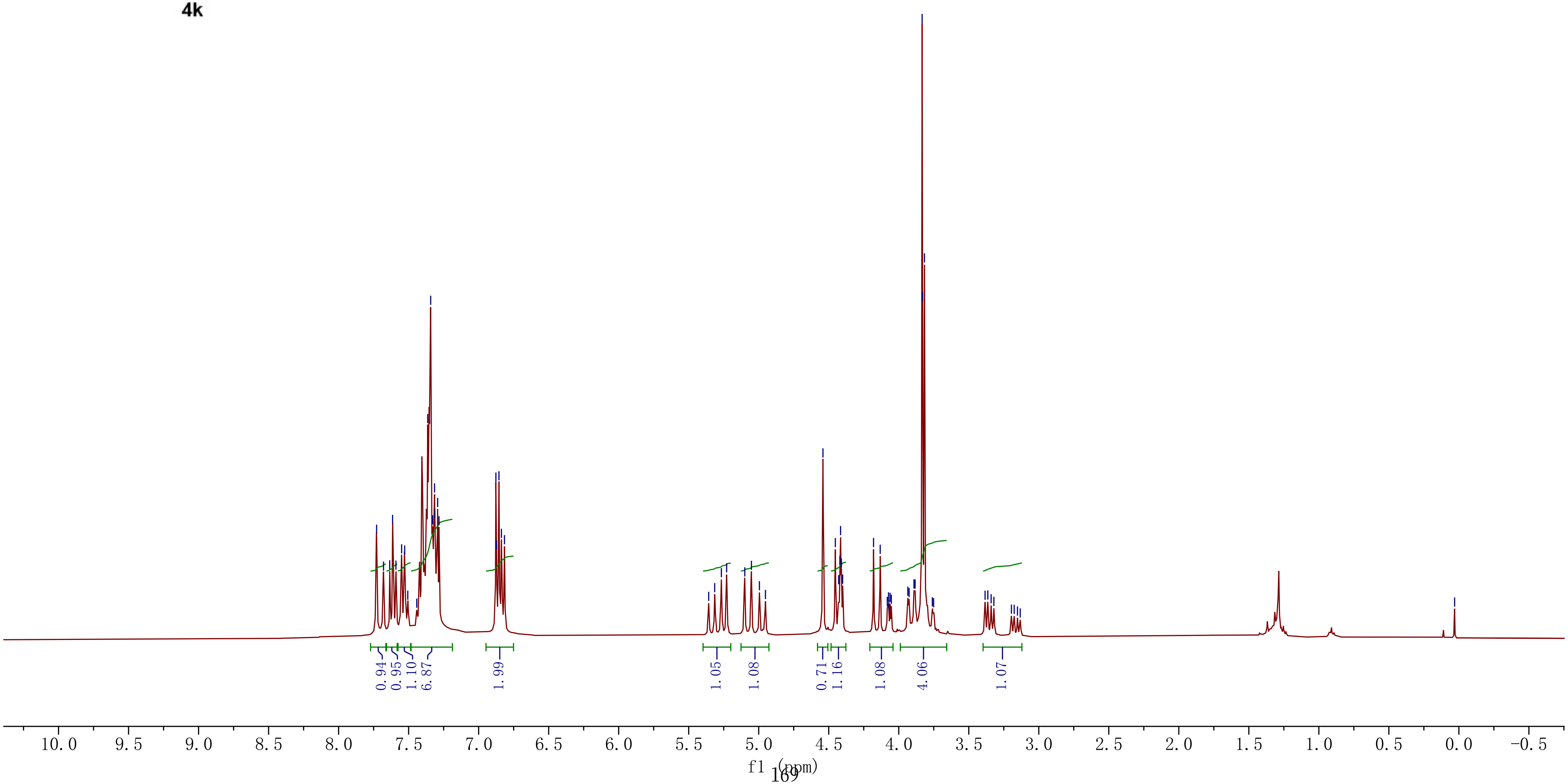

Fig. 3: 4k, ^{13}C NMR (101 MHz, $CDCl_3$)

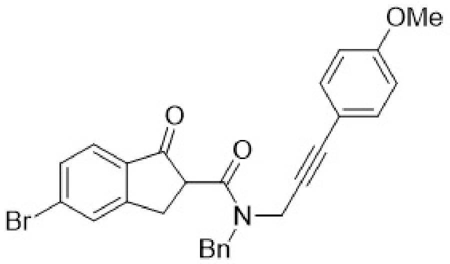

4k

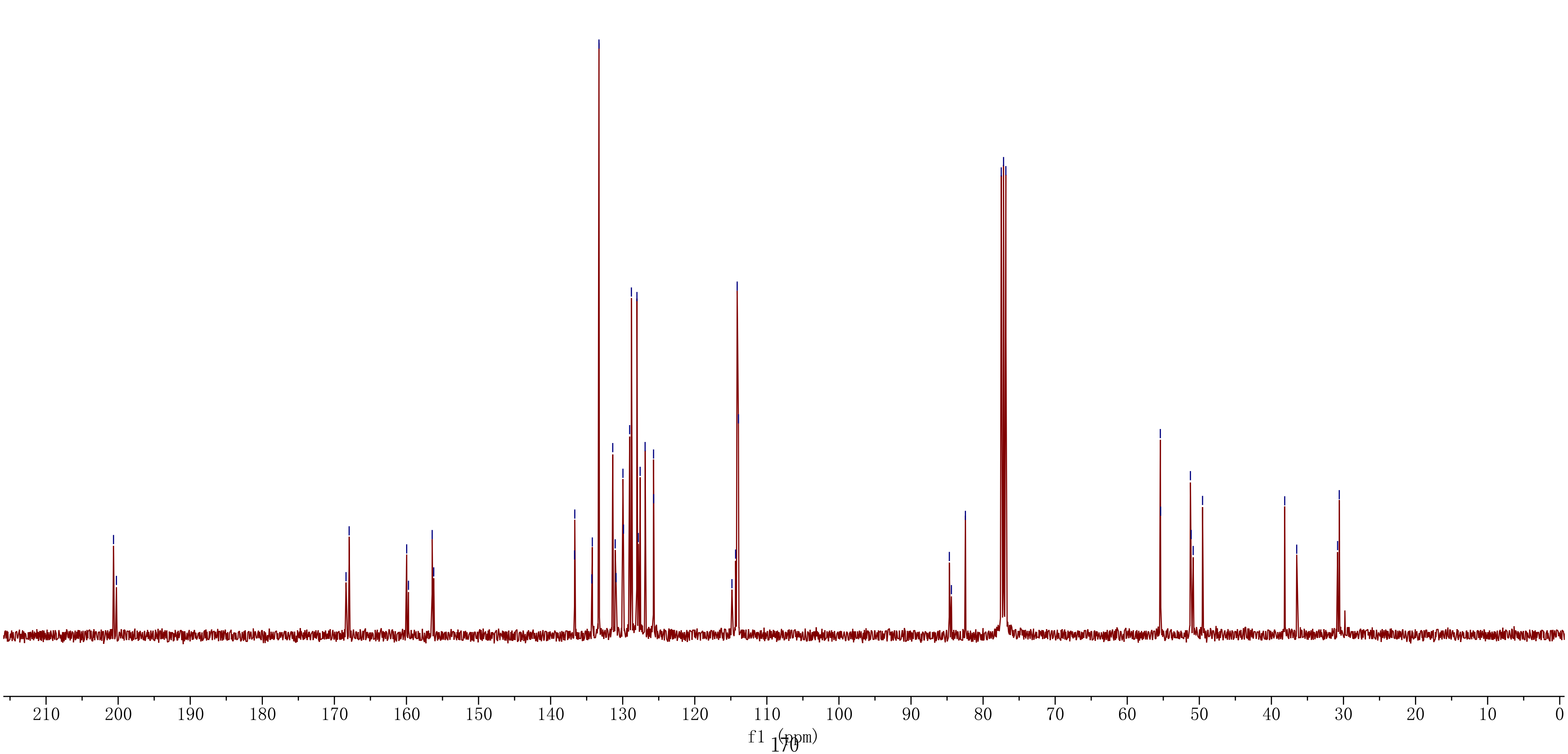

Fig. 3: 4l, ^{1}H NMR (400 MHz, $CDCl_3$)

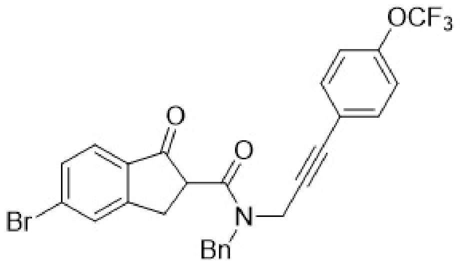

4l

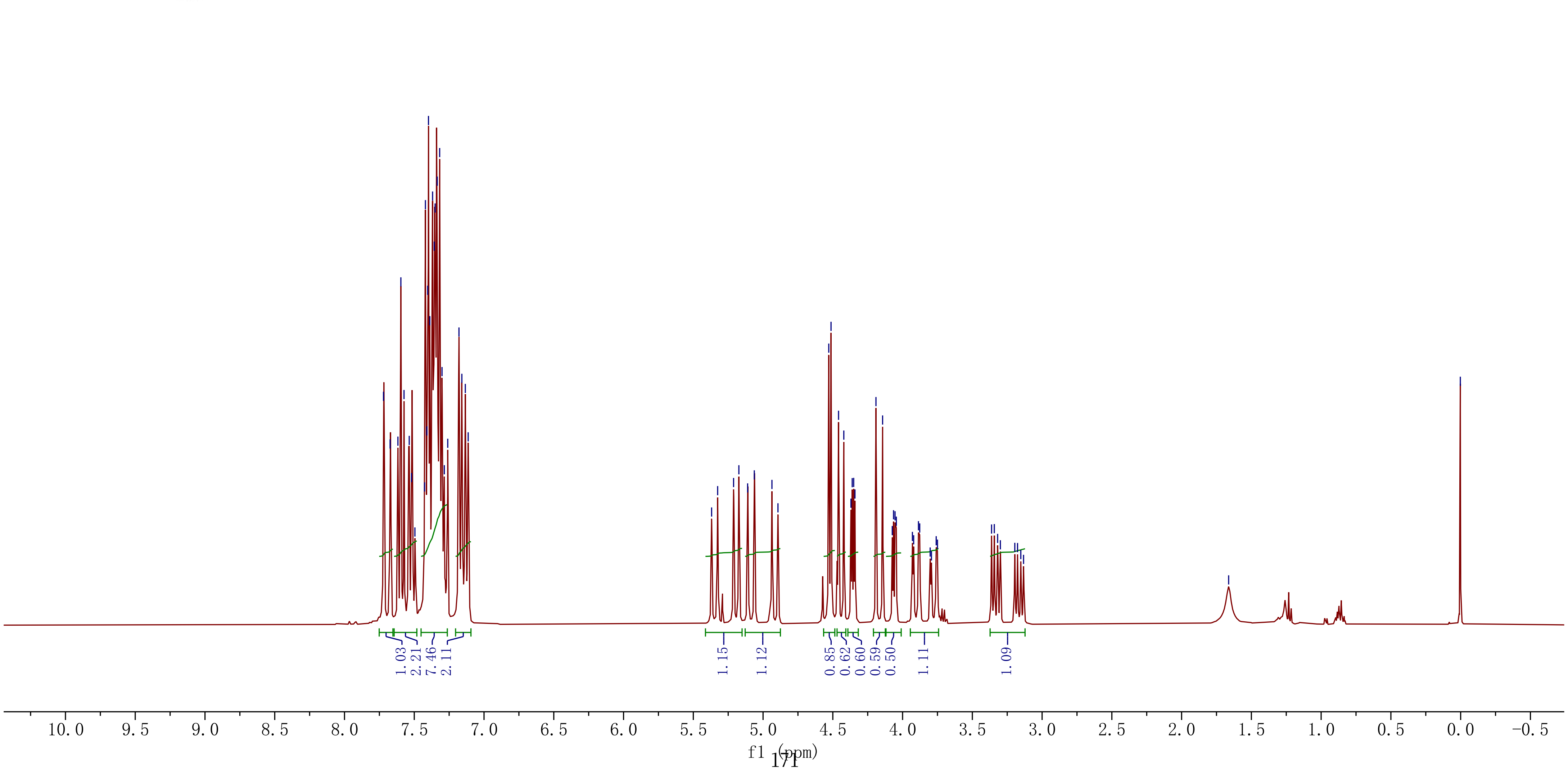

Fig. 3: 4l, ^{19}F NMR (376 MHz, $CDCl_3$)

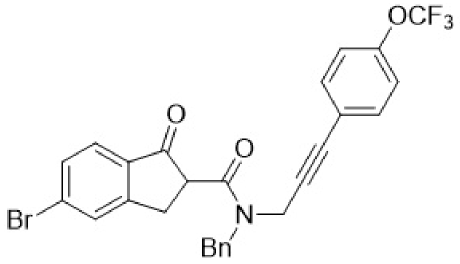

4l

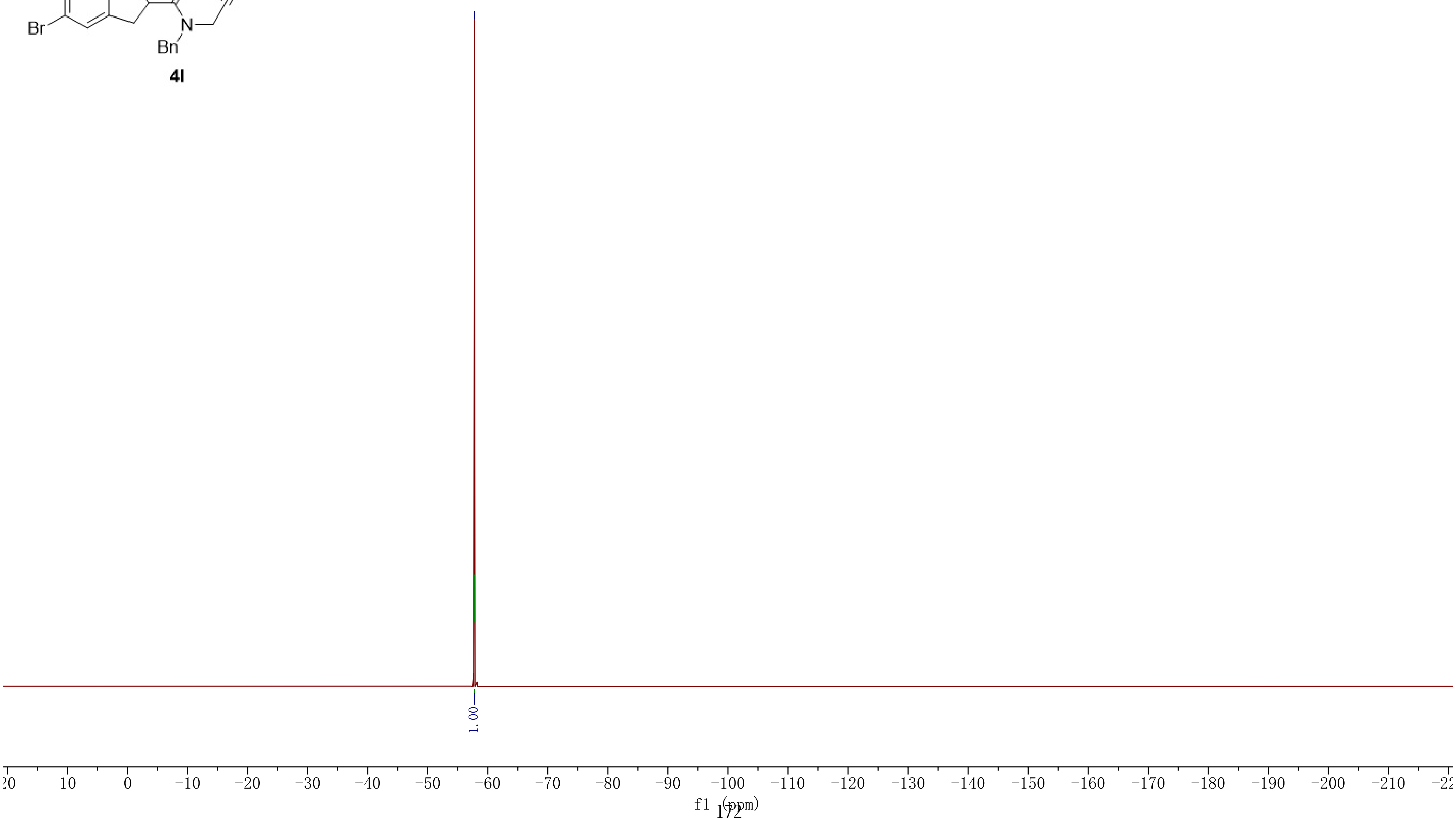

Fig. 3: 4l, ^{13}C NMR (101 MHz, $CDCl_3$)

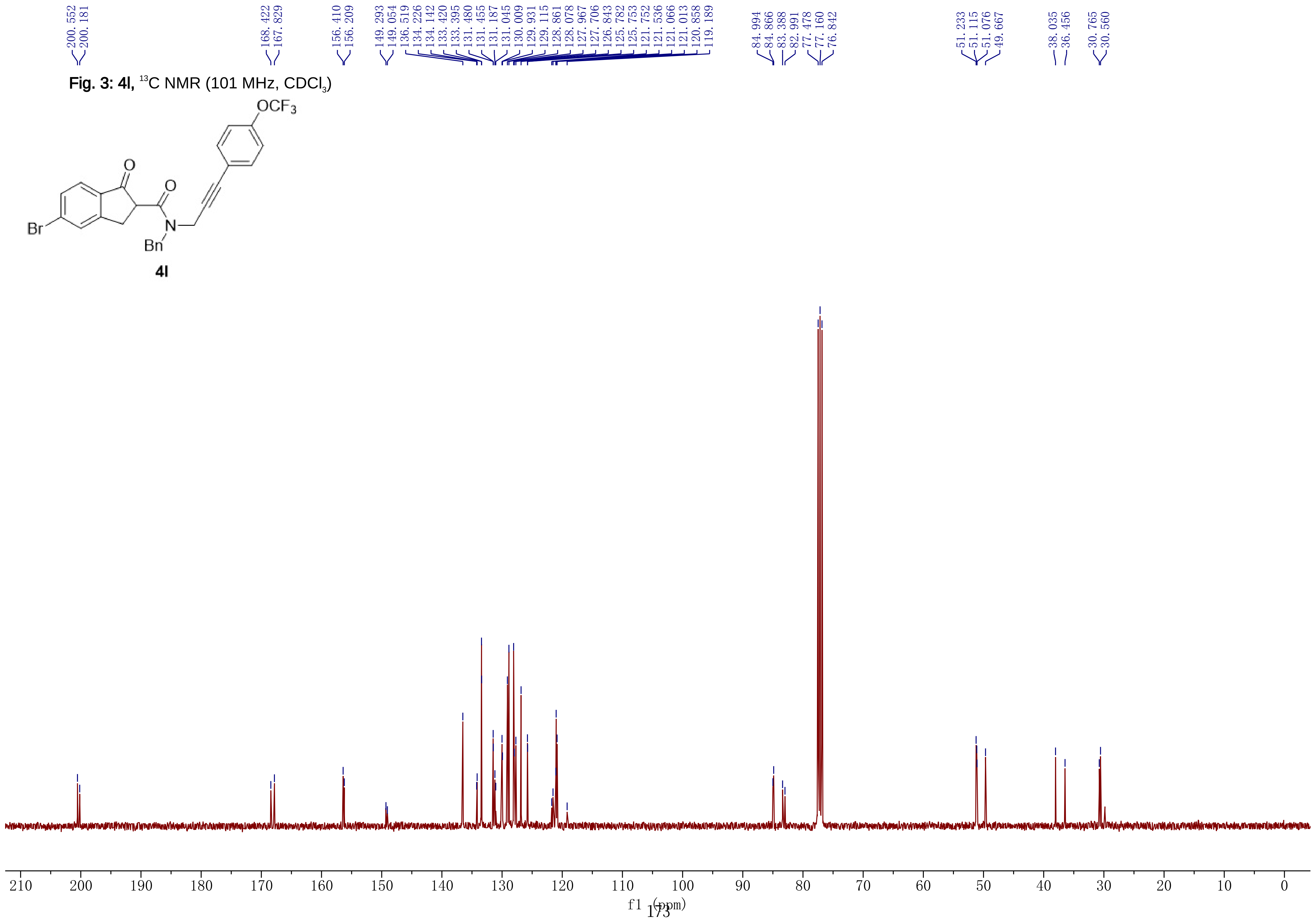

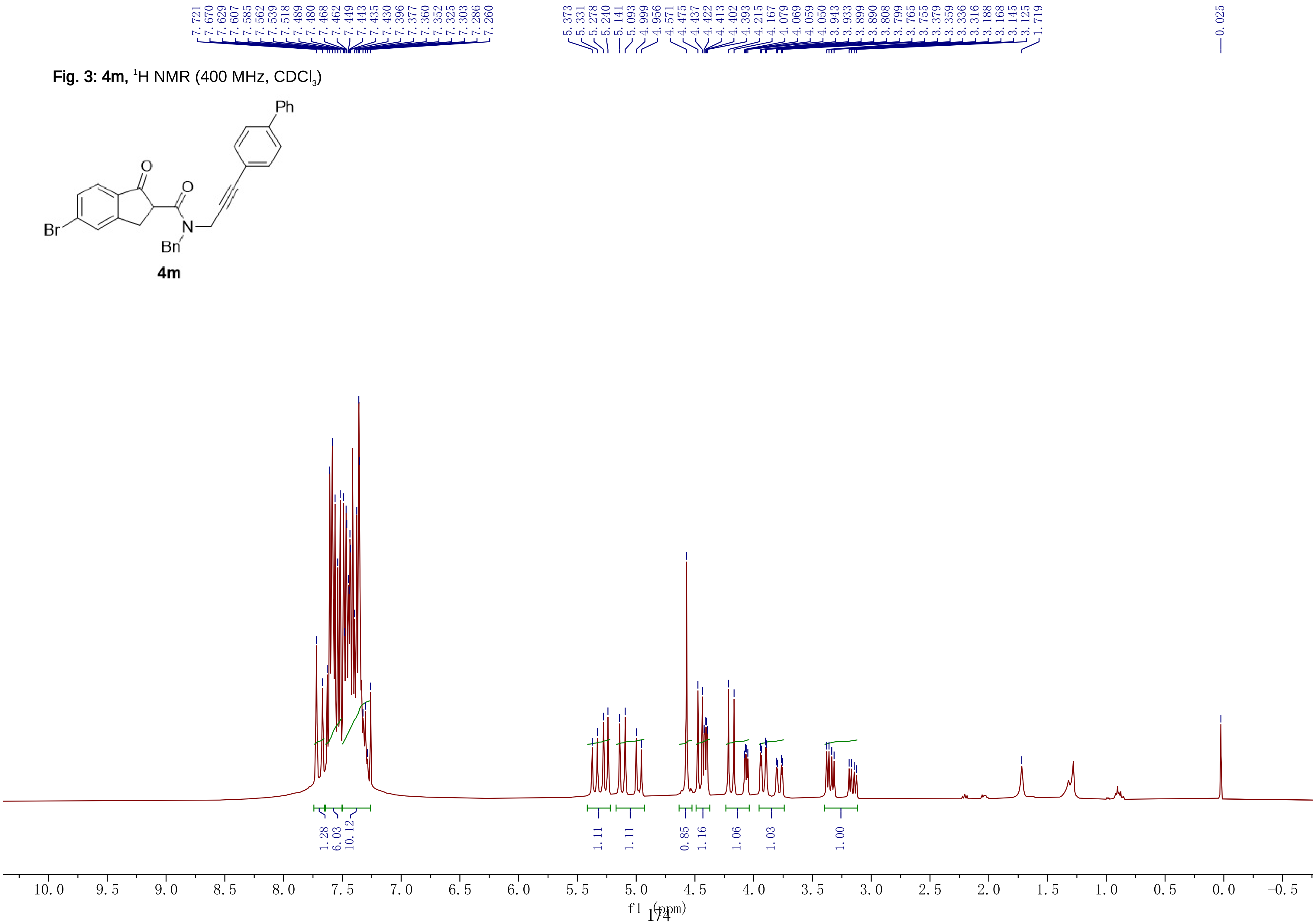

Fig. 3: 4m, ^1H NMR (400 MHz, $CDCl_3$)

4m

Fig. 3: 4m, ^{13}C NMR (101 MHz, $CDCl_3$)

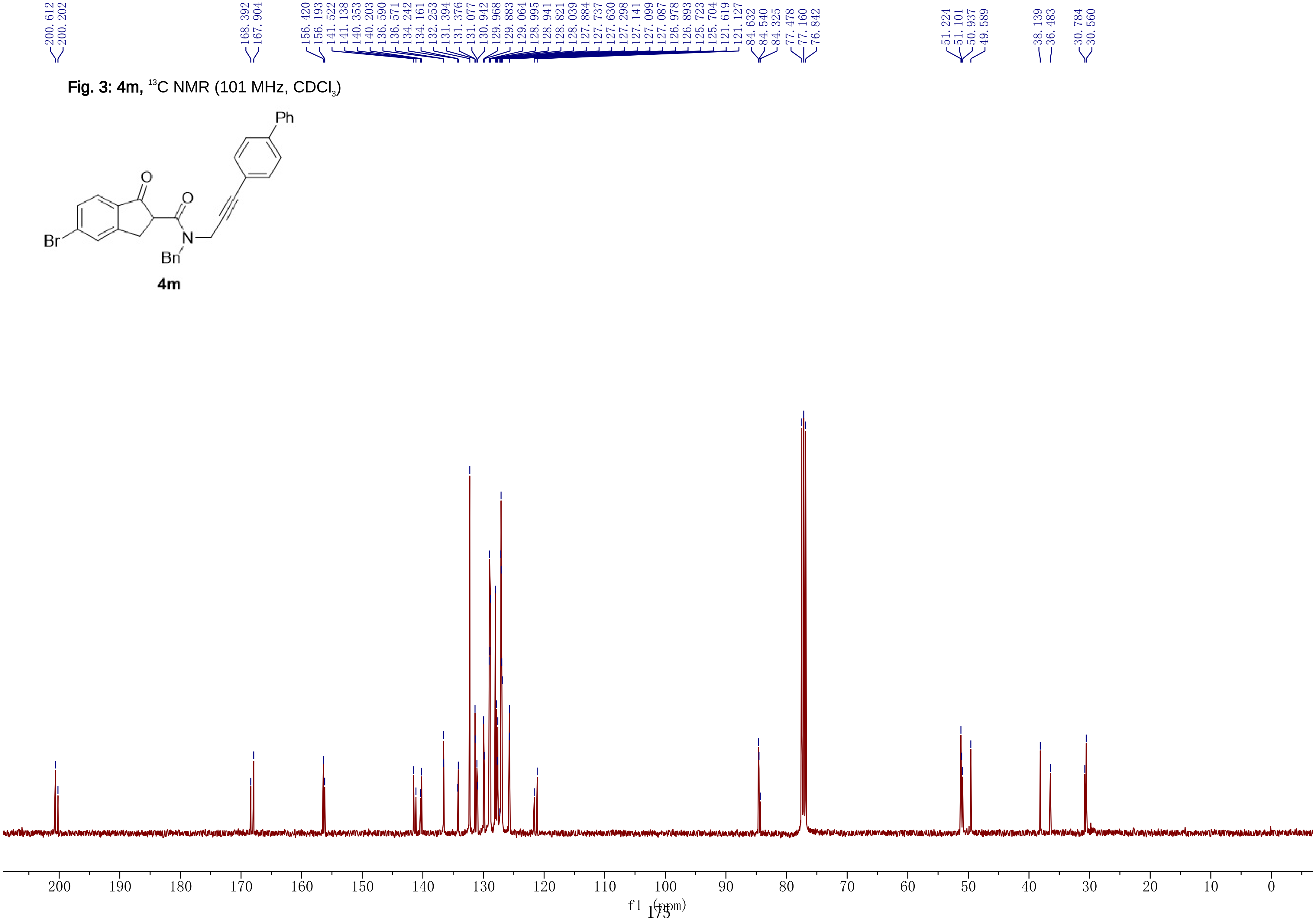

Fig. 3: 4n, ^{1}H NMR (400 MHz, $CDCl_3$)

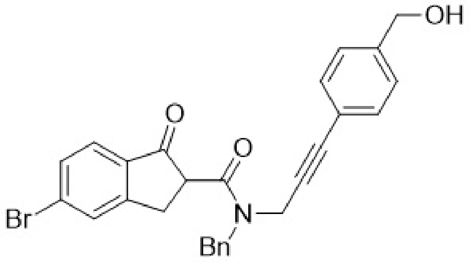

4n

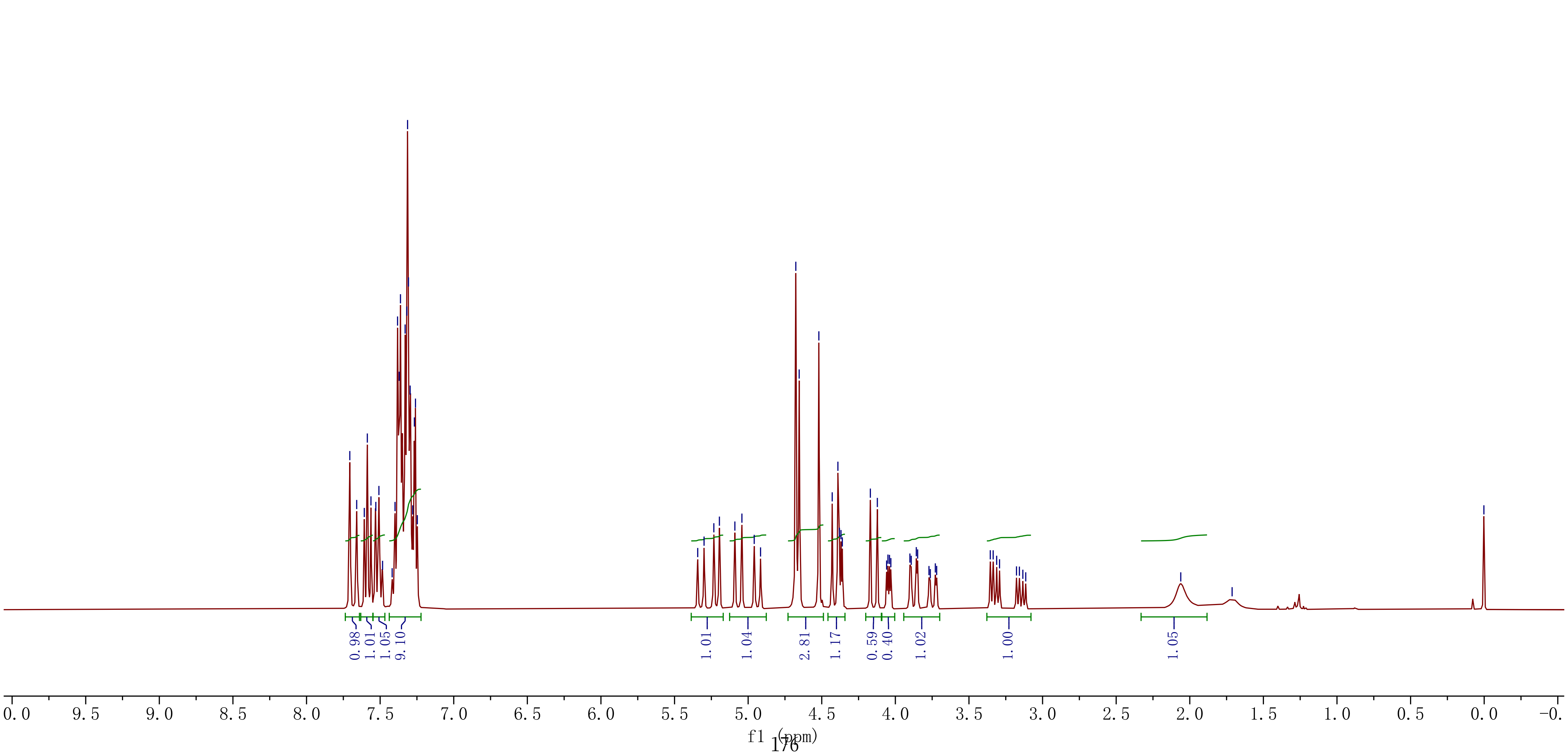

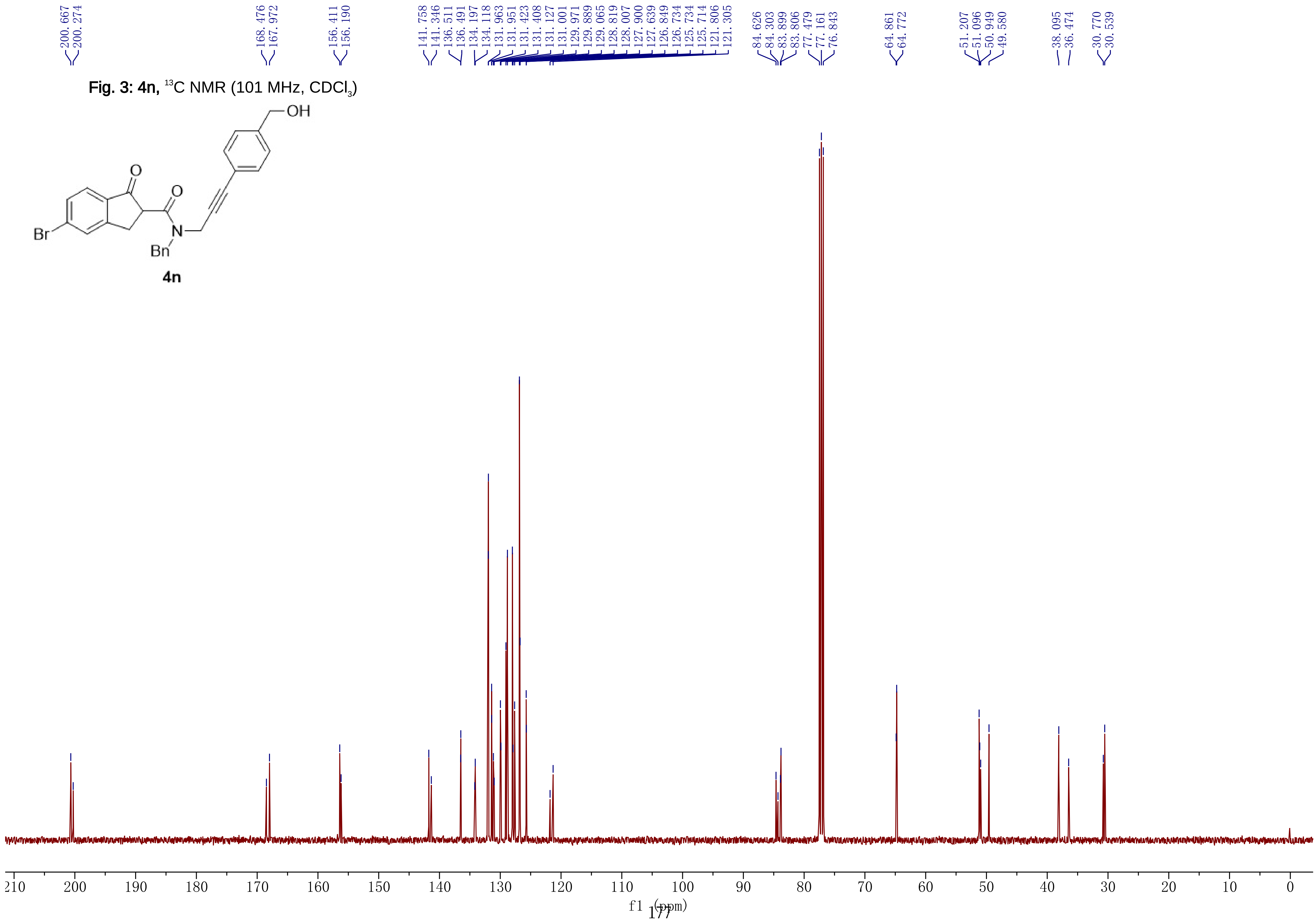

Fig. 3: 4n, ^{13}C NMR (101 MHz, $CDCl_3$)

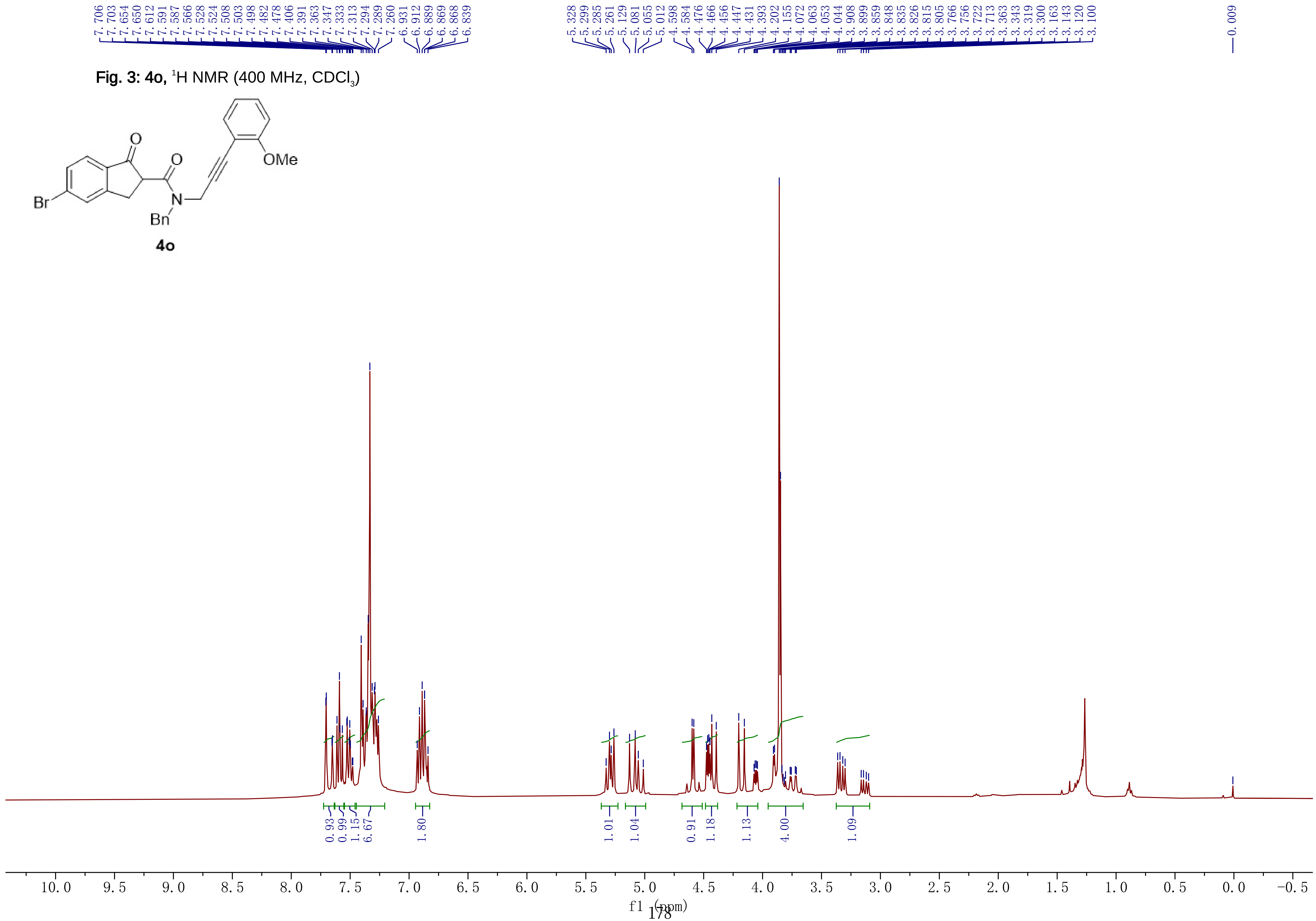

Fig. 3: 4o, ^{1}H NMR (400 MHz, $CDCl_3$)

Fig. 3: 4o, ^{13}C NMR (101 MHz, $CDCl_3$)

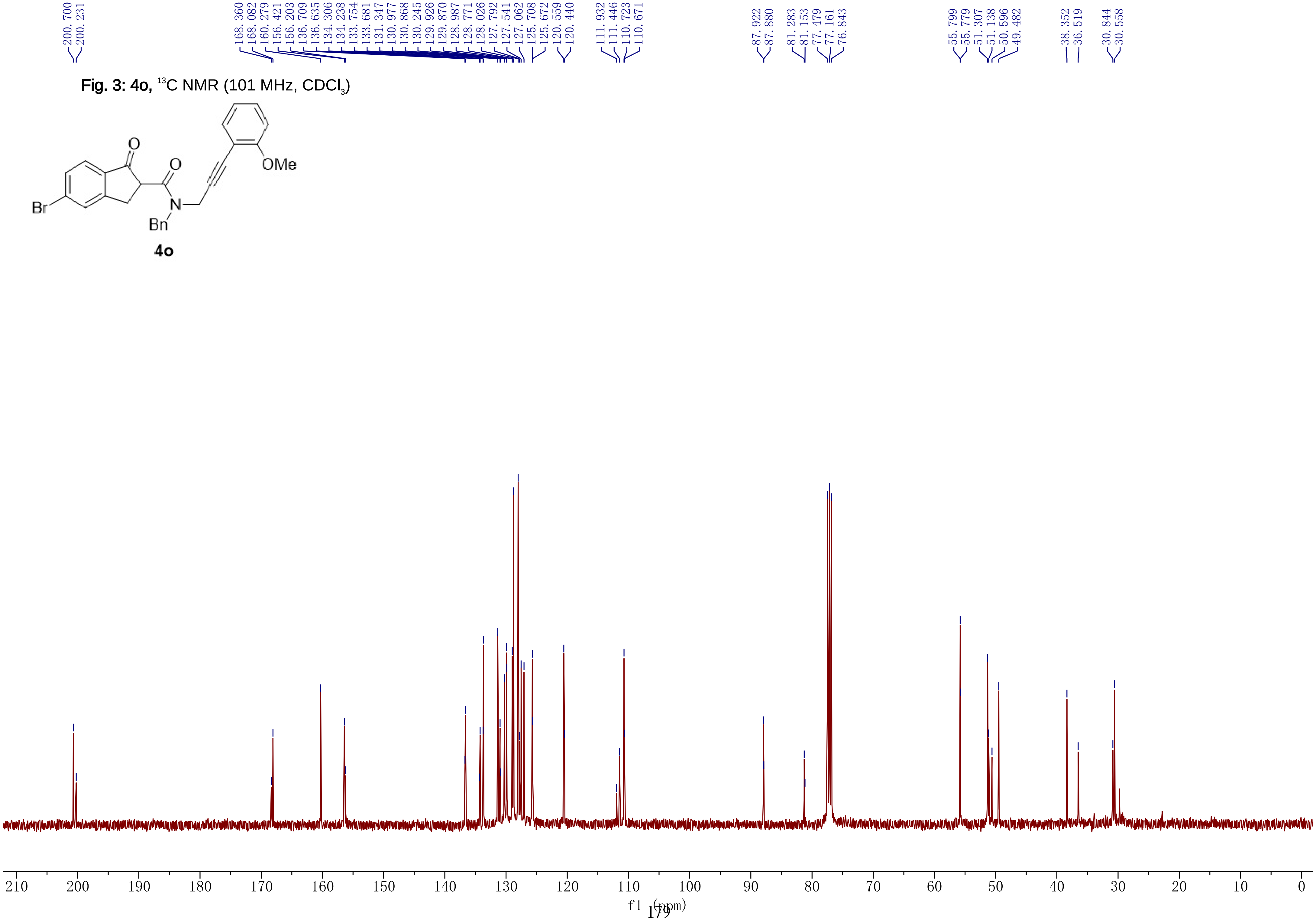

4o

Fig. 3: 4p, ^{1}H NMR (400 MHz, $CDCl_3$)

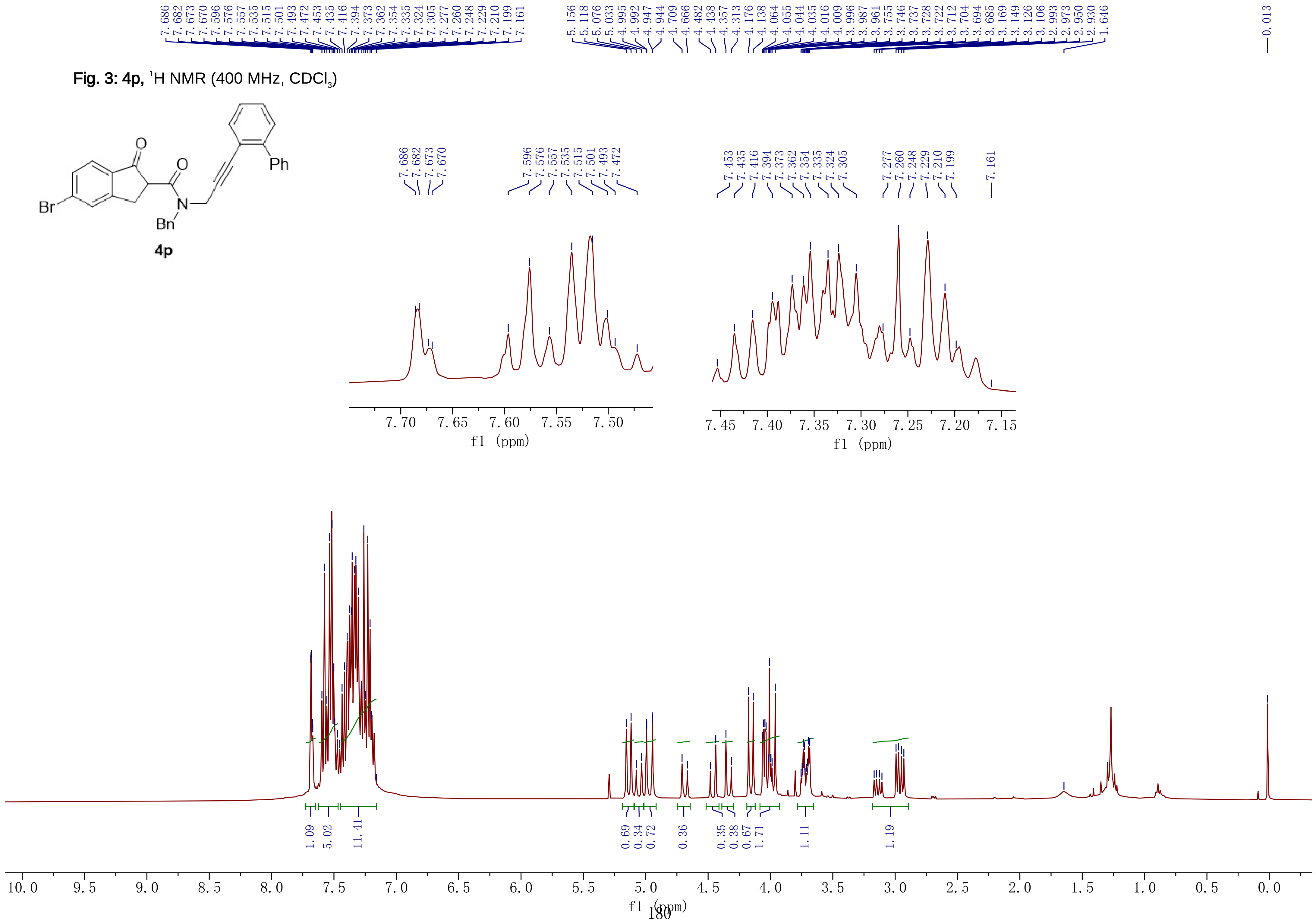

Fig. 3: 4p, ^{13}C NMR (101 MHz, $CDCl_3$)

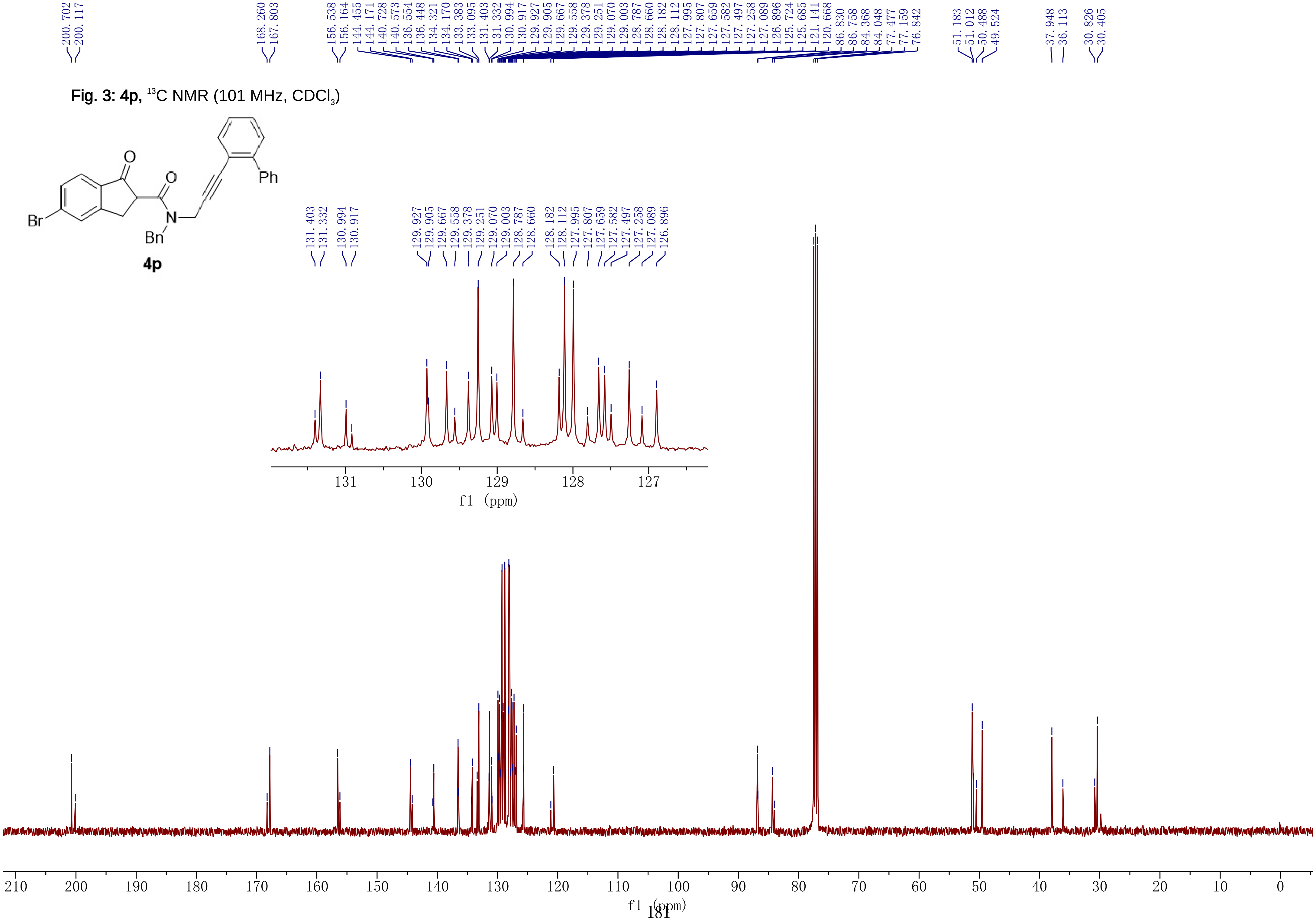

4p

Fig. 3: 4q, ^1H NMR (400 MHz, $CDCl_3$)

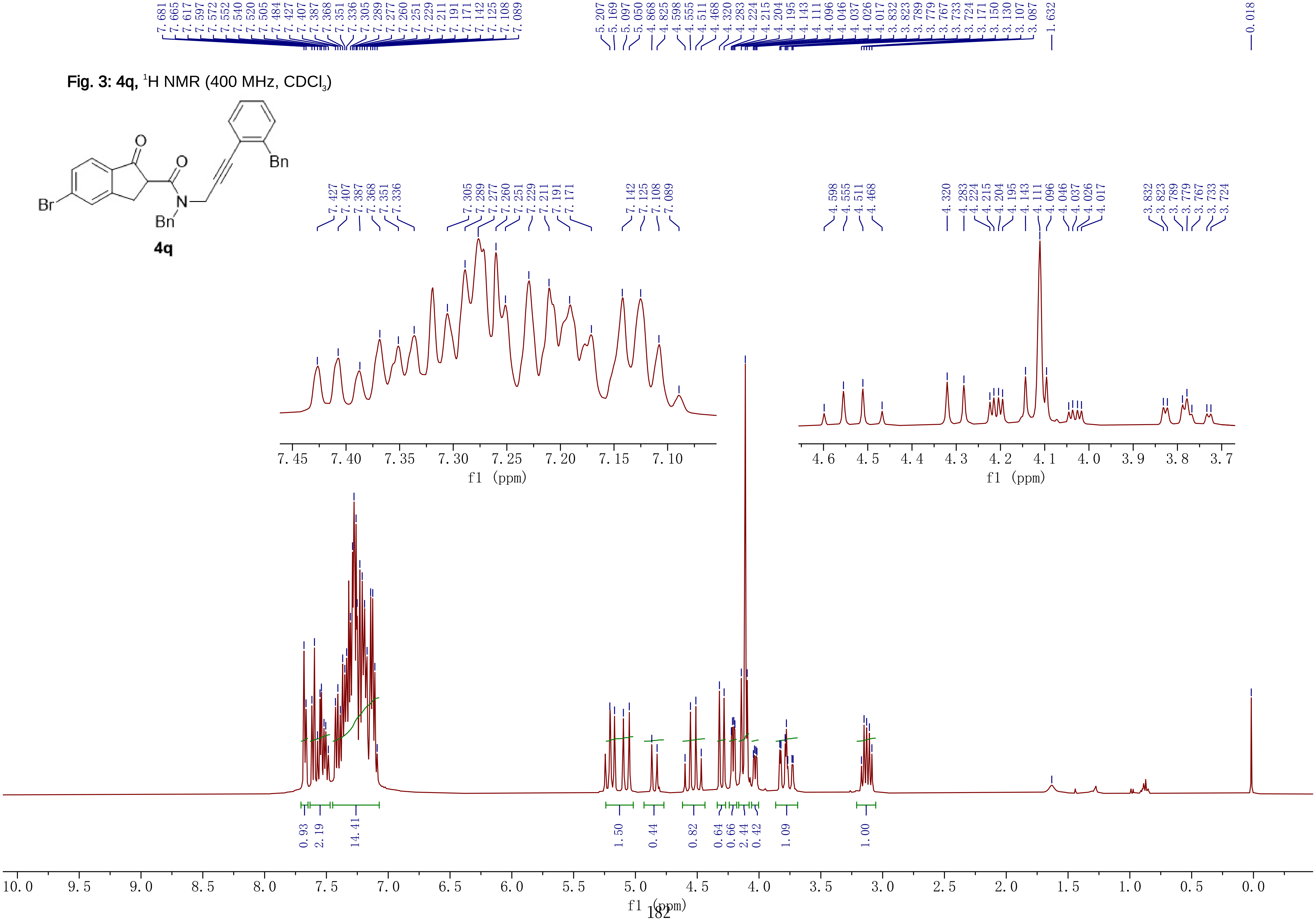

Fig. 3: 4q, ^{13}C NMR (101 MHz, $CDCl_3$)

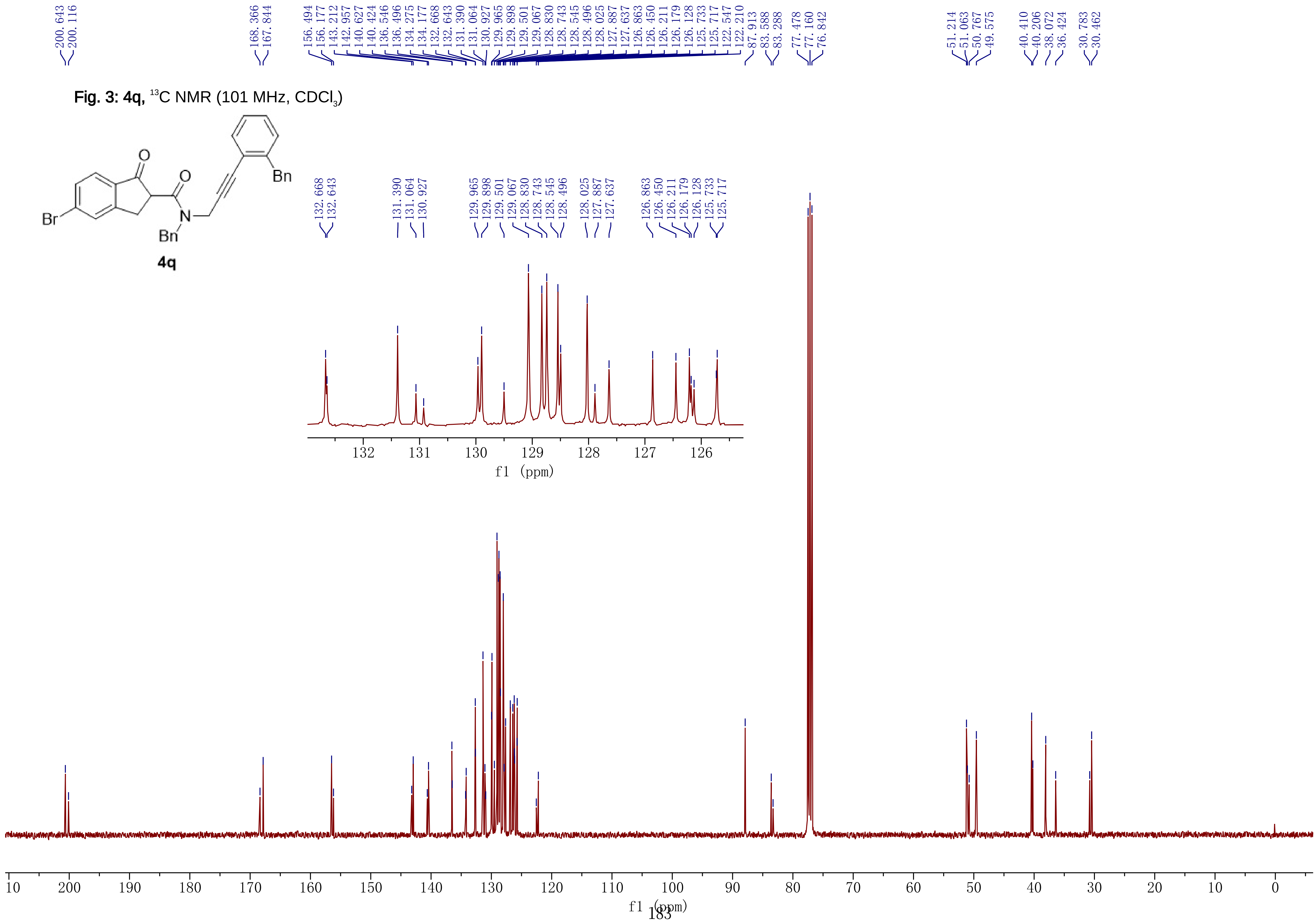

Fig. 3: 4r, ^{1}H NMR (400 MHz, $CDCl_3$)

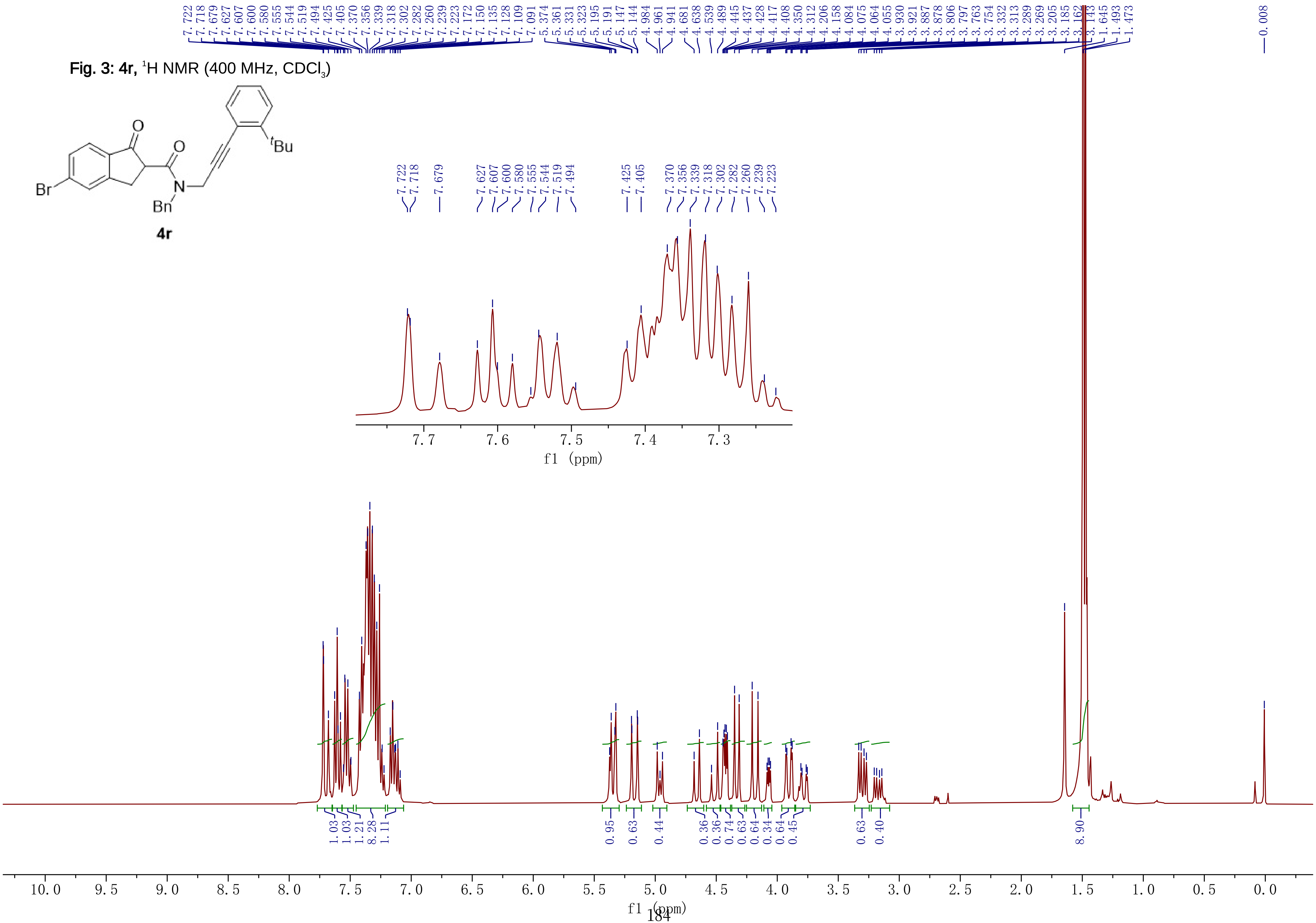

Fig. 3: 4r, ^{13}C NMR (101 MHz, $CDCl_3$)

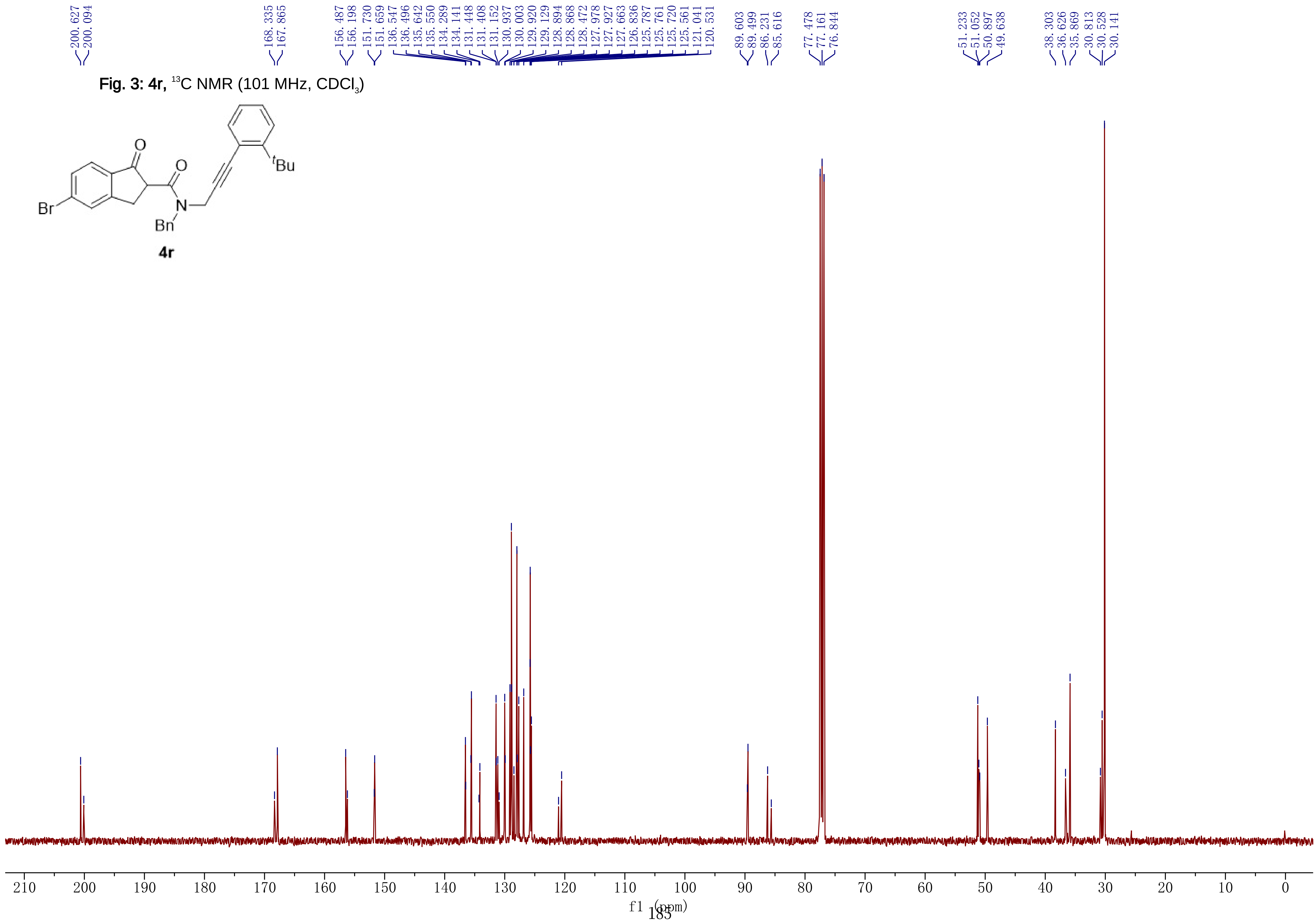

Fig. 3: 4s, ^{1}H NMR (400 MHz, $CDCl_3$)

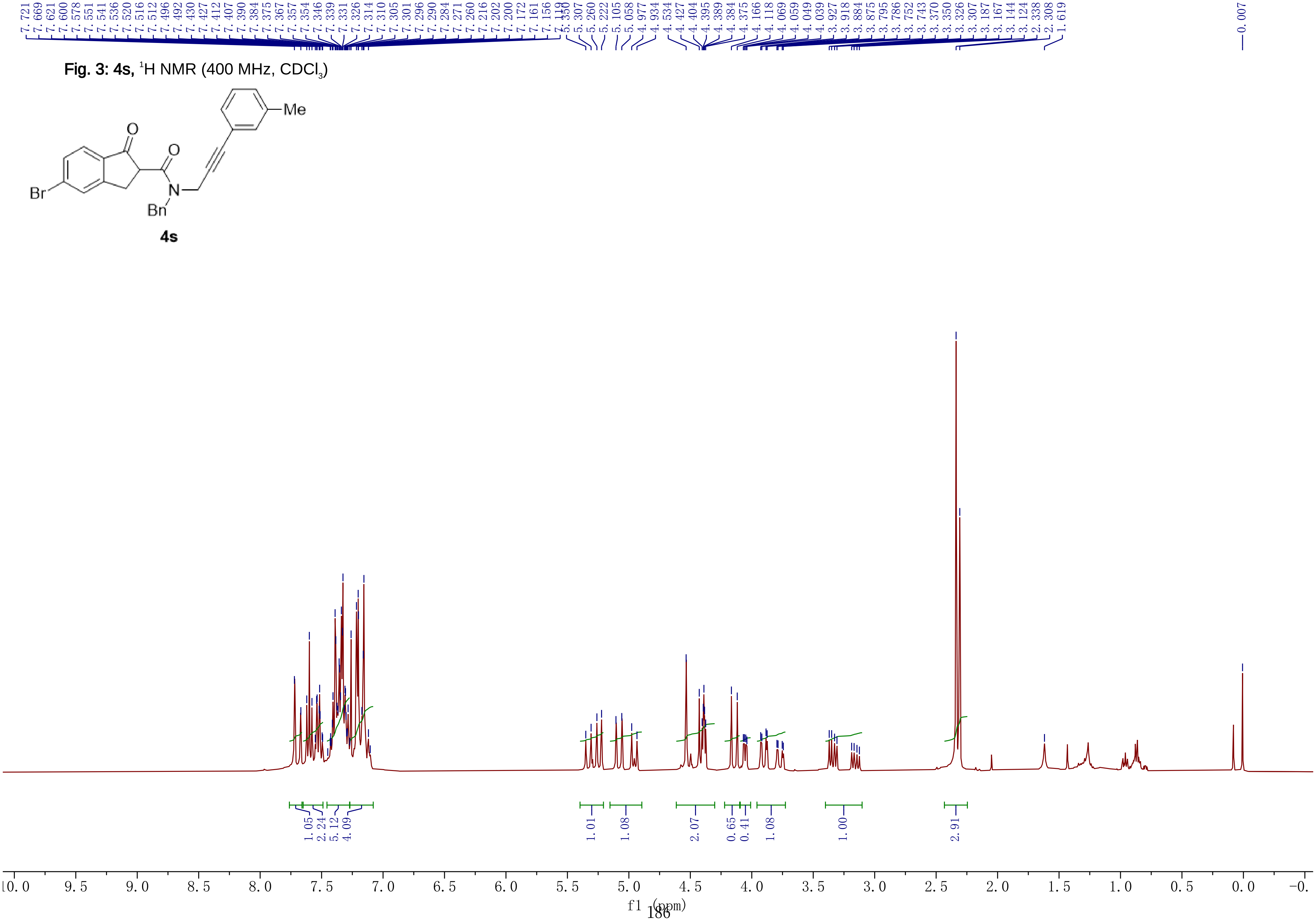

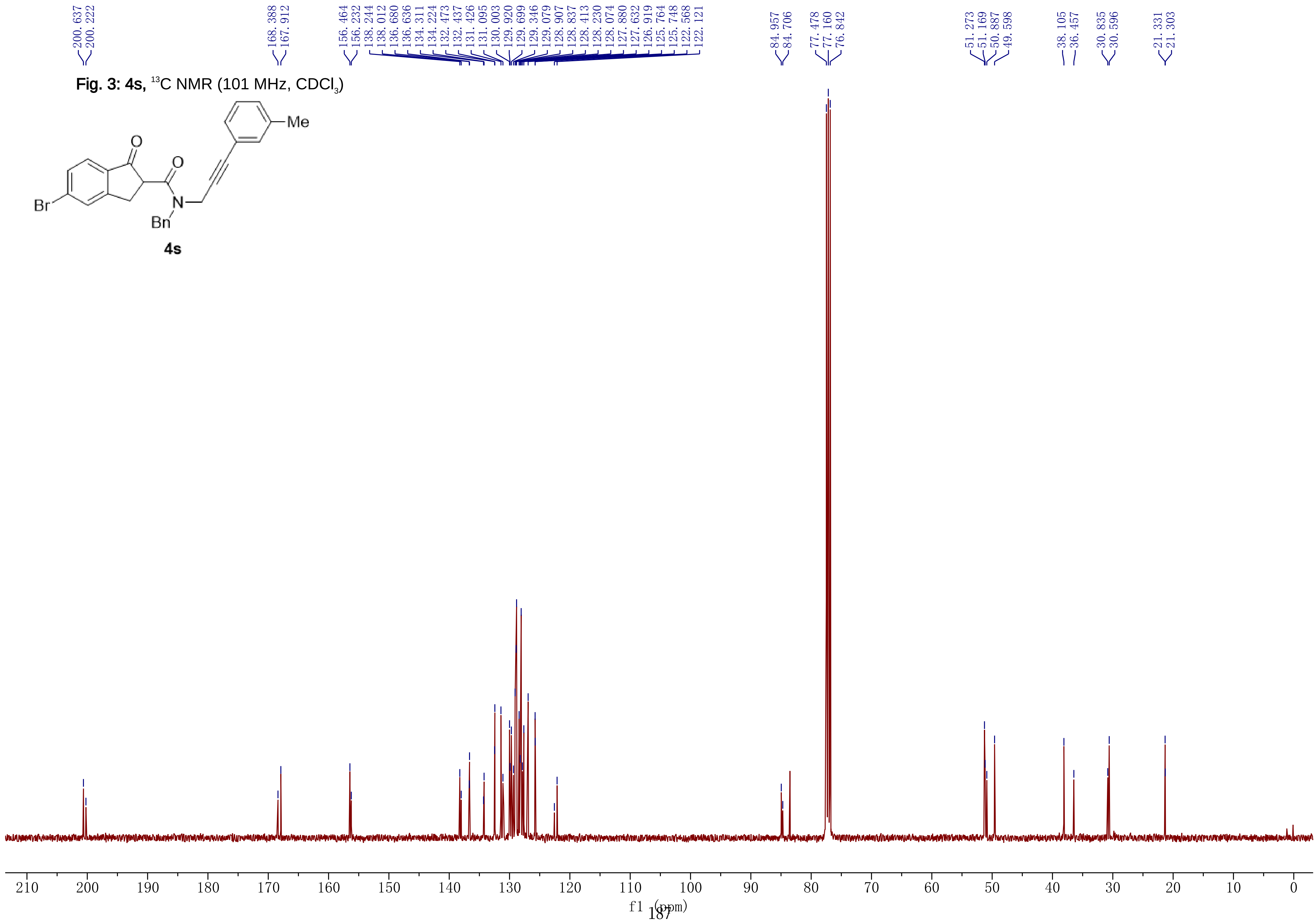

Fig. 3: 4s, ^{13}C NMR (101 MHz, $CDCl_3$)

4s

Fig. 3: 4t, ^{1}H NMR (400 MHz, $CDCl_3$)

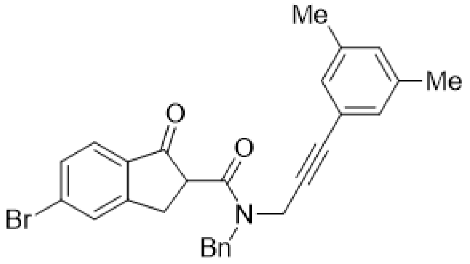

4t

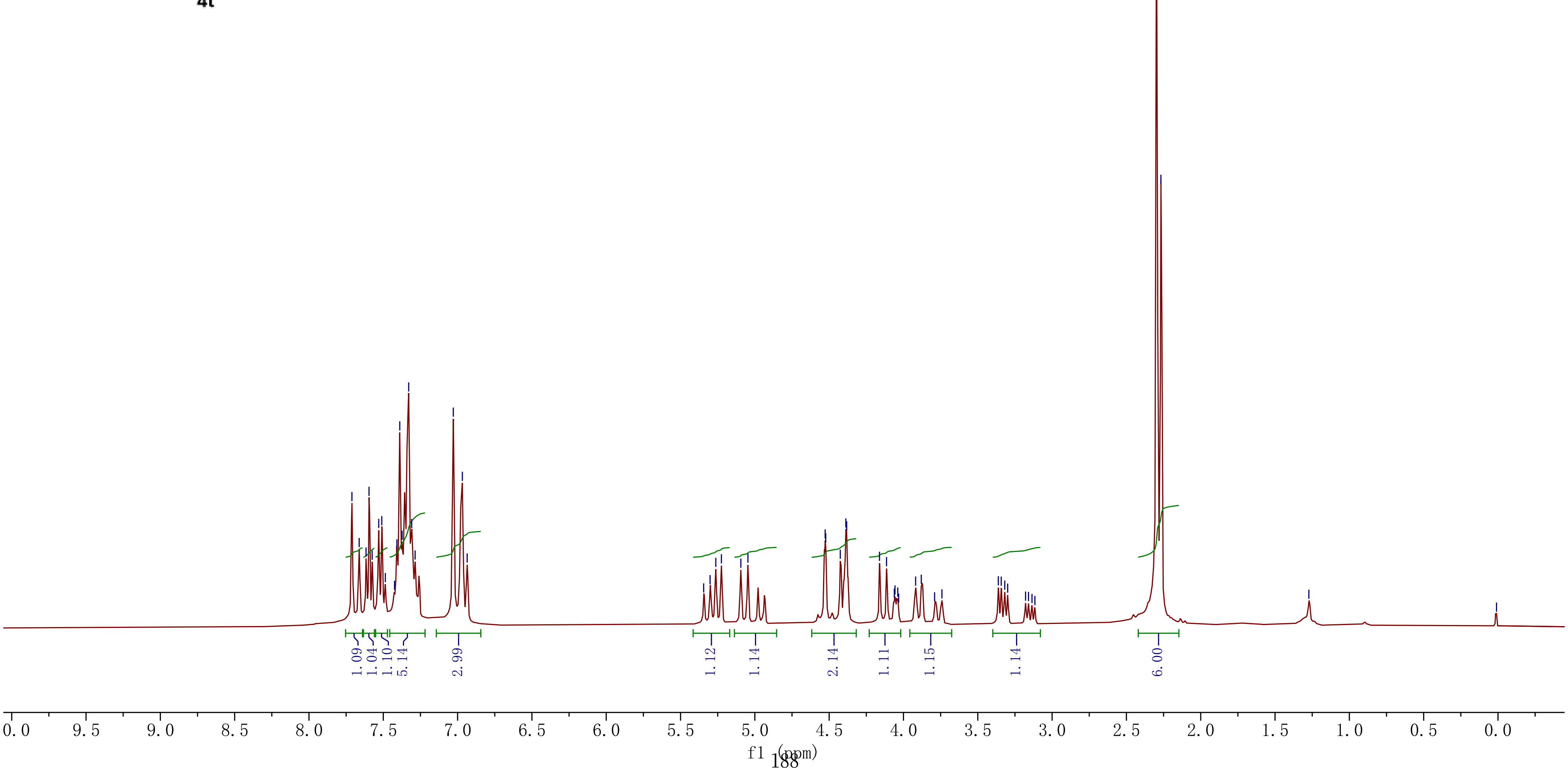

Fig. 3: 4t, ^{13}C NMR (101 MHz, $CDCl_3$)

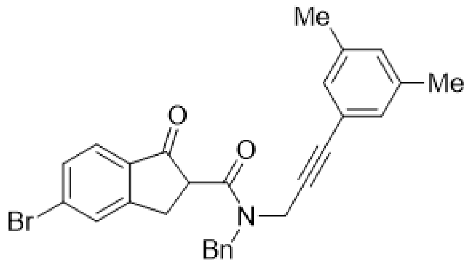

4t

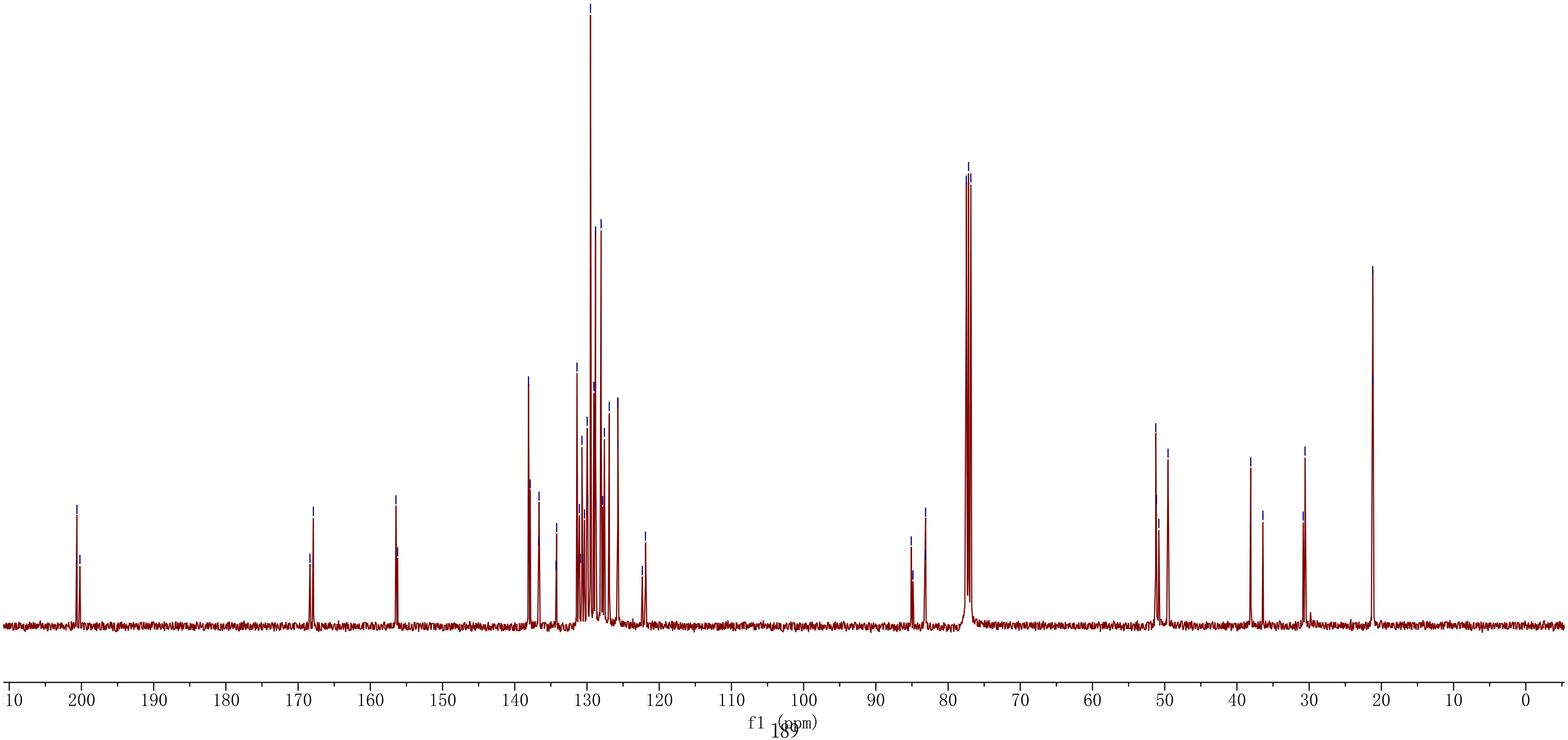

Fig. 3: 4u, ^{1}H NMR (400 MHz, $CDCl_3$)

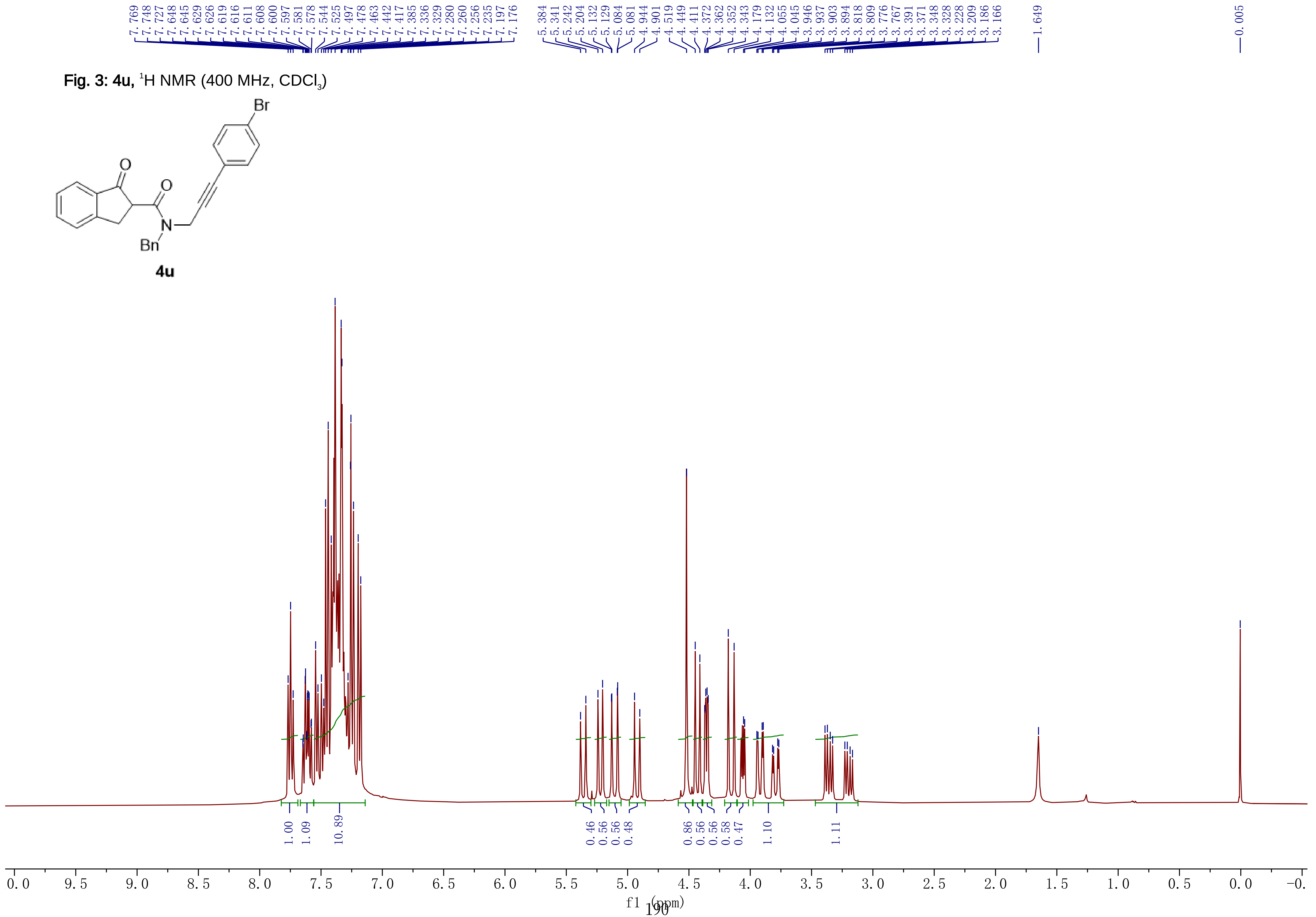

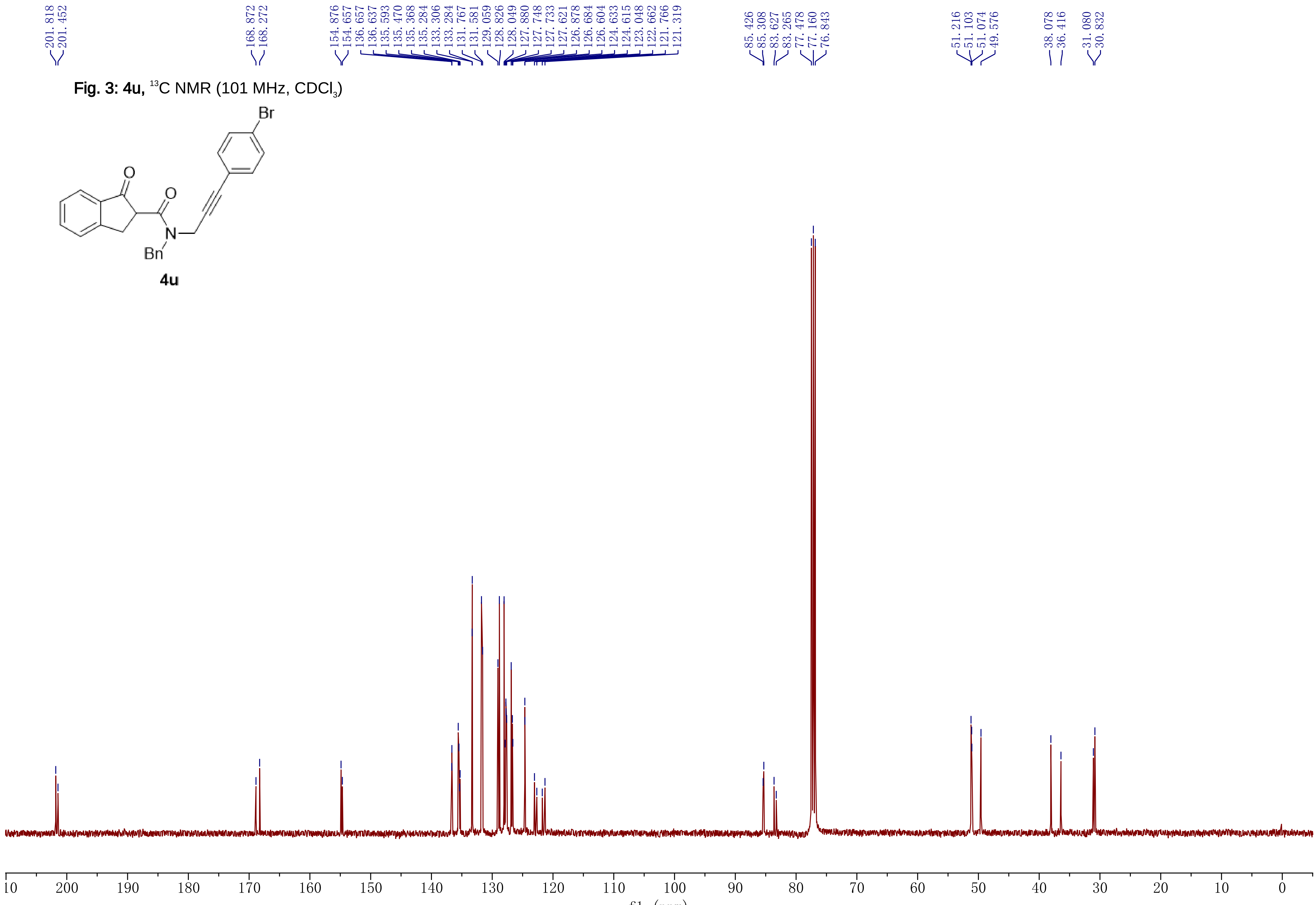

Fig. 3: 4u, ^{13}C NMR (101 MHz, $CDCl_3$)

4u

Fig. 3: 4v, ^{1}H NMR (400 MHz, $CDCl_3$)

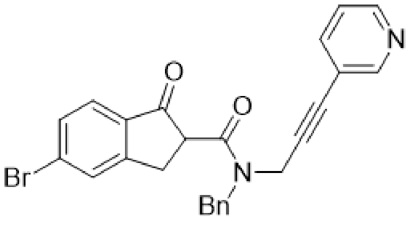

4v

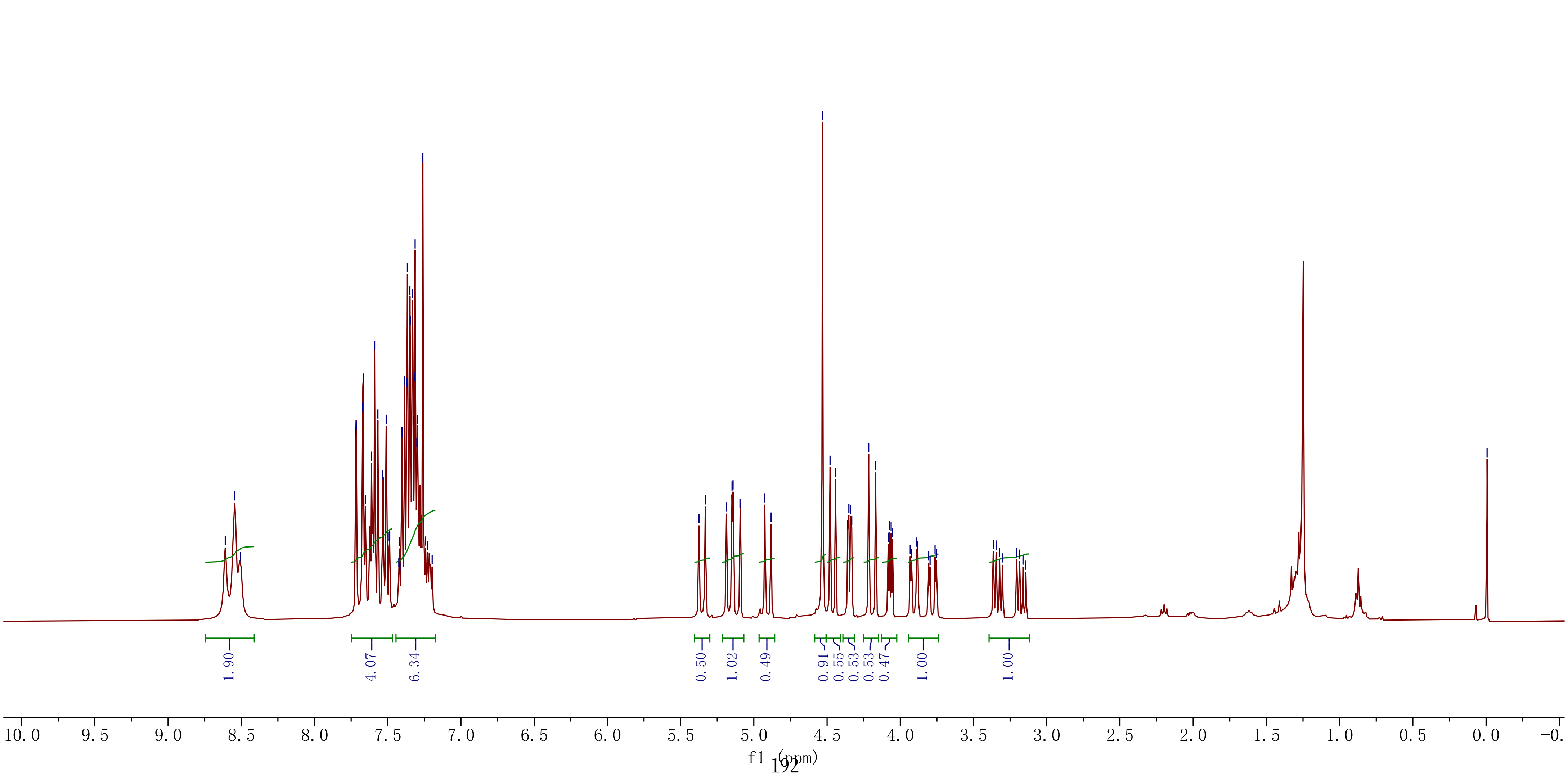

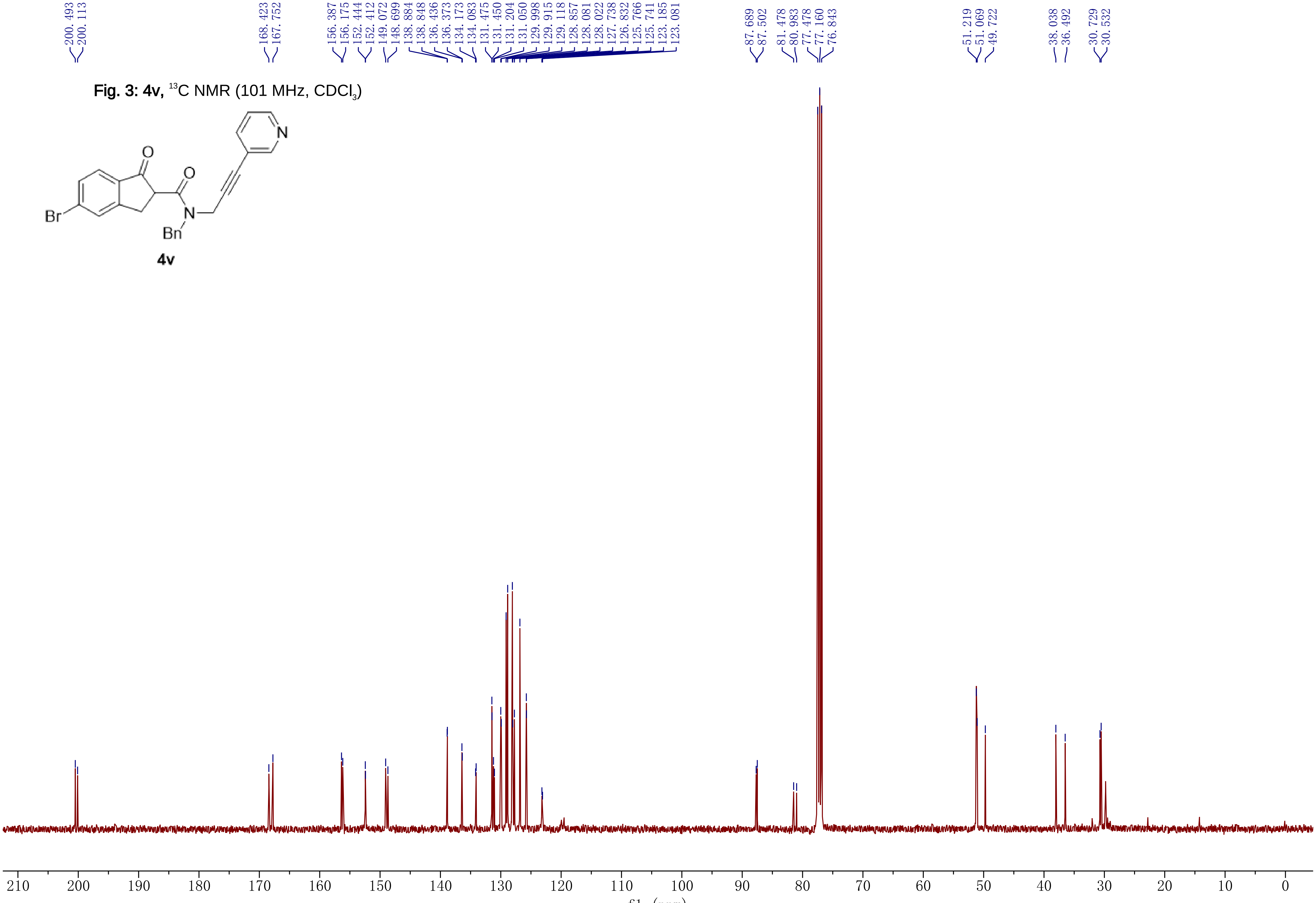

Fig. 3: 4v, ^{13}C NMR (101 MHz, $CDCl_3$)

Fig. 3: 4w, ^{1}H NMR (400 MHz, $CDCl_3$)

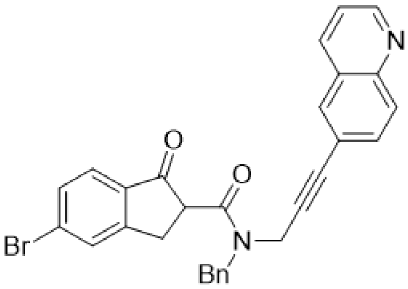

4w

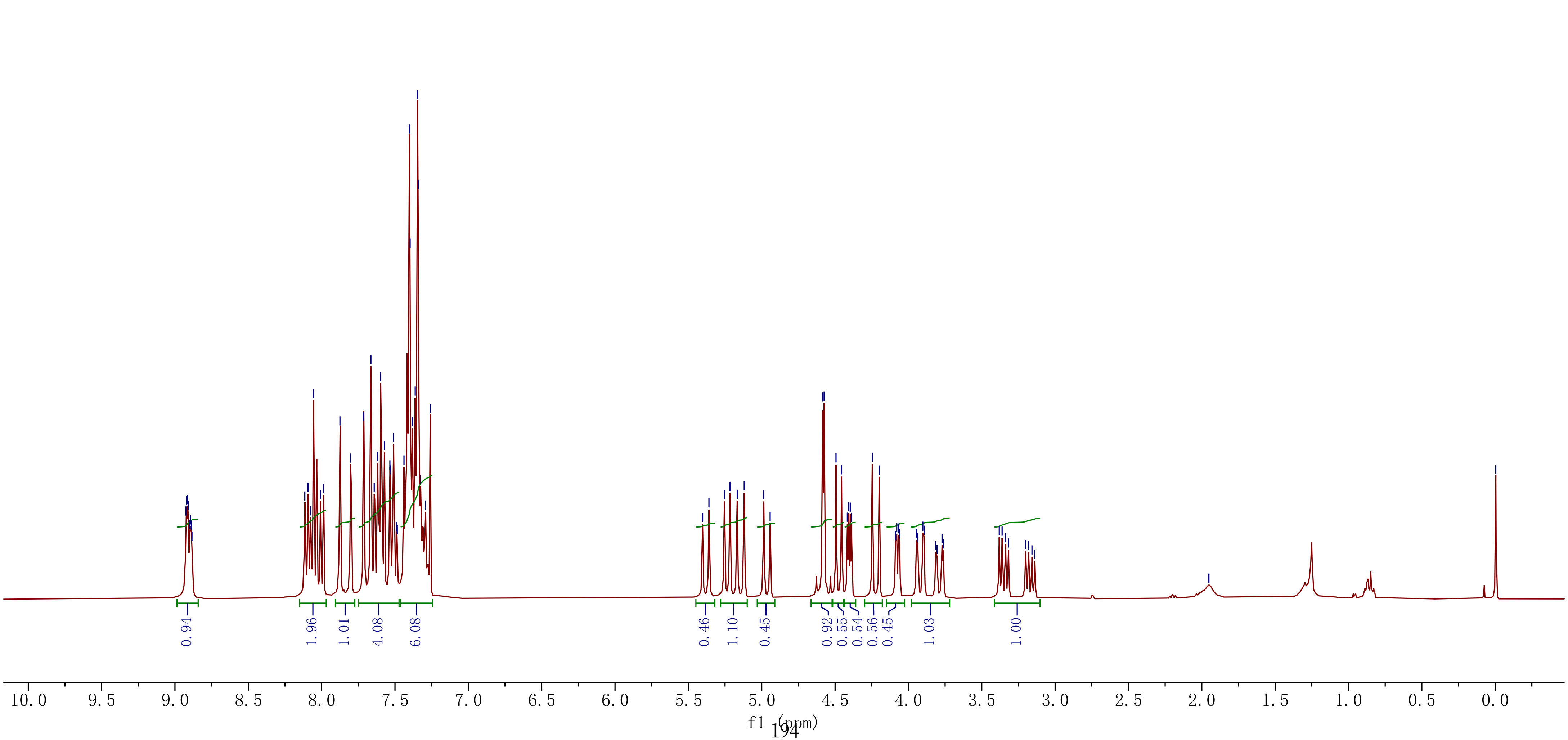

Fig. 3: 4w, ^{13}C NMR (101 MHz, $CDCl_3$)

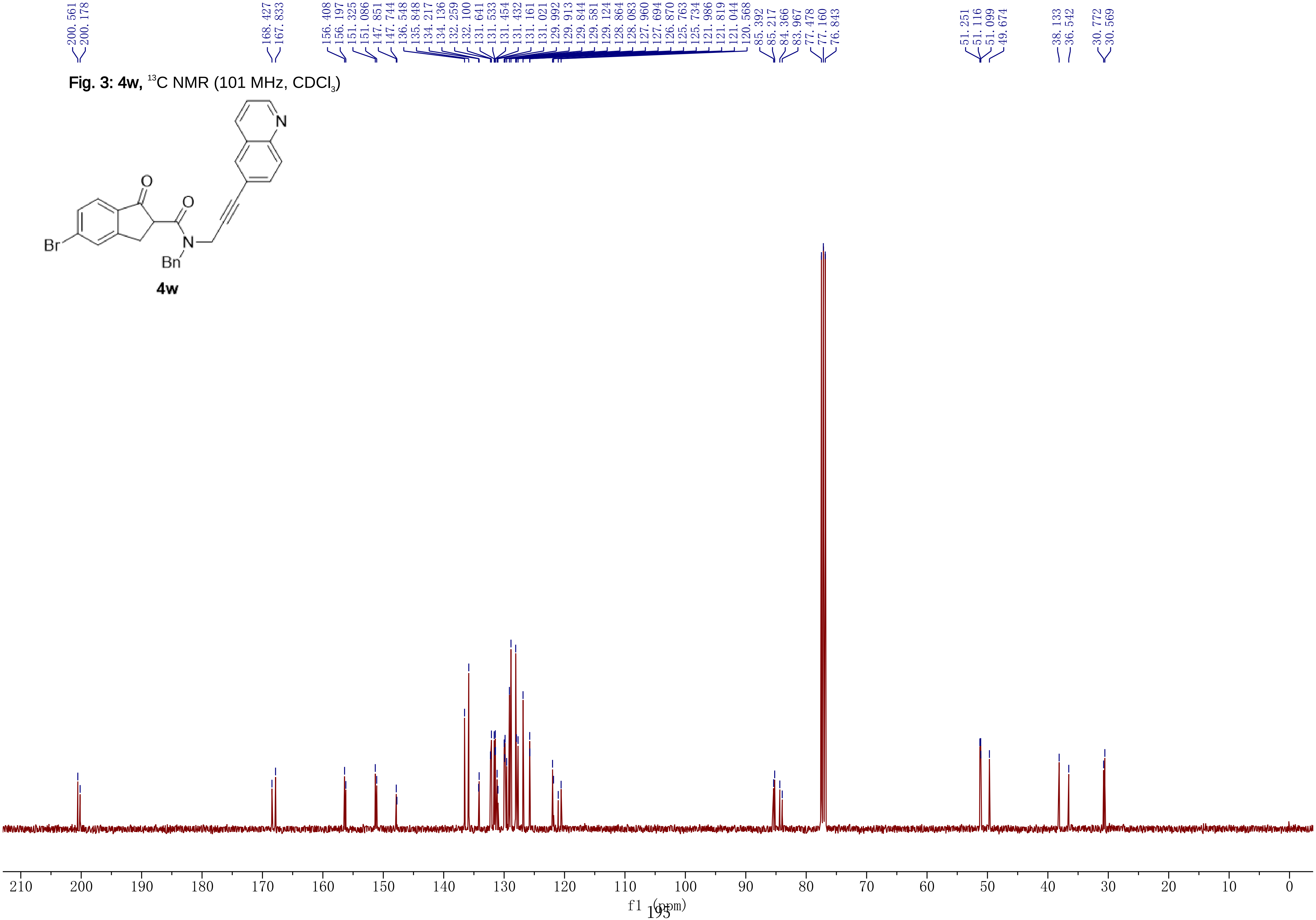

Fig. 3: 4x, ^{1}H NMR (400 MHz, $CDCl_3$)

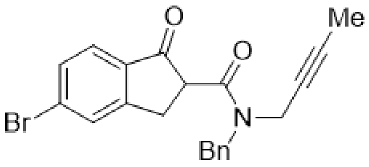

4x

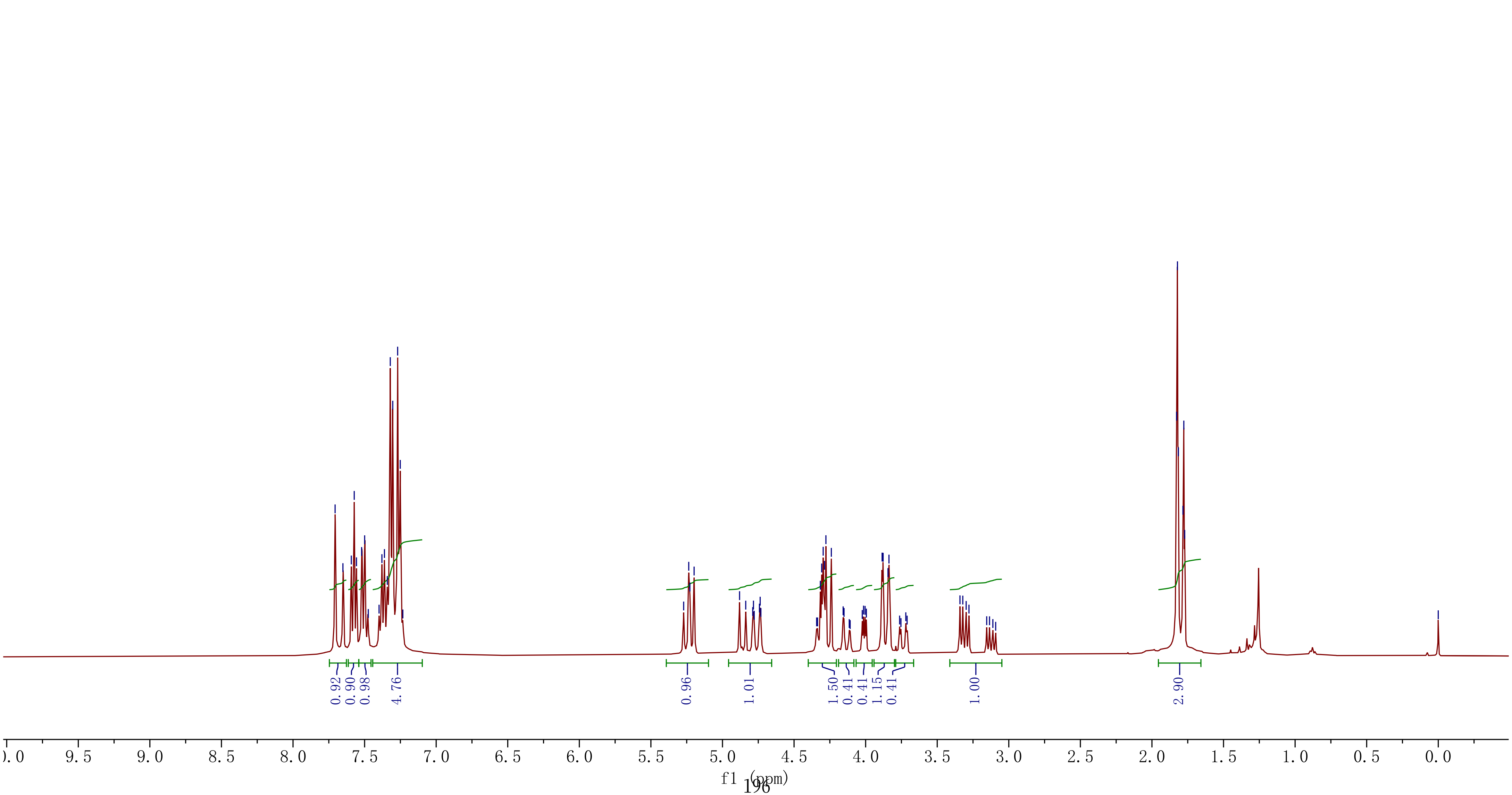

Fig. 3: 4x, ^{13}C NMR (101 MHz, $CDCl_3$)

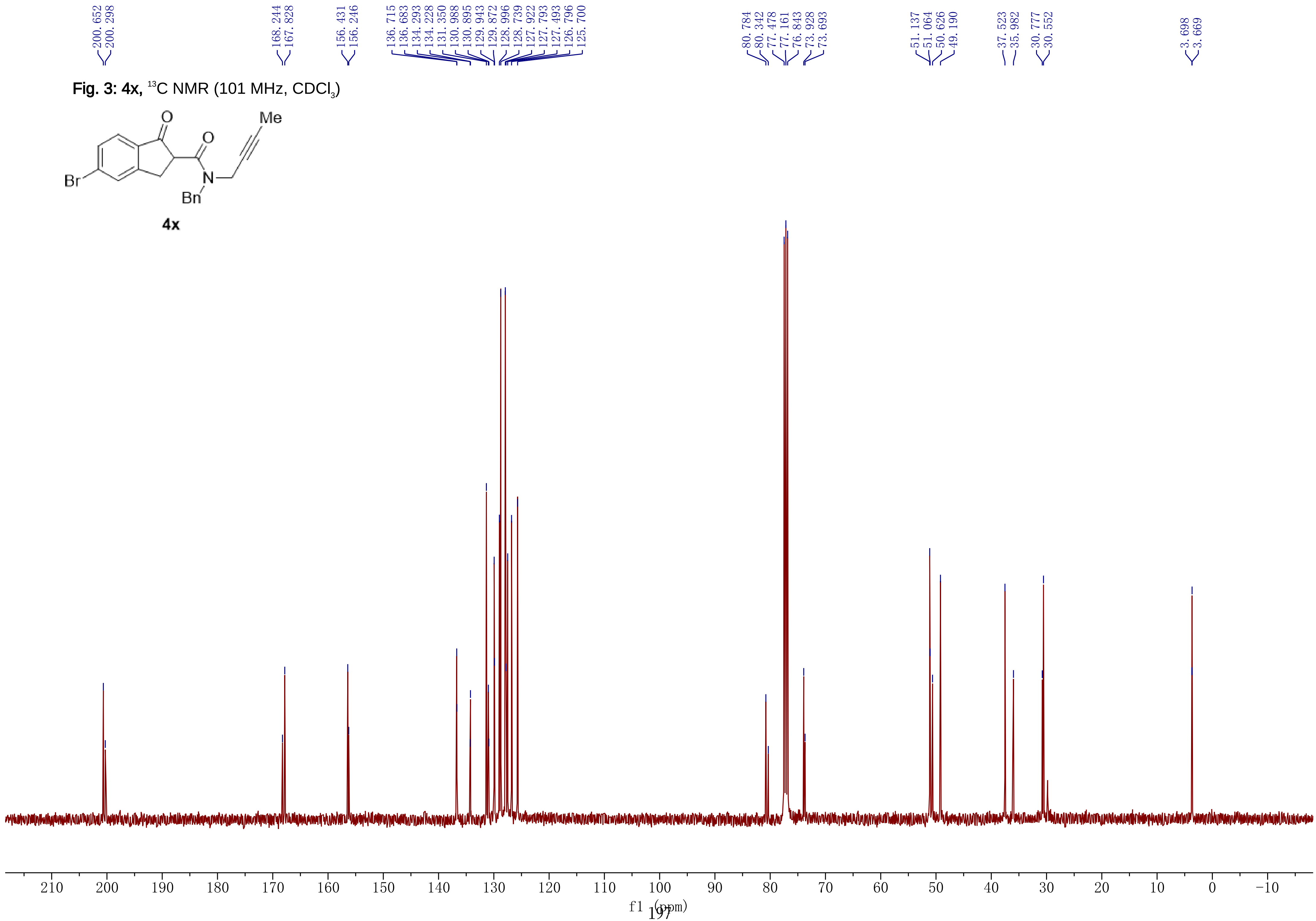

Fig. 3: 4y, ^{1}H NMR (400 MHz, $CDCl_3$)

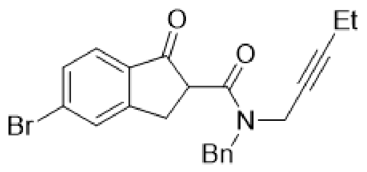

4y

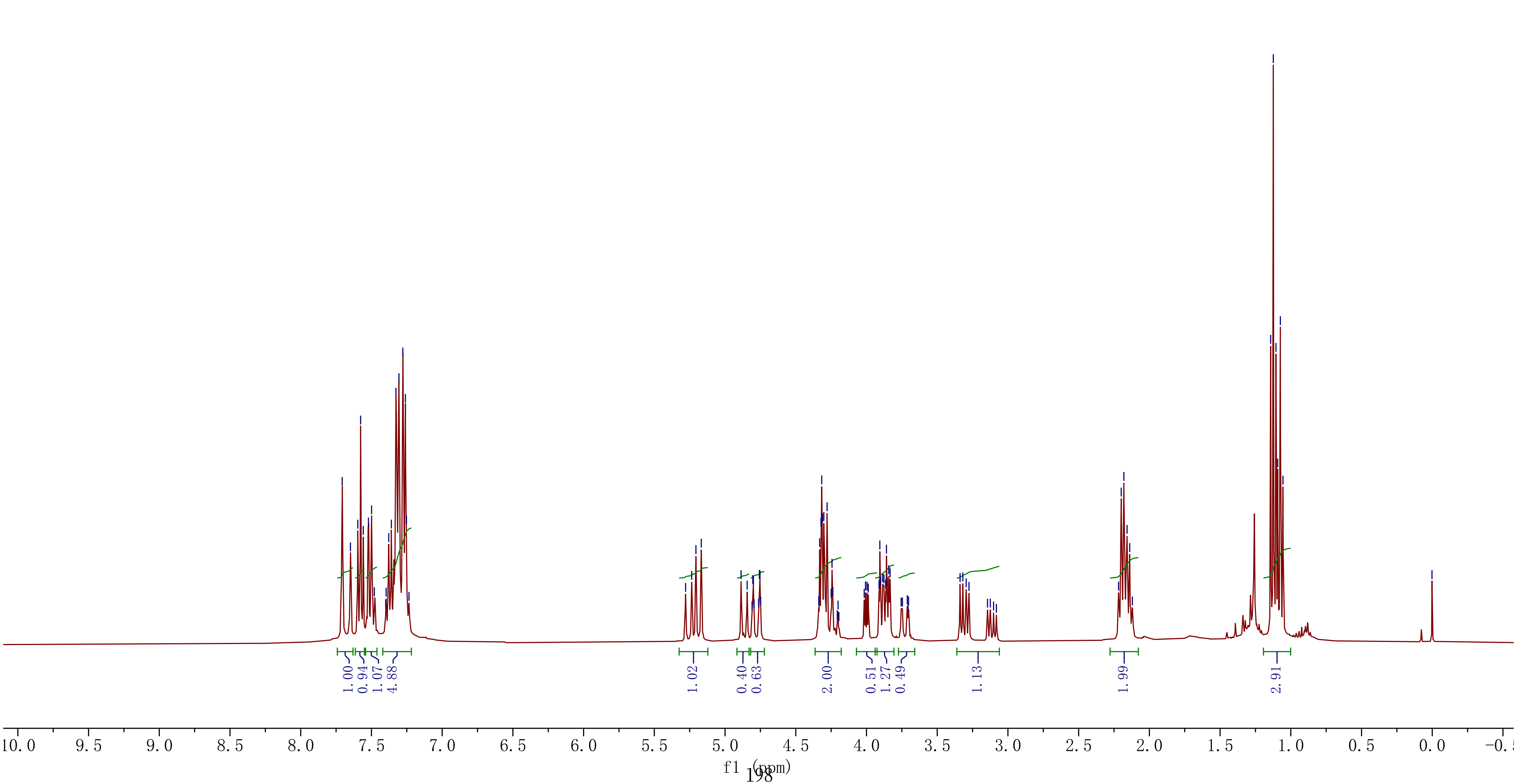

Fig. 3: 4y, ^{13}C NMR (101 MHz, $CDCl_3$)

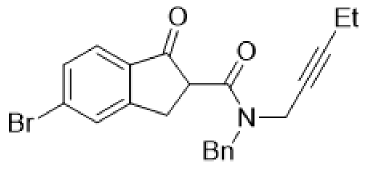

4y

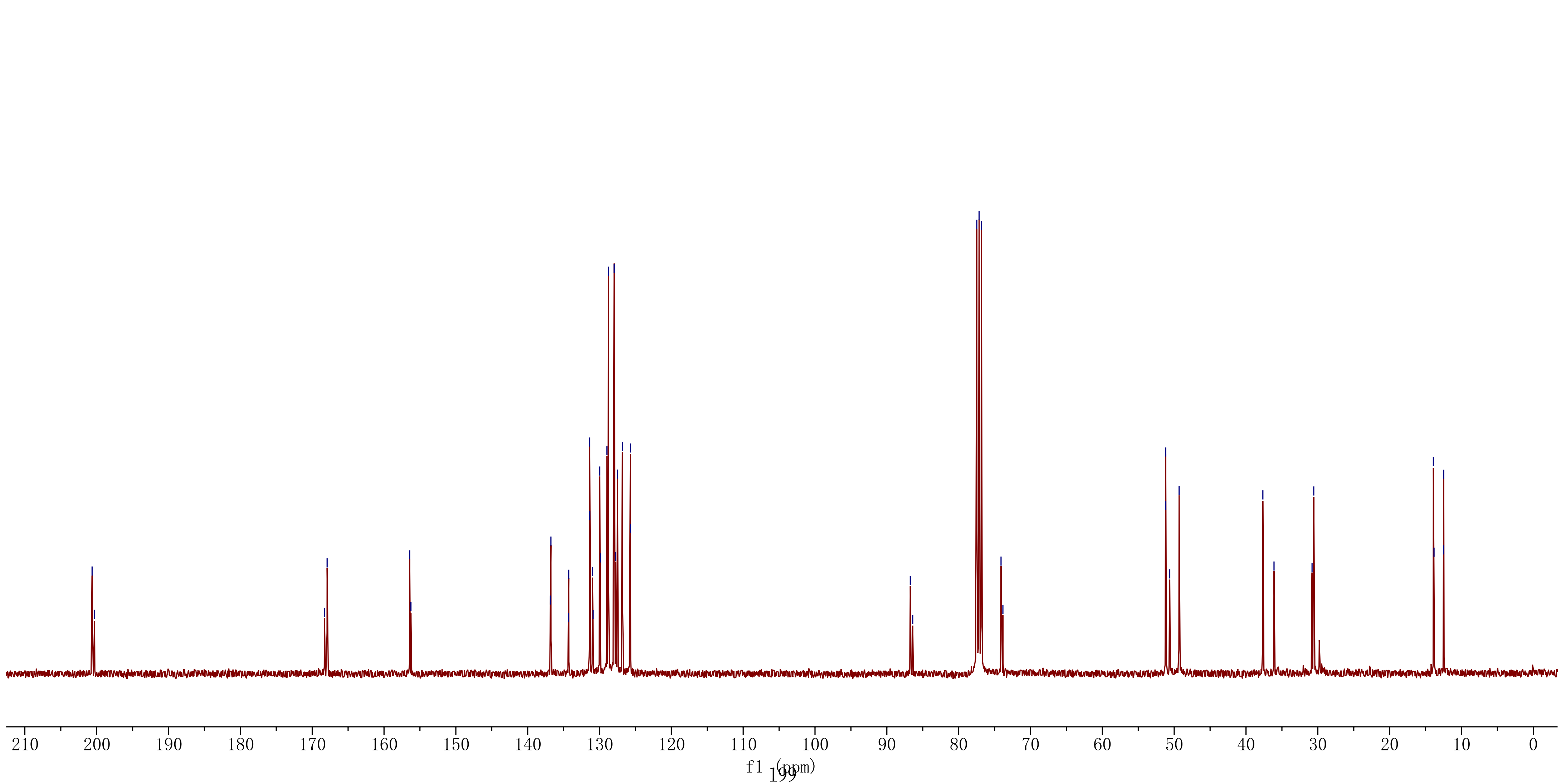

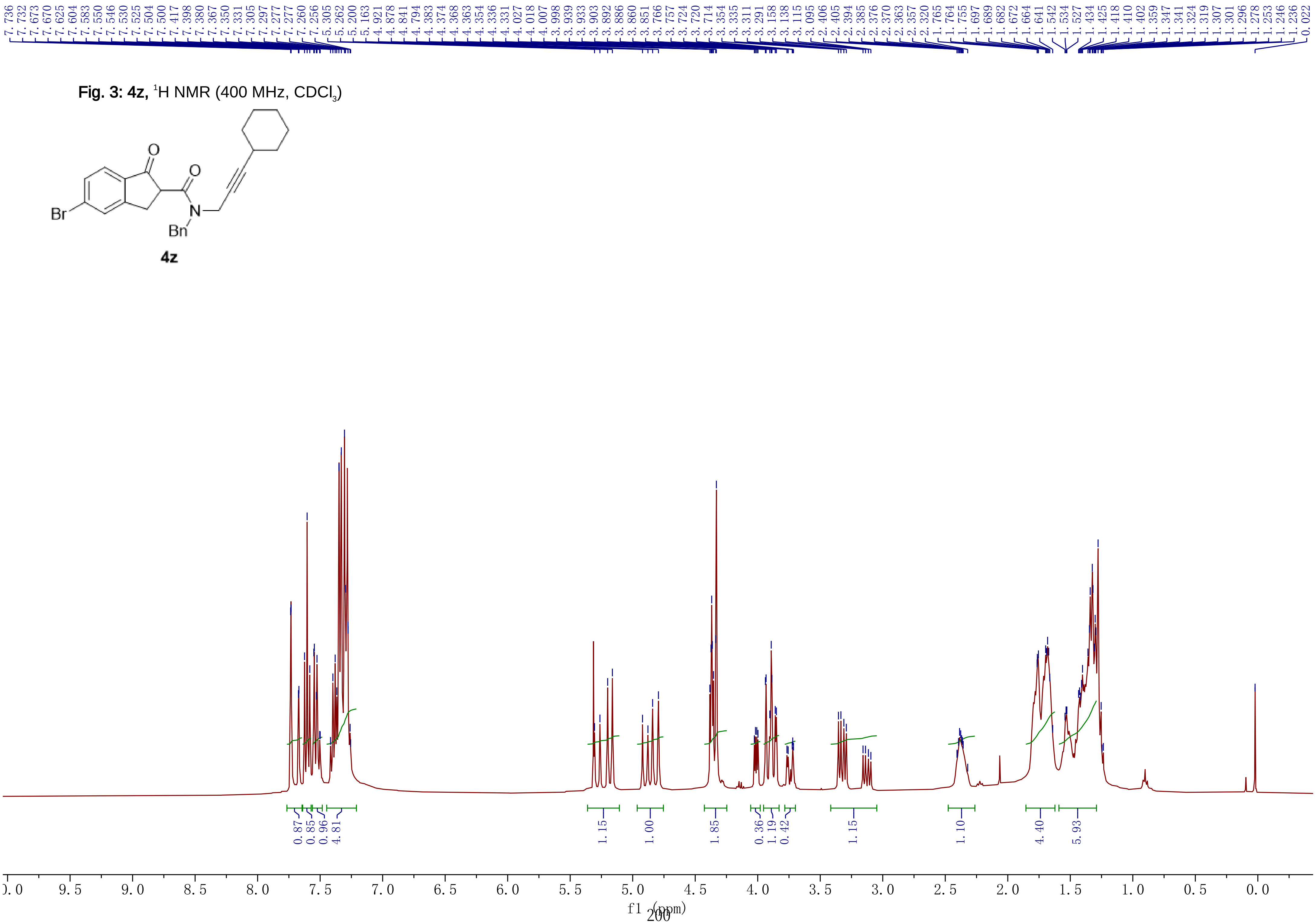

Fig. 3: 4z, ^{1}H NMR (400 MHz, $CDCl_3$)

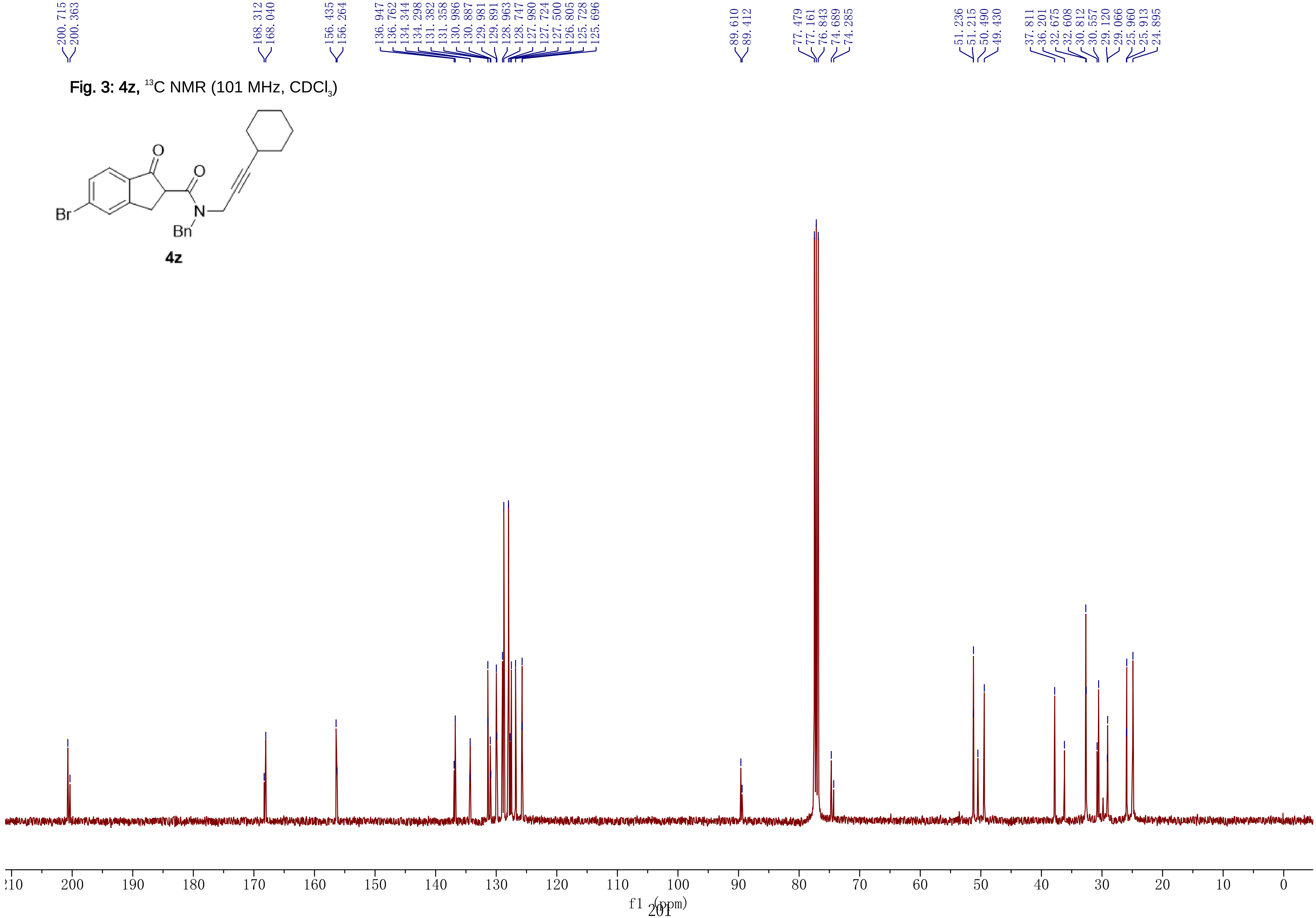

Fig. 3: 4z, ^{13}C NMR (101 MHz, $CDCl_3$)

4z

Fig. 2: 3a, ^{1}H NMR (400 MHz, $CDCl_3$)

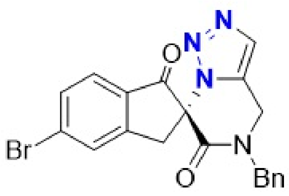

3a

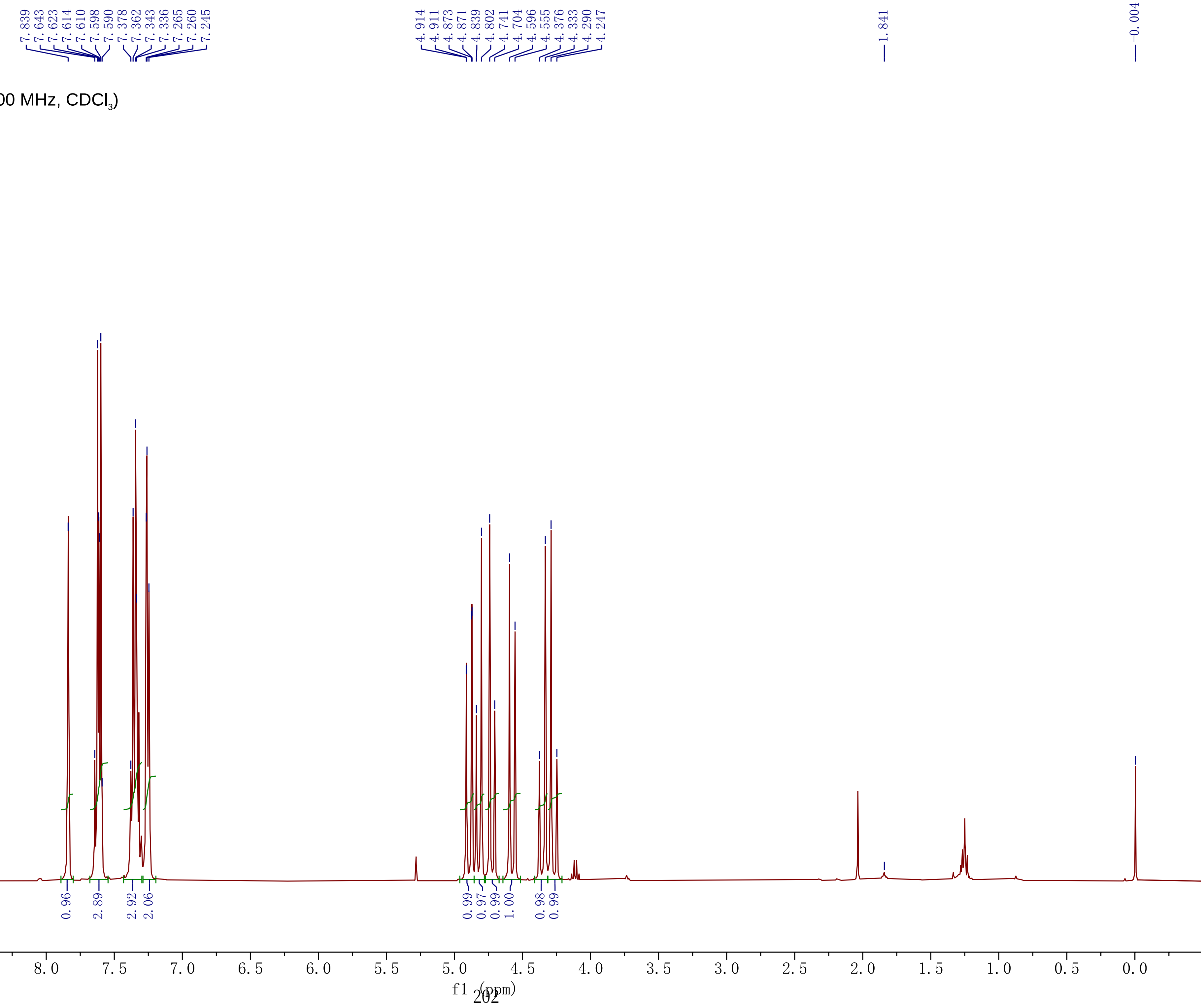

Fig. 2: 3a, ^{13}C NMR (101 MHz, $CDCl_3$)

3a

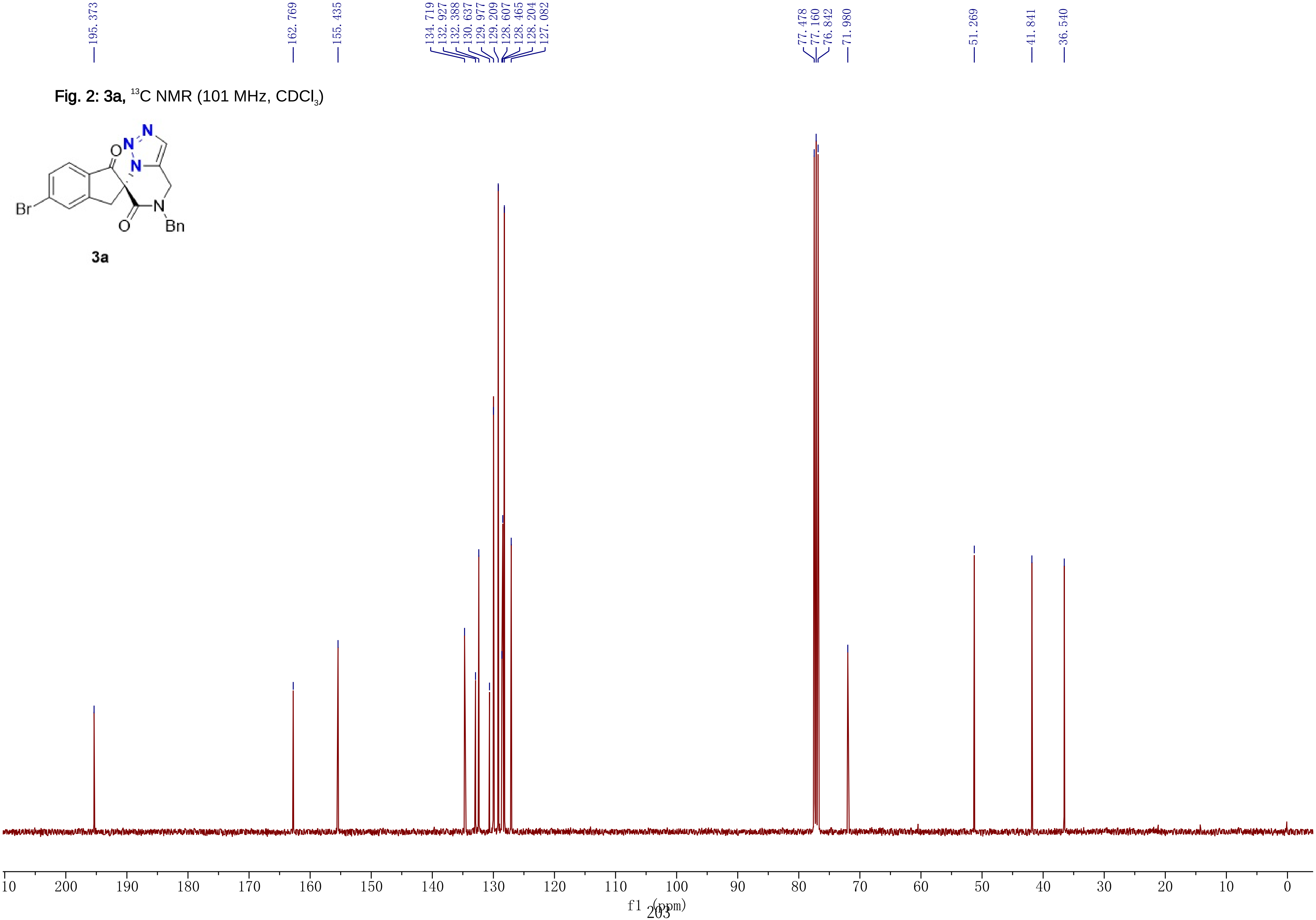

Fig. 2: 3b, ^{1}H NMR (400 MHz, $CDCl_3$)

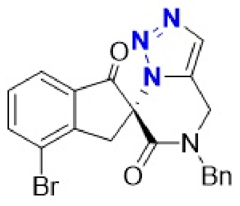

3b

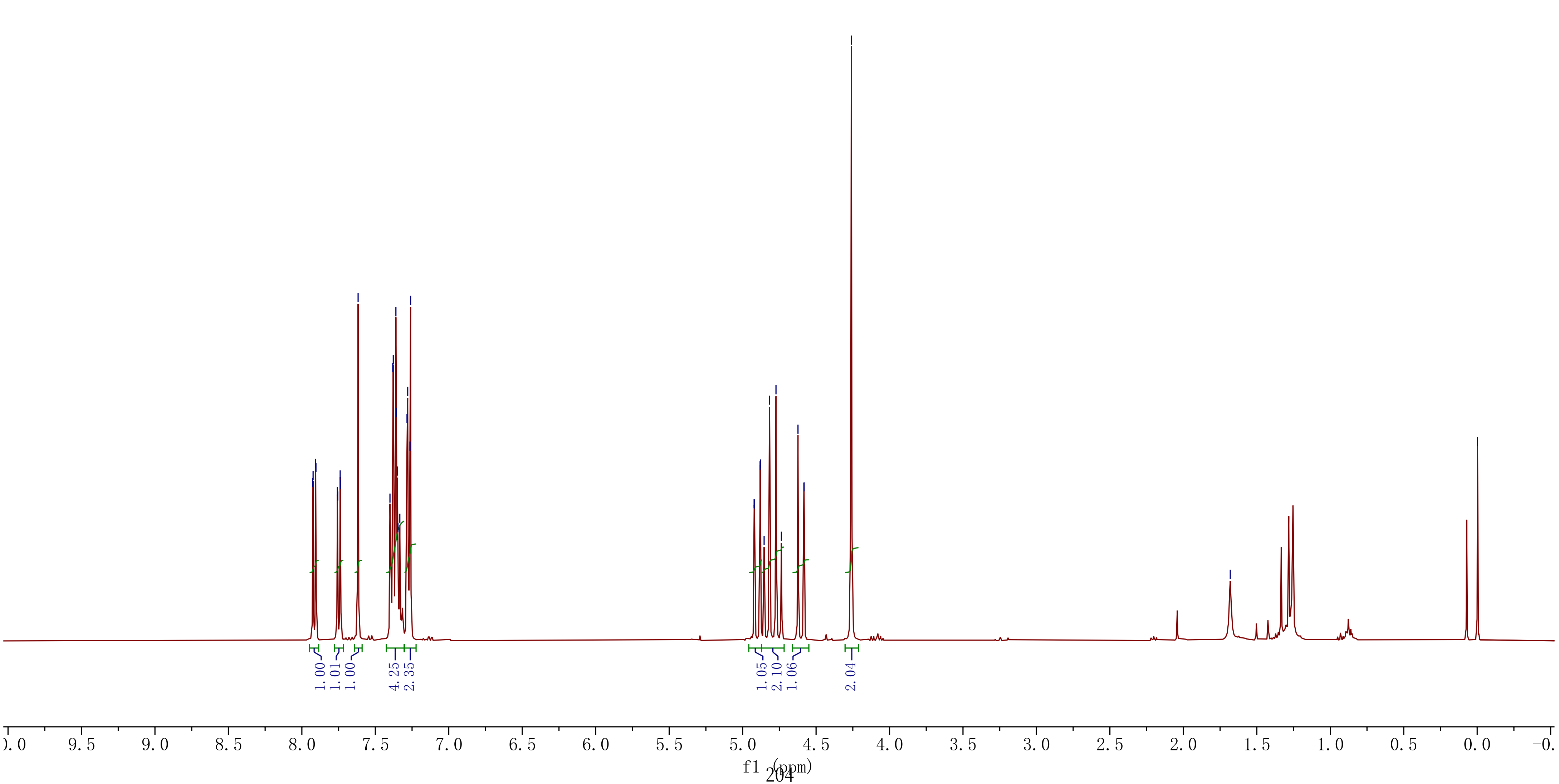

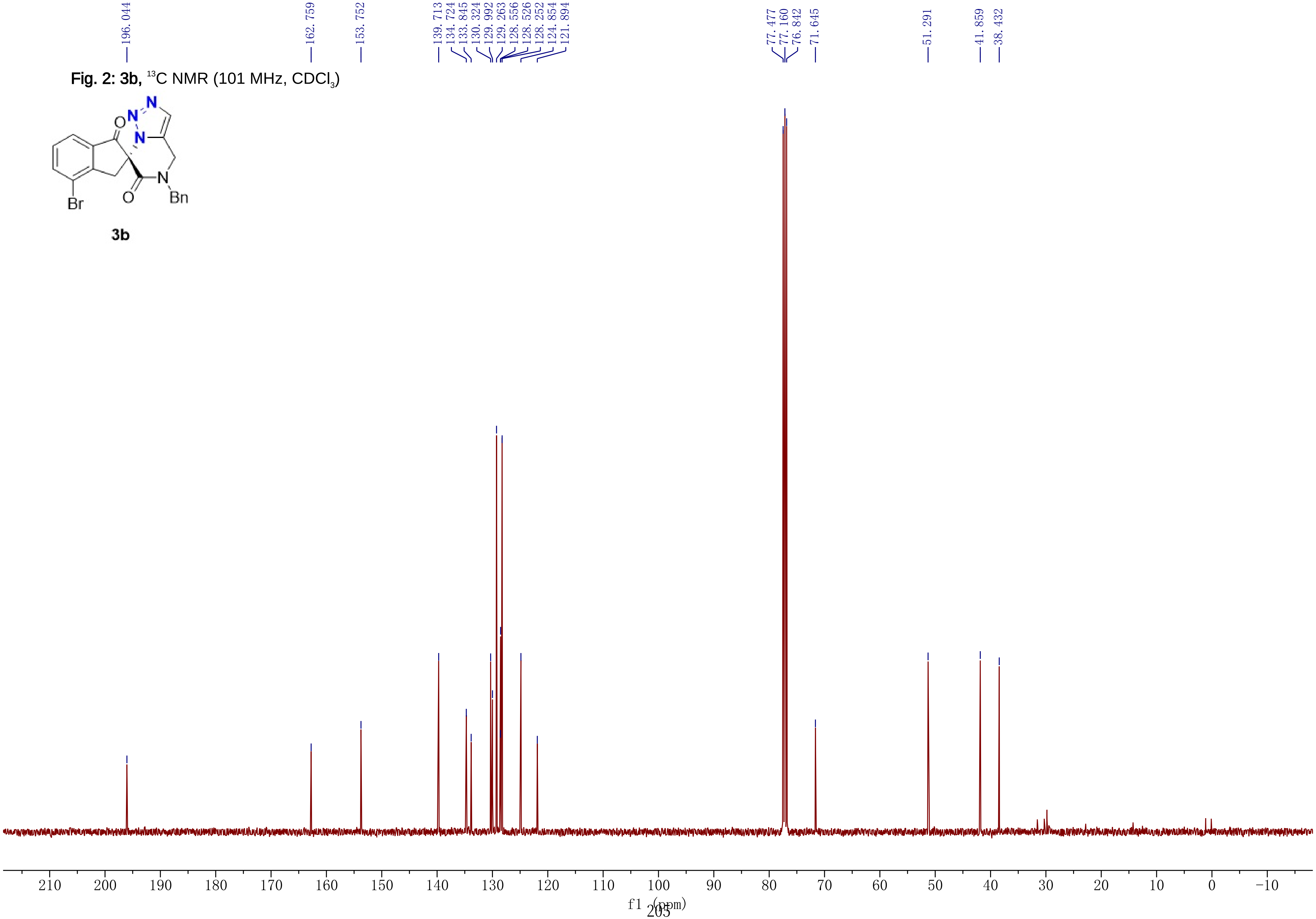

Fig. 2: 3b, ^{13}C NMR (101 MHz, $CDCl_3$)

3b

Fig. 2: 3c, ^{1}H NMR (400 MHz, $CDCl_3$)

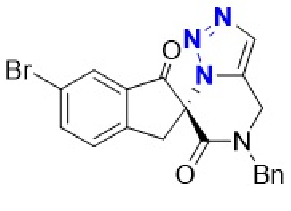

3c

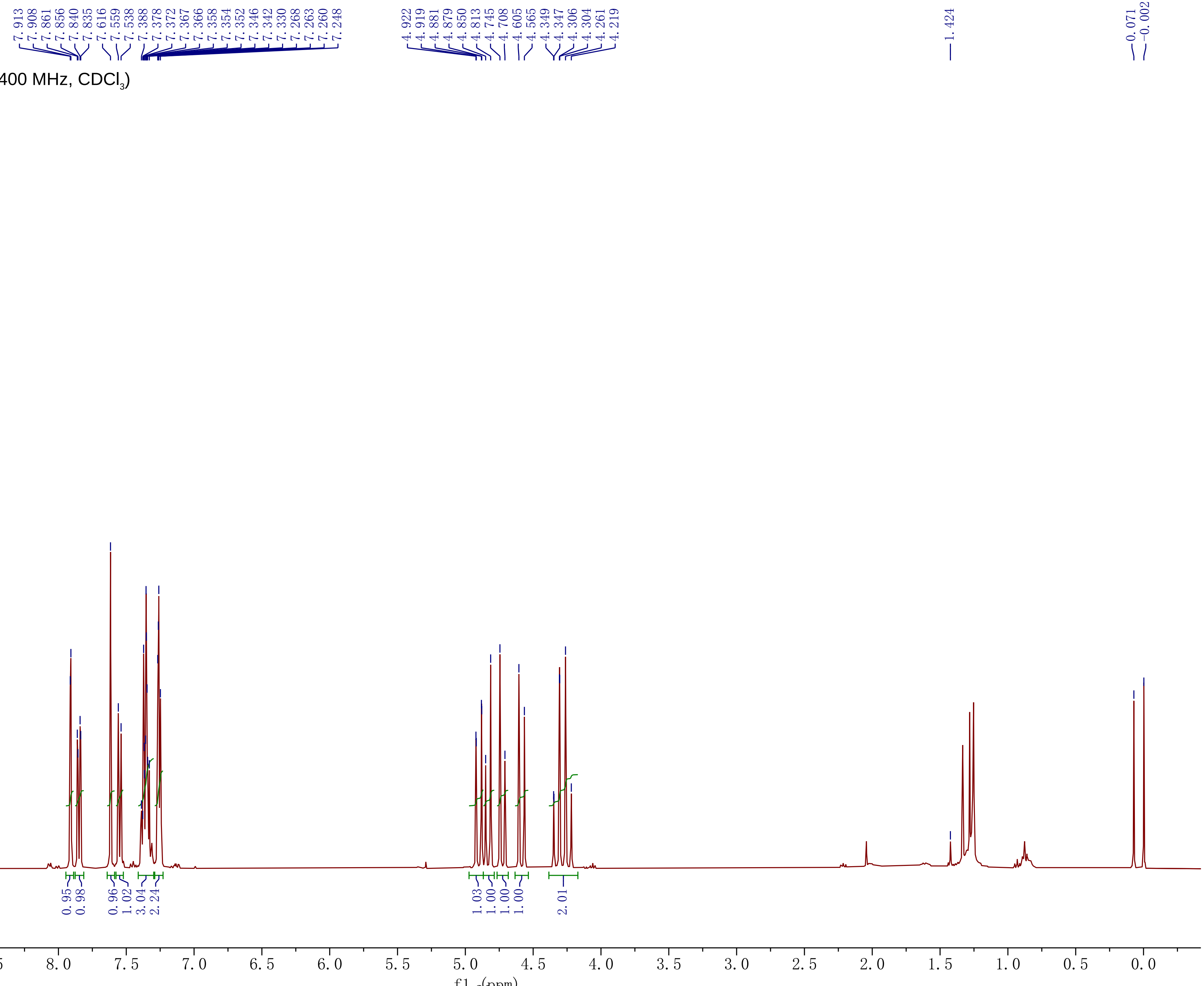

Fig. 2: 3c, ^{13}C NMR (101 MHz, $CDCl_3$)

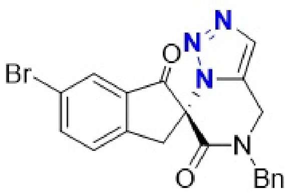

3c

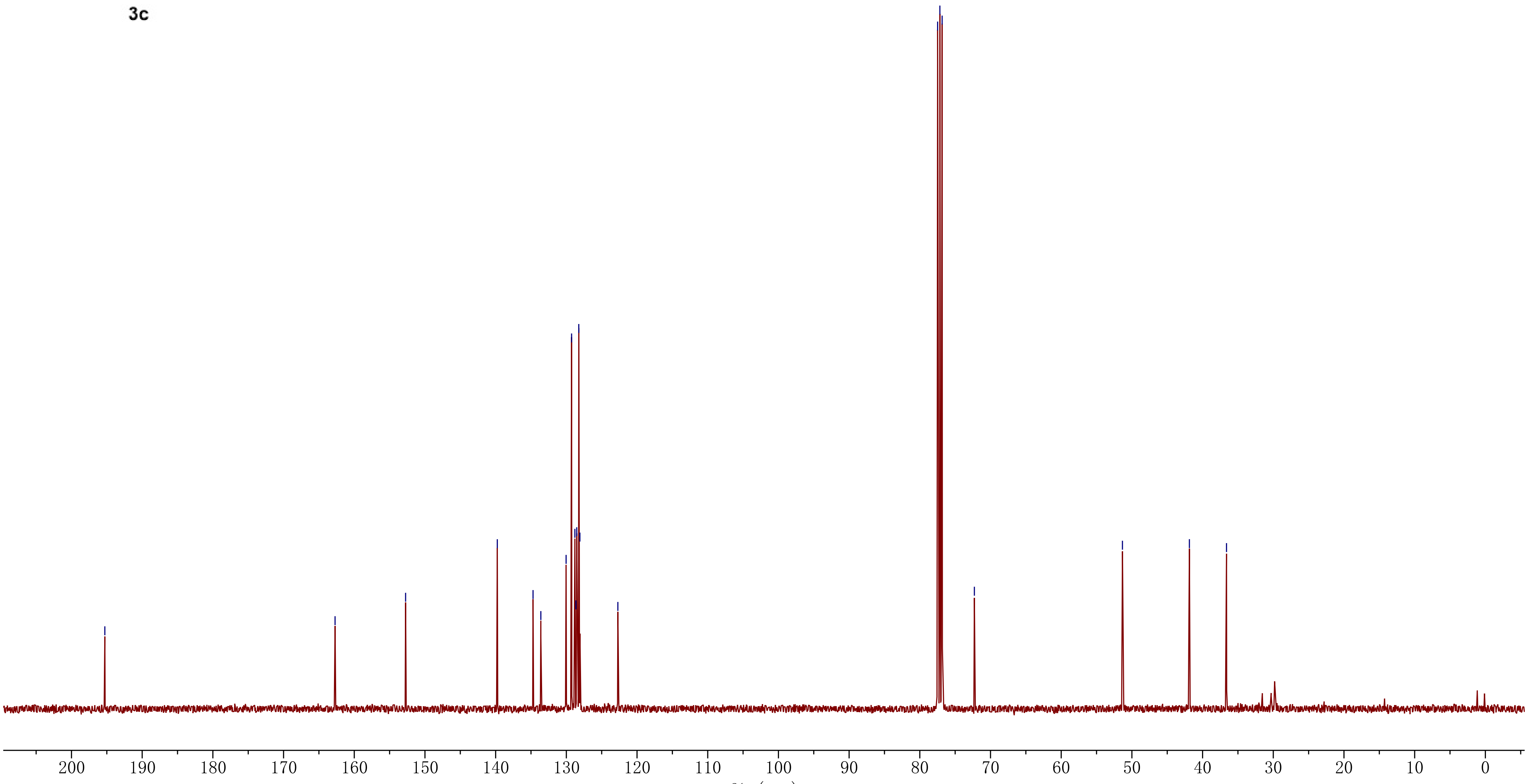

Fig. 2: 3d, ^{1}H NMR (400 MHz, $CDCl_3$)

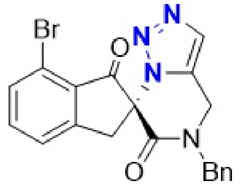

3d

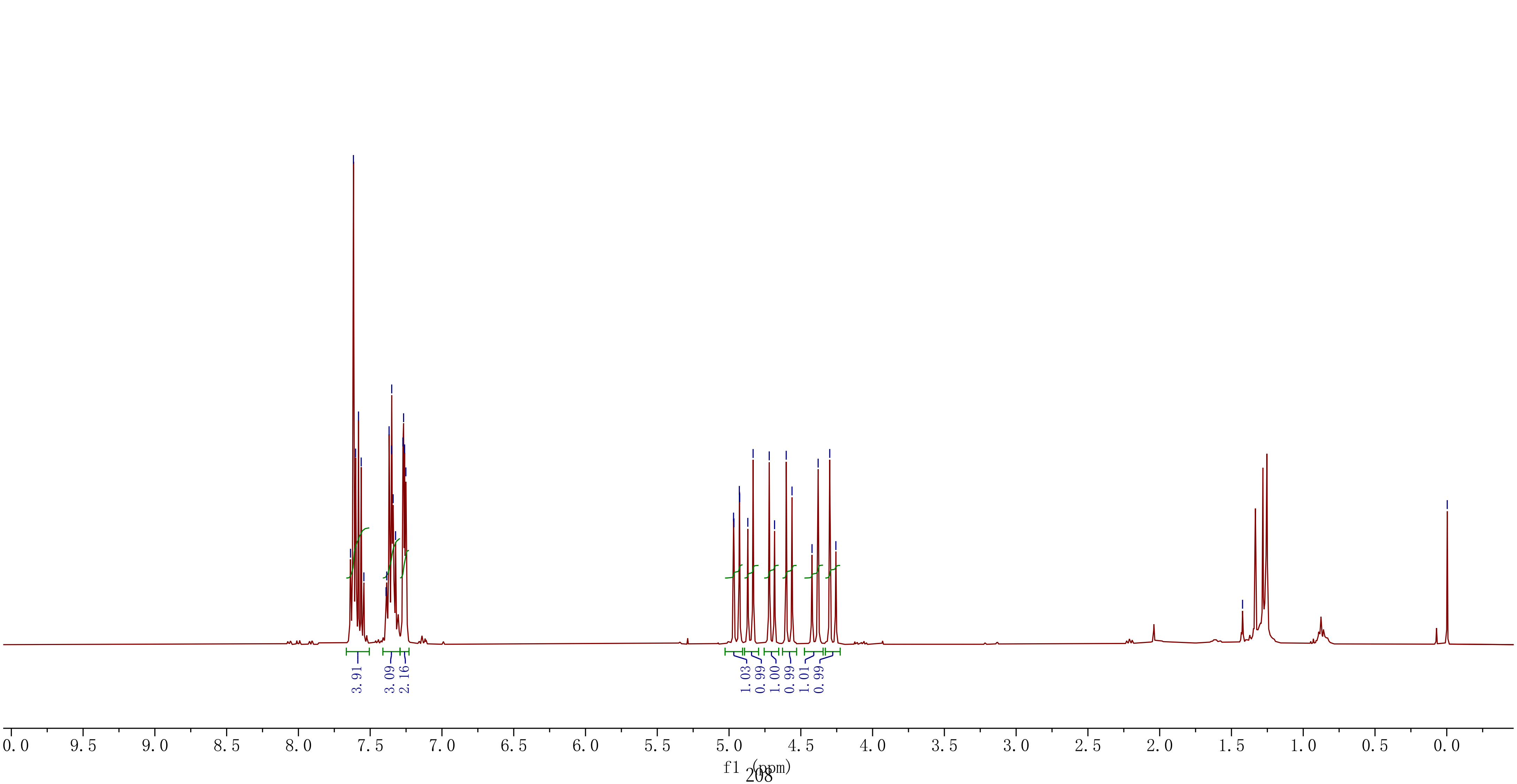

Fig. 2: 3d, ^{13}C NMR (101 MHz, $CDCl_3$)

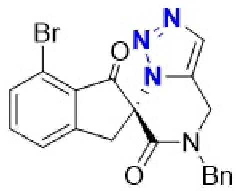

3d

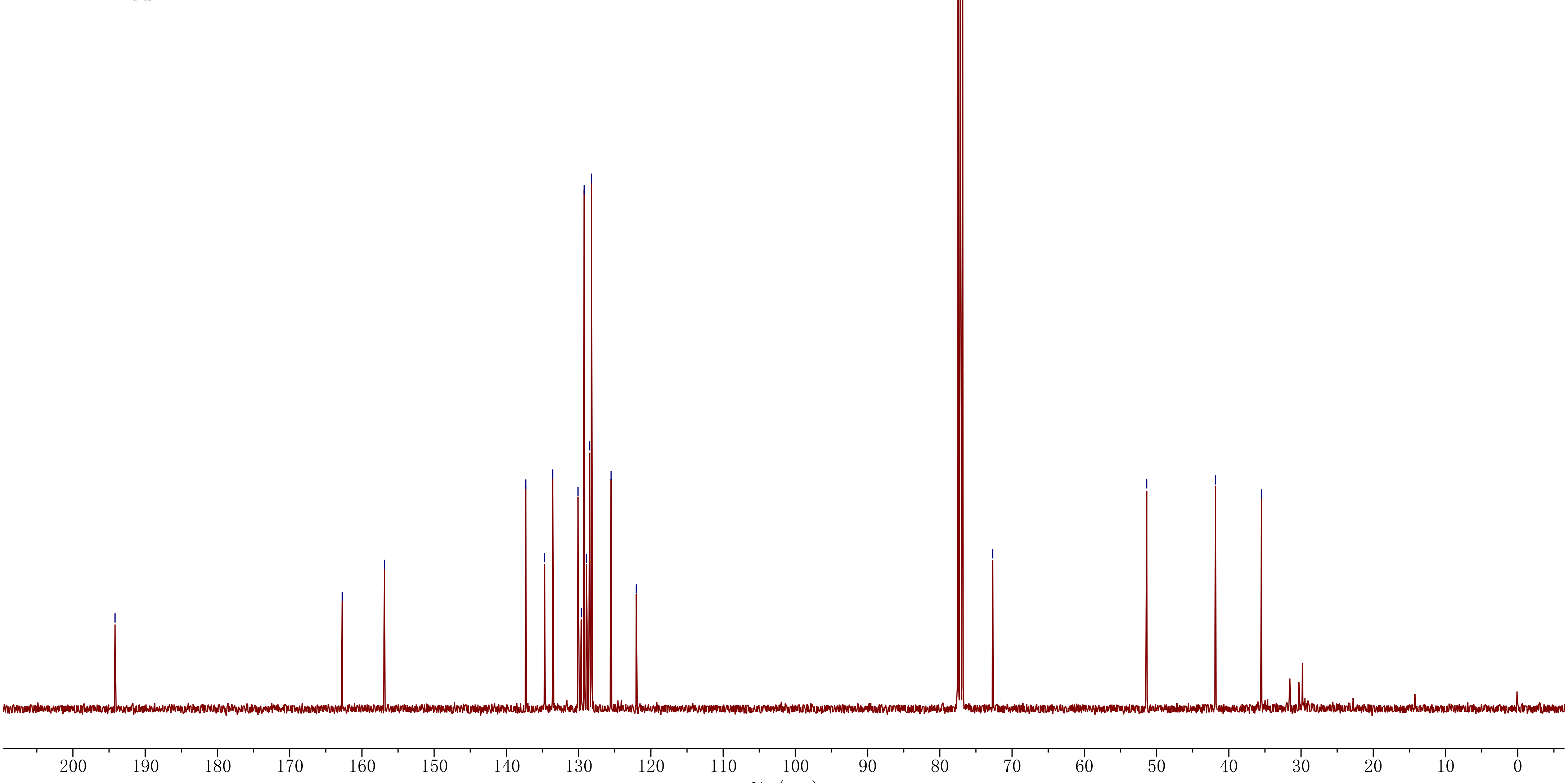

Fig. 2: 3e, ¹H NMR (400 MHz, $CDCl_3$)

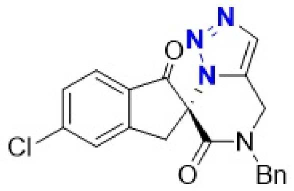

3e

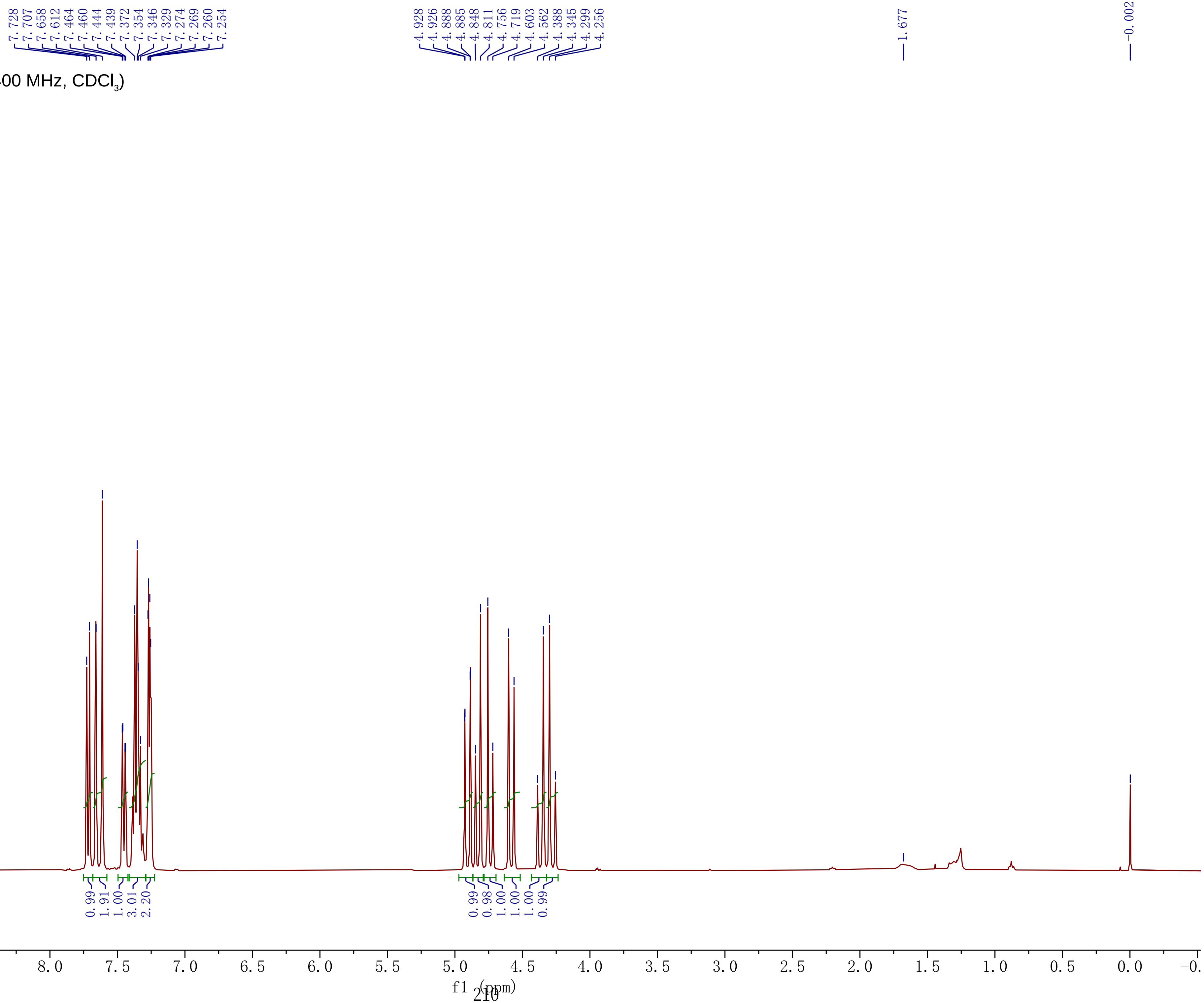

Fig. 2: 3e, ^{13}C NMR (101 MHz, $CDCl_3$)

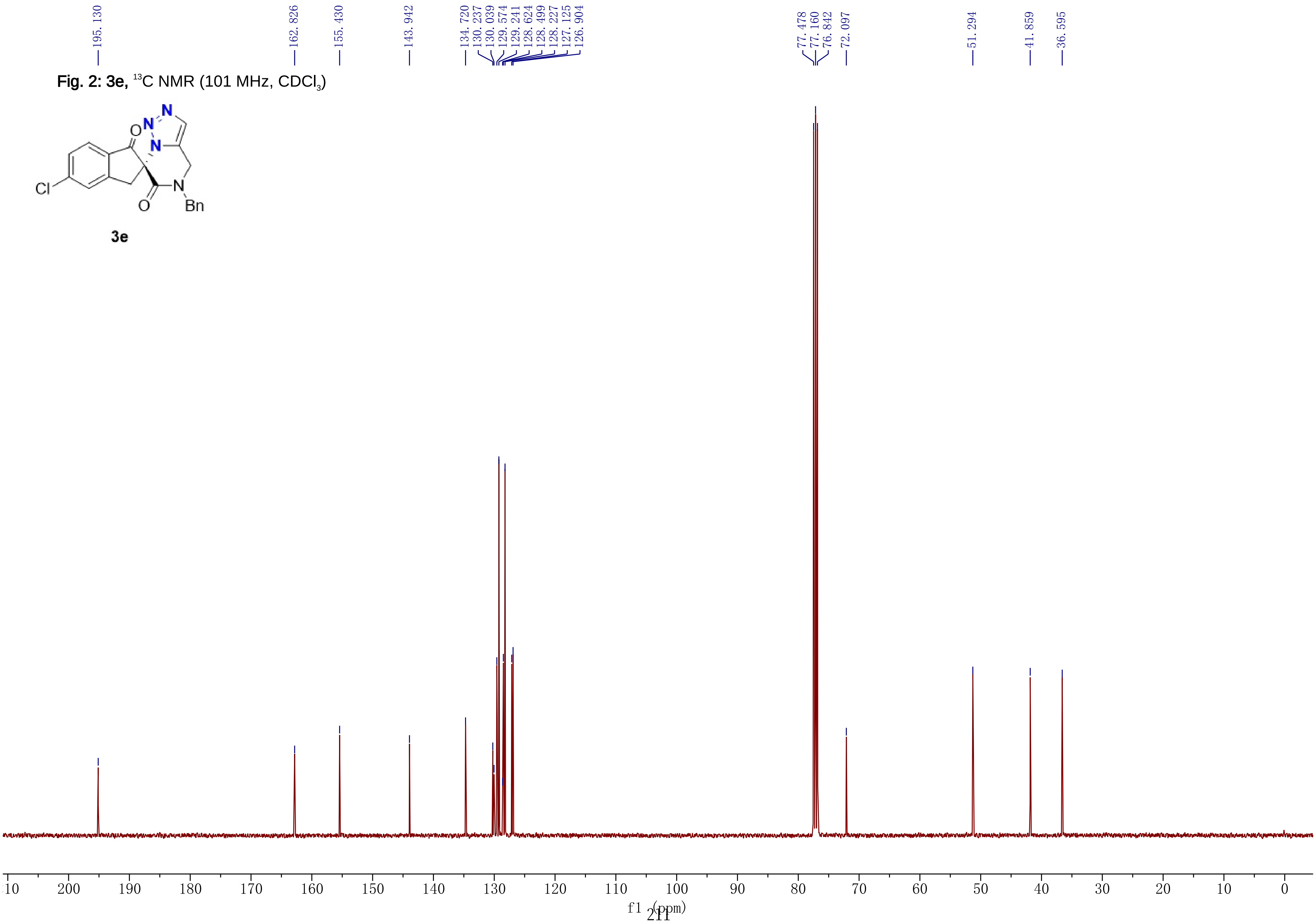

Fig. 2: 3f, ^{1}H NMR (400 MHz, $CDCl_3$)

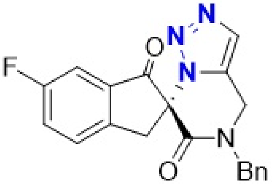

3f

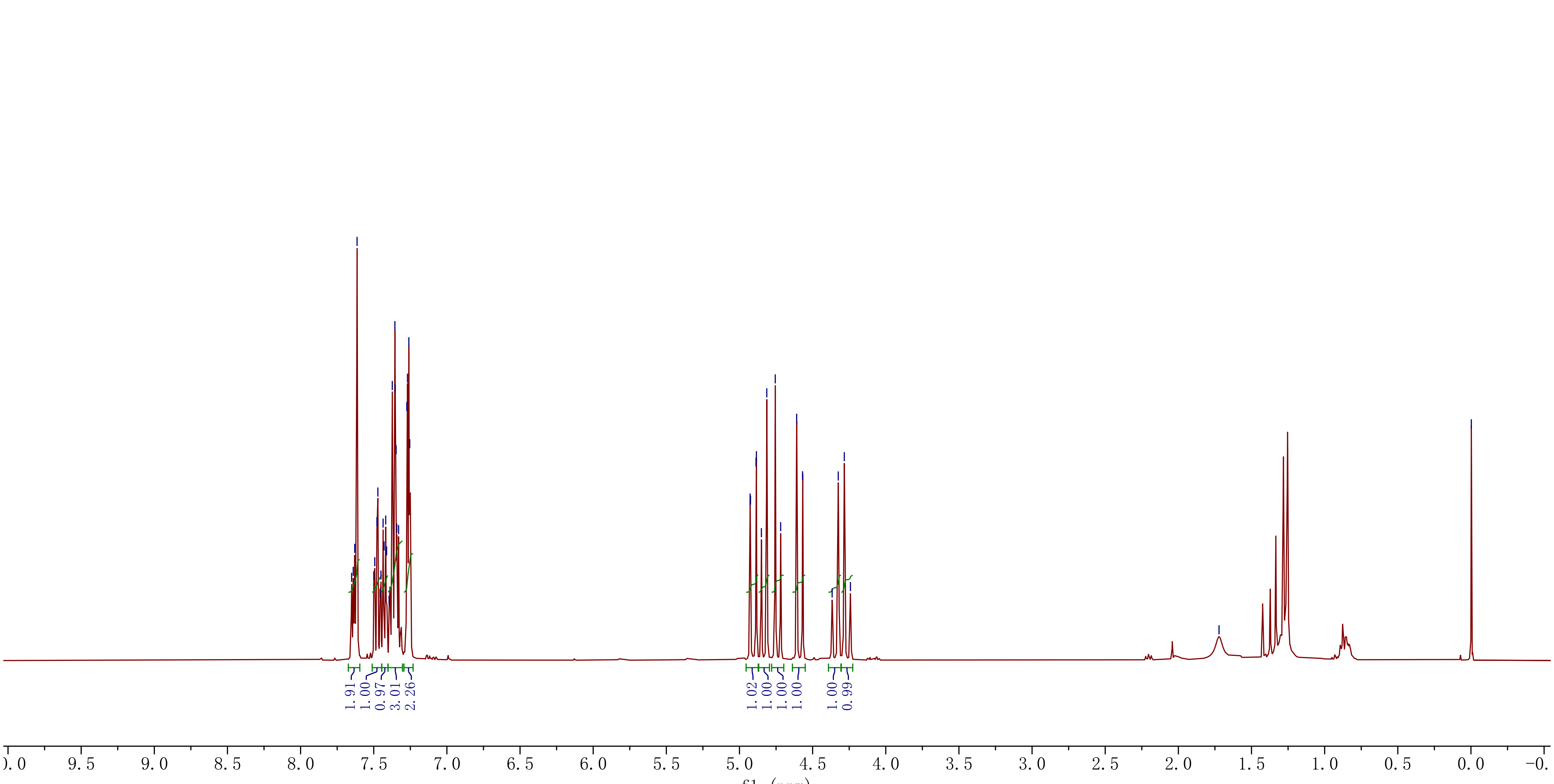

Fig. 2: 3f, ^{19}F NMR (376 MHz, $CDCl_3$)

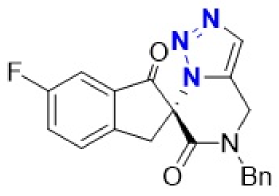
3f

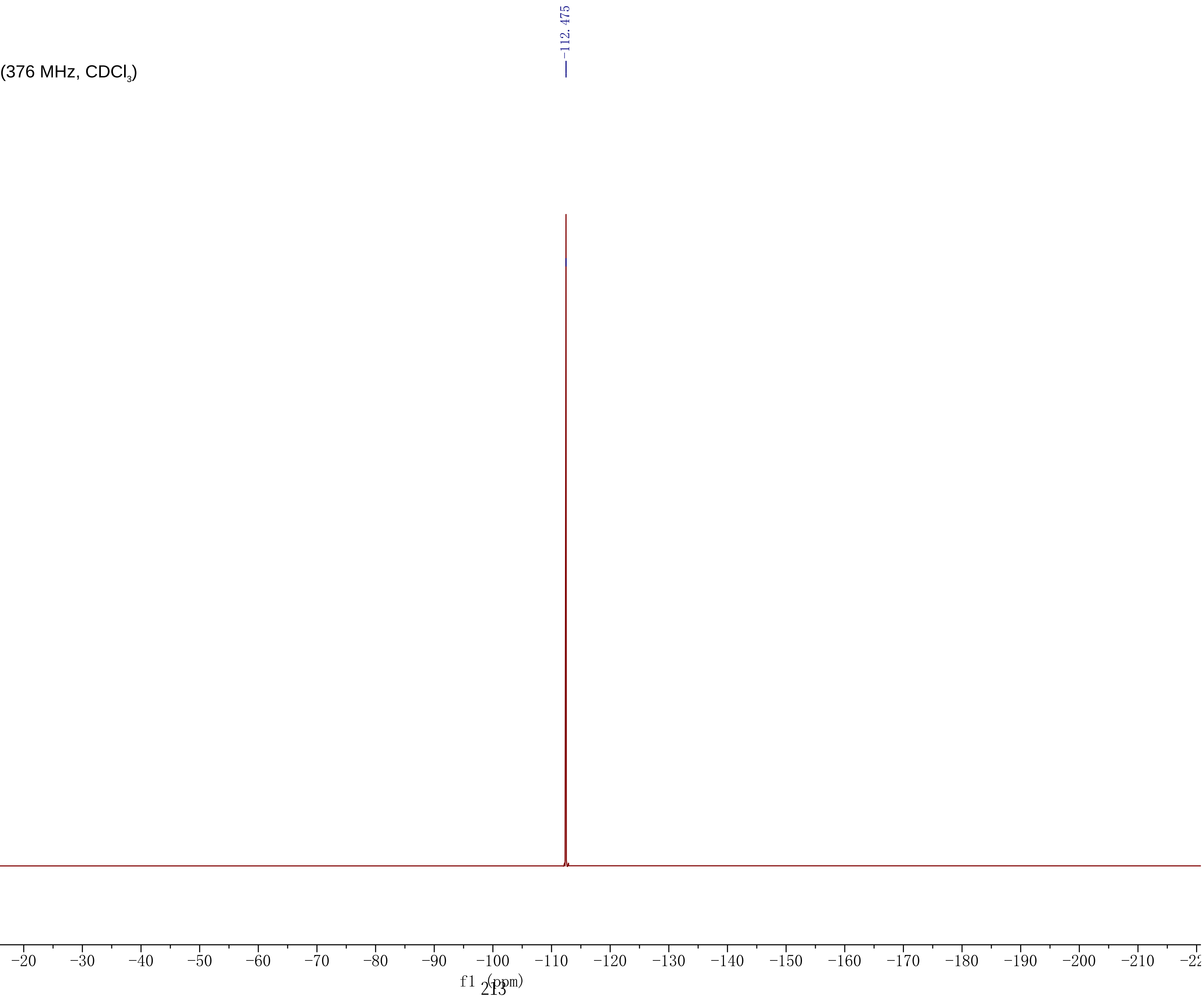

Fig. 2: 3f, ^{13}C NMR (101 MHz, $CDCl_3$)

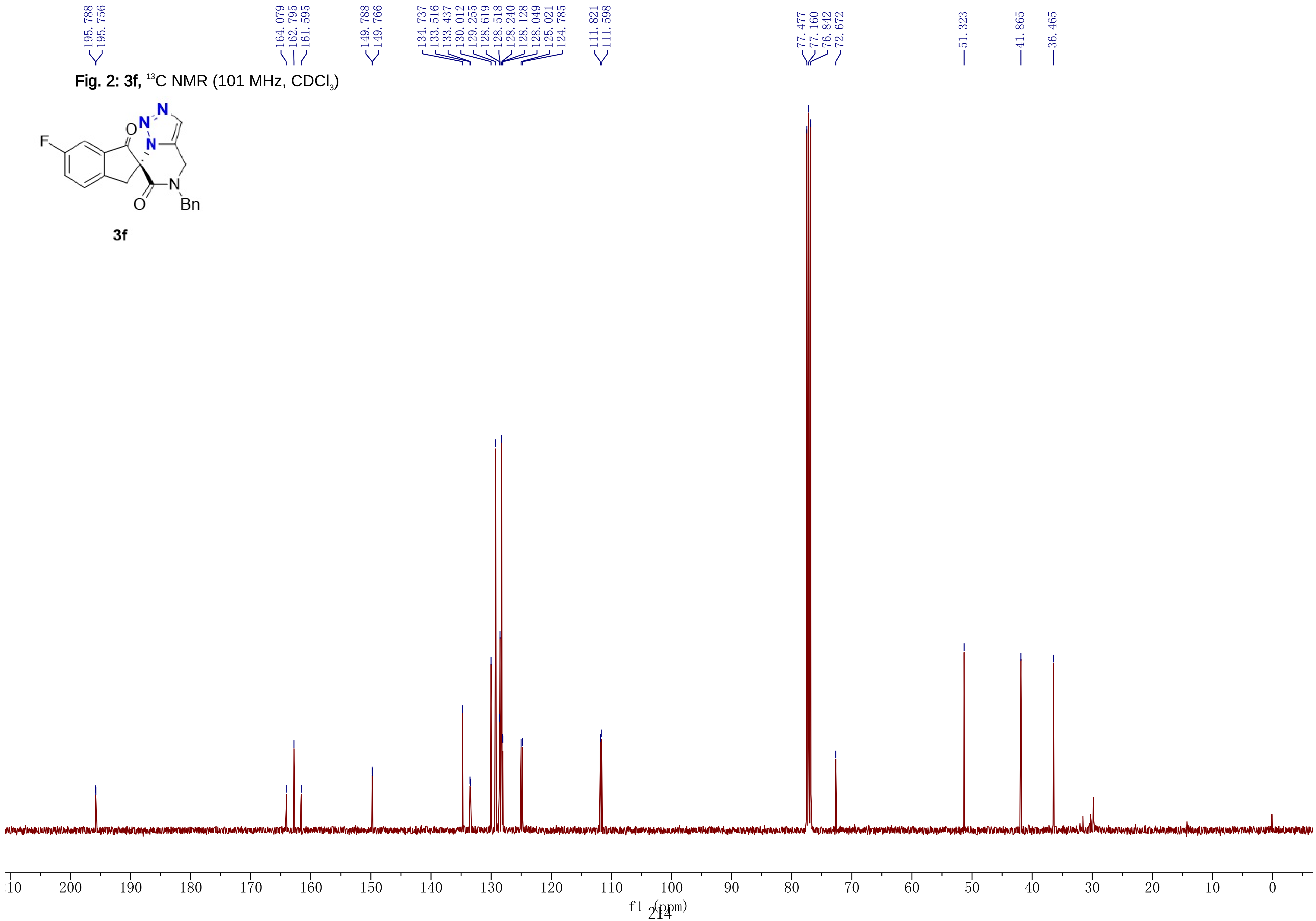

Fig. 2: 3g, ^{1}H NMR (400 MHz, $CDCl_3$)

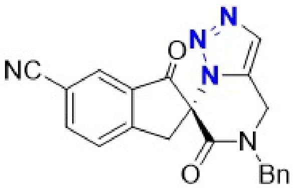

3g

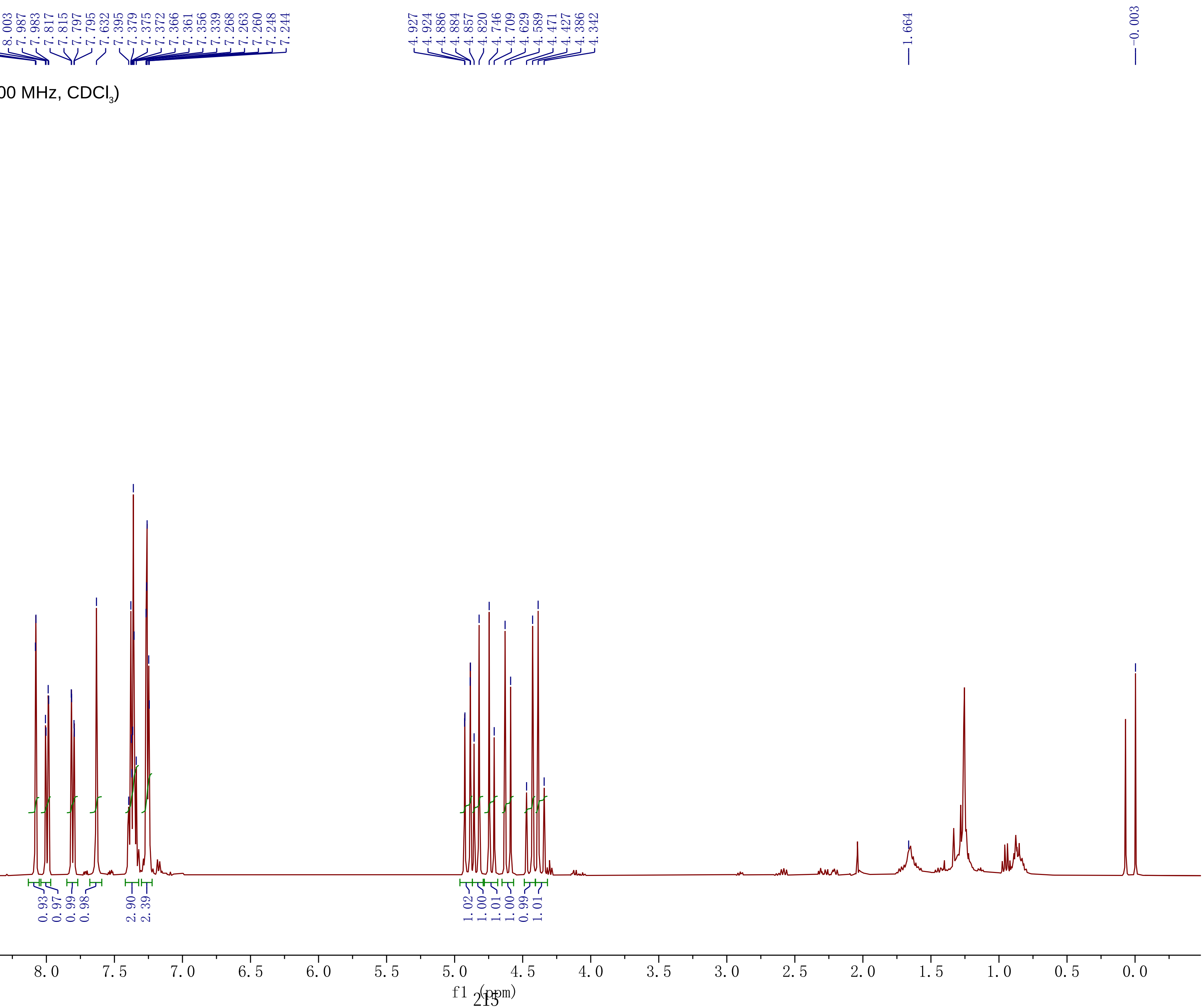

Fig. 2: 3g, ^{13}C NMR (101 MHz, $CDCl_3$)

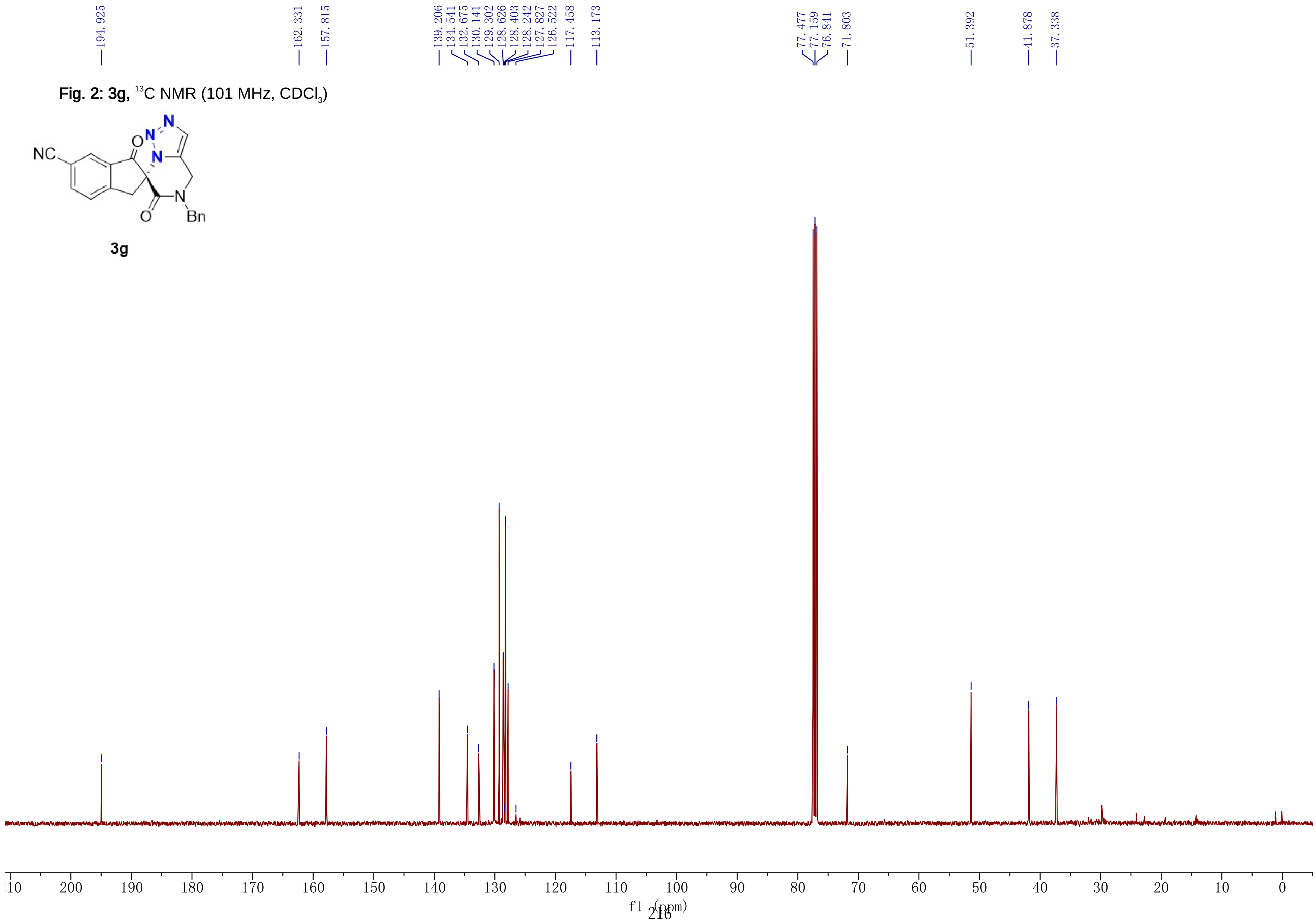

Fig. 2: 3h, ^{1}H NMR (400 MHz, $CDCl_3$)

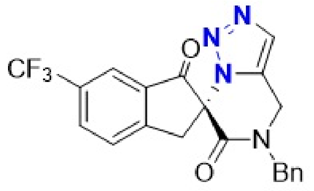

3h

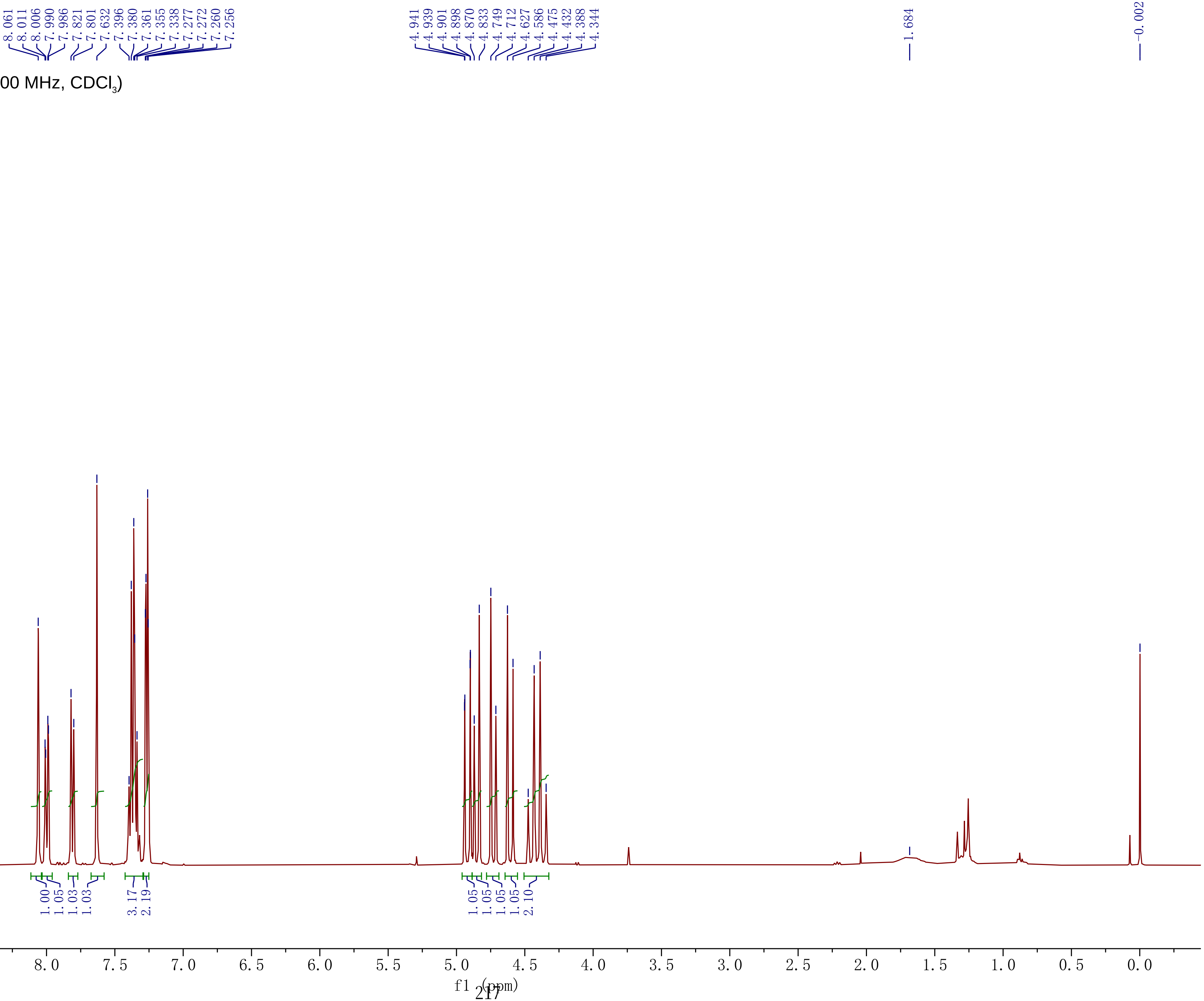

Fig. 2: 3h, ^{19}F NMR (376 MHz, $CDCl_3$)

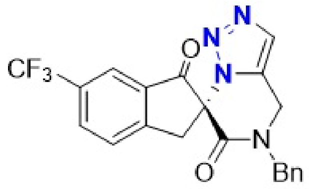

3h

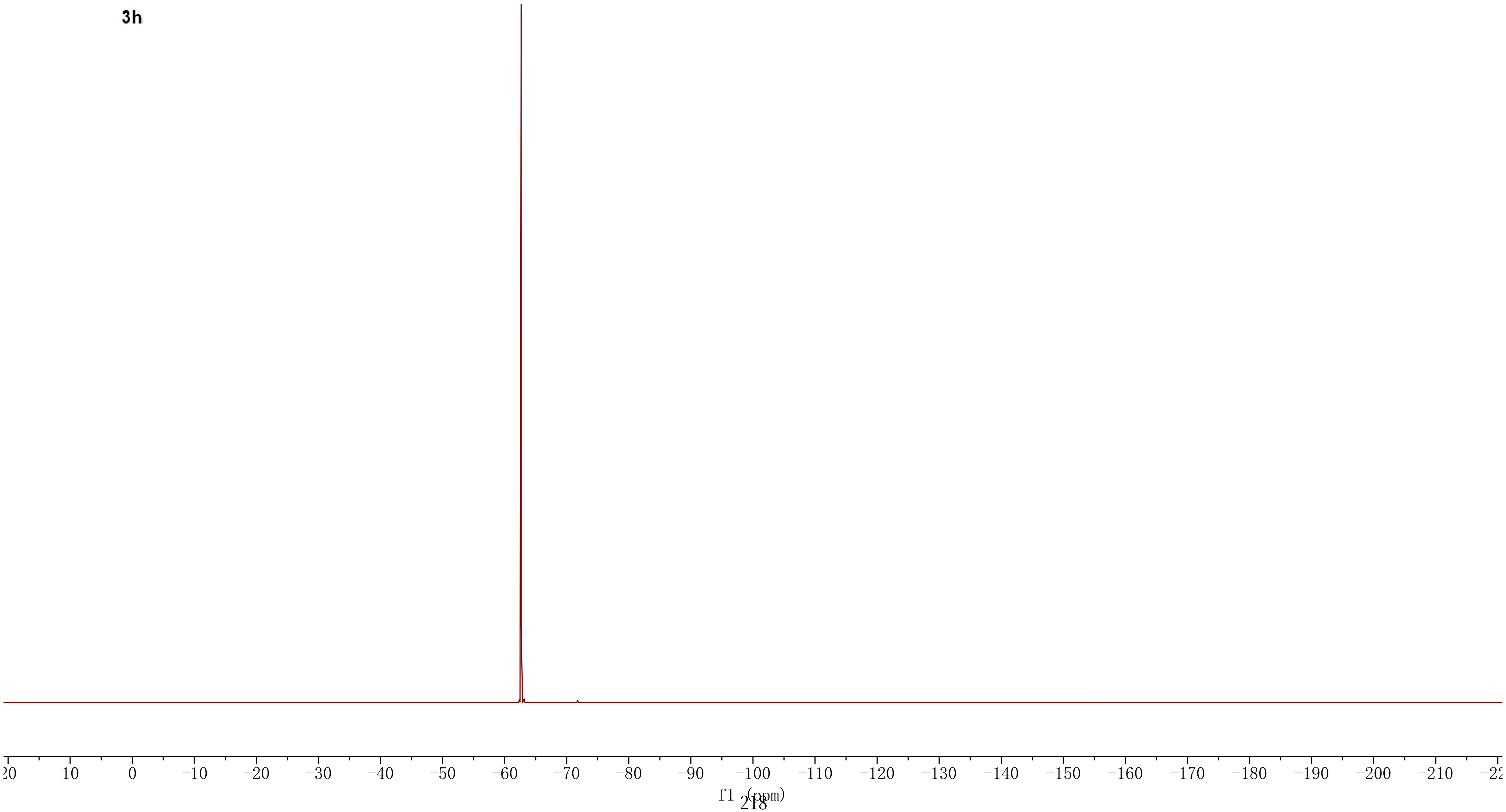

Fig. 2: 3h, ^{13}C NMR (101 MHz, $CDCl_3$)

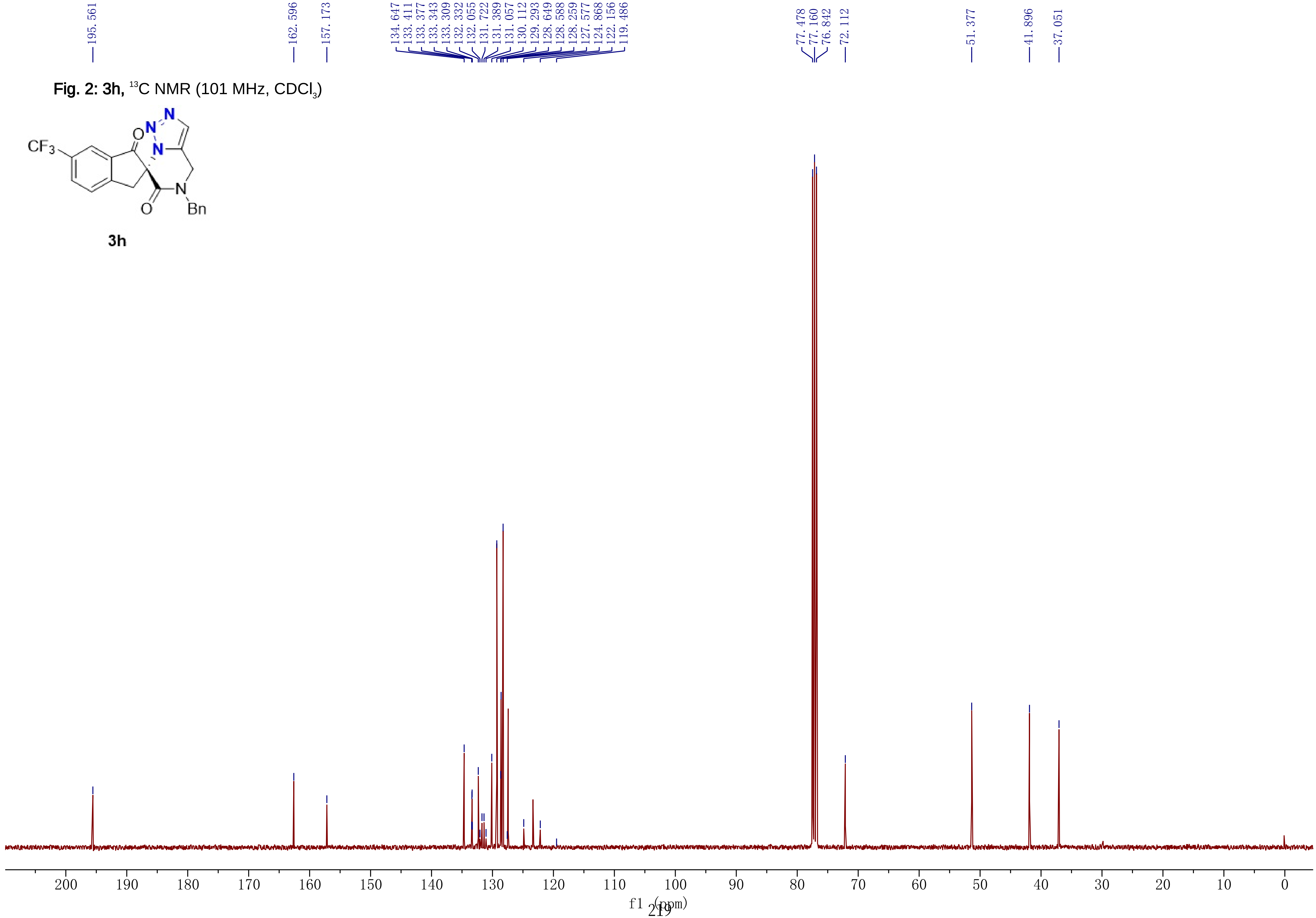

3h

Fig. 2: 3i, ^{1}H NMR (400 MHz, $CDCl_3$)

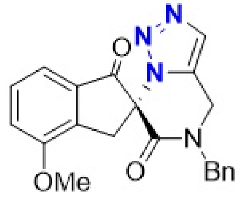

3i

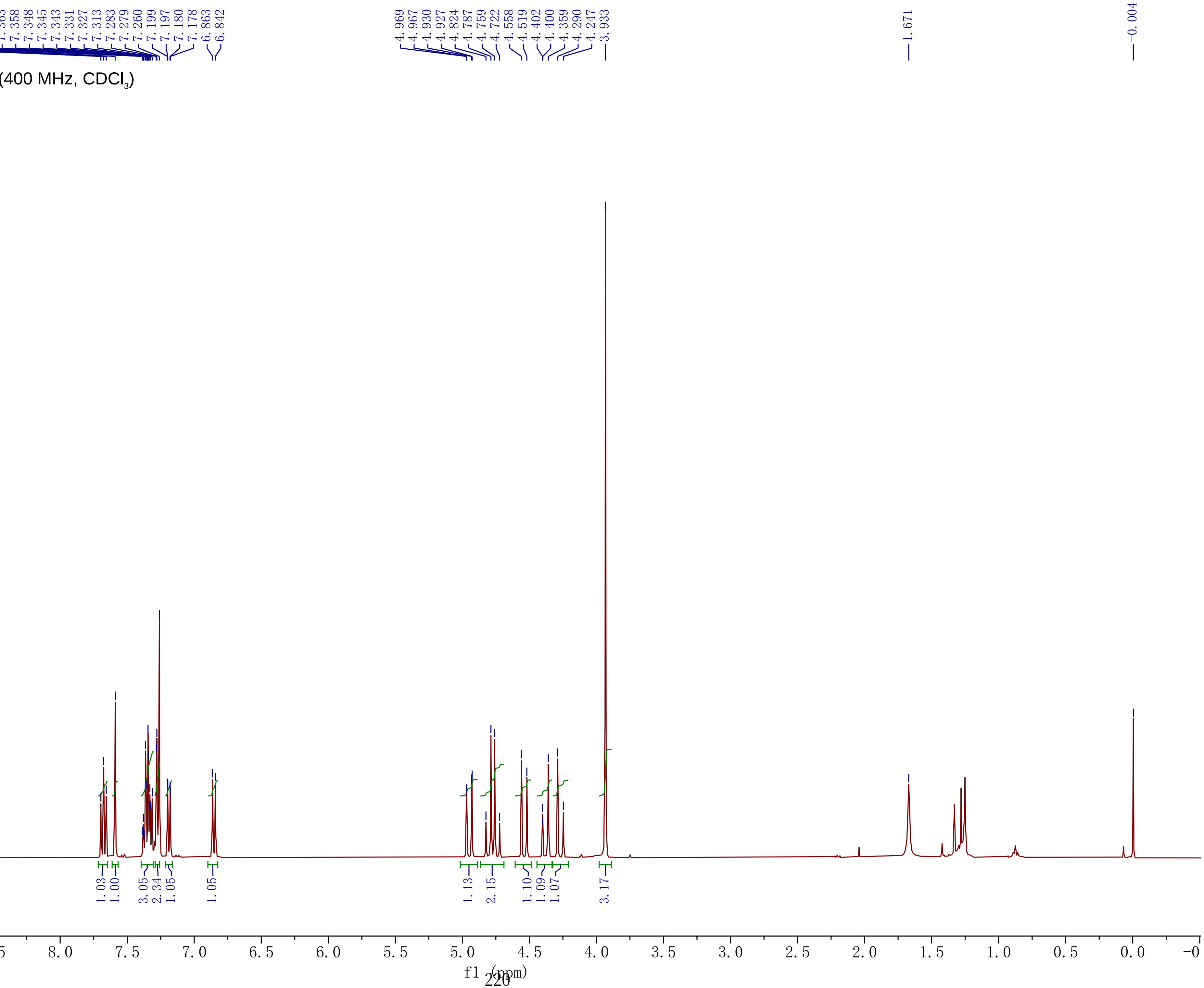

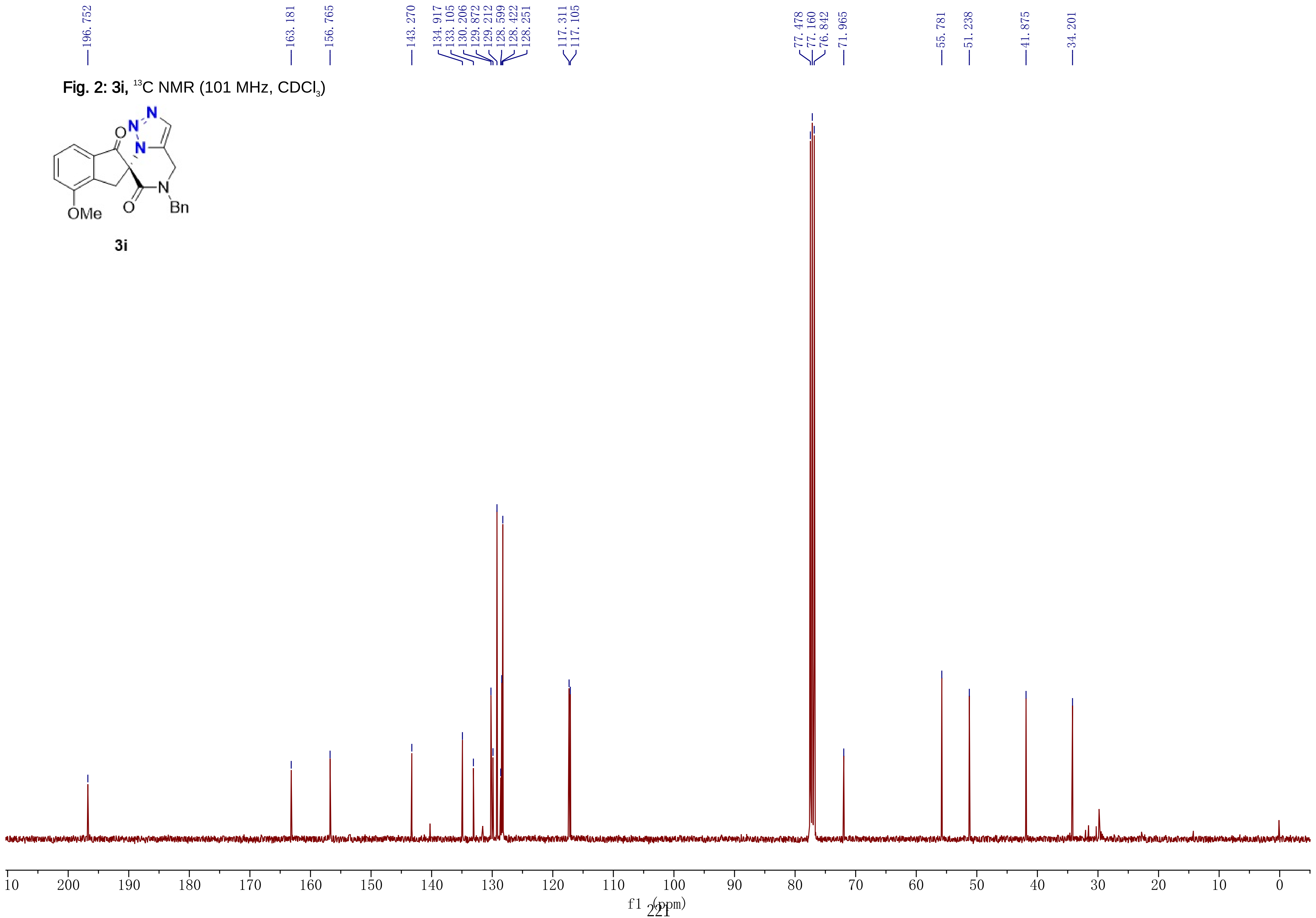

Fig. 2: 3i, ^{13}C NMR (101 MHz, $CDCl_3$)

Fig. 2: 3j, ^{1}H NMR (400 MHz, $CDCl_3$)

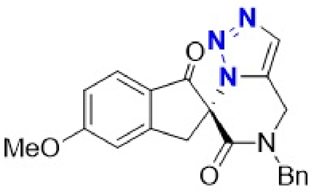

3j

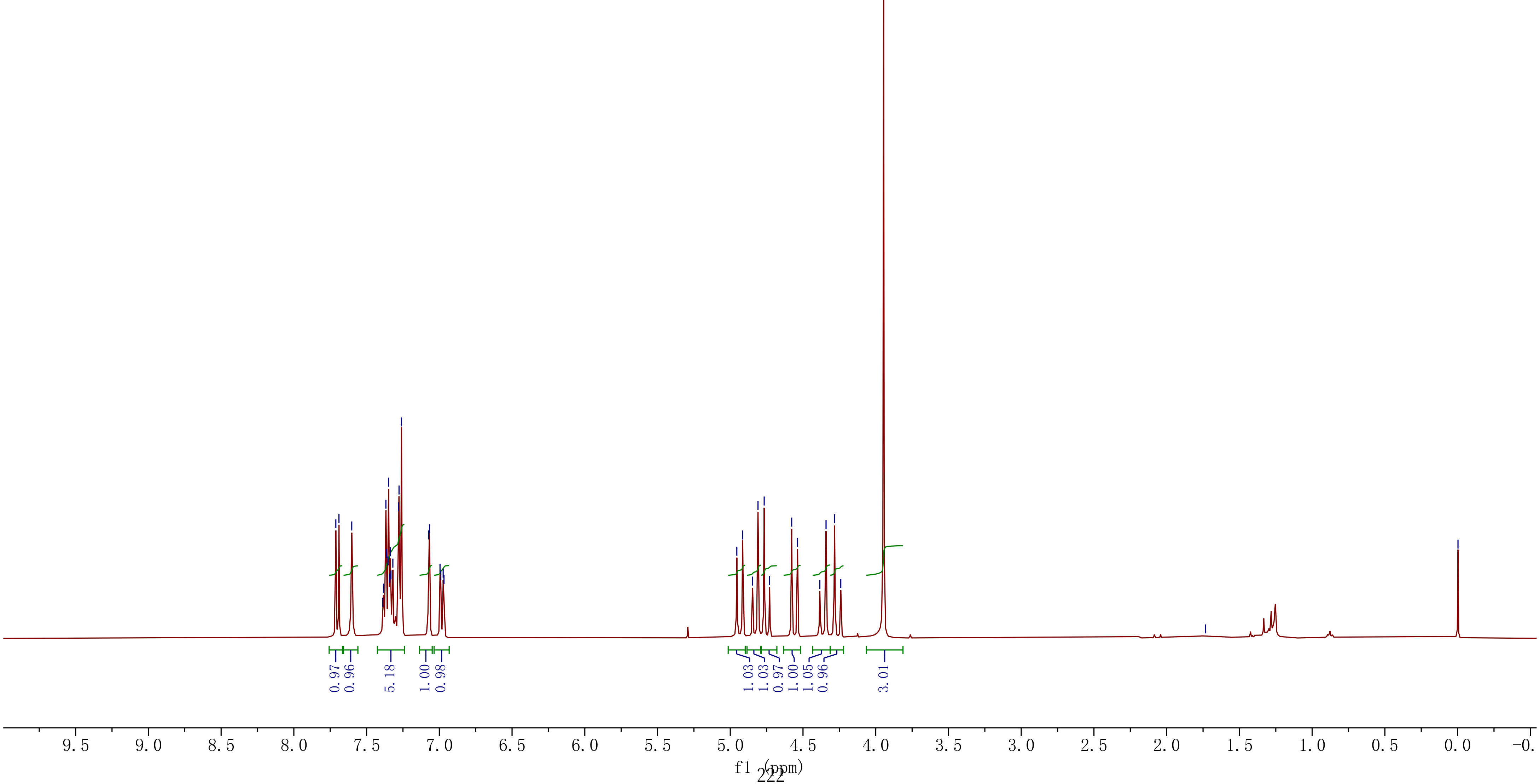

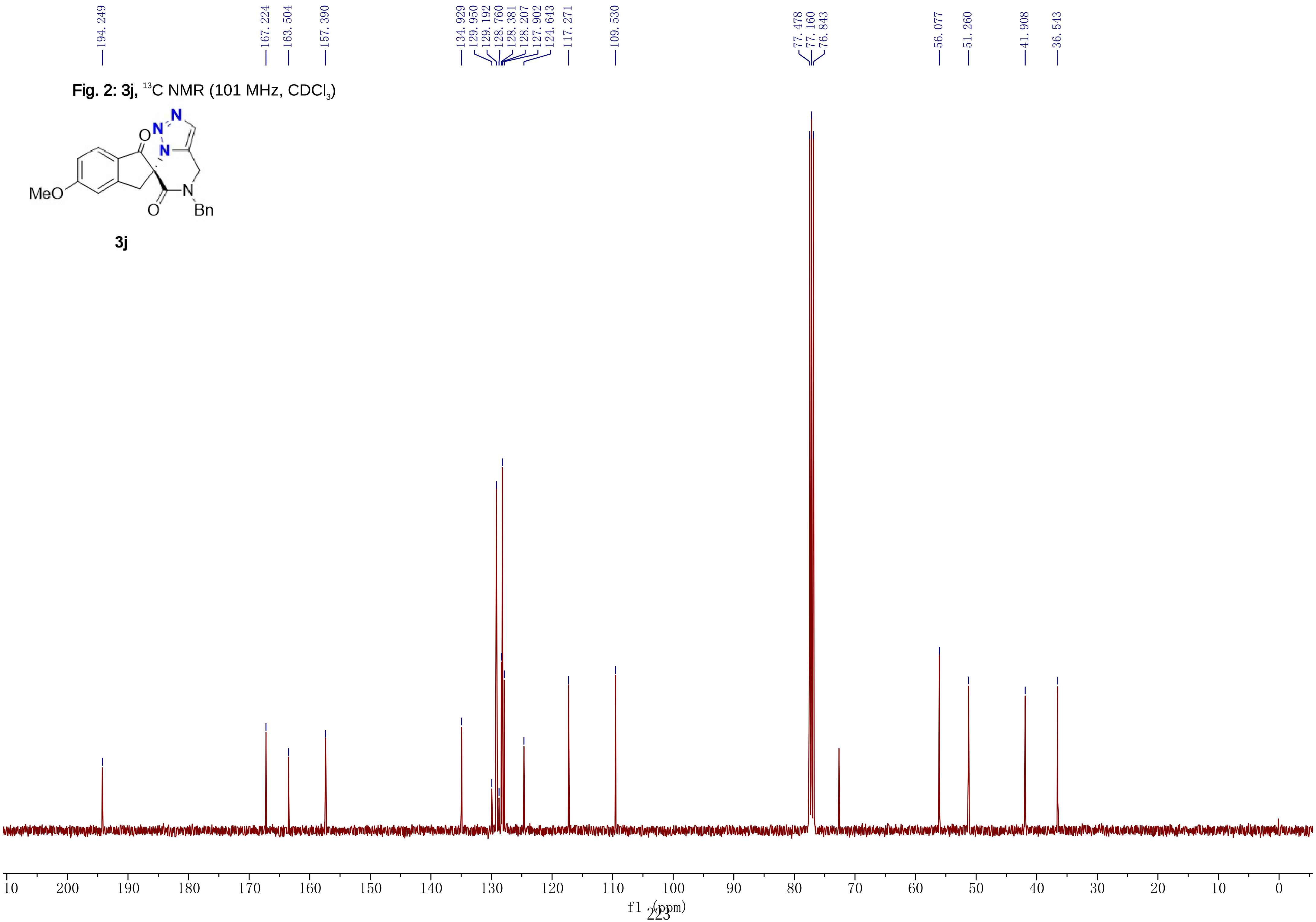

Fig. 2: 3j, ^{13}C NMR (101 MHz, $CDCl_3$)

Fig. 2: 3k, ^{1}H NMR (400 MHz, $CDCl_3$)

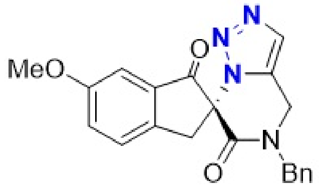

3k

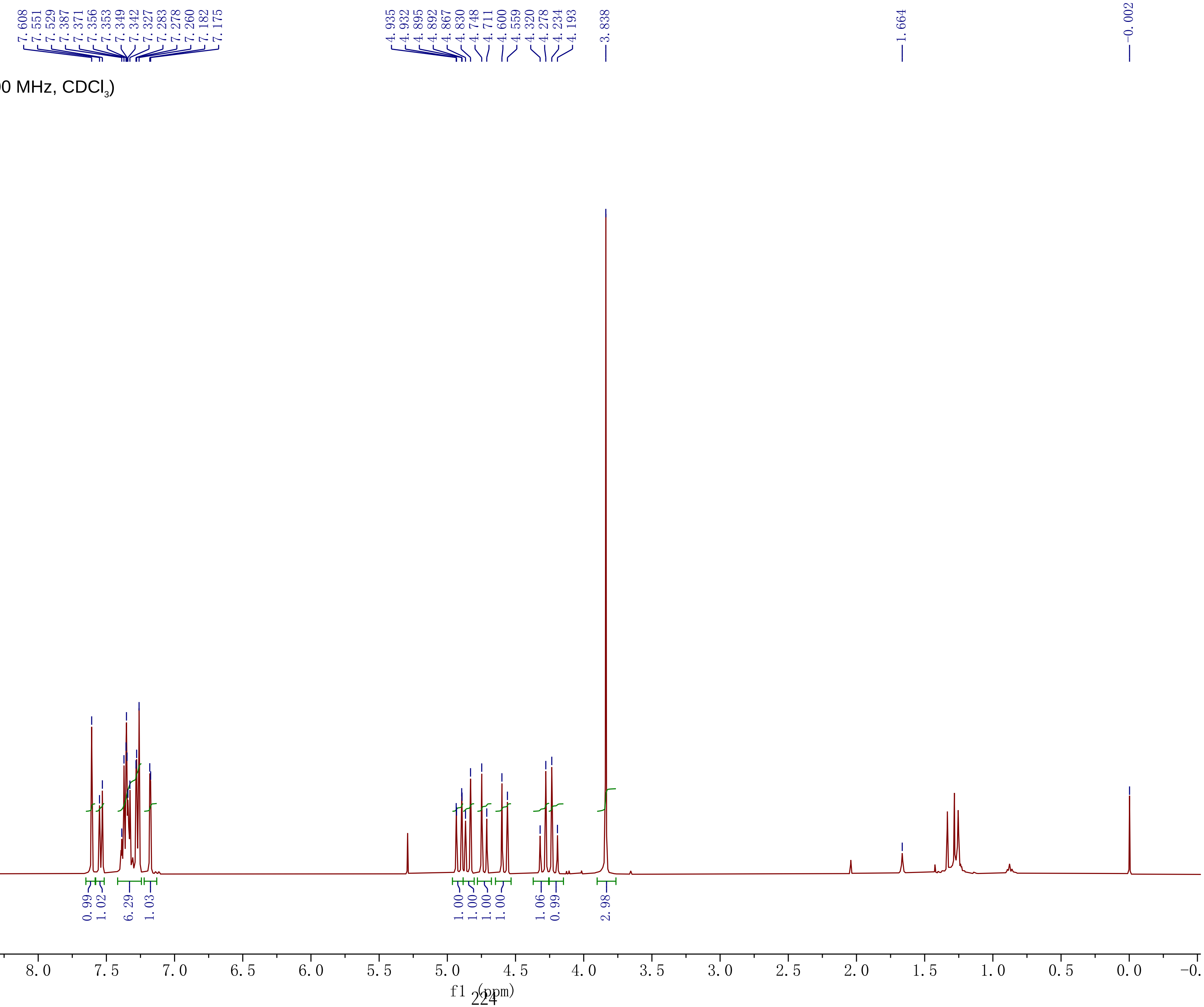

Fig. 2: **3k**, ^{13}C NMR (101 MHz, $CDCl_3$)

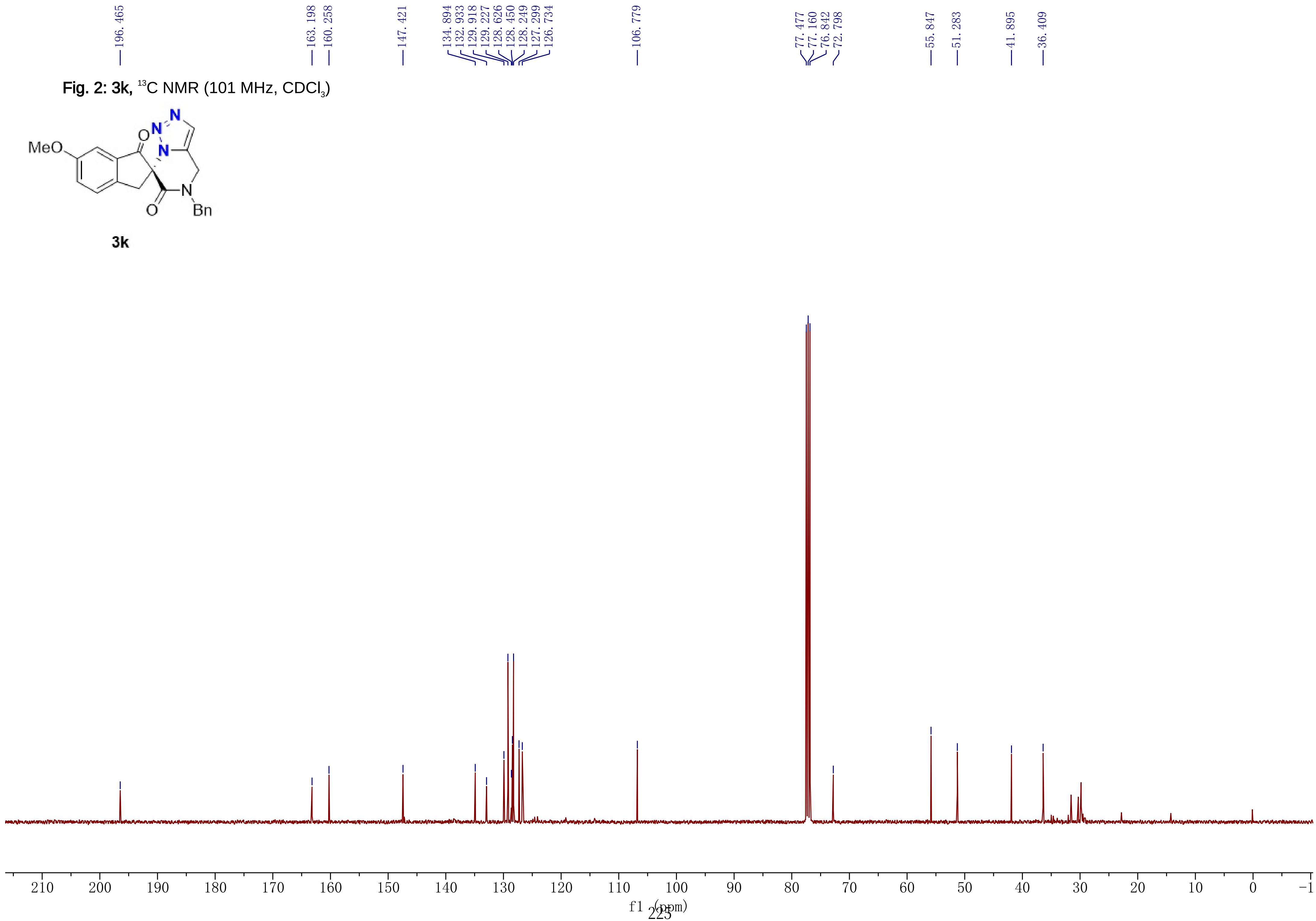

Fig. 2: 3l, ^1H NMR (400 MHz, $CDCl_3$)

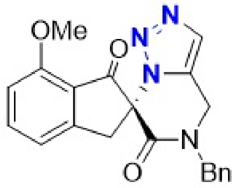

3l

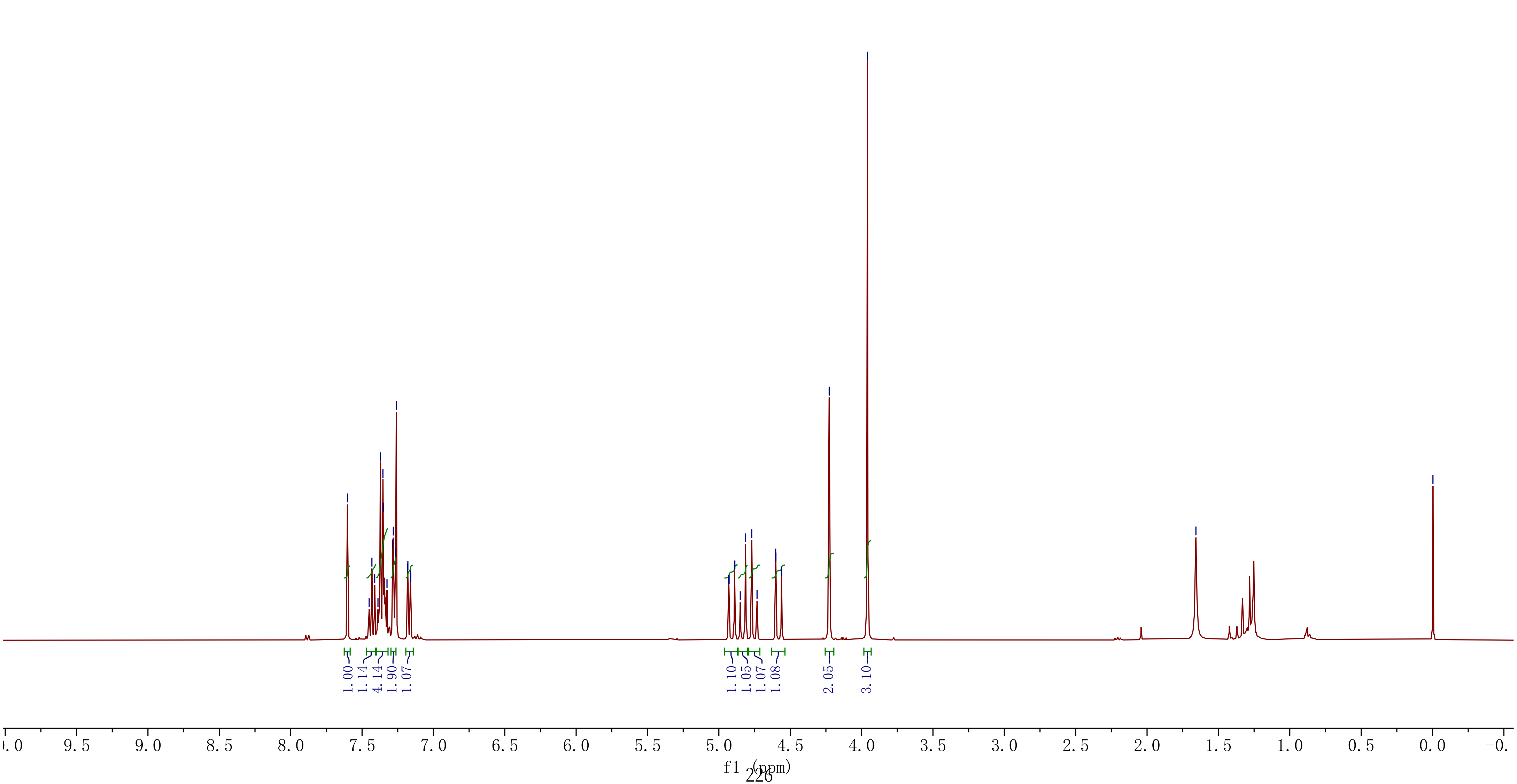

Fig. 2: 3l, ^{13}C NMR (101 MHz, $CDCl_3$)

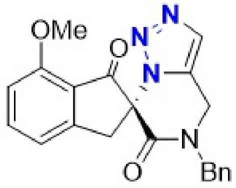

3l

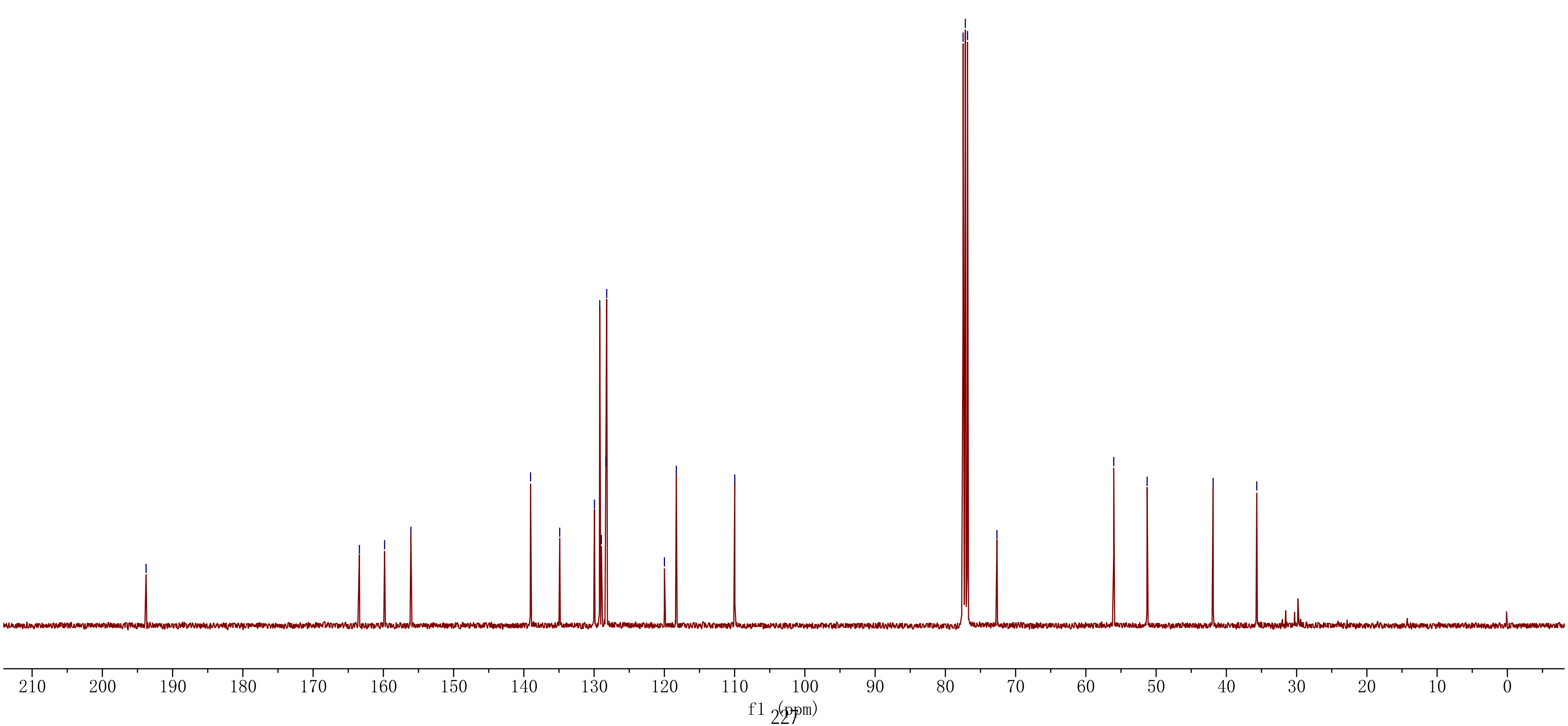

Fig. 2: 3m, ^{1}H NMR (400 MHz, $CDCl_3$)

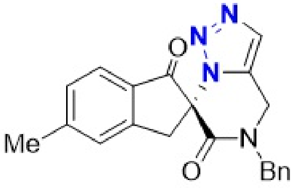

3m

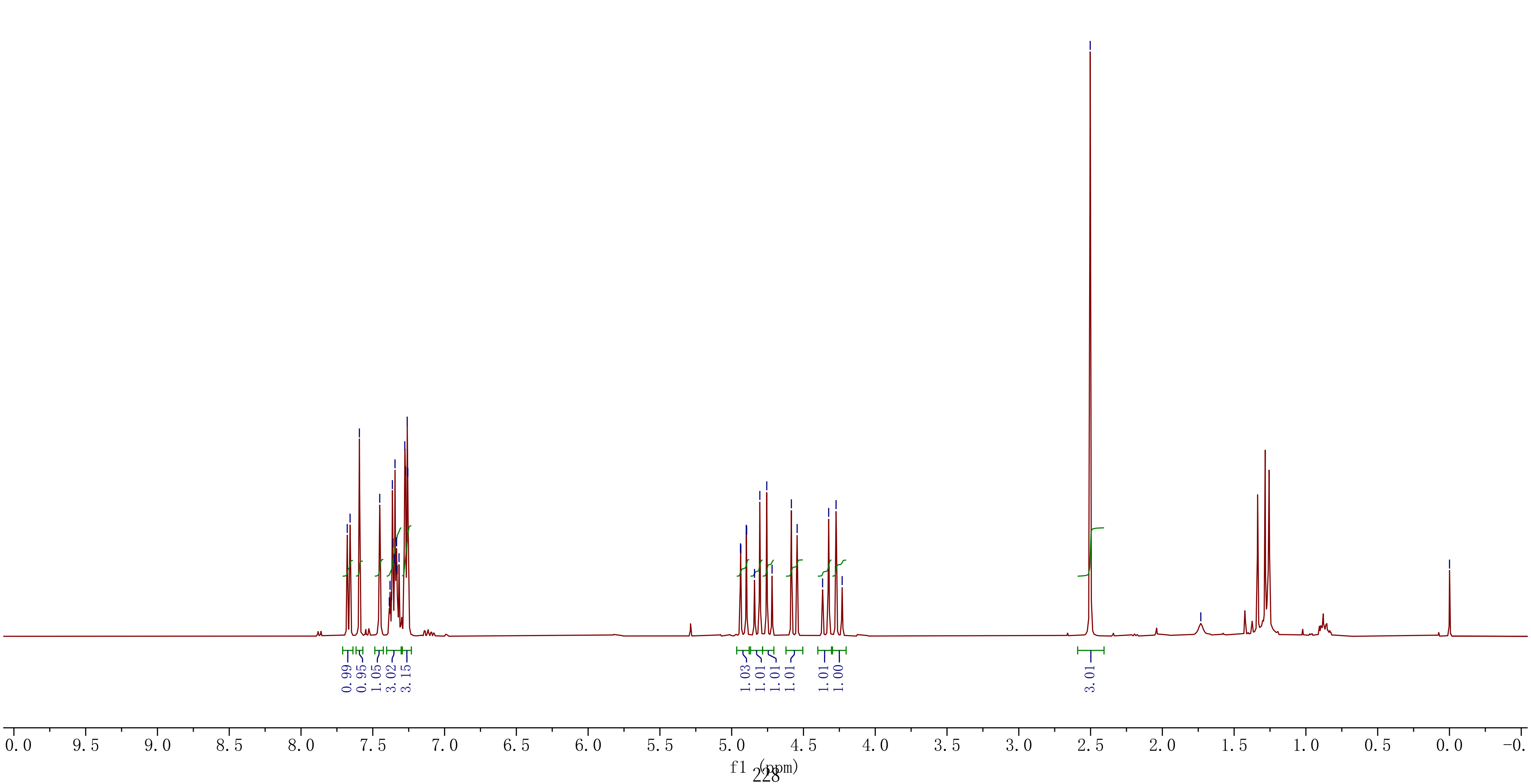

Fig. 2: 3m, ^{13}C NMR (101 MHz, $CDCl_3$)

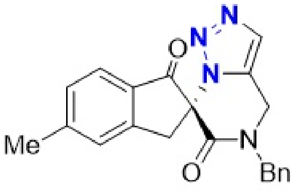

3m

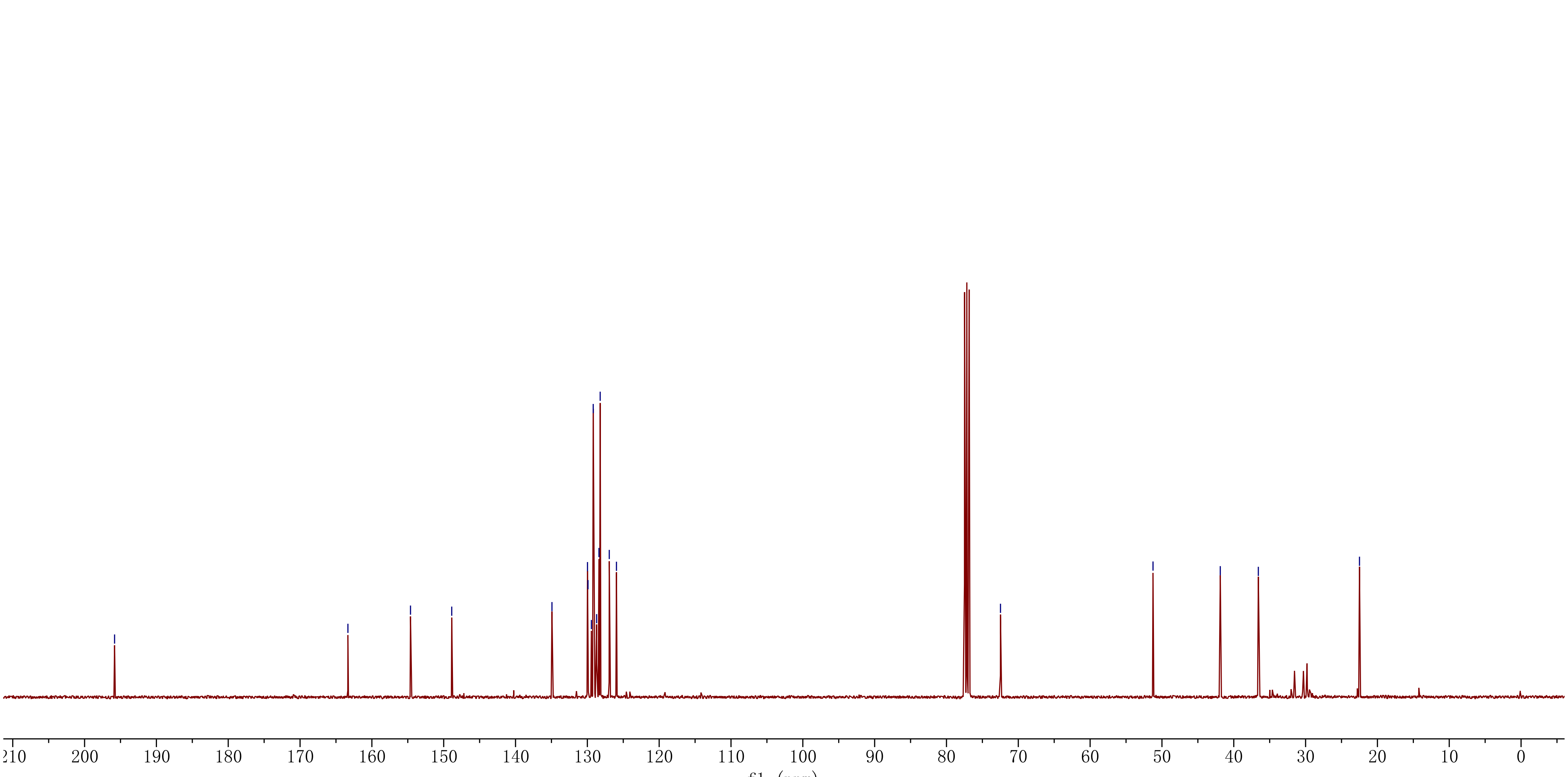

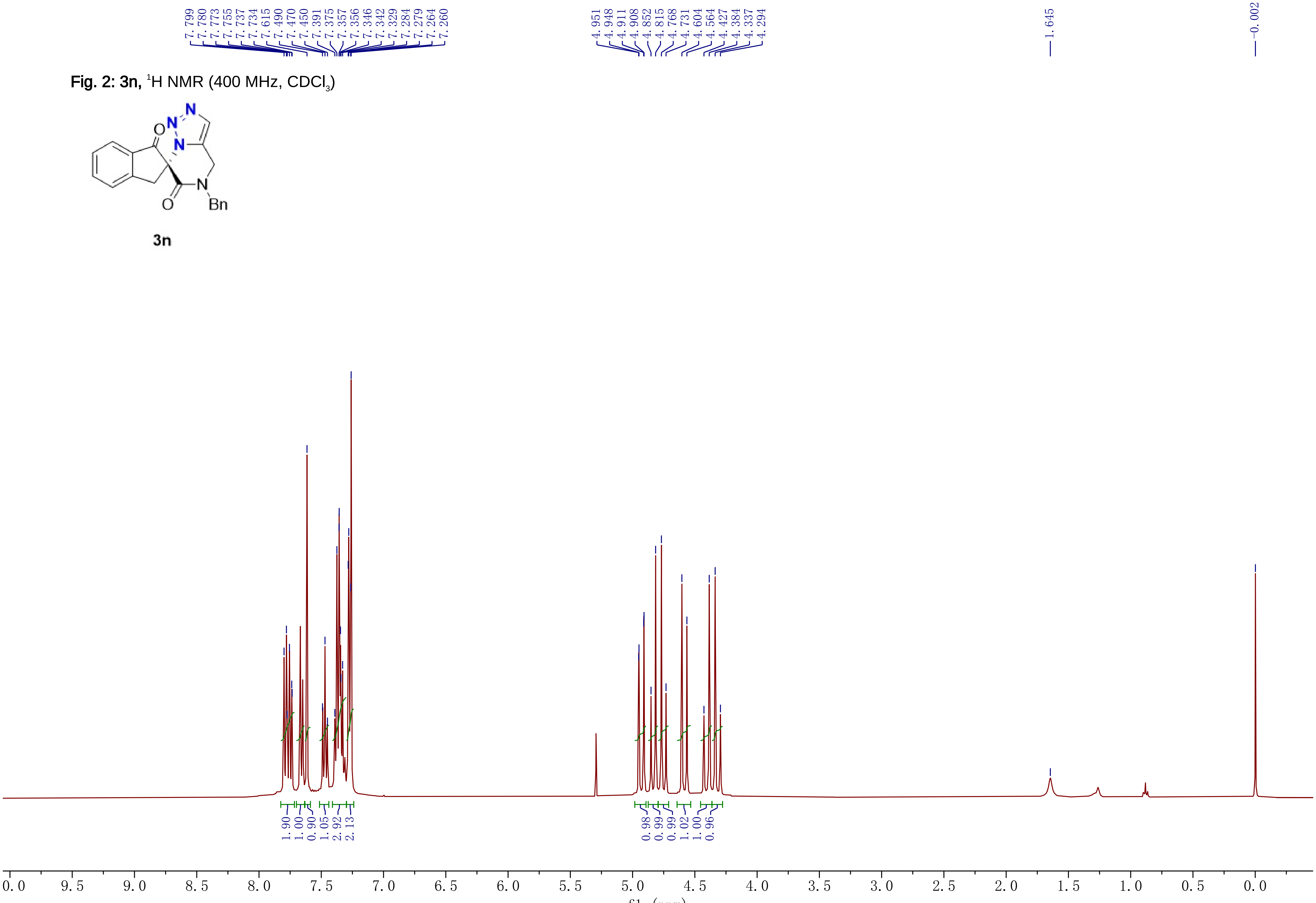

Fig. 2: 3n, ^{1}H NMR (400 MHz, $CDCl_3$)

3n

Fig. 2: **3n**, ^{13}C NMR (101 MHz, $CDCl_3$)

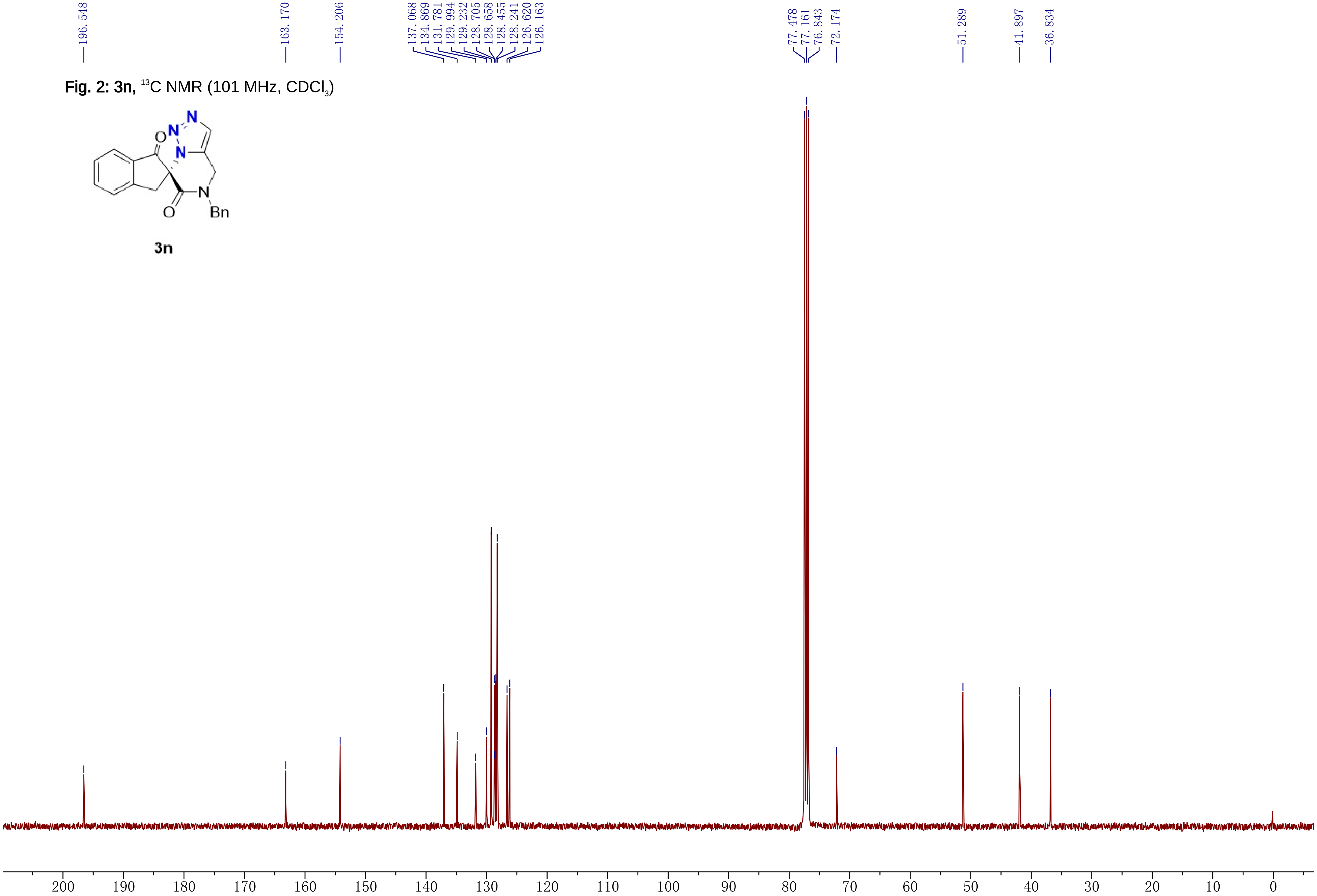

Fig. 2: 3o, ^{1}H NMR (400 MHz, $CDCl_3$)

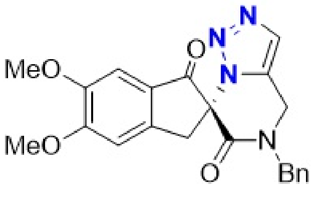

3o

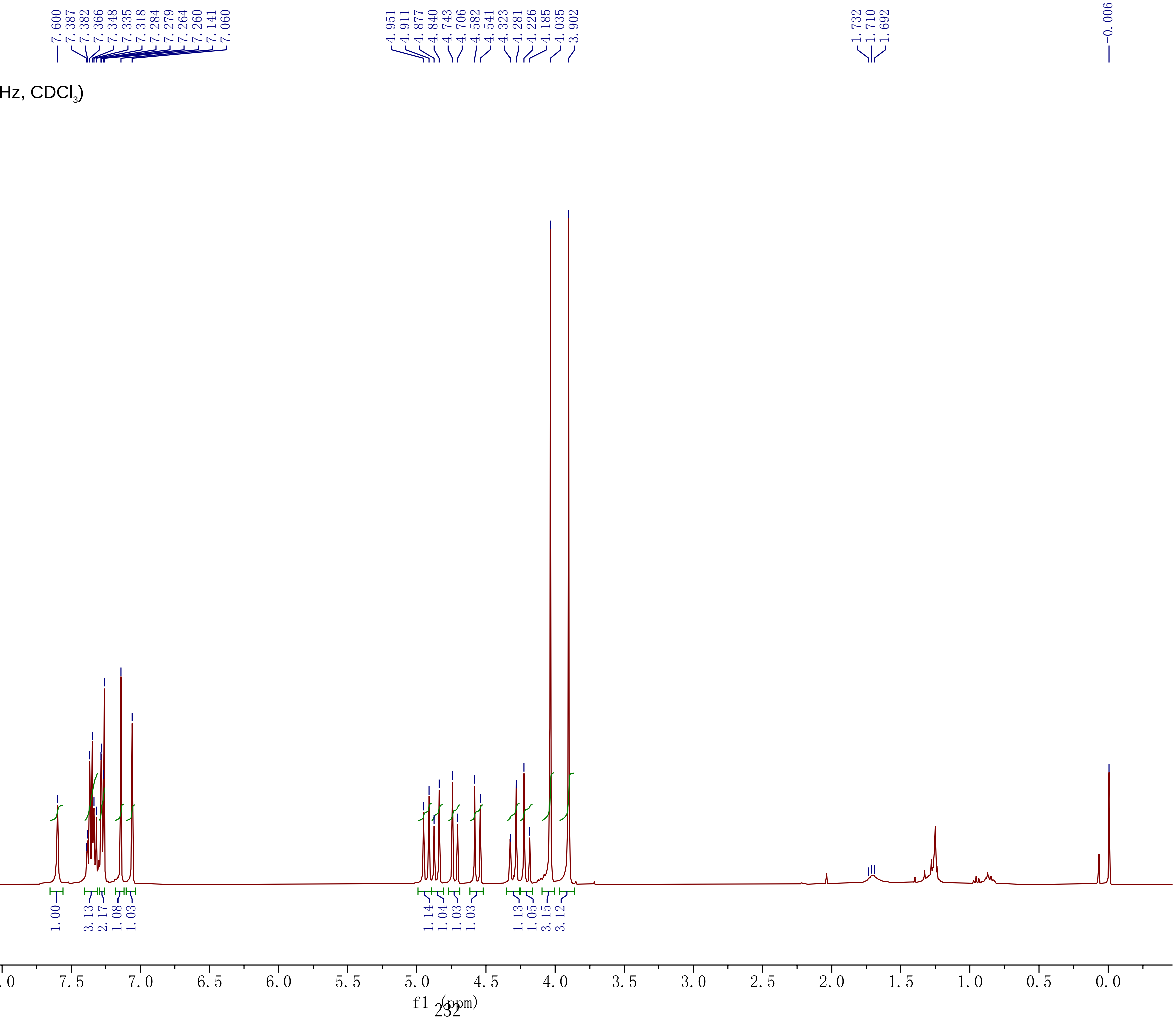

Fig. 2: 3o, ^{13}C NMR (101 MHz, $CDCl_3$)

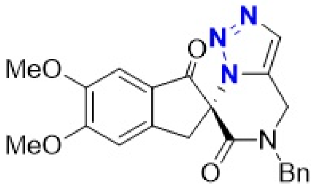

3o

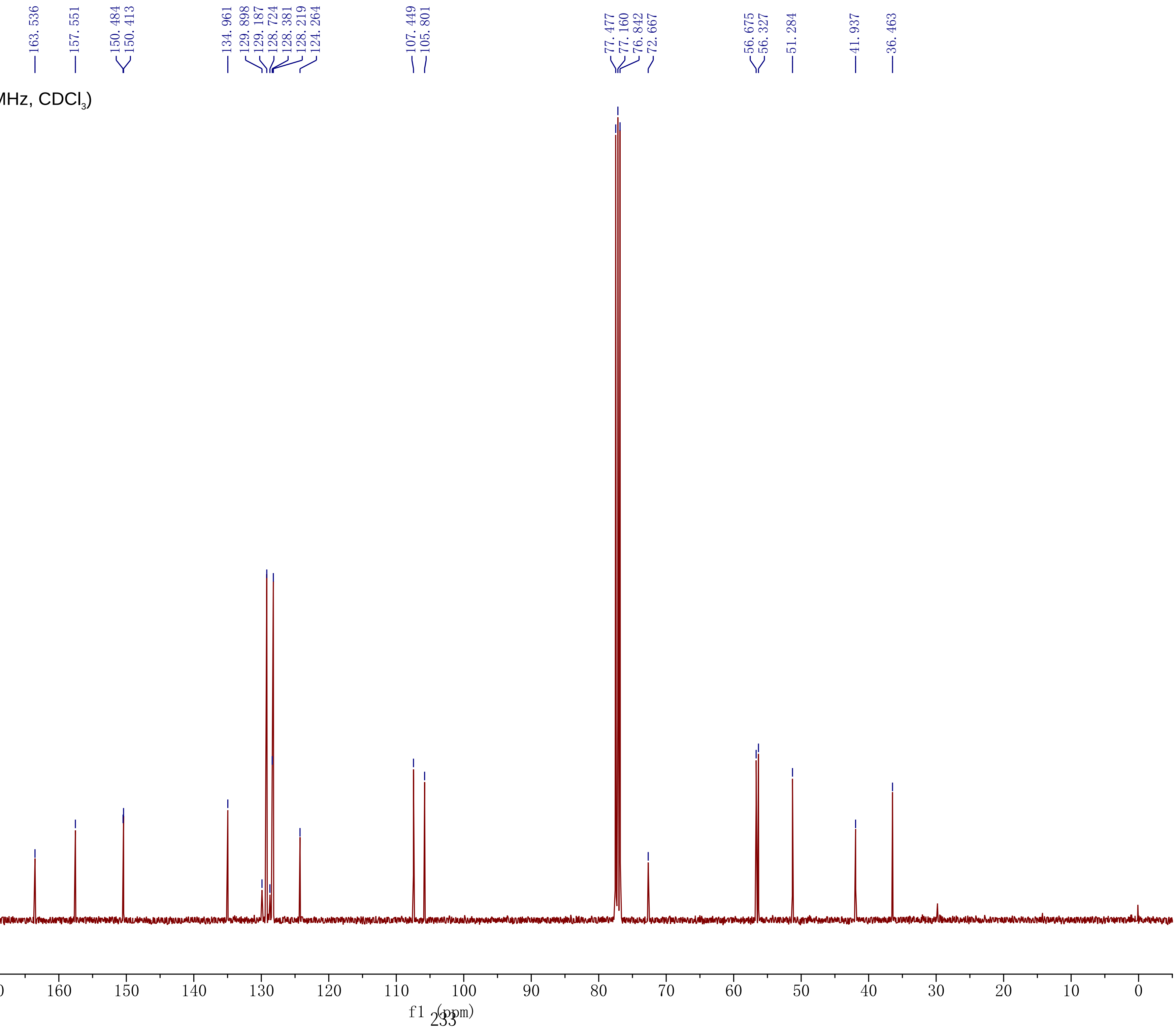

Fig. 2: 3p, ^{1}H NMR (400 MHz, $CDCl_3$)

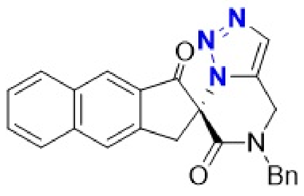

3p

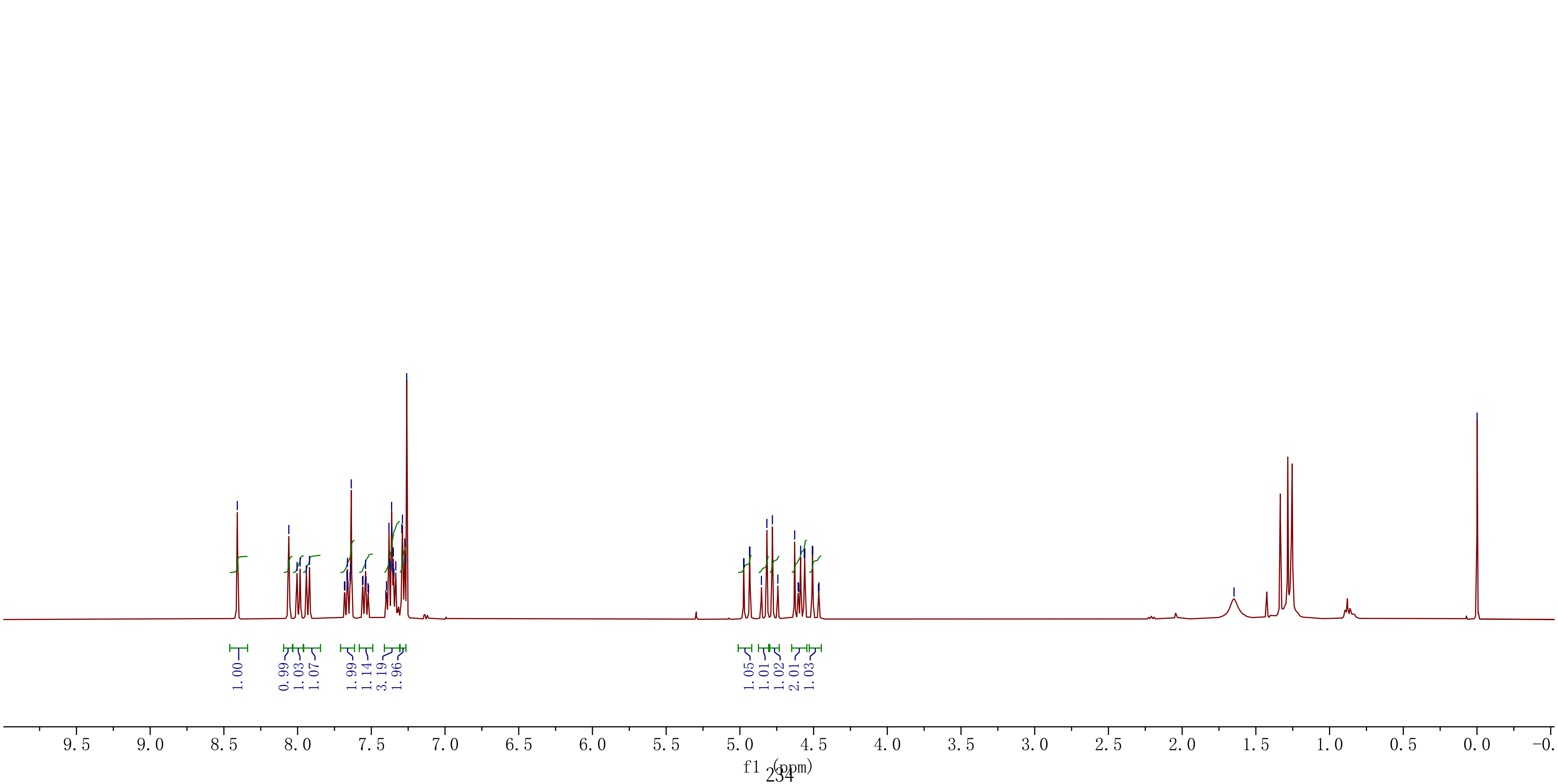

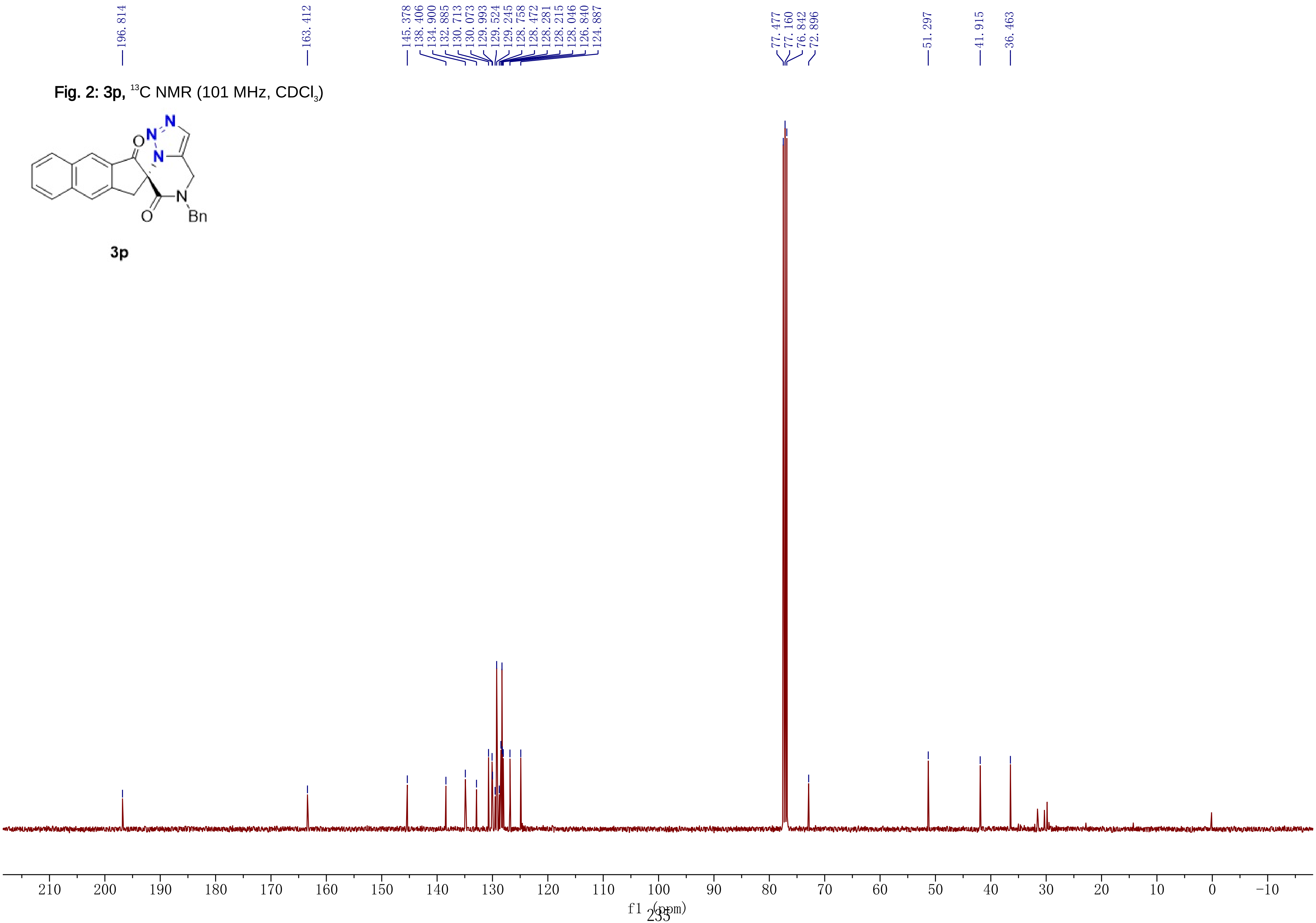
196.814
163.412
145.378
138.406
134.900
132.885
130.713
130.073
129.993
129.524
129.245
128.758
128.472
128.281
128.215
128.046
126.840
124.887
77.477
77.160
76.842
72.896
51.297
41.915
36.463
Fig. 2: 3p, ^{13}C NMR (101 MHz, CDCl$_3$)
O
N
N
N
N
O
Bn
3p
210 200 190 180 170 160 150 140 130 120 110 100 90 80 70 60 50 40 30 20 10 0 −10
f1 (ppm)

Fig. 2: **3q,** ^{1}H NMR (400 MHz, $CDCl_3$)

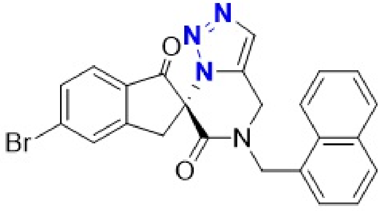

3q

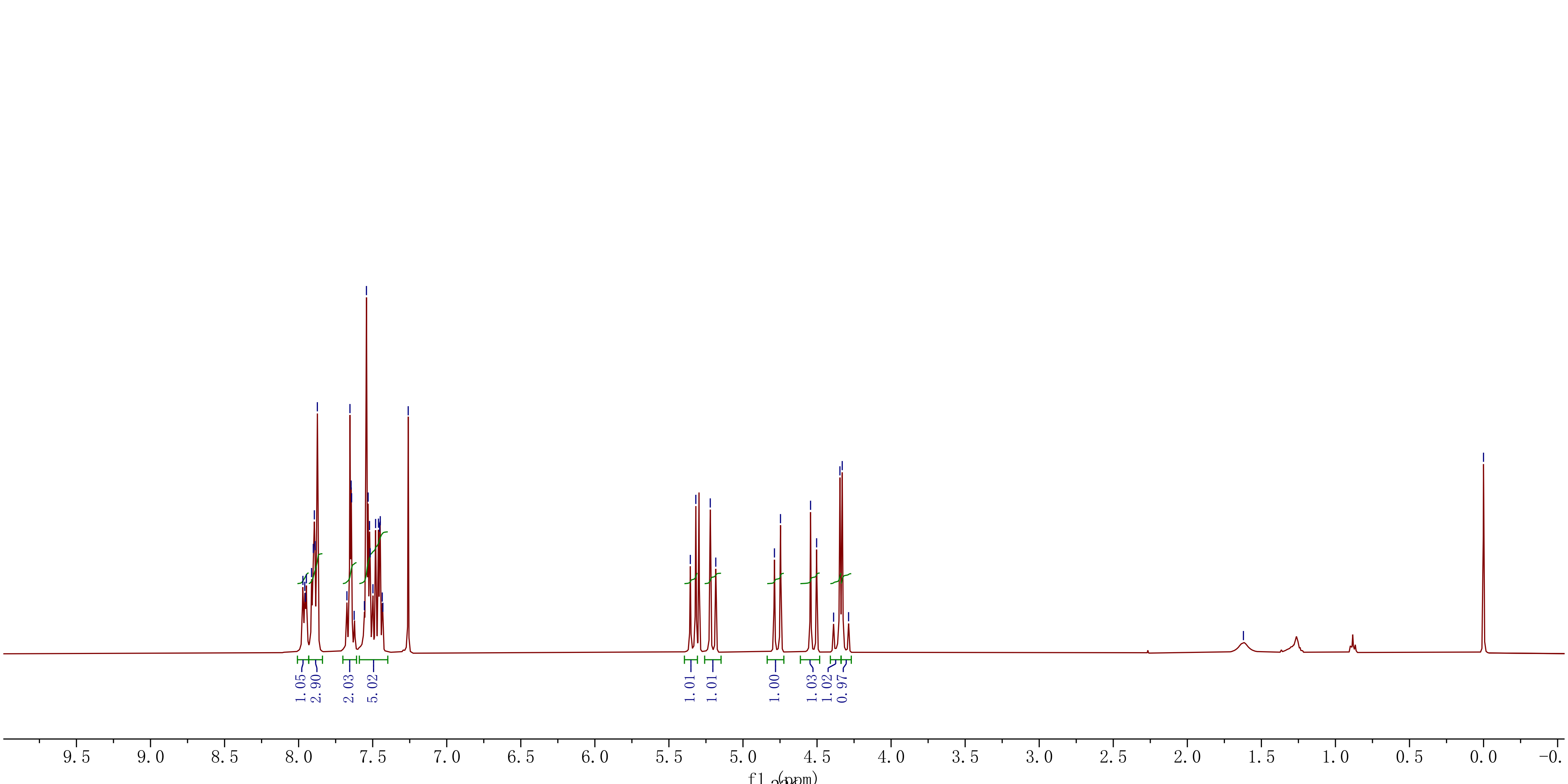

Fig. 2: 3q, ^{13}C NMR (101 MHz, $CDCl_3$)

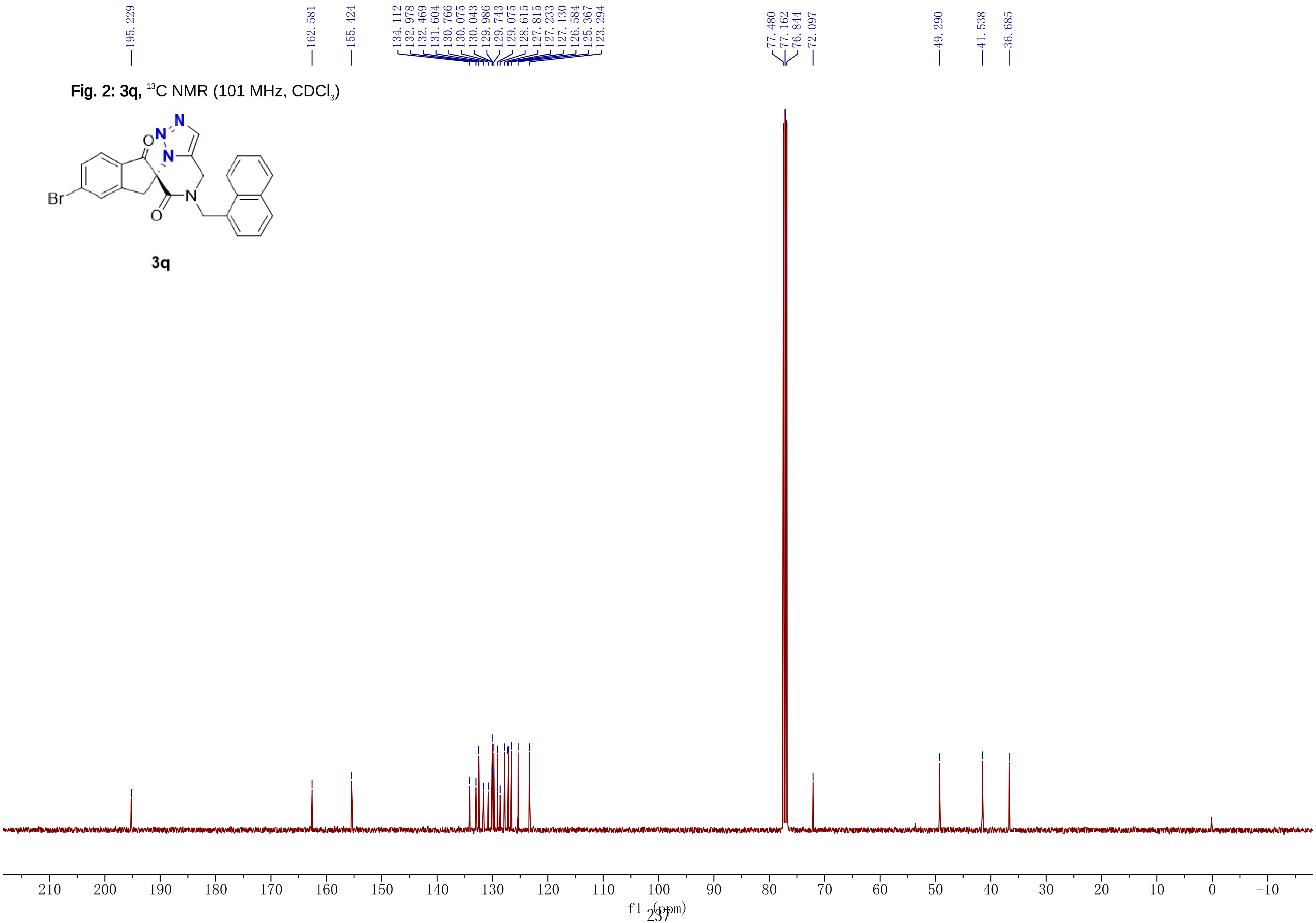

Fig. 2: 3r, ^{1}H NMR (400 MHz, CDCl$_3$)

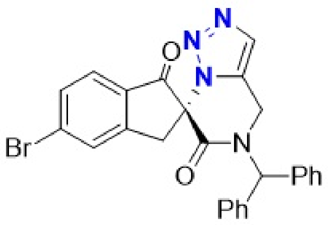

3r

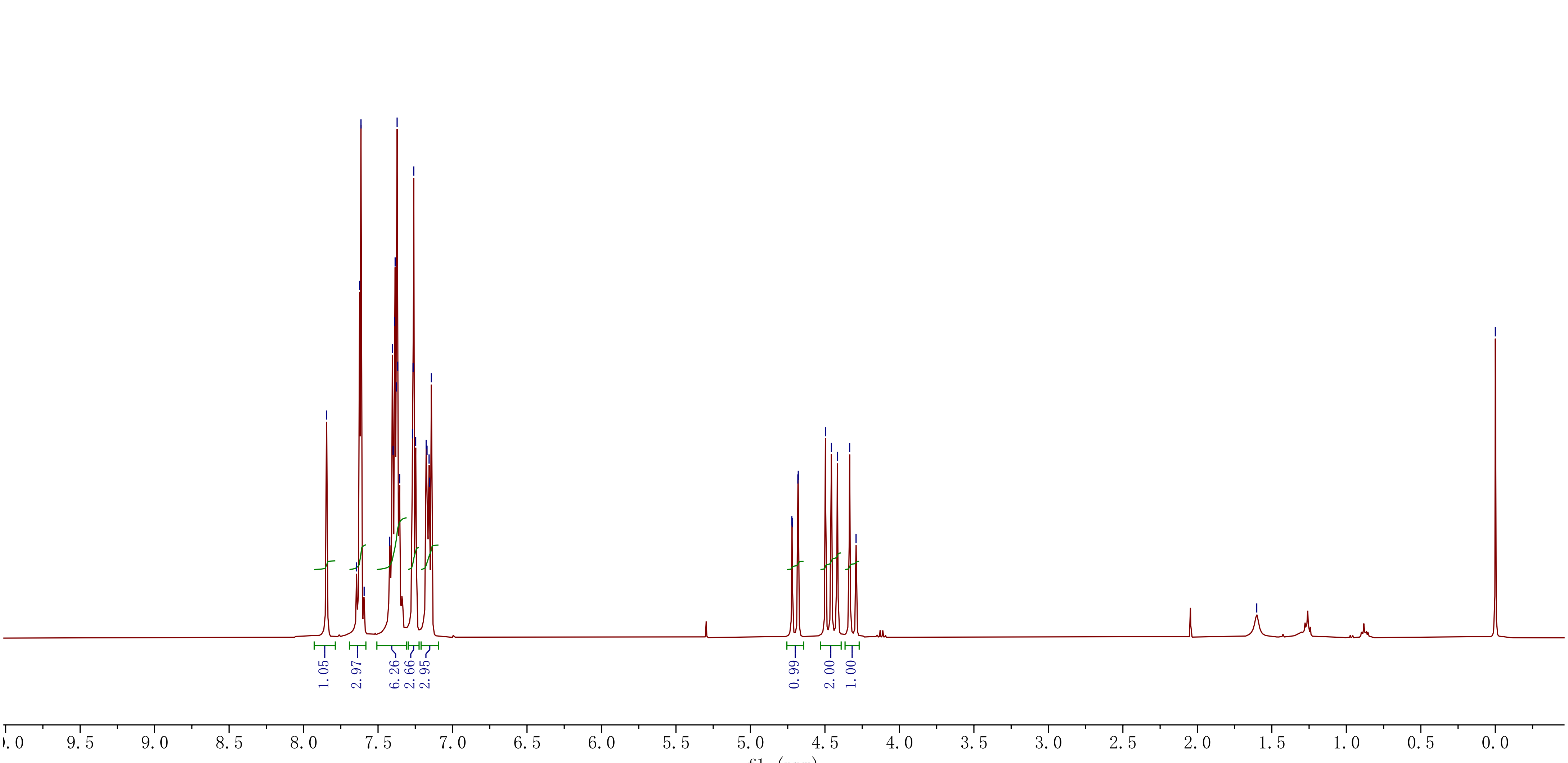

Fig. 2: 3r, ^{13}C NMR (101 MHz, $CDCl_3$)

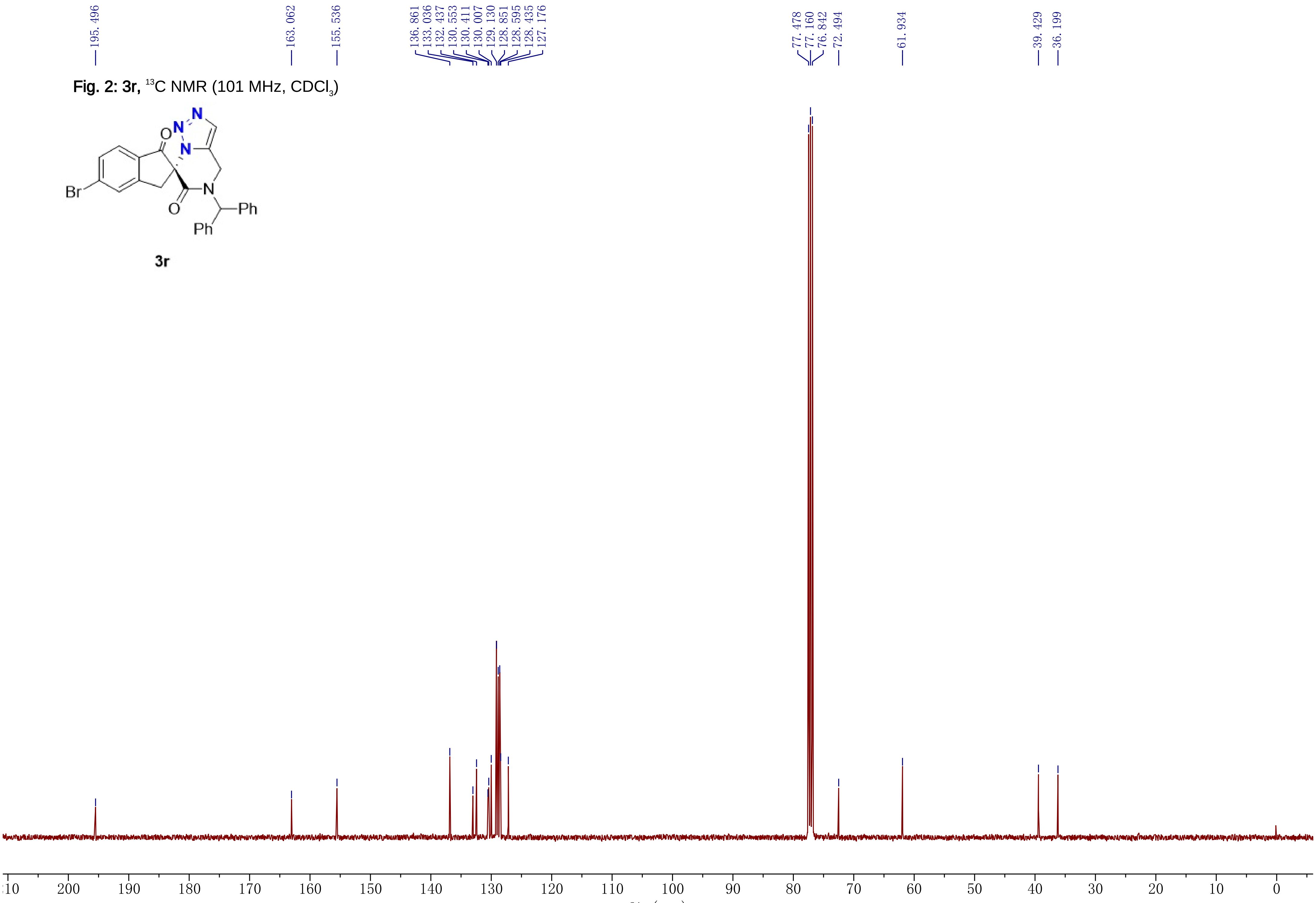

Fig. 2: 3s, ^{1}H NMR (400 MHz, $CDCl_3$)

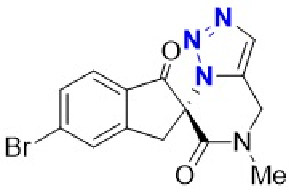

3s

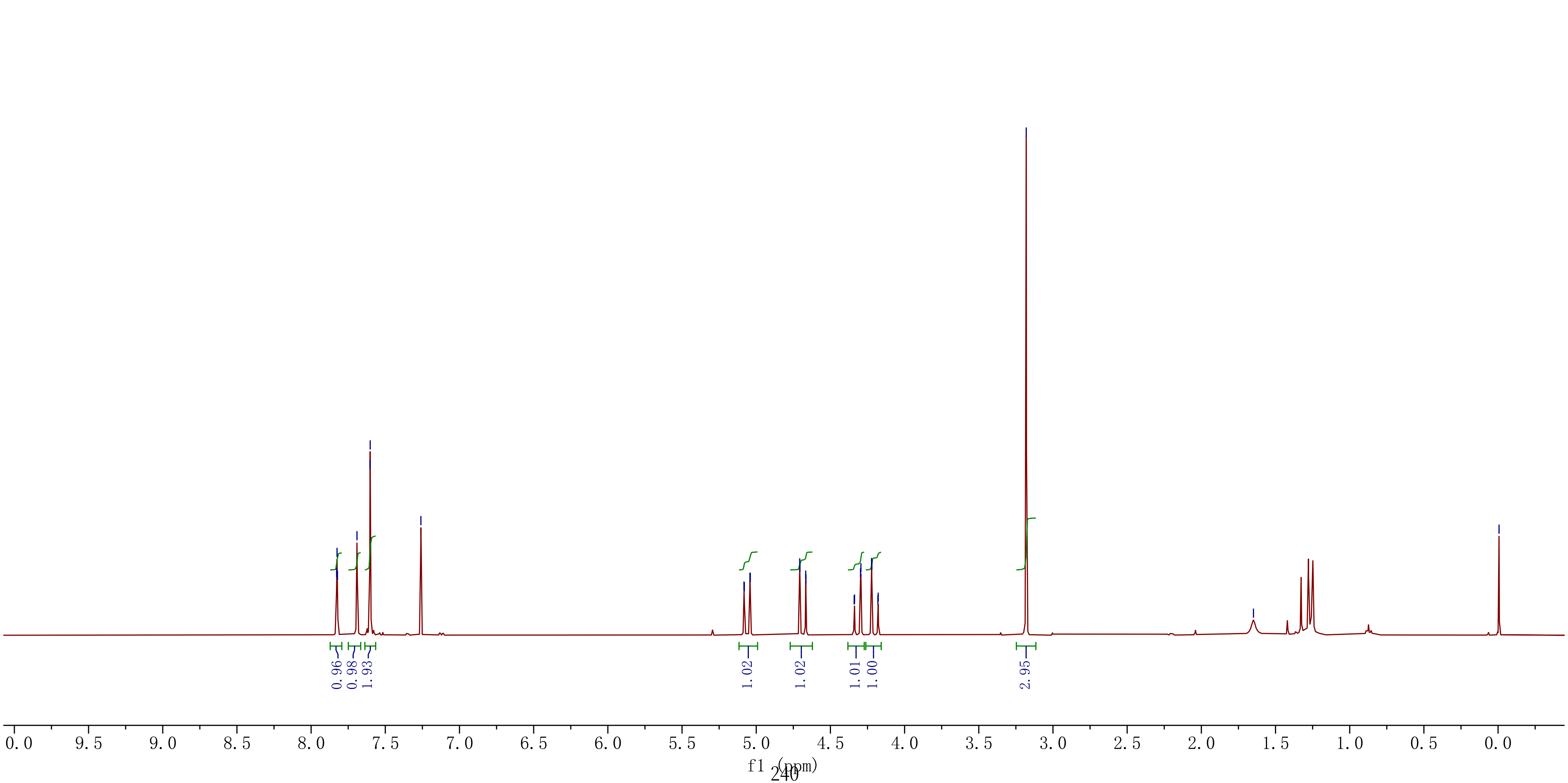

Fig. 2: 3s, ^{13}C NMR (101 MHz, $CDCl_3$)

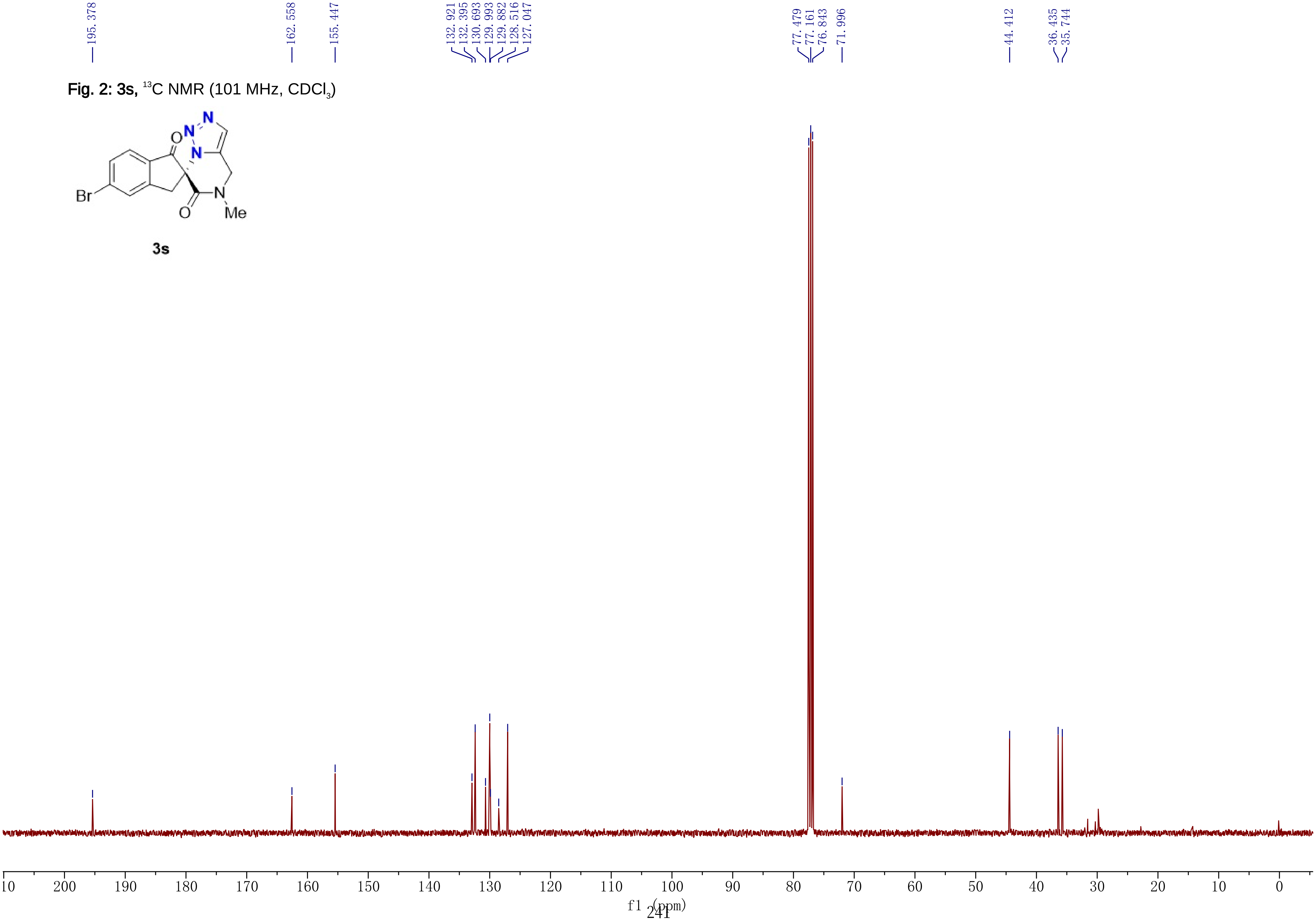

Fig. 2: 3t, ^{1}H NMR (400 MHz, $CDCl_3$)

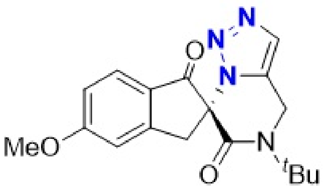

3t

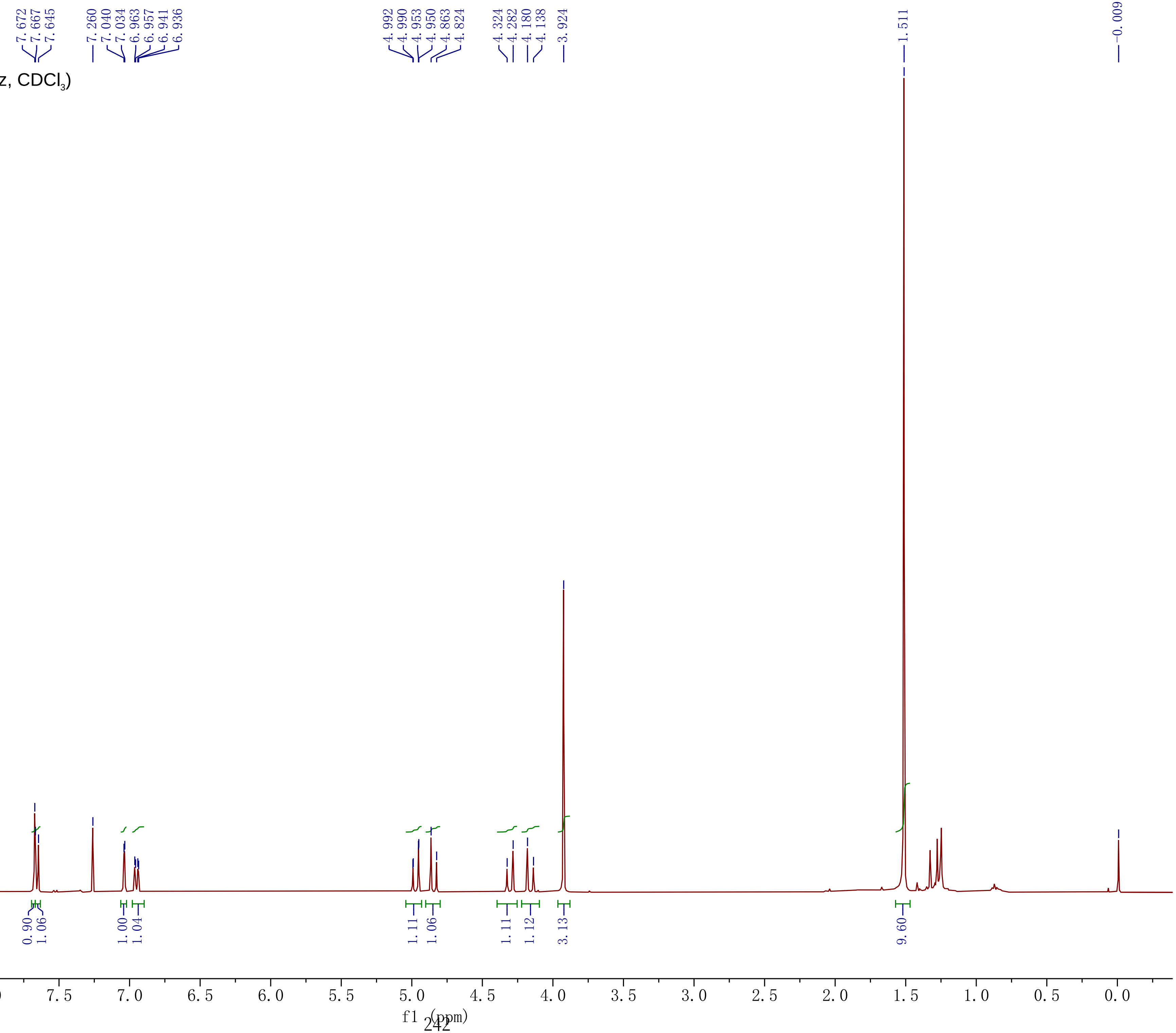

Fig. 2: 3t, ^{13}C NMR (101 MHz, $CDCl_3$)

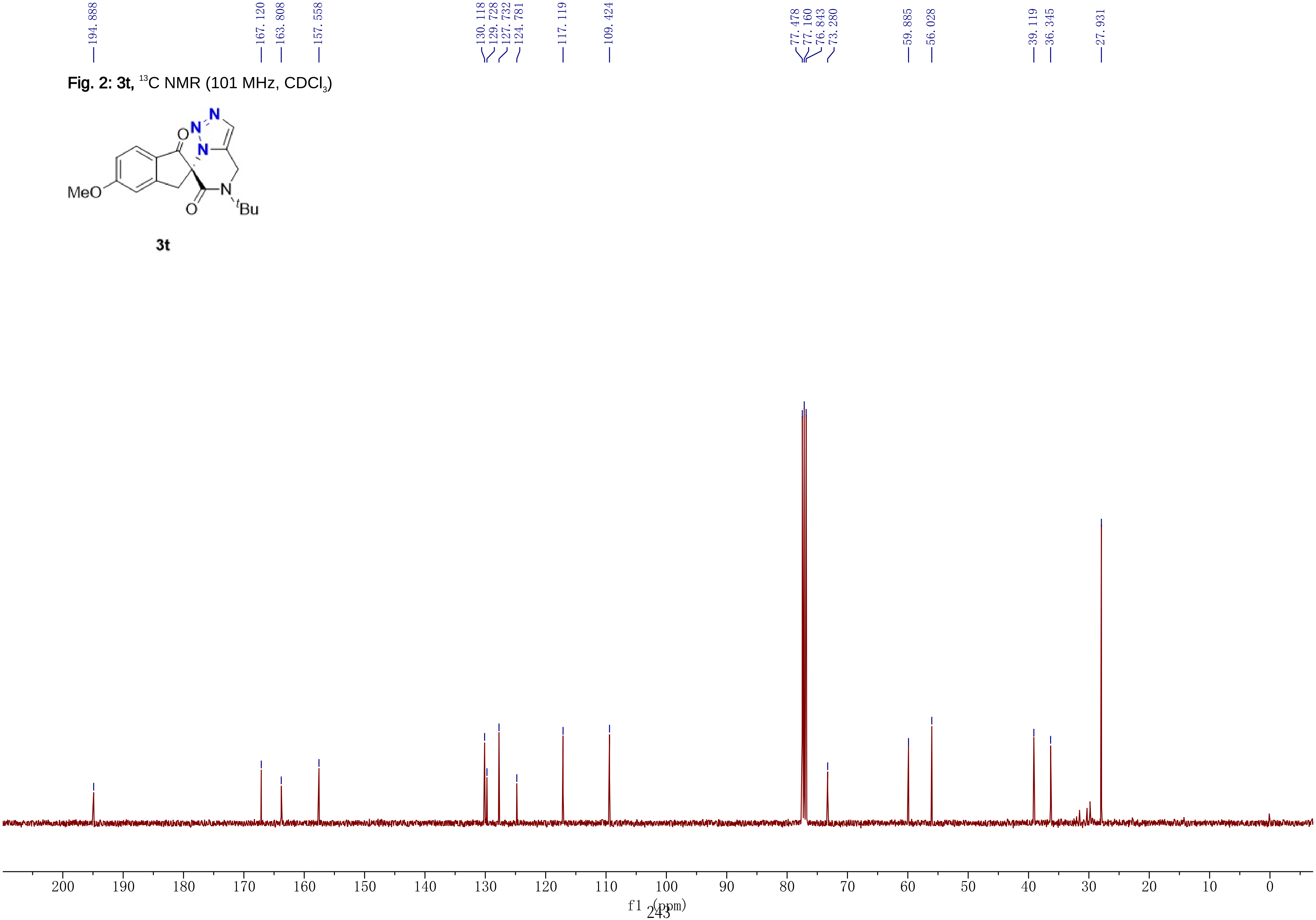

Fig. 2: 3u, ^{1}H NMR (400 MHz, $CDCl_3$)

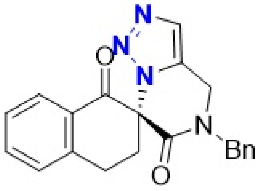

3u

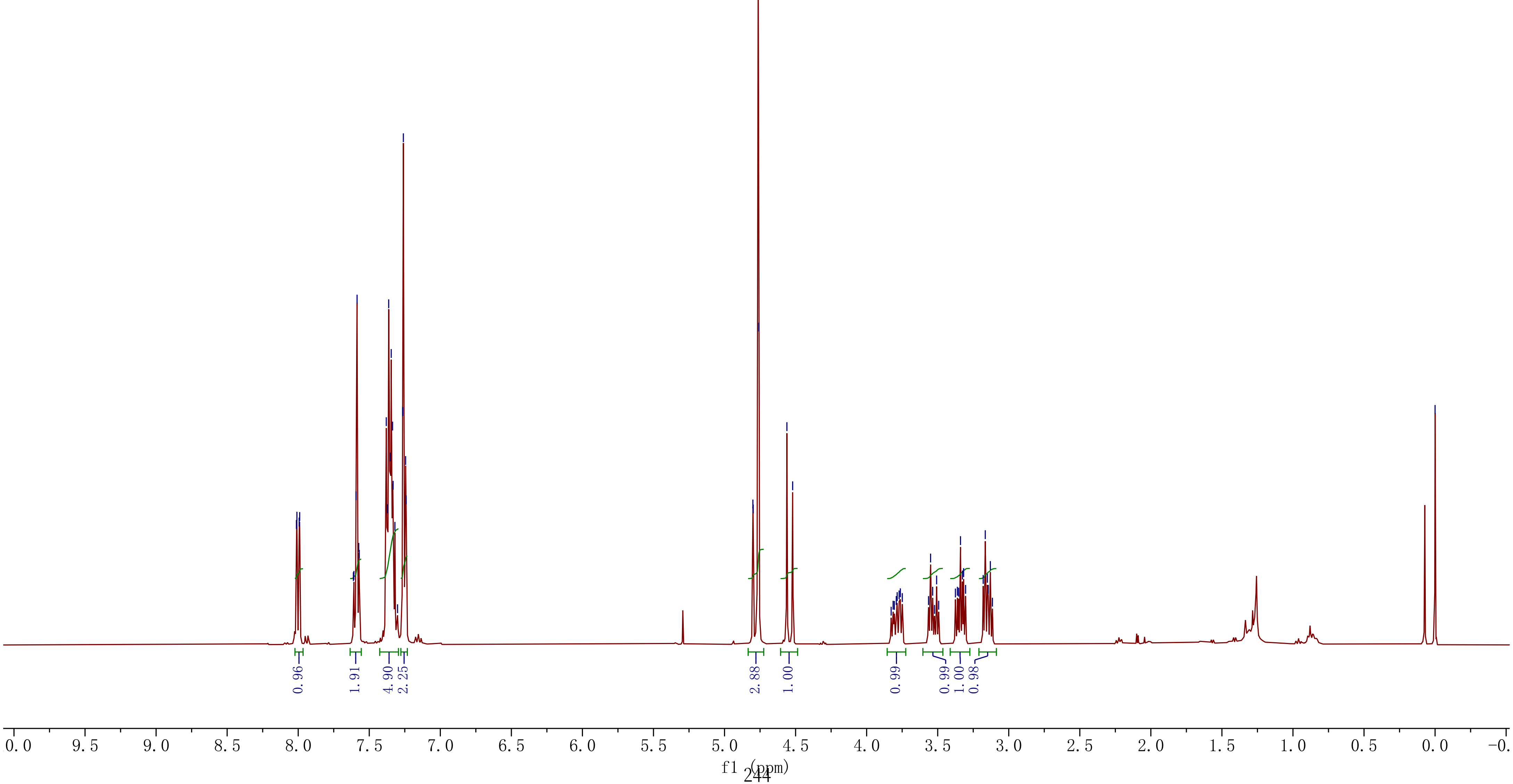

Fig. 2: 3u, ^{13}C NMR (101 MHz, $CDCl_3$)

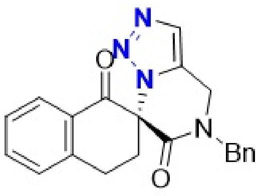

3u

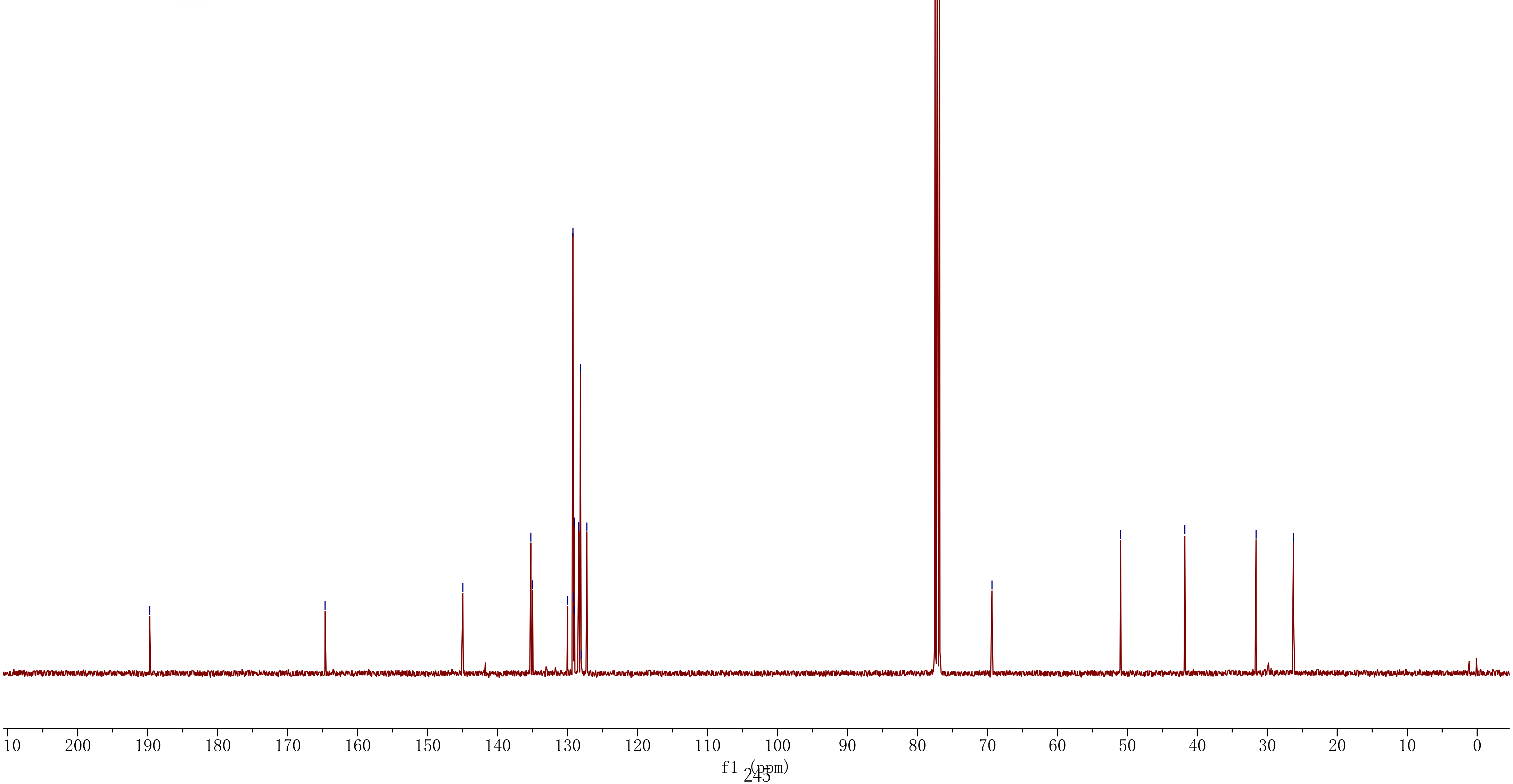

Fig. 2: 3v, ^{1}H NMR (400 MHz, $CDCl_3$)

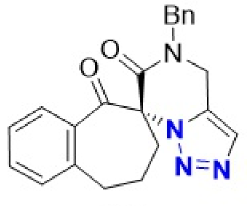

3v

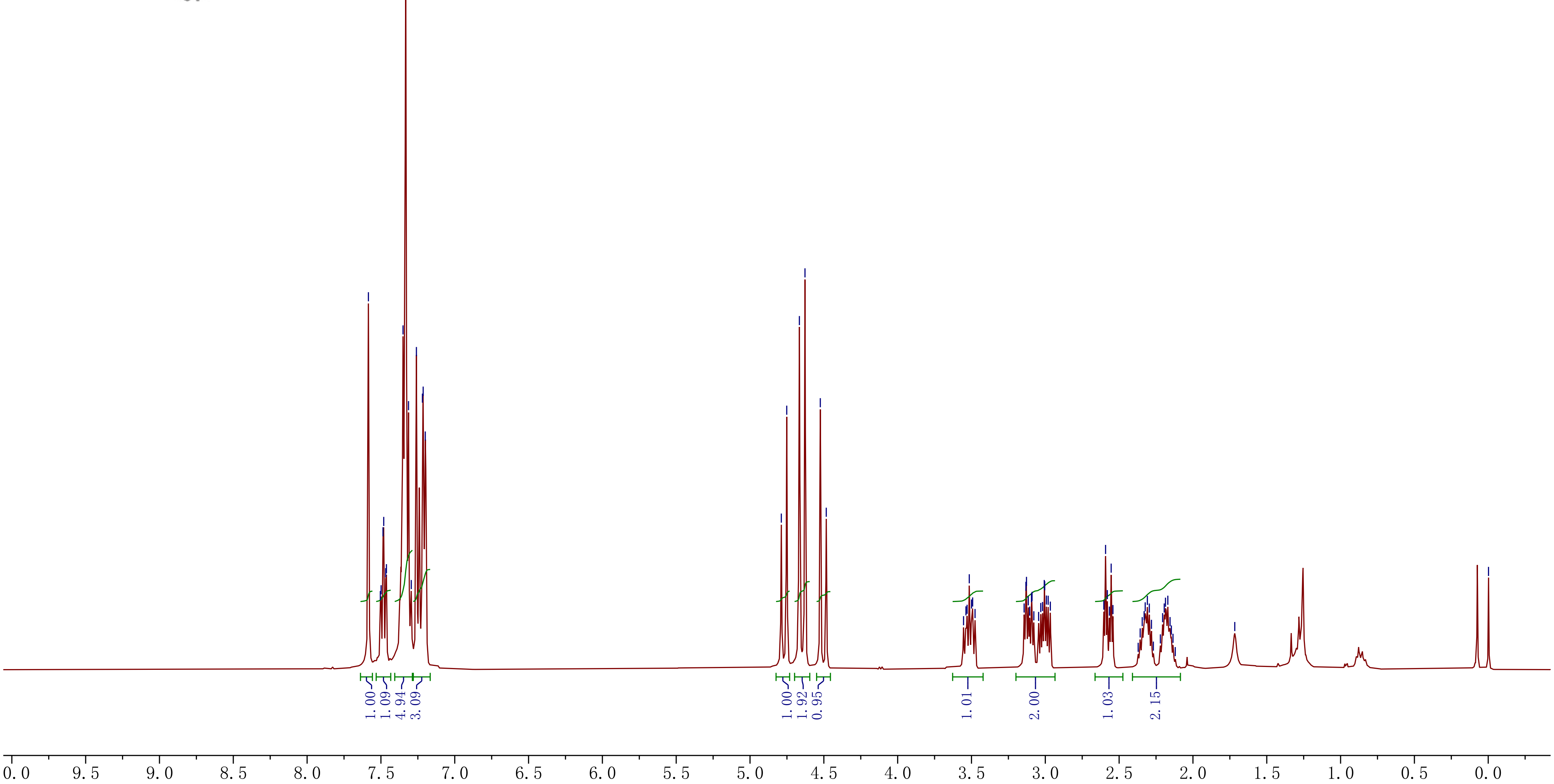

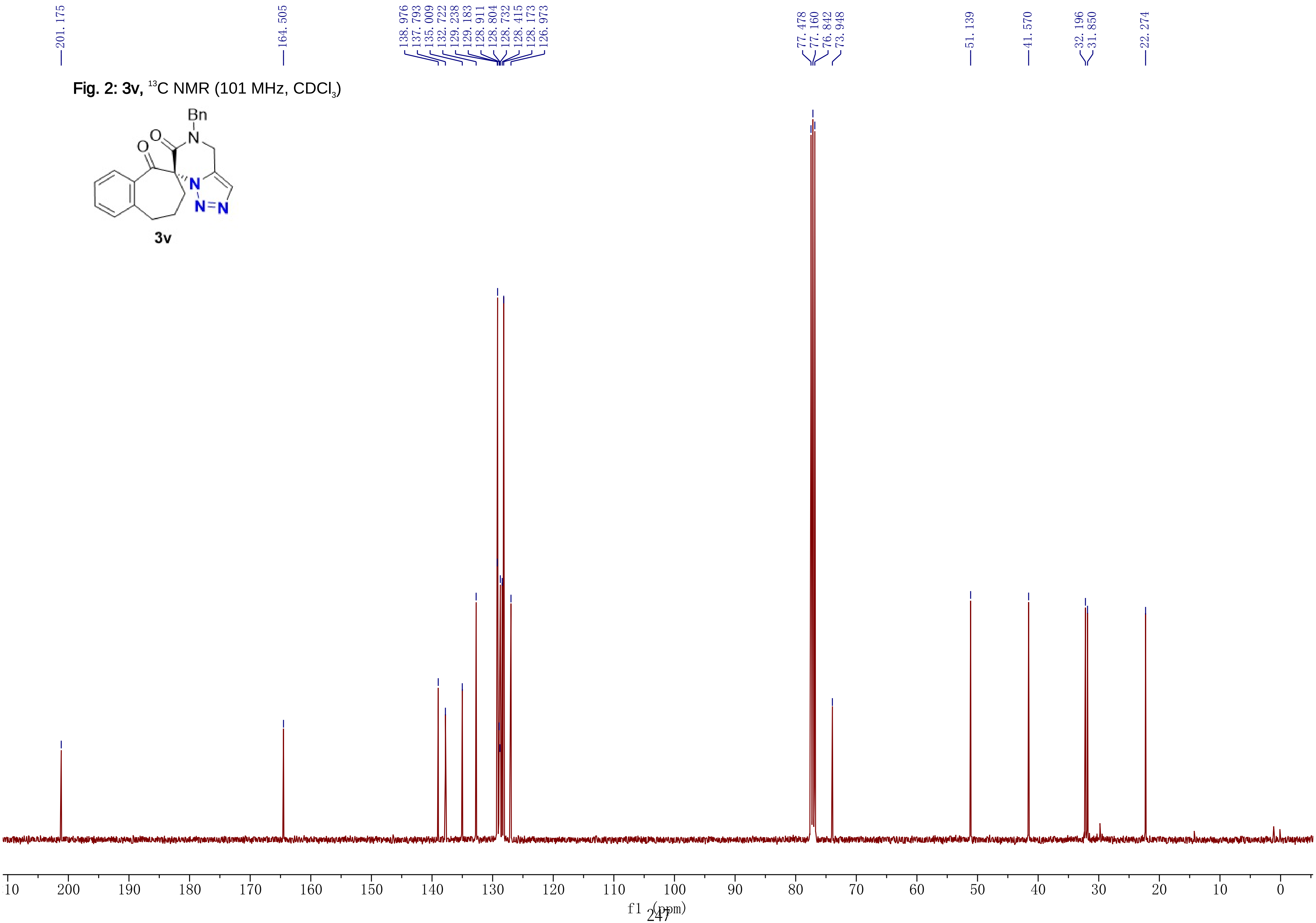

Fig. 2: 3v, ^{13}C NMR (101 MHz, $CDCl_3$)

Fig. 2: 3w', ^{1}H NMR (400 MHz, $CDCl_3$)

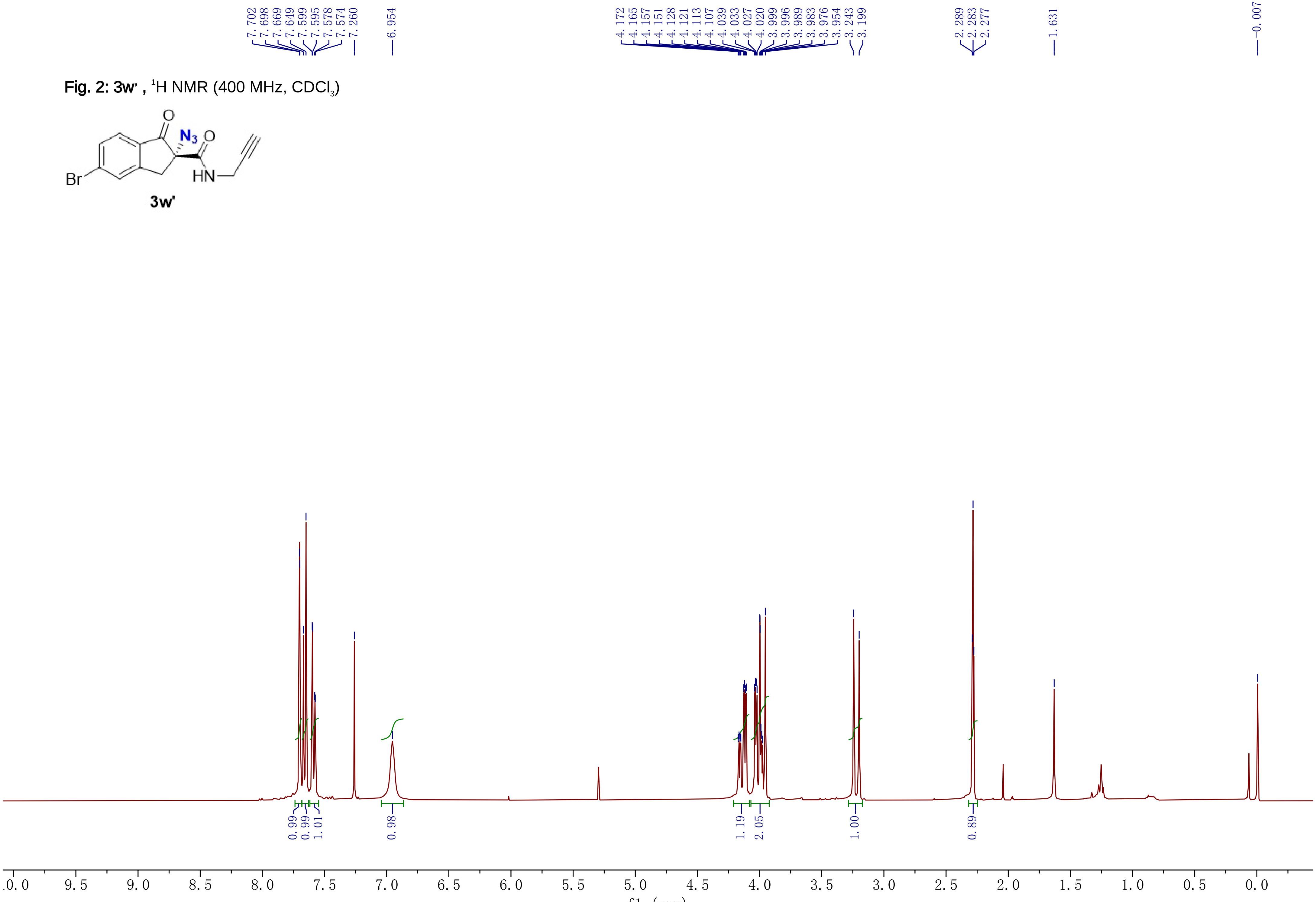

Fig. 2: 3w' , ^{13}C NMR (101 MHz, $CDCl_3$)

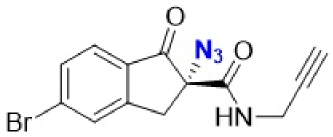

3w'

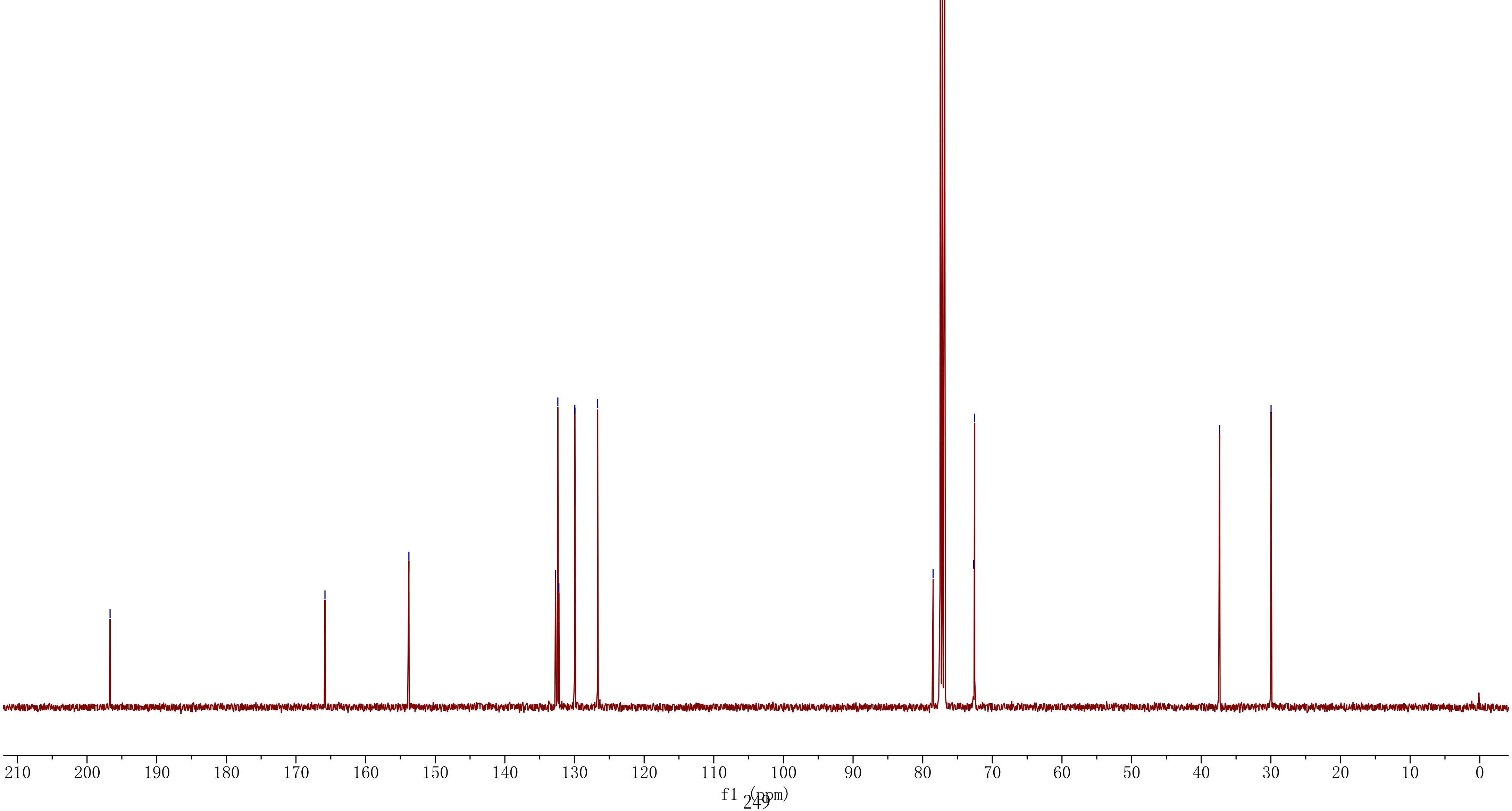

Fig. 2: 3x' , ^{1}H NMR (400 MHz, $CDCl_3$)

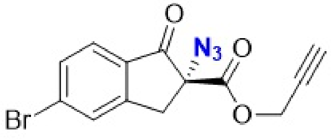

3x'

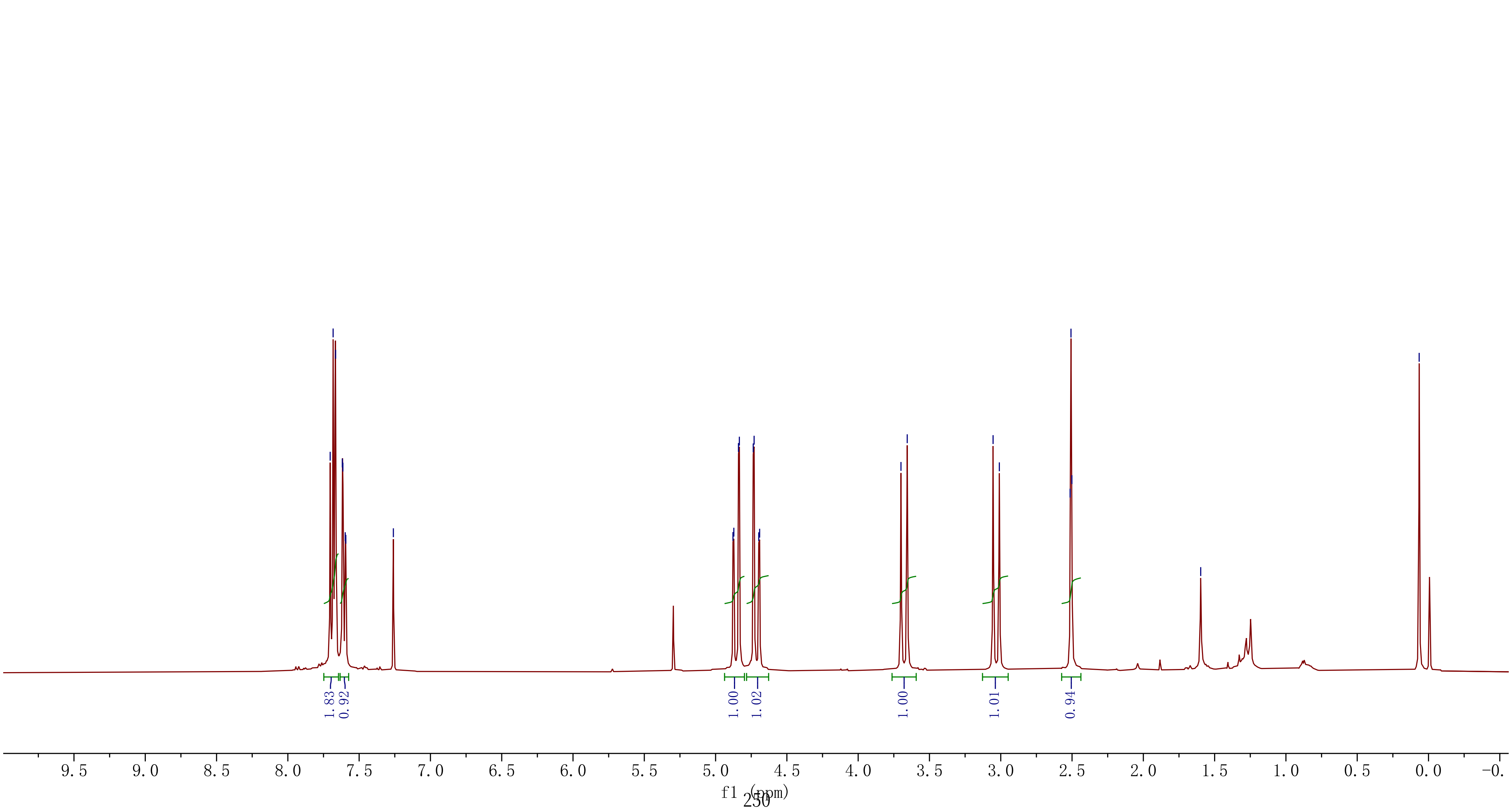

Fig. 2: 3x' , ^{13}C NMR (101 MHz, $CDCl_3$)

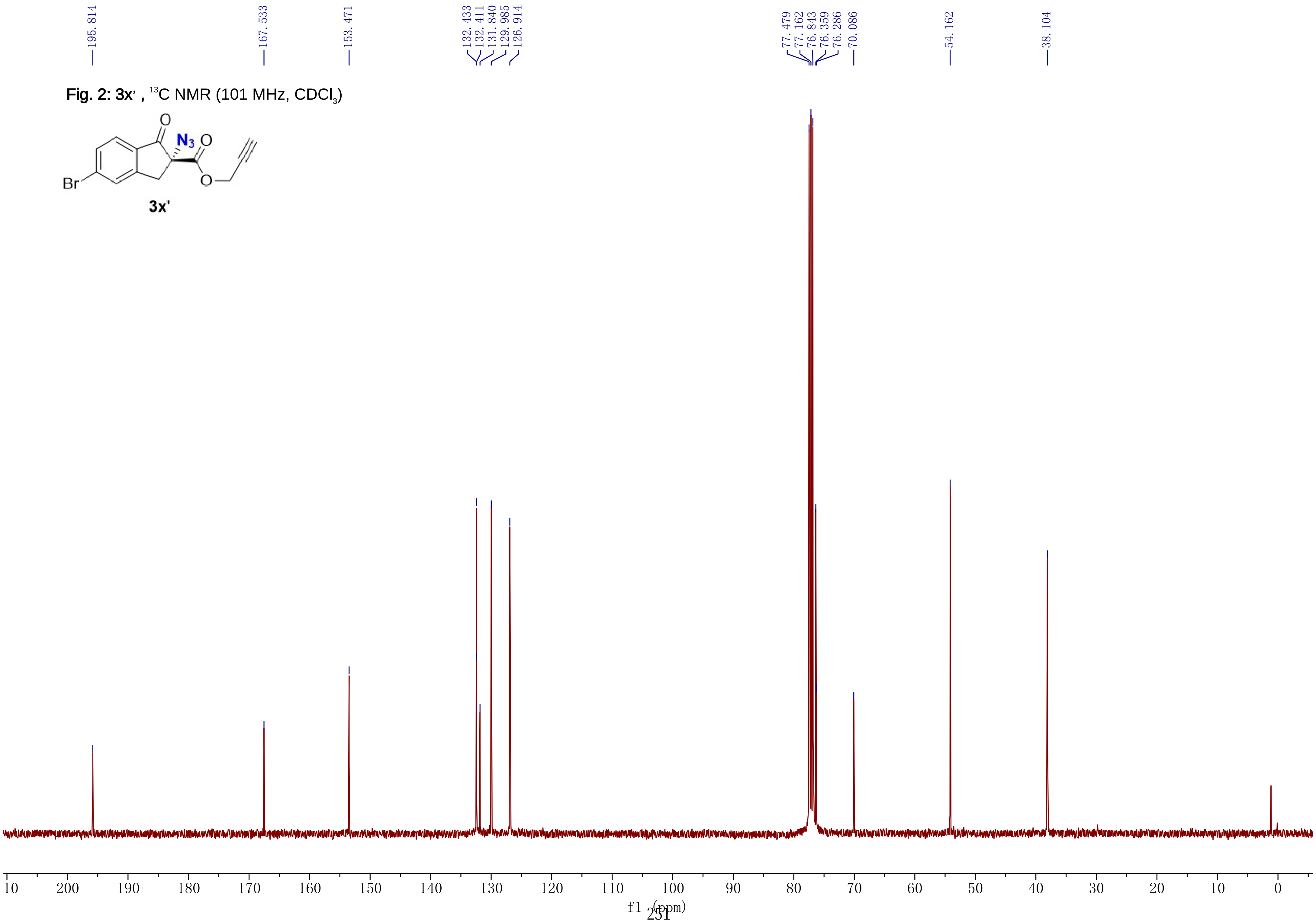

Fig. 3: 5a, ^{1}H NMR (400 MHz, $CDCl_3$)

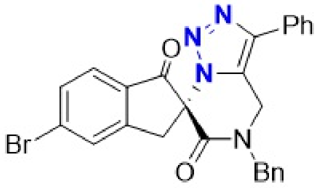

5a

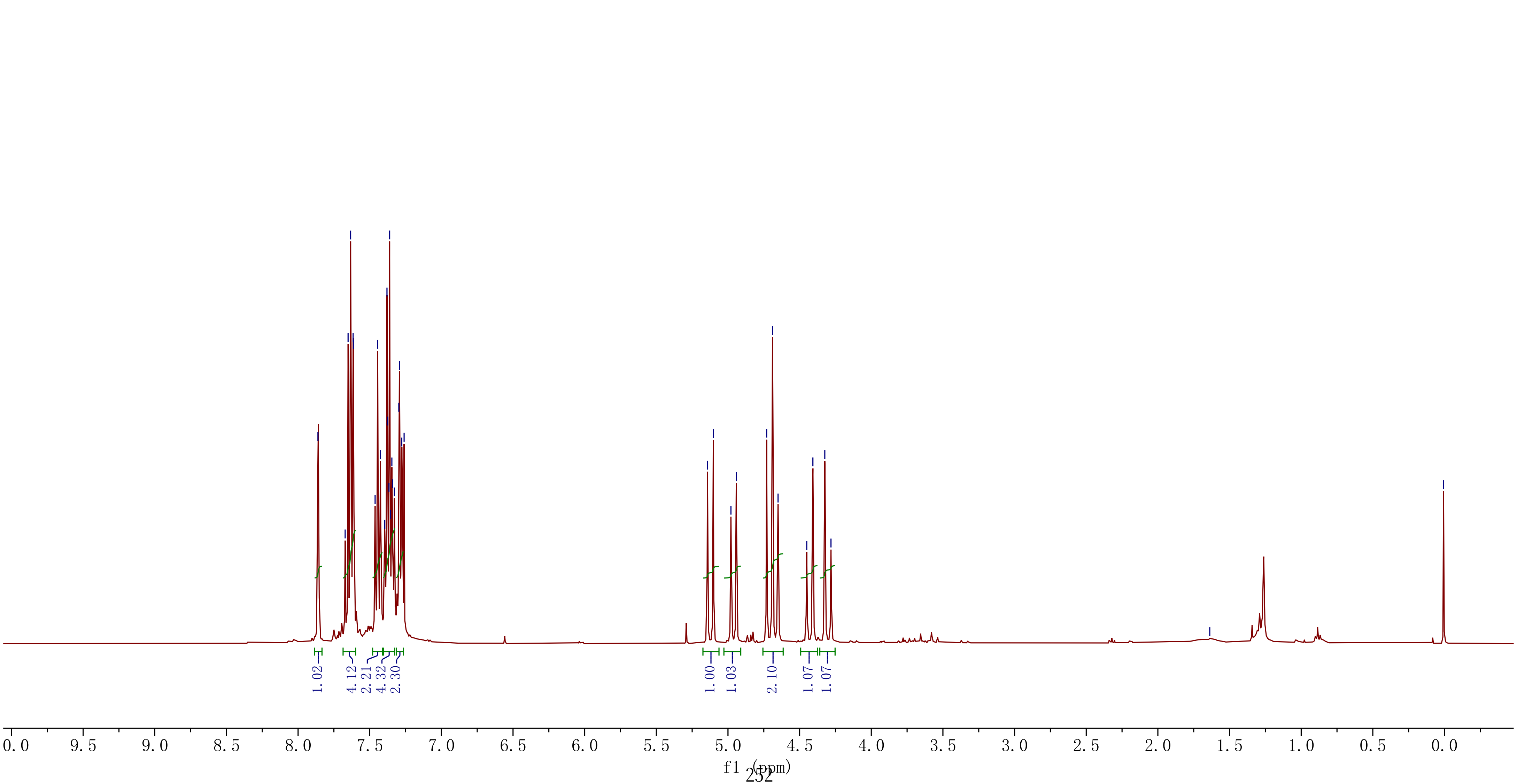

Fig. 3: 5a, ^{13}C NMR (101 MHz, $CDCl_3$)

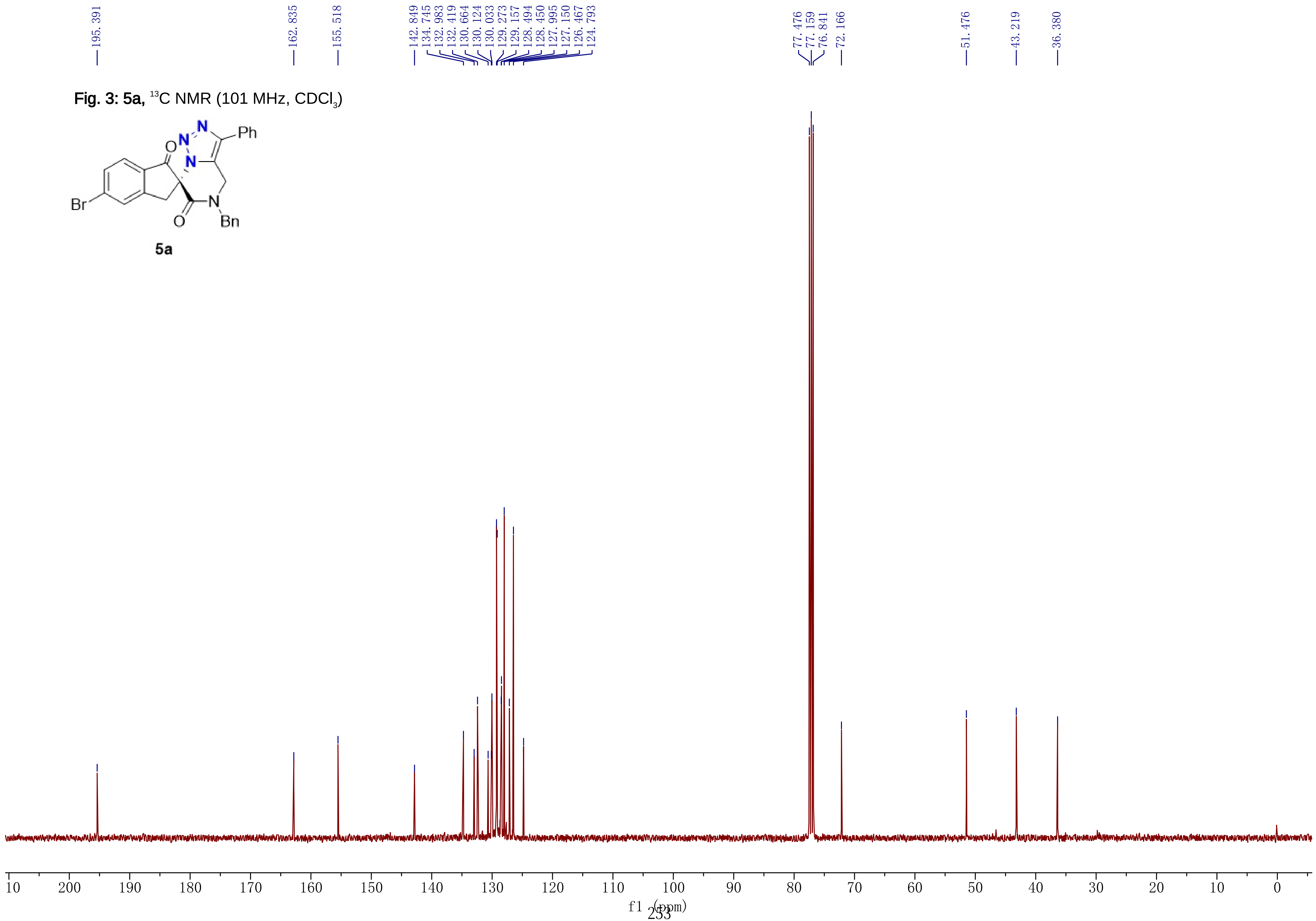

5a

Fig. 3: 5b, ^{1}H NMR (400 MHz, $CDCl_3$)

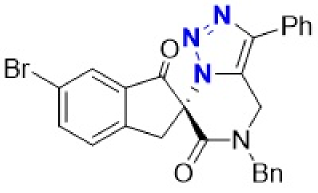

5b

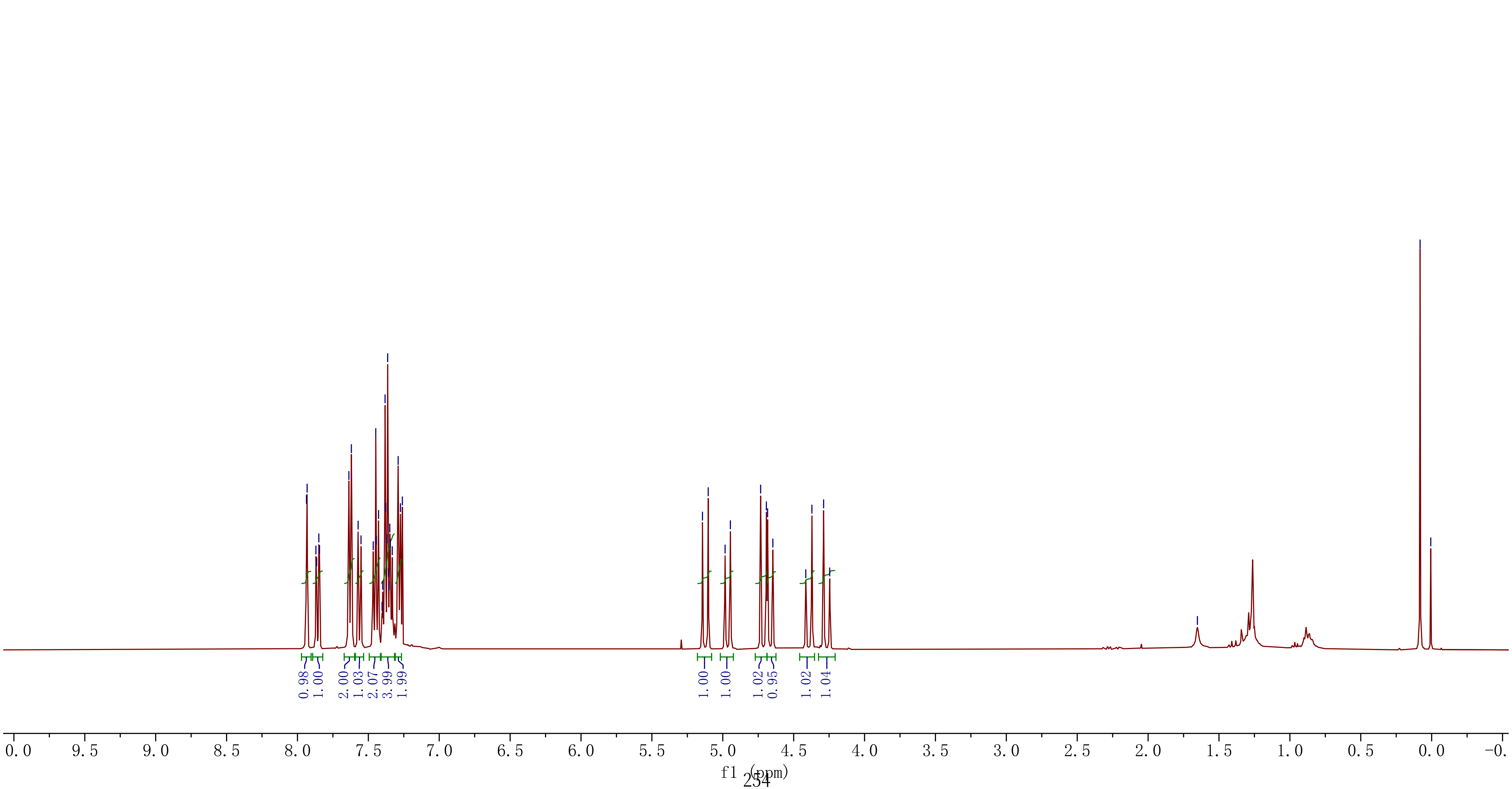

Fig. 3: 5b, ^{13}C NMR (101 MHz, $CDCl_3$)

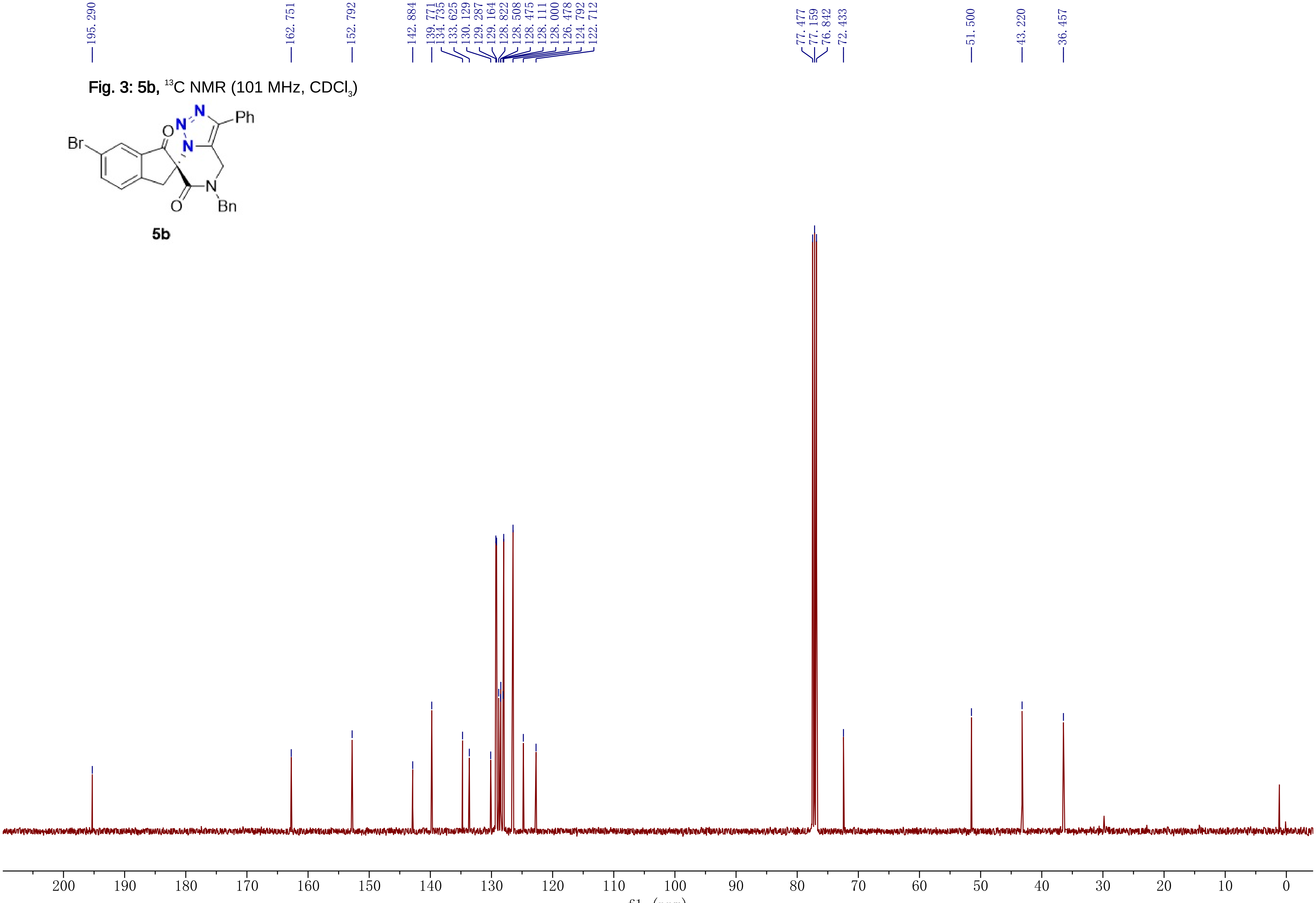

5b

Fig. 3: 5c, ^{1}H NMR (400 MHz, $CDCl_3$)

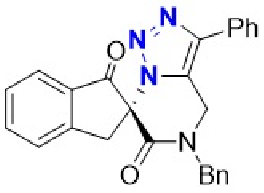

5c

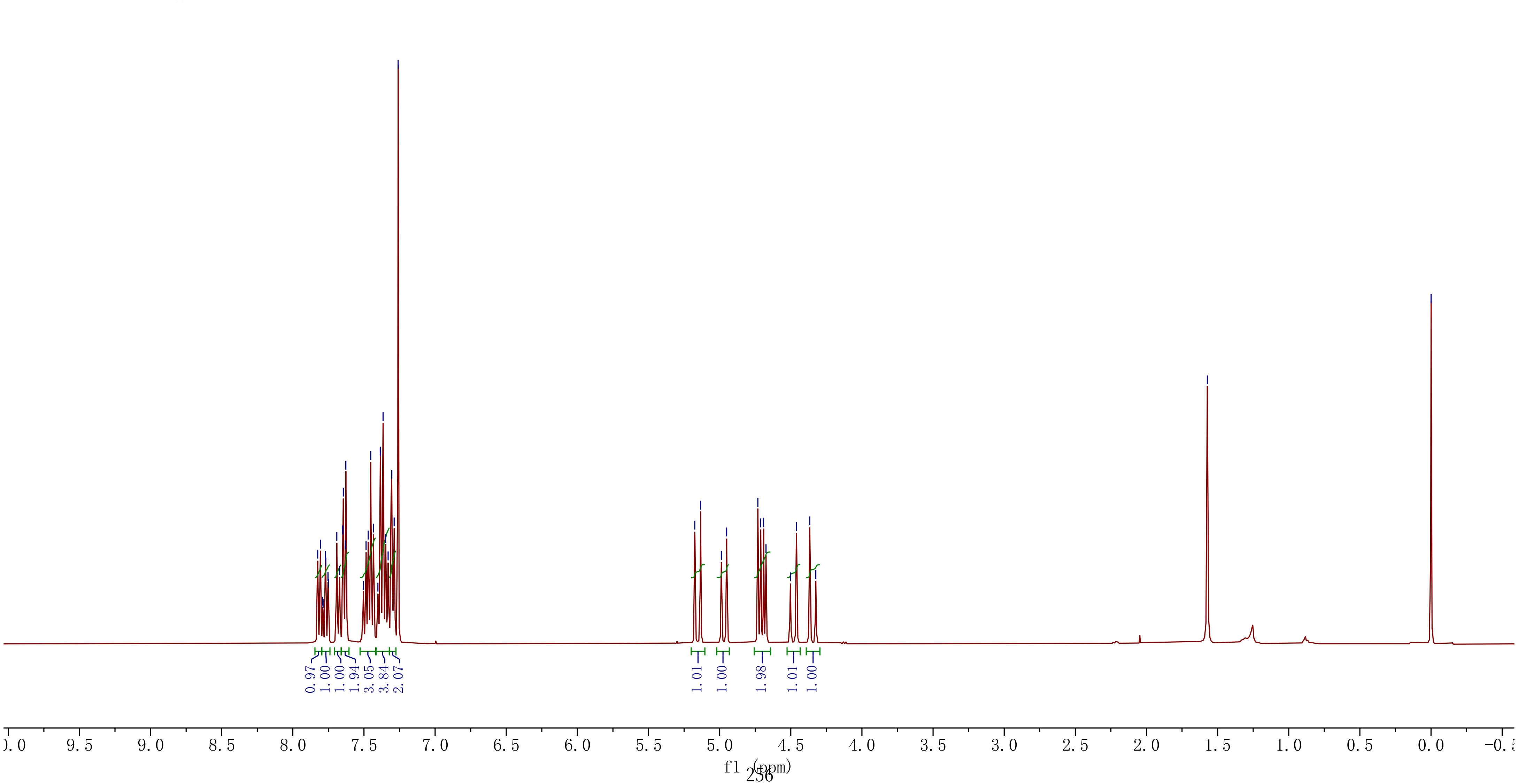

Fig. 3: 5c, ^{13}C NMR (101 MHz, $CDCl_3$)

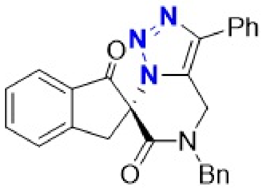

5c

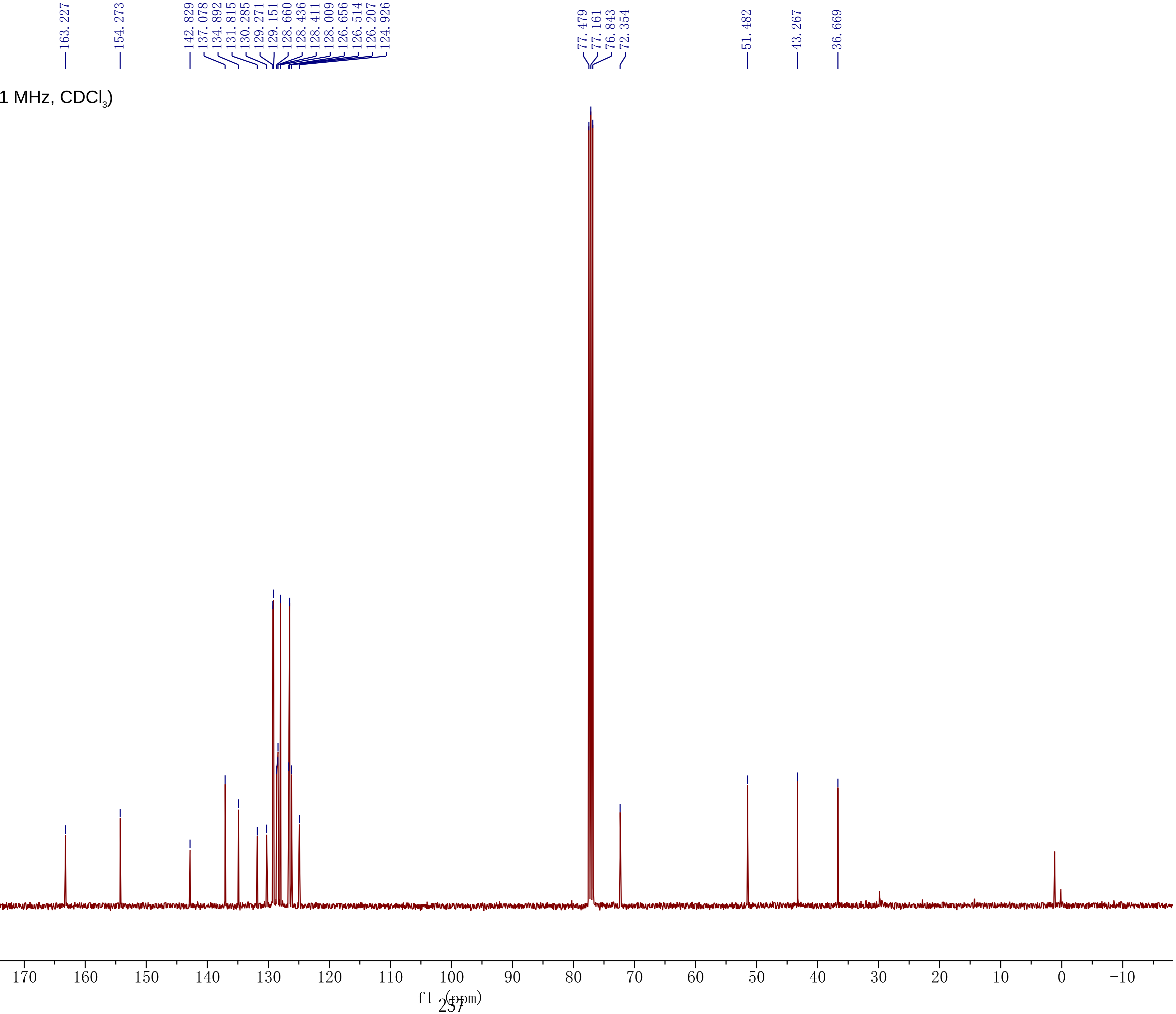

Fig. 3: 5d, ^1H NMR (400 MHz, $CDCl_3$)

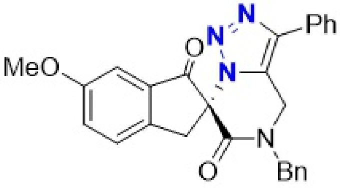

5d

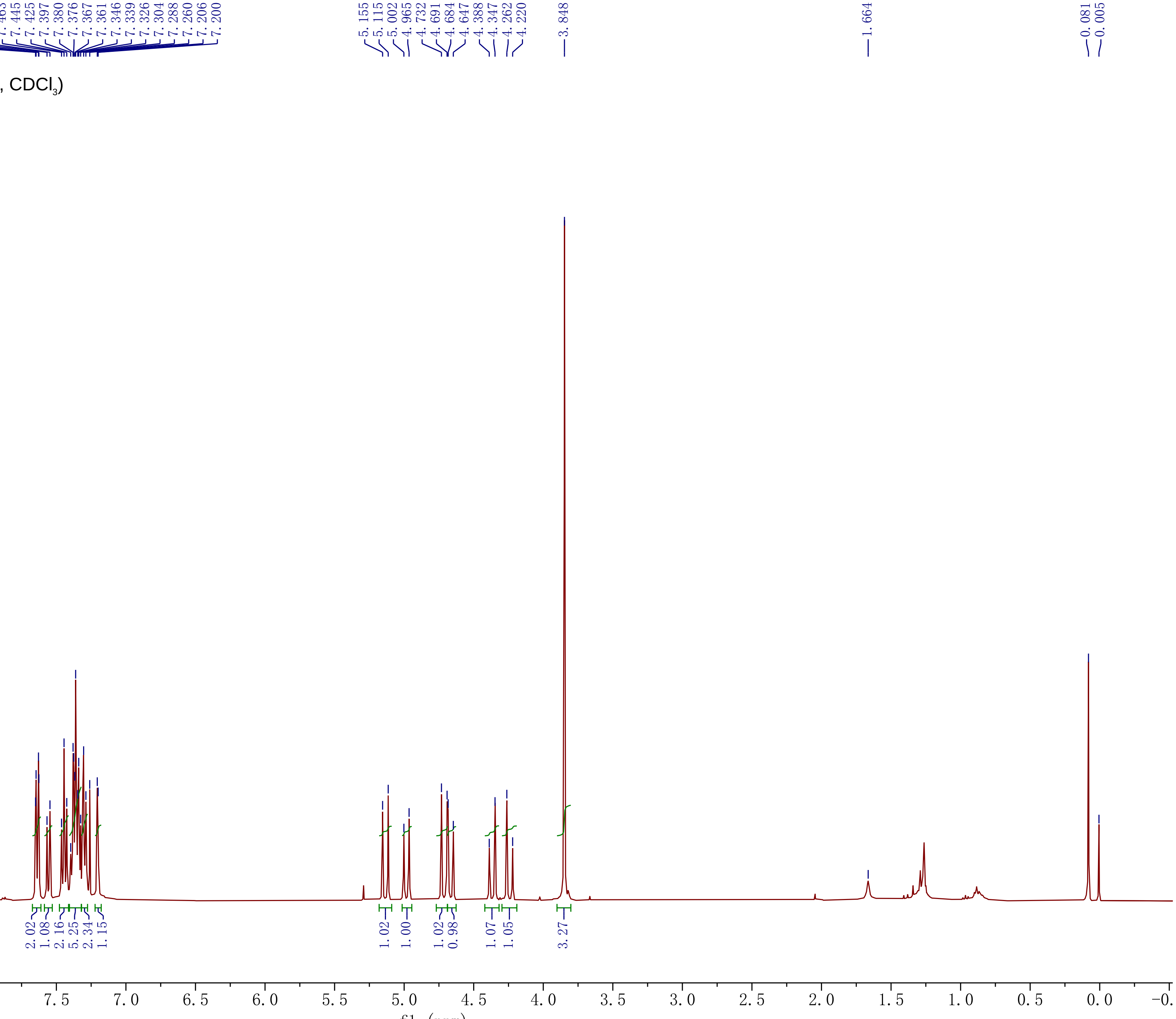

Fig. 3: 5d, ^{13}C NMR (101 MHz, $CDCl_3$)

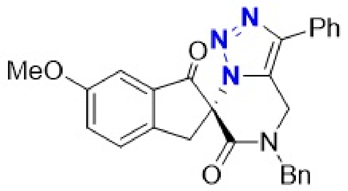

5d

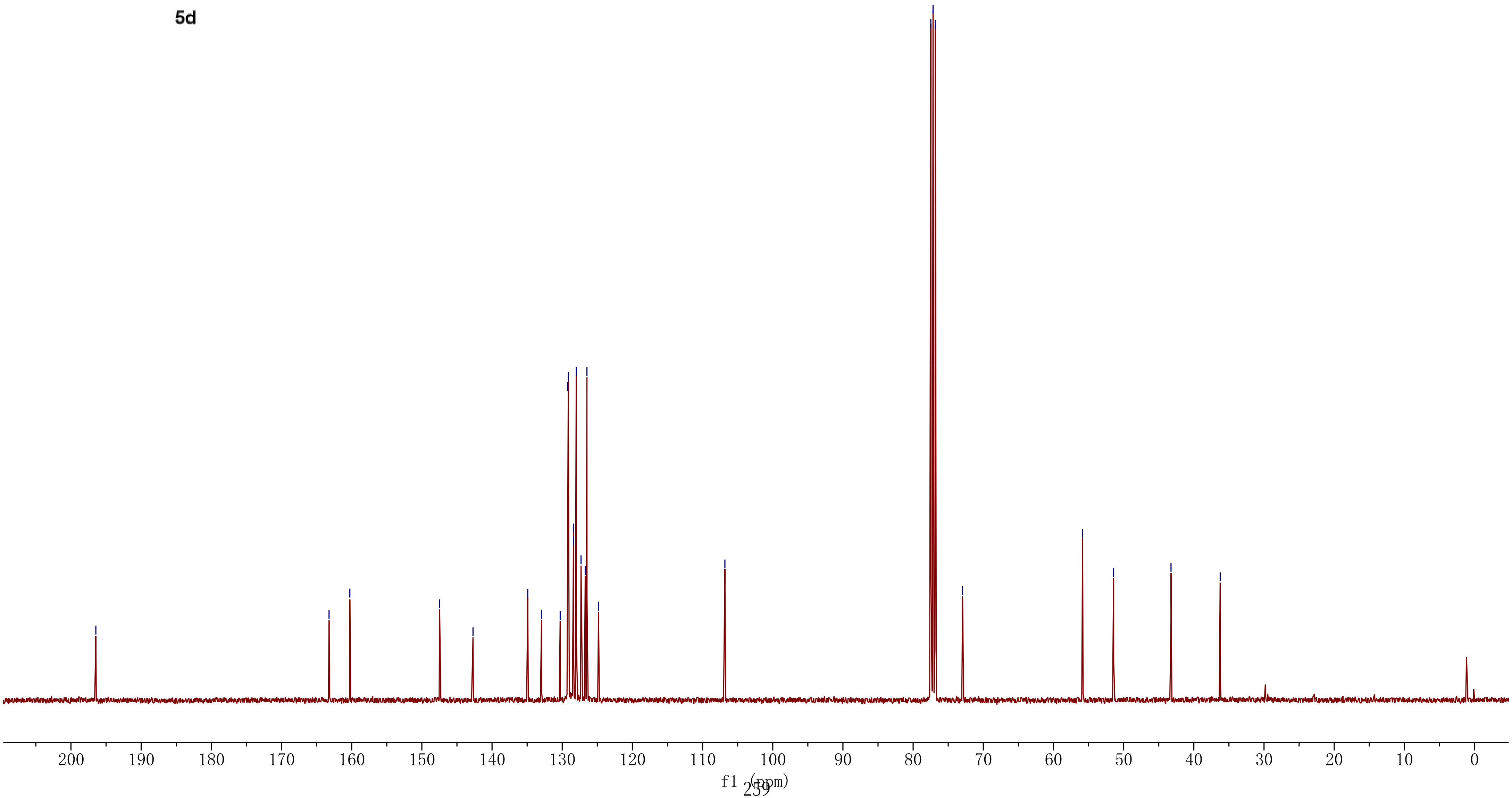

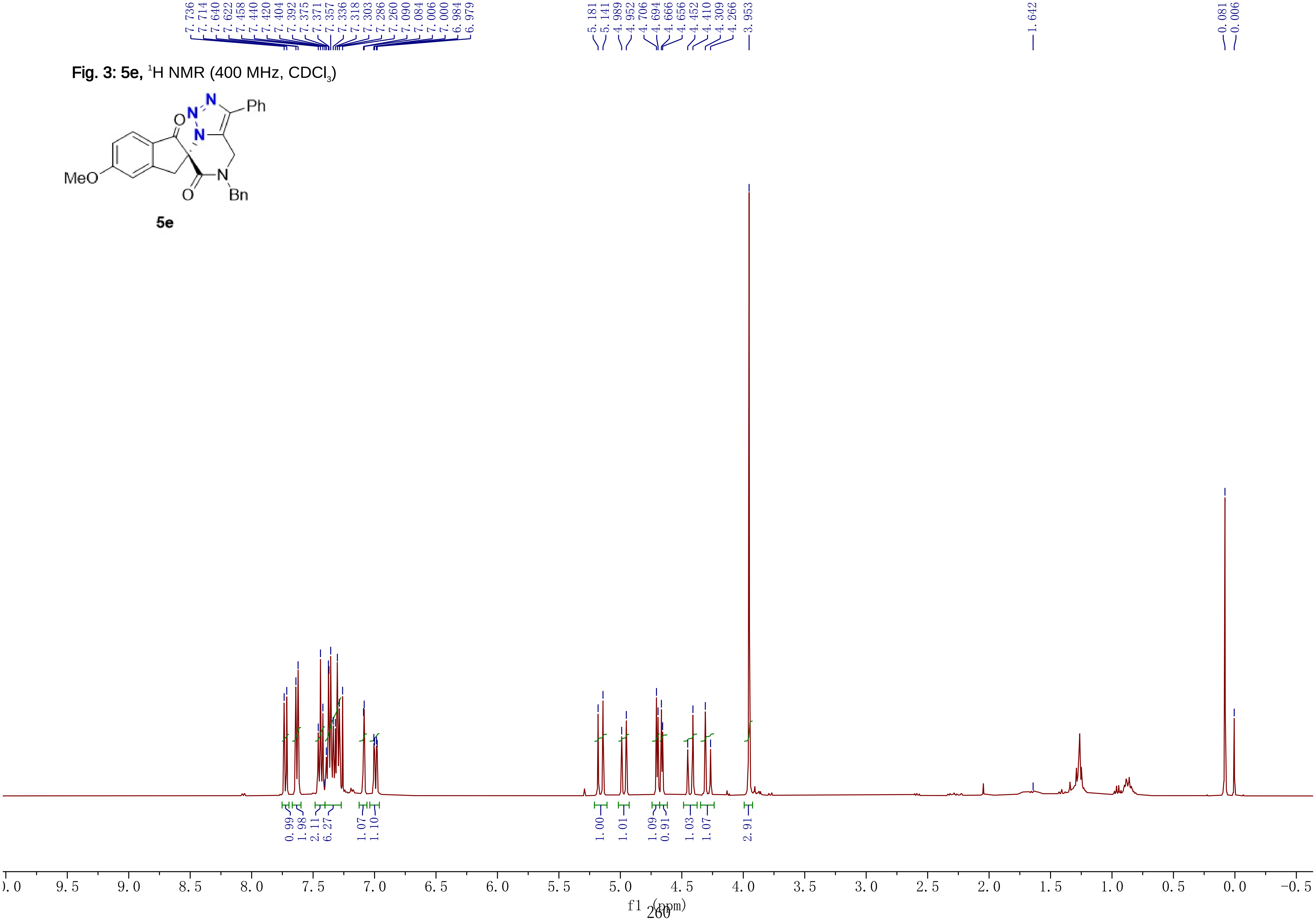

Fig. 3: 5e, ¹H NMR (400 MHz, $CDCl_3$)

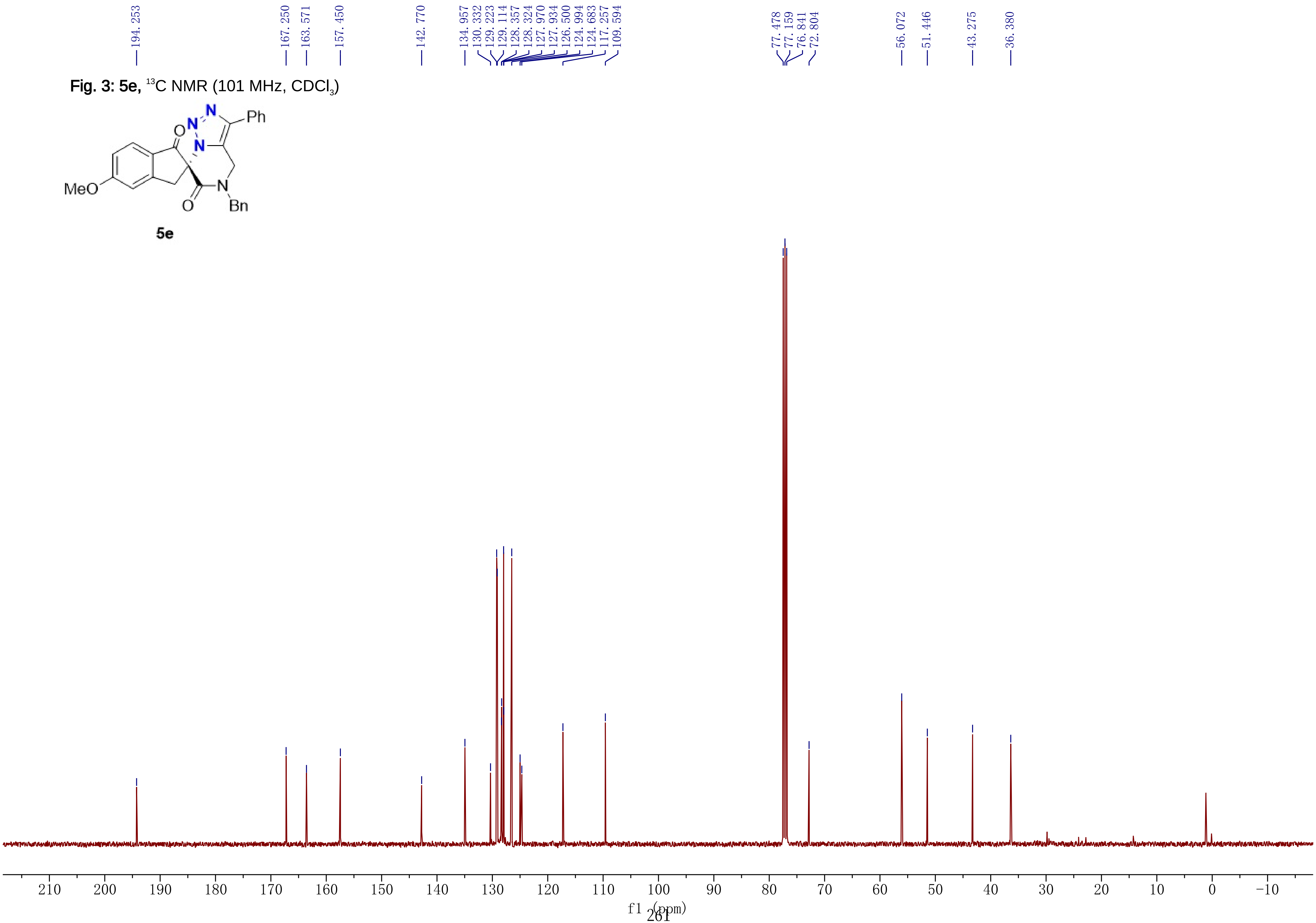

Fig. 3: 5e, ^{13}C NMR (101 MHz, $CDCl_3$)

Fig. 3: 5f, ^{1}H NMR (400 MHz, $CDCl_3$)

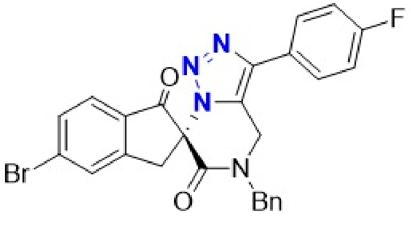

5f

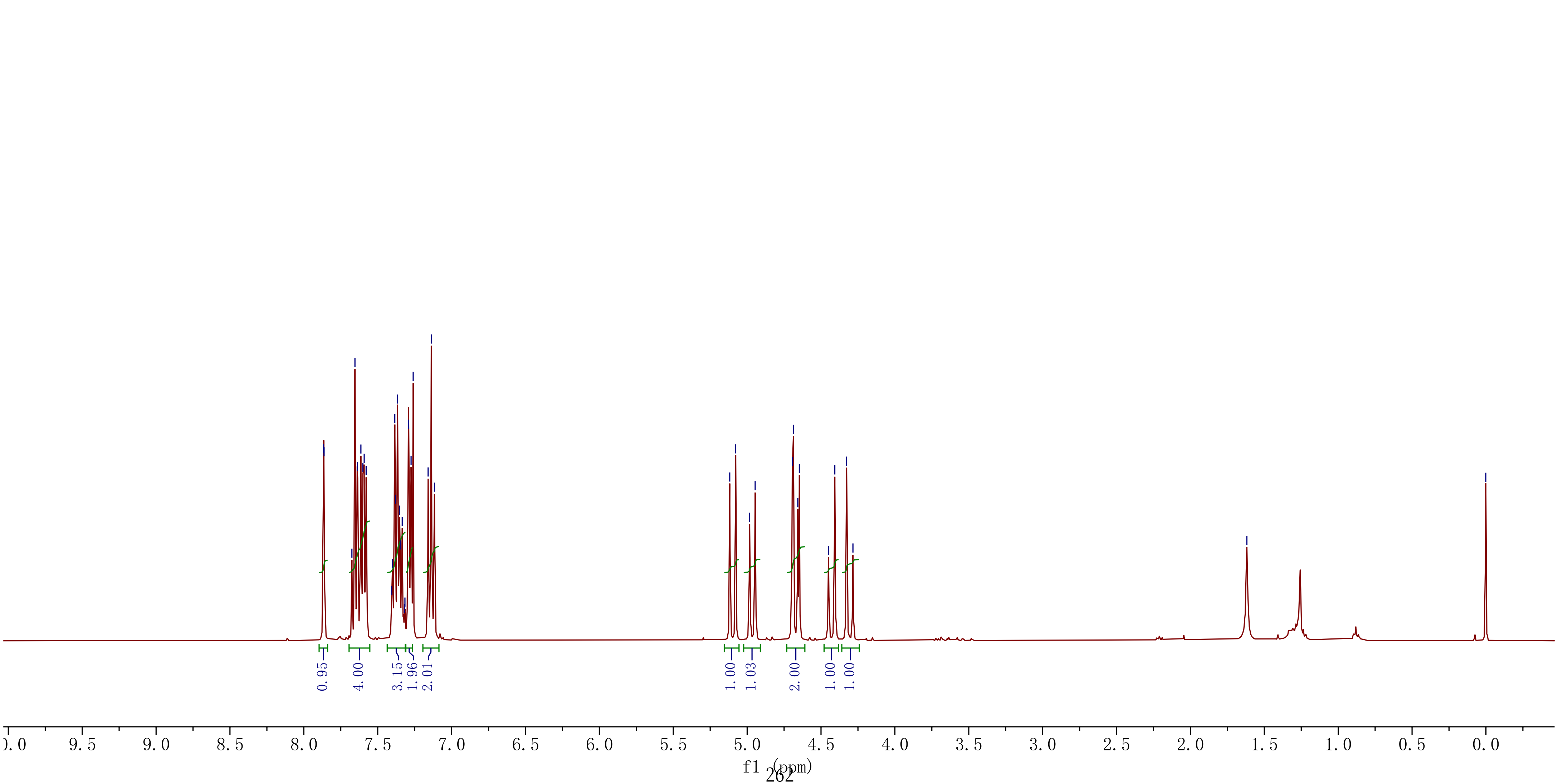

Fig. 3: 5f, ^{19}F NMR (376 MHz, $CDCl_3$)

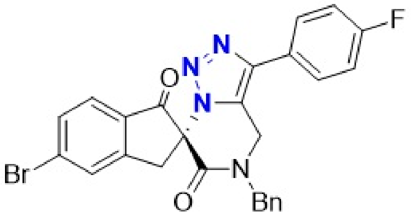

5f

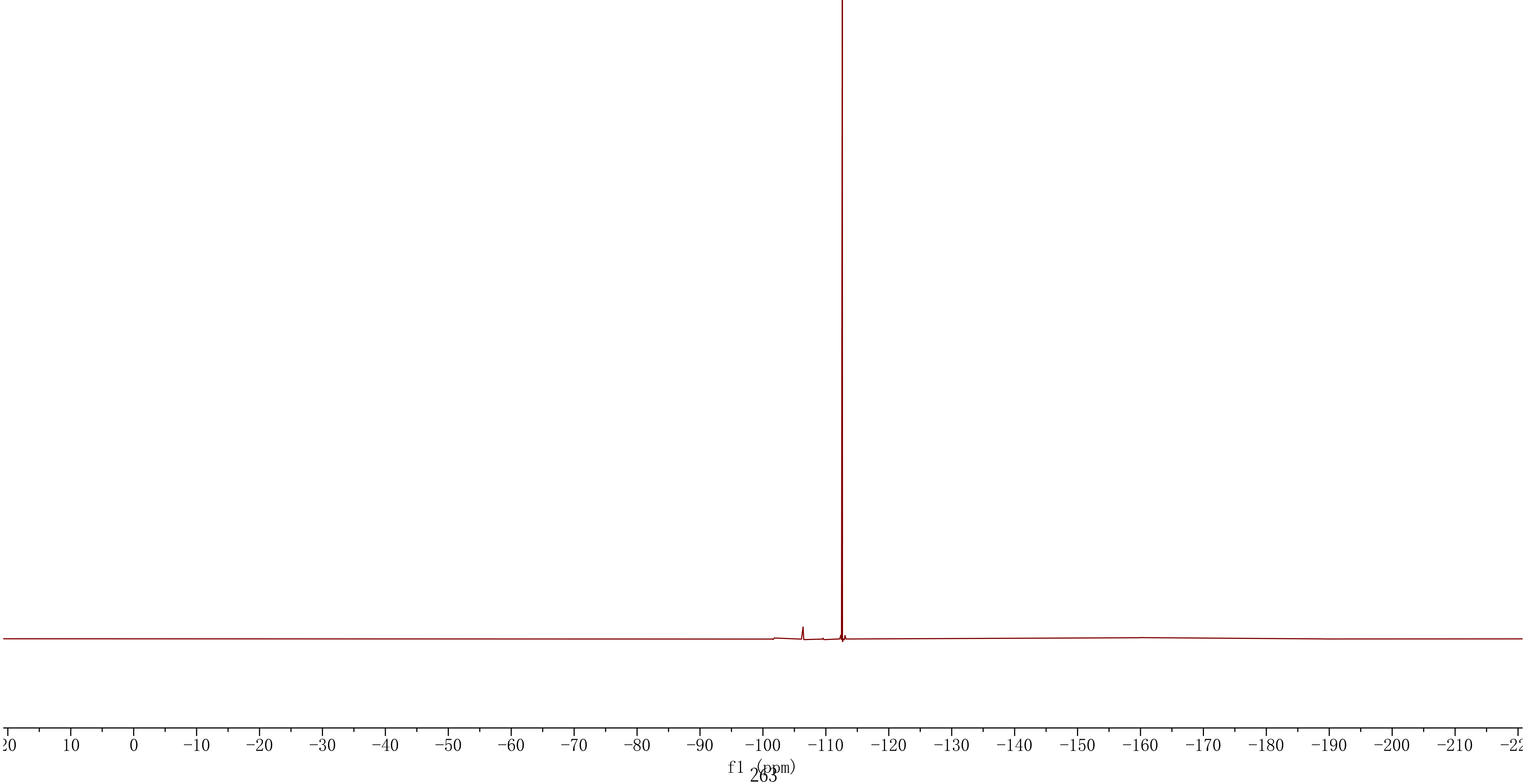

Fig. 3: 5f, ^{13}C NMR (101 MHz, $CDCl_3$)

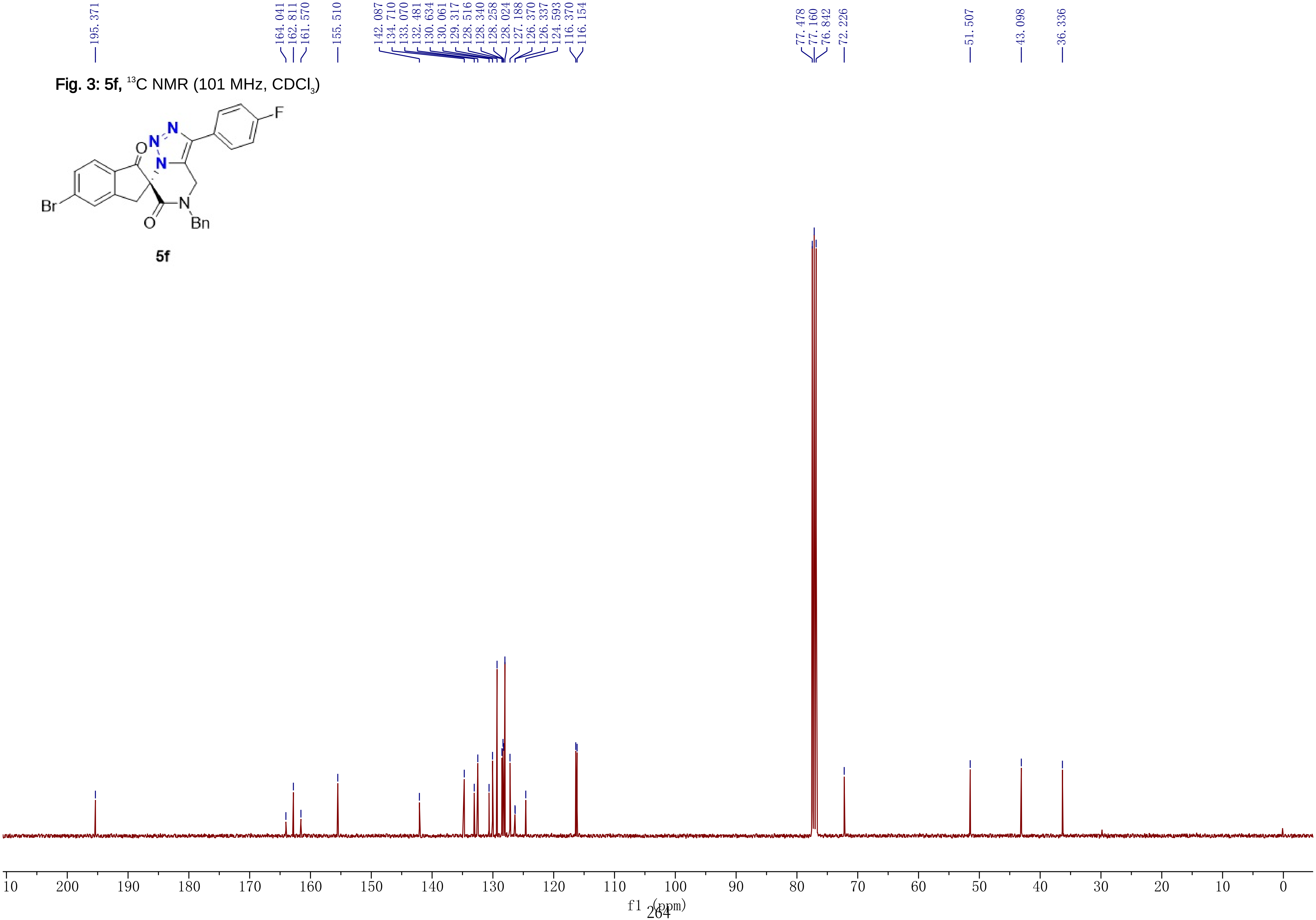

Fig. 3: 5g, ^{1}H NMR (400 MHz, $CDCl_3$)

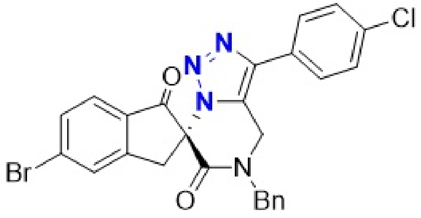

5g

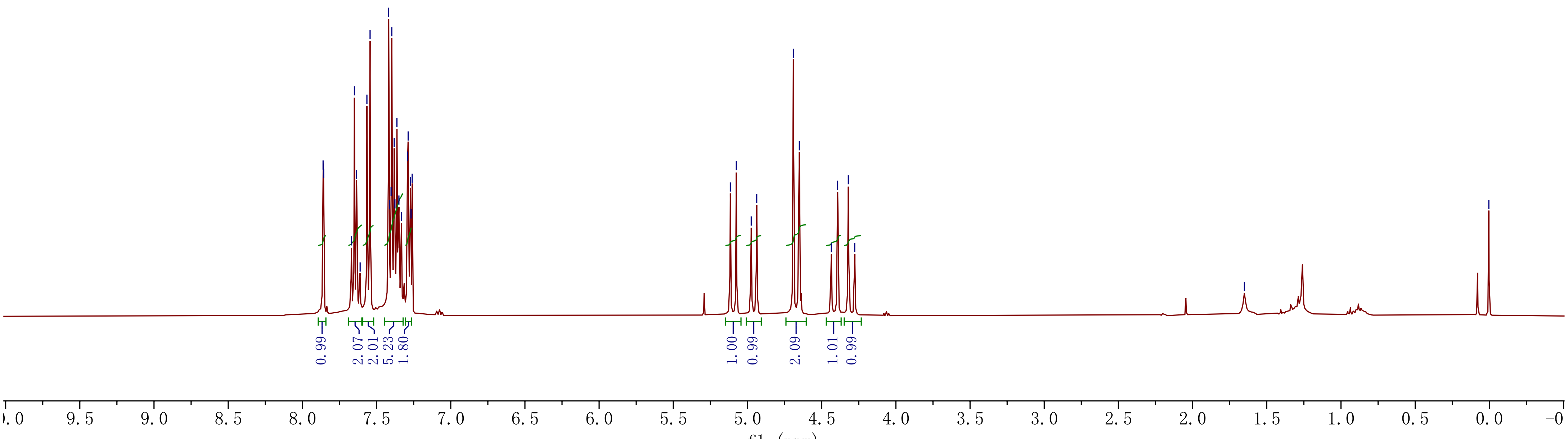

Fig. 3: 5g, ^{13}C NMR (101 MHz, $CDCl_3$)

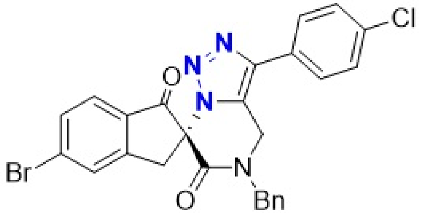

5g

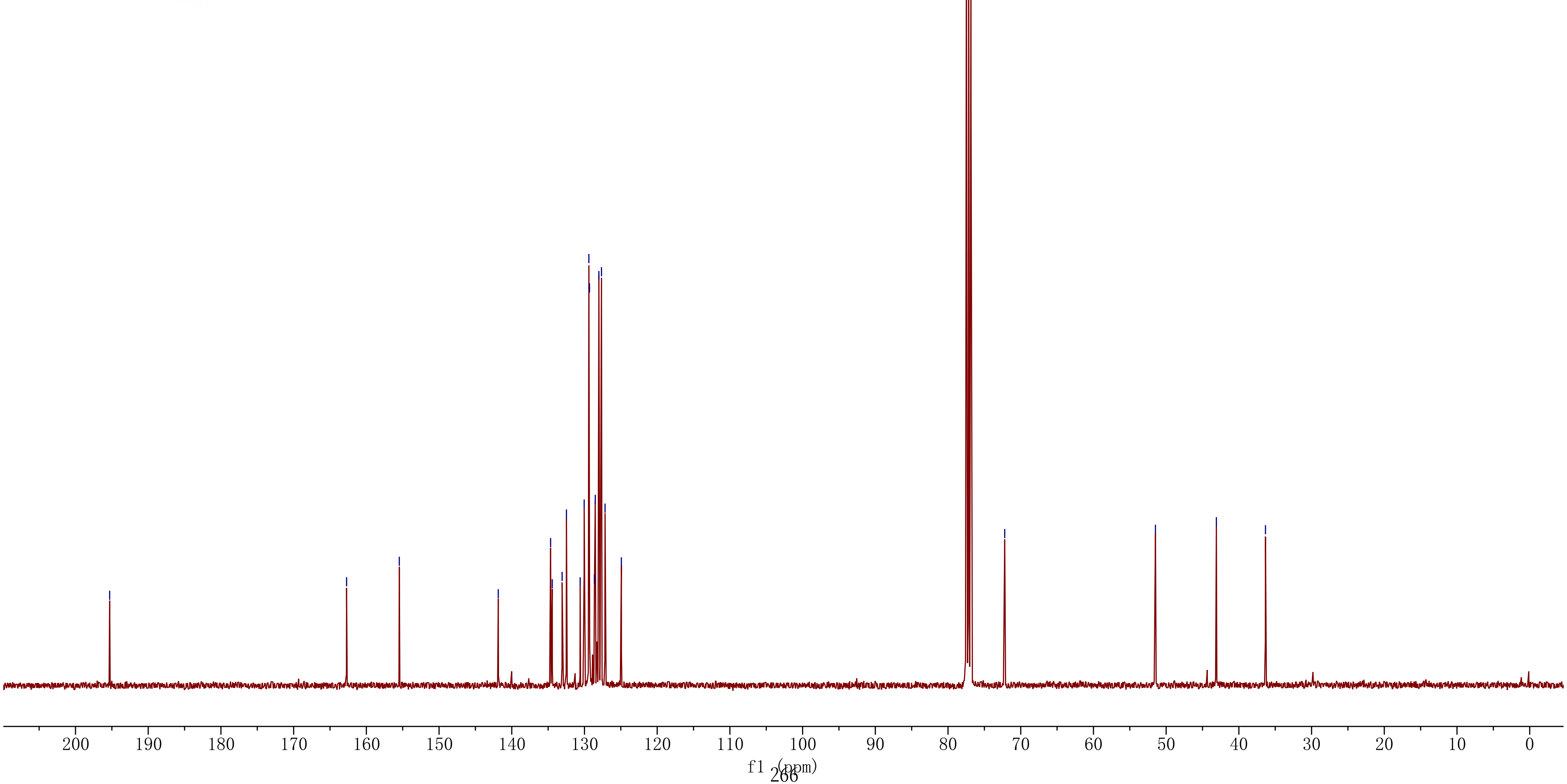

Fig. 3: 5h, ^{1}H NMR (400 MHz, $CDCl_3$)

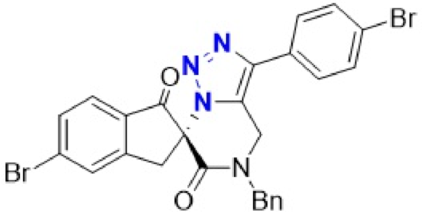

5h

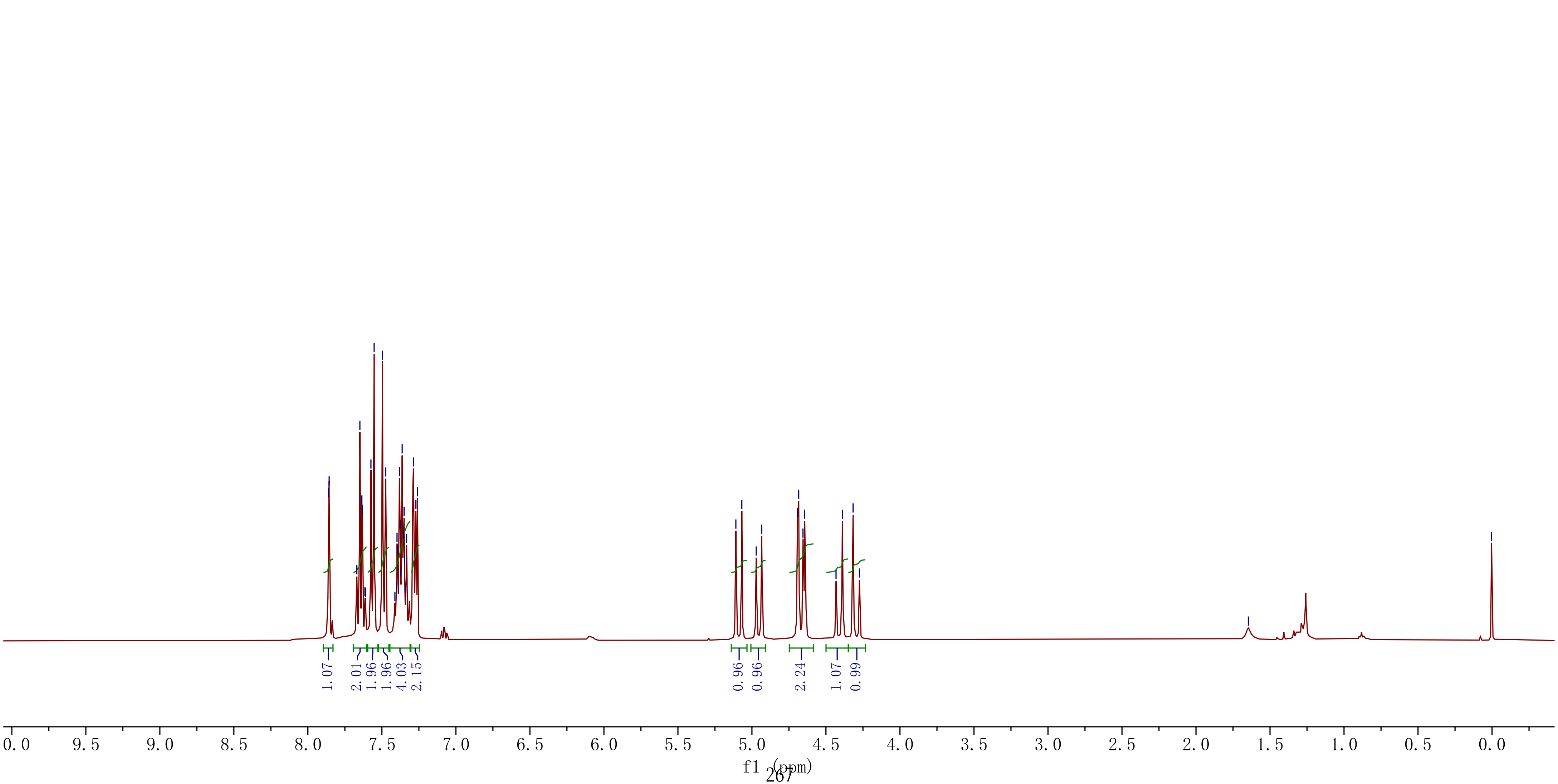

Fig. 3: 5h, ^{13}C NMR (101 MHz, $CDCl_3$)

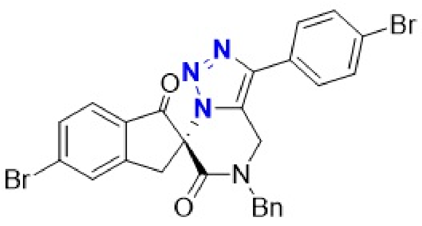

5h

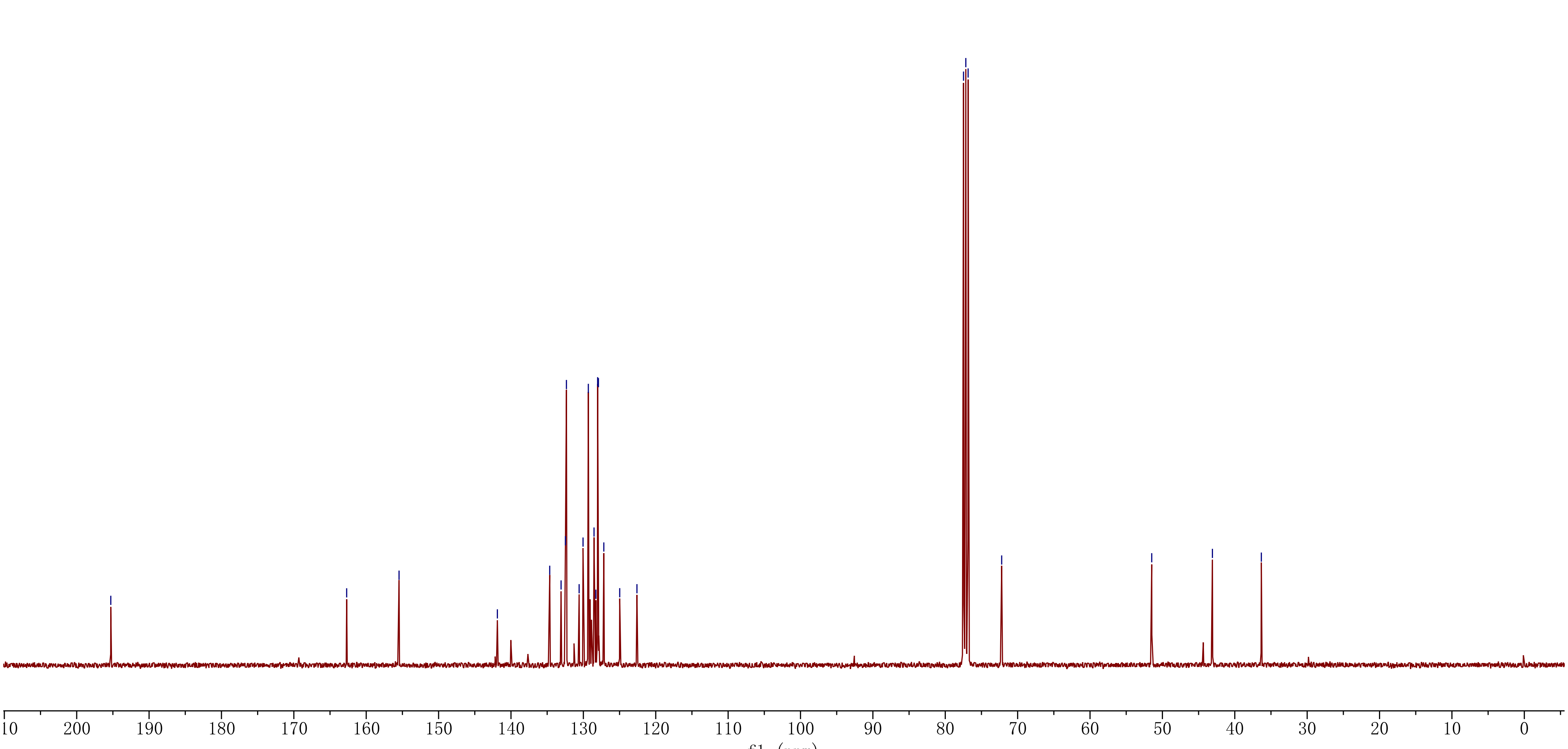

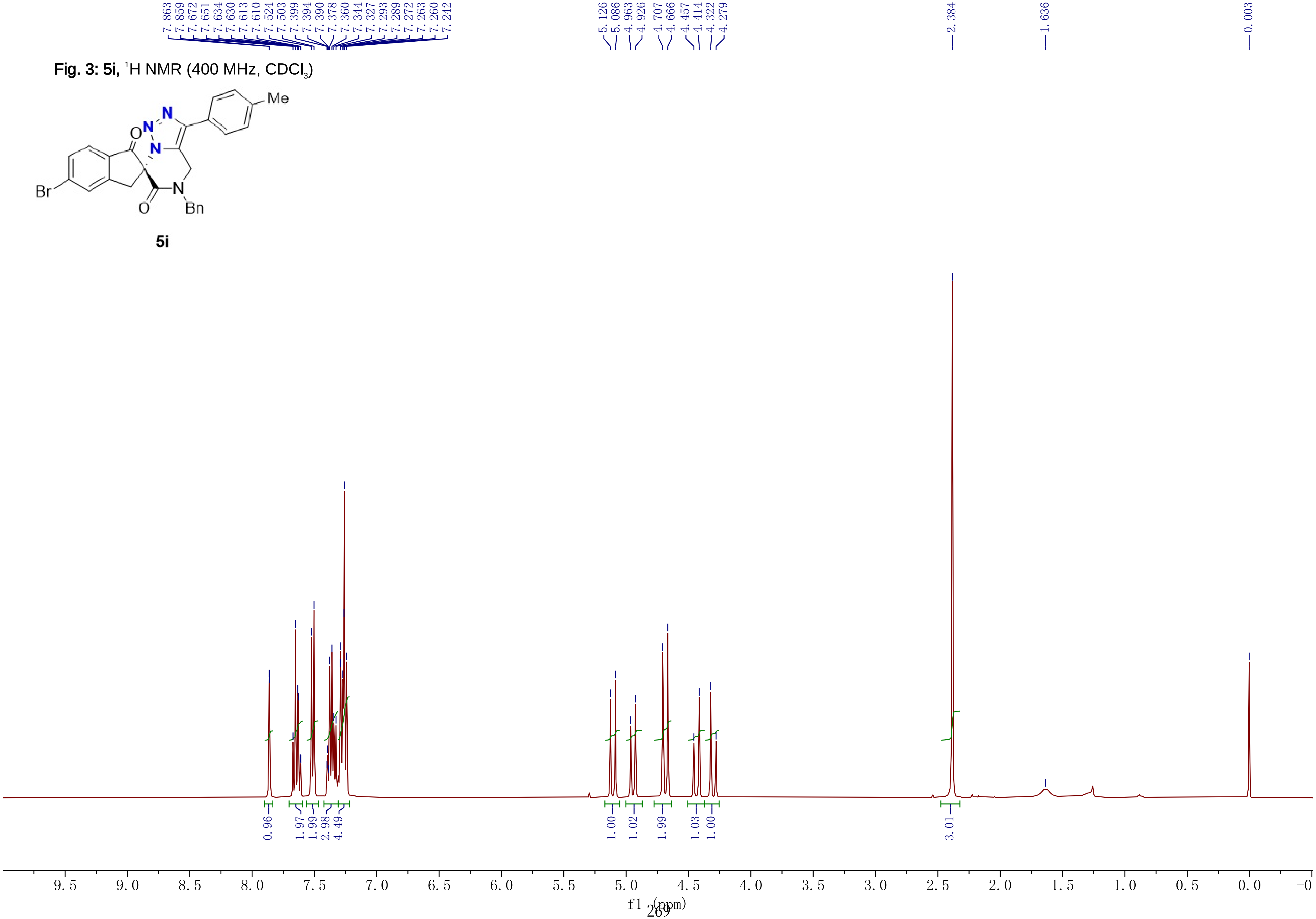

Fig. 3: 5i, ^{1}H NMR (400 MHz, $CDCl_3$)

5i

Fig. 3: 5i, ^{13}C NMR (101 MHz, $CDCl_3$)

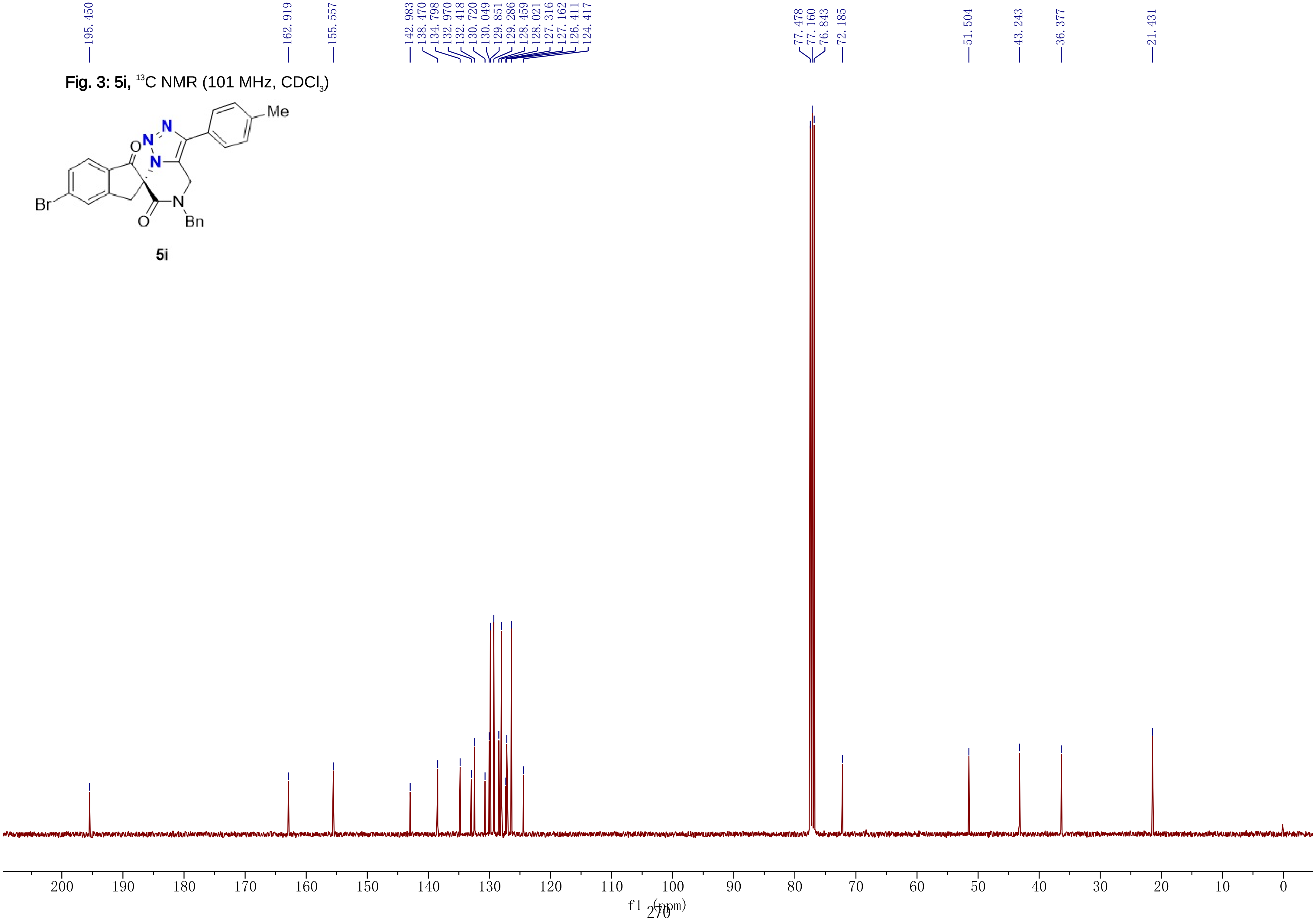

5i

Fig. 3: 5j, ^{1}H NMR (400 MHz, $CDCl_3$)

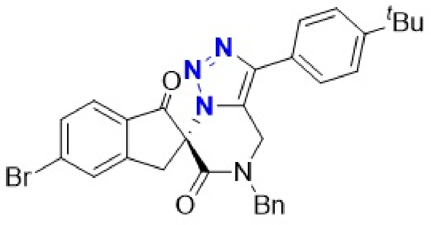

5j

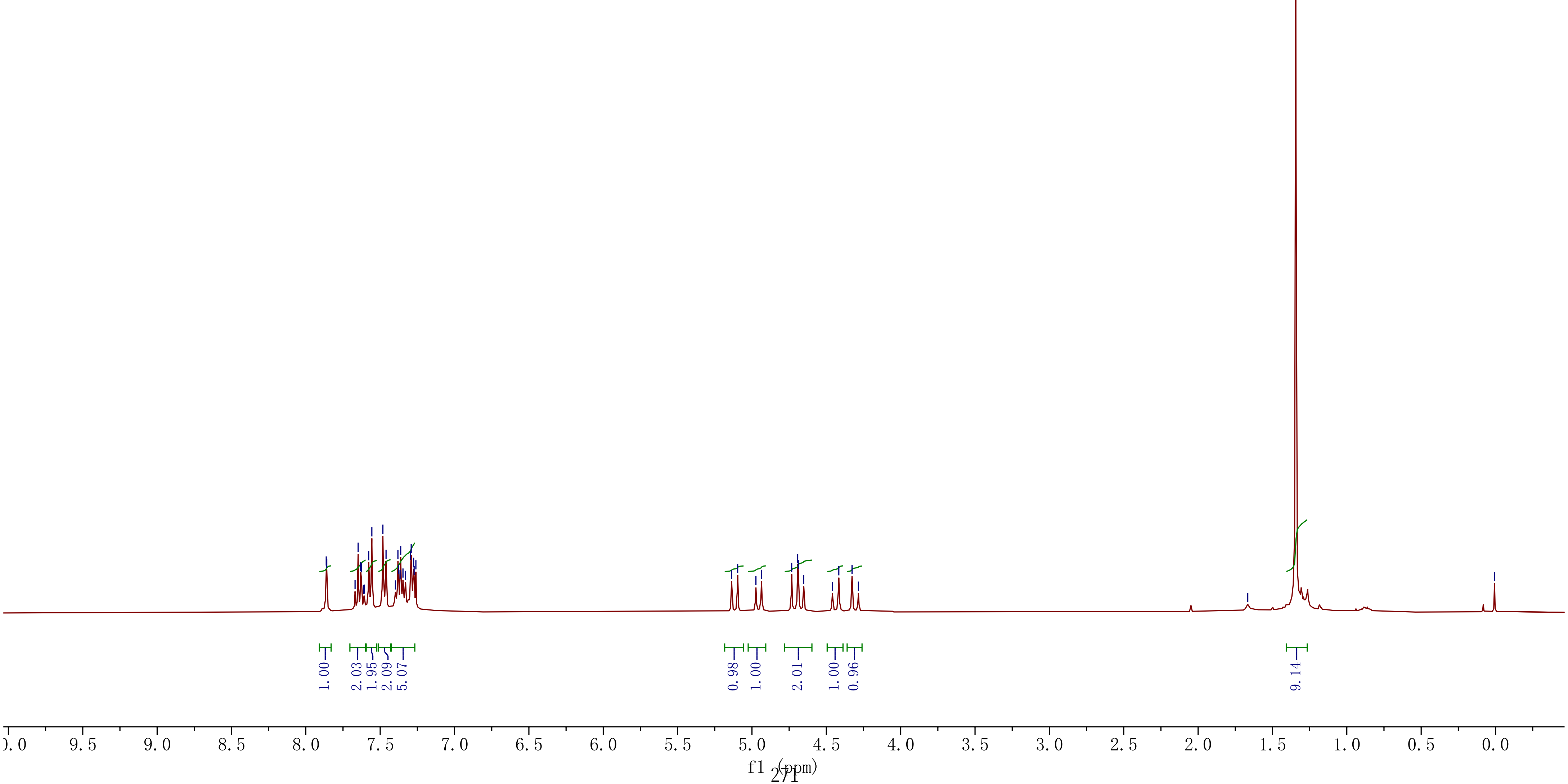

Fig. 3: 5j, ^{13}C NMR (101 MHz, $CDCl_3$)

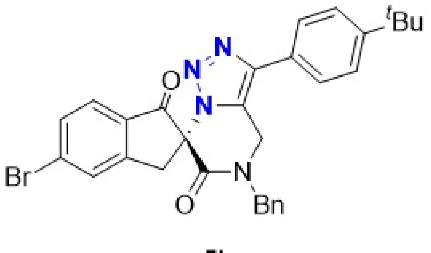

5j

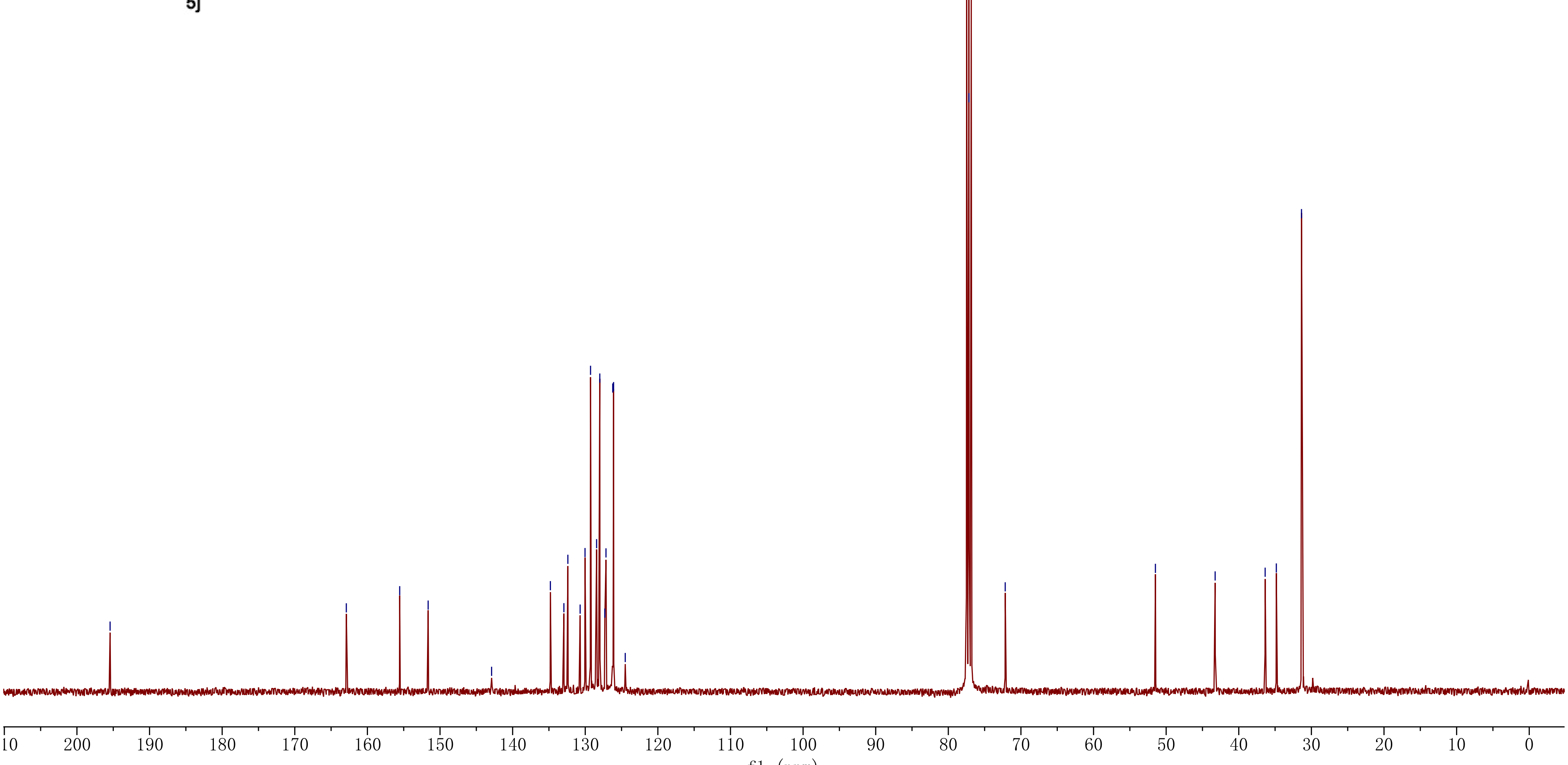

Fig. 3: 5k, ^{1}H NMR (400 MHz, $CDCl_3$)

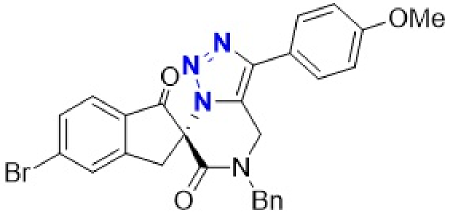

5k

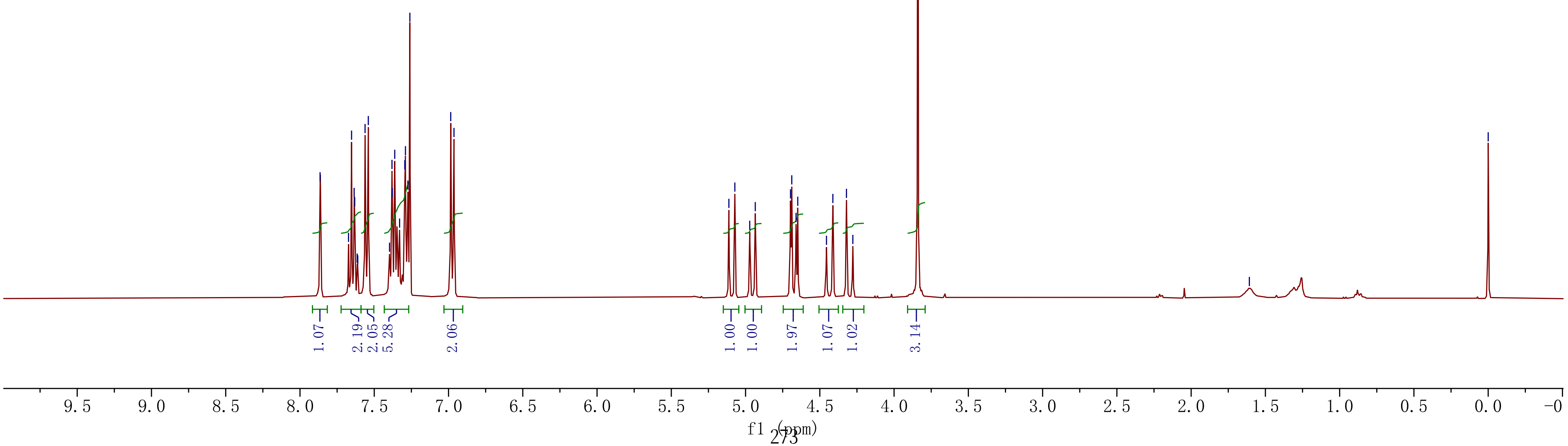

Fig. 3: 5k, ^{13}C NMR (101 MHz, $CDCl_3$)

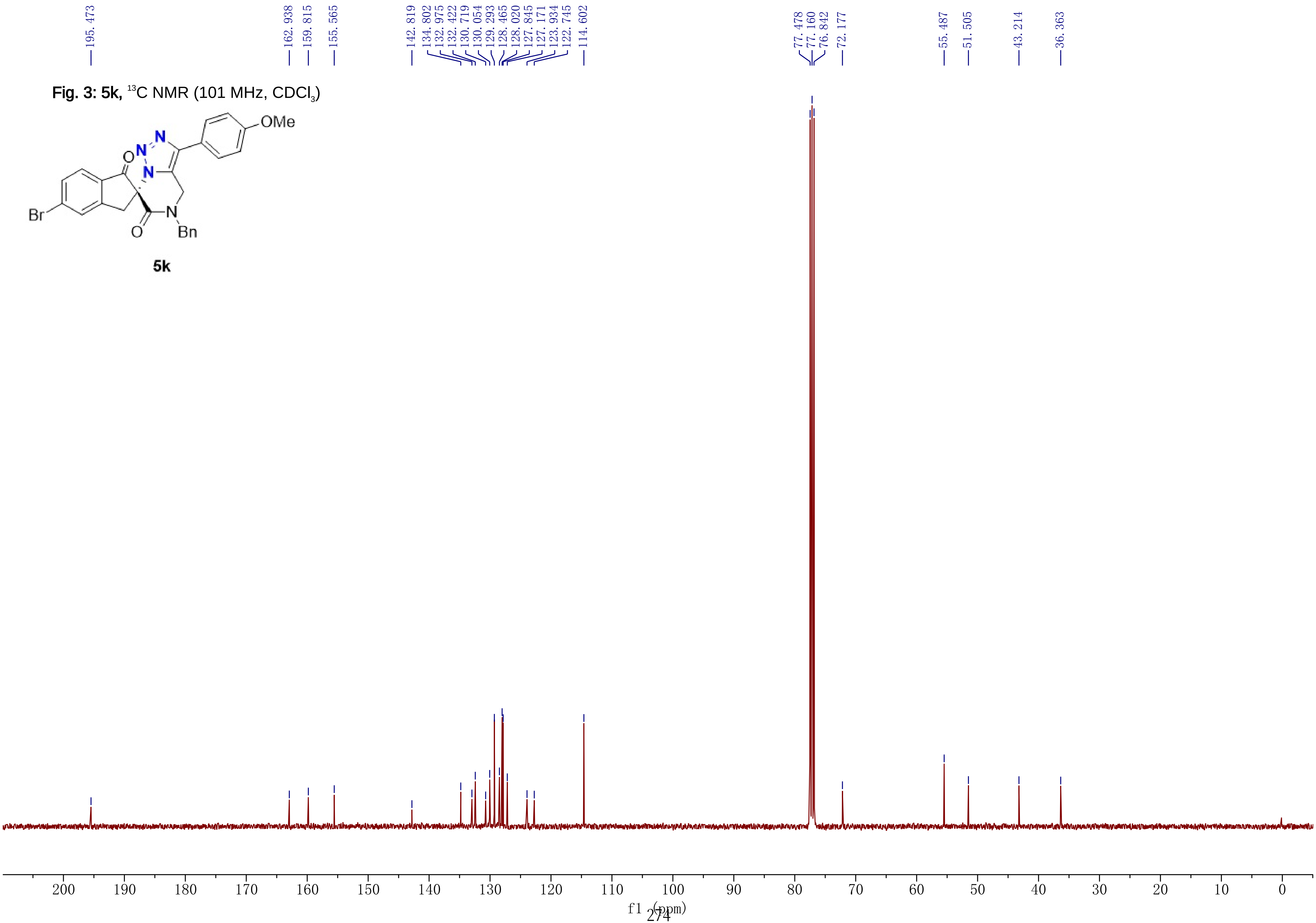

Fig. 3: 5l, ^{1}H NMR (400 MHz, $CDCl_3$)

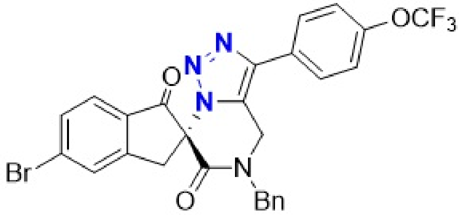

5l

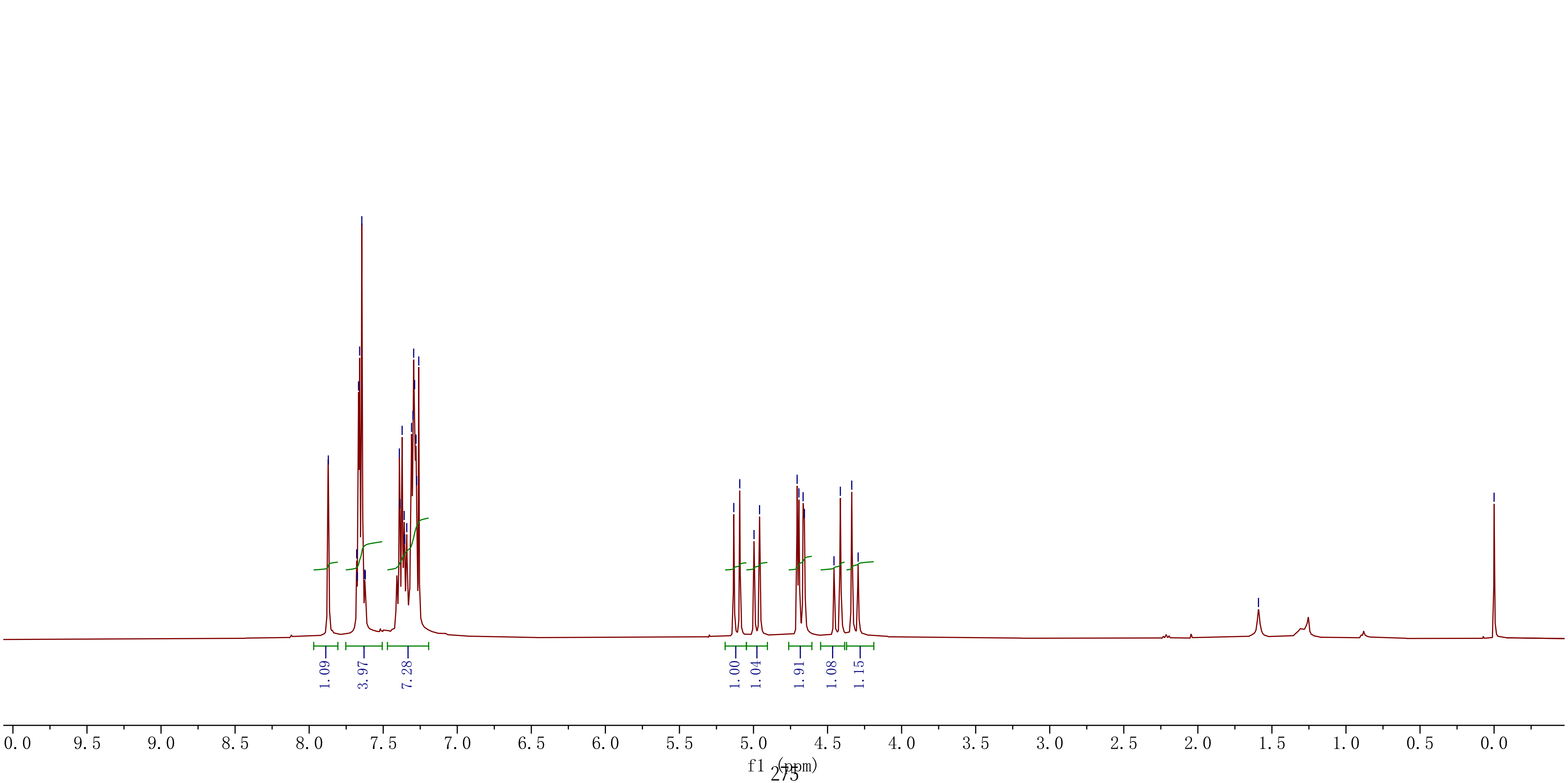

Fig. 2: 5l, ^{19}F NMR (376 MHz, $CDCl_3$)

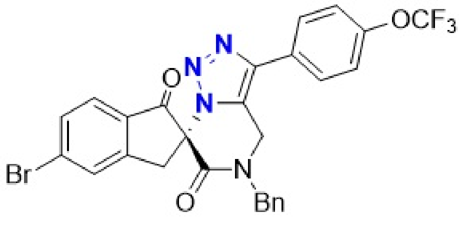

5l

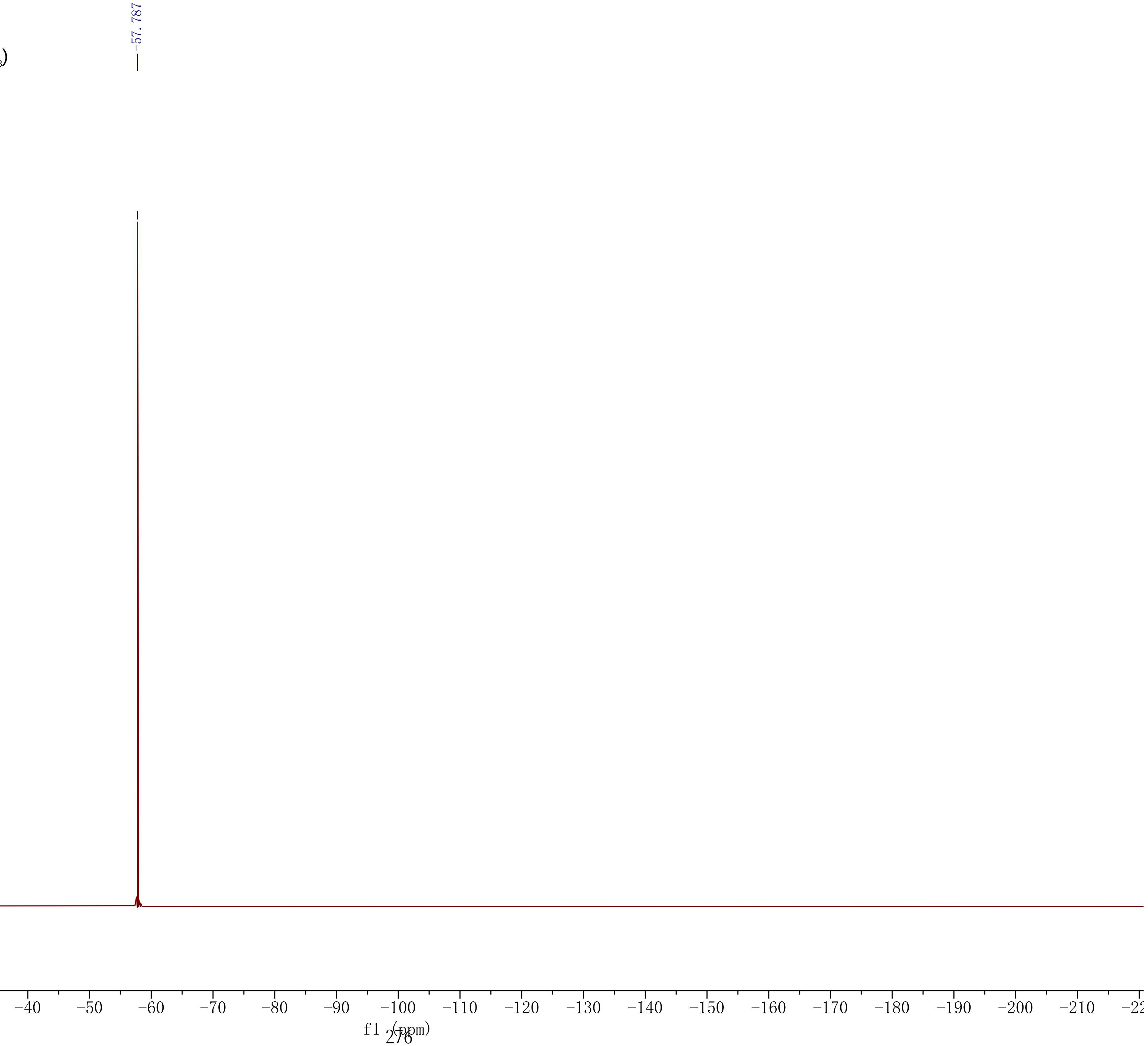

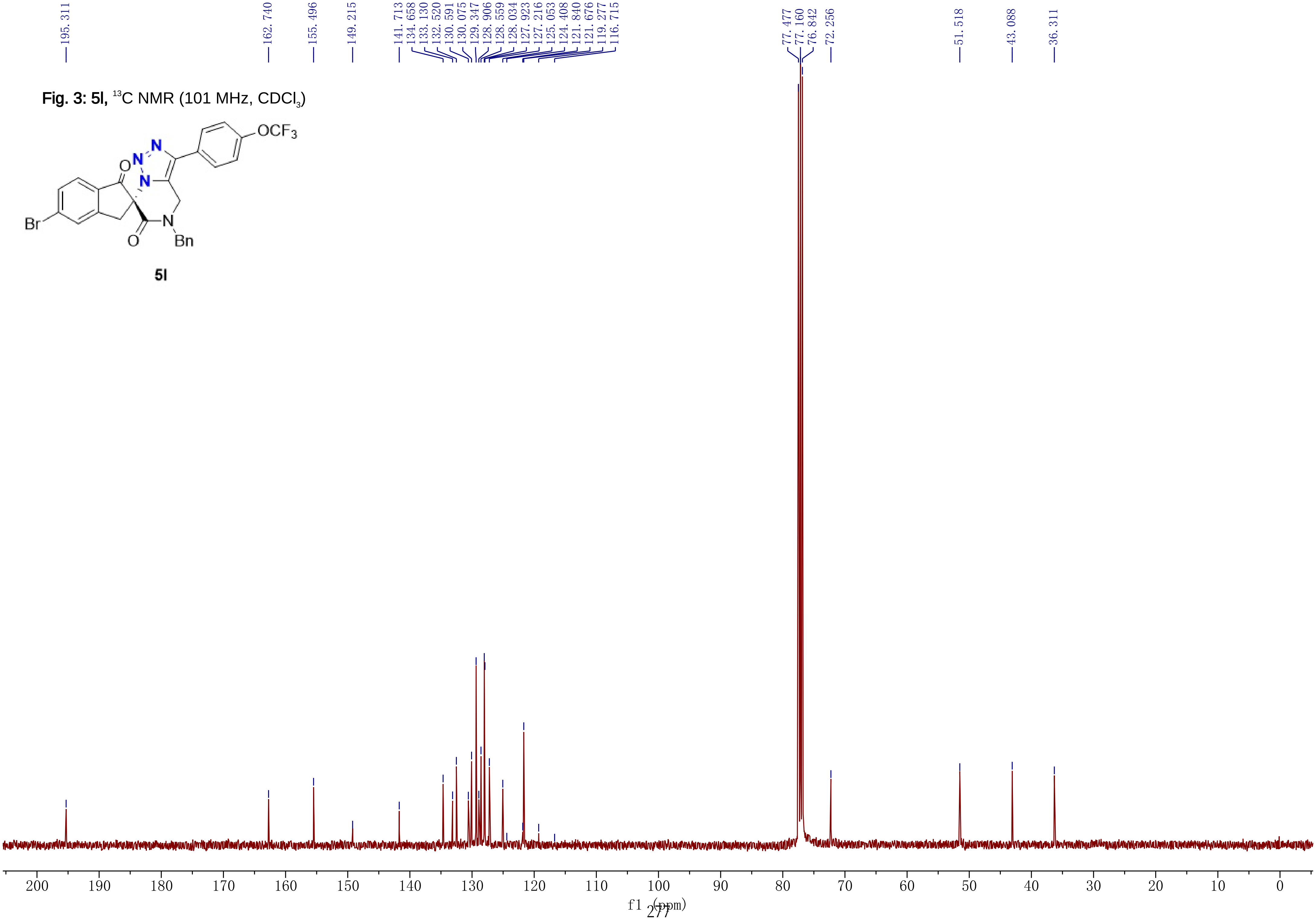

Fig. 3: 5l, ^{13}C NMR (101 MHz, $CDCl_3$)

Fig. 3: 5m, ^{1}H NMR (400 MHz, $CDCl_3$)

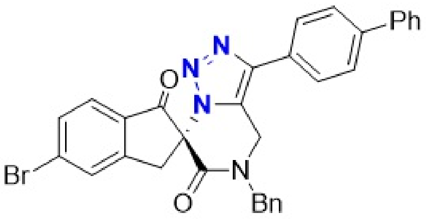

5m

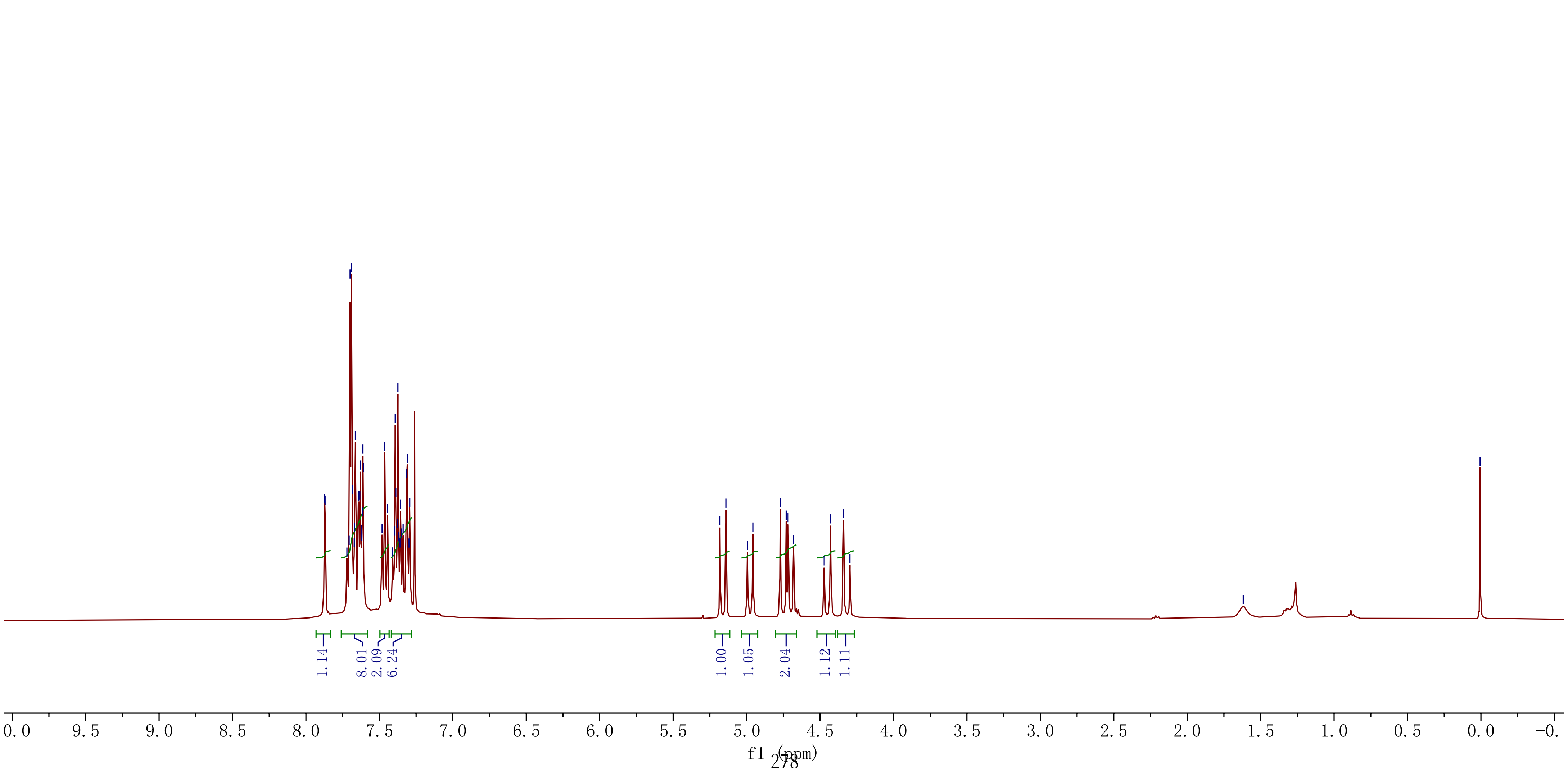

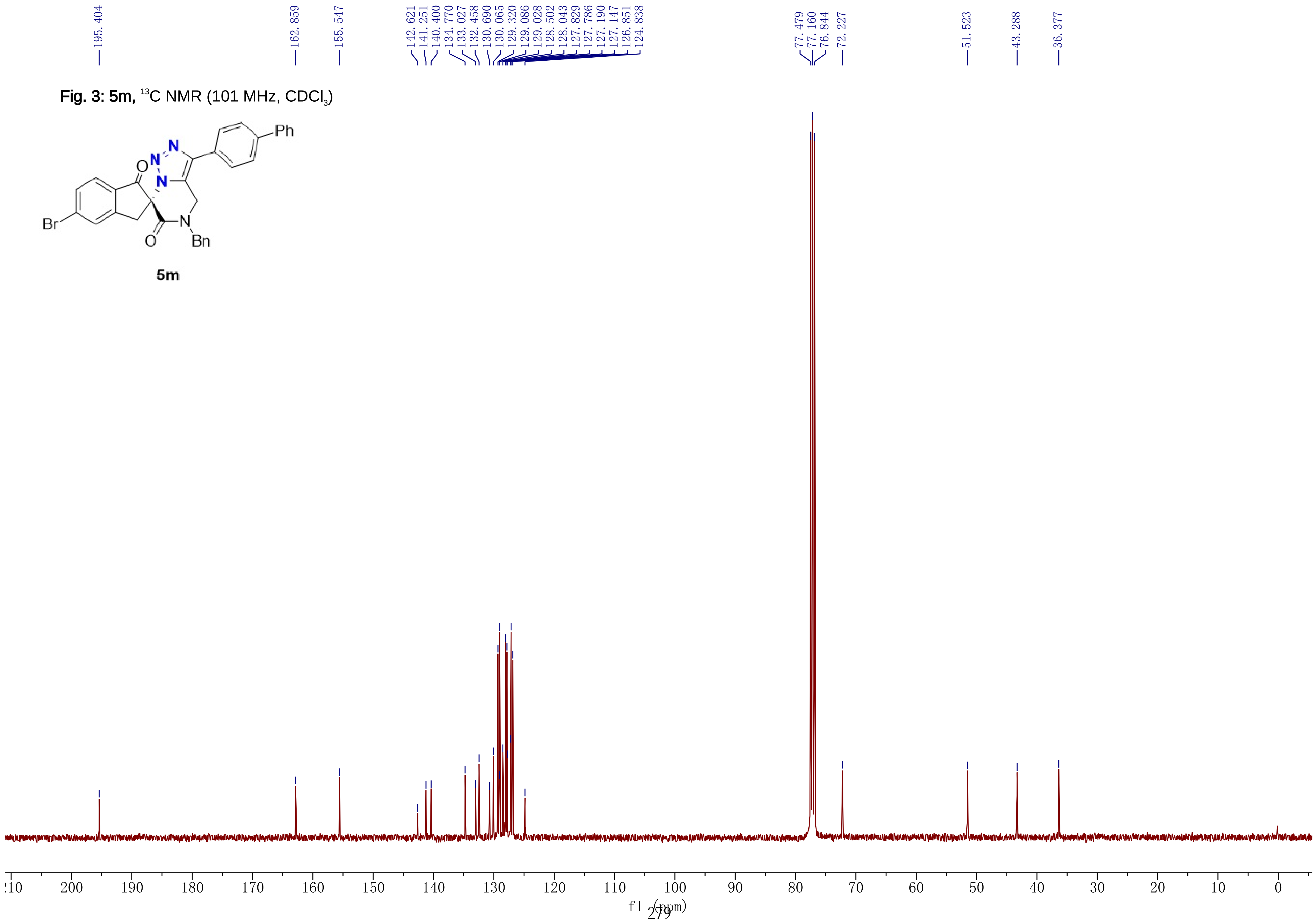

Fig. 3: 5m, ^{13}C NMR (101 MHz, $CDCl_3$)

5m

Fig. 3: 5n, ^{1}H NMR (400 MHz, $CDCl_3$)

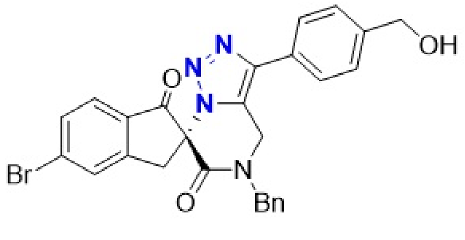

5n

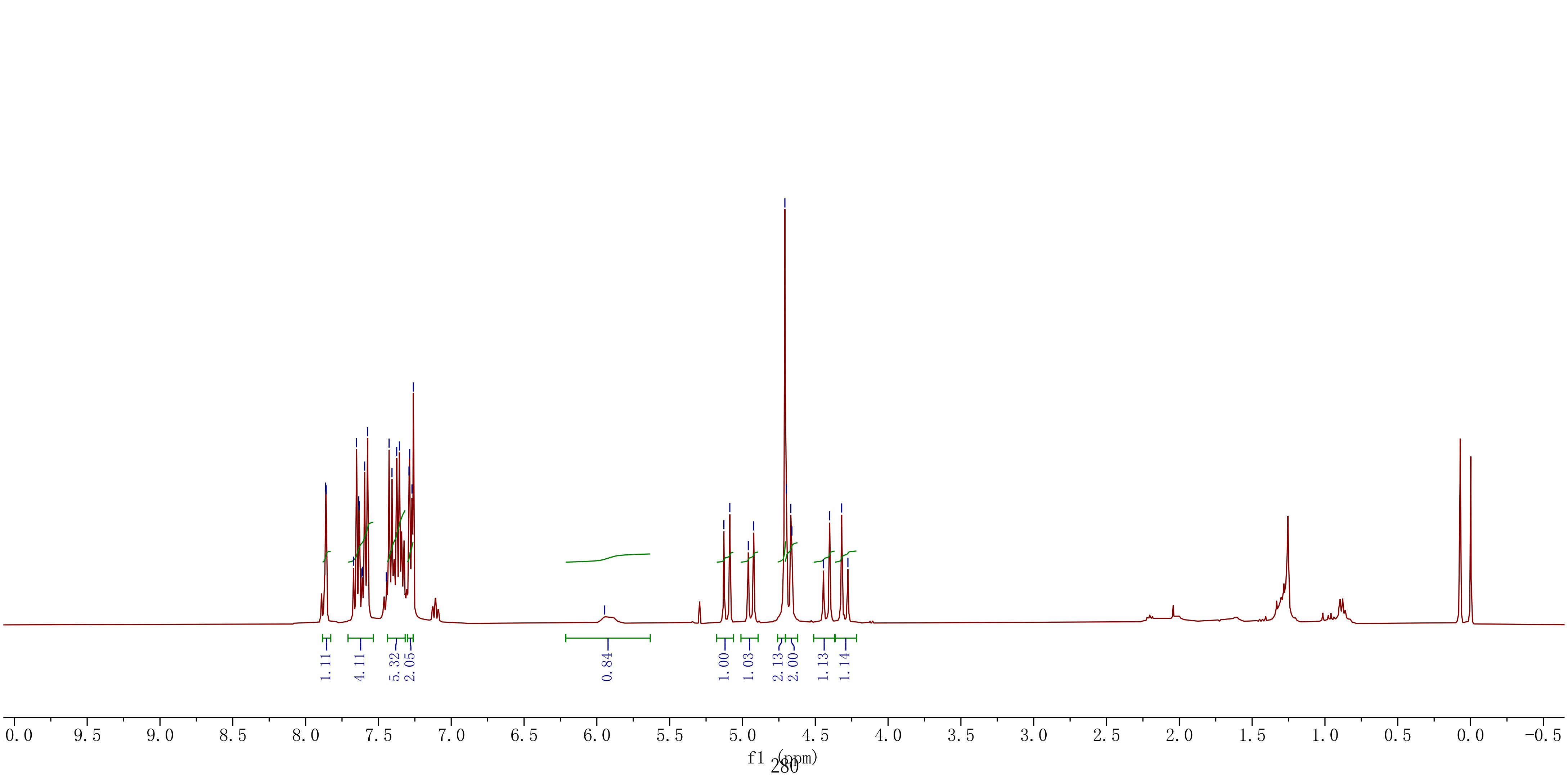

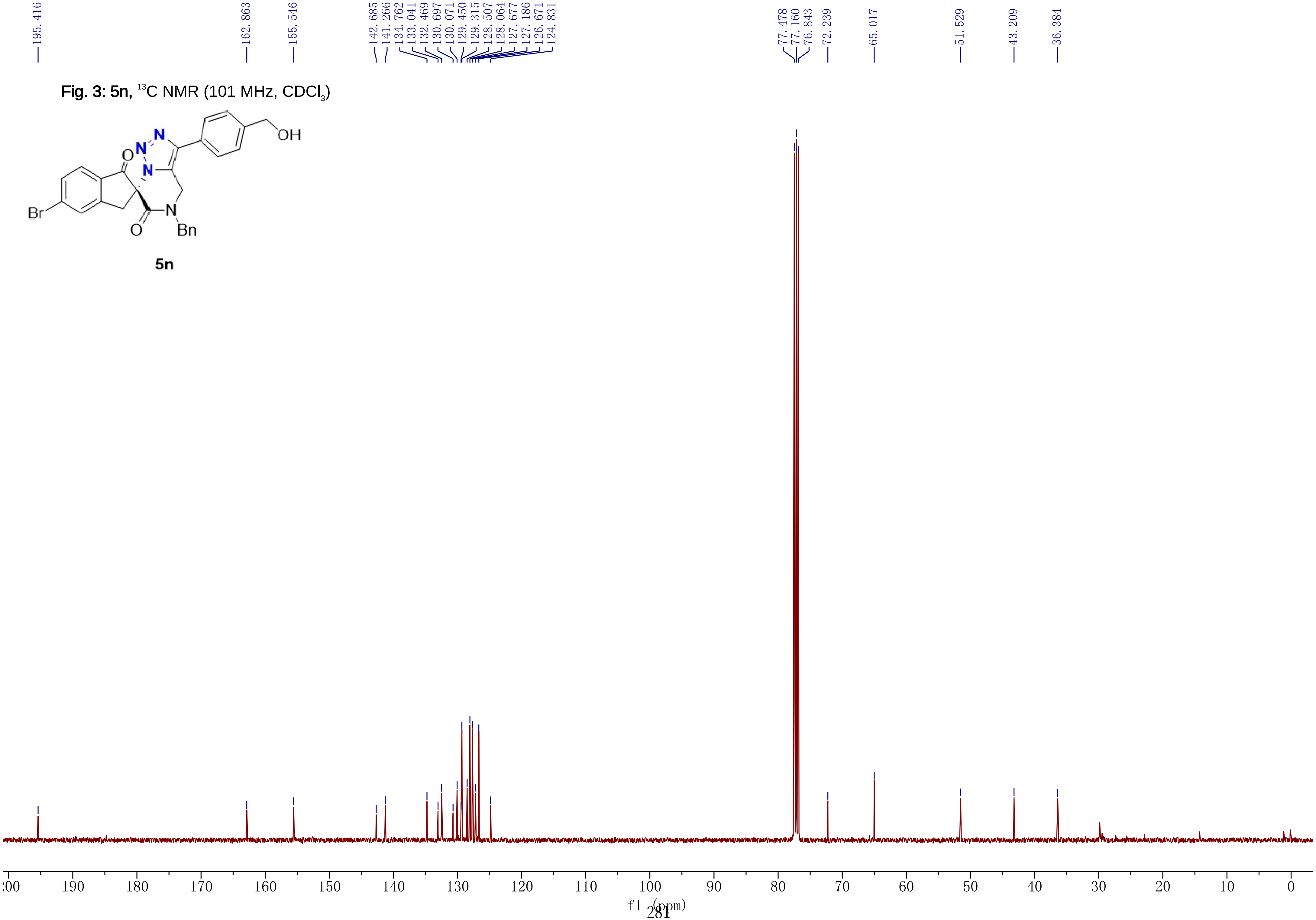

Fig. 3: 5n, ^{13}C NMR (101 MHz, $CDCl_3$)

Fig. 3: 5o, ^{1}H NMR (400 MHz, $CDCl_3$)

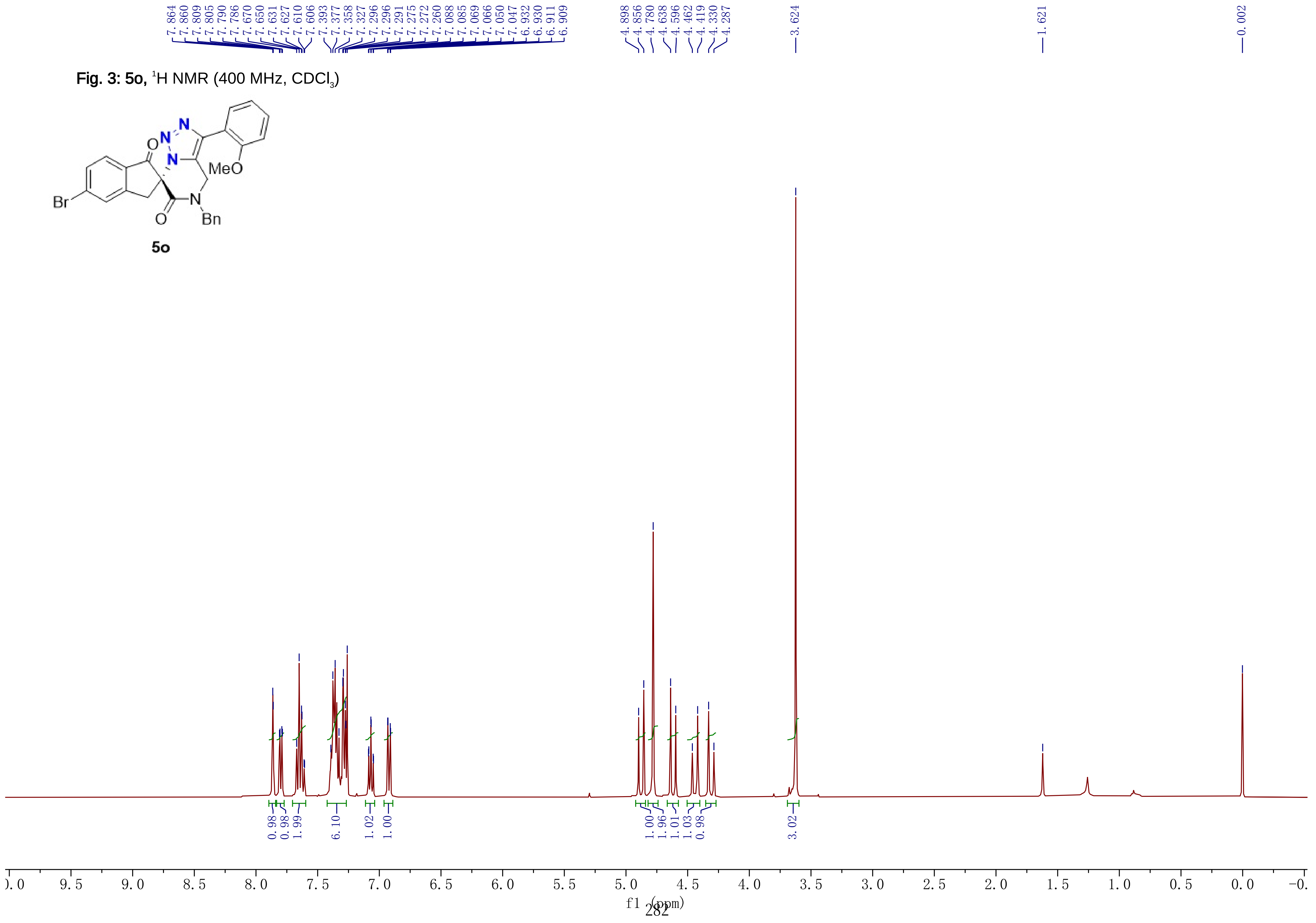

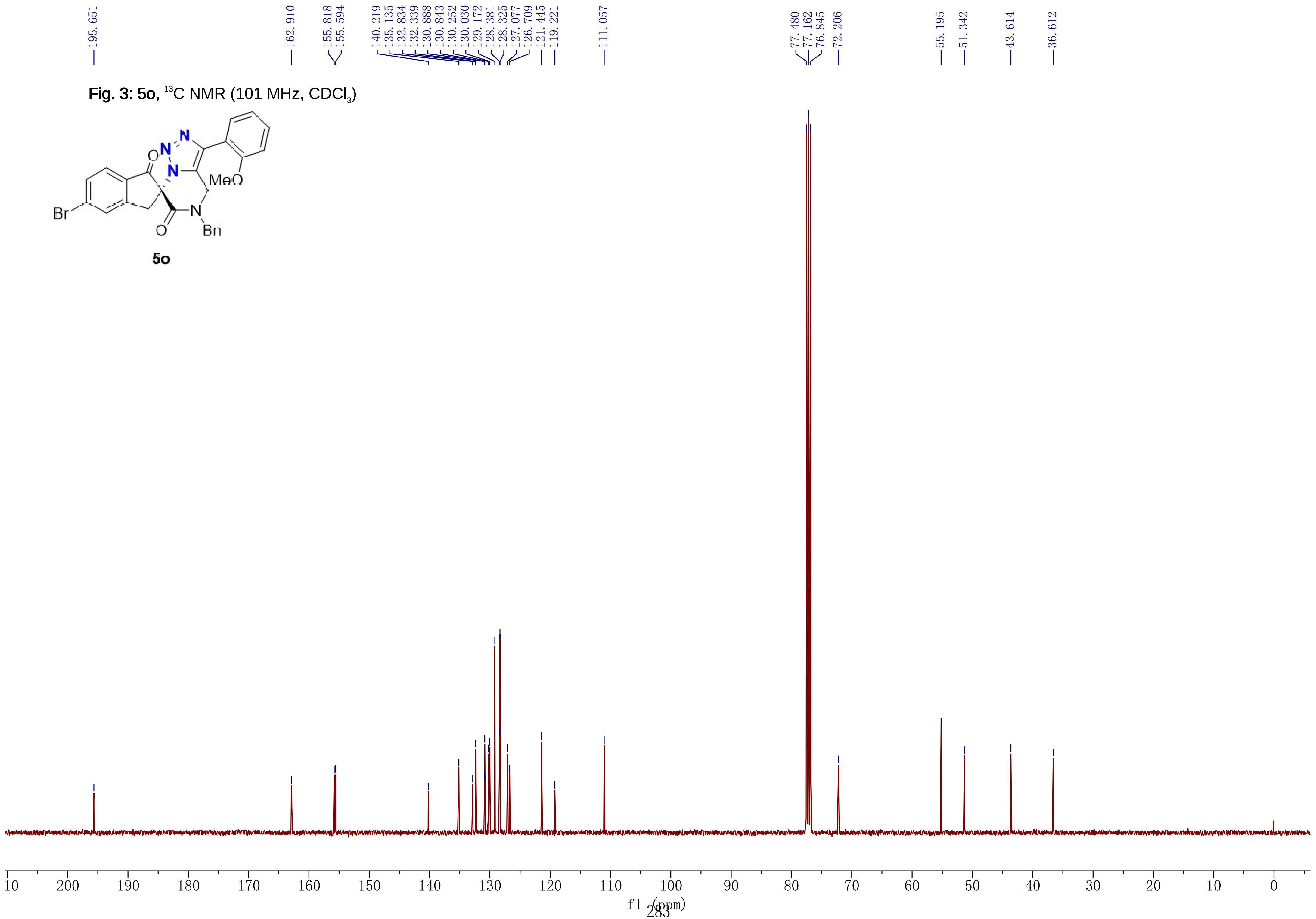

Fig. 3: 5o, ^{13}C NMR (101 MHz, $CDCl_3$)

Fig. 3: 5p, ^{1}H NMR (400 MHz, $CDCl_3$)

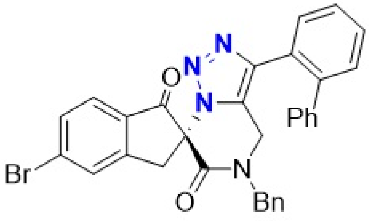

5p

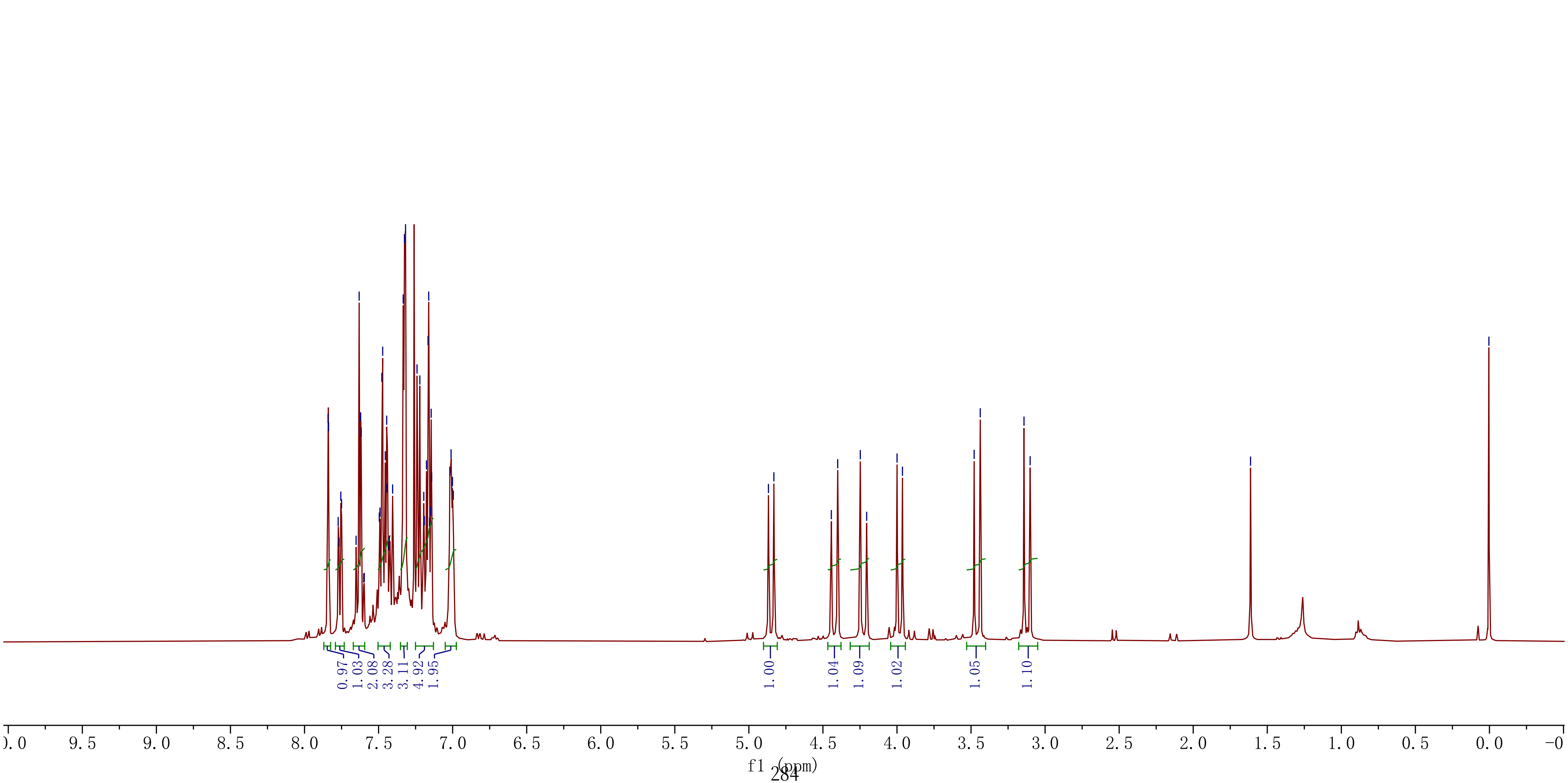

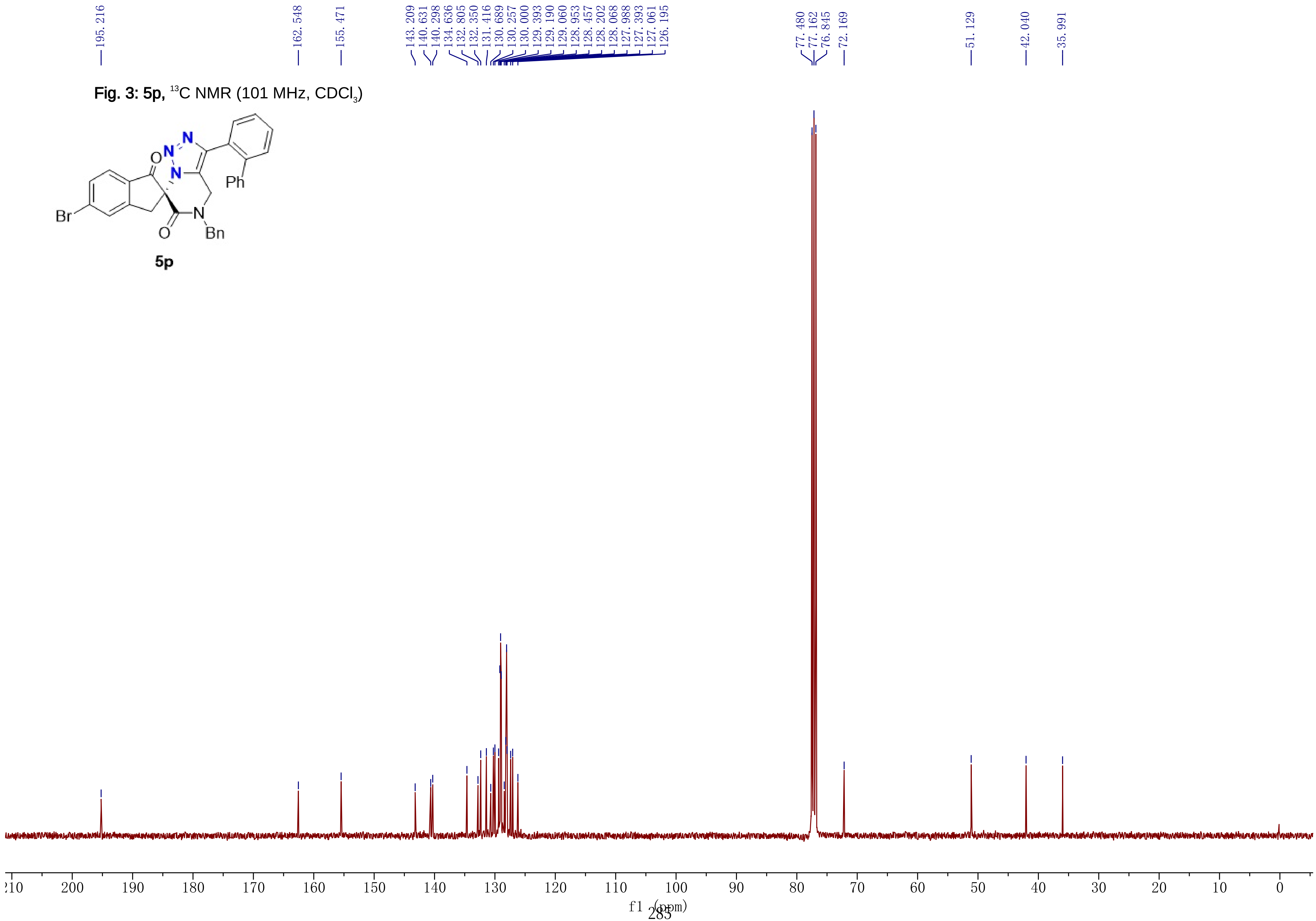

Fig. 3: 5p, ^{13}C NMR (101 MHz, $CDCl_3$)

5p

Fig. 3: 5q, ^{1}H NMR (400 MHz, $CDCl_3$)

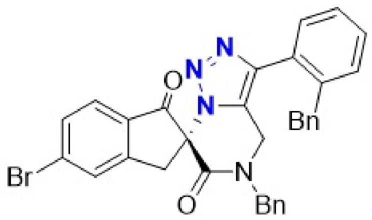

5q

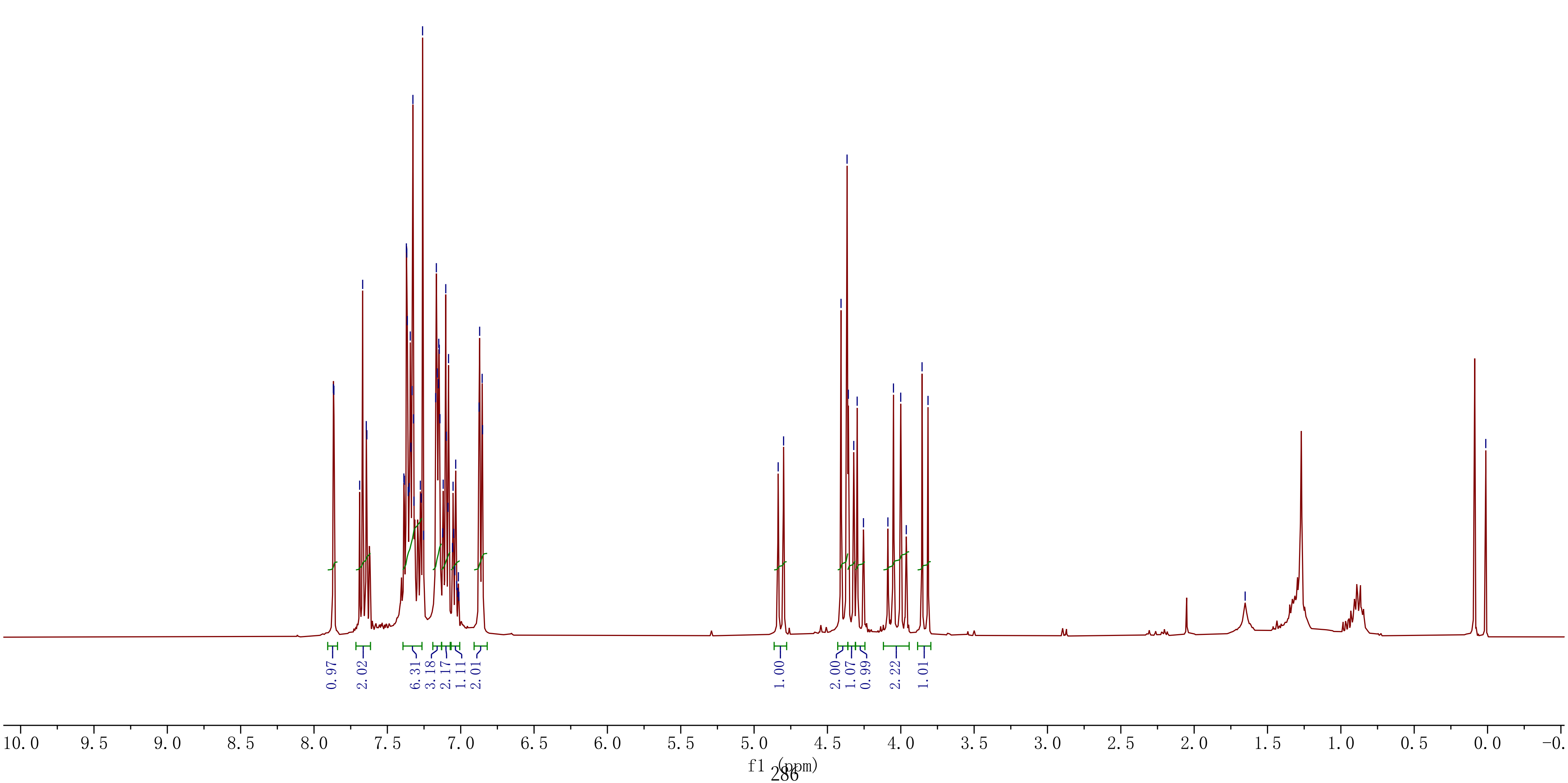

Fig. 3: 5q, ^{13}C NMR (101 MHz, $CDCl_3$)

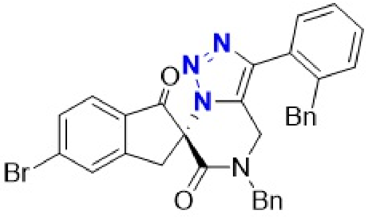

5q

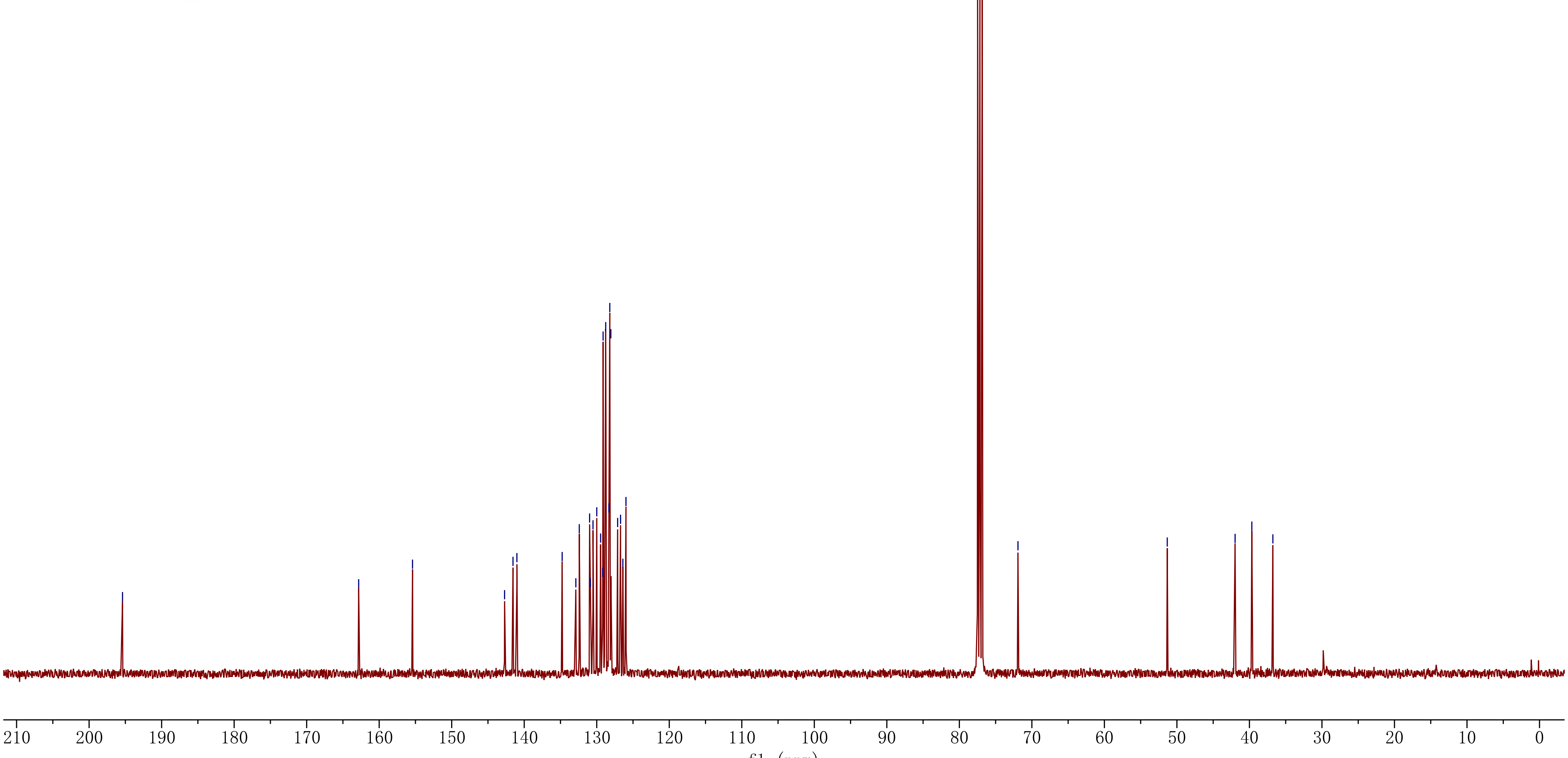

Fig. 3: 5r, ^{1}H NMR (400 MHz, $CDCl_3$)

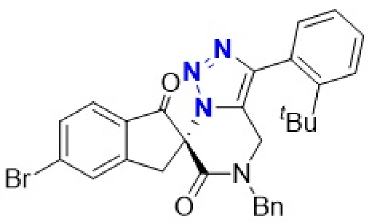

5r

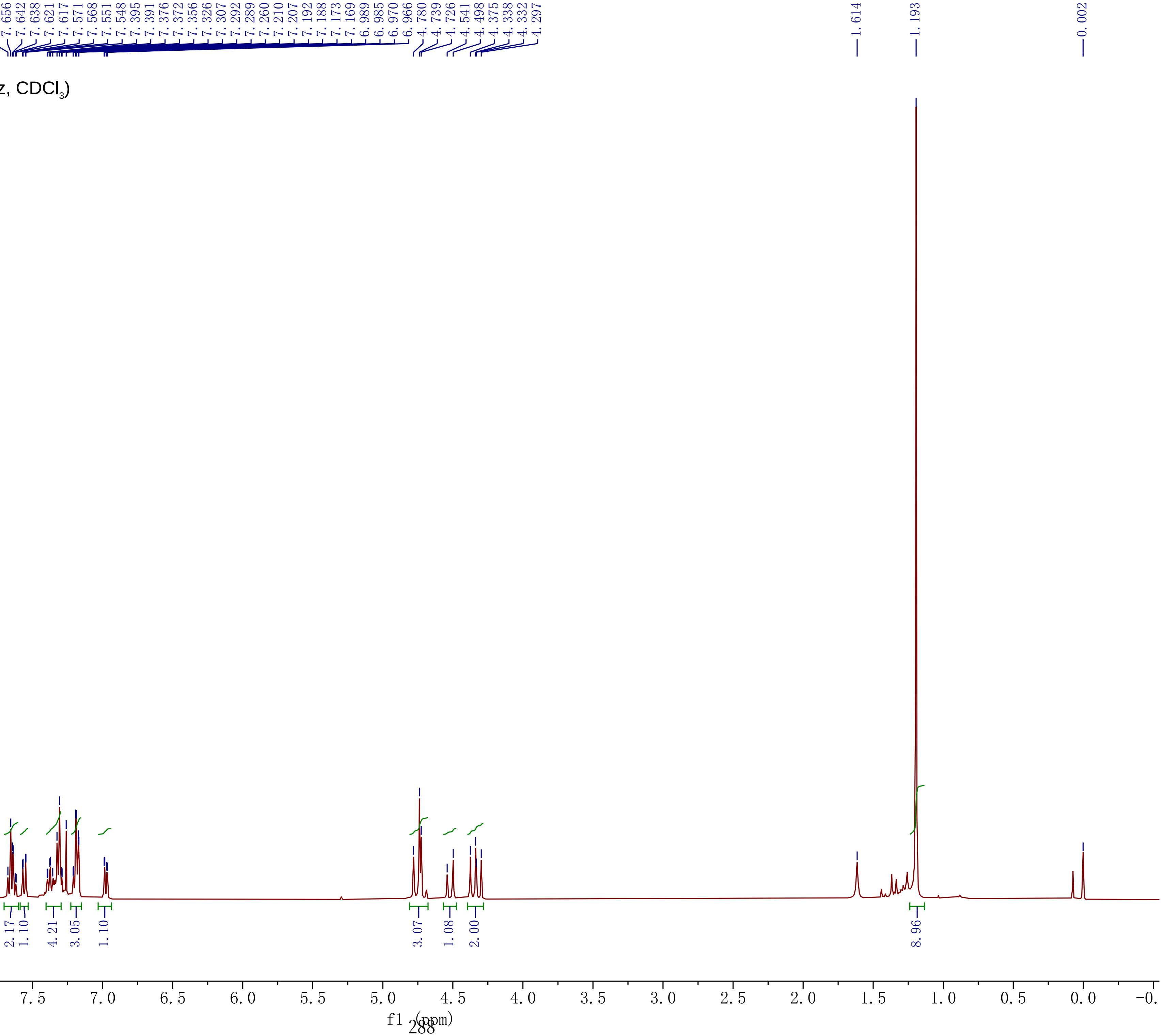

Fig. 3: 5r, ^{13}C NMR (101 MHz, $CDCl_3$)

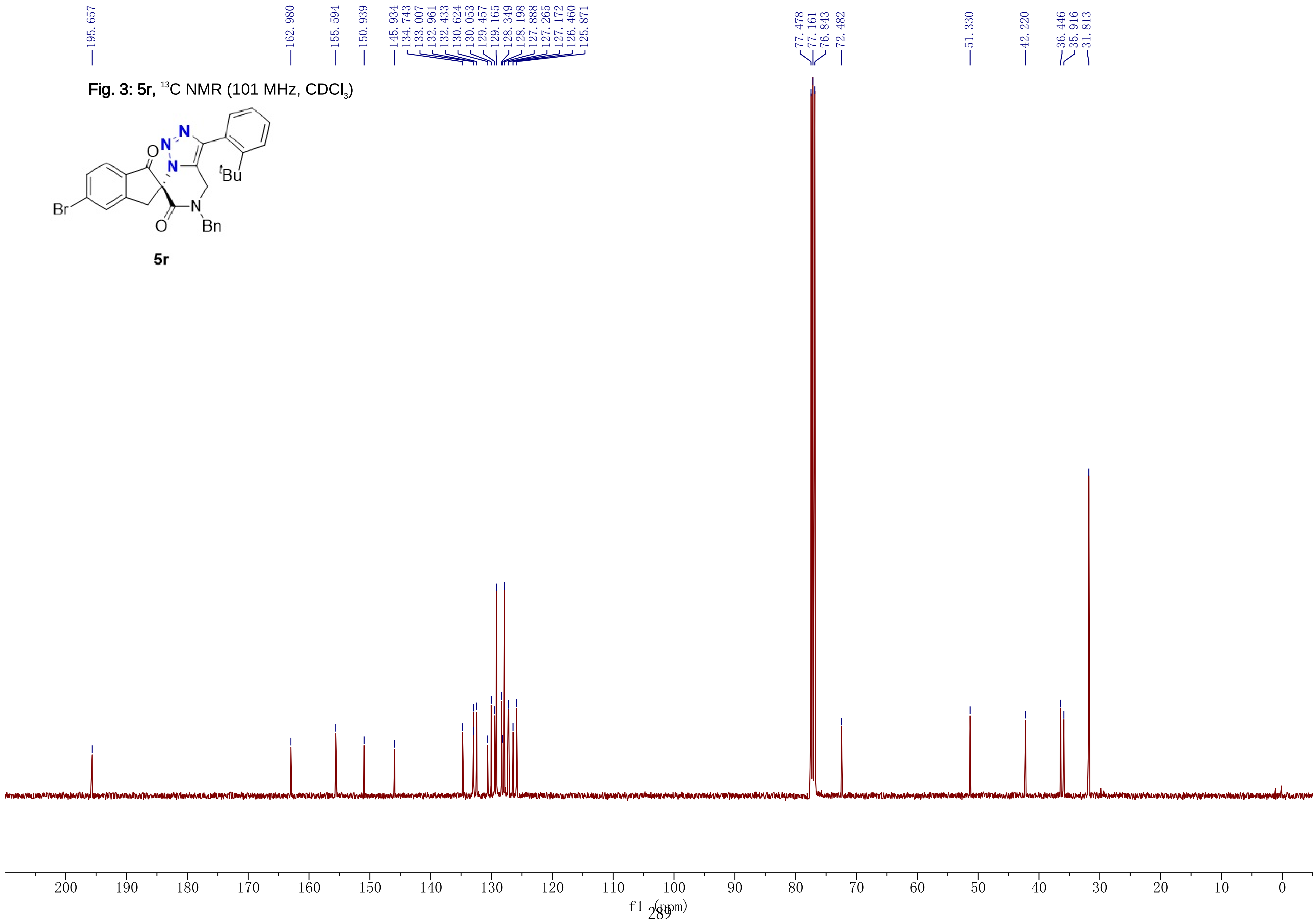

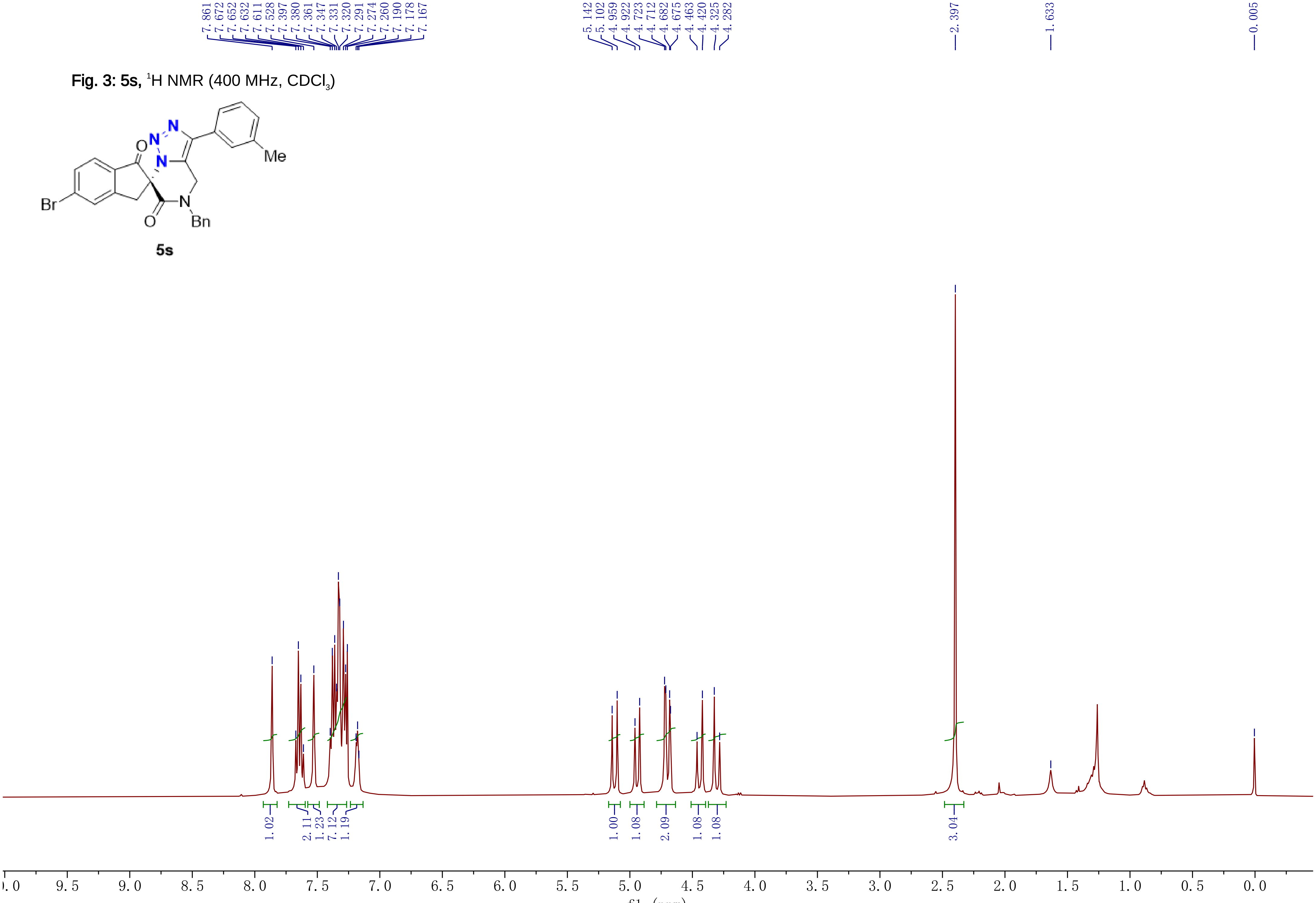

Fig. 3: 5s, ^{1}H NMR (400 MHz, $CDCl_3$)

5s

Fig. 3: 5s, ^{13}C NMR (101 MHz, $CDCl_3$)

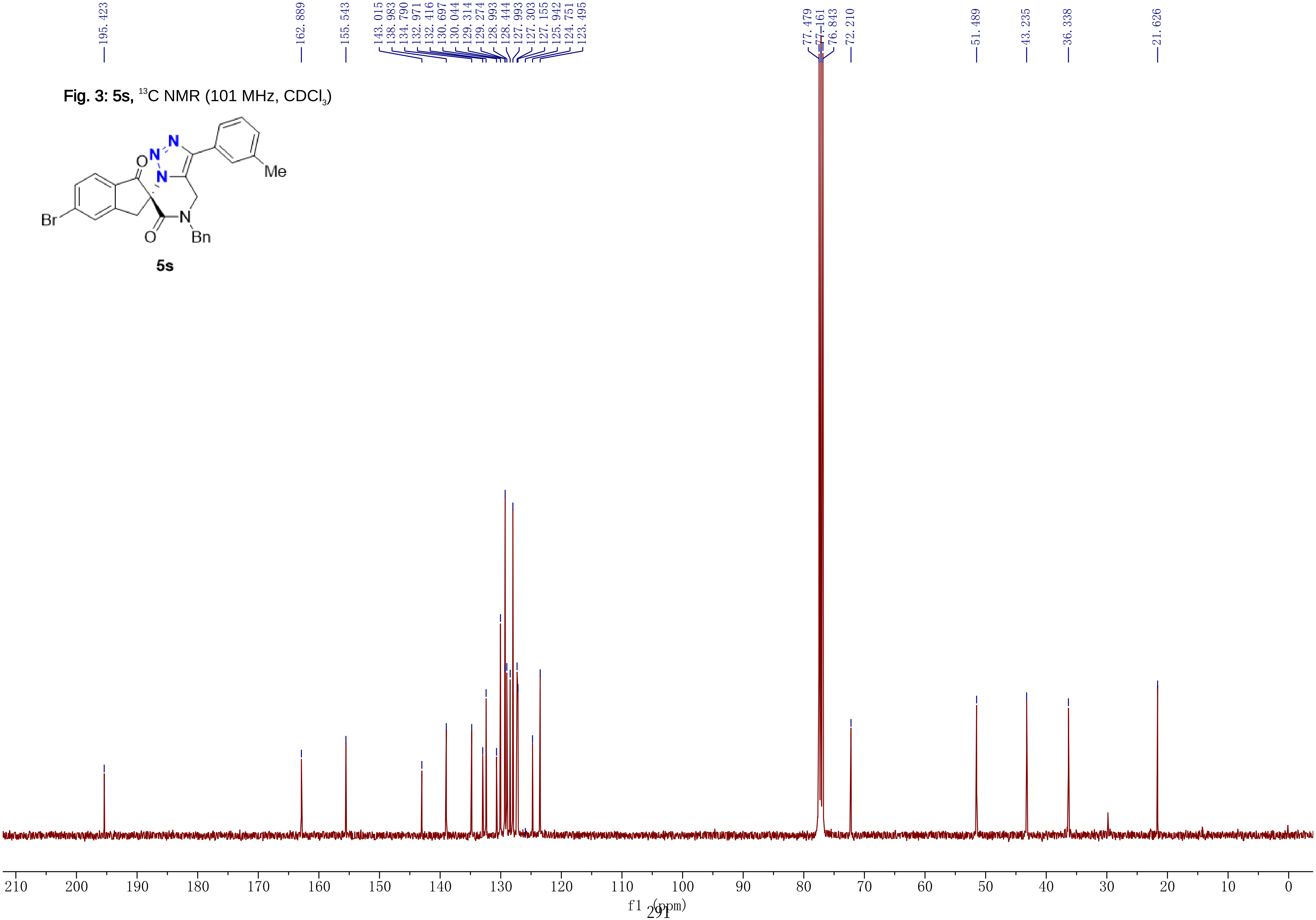

Fig. 3: 5t, ^{1}H NMR (400 MHz, $CDCl_3$)

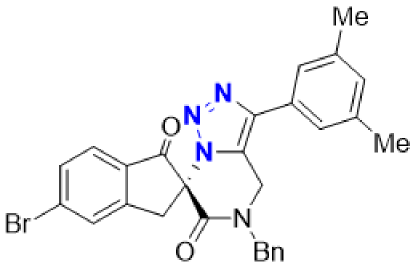

5t

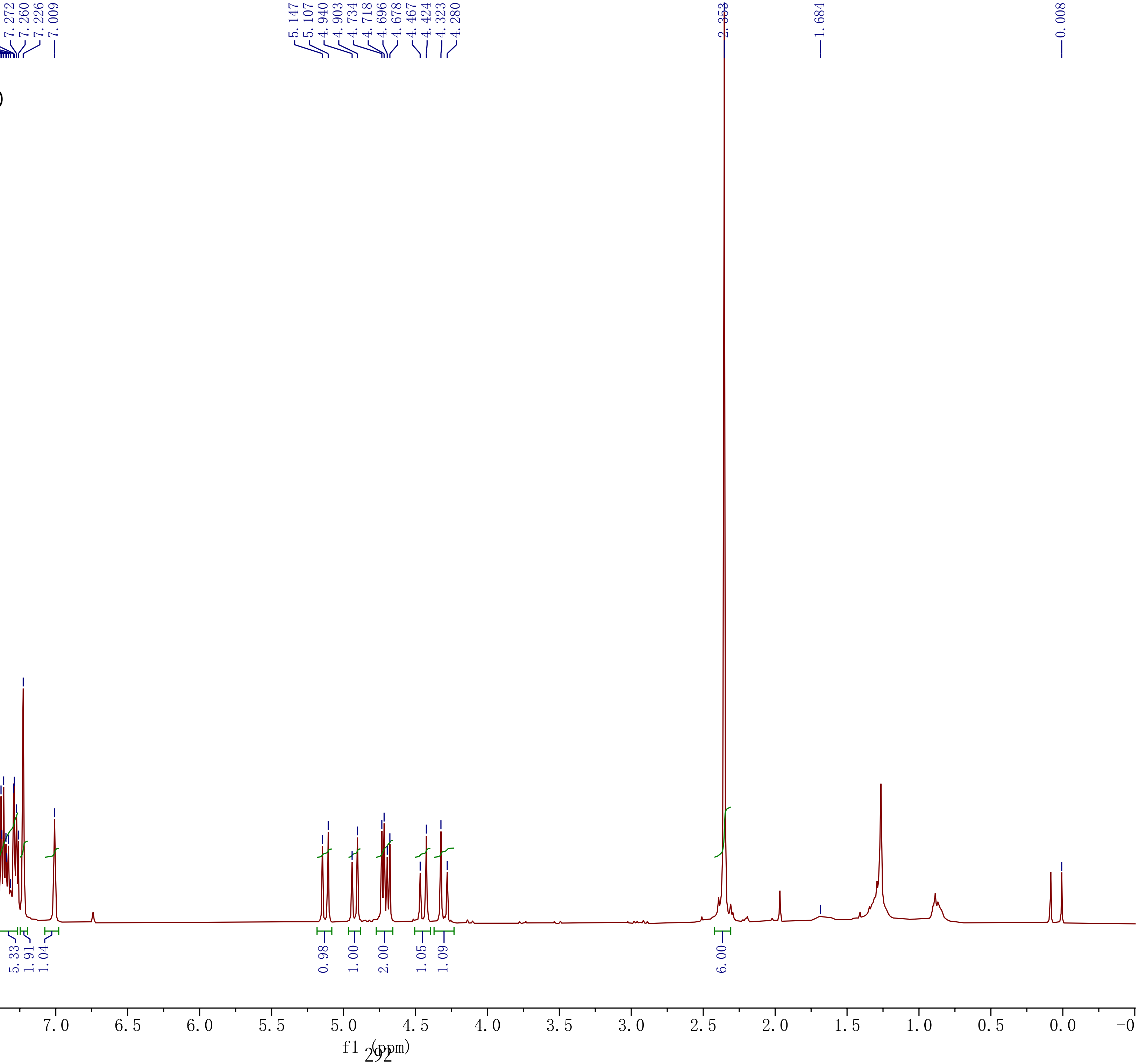

Fig. 3: 5t, ^{13}C NMR (101 MHz, $CDCl_3$)

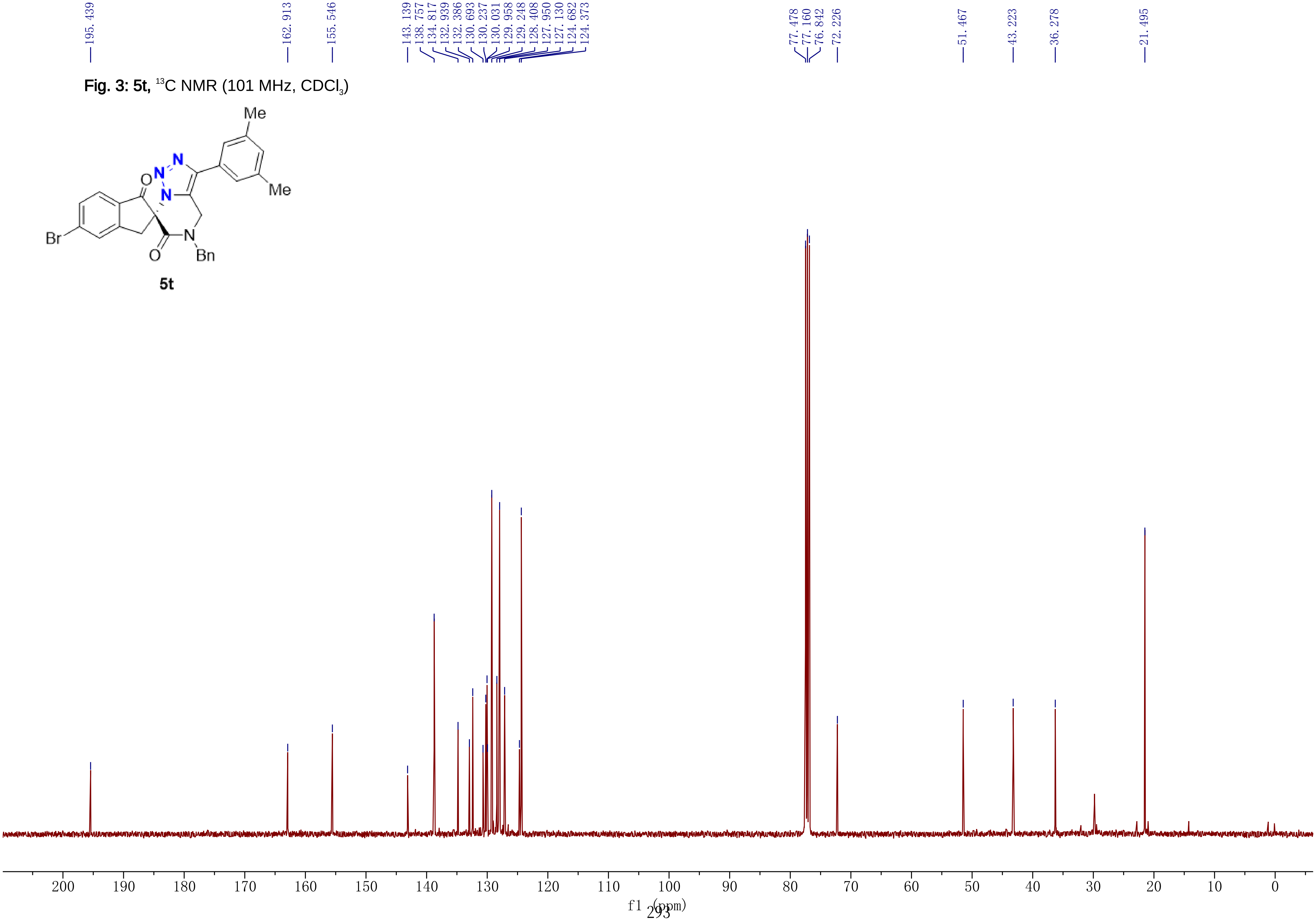

Fig. 3: 5u, ^{1}H NMR (400 MHz, $CDCl_3$)

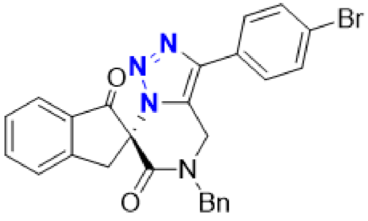

5u

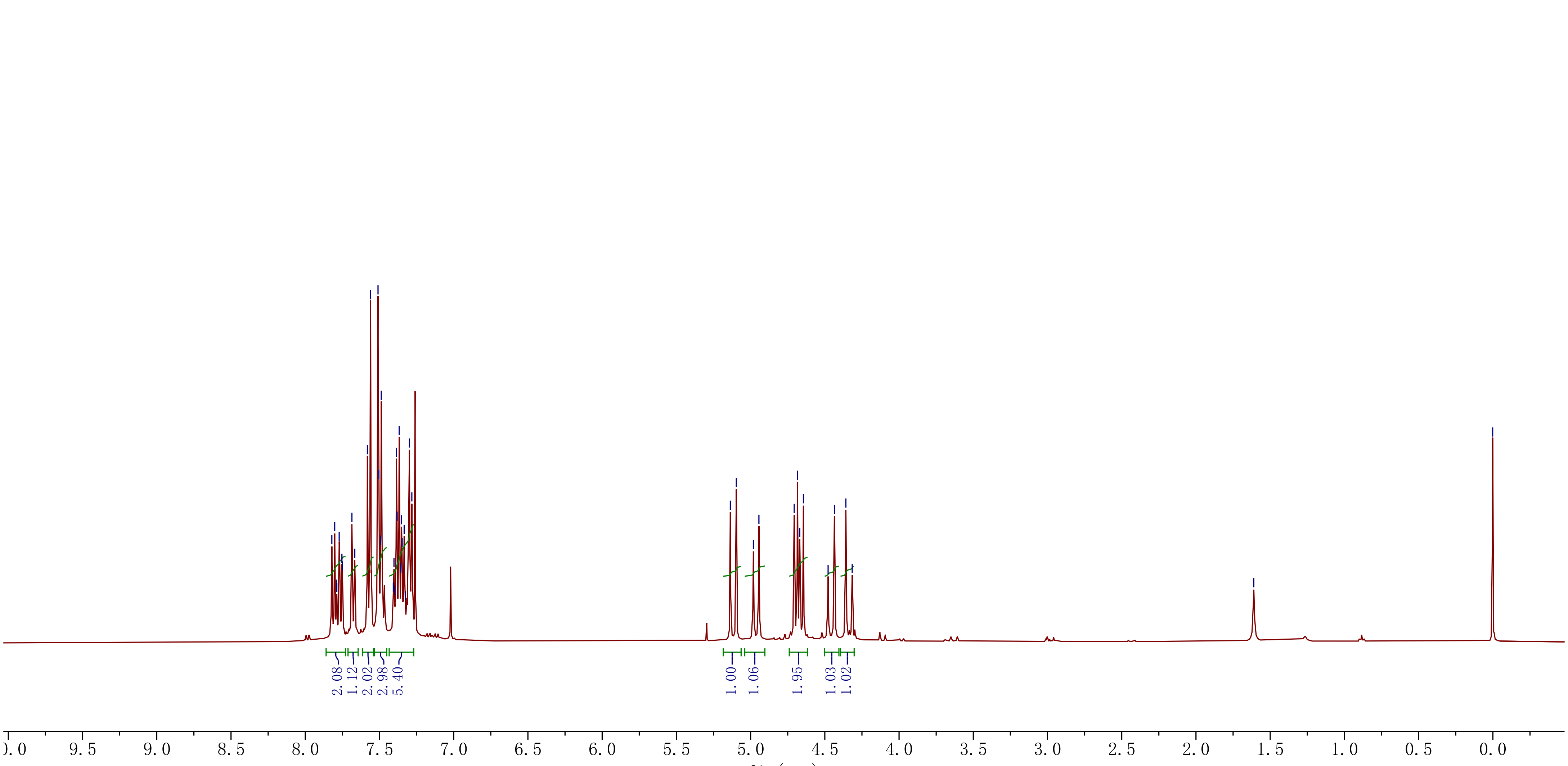

Fig. 3: 5u, ^{13}C NMR (101 MHz, $CDCl_3$)

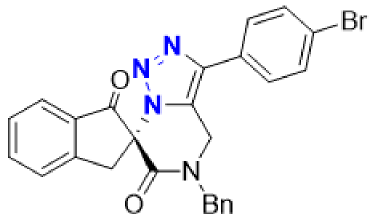

5u

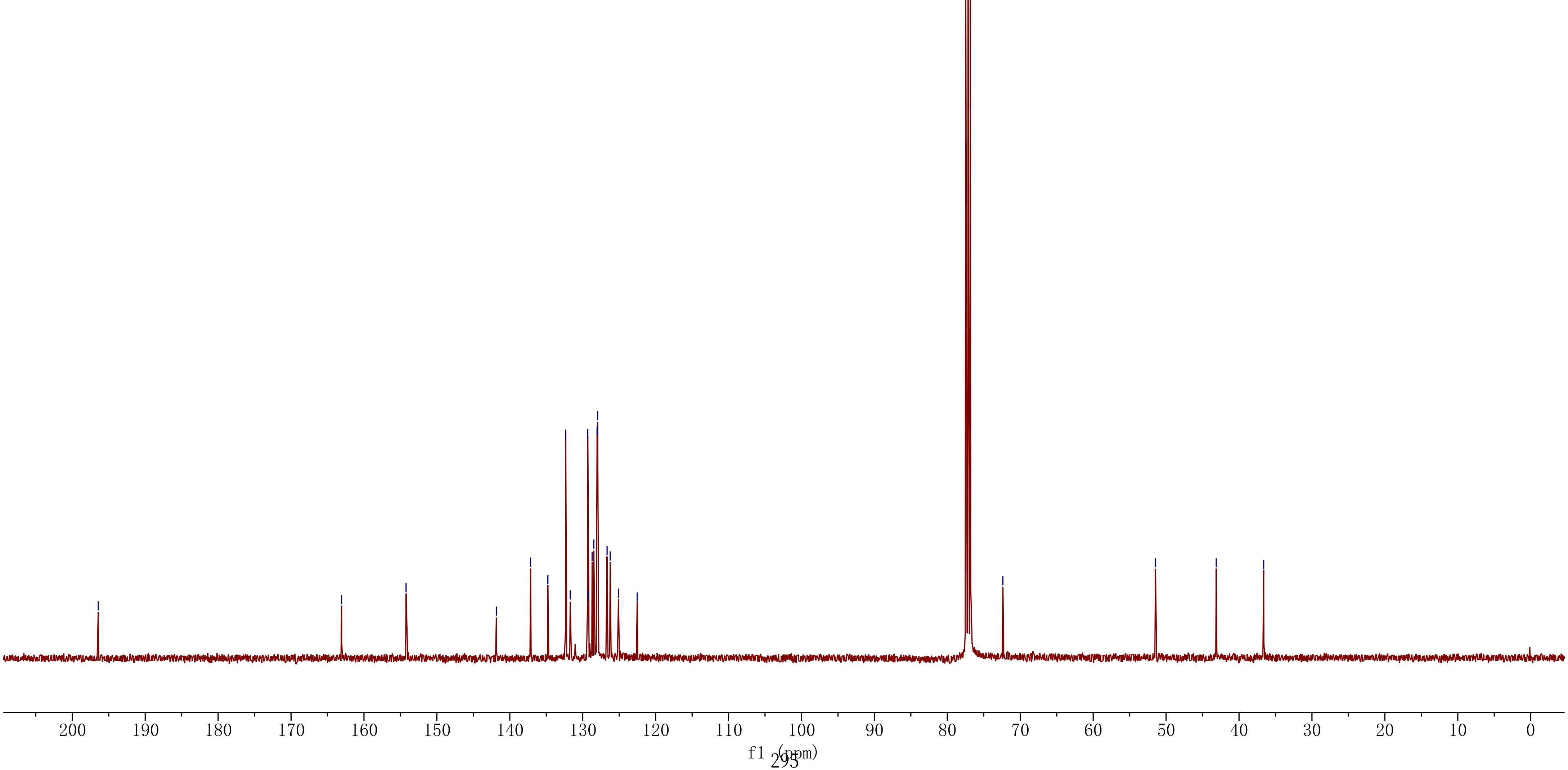

Fig. 3: 5v, ^{1}H NMR (400 MHz, $CDCl_3$)

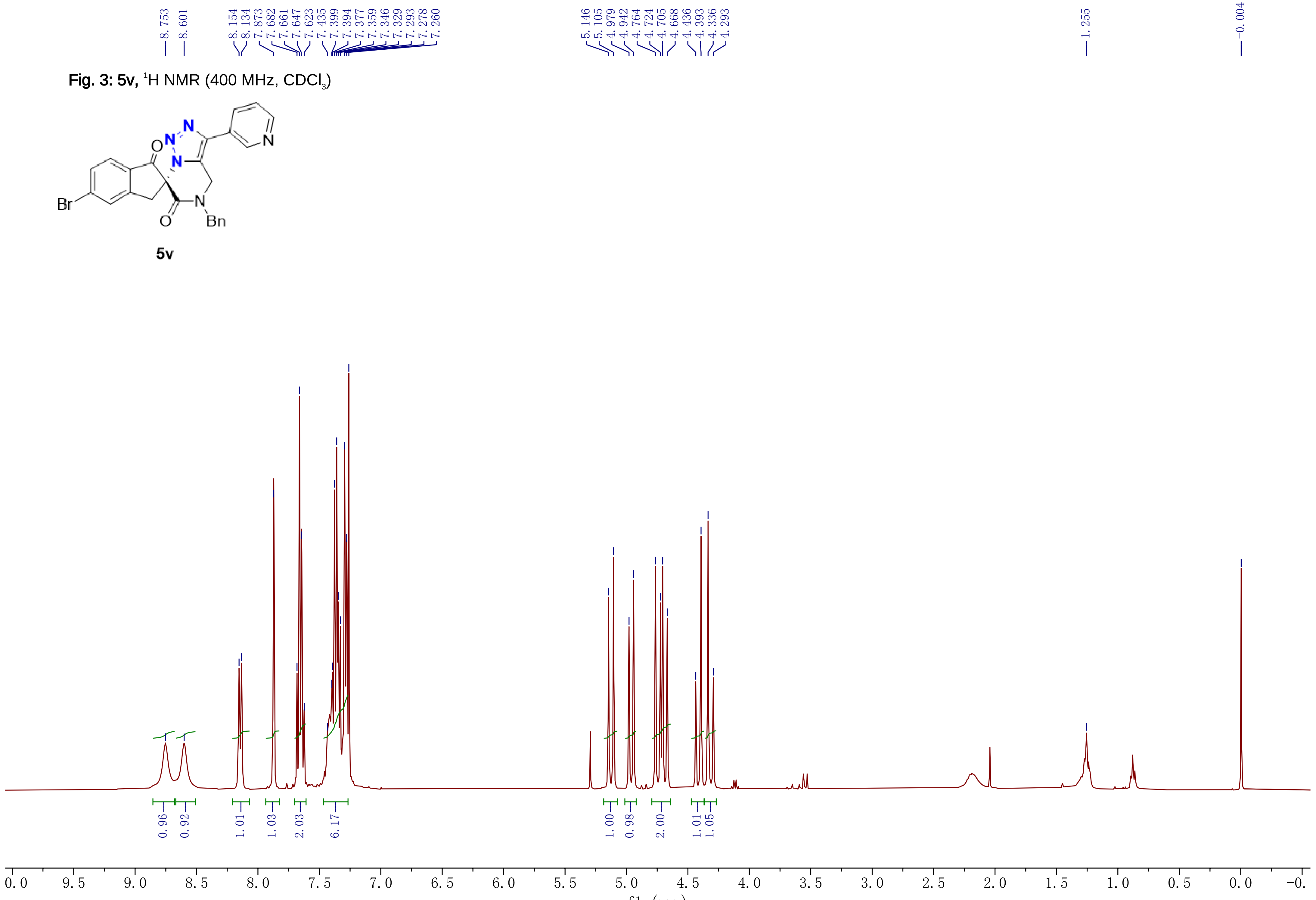

5v

Fig. 3: 5v, ^{13}C NMR (101 MHz, $CDCl_3$)

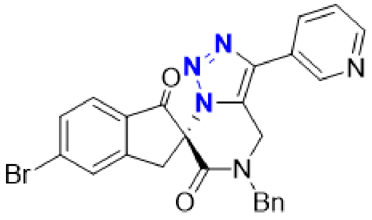

5v

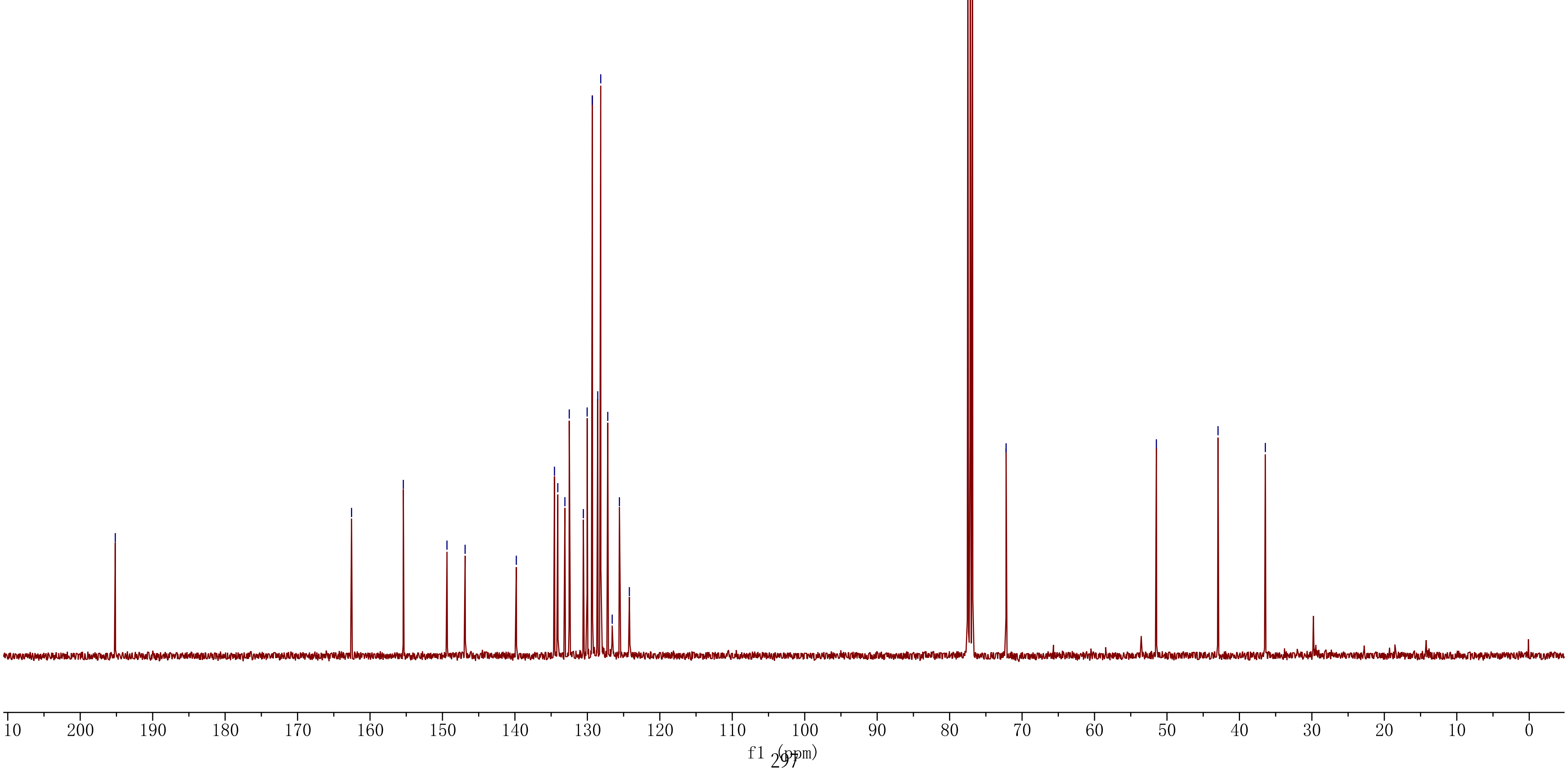

Fig. 3: 5w, ^{1}H NMR (400 MHz, $CDCl_3$)

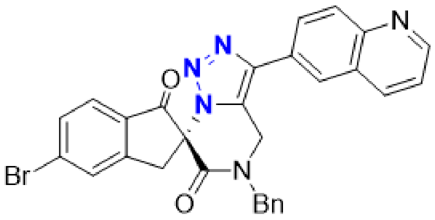

5w

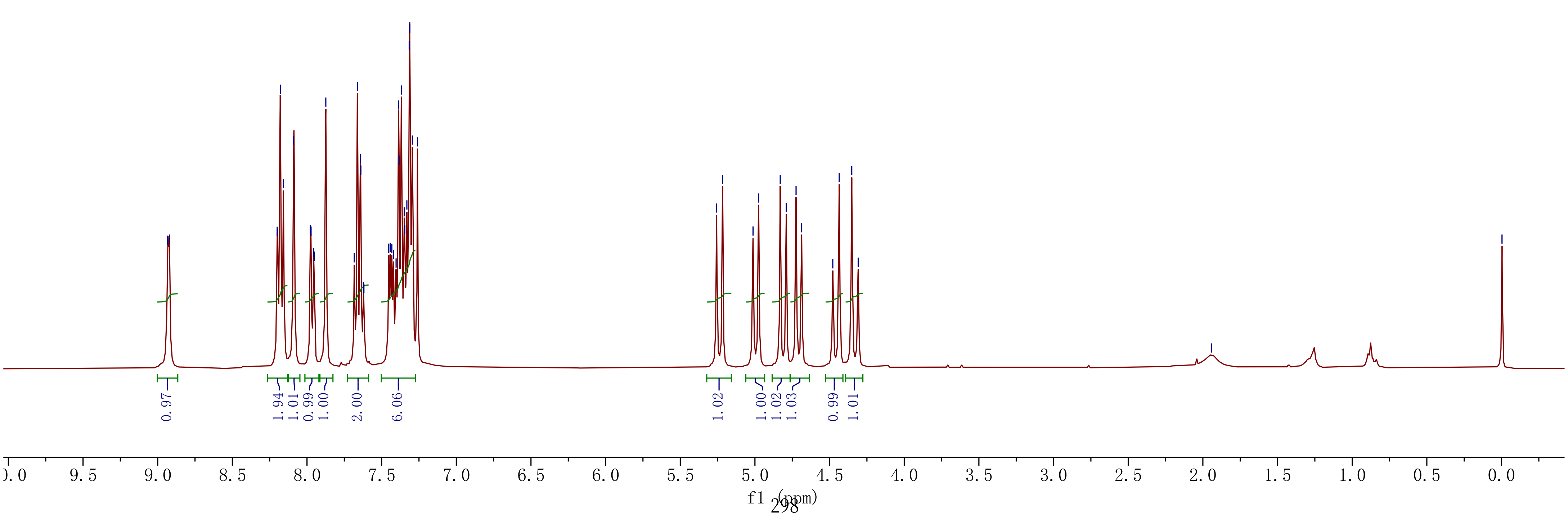

Fig. 3: 5w, ^{13}C NMR (101 MHz, $CDCl_3$)

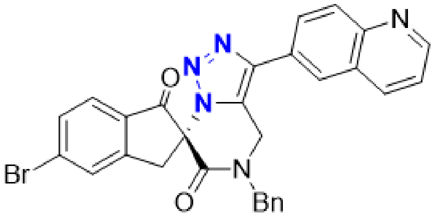

5w

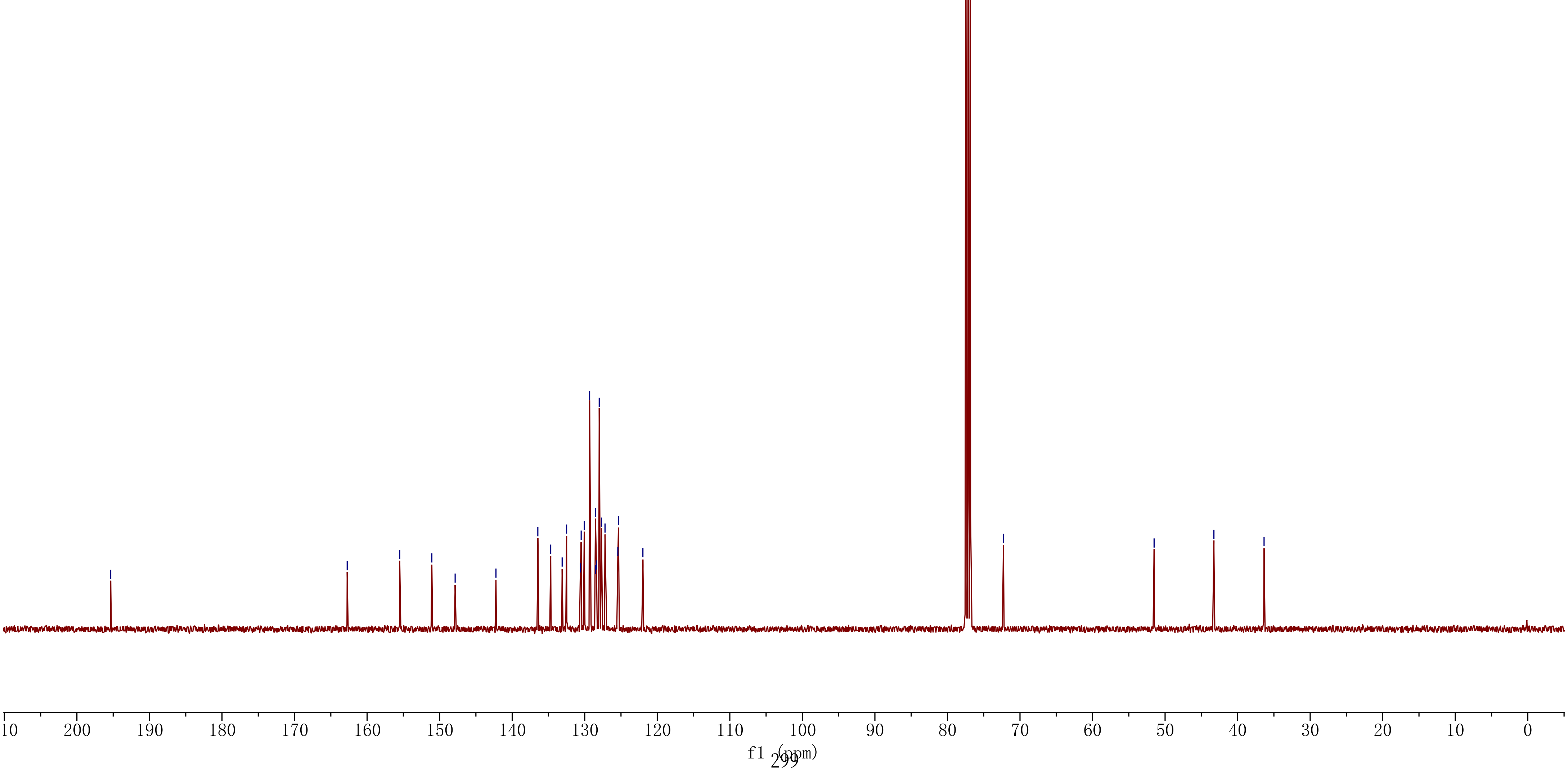

Fig. 3: 5x, ^{1}H NMR (400 MHz, $CDCl_3$)

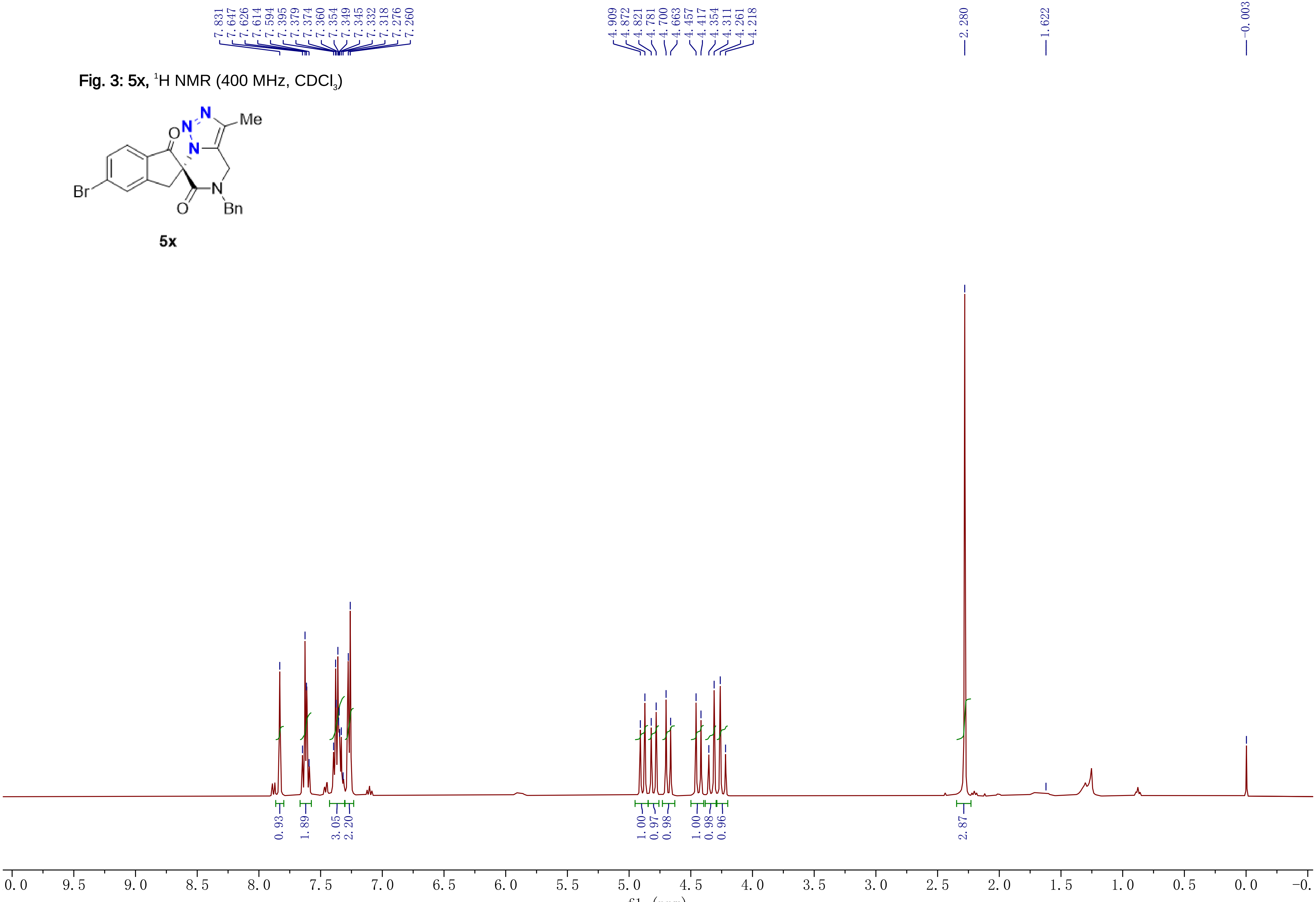

Fig. 3: 5x, ^{13}C NMR (101 MHz, $CDCl_3$)

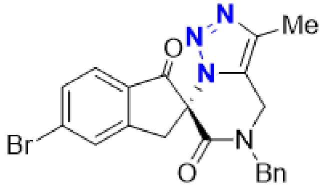

5x

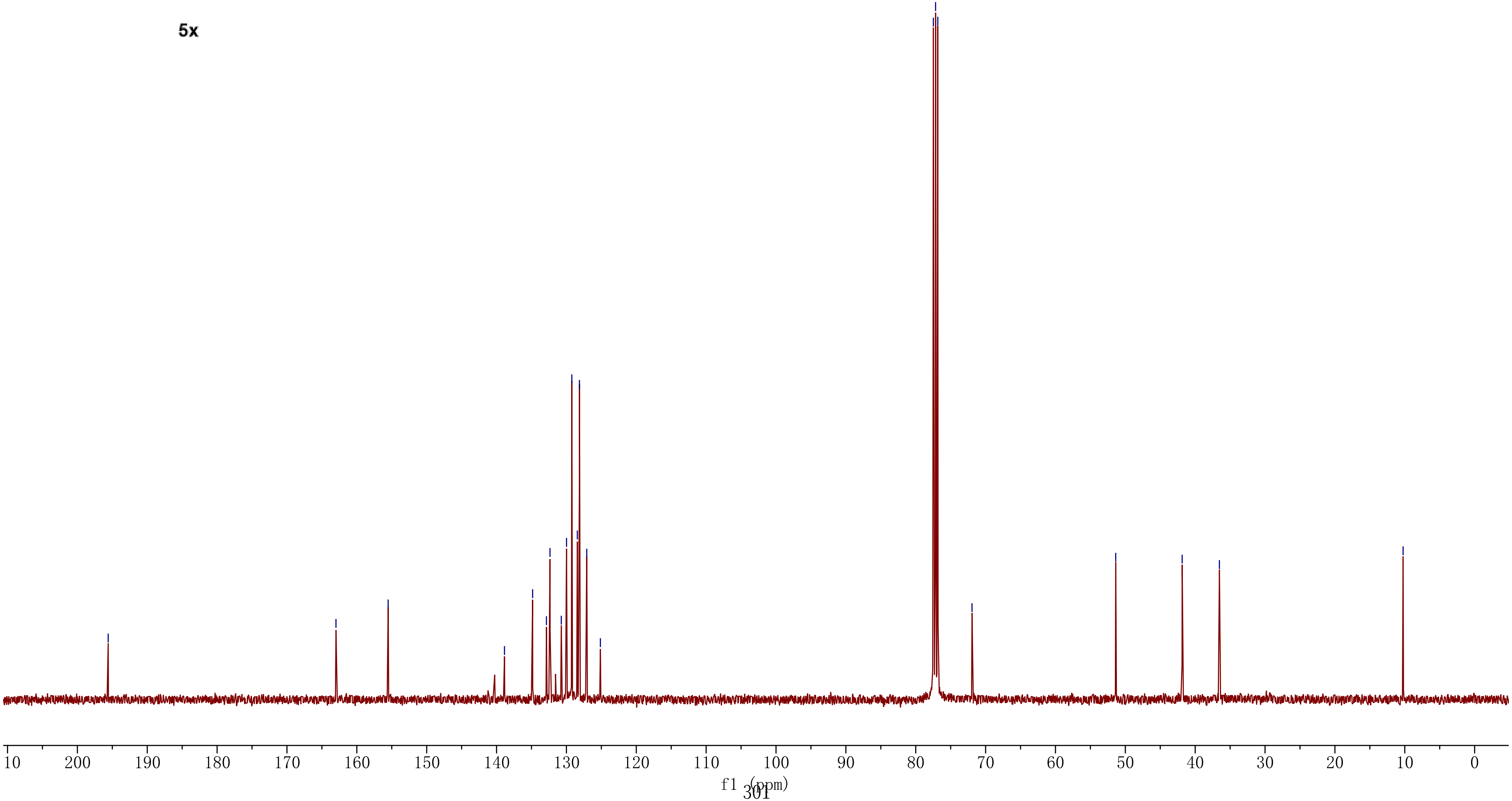

Fig. 3: 5y, ^{1}H NMR (400 MHz, $CDCl_3$)

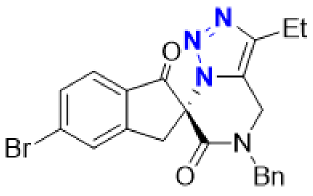

5y

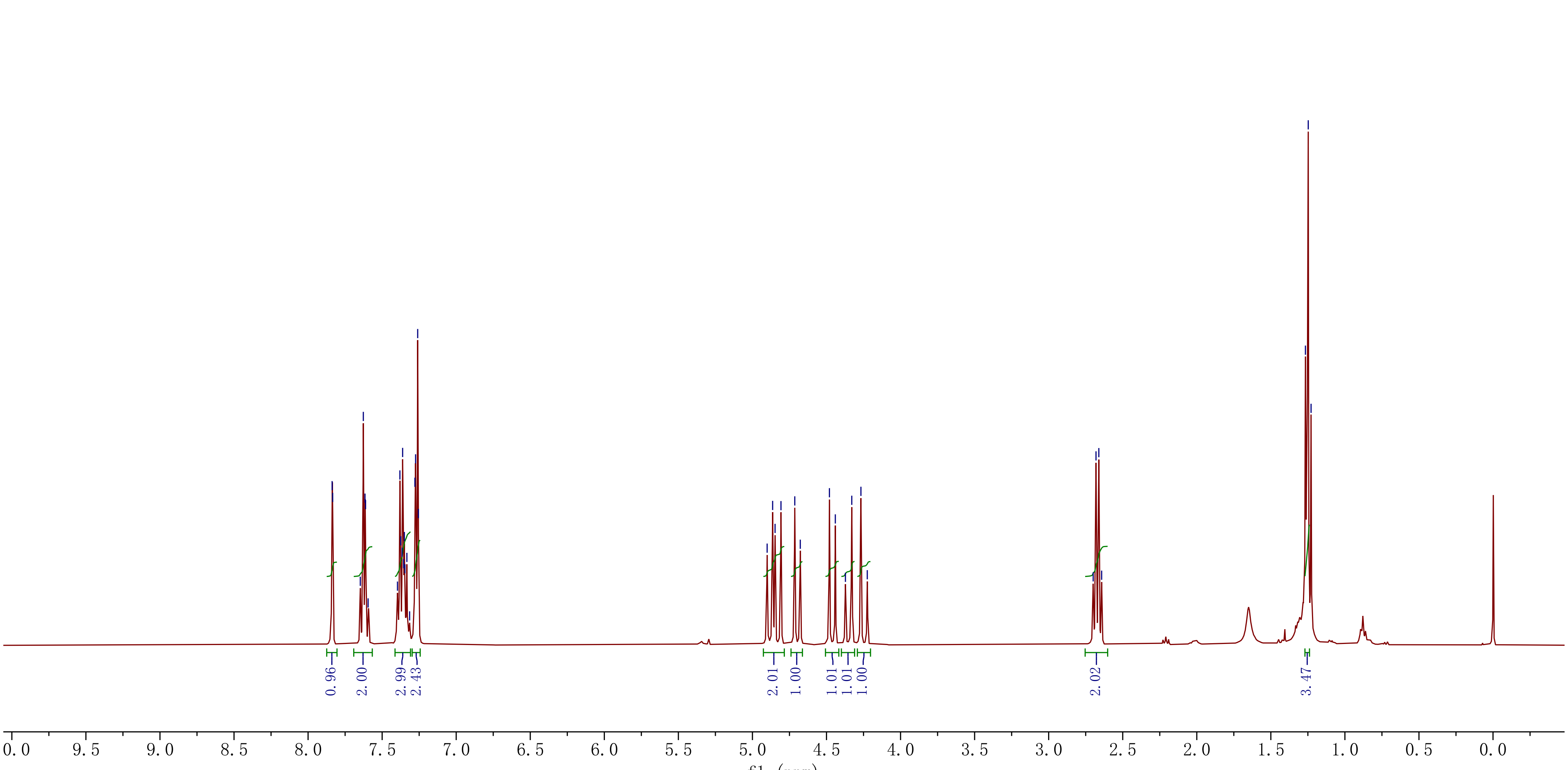

Fig. 3: 5y, ^{13}C NMR (101 MHz, $CDCl_3$)

5y

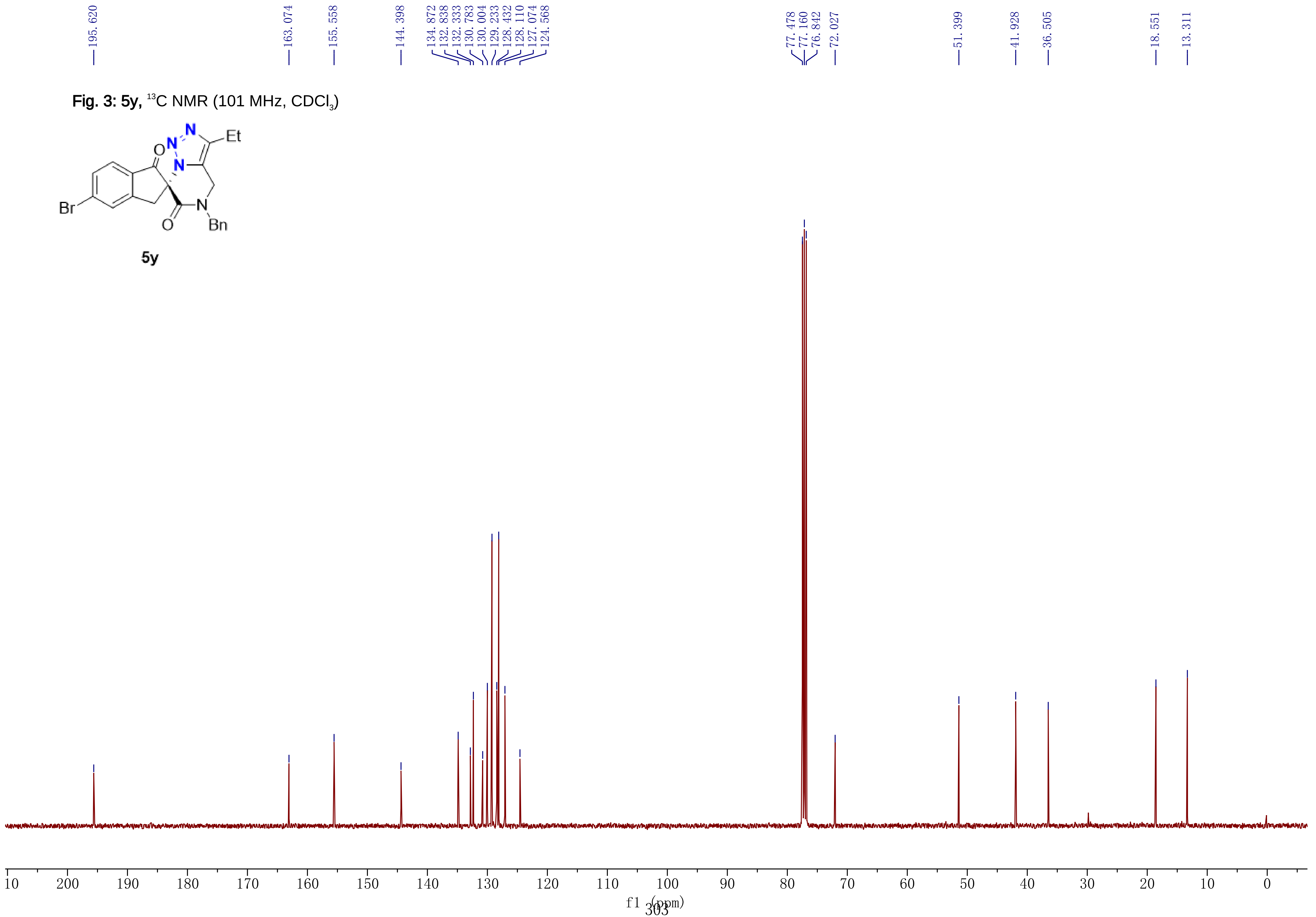

Fig. 3: 5z, ^{1}H NMR (400 MHz, $CDCl_3$)

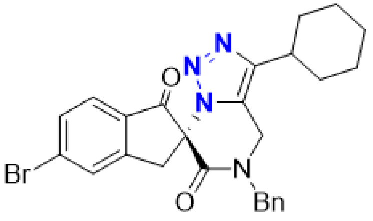

5z

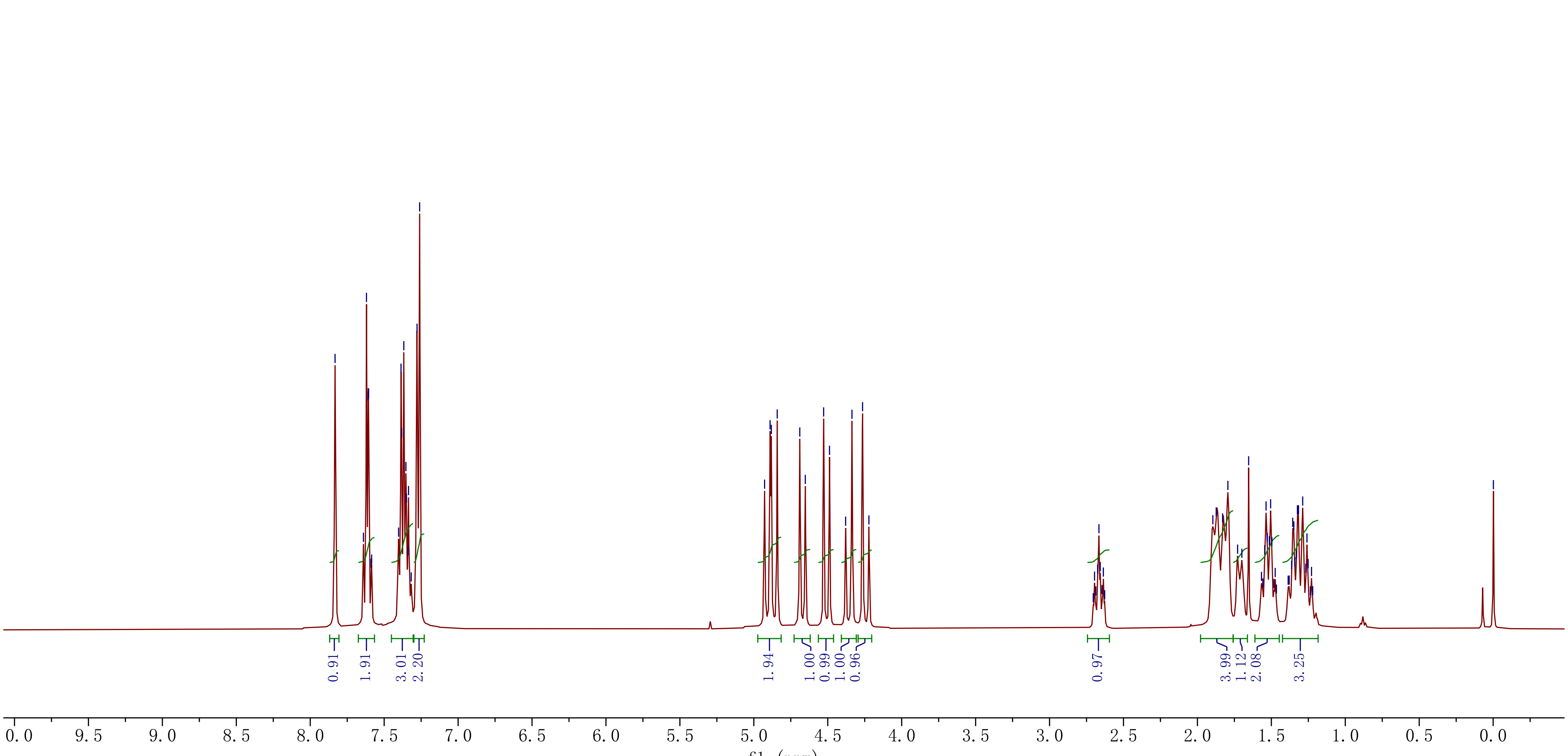

Fig. 3: 5z, ^{13}C NMR (101 MHz, $CDCl_3$)

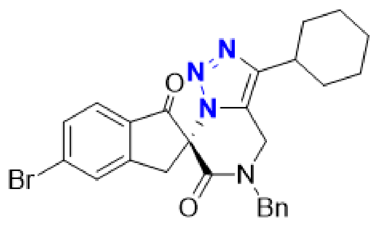

5z

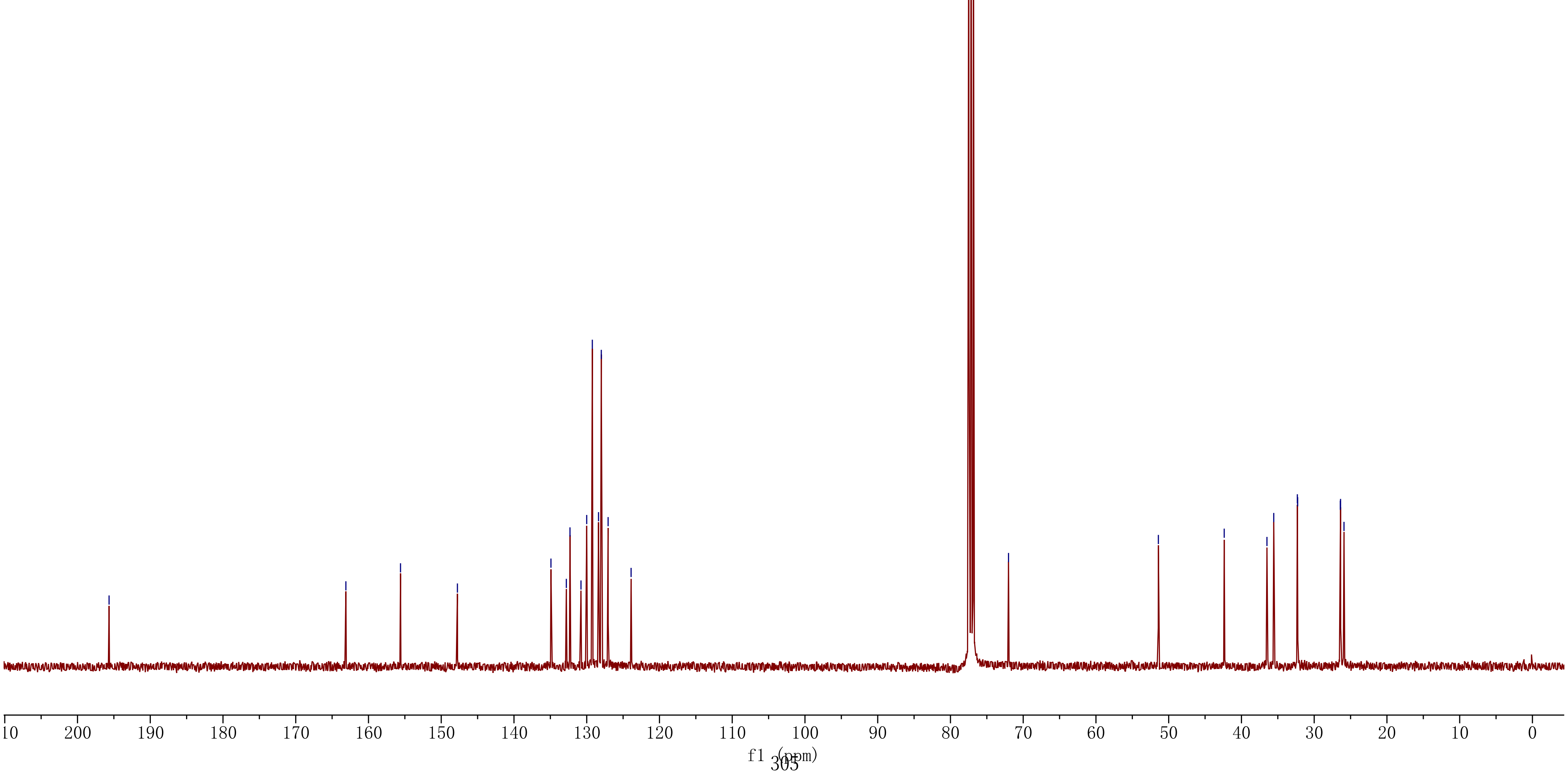

Fig. 4: 6, ^{1}H NMR (400 MHz, $CDCl_3$)

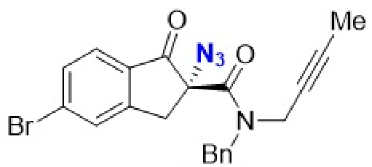

6

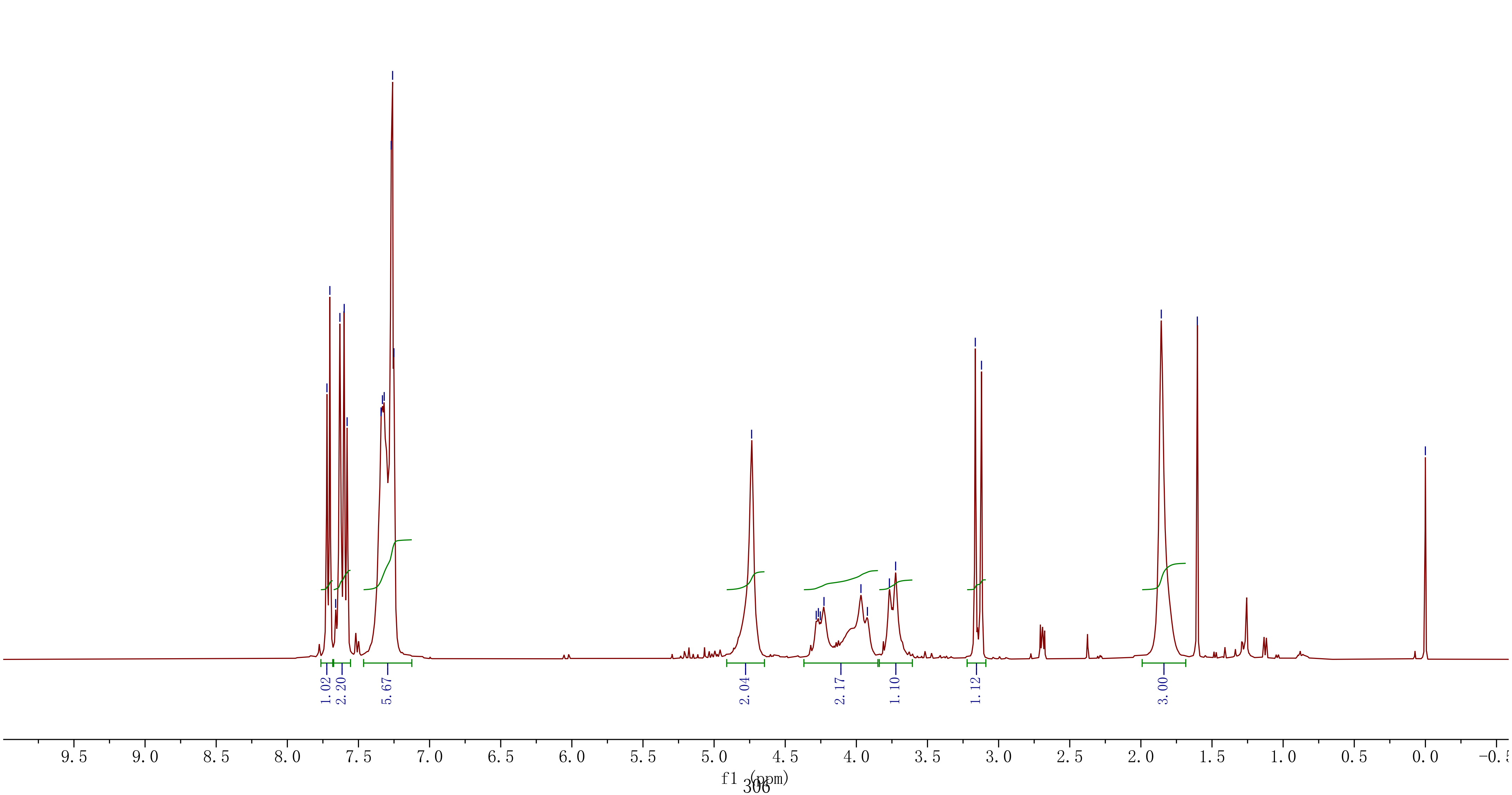

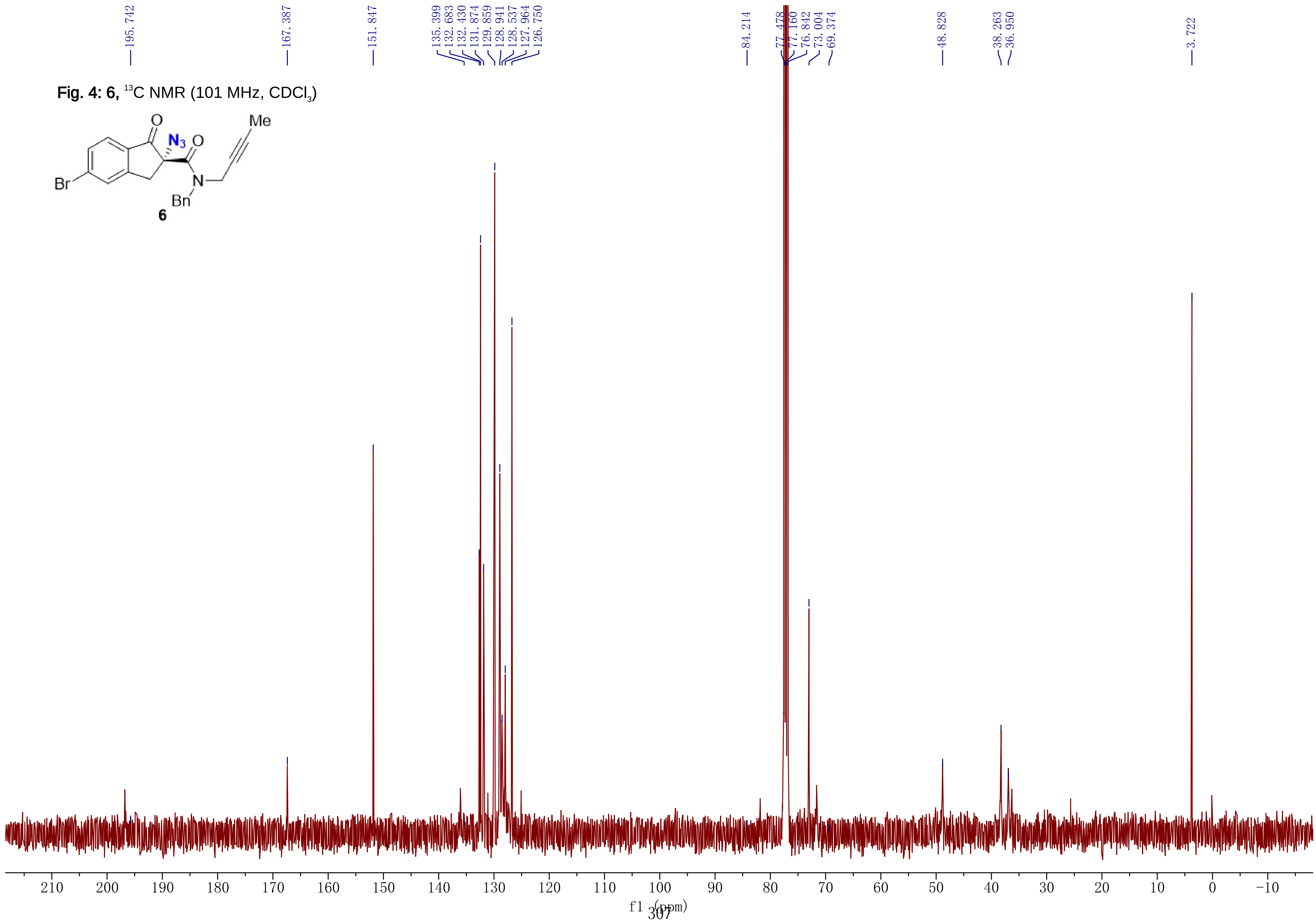

Fig. 4: 6, ^{13}C NMR (101 MHz, $CDCl_3$)

Fig. 4: 7c, ^{1}H NMR (400 MHz, $CDCl_3$)

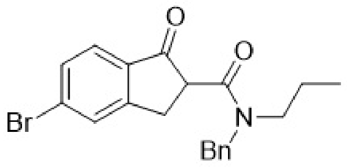

7c

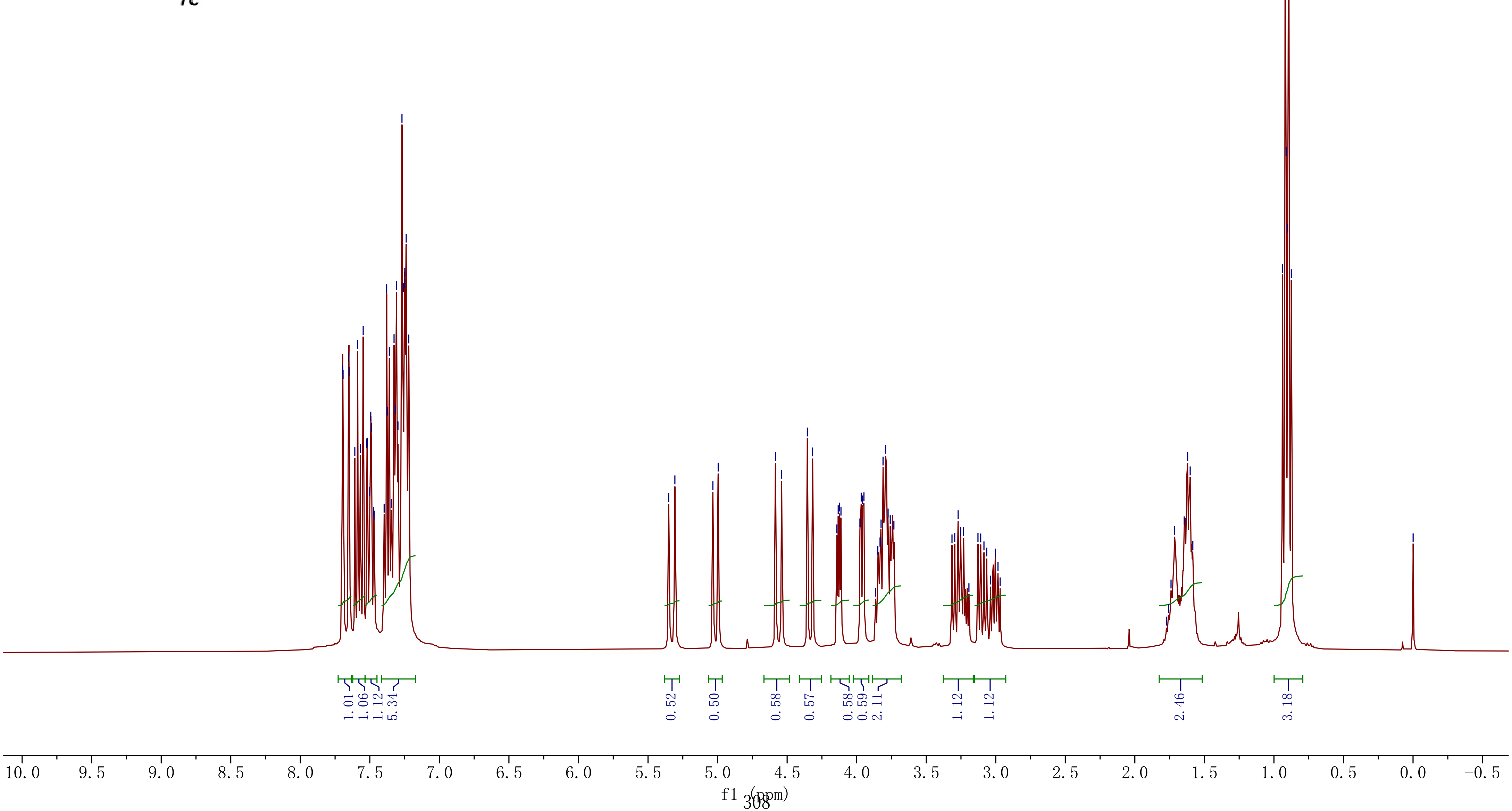

Fig. 4: 7c, ^{13}C NMR (101 MHz, $CDCl_3$)

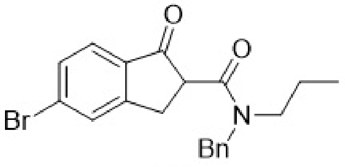

7c

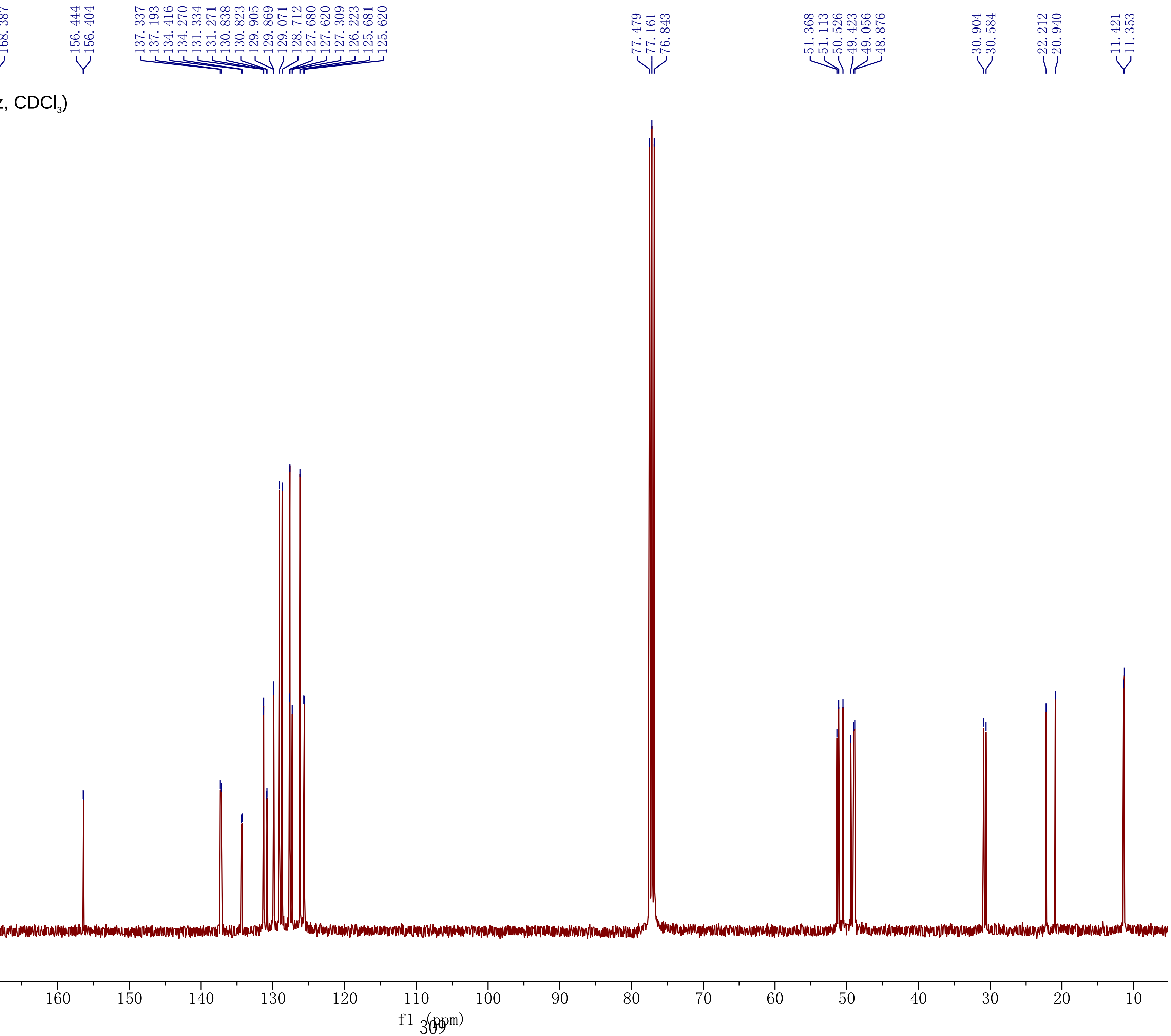

Fig. 4: 7d, ^{1}H NMR (400 MHz, $CDCl_3$)

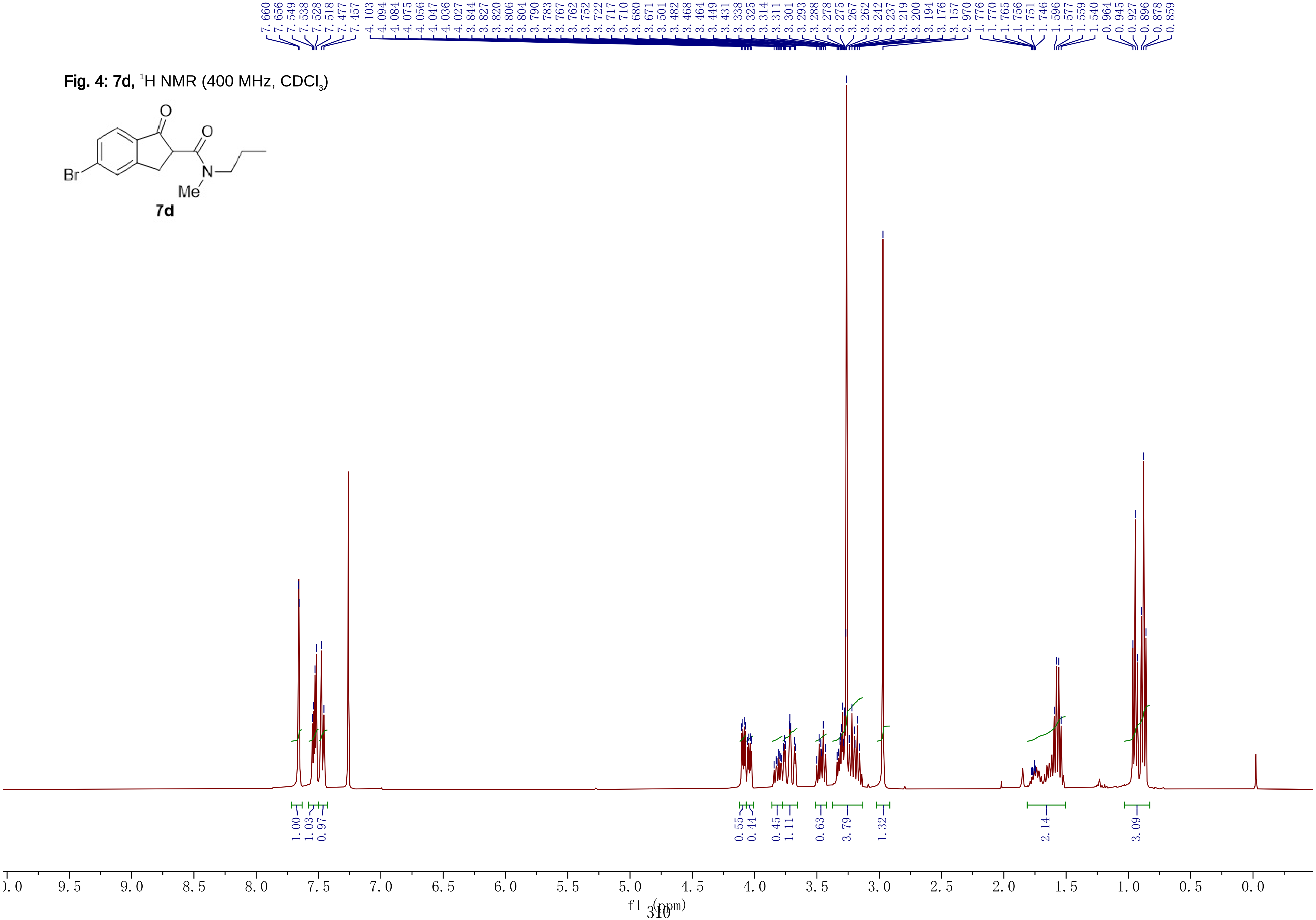

Fig. 4: 7d, ^{13}C NMR (101 MHz, $CDCl_3$)

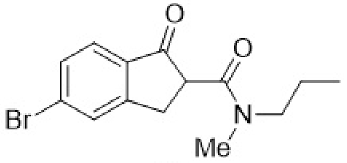

7d

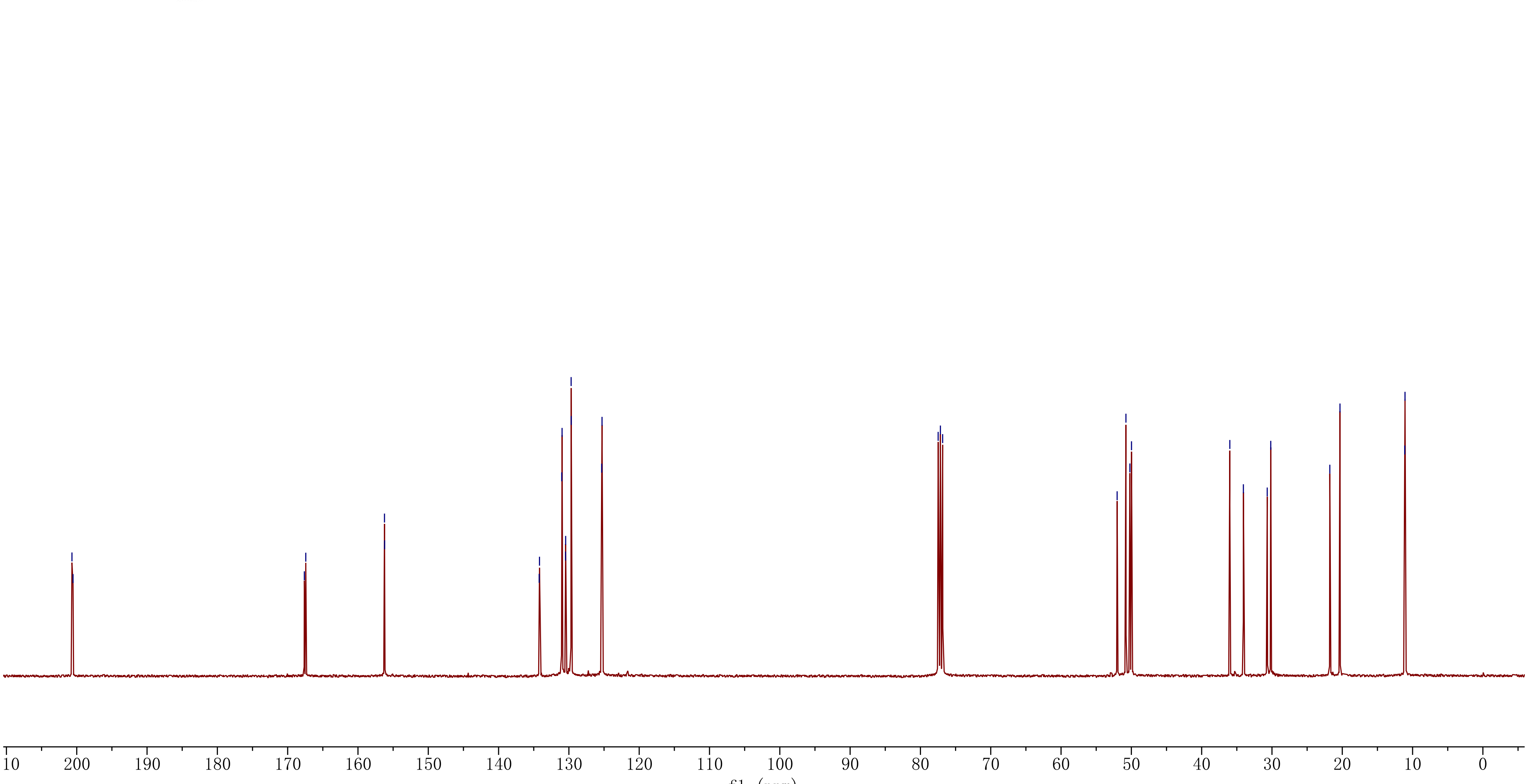

Fig. 4: 8b, ¹H NMR (400 MHz, $CDCl_3$)

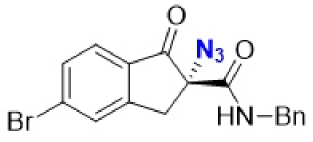

8b

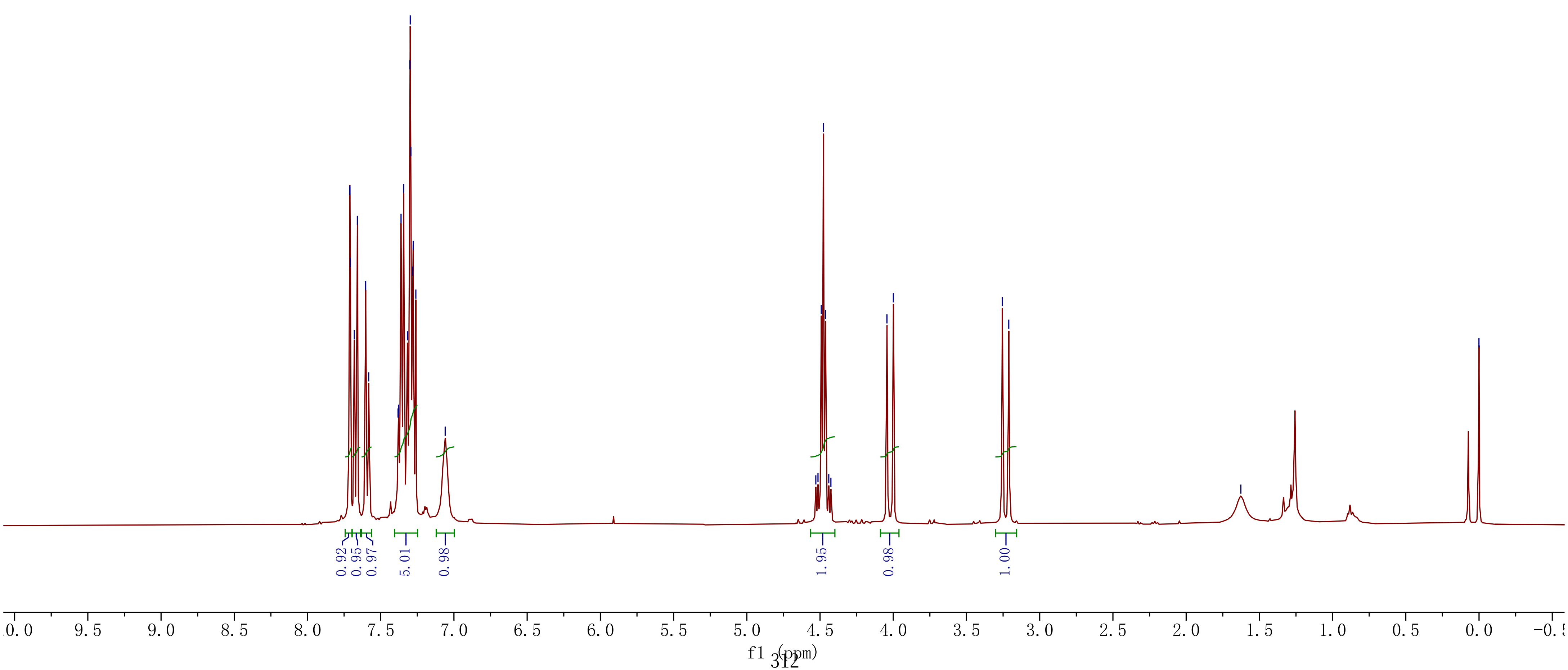

Fig. 4: **8b** ^{13}C NMR (101 MHz, $CDCl_3$)

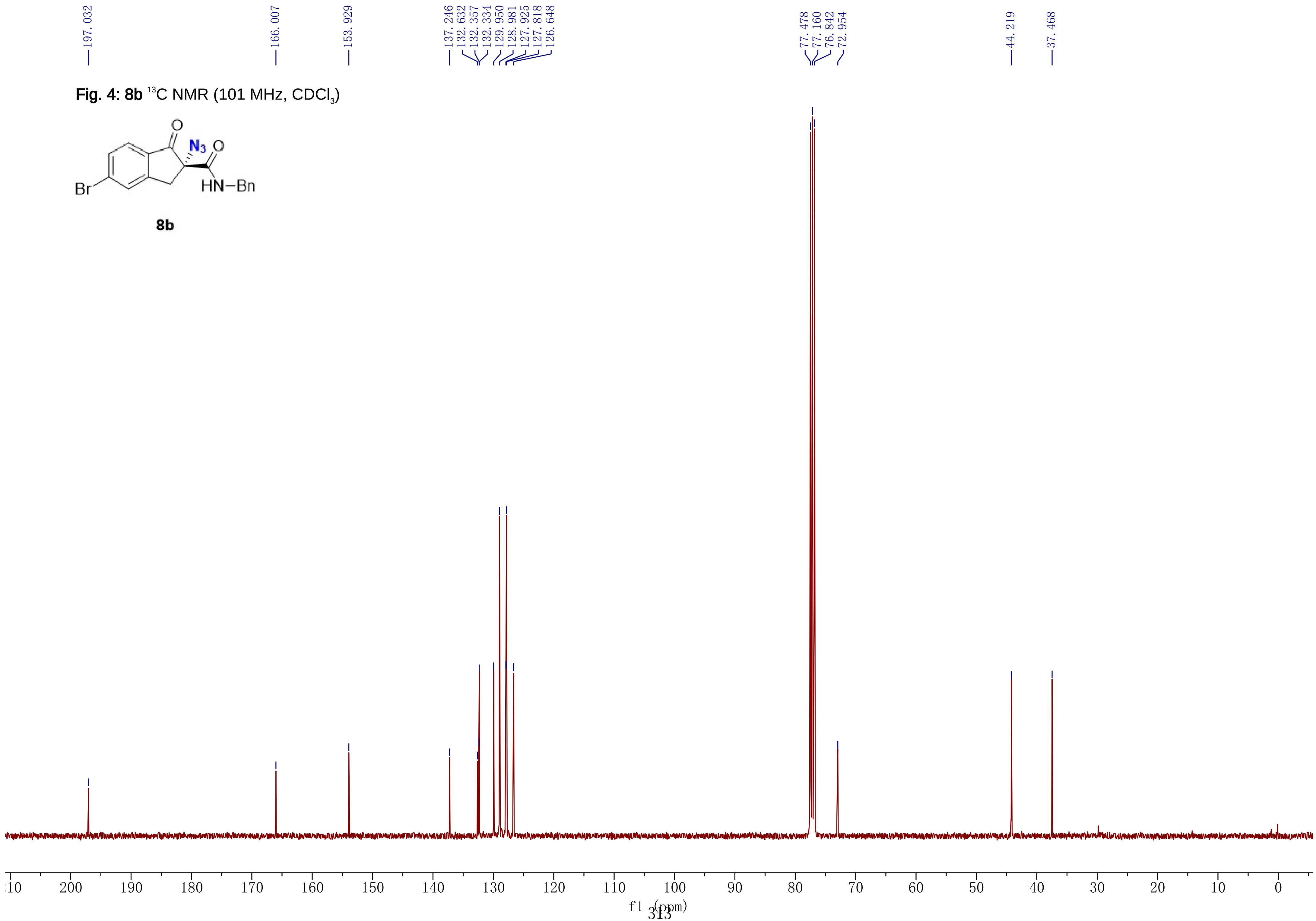

Fig. 4: 8c, ^{1}H NMR (400 MHz, $CDCl_3$)

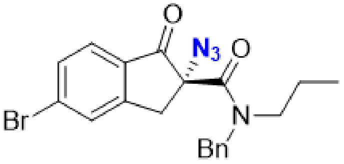

8c

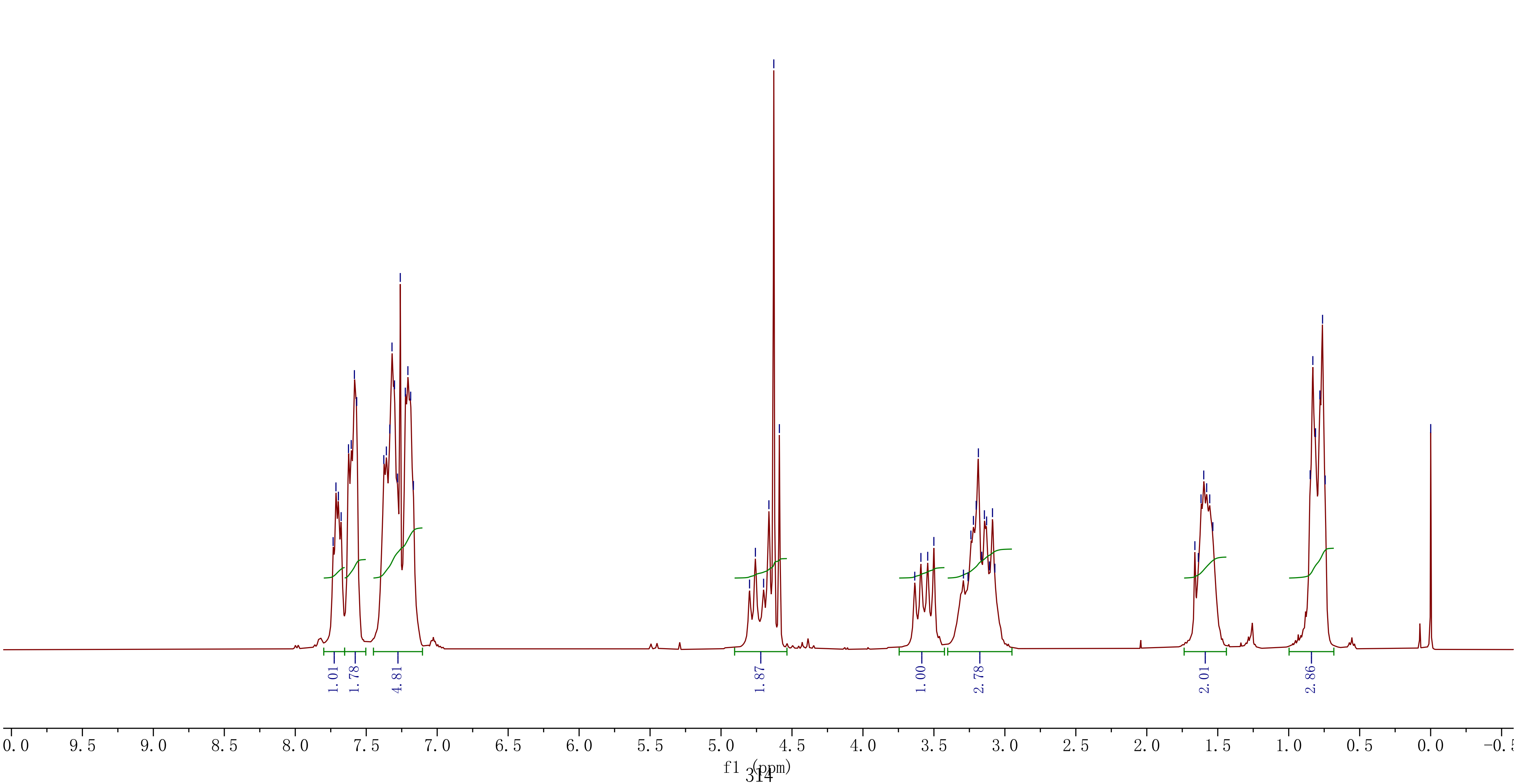

Fig. 4: 8c, ^{13}C NMR (101 MHz, $CDCl_3$)

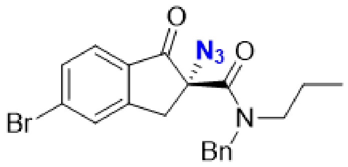

8c

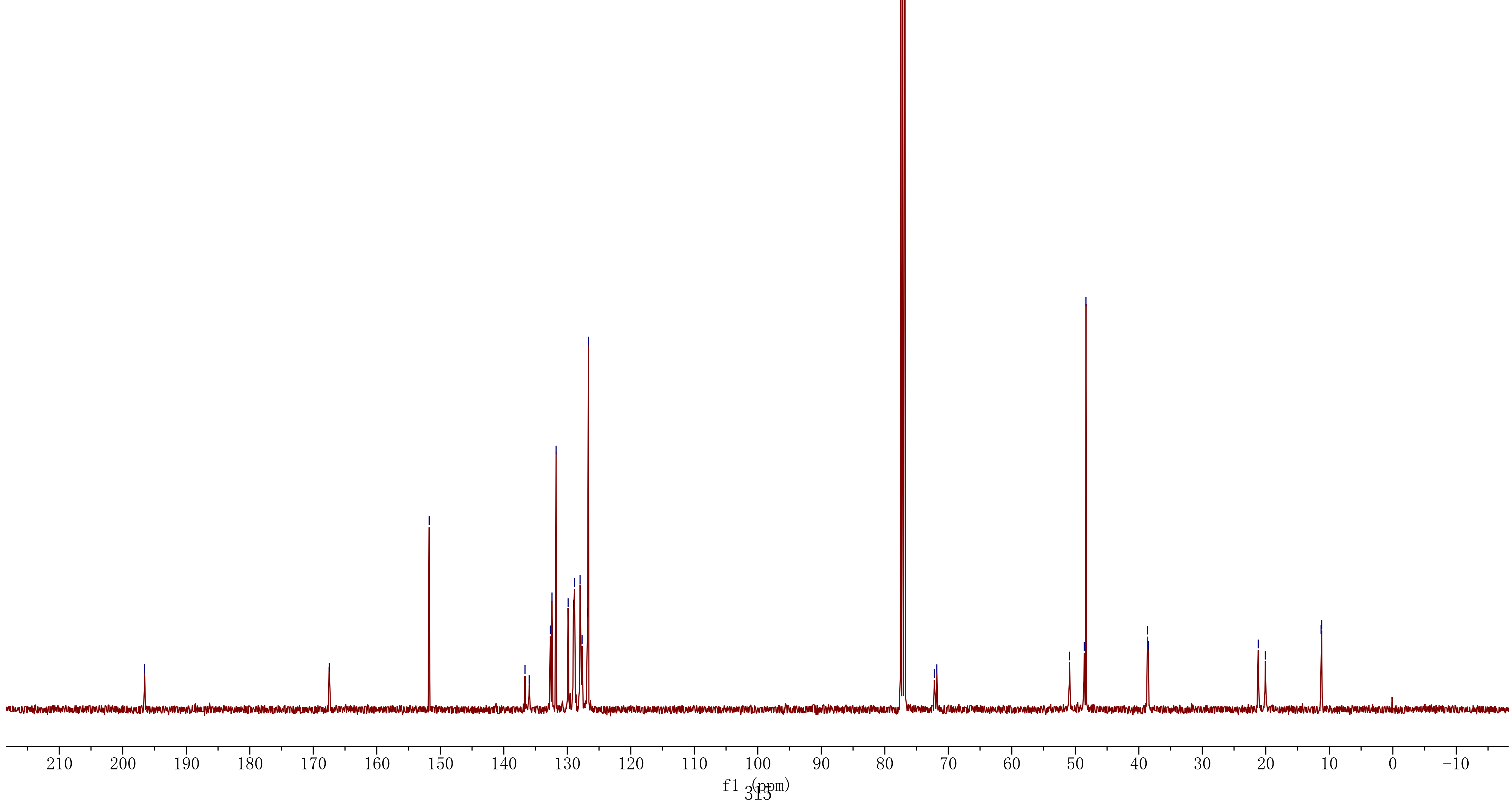

Fig. 4: 8d, ^{1}H NMR (400 MHz, $CDCl_3$)

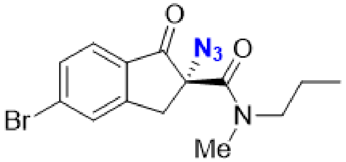

8d

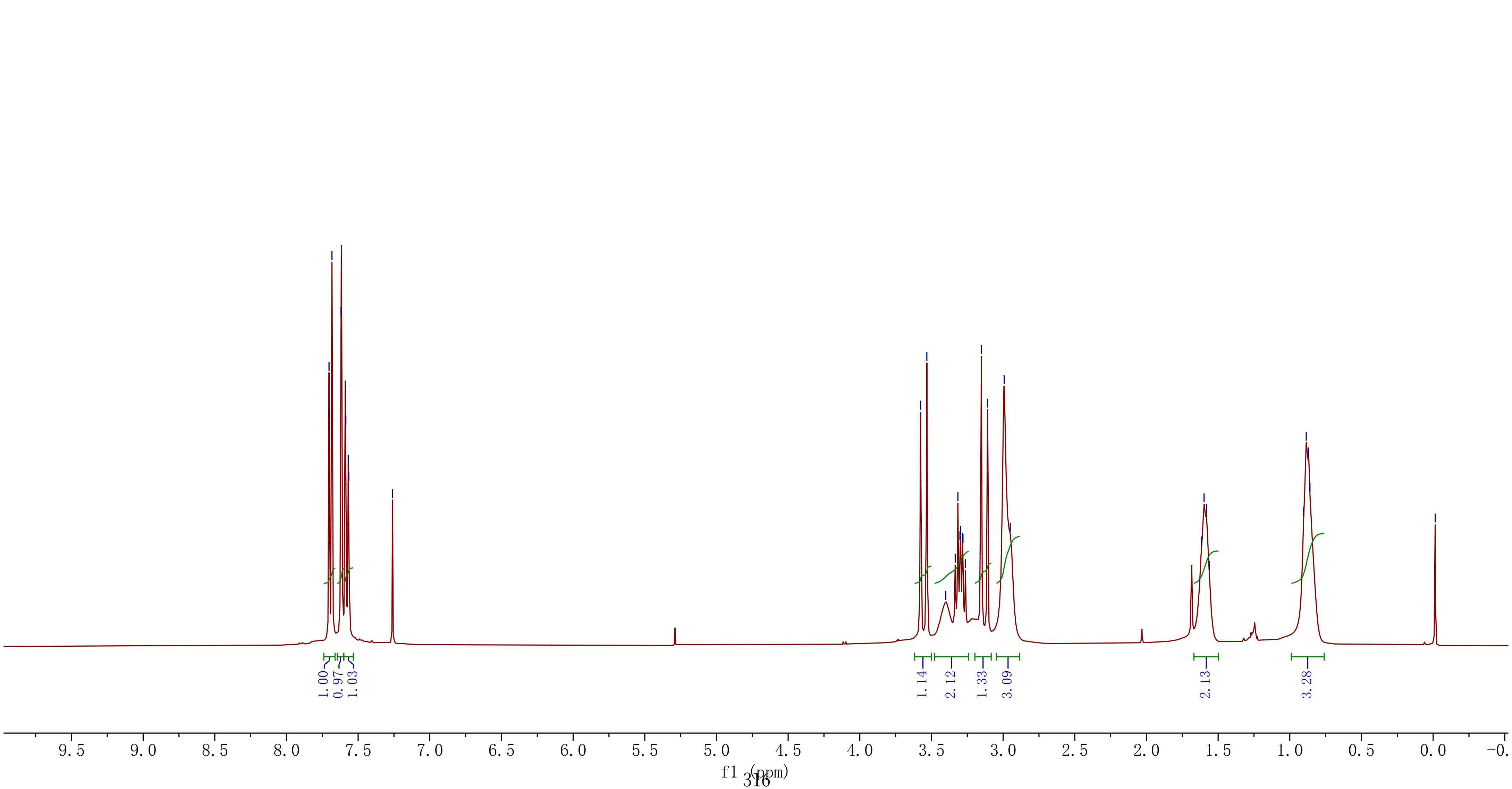

Fig. 4: 8d, ^{13}C NMR (101 MHz, $CDCl_3$)

8d

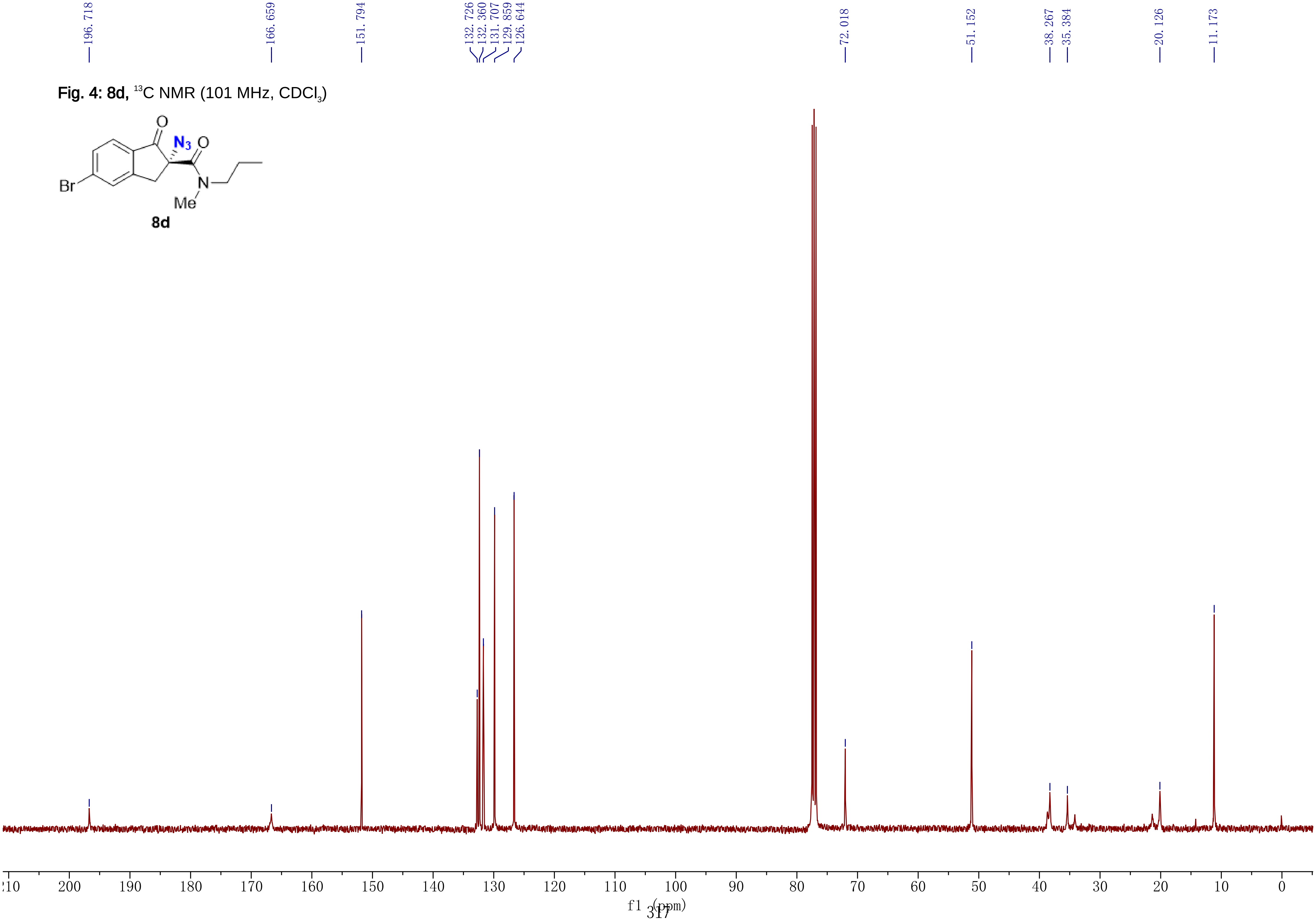

4.2 Chromatographic Data for Chiral Products

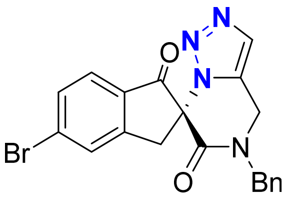

3a

HPLC Conditions
Column: Chiralpak AD-H,
Daicel Chemical Industries Ltd.
Eluent: Hexanes / Isopropanol (70:30)
Flow rate: 1.0 mL/min
Detection: UV 254 nm

Chiral

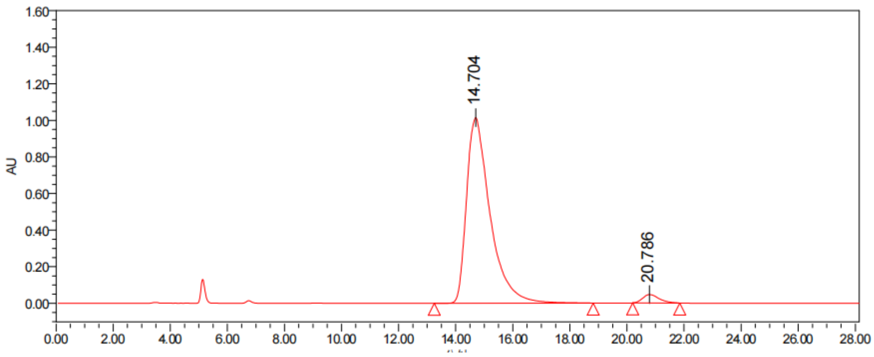

	保留时间 (分钟)	面积	% 面积
1	14.704	58013661	96.92
2	20.786	1841574	3.08

Racemic

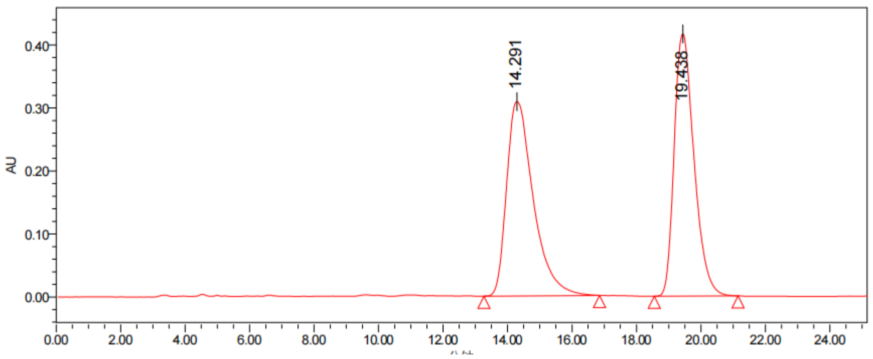

	保留时间 (分钟)	面积	% 面积
1	14.291	17748788	49.98
2	19.438	17762094	50.02

Supplementary Fig. 7. HPLC spectrum of compound **3a**

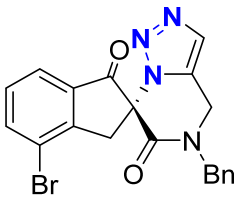

3b

HPLC Conditions
Column: Chiralpak OD-H,
Daicel Chemical Industries Ltd.
Eluent: Hexanes / Isopropanol (70:30)
Flow rate: 1.0 mL/min
Detection: UV 254 nm

Chiral

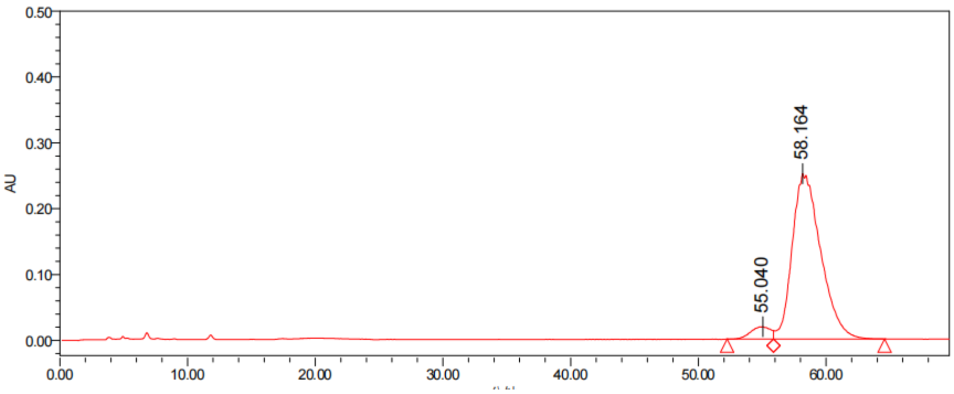

	保留时间 (分钟)	面积	% 面积
1	55.040	2017644	4.91
2	58.164	39033574	95.09

Racemic

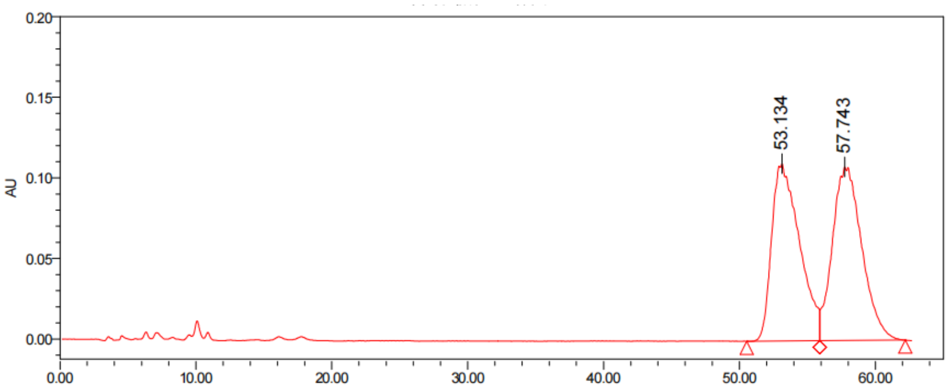

	保留时间 (分钟)	面积	% 面积
1	53.134	15672912	49.05
2	57.743	16278298	50.95

Supplementary Fig. 8. HPLC spectrum of compound **3b**

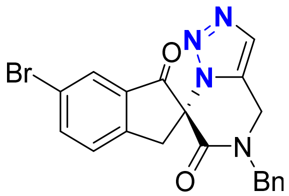

3c

HPLC Conditions
Column: Chiralpak AD-H,
Daicel Chemical Industries Ltd.
Eluent: Hexanes / Isopropanol (70:30)
Flow rate: 1.0 mL/min
Detection: UV 254 nm

Chiral

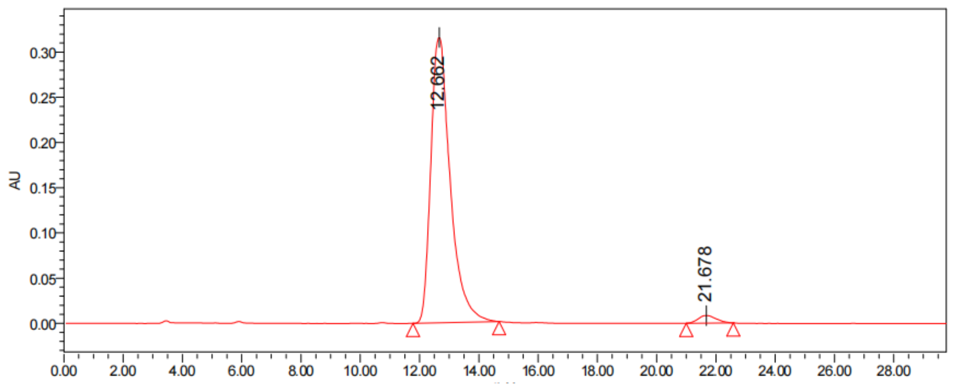

	保留时间 (分钟)	面积	% 面积
1	12.662	14330987	97.60
2	21.678	352937	2.40

Racemic

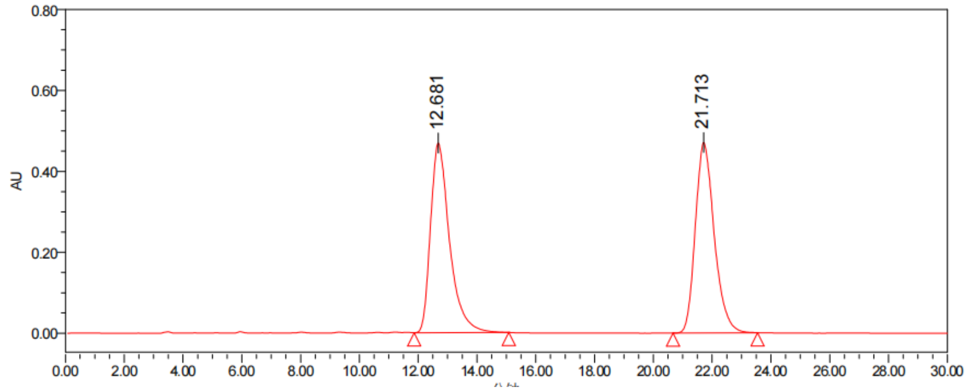

	保留时间 (分钟)	面积	% 面积
1	12.681	20988044	49.94
2	21.713	21036811	50.06

Supplementary Fig. 9. HPLC spectrum of compound **3c**

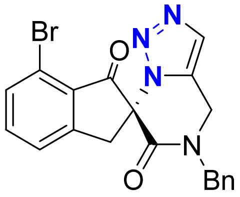

3d

HPLC Conditions
Column: Chiralpak AD-H,
Daicel Chemical Industries Ltd.
Eluent: Hexanes / Isopropanol (70:30)
Flow rate: 1.0 mL/min
Detection: UV 254 nm

Chiral

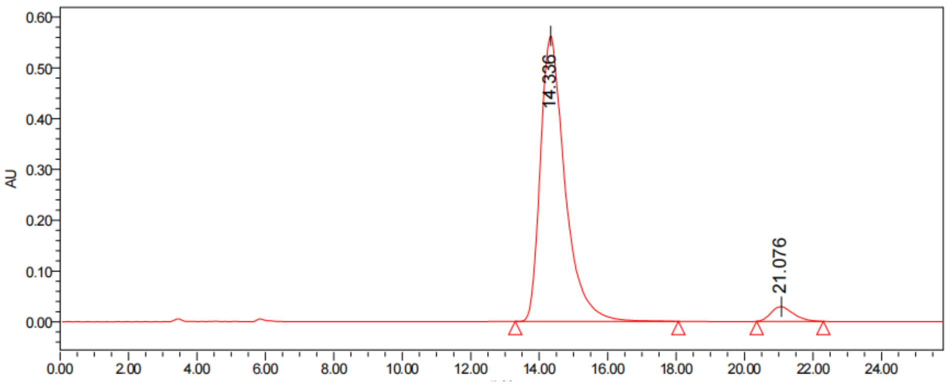

	保留时间 (分钟)	面积	% 面积
1	14.336	28004057	95.53
2	21.076	1311520	4.47

Racemic

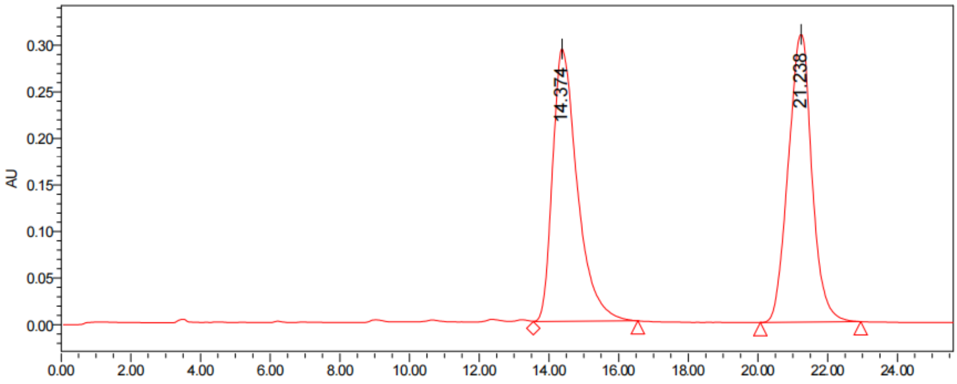

	保留时间 (分钟)	面积	% 面积
1	14.374	14434518	49.74
2	21.238	14585151	50.26

Supplementary Fig. 10. HPLC spectrum of compound **3d**

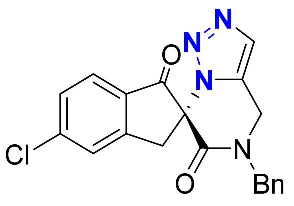

3e

HPLC Conditions
Column: Chiralpak AD-H,
Daicel Chemical Industries Ltd.
Eluent: Hexanes / Isopropanol (70:30)
Flow rate: 1.0 mL/min
Detection: UV 254 nm

Chiral

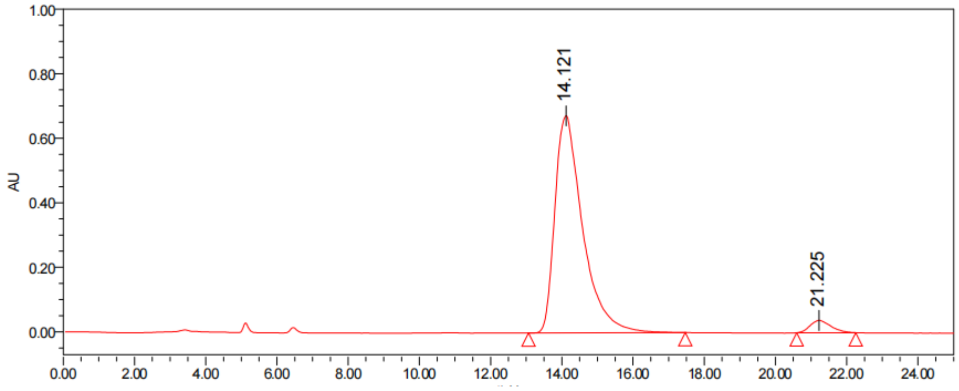

	保留时间 (分钟)	面积	% 面积
1	14.121	36451599	95.95
2	21.225	1537445	4.05

Racemic

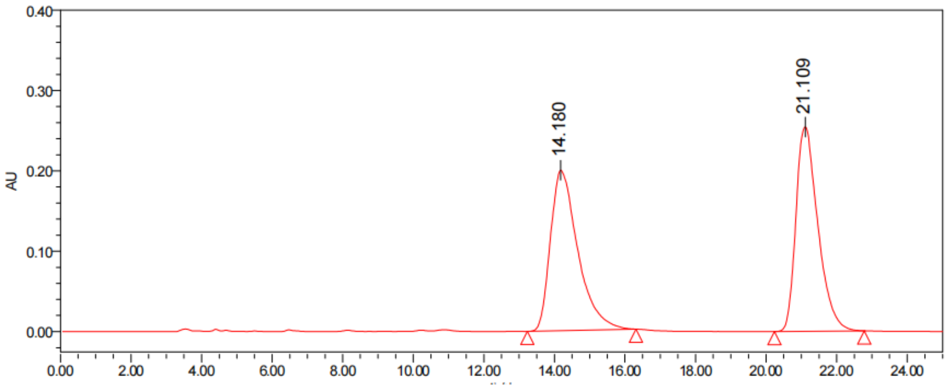

	保留时间 (分钟)	面积	% 面积
1	14.180	10783183	49.53
2	21.109	10989306	50.47

Supplementary Fig. 11. HPLC spectrum of compound **3e**

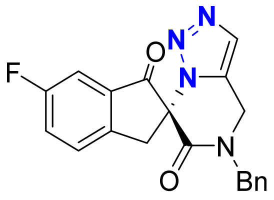

3f

HPLC Conditions

Column: Chiralpak AD-H,
Daicel Chemical Industries Ltd.
Eluent: Hexanes / Isopropanol (70:30)
Flow rate: 1.0 mL/min
Detection: UV 254 nm

Chiral

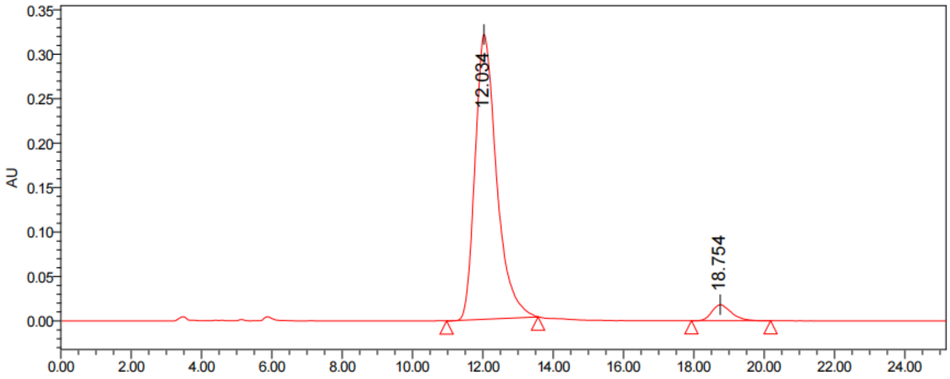

	保留时间 (分钟)	面积	% 面积
1	12.034	13598755	95.15
2	18.754	693850	4.85

Racemic

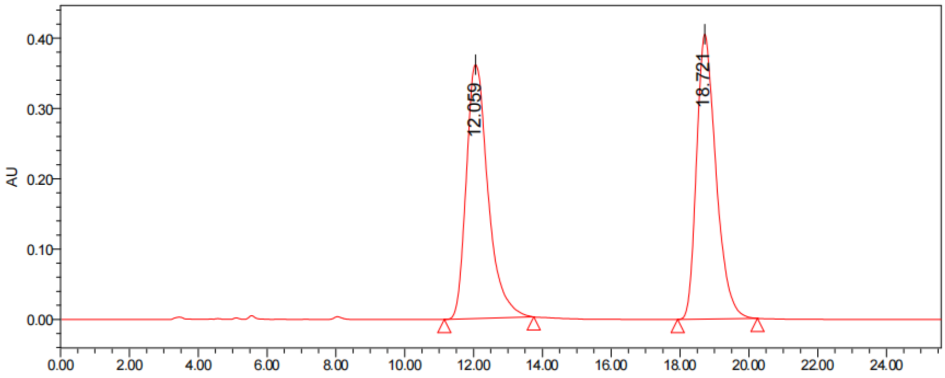

	保留时间 (分钟)	面积	% 面积
1	12.059	15896327	50.01
2	18.721	15887447	49.99

Supplementary Fig. 12. HPLC spectrum of compound **3f**

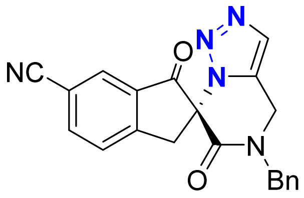

3g

HPLC Conditions
Column: Chiralpak AD-H,
Daicel Chemical Industries Ltd.
Eluent: Hexanes / Isopropanol (60:40)
Flow rate: 1.0 mL/min
Detection: UV 254 nm

Chiral

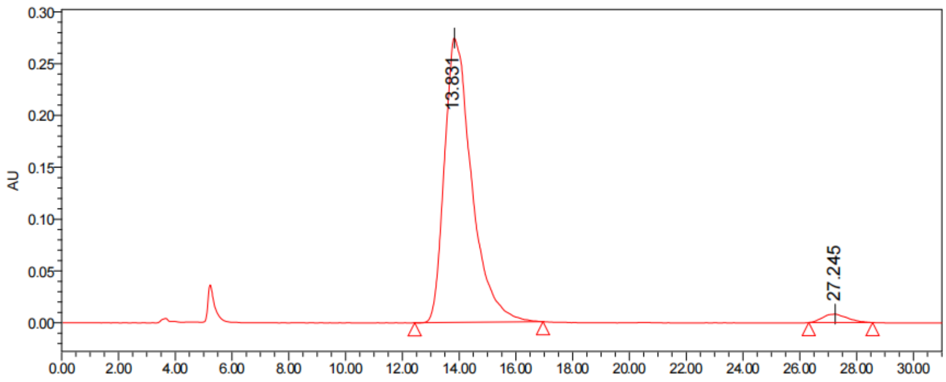

	保留时间 (分钟)	面积	% 面积
1	13.831	18340667	97.44
2	27.245	482071	2.56

Racemic

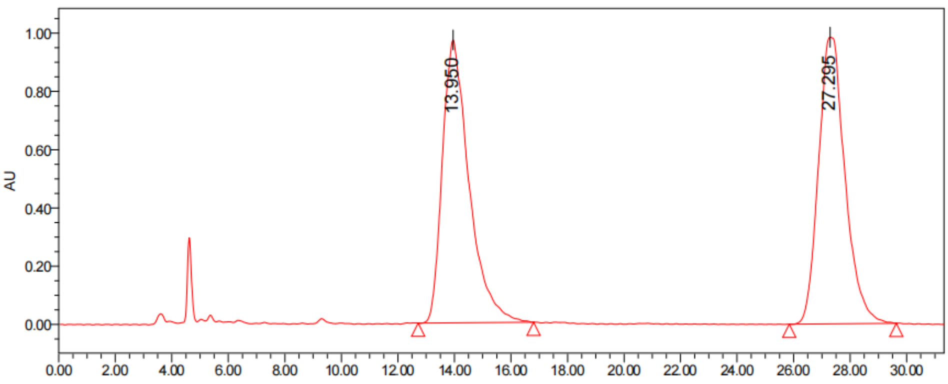

	保留时间 (分钟)	面积	% 面积
1	13.950	63643253	49.82
2	27.295	64112148	50.18

Supplementary Fig. 13. HPLC spectrum of compound **3g**

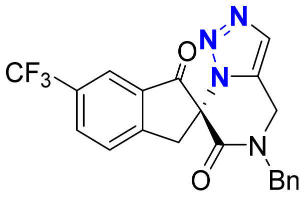

3h

HPLC Conditions
Column: Chiralpak AD-H,
Daicel Chemical Industries Ltd.
Eluent: Hexanes / Isopropanol (70:30)
Flow rate: 1.0 mL/min
Detection: UV 254 nm

Chiral

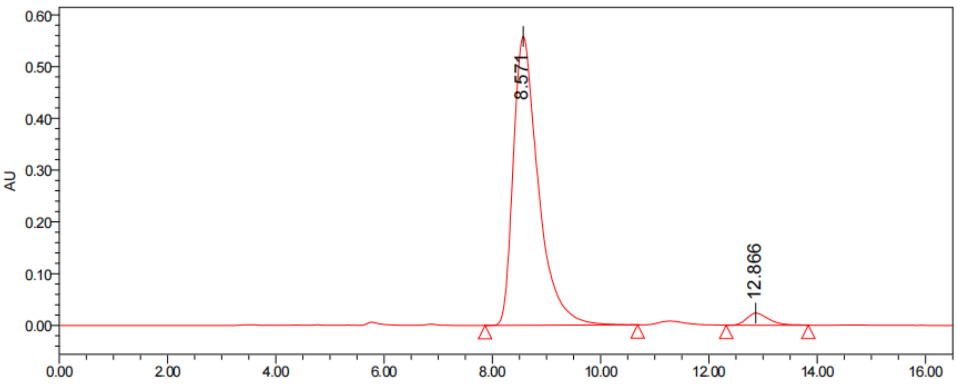

	保留时间 (分钟)	面积	% 面积
1	8.571	17909719	96.53
2	12.866	642951	3.47

Racemic

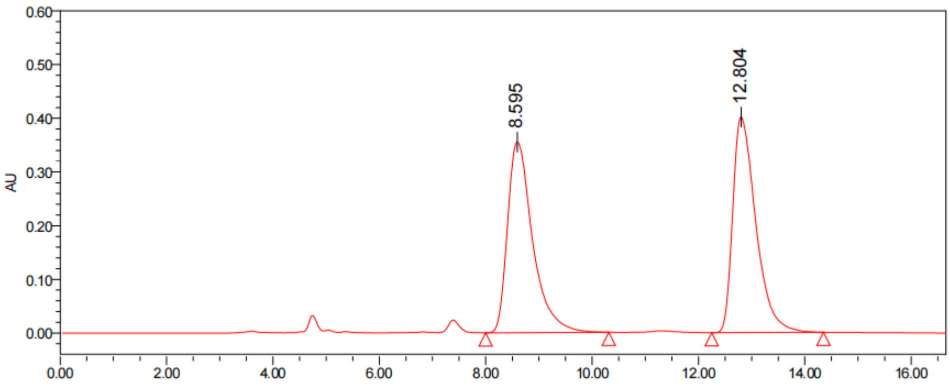

	保留时间 (分钟)	面积	% 面积
1	8.595	11657444	49.05
2	12.804	12107972	50.95

Supplementary Fig. 14. HPLC spectrum of compound **3h**

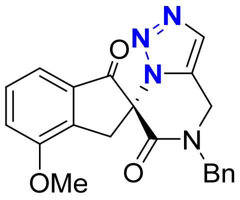

3i

HPLC Conditions
Column: Chiralpak OD-H,
Daicel Chemical Industries Ltd.
Eluent: Hexanes / Isopropanol (50:50)
Flow rate: 1.0 mL/min
Detection: UV 254 nm

Chiral

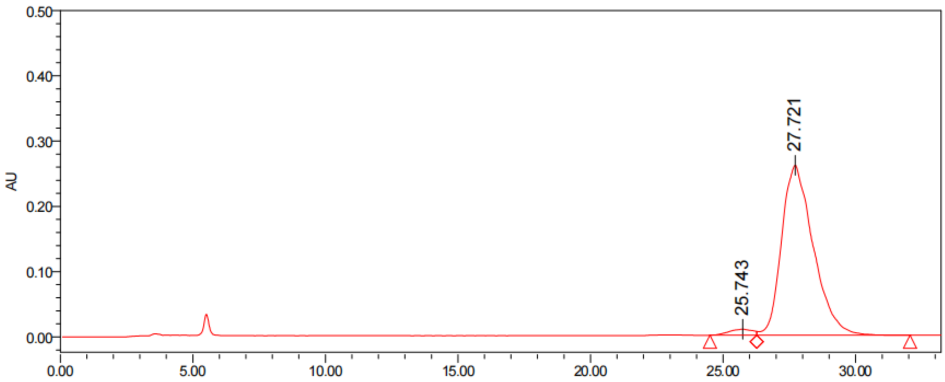

	保留时间 (分钟)	面积	% 面积
1	25.743	571454	2.57
2	27.721	21626812	97.43

Racemic

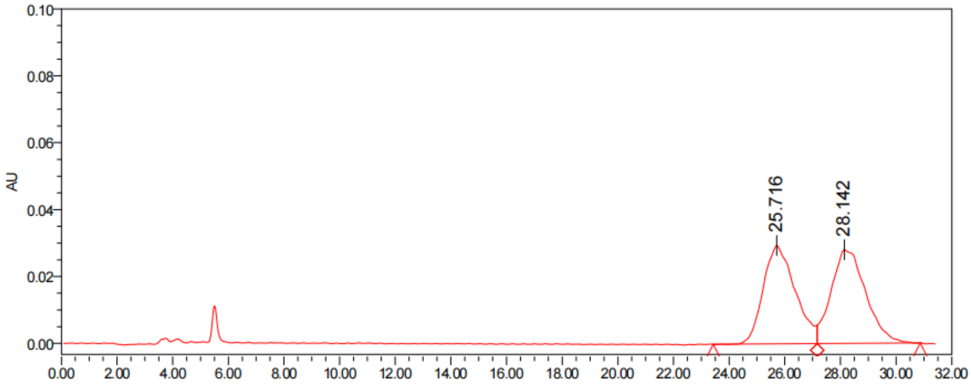

	保留时间 (分钟)	面积	% 面积
1	25.716	2352153	49.24
2	28.142	2424279	50.76

Supplementary Fig. 15. HPLC spectrum of compound **3i**

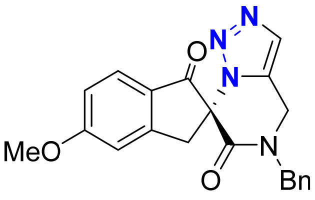

3j

HPLC Conditions
Column: Chiralpak AD-H,
Daicel Chemical Industries Ltd.
Eluent: Hexanes / Isopropanol (70:30)
Flow rate: 1.0 mL/min
Detection: UV 254 nm

Chiral

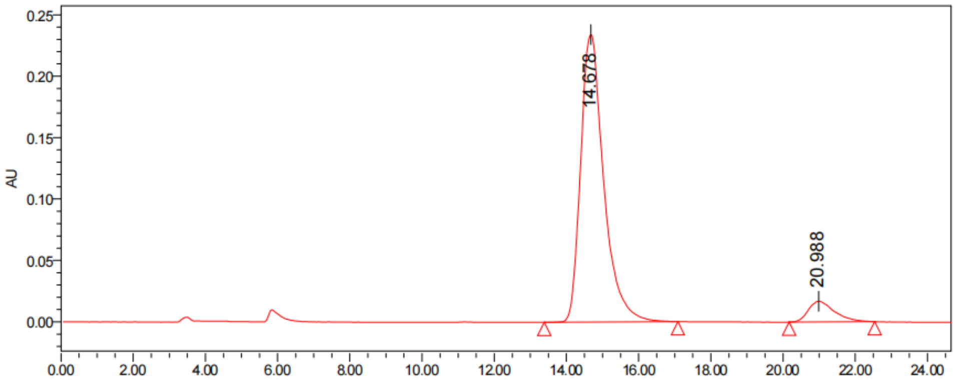

	保留时间 (分钟)	面积	% 面积
1	14.678	10382250	92.46
2	20.988	846268	7.54

Racemic

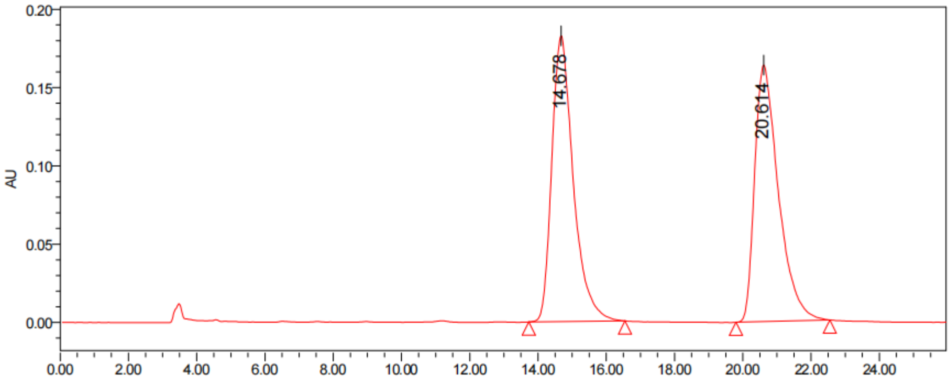

	保留时间 (分钟)	面积	% 面积
1	14.678	7979254	50.22
2	20.614	7908708	49.78

Supplementary Fig. 16. HPLC spectrum of compound **3j**

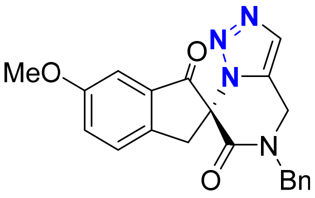

3k

HPLC Conditions
Column: Chiralpak AD-H,
Daicel Chemical Industries Ltd.
Eluent: Hexanes / Isopropanol (70:30)
Flow rate: 1.0 mL/min
Detection: UV 254 nm

Chiral

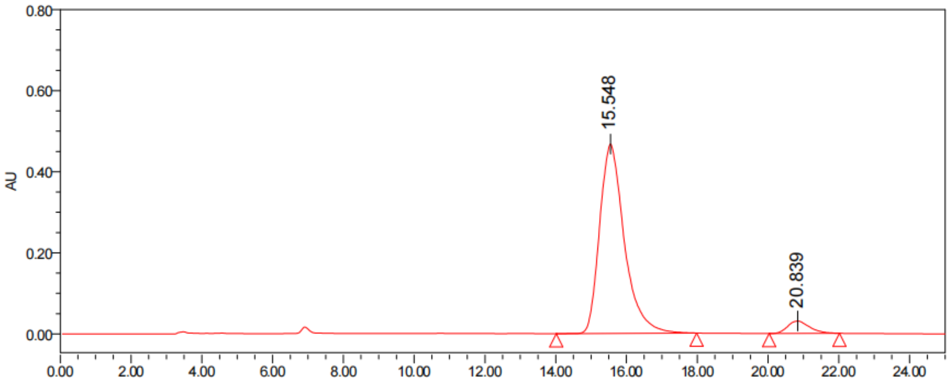

	保留时间 (分钟)	面积	% 面积
1	15.548	22672384	94.50
2	20.839	1319857	5.50

Racemic

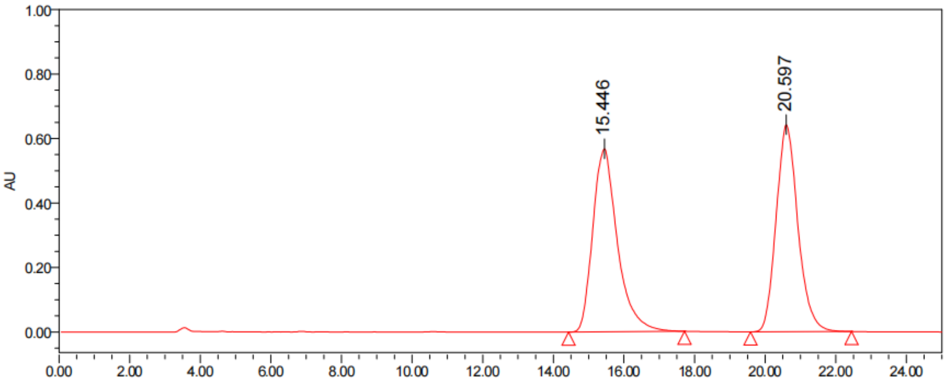

	保留时间 (分钟)	面积	% 面积
1	15.446	27487167	50.03
2	20.597	27459019	49.97

Supplementary Fig. 17. HPLC spectrum of compound **3k**

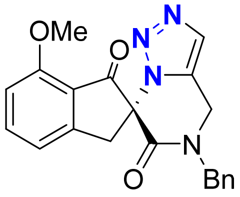

3l

HPLC Conditions
Column: Chiralpak OD-H,
Daicel Chemical Industries Ltd.
Eluent: Hexanes / Isopropanol (50:50)
Flow rate: 1.0 mL/min
Detection: UV 254 nm

Chiral

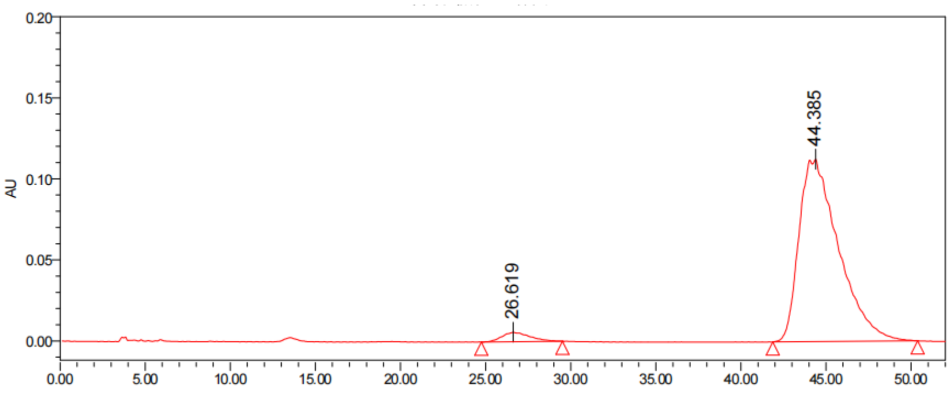

	保留时间 (分钟)	面积	% 面积
1	26.619	660408	3.50
2	44.385	18202343	96.50

Racemic

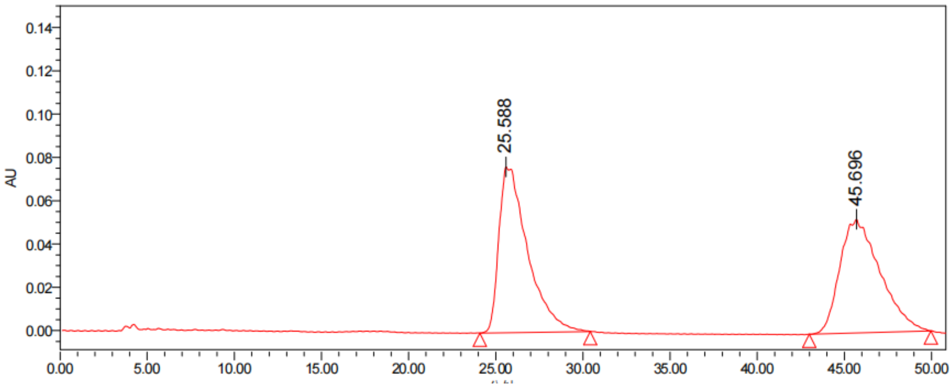

	保留时间 (分钟)	面积	% 面积
1	25.588	8781656	51.04
2	45.696	8423206	48.96

Supplementary Fig. 18. HPLC spectrum of compound **3l**

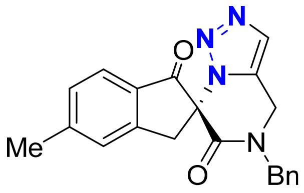

3m

HPLC Conditions
Column: Chiralpak AD-H,
Daicel Chemical Industries Ltd.
Eluent: Hexanes / Isopropanol (70:30)
Flow rate: 1.0 mL/min
Detection: UV 254 nm

Chiral

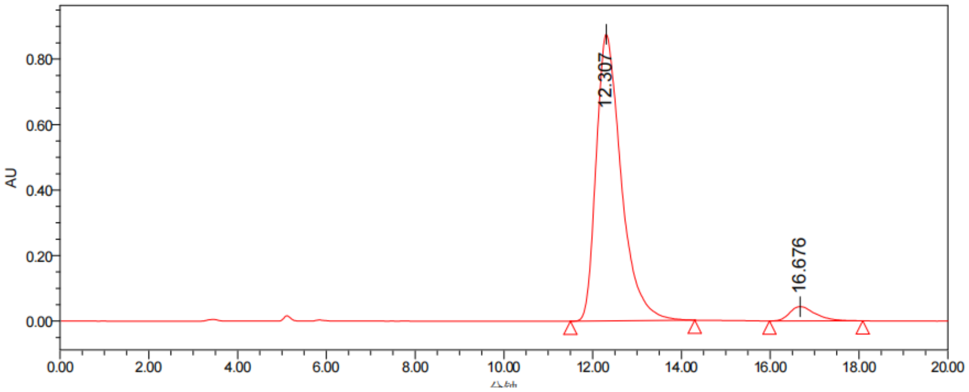

	保留时间 (分钟)	面积	% 面积
1	12.307	34273138	95.17
2	16.676	1737764	4.83

Racemic

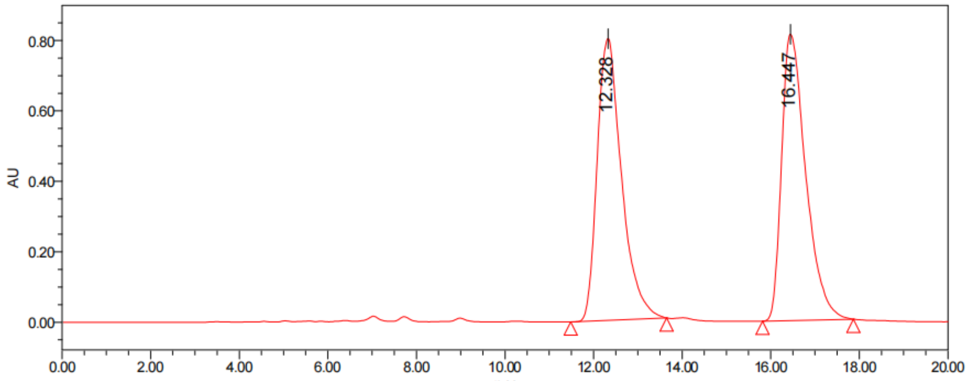

	保留时间 (分钟)	面积	% 面积
1	12.328	30601548	49.86
2	16.447	30769965	50.14

Supplementary Fig. 19. HPLC spectrum of compound **3m**

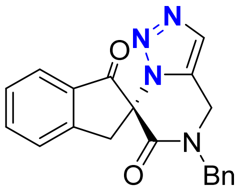

3n

HPLC Conditions

Column: Chiralpak AD-H,

Daicel Chemical Industries Ltd.

Eluent: Hexanes / Isopropanol (70:30)

Flow rate: 1.0 mL/min

Detection: UV 254 nm

Chiral

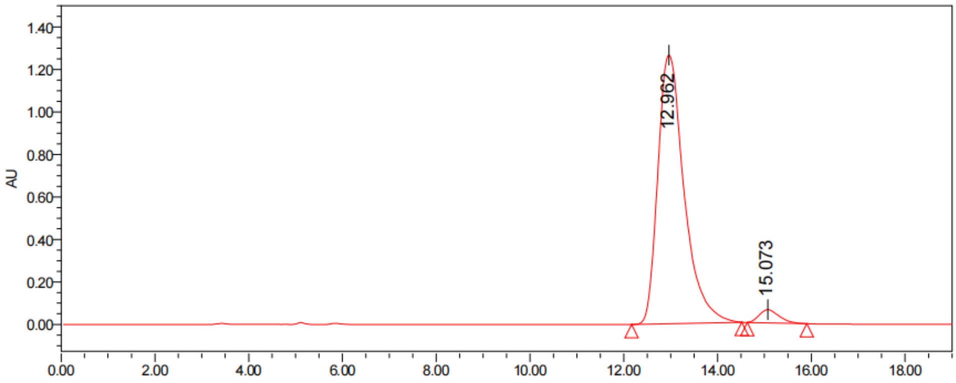

	保留时间 (分钟)	面积	% 面积
1	12.962	49190755	96.45
2	15.073	1812834	3.55

Racemic

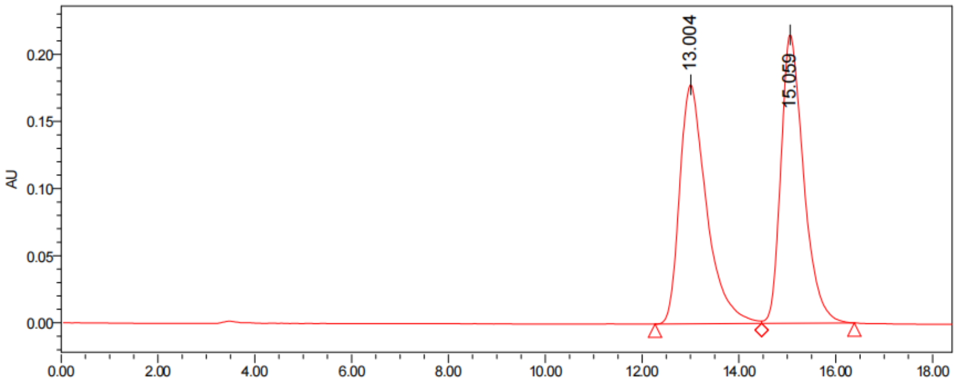

	保留时间 (分钟)	面积	% 面积
1	13.004	6880692	49.55
2	15.059	7006098	50.45

Supplementary Fig. 20. HPLC spectrum of compound **3n**

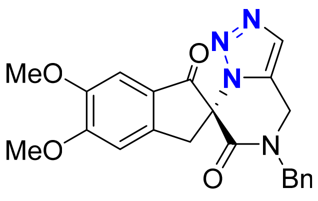

3o

HPLC Conditions
Column: Chiralpak AD-H,
Daicel Chemical Industries Ltd.
Eluent: Hexanes / Isopropanol (60:40)
Flow rate: 1.0 mL/min
Detection: UV 254 nm

Chiral

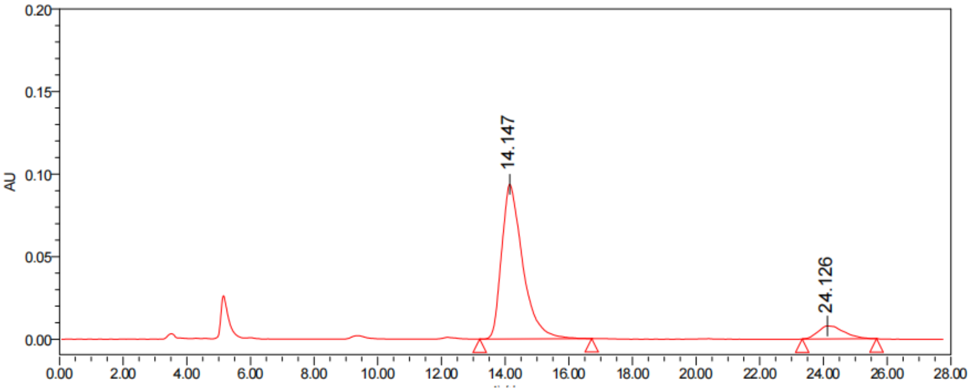

	保留时间 (分钟)	面积	% 面积
1	14.147	4297604	90.58
2	24.126	447017	9.42

Racemic

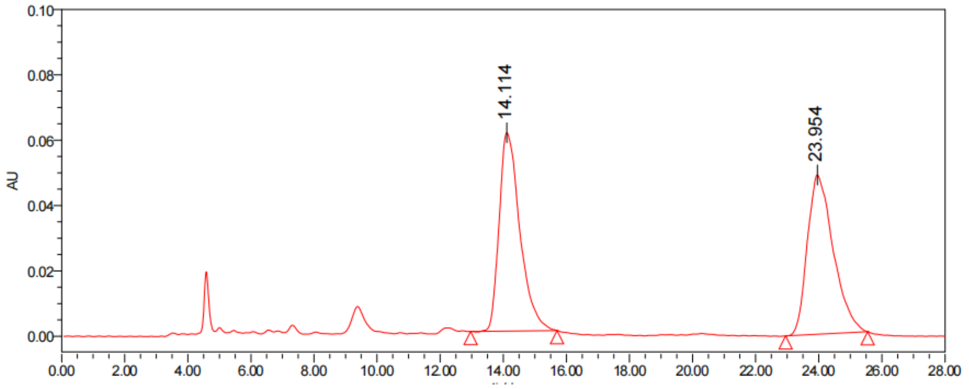

	保留时间 (分钟)	面积	% 面积
1	14.114	2772424	49.92
2	23.954	2780886	50.08

Supplementary Fig. 21. HPLC spectrum of compound **3o**

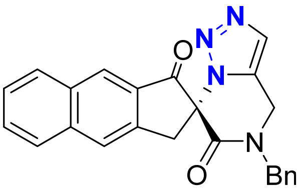

3p

HPLC Conditions
Column: Chiralpak AD-H,
Daicel Chemical Industries Ltd.
Eluent: Hexanes / Isopropanol (70:30)
Flow rate: 1.0 mL/min
Detection: UV 254 nm

Chiral

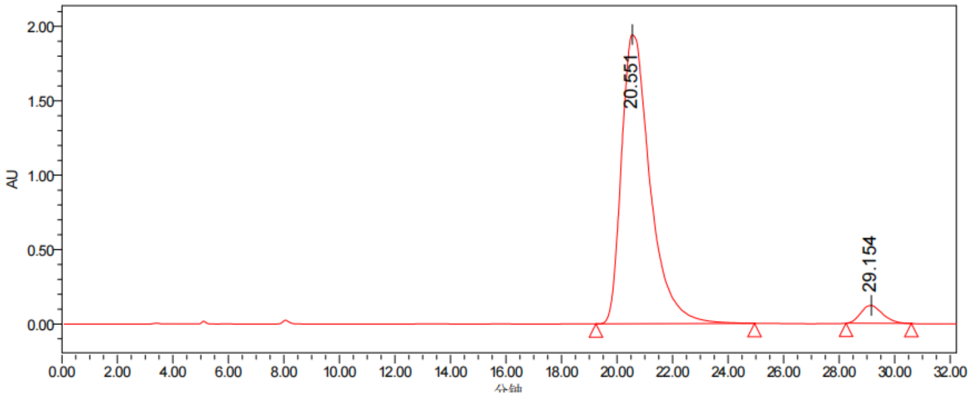

	保留时间 (分钟)	面积	% 面积
1	20.551	139303550	95.47
2	29.154	6615139	4.53

Racemic

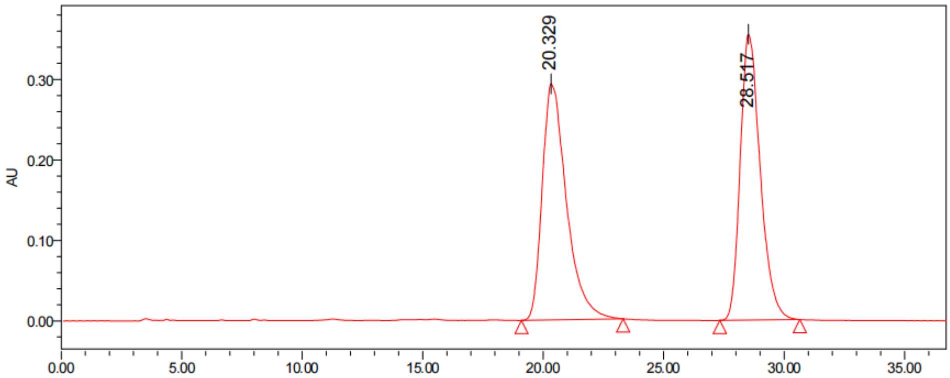

	保留时间 (分钟)	面积	% 面积
1	20.329	20512651	50.03
2	28.517	20485582	49.97

Supplementary Fig. 22. HPLC spectrum of compound **3p**

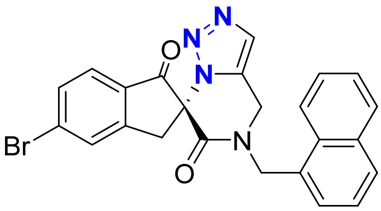

3q

HPLC Conditions
Column: Chiralpak AS-H,
Daicel Chemical Industries Ltd.
Eluent: Hexanes / Isopropanol (60:40)
Flow rate: 1.0 mL/min
Detection: UV 254 nm

Chiral

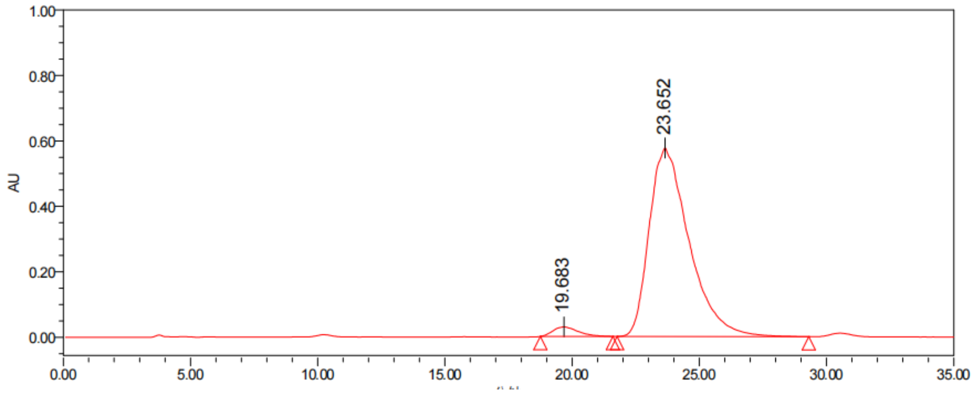

	保留时间 (分钟)	面积	% 面积
1	19.683	1991514	3.08
2	23.652	62720750	96.92

Racemic

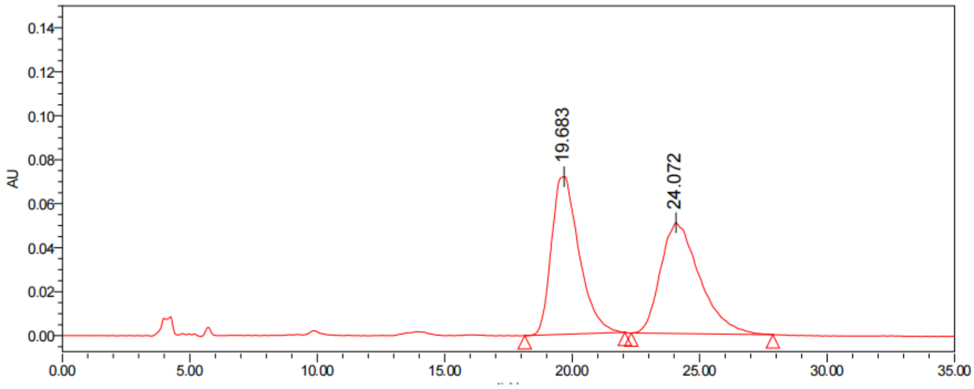

	保留时间 (分钟)	面积	% 面积
1	19.683	5401188	50.13
2	24.072	5372816	49.87

Supplementary Fig. 23. HPLC spectrum of compound **3q**

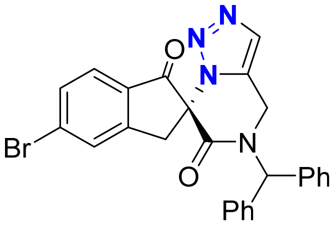

3r

HPLC Conditions
Column: Chiralpak AD-H,
Daicel Chemical Industries Ltd.
Eluent: Hexanes / Isopropanol (70:30)
Flow rate: 1.0 mL/min
Detection: UV 254 nm

Chiral

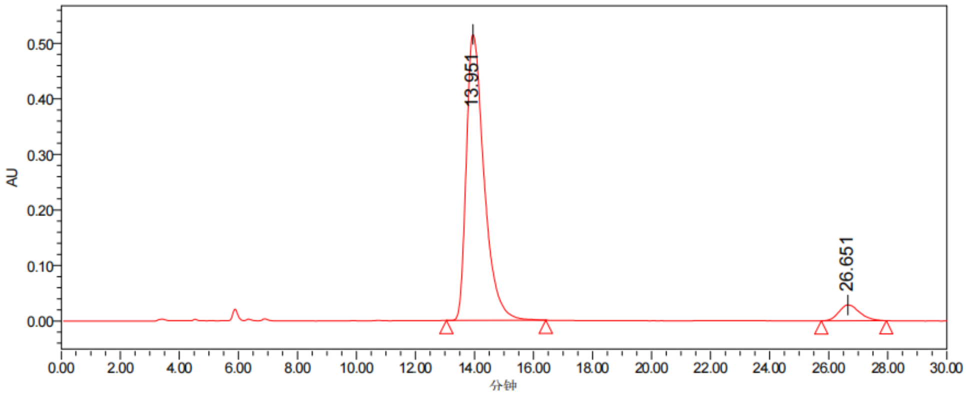

	保留时间 (分钟)	面积	% 面积
1	13.951	21525241	93.94
2	26.651	1387615	6.06

Racemic

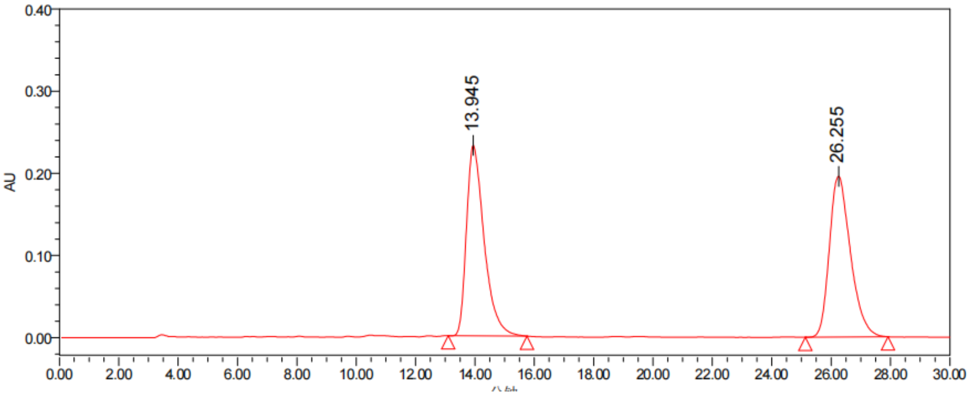

	保留时间 (分钟)	面积	% 面积
1	13.945	9582787	49.73
2	26.255	9686649	50.27

Supplementary Fig. 24. HPLC spectrum of compound **3r**

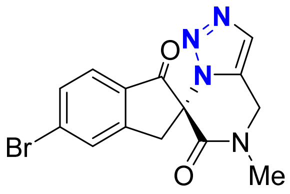

3s

HPLC Conditions
Column: Chiralpak AD-H,
Daicel Chemical Industries Ltd.
Eluent: Hexanes / Isopropanol (90:10)
Flow rate: 1.0 mL/min
Detection: UV 254 nm

Chiral

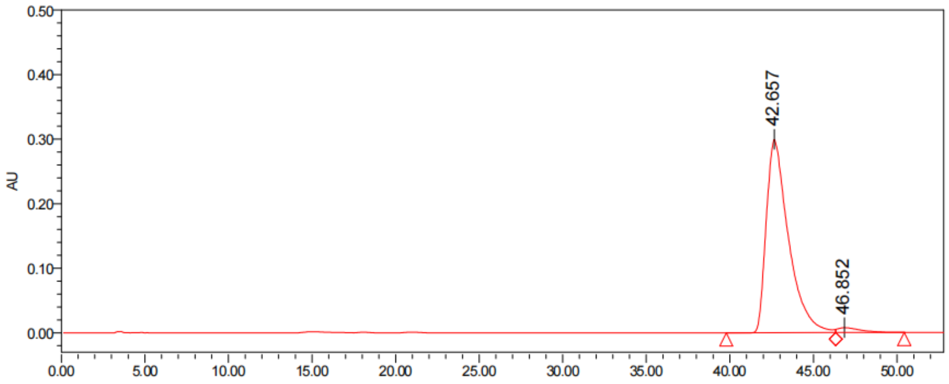

	保留时间 (分钟)	面积	% 面积
1	42.657	27661299	97.49
2	46.852	712599	2.51

Racemic

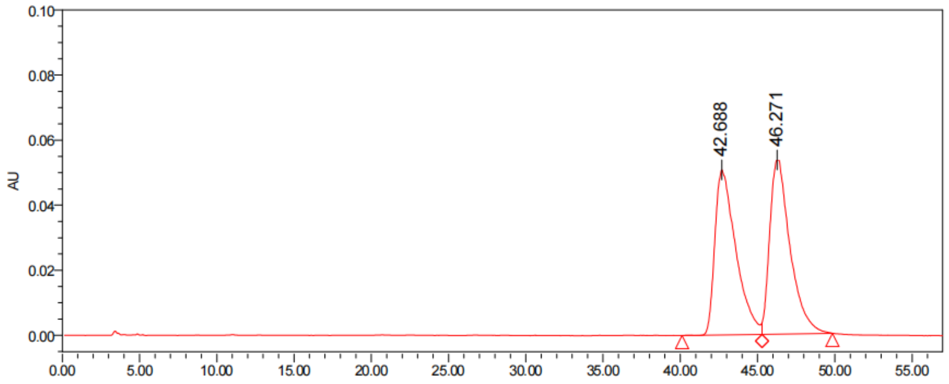

	保留时间 (分钟)	面积	% 面积
1	42.688	4791308	49.37
2	46.271	4914212	50.63

Supplementary Fig. 25. HPLC spectrum of compound **3s**

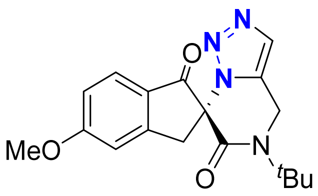

3t

HPLC Conditions
Column: Chiralpak AD-H,
Daicel Chemical Industries Ltd.
Eluent: Hexanes / Isopropanol (70:30)
Flow rate: 1.0 mL/min
Detection: UV 254 nm

Chiral

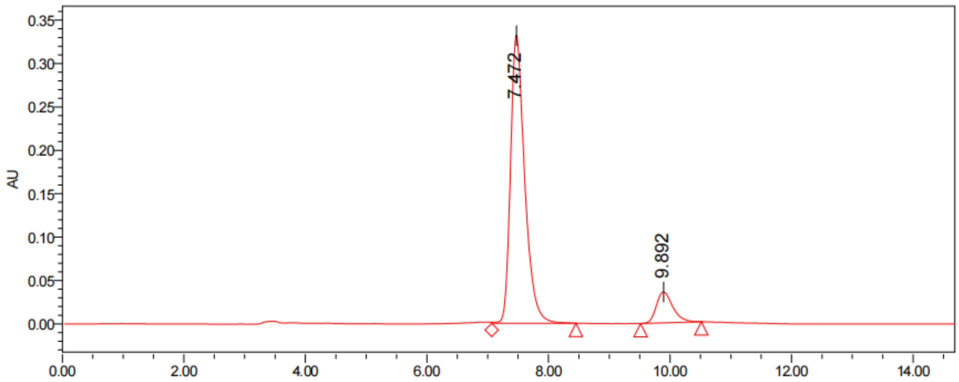

	保留时间 (分钟)	面积	% 面积
1	7.472	5372963	89.00
2	9.892	664060	11.00

Racemic

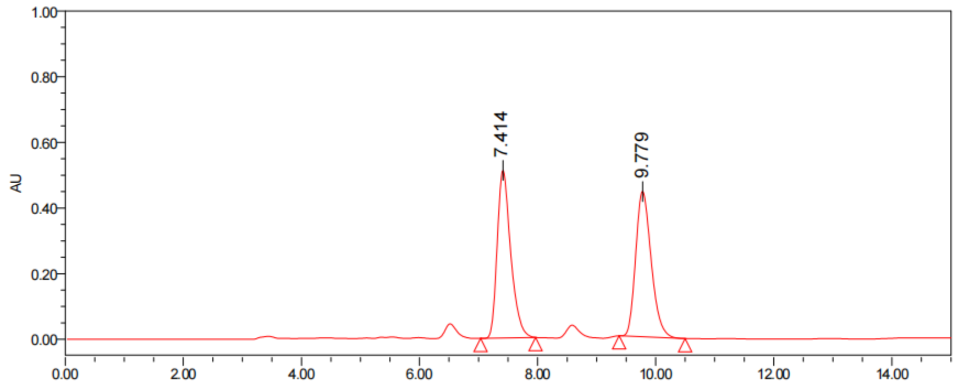

	保留时间 (分钟)	面积	% 面积
1	7.414	8125313	50.24
2	9.779	8049164	49.76

Supplementary Fig. 26. HPLC spectrum of compound **3t**

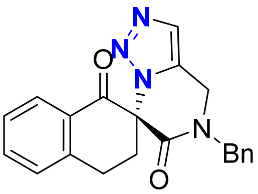

3u

HPLC Conditions
Column: Chiralpak AD-H,
Daicel Chemical Industries Ltd.
Eluent: Hexanes / Isopropanol (70:30)
Flow rate: 1.0 mL/min
Detection: UV 254 nm

Chiral

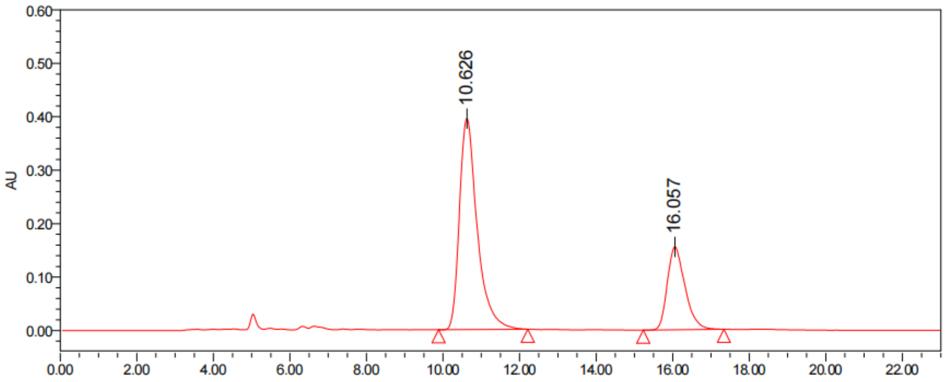

	保留时间 (分钟)	面积	% 面积
1	10.626	12754082	71.86
2	16.057	4993694	28.14

Racemic

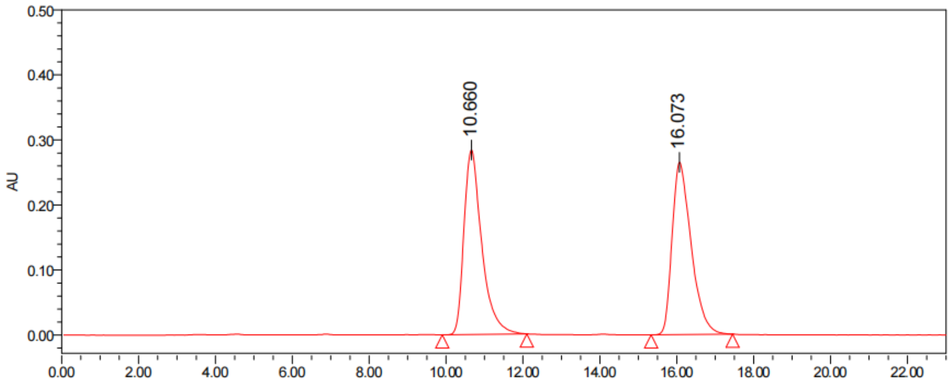

	保留时间 (分钟)	面积	% 面积
1	10.660	9135024	50.01
2	16.073	9131714	49.99

Supplementary Fig. 27. HPLC spectrum of compound **3u**

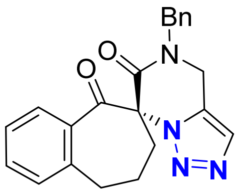

3v

HPLC Conditions
Column: Chiralpak AD-H,
Daicel Chemical Industries Ltd.
Eluent: Hexanes / Isopropanol (70:30)
Flow rate: 1.0 mL/min
Detection: UV 254 nm

Chiral

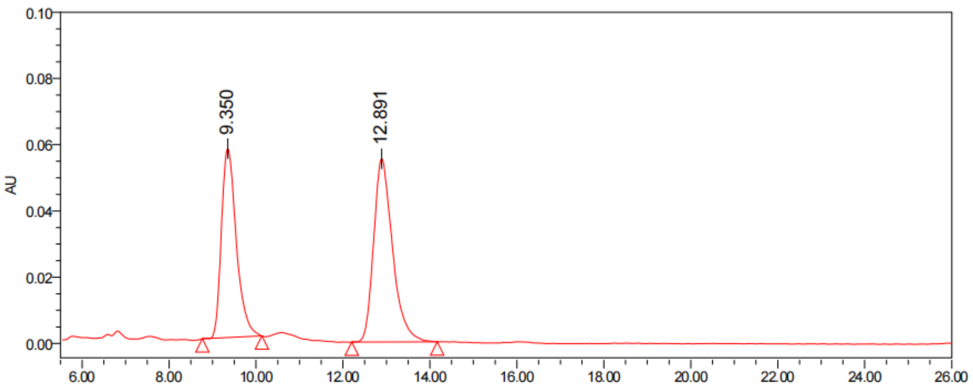

	保留时间 (分钟)	面积	% 面积
1	9.350	1358525	44.85
2	12.891	1670399	55.15

Racemic

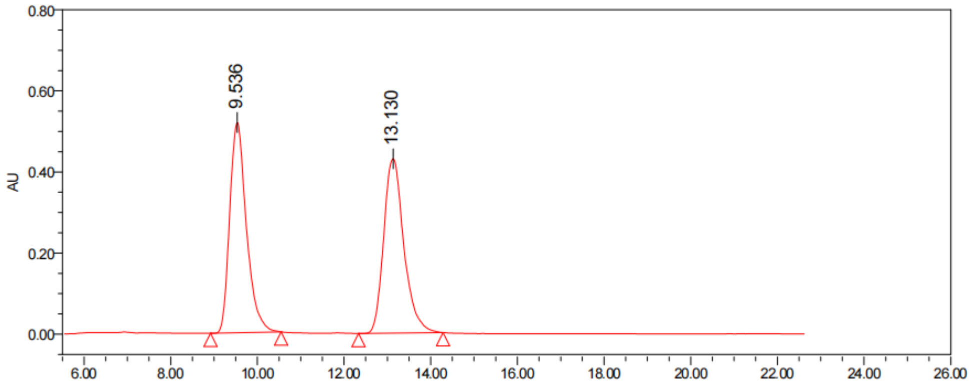

	保留时间 (分钟)	面积	% 面积
1	9.536	13889859	50.01
2	13.130	13884651	49.99

Supplementary Fig. 28. HPLC spectrum of compound **3v**

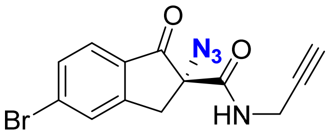

3w'

HPLC Conditions
Column: Chiralpak AD-H,
Daicel Chemical Industries Ltd.
Eluent: Hexanes / Isopropanol (90:10)
Flow rate: 1.0 mL/min
Detection: UV 254 nm

Chiral

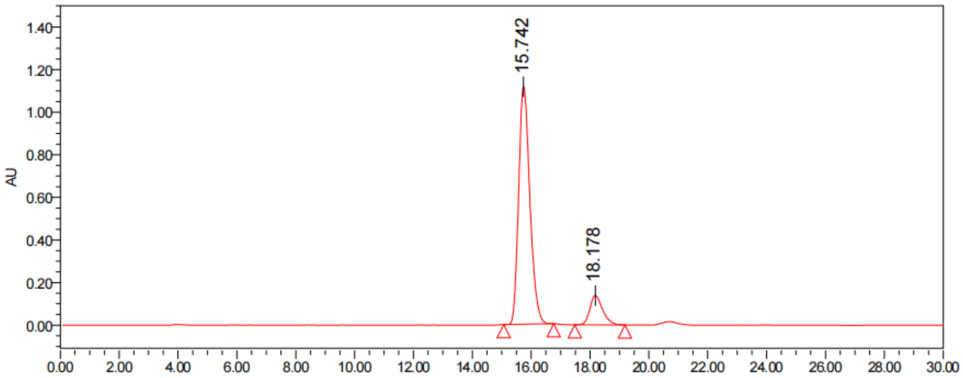

	保留时间 (分钟)	面积	% 面积
1	15.742	29528095	87.72
2	18.178	4132625	12.28

Racemic

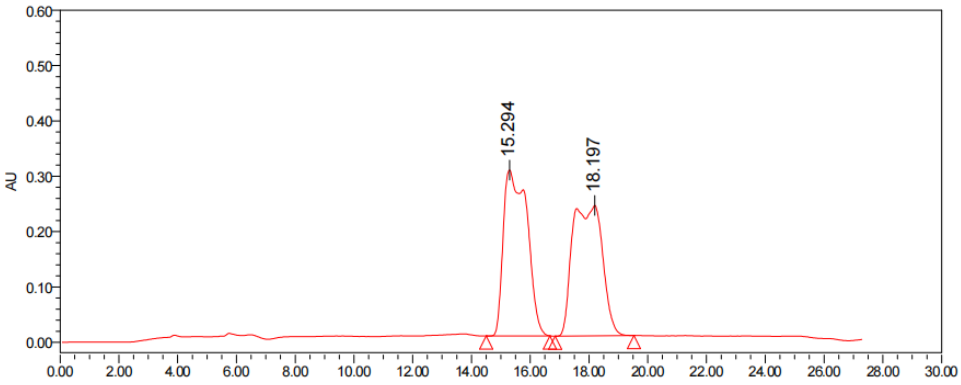

	保留时间 (分钟)	面积	% 面积
1	15.294	16825642	49.99
2	18.197	16833906	50.01

Supplementary Fig. 29. HPLC spectrum of compound **3w**′

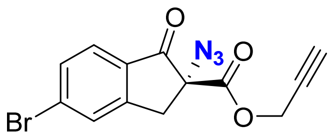

3x'

HPLC Conditions
Column: Chiralpak AD-H,
Daicel Chemical Industries Ltd.
Eluent: Hexanes / Isopropanol (95:5)
Flow rate: 1.0 mL/min
Detection: UV 254 nm

Chiral

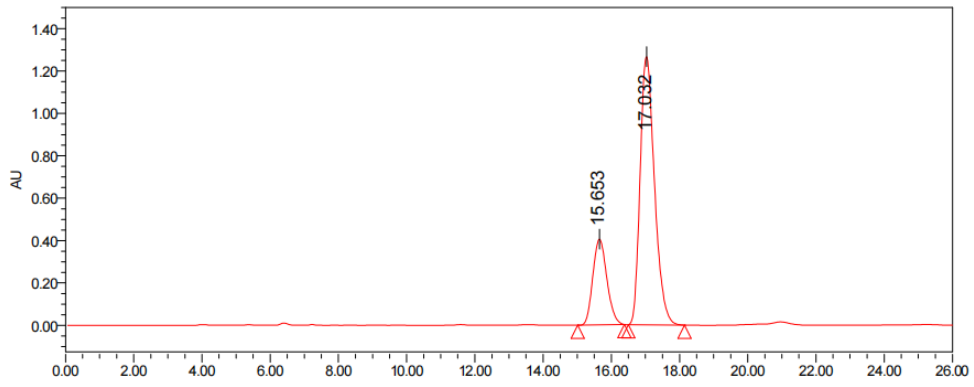

	保留时间 (分钟)	面积	% 面积
1	15.653	11543362	23.84
2	17.032	36876325	76.16

Racemic

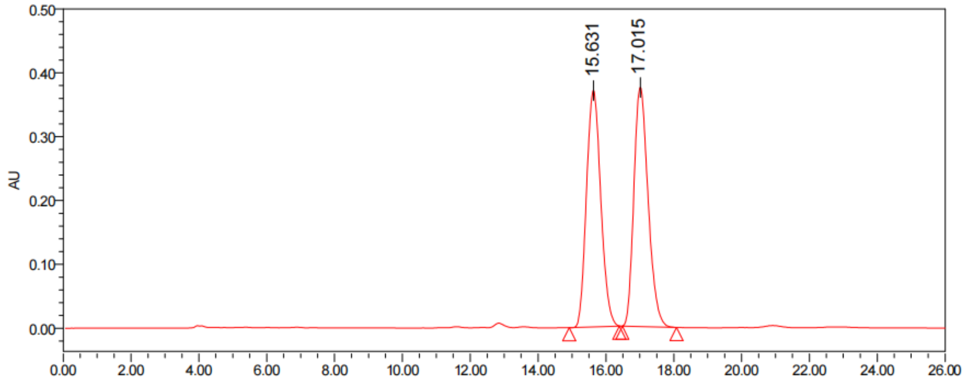

	保留时间 (分钟)	面积	% 面积
1	15.631	10821893	49.74
2	17.015	10933486	50.26

Supplementary Fig. 30. HPLC spectrum of compound **3x**'

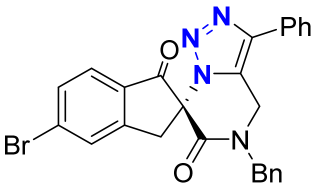

5a

HPLC Conditions
Column: Chiralpak AD-H,
Daicel Chemical Industries Ltd.
Eluent: Hexanes / Isopropanol (70:30)
Flow rate: 1.0 mL/min
Detection: UV 254 nm

Chiral

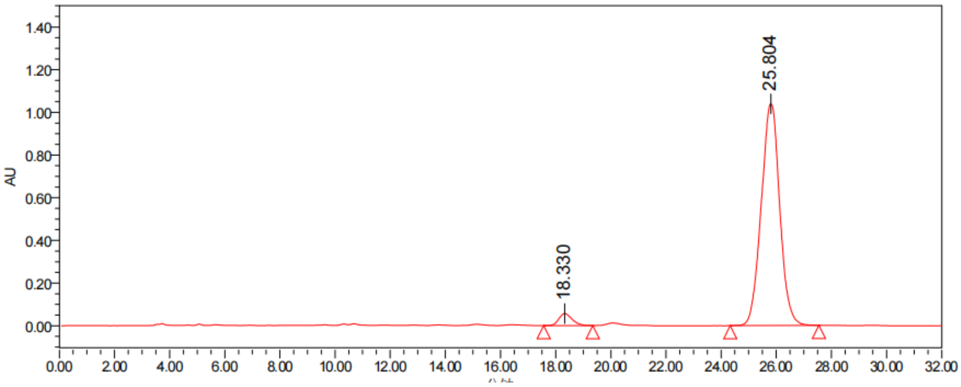

	保留时间 (分钟)	面积	% 面积
1	18.330	1887368	3.75
2	25.804	48376166	96.25

Racemic

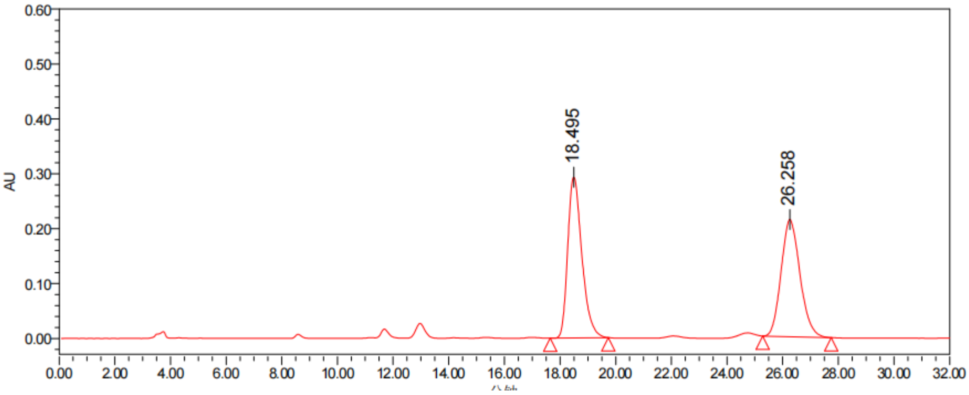

	保留时间 (分钟)	面积	% 面积
1	18.495	10352242	50.67
2	26.258	10078694	49.33

Supplementary Fig. 31. HPLC spectrum of compound **5a**

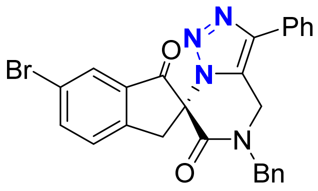

5b

HPLC Conditions
Column: Chiralpak AD-H,
Daicel Chemical Industries Ltd.
Eluent: Hexanes / Isopropanol (70:30)
Flow rate: 1.0 mL/min
Detection: UV 254 nm

Chiral

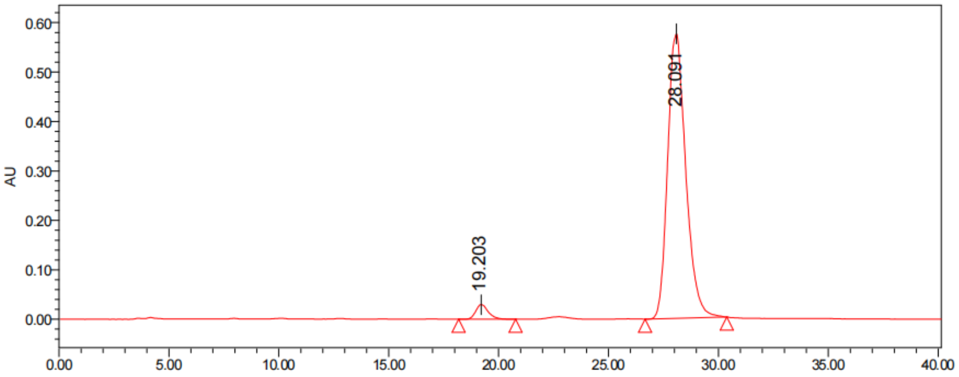

	保留时间 (分钟)	面积	% 面积
1	19.203	1173036	3.43
2	28.091	33005376	96.57

Racemic

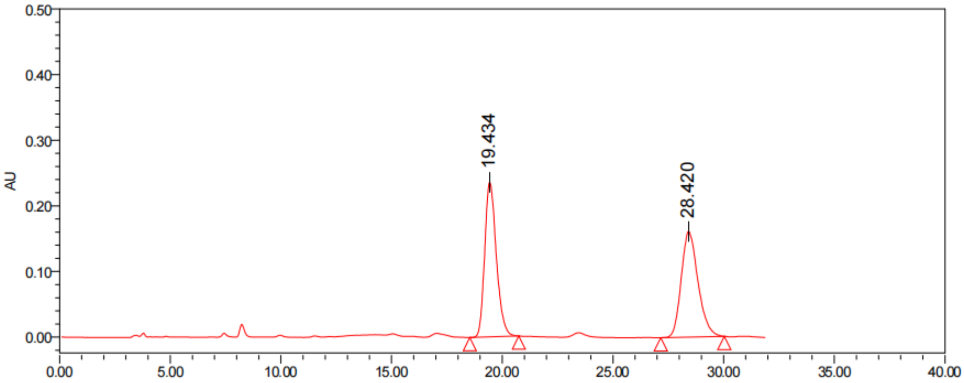

	保留时间 (分钟)	面积	% 面积
1	19.434	8596329	50.42
2	28.420	8454773	49.58

Supplementary Fig. 32. HPLC spectrum of compound **5b**

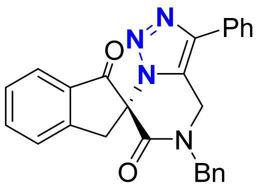

5c

HPLC Conditions
Column: Chiralpak AD-H,
Daicel Chemical Industries Ltd.
Eluent: Hexanes / Isopropanol (70:30)
Flow rate: 1.0 mL/min
Detection: UV 254 nm

Chiral

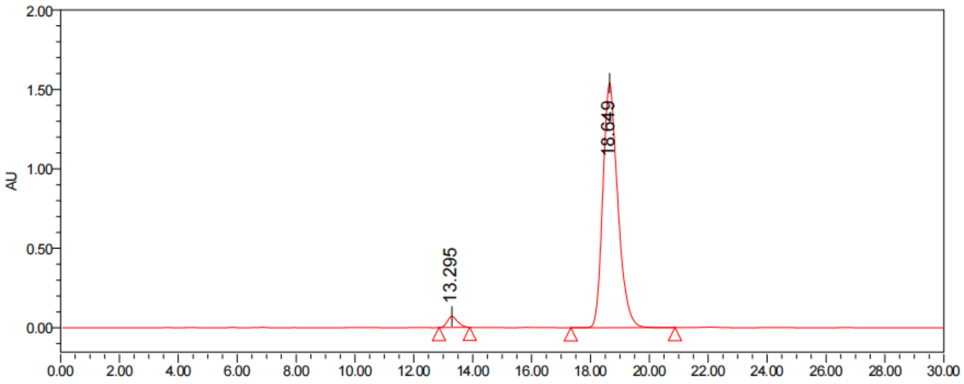

	保留时间 (分钟)	面积	% 面积
1	13.295	1709896	3.06
2	18.649	54137899	96.94

Racemic

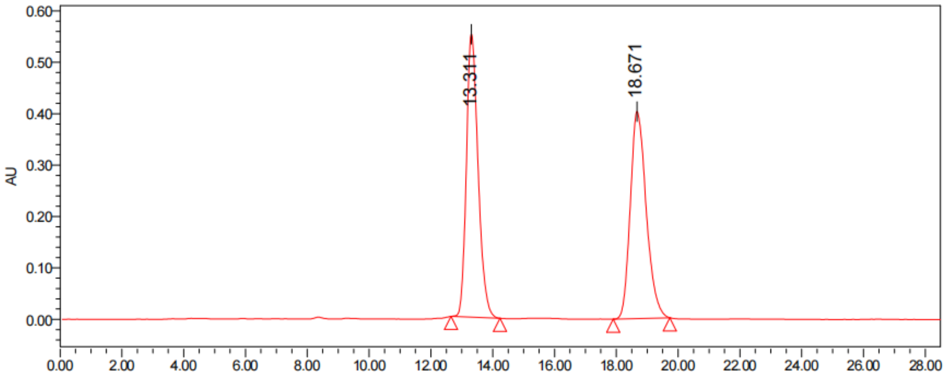

	保留时间 (分钟)	面积	% 面积
1	13.311	14476887	50.10
2	18.671	14417206	49.90

Supplementary Fig. 33. HPLC spectrum of compound **5c**

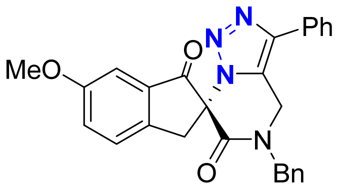

5d

HPLC Conditions
Column: Chiralpak AD-H,
Daicel Chemical Industries Ltd.
Eluent: Hexanes / Isopropanol (70:30)
Flow rate: 1.0 mL/min
Detection: UV 254 nm

Chiral

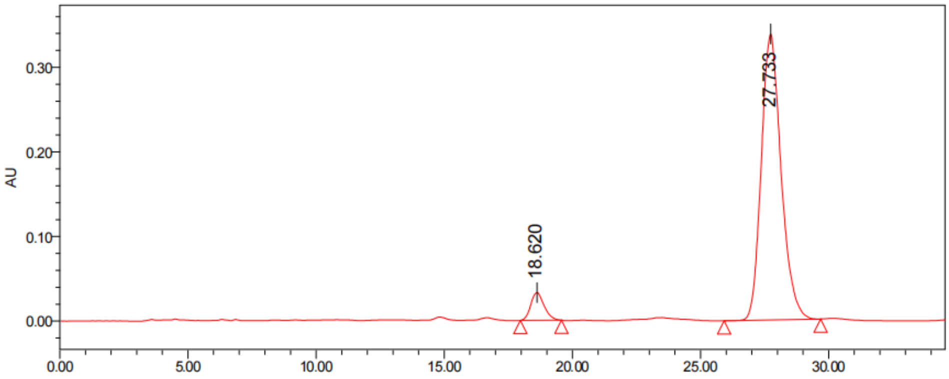

	保留时间 (分钟)	面积	% 面积
1	18.620	1183440	6.12
2	27.733	18154461	93.88

Racemic

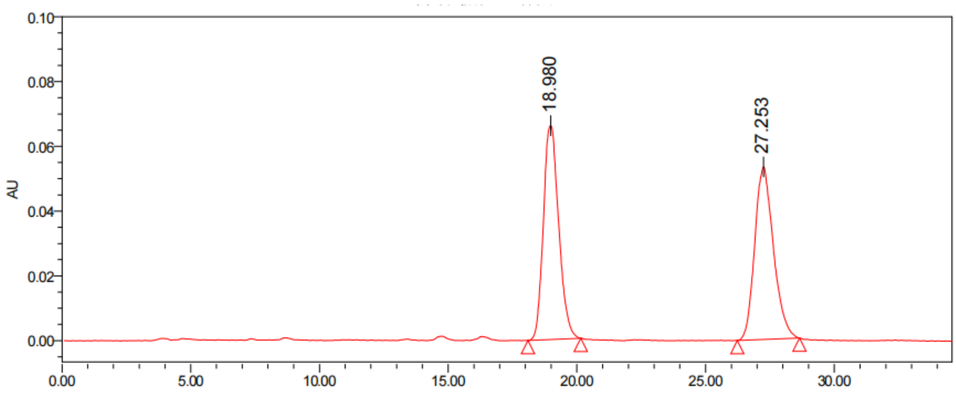

	保留时间 (分钟)	面积	% 面积
1	18.980	2731501	50.36
2	27.253	2692880	49.64

Supplementary Fig. 34. HPLC spectrum of compound **5d**

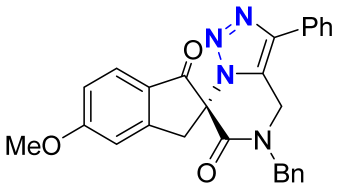

5e

HPLC Conditions
Column: Chiralpak AD-H,
Daicel Chemical Industries Ltd.
Eluent: Hexanes / Isopropanol (70:30)
Flow rate: 1.0 mL/min
Detection: UV 254 nm

Chiral

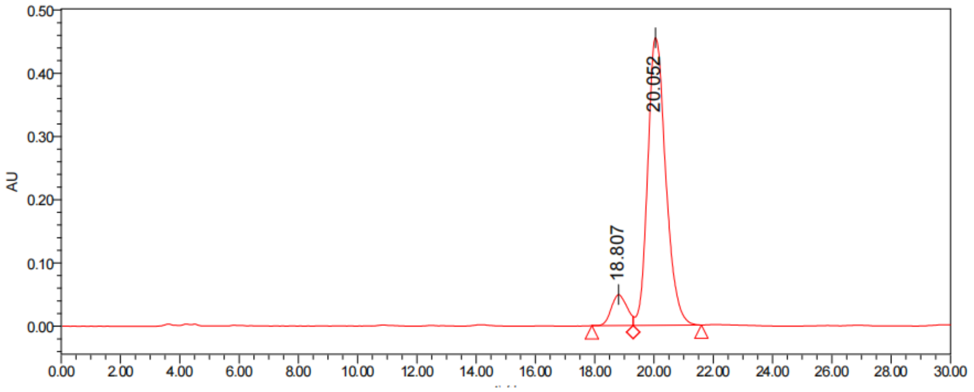

	保留时间 (分钟)	面积	% 面积
1	18.807	1778670	8.47
2	20.052	19217387	91.53

Racemic

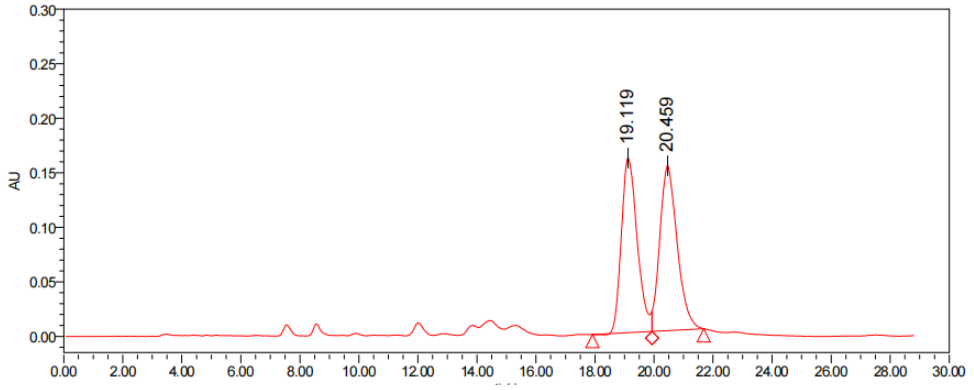

	保留时间 (分钟)	面积	% 面积
1	19.119	6019527	49.31
2	20.459	6188237	50.69

Supplementary Fig. 35. HPLC spectrum of compound **5e**

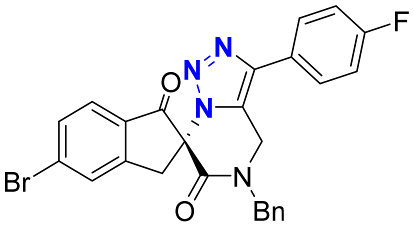

5f

HPLC Conditions
Column: Chiralpak AD-H,
Daicel Chemical Industries Ltd.
Eluent: Hexanes / Isopropanol (70:30)
Flow rate: 1.0 mL/min
Detection: UV 254 nm

Chiral

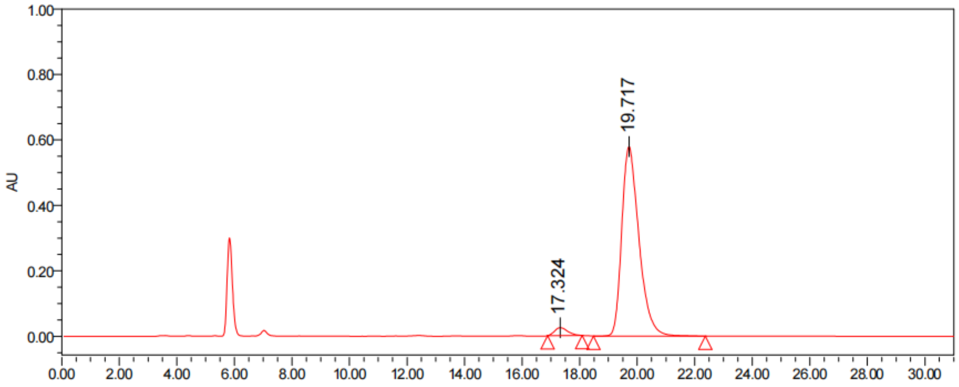

	保留时间 (分钟)	面积	% 面积
1	17.324	762233	3.08
2	19.717	23999958	96.92

Racemic

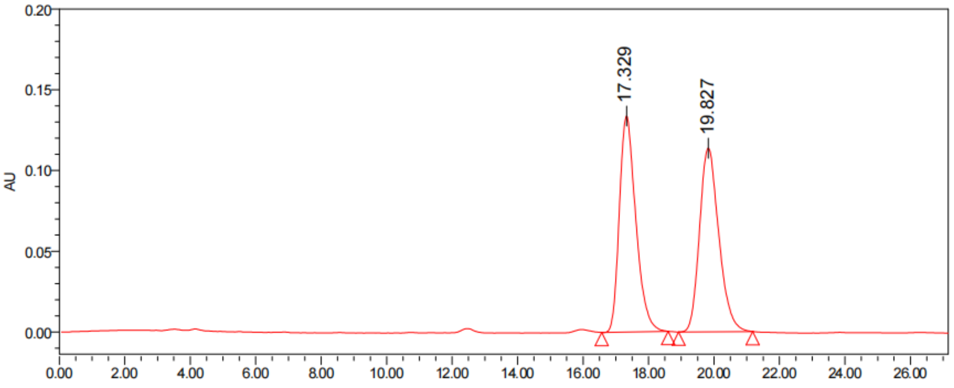

	保留时间 (分钟)	面积	% 面积
1	17.329	4676318	50.10
2	19.827	4658152	49.90

Supplementary Fig. 36. HPLC spectrum of compound **5f**

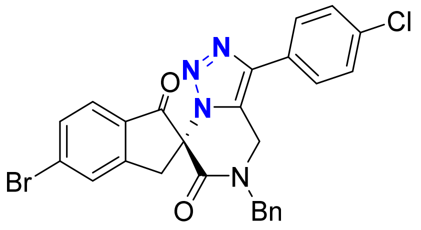

5g

HPLC Conditions
Column: Chiralpak AD-H,
Daicel Chemical Industries Ltd.
Eluent: Hexanes / Isopropanol (70:30)
Flow rate: 1.0 mL/min
Detection: UV 254 nm

Chiral

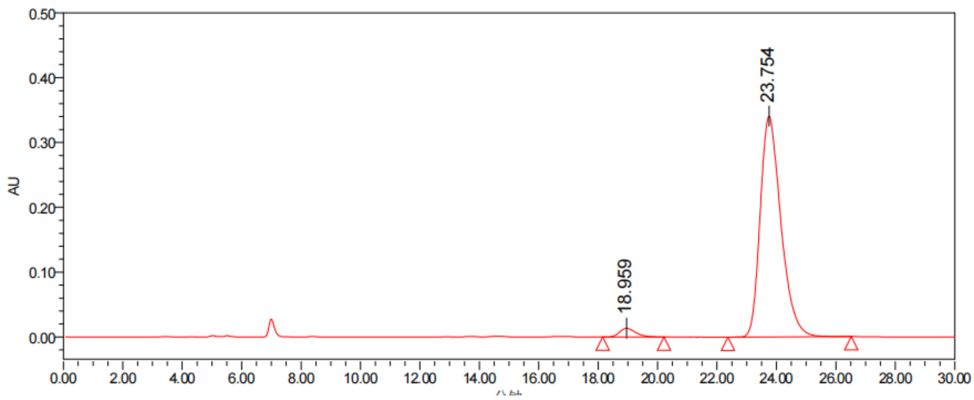

	保留时间 (分钟)	面积	% 面积
1	18.959	509934	3.00
2	23.754	16513130	97.00

Racemic

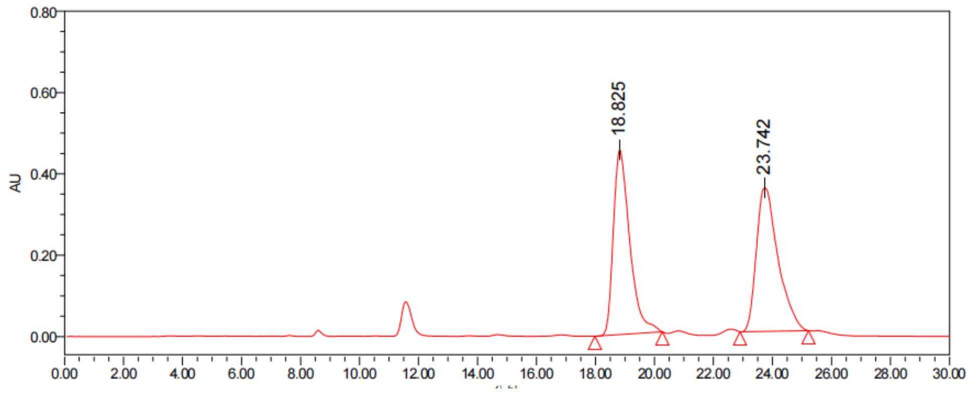

	保留时间 (分钟)	面积	% 面积
1	18.825	17588911	49.36
2	23.742	18045576	50.64

Supplementary Fig. 37. HPLC spectrum of compound **5g**

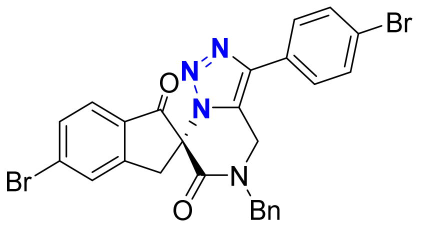

5h

HPLC Conditions
Column: Chiralpak AD-H,
Daicel Chemical Industries Ltd.
Eluent: Hexanes / Isopropanol (70:30)
Flow rate: 1.0 mL/min
Detection: UV 254 nm

Chiral

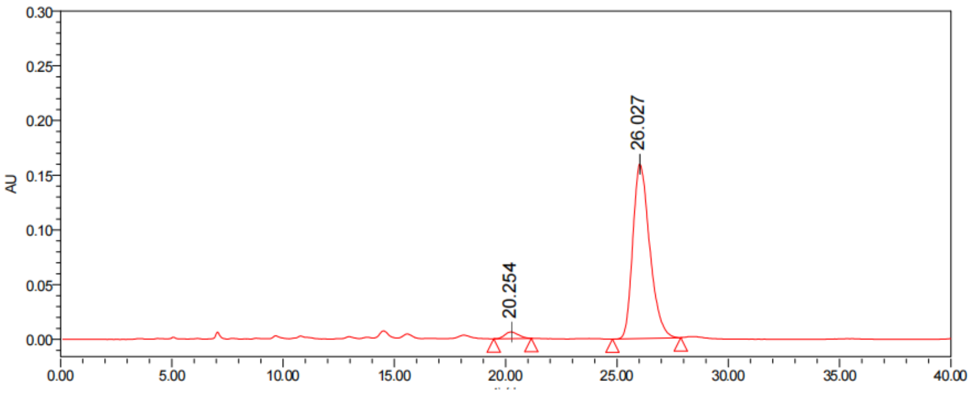

	保留时间 (分钟)	面积	% 面积
1	20.254	254885	2.94
2	26.027	8424208	97.06

Racemic

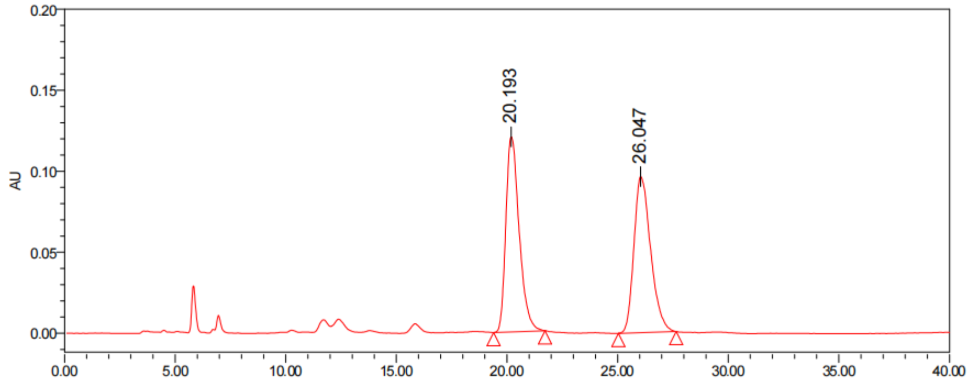

	保留时间 (分钟)	面积	% 面积
1	20.193	5035325	49.88
2	26.047	5059293	50.12

Supplementary Fig. 38. HPLC spectrum of compound **5h**

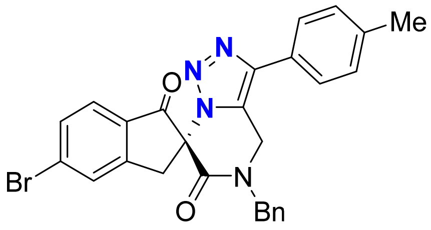

5i

HPLC Conditions
Column: Chiralpak AD-H,
Daicel Chemical Industries Ltd.
Eluent: Hexanes / Isopropanol (70:30)
Flow rate: 1.0 mL/min
Detection: UV 254 nm

Chiral

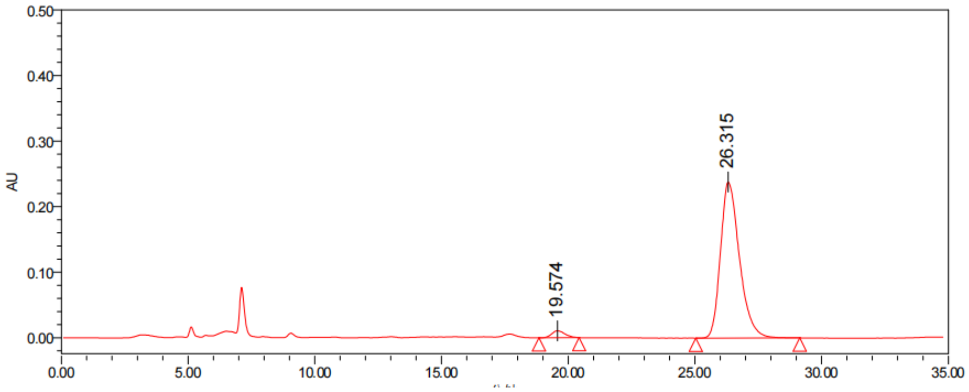

	保留时间 (分钟)	面积	% 面积
1	19.574	384476	3.01
2	26.315	12397453	96.99

Racemic

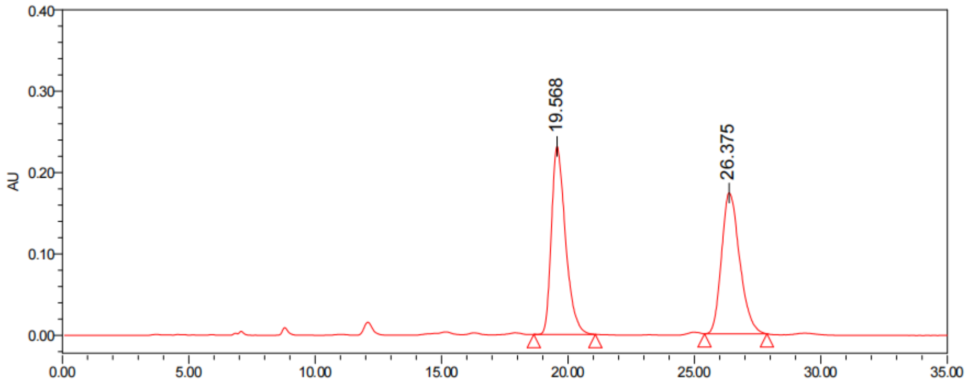

	保留时间 (分钟)	面积	% 面积
1	19.568	8929734	50.75
2	26.375	8666262	49.25

Supplementary Fig. 39. HPLC spectrum of compound **5i**

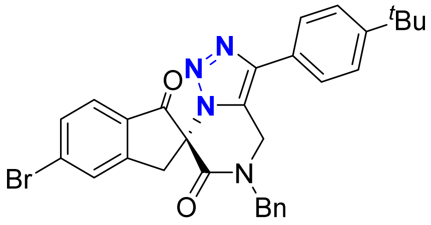

5j

HPLC Conditions
Column: Chiralpak AD-H,
Daicel Chemical Industries Ltd.
Eluent: Hexanes / Isopropanol (70:30)
Flow rate: 1.0 mL/min
Detection: UV 254 nm

Chiral

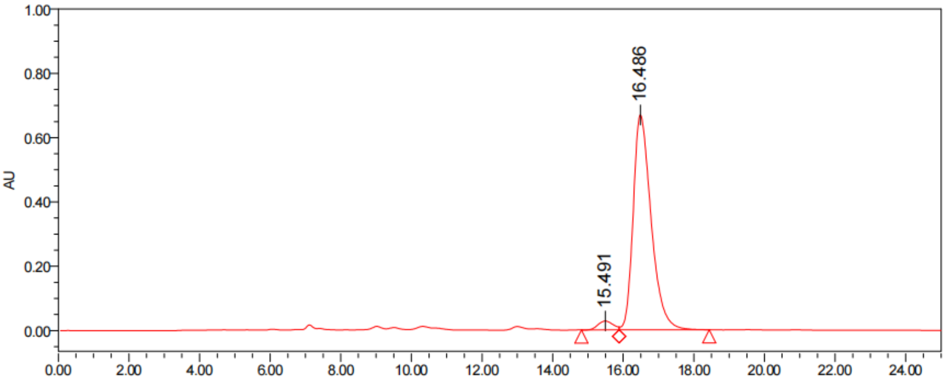

	保留时间 (分钟)	面积	% 面积
1	15.491	810253	3.32
2	16.486	23616505	96.68

Racemic

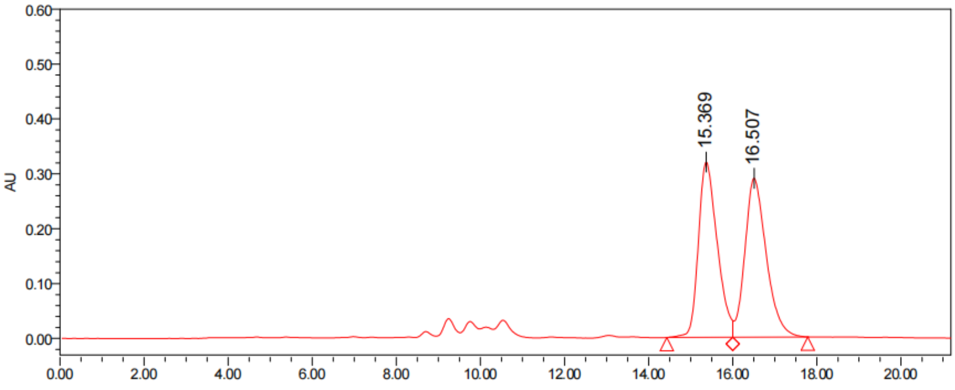

	保留时间 (分钟)	面积	% 面积
1	15.369	9915633	49.19
2	16.507	10242160	50.81

Supplementary Fig. 40. HPLC spectrum of compound **5j**

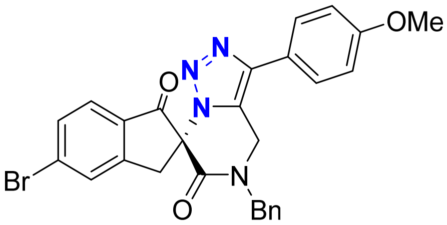

5k

HPLC Conditions
Column: Chiralpak AS-H,
Daicel Chemical Industries Ltd.
Eluent: Hexanes / Isopropanol (60:40)
Flow rate: 1.0 mL/min
Detection: UV 254 nm

Chiral

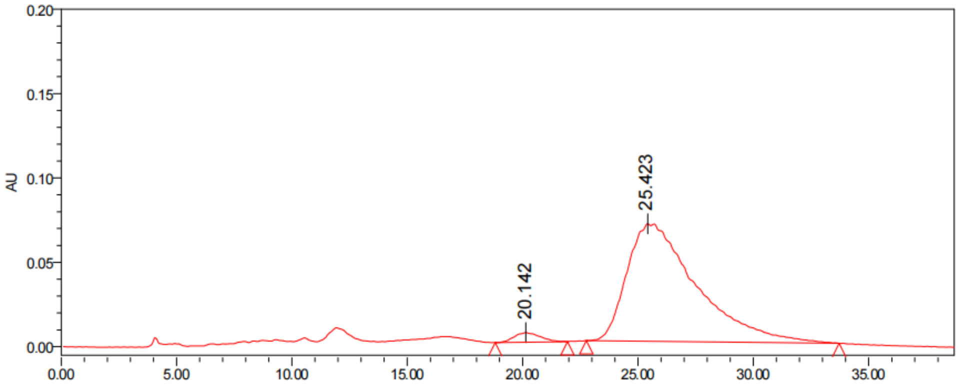

	保留时间 (分钟)	面积	% 面积
1	20.142	438438	2.92
2	25.423	14562375	97.08

Racemic

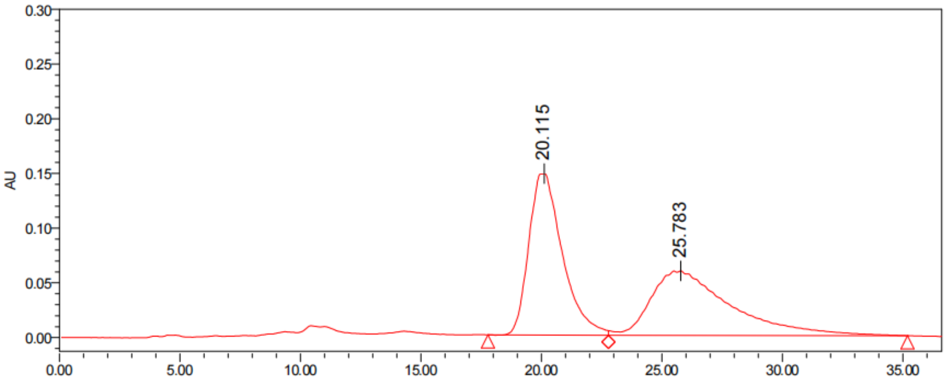

	保留时间 (分钟)	面积	% 面积
1	20.115	14305458	51.41
2	25.783	13519114	48.59

Supplementary Fig. 41. HPLC spectrum of compound **5k**

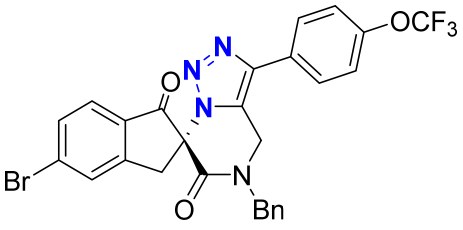

5l

HPLC Conditions
Column: Chiralpak AD-H,
Daicel Chemical Industries Ltd.
Eluent: Hexanes / Isopropanol (70:30)
Flow rate: 1.0 mL/min
Detection: UV 254 nm

Chiral

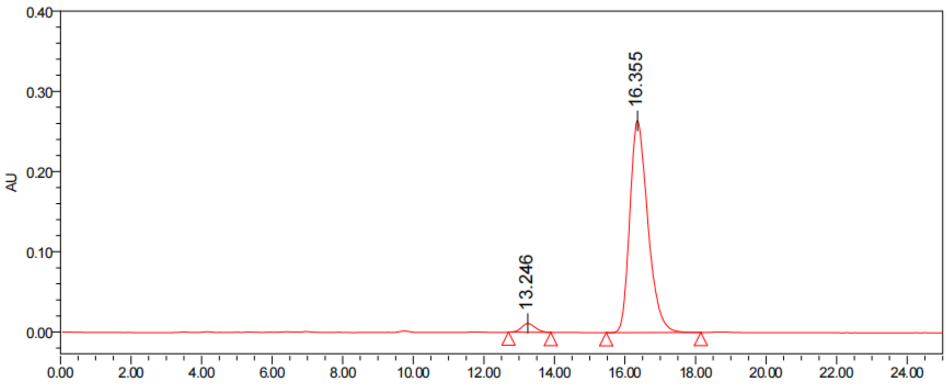

	保留时间 (分钟)	面积	% 面积
1	13.246	295328	3.05
2	16.355	9382239	96.95

Racemic

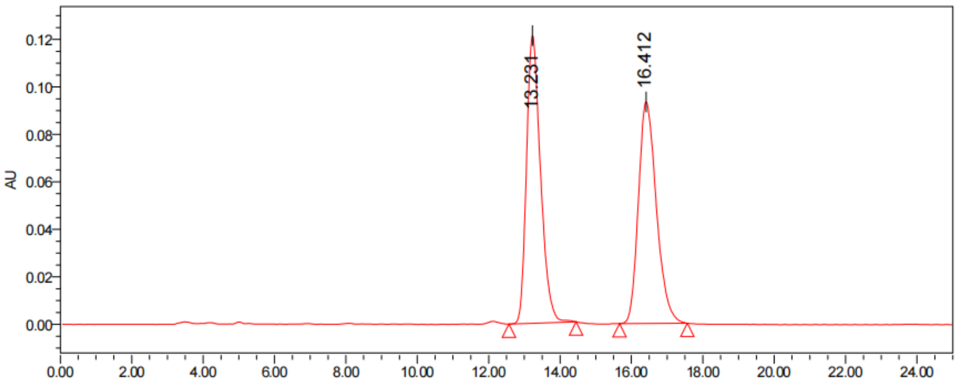

	保留时间 (分钟)	面积	% 面积
1	13.231	3302690	50.27
2	16.412	3266642	49.73

Supplementary Fig. 42. HPLC spectrum of compound **5l**

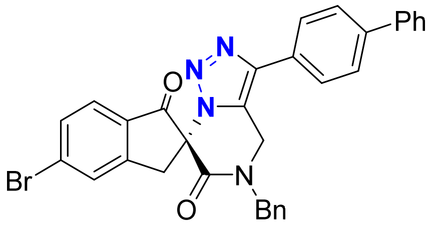

5m

HPLC Conditions
Column: Chiralpak OD-H,
Daicel Chemical Industries Ltd.
Eluent: Hexanes / Isopropanol (50:50)
Flow rate: 1.0 mL/min
Detection: UV 254 nm

Chiral

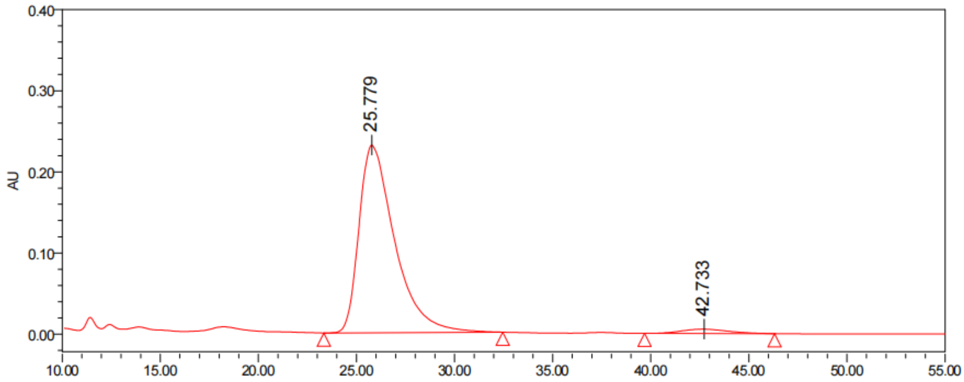

	保留时间 (分钟)	面积	% 面积
1	25.779	29134422	97.07
2	42.733	880338	2.93

Racemic

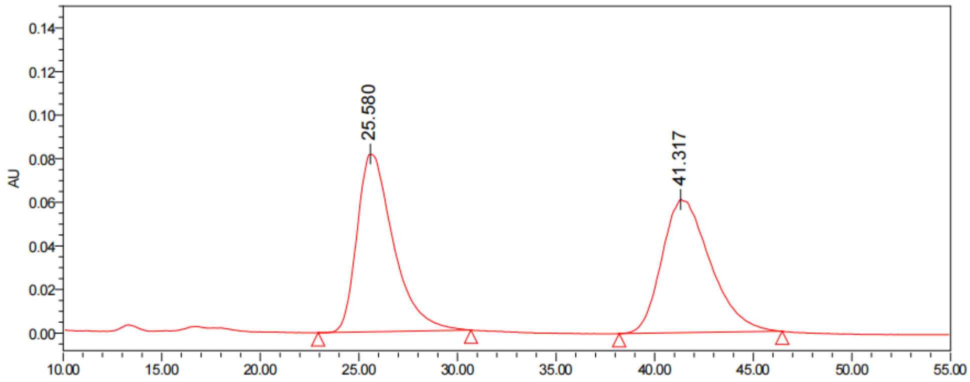

	保留时间 (分钟)	面积	% 面积
1	25.580	10455040	50.02
2	41.317	10444764	49.98

Supplementary Fig. 43. HPLC spectrum of compound **5m**

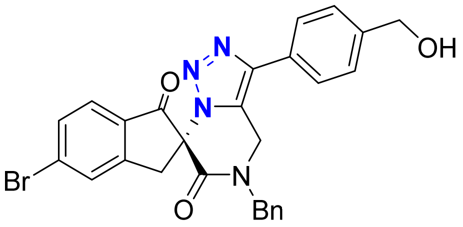

5n

HPLC Conditions
Column: Chiralpak AD-H,
Daicel Chemical Industries Ltd.
Eluent: Hexanes / Isopropanol (60:40)
Flow rate: 1.0 mL/min
Detection: UV 254 nm

Chiral

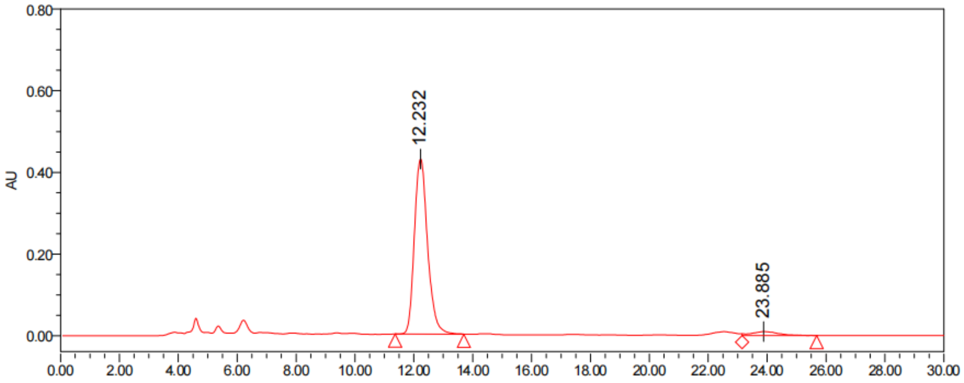

	保留时间 (分钟)	面积	% 面积
1	12.232	13337758	95.90
2	23.885	569973	4.10

Racemic

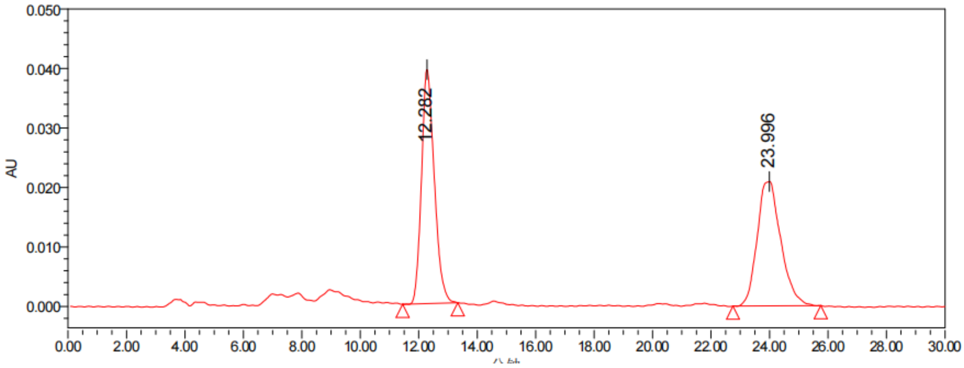

	保留时间 (分钟)	面积	% 面积
1	12.282	1195466	50.42
2	23.996	1175585	49.58

Supplementary Fig. 44. HPLC spectrum of compound **5n**

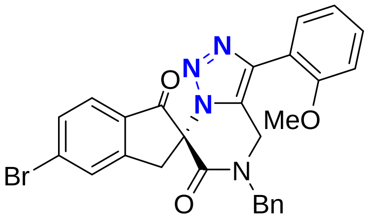

5o

HPLC Conditions
Column: Chiralpak AD-H,
Daicel Chemical Industries Ltd.
Eluent: Hexanes / Isopropanol (60:40)
Flow rate: 1.0 mL/min
Detection: UV 254 nm

Chiral

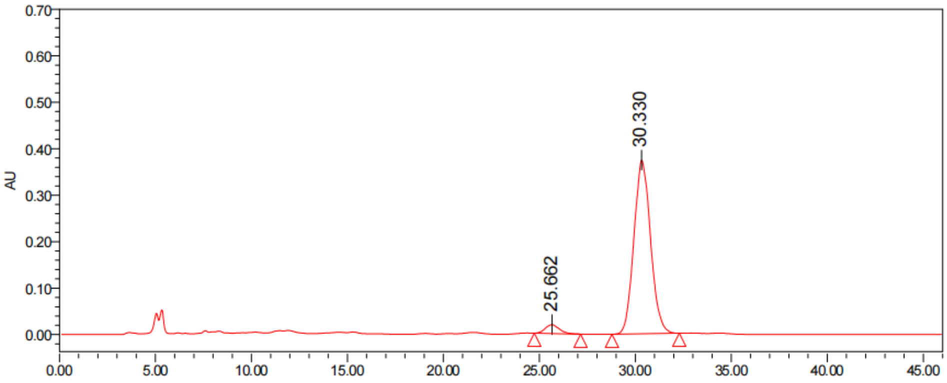

	保留时间 (分钟)	面积	% 面积
1	25.662	956908	4.08
2	30.330	22503222	95.92

Racemic

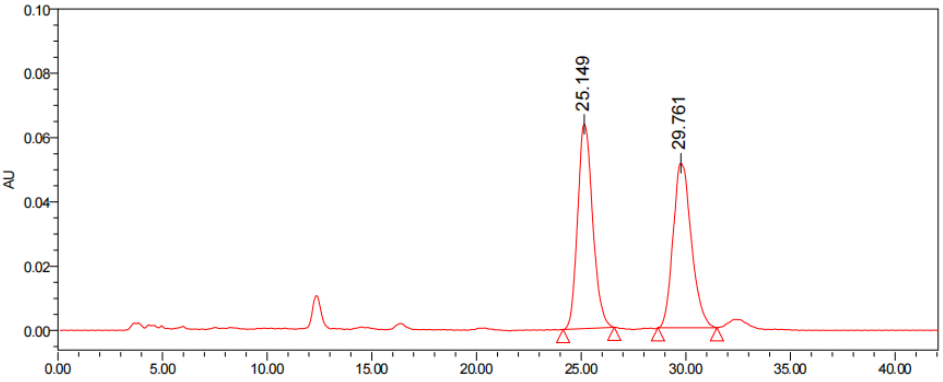

	保留时间 (分钟)	面积	% 面积
1	25.149	3177126	50.45
2	29.761	3120923	49.55

Supplementary Fig. 45. HPLC spectrum of compound **5o**

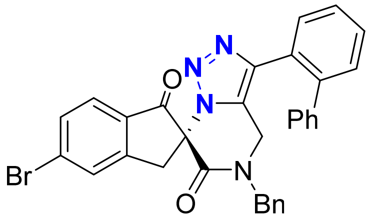

5p

HPLC Conditions
Column: Chiralpak AD-H,
Daicel Chemical Industries Ltd.
Eluent: Hexanes / Isopropanol (80:20)
Flow rate: 1.0 mL/min
Detection: UV 254 nm

Chiral

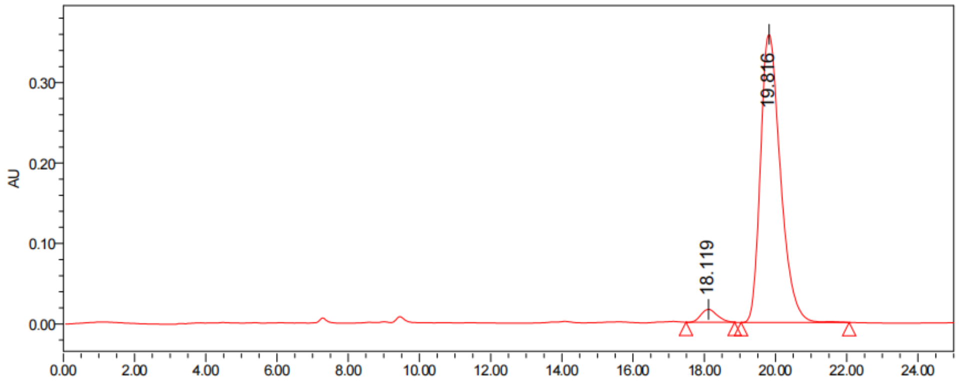

	保留时间 (分钟)	面积	% 面积
1	18.119	510229	3.53
2	19.816	13958002	96.47

Racemic

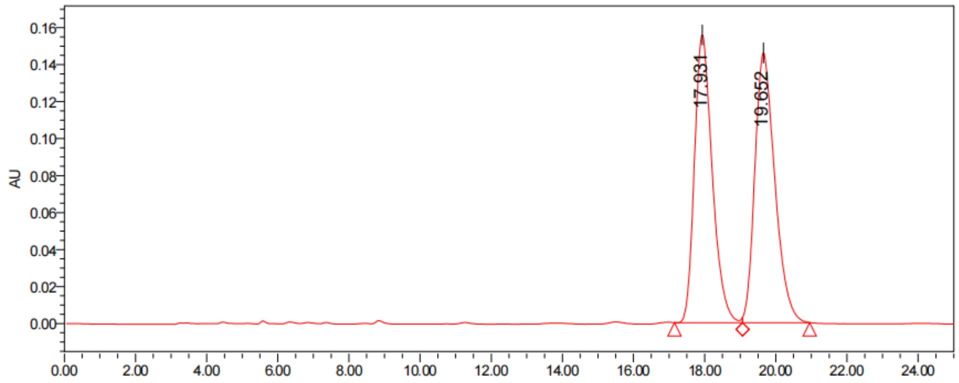

	保留时间 (分钟)	面积	% 面积
1	17.931	5412398	48.73
2	19.652	5695130	51.27

Supplementary Fig. 46. HPLC spectrum of compound **5p**

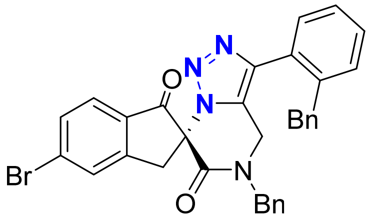

5q

HPLC Conditions
Column: Chiralpak AD-H,
Daicel Chemical Industries Ltd.
Eluent: Hexanes / Isopropanol (50:50)
Flow rate: 1.0 mL/min
Detection: UV 254 nm

Chiral

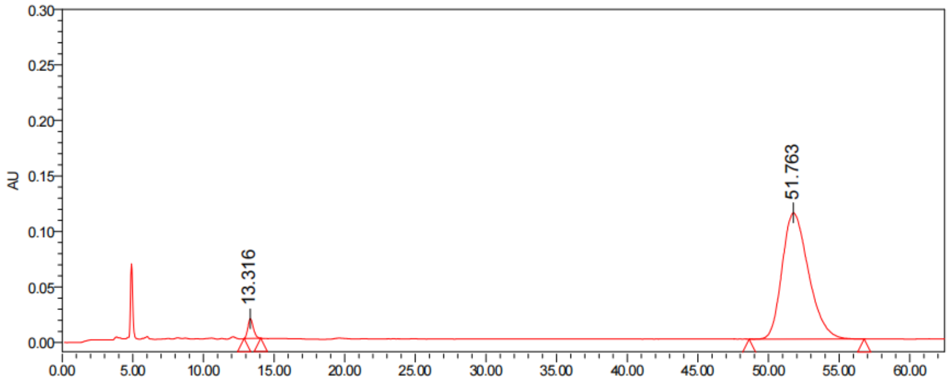

	保留时间 (分钟)	面积	% 面积
1	13.316	479915	3.15
2	51.763	14764945	96.85

Racemic

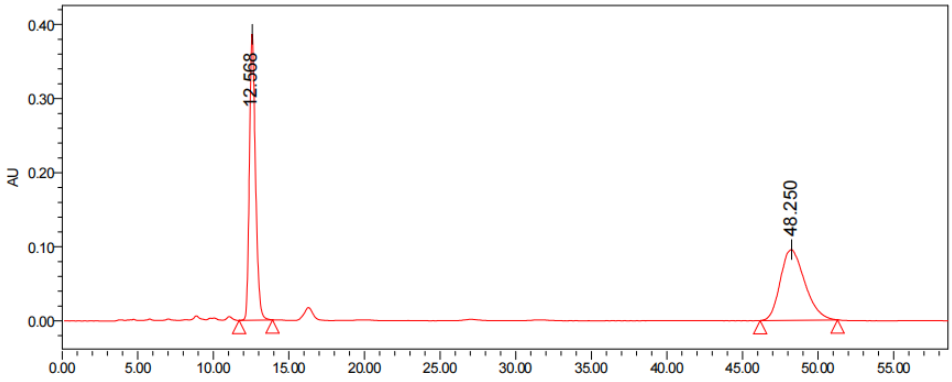

	保留时间 (分钟)	面积	% 面积
1	12.568	10836129	50.24
2	48.250	10733123	49.76

Supplementary Fig. 47. HPLC spectrum of compound **5q**

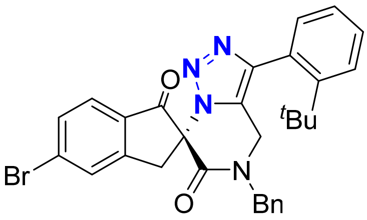

5r

HPLC Conditions
Column: Chiralpak AD-H,
Daicel Chemical Industries Ltd.
Eluent: Hexanes / Isopropanol (50:50)
Flow rate: 1.0 mL/min
Detection: UV 254 nm

Chiral

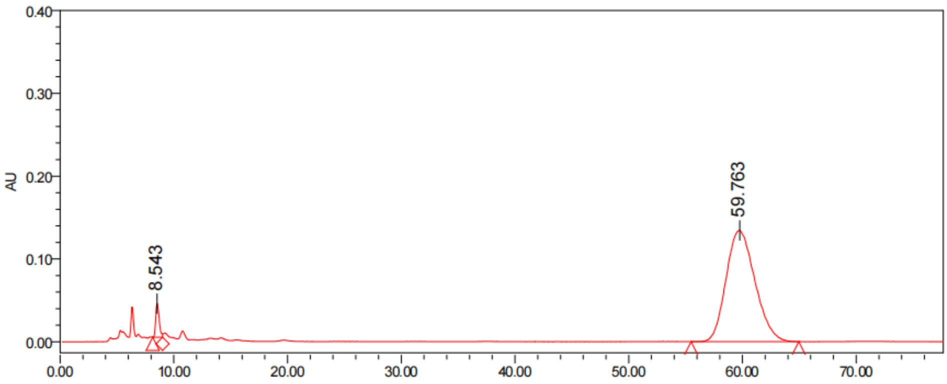

	保留时间 (分钟)	面积	% 面积
1	8.543	851809	3.47
2	59.763	23677266	96.53

Racemic

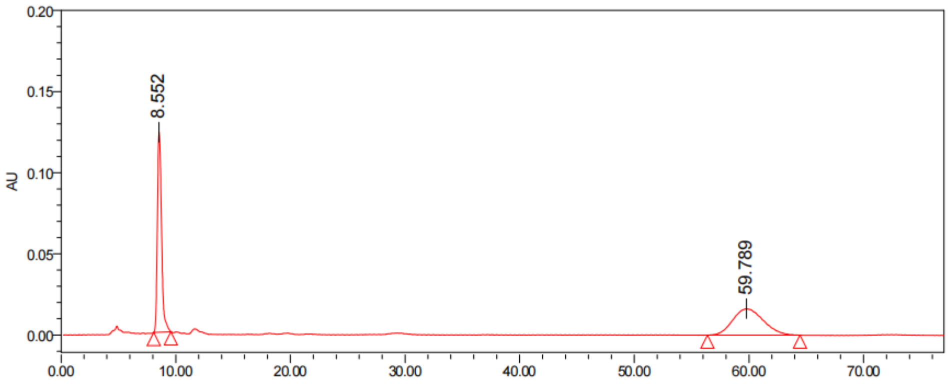

	保留时间 (分钟)	面积	% 面积
1	8.552	3113354	50.83
2	59.789	3011753	49.17

Supplementary Fig. 48. HPLC spectrum of compound **5r**

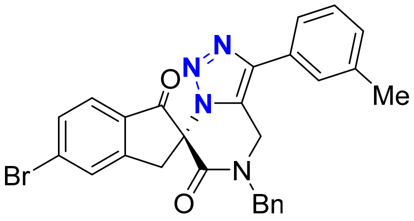

5s

HPLC Conditions
Column: Chiralpak AD-H,
Daicel Chemical Industries Ltd.
Eluent: Hexanes / Isopropanol (70:30)
Flow rate: 1.0 mL/min
Detection: UV 254 nm

Chiral

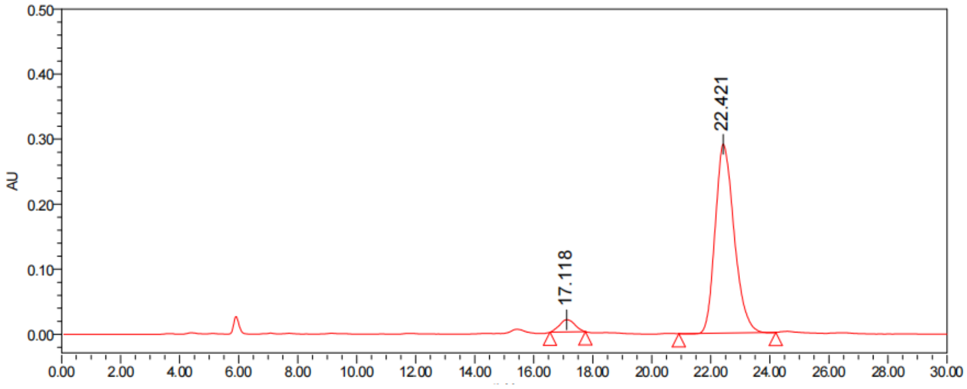

	保留时间 (分钟)	面积	% 面积
1	17.118	688303	5.02
2	22.421	13015169	94.98

Racemic

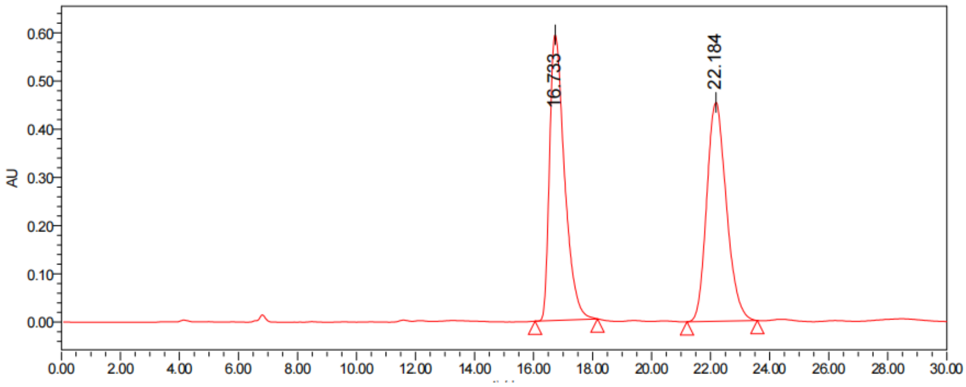

	保留时间 (分钟)	面积	% 面积
1	16.733	20839126	49.77
2	22.184	21031166	50.23

Supplementary Fig. 49. HPLC spectrum of compound **5s**

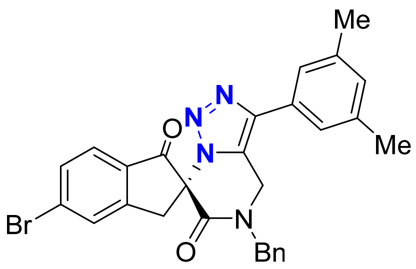

5t

HPLC Conditions
Column: Chiralpak AD-H,
Daicel Chemical Industries Ltd.
Eluent: Hexanes / Isopropanol (50:50)
Flow rate: 1.0 mL/min
Detection: UV 254 nm

Chiral

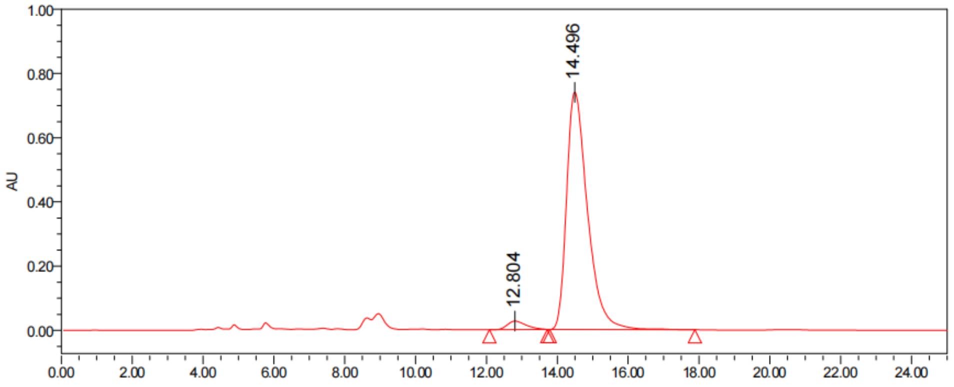

	保留时间 (分钟)	面积	% 面积
1	12.804	969436	3.09
2	14.496	30381592	96.91

Racemic

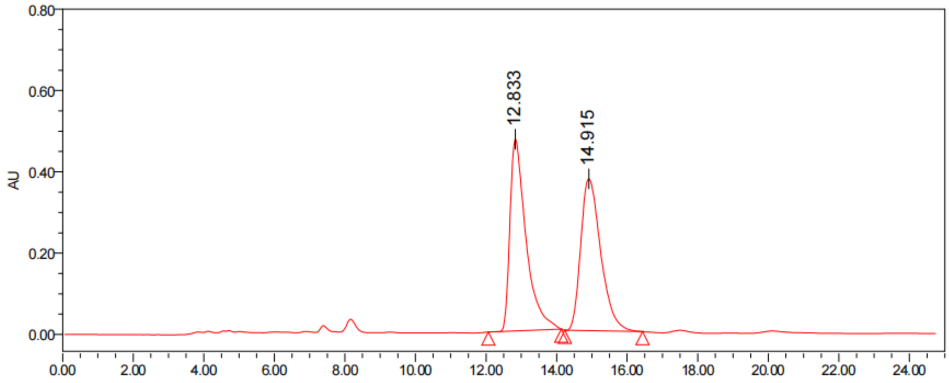

	保留时间 (分钟)	面积	% 面积
1	12.833	15004483	50.44
2	14.915	14743255	49.56

Supplementary Fig. 50. HPLC spectrum of compound **5t**

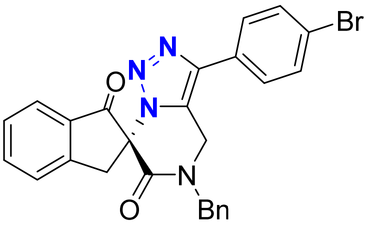

5u

HPLC Conditions
Column: Chiralpak AD-H,
Daicel Chemical Industries Ltd.
Eluent: Hexanes / Isopropanol (70:30)
Flow rate: 1.0 mL/min
Detection: UV 254 nm

Chiral

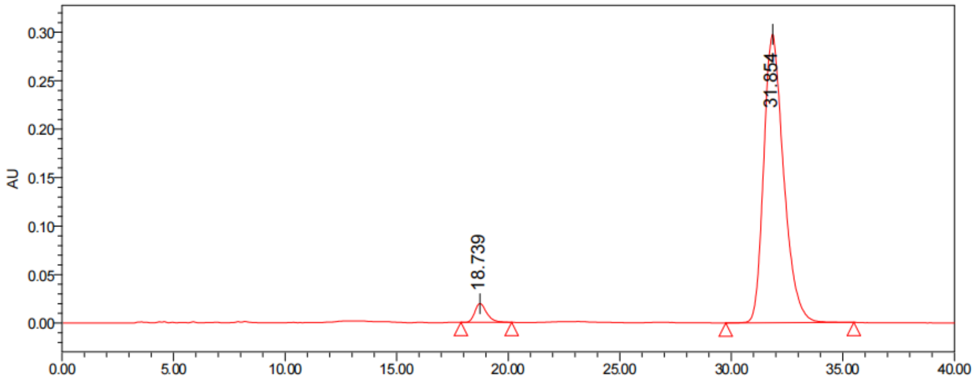

	保留时间 (分钟)	面积	% 面积
1	18.739	731279	3.80
2	31.854	18523248	96.20

Racemic

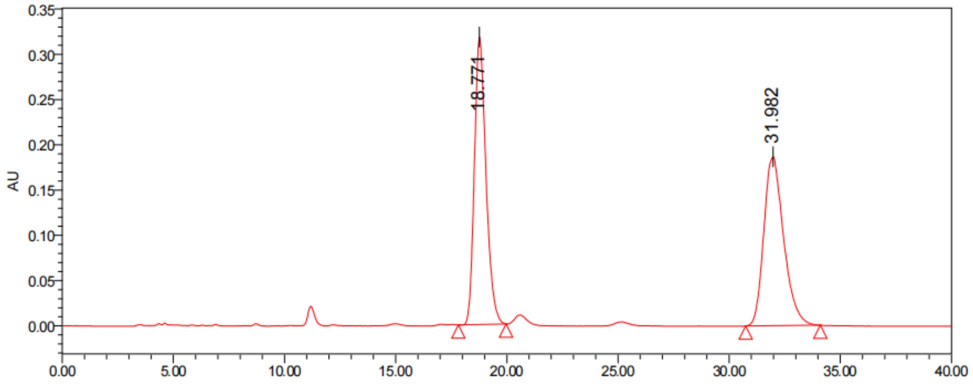

	保留时间 (分钟)	面积	% 面积
1	18.771	11466928	49.80
2	31.982	11558101	50.20

Supplementary Fig. 51. HPLC spectrum of compound **5u**

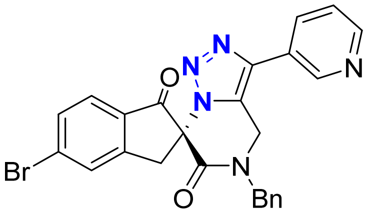

5v

HPLC Conditions
Column: Chiralpak AD-H,
Daicel Chemical Industries Ltd.
Eluent: Hexanes / Isopropanol (60:40)
Flow rate: 1.0 mL/min
Detection: UV 254 nm

Chiral

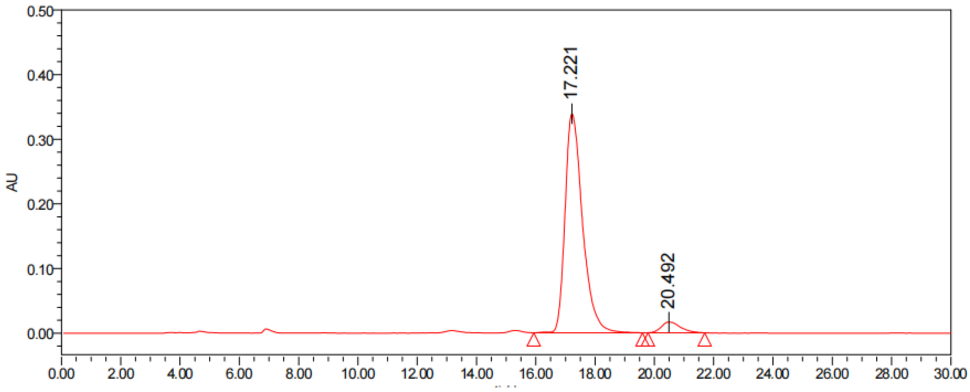

	保留时间 (分钟)	面积	% 面积
1	17.221	13939379	94.99
2	20.492	735099	5.01

Racemic

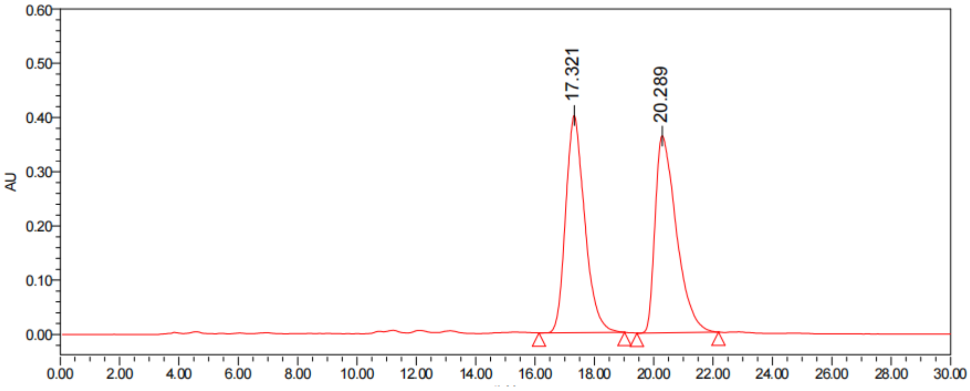

	保留时间 (分钟)	面积	% 面积
1	17.321	17885746	49.76
2	20.289	18060970	50.24

Supplementary Fig. 52. HPLC spectrum of compound **5v**

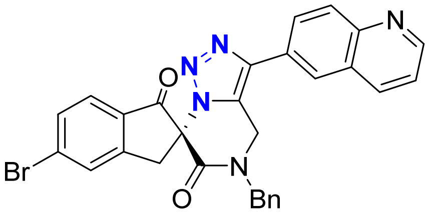

5w

HPLC Conditions
Column: Chiralpak AD-H,
Daicel Chemical Industries Ltd.
Eluent: Hexanes / Isopropanol (60:40)
Flow rate: 1.0 mL/min
Detection: UV 254 nm

Chiral

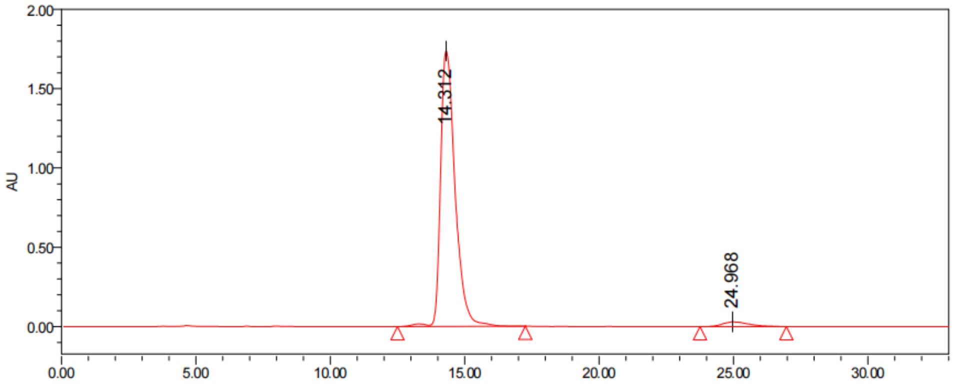

	保留时间 (分钟)	面积	% 面积
1	14.312	65219657	97.05
2	24.968	1979095	2.95

Racemic

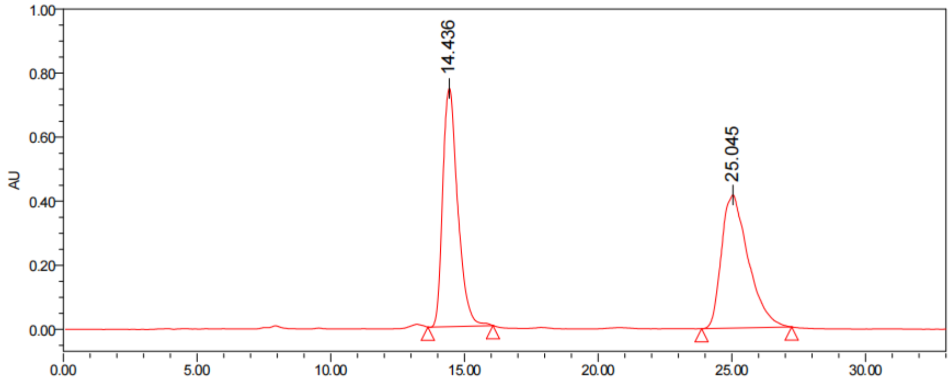

	保留时间 (分钟)	面积	% 面积
1	14.436	28957141	49.95
2	25.045	29017213	50.05

Supplementary Fig. 53. HPLC spectrum of compound **5w**

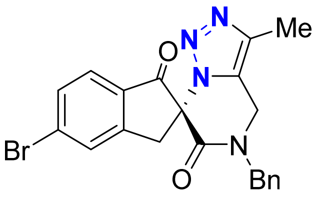

5x

HPLC Conditions
Column: Chiralpak AD-H,
Daicel Chemical Industries Ltd.
Eluent: Hexanes / Isopropanol (70:30)
Flow rate: 1.0 mL/min
Detection: UV 254 nm

Chiral

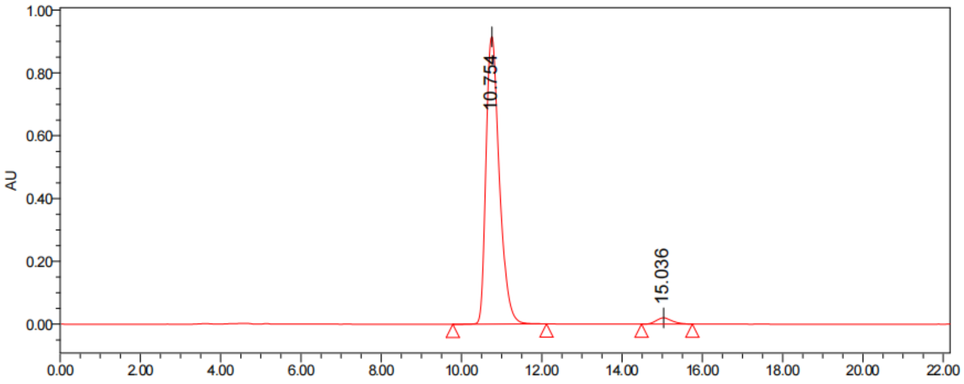

	保留时间 (分钟)	面积	% 面积
1	10.754	20945640	97.47
2	15.036	542895	2.53

Racemic

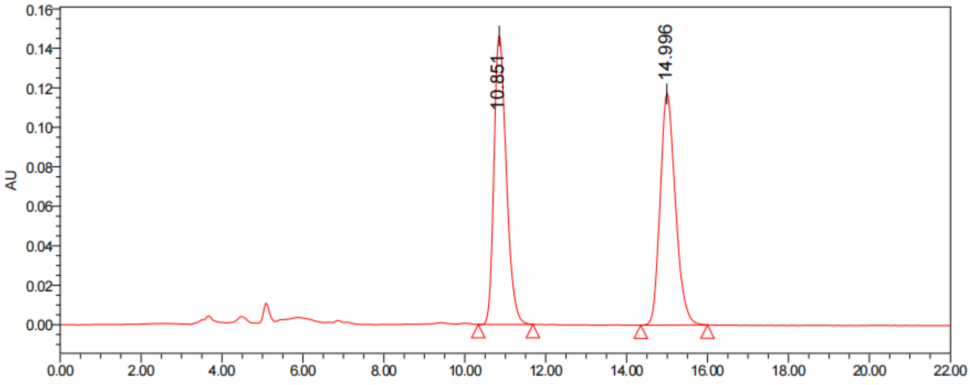

	保留时间 (分钟)	面积	% 面积
1	10.851	3066245	50.05
2	14.996	3059750	49.95

Supplementary Fig. 54. HPLC spectrum of compound **5x**

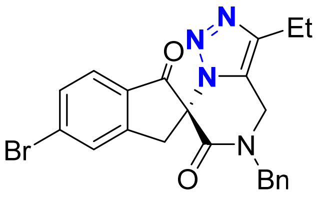

5y

HPLC Conditions
Column: Chiralpak AD-H,
Daicel Chemical Industries Ltd.
Eluent: Hexanes / Isopropanol (70:30)
Flow rate: 1.0 mL/min
Detection: UV 254 nm

Chiral

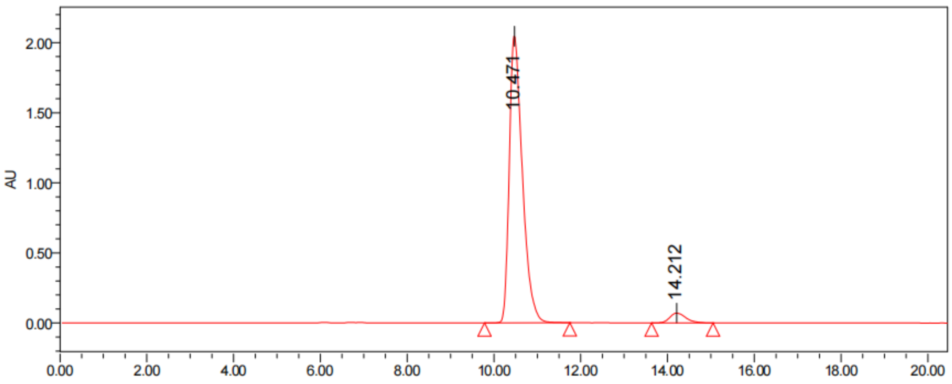

	保留时间 (分钟)	面积	% 面积
1	10.471	42894610	95.99
2	14.212	1792042	4.01

Racemic

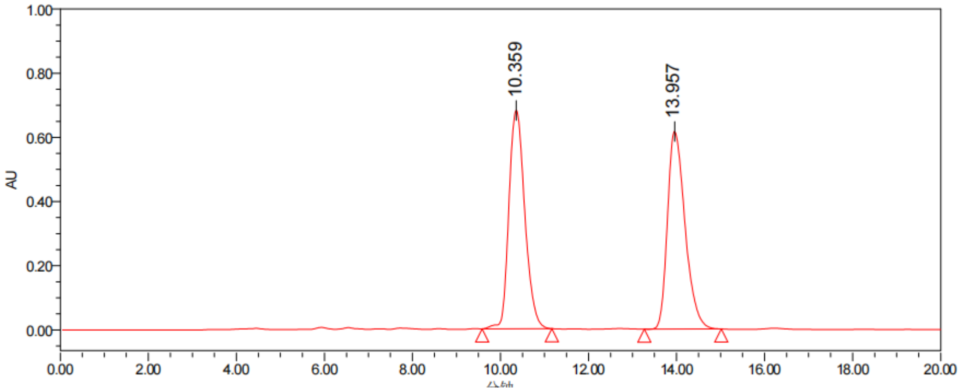

	保留时间 (分钟)	面积	% 面积
1	10.359	17131643	50.05
2	13.957	17100463	49.95

Supplementary Fig. 55. HPLC spectrum of compound **5y**

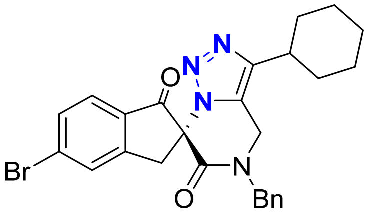

5z

HPLC Conditions
Column: Chiralpak AD-H,
Daicel Chemical Industries Ltd.
Eluent: Hexanes / Isopropanol (70:30)
Flow rate: 1.0 mL/min
Detection: UV 254 nm

Chiral

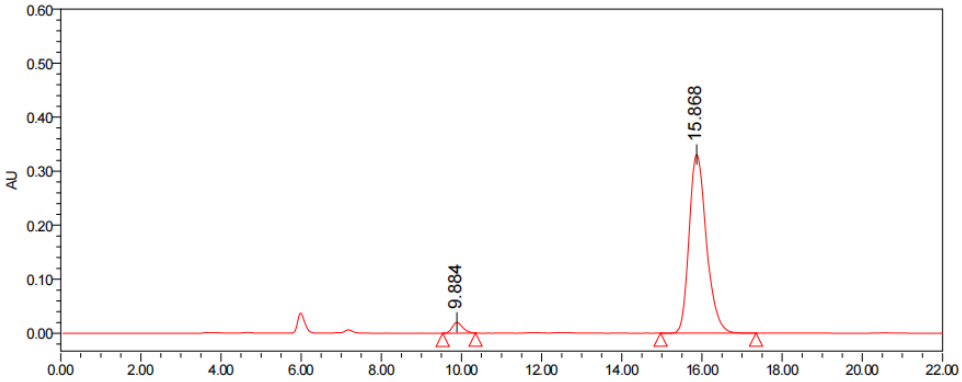

	保留时间 (分钟)	面积	% 面积
1	9.884	362022	3.52
2	15.868	9935228	96.48

Racemic

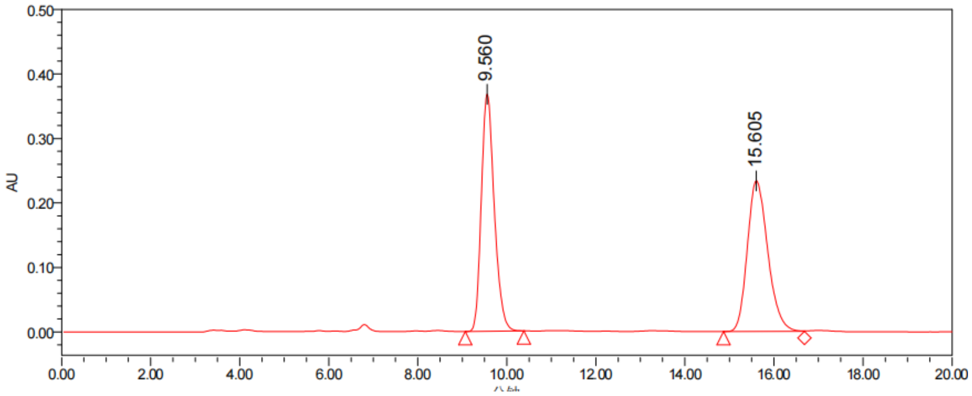

	保留时间 (分钟)	面积	% 面积
1	9.560	7440329	49.89
2	15.605	7472805	50.11

Supplementary Fig. 56. HPLC spectrum of compound **5z**

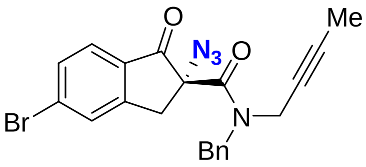

6

HPLC Conditions
Column: Chiralpak AD-H,
Daicel Chemical Industries Ltd.
Eluent: Hexanes / Isopropanol (90:10)
Flow rate: 1.0 mL/min
Detection: UV 254 nm

Chiral

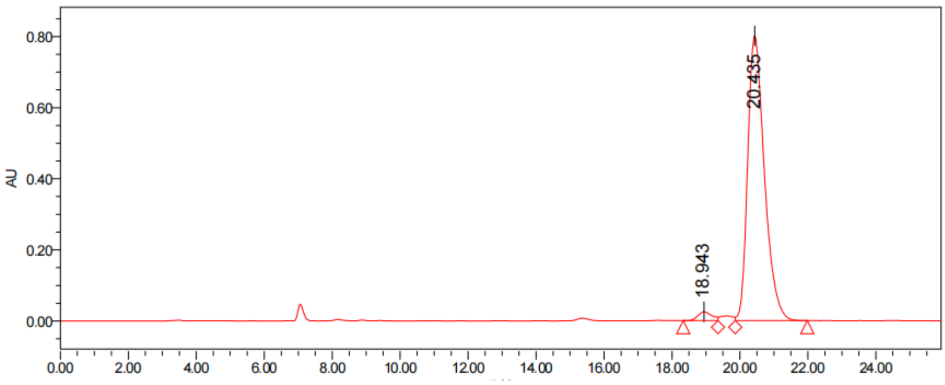

	保留时间 (分钟)	面积	% 面积
1	18.943	699078	2.49
2	20.435	27353426	97.51

Racemic

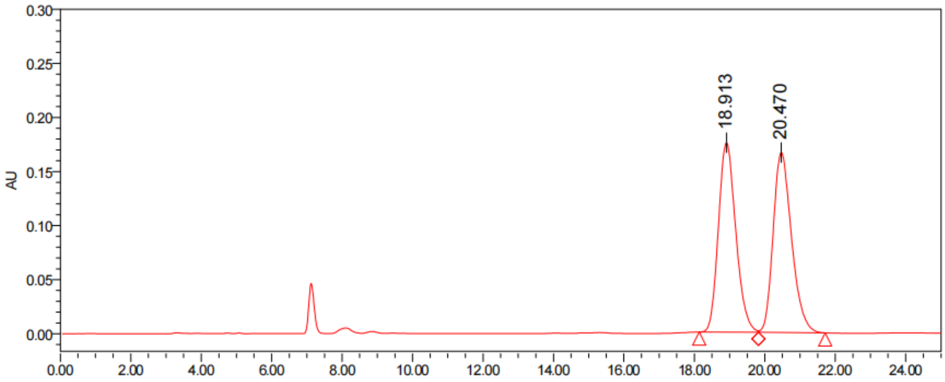

	保留时间 (分钟)	面积	% 面积
1	18.913	6004168	49.79
2	20.470	6055205	50.21

Supplementary Fig. 57. HPLC spectrum of compound **6**

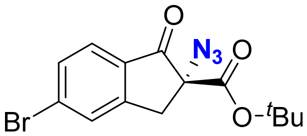

8a

HPLC Conditions
Column: Chiralpak AD-H,
Daicel Chemical Industries Ltd.
Eluent: Hexanes / Isopropanol (99:1)
Flow rate: 1.0 mL/min
Detection: UV 254 nm

Chiral

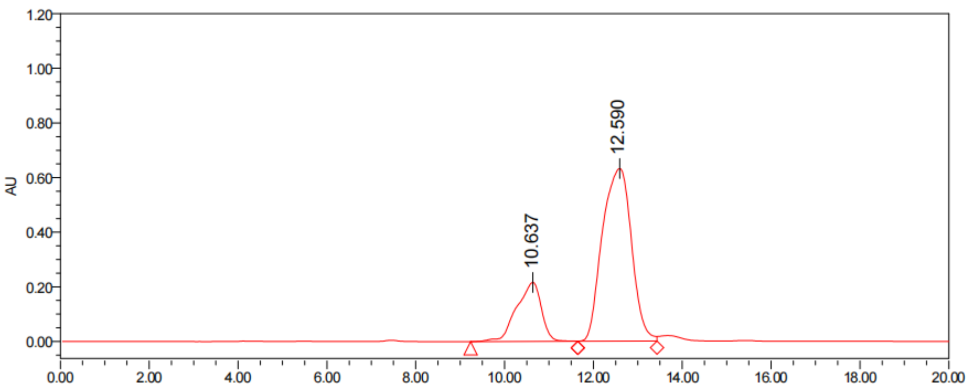

	保留时间 (分钟)	面积	% 面积
1	10.637	8371847	22.49
2	12.590	28846765	77.51

Racemic

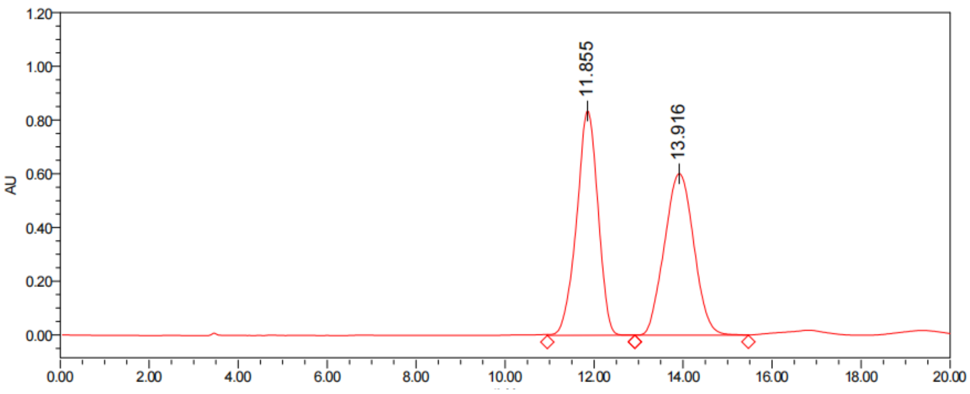

	保留时间 (分钟)	面积	% 面积
1	11.855	27901671	50.08
2	13.916	27814411	49.92

Supplementary Fig. 58. HPLC spectrum of compound **8a**

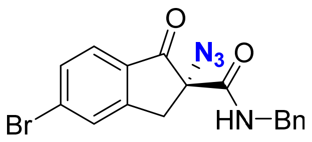

8b

HPLC Conditions
Column: Chiralpak AD-H,
Daicel Chemical Industries Ltd.
Eluent: Hexanes / Isopropanol (80:20)
Flow rate: 1.0 mL/min
Detection: UV 254 nm

Chiral

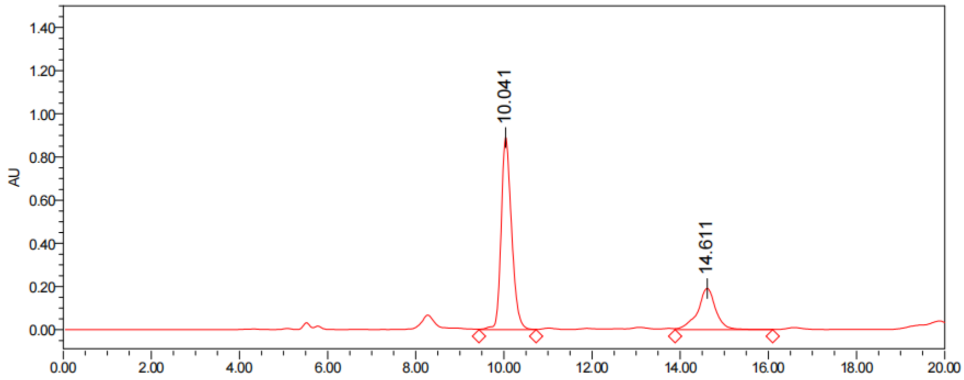

	保留时间 (分钟)	面积	% 面积
1	10.041	14615121	73.05
2	14.611	5392312	26.95

Racemic

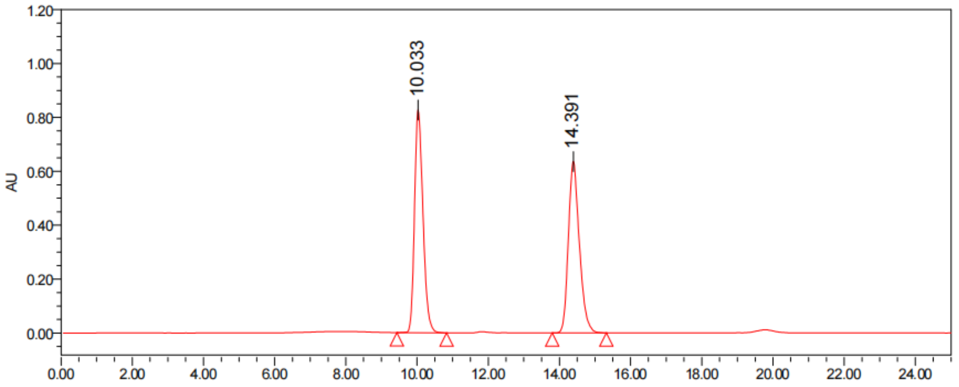

	保留时间 (分钟)	面积	% 面积
1	10.033	13629324	49.91
2	14.391	13676714	50.09

Supplementary Fig. 59. HPLC spectrum of compound **8b**

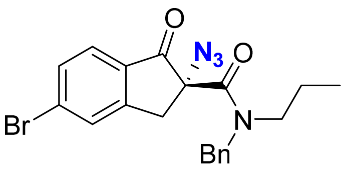

8c

HPLC Conditions
Column: Chiralpak AD-H,
Daicel Chemical Industries Ltd.
Eluent: Hexanes / Isopropanol (90:10)
Flow rate: 1.0 mL/min
Detection: UV 254 nm

Chiral

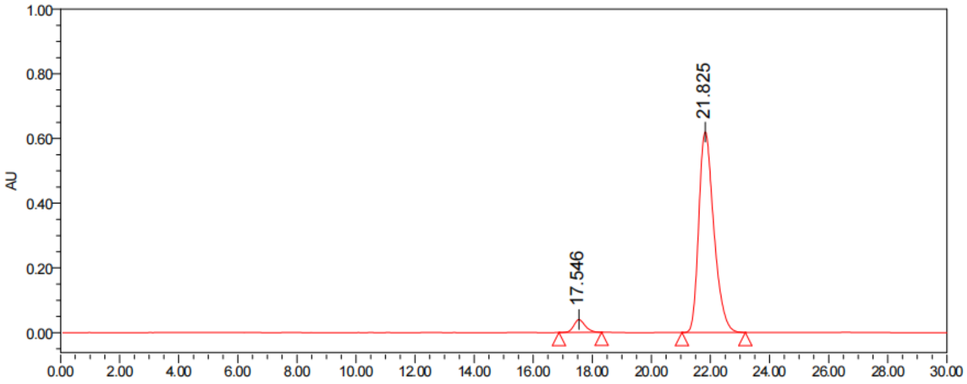

	保留时间 (分钟)	面积	% 面积
1	17.546	1057531	4.59
2	21.825	21977714	95.41

Racemic

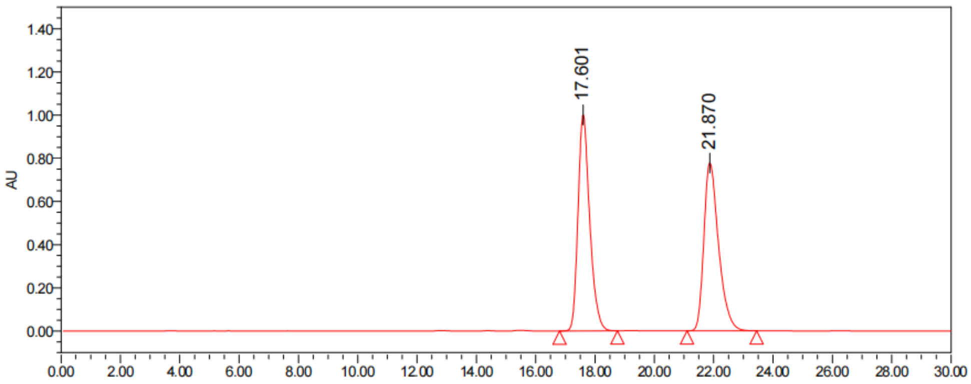

	保留时间 (分钟)	面积	% 面积
1	17.601	27430558	50.62
2	21.870	26754989	49.38

Supplementary Fig. 60. HPLC spectrum of compound **8c**

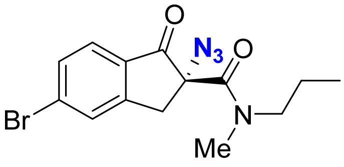

8d

HPLC Conditions
Column: Chiralpak AD-H,
Daicel Chemical Industries Ltd.
Eluent: Hexanes / Isopropanol (80:20)
Flow rate: 1.0 mL/min
Detection: UV 254 nm

Chiral

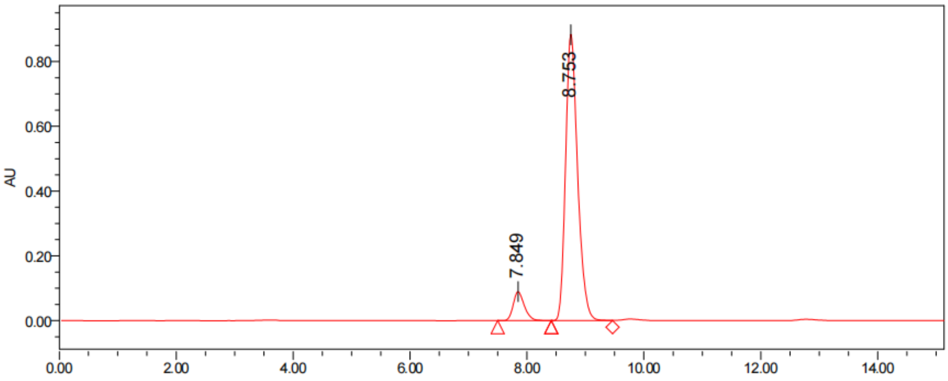

	保留时间 (分钟)	面积	% 面积
1	7.849	1174435	8.51
2	8.753	12622371	91.49

Racemic

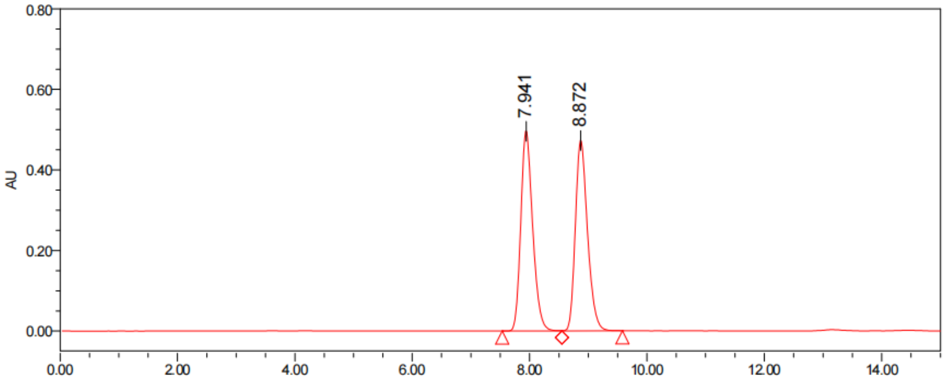

	保留时间 (分钟)	面积	% 面积
1	7.941	6969836	50.56
2	8.872	6814648	49.44

Supplementary Fig. 61. HPLC spectrum of compound **8d**

5. Supplementary References

[1] Ding, T., Jiang, L., Yang, J., Xu, Y., Wang, G. & Yi, W. Highly carbon-selective monofluoromethylation of β-ketoesters with fluoromethyl iodide. *Org. Lett.* **21**, 6025-6028 (2019).

[2] Poeira, D. L. et al. Hypervalent iodine (III) reagents with transferable primary amines: Structure and reactivity on the electrophilic α-amination of stabilized enolates. *Org. Lett.* **24**, 776-781 (2022).

[3] Yao, C. et al. HDAC1/MAO-B dual inhibitors against alzheimer's disease: Design, synthesis and biological evaluation of N-propargylamine-hydroxamic acid/O-aminobenzamide hybrids. *Bioorg. Chem.* **122**, 105724 (2022).

[4] Rzayev, J., Zhang, Z., Durand, N. & Soulé, J. F. Upgrading carbazolyl-derived phosphine ligands using Rh (I)-catalyzed P (III)-directed C–H bond alkylation for catalytic CO_2-fixation reactions. *Org. Lett.* **24**, 6755-6760 (2022).

[5] Das, A., Buzzetti, L., Puriņš, M. & Waser, J. Palladium-catalyzed trans-hydroalkoxylation: Counterintuitive use of an aryl iodide additive to promote C–H bond formation. *ACS Catal.* **12**, 7565-7570 (2022).

[6] Vita, M. V. & Waser, J. Azidation of β-keto esters and silyl enol ethers with a benziodoxole reagent. *Org. Lett.* **15**, 3246-3249 (2013).

[7] Chen, Y.-X. et al. Azidobenziodazolones as azido sources for the enantioselective copper-catalyzeda zidation of N-unprotected 3-trifluoromethylated oxindoles. *Org. Lett.* **25**, 2739-2744 (2023).

[8] Deng, Q.-H. et al. Enantioselective iron-catalyzed azidation of β-keto esters and oxindoles. *J. Am. Chem. Soc.* **135**, 5356-5359 (2013).

[9] Zheng, L.-S., Wei, Y.-L., Jiang, K.-Z., Deng, Y., Zheng, Z.-J. & Xu, L.-W. Enantioselective fluorination of β-ketoamides catalyzed by Ar-BINMOL-derived salan-copper complex. *Adv. Synth. Catal.* **356**, 3769-3776 (2014).

[10] Lin, C.-Z. et al. Enantioselective synthesis of 3a-azido-pyrroloindolines by copper-catalyzed asymmetric dearomative azidation of tryptamines. *Chem. Commun.* **59**, 7831-7834 (2023).

[11] Guo, J. et al. Iodine-catalyzed azidation/cyclization cascade approach to 3a-azidofuroindolines and -pyrroloindolines under mild conditions. *Eur. J. Org. Chem.* **2017**, 4773-4777 (2017).

[12] Zhang, P., Sun, W., Li, G., Hong, L. & Wang, R. Copper-catalyzed cascade azidation–cyclization of tryptophols and tryptamines. *Chem. Commun* **51**, 12293-12296 (2015).

[13] Scattolin, T., Bouayad-Gervais, S. & Schoenebeck, F. Straightforward access to N-trifluoromethyl amides, carbamates, thiocarbamates and ureas. *Nature* **573**, 102-107 (2019).

[14] Tu, H.-F., Zhang, X., Zheng, C., Zhu, M. & You, S.-L. Enantioselective dearomative prenylation of indole derivatives. *Nature Catal.* **1**, 601-608 (2018).

[15] Grimme, S., Ehrlich, S. & Goerigk, L. Effect of the damping function in dispersion corrected density functional theory. *J. Comput. Chem.* **32**, 1456-1465 (2011).
